# Photocatalyst-free, visible-light-induced regio- and stereoselective synthesis of phosphorylated enamines from *N*-allenamides *via* [1,3]-sulfonyl shift at room temperature†

**DOI:** 10.1039/d4sc05190d

**Published:** 2024-10-03

**Authors:** Jia-Dong Guo, Feven-Alemu Korsaye, Dorian Schutz, Ilaria Ciofini, Laurence Miesch

**Affiliations:** a Equipe Synthèse Organique et Phytochimie, Institut de Chimie, CNRS-UdS UMR 7177, 4 rue Blaise Pascal, CS 90032 67081 Strasbourg France lmiesch@unistra.fr; b Chemical Theory and Modelling Group, Chimie ParisTech, PSL University, CNRS, Institute of Chemistry for Life and Health Sciences F-75005 Paris France ilaria.ciofini@chimieparistech.psl.eu

## Abstract

Herein, we report the first visible-light-induced strategy for the rapid synthesis of densely functionalized α- and γ-phosphorylated β-sulfonyl enamines in a regio- and stereoselective manner from *N*-sulfonyl allenamides and H-phosphine oxides. The transformation displays a broad substrate scope, while operating at room temperature under photocatalyst- and additive-free conditions. In this atom-economical process, either terminal or substituted *N*-sulfonyl allenamides trigger an unprecedented *N*-to-*C* [1,3]-sulfonyl shift, relying on a dual radical allyl resonance and α-heteroatom effect in its triplet excited state. A plausible reaction mechanism is proposed which was supported by the outcomes of theoretical approaches based on Density Functional Theory (DFT) calculations.

## Abstract 2

Herein, we report the first visible-light-induced strategy for the rapid synthesis of densely functionalized α- and γ-phosphorylated β-sulfonyl enamines in a regio- and stereoselective manner from *N*-sulfonyl allenamides and H-phosphine oxides.

## Graphical abstract

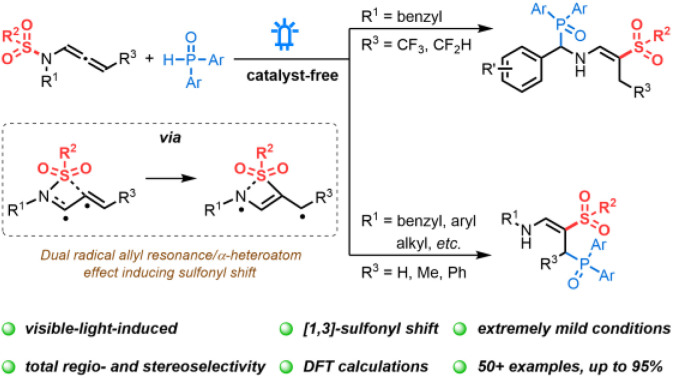

## Introduction

The development of mild, efficient, and sustainable approaches to build complex molecular skeletons has become fundamental in organic synthesis today.^1^ Recent advancements in visible-light catalysis have made tremendous inroads toward these goals with the development of environmentally-friendly techniques.^2^ In this context, the activation of alkene functionalities allows the construction of complex molecular structures through either electron transfer or energy transfer from a species excited by visible-light irradiation.^3^ However, when it comes to allenes, visible-light catalysis remains highly challenging due to the formation of unstable vinyl radical species, whose unreliable reactivity can lead to various byproducts through unwanted reaction paths.^4^

**Scheme 1:**
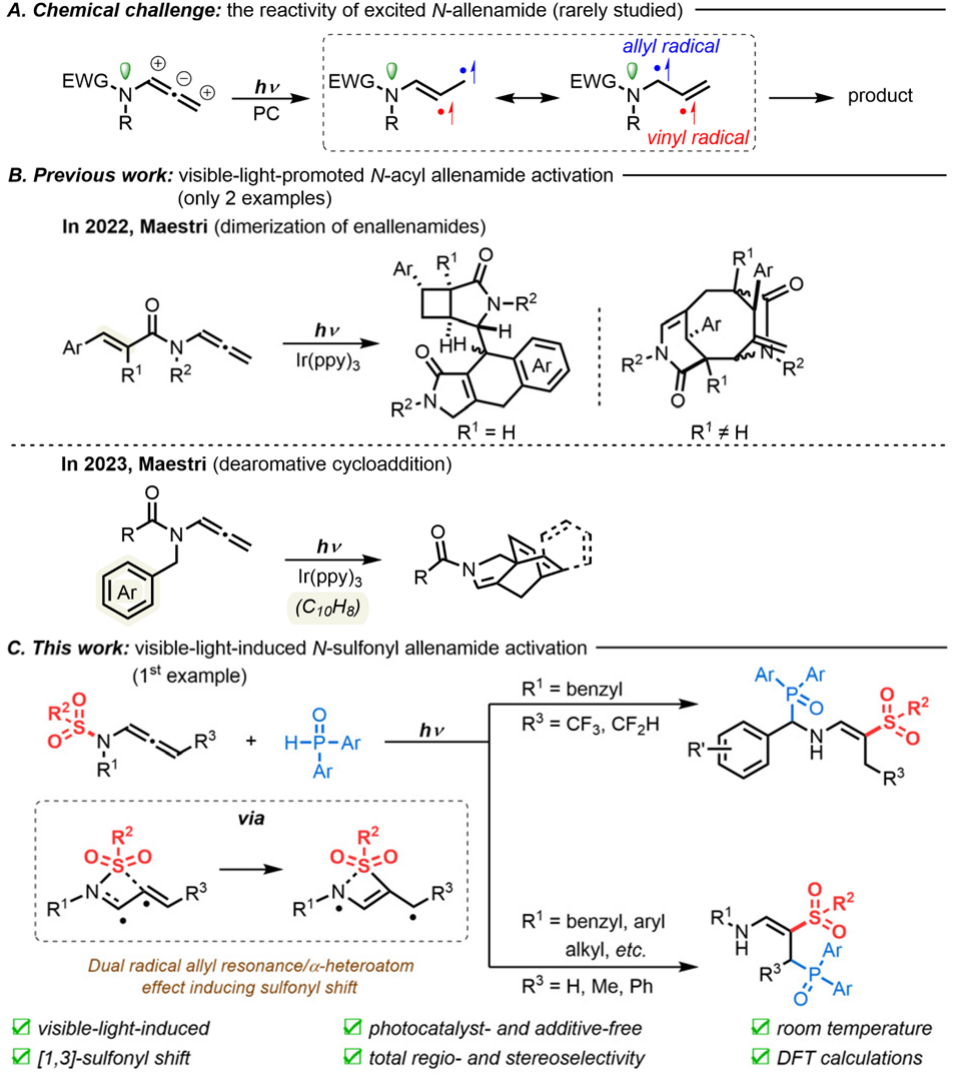
Visible-light-induced activation of allenes in *N*-allenamide derivatives.

As readily available allene sources, *N*-allenamides bearing an electron-withdrawing group (EWG) on the nitrogen atom have emerged as powerful nitrogen-containing synthons in a variety of transformations.^5^ In contrast to their well-established thermal reactivity, which depends on an electronic bias in their ground state (S_0_),^6^ their properties and reactivity in the triplet excited state (T_1_) remain elusive (Scheme 1A). So far, only two examples, which involve a vinyl radical intramolecular addition process, have been depicted, using tailor-made *N*-acyl allenamides in the presence of an Ir(ppy)_3_ photocatalyst (Scheme 1B). In 2022, Maestri's group reported the first visible-light-promoted dimerization of enallenamides to trigger a domino reaction for the synthesis of complex polycyclic systems.^7^ Later, the same group reported the preparation of complex [2.2.2]-(hetero)-bicyclooctadienes by the dearomative cycloaddition of *N*-acyl allenamides.^8^

We were, however, puzzled that the reactivity of excited *N*-sulfonyl allenamides was still unexplored. We believed that the presence of the sulfonyl moiety could be used to our advantage to control the fate of the vinyl radical intermediate and to unlock new reactivities. We thus envisioned the following reaction design: Once a diradical species is formed, under visible-light, from *N*-allenamides, we reasoned that a direct *N*-to-*C* [1,3]-sulfonyl shift process could take place. Then, depending on the substrate pattern, two divergent pathways could be envisioned, yielding either α- or γ-phosphorylated β-sulfonyl enamines following trapping by H-phosphine oxides. Such a strategy would provide densely functionalized compounds that incorporate relevant functional groups for drug discovery,^9^ including vinyl-sulfones^10^ and amino-phosphonates.^11^ In contrast to the numerous examples of N–S cleavage generating sulfonyl radicals through the use of radical initiators^12^ or high-energy photons,^13^ the direct *N*-to-*C* [1,3]-sulfonyl shift of *N*-sulfonyl allenamides under visible-light irradiation would constitute a first, which could pave the way for unprecedented reactivity in the foreseeable future. Here, by using *N*-sulfonyl allenamides as substrates, we describe the first regio- and stereoselective synthesis of α- and γ-phosphorylated enamines in the presence of H-phosphine oxides under visible-light irradiation (Scheme 1C).

## Results and discussion

**Scheme 2:**
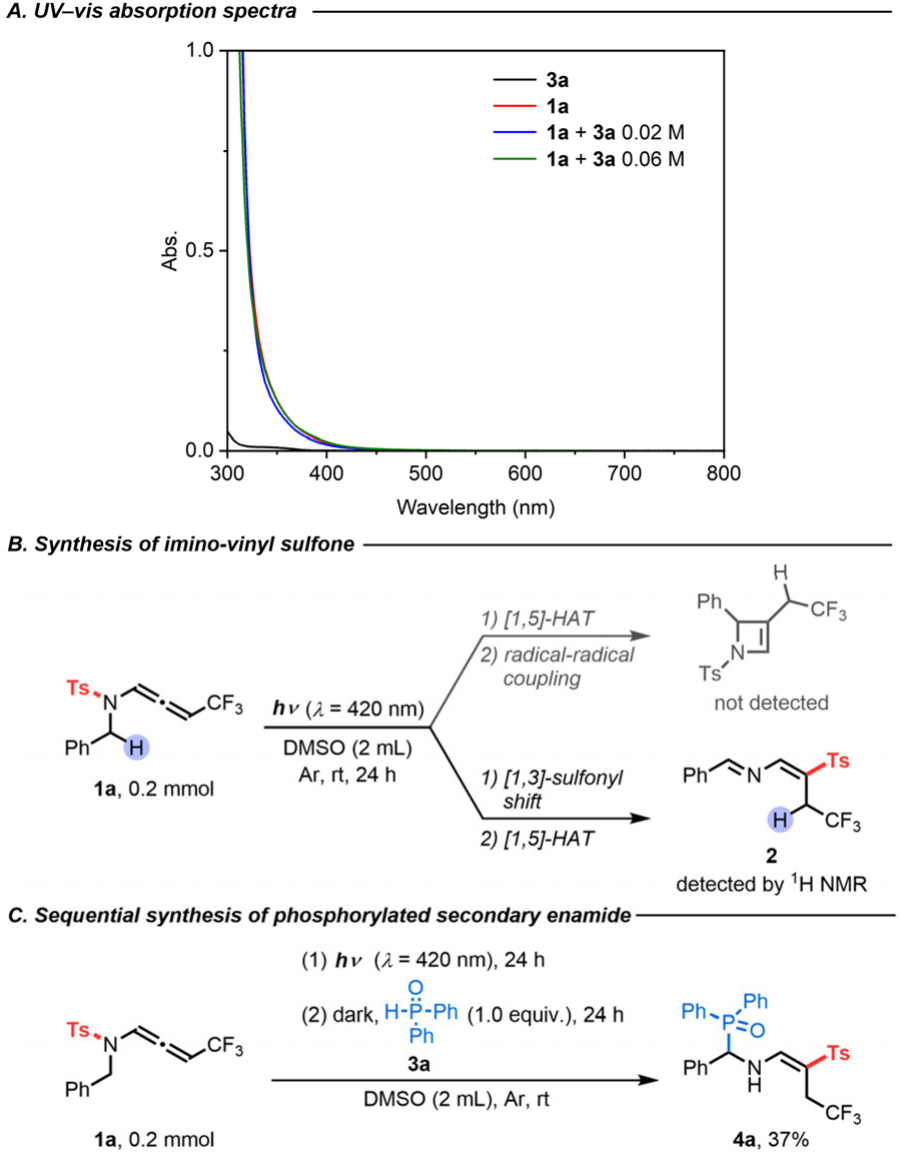
Preliminary experiments: (A) UV-vis absorption spectra of 3a (2.0 × 10^−2^ M) in DMSO (black); 1a (2.0 × 10^−2^ M) in DMSO (red); 1a (2.0 × 10^−2^ M) and 3a (2.0 × 10^−2^ M) in DMSO under an argon atmosphere (blue); 1a (2.0 × 10^−2^ M) and 3a (6.0 × 10^−2^ M) in DMSO under an argon atmosphere (green). (B) Synthesis of imino-vinyl sulfone. (C) Sequential synthesis of phosphorylated secondary enamide.

Initially, we tested our working hypothesis on CF_3_-substituted *N*-allenamide 1a. As shown in its UV-visible absorption spectra, compound 1a exhibited a wide and strong absorption band in the UV-light region that slightly extends into the visible-light region (*ε* = 0.45 L mol^−1^ cm^−1^ in 420 nm, Scheme 2A). At first, we envisioned that the highly reactive triplet state intermediate of compound 1a could potentially undergo a Norrish–Yang-type reaction,^14^ which would involve an intramolecular [1,5]-hydrogen atom transfer (HAT)^15^ followed by radical–radical coupling to form an unsaturated azetidine (Scheme 2B).^16^ To test this hypothesis, we irradiated substrate 1a (0.2 mmol) in degassed DMSO (2 mL) with blue LEDs (*λ* = 420 nm) for 24 h at room temperature. However, the azetidine was not detected, while the formation of a new product – imino-vinyl sulfone 2 – was observed by ^1^H NMR (Scheme 2B). Such a product would result from a stereoselective *N*-to-*C* [1,3]-sulfonyl shift. Interestingly, the N–S bond dissociation energy of compound 1a, which was calculated to be 47.3 kcal mol^−1^,^17^ and the computed spin density distribution of the excited state as illustrated in the resonance forms (Scheme 1C) are consistent with a *N*-to-*C* [1,3]-sulfonyl shift process.

To facilitate the characterization of this compound, diphenylphosphine oxide (3a) was added to the reaction mixture after the photoreaction to engineer a carbo-phosphorylation, affording (*E*)-diphenyl(phenyl((4,4,4-trifluoro-2-tosylbut-1-en-1-yl)amino)methyl)phosphine oxide (4a) in 37% yield (Scheme 2C).^18^ Furthermore, a direct addition of the H-phosphine oxide 3a to the CF_3_-substituted *N*-sulfonyl allenamide 1a under visible-light irradiation ultimately produced the α-phosphorylated β-sulfonyl enamine 4a with a higher yield of 86% (Table 1, entry 1). Of note, no side products were observed in this atom-economical process. During the optimization phase of this study, we screened a series of solvents, all of which showed lower reactivity but were still effective for the protocol (Table 1, entries 2–7). Moreover, the fact that the reaction does not proceed in the absence of visible-light irradiation supports the photoactivated nature of the reaction (Table S1,† entry 8). Anaerobic conditions were required for the transformation to take place (Table S1,† entry 9). Finally, a light on/off reaction was conducted (Fig. S7†), showing the advancement of transformation under blue LED irradiation. The absence of reaction in the dark is consistent with the necessity of a continuous irradiation.

**Table 1:** Variation from standard conditions^a^^b^

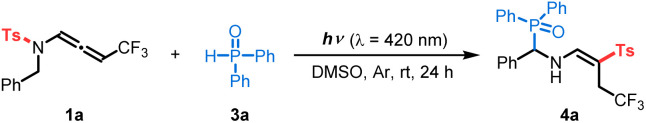		
Entry	Solvent	Yield^b^ (%)
1	None	86
2	MeCN	51
3	THF	28
4	Ethyl acetate	31
5	CH_2_Cl_2_	32
6	Toluene	60
7	Acetone	14

a Standard conditions: 1a (0.2 mmol), 3a (0.2 mmol, 1.0 equiv.) in DMSO (2 mL) was irradiated for 24 h with blue LEDs (*λ* = 420 nm) under an argon atmosphere at room temperature.

b Isolated yields, n.r. = no reaction.

**Scheme 3:**
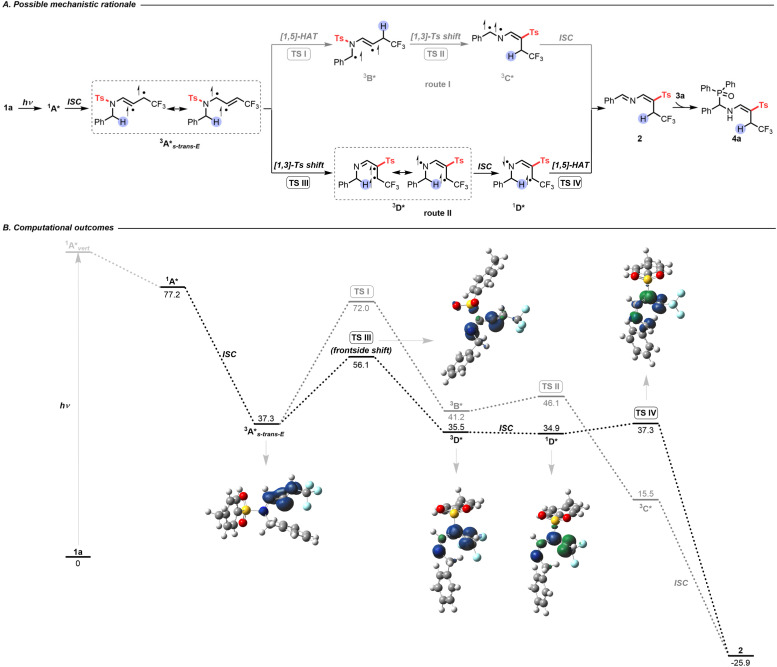
(A) Possible mechanistic rationale. (B) Computational outcomes. Gibbs free energies (kcal mol^−1^) were computed at the PBE0/6-311++G(d,p) level of theory using DMSO as an implicit solvent with the PCM method. Spin densities are reported for the triplet and singlet diradical states (isocontour value 0.01 au).

Based on the above preliminary investigations, Density Functional Theory (DFT) and Time Dependent (TD-DFT) calculations suggested a possible mechanistic rationale as shown in Scheme 3. Under visible-light irradiation, compound 1a is promoted vertically to the intermediate ^1^A*_vert_, which undergoes vibrational relaxation to produce intermediate ^1^A* (Fig. S3†). Subsequently, a triplet excited state diradical intermediate ^3^A* is generated through intersystem crossing (ISC) (Fig. S5†).^19^ To evaluate the most plausible geometry of intermediate ^3^A*, four possible conformations have been hypothesized. The intermediate ^3^A*_s-trans-E_ is found to be the most energetically stable and is therefore the one considered in the following study (Scheme S10†). At this point, two potential routes could exist: [1,5]-HAT followed by a [1,3]-tosyl (Ts) shift (route I, Scheme 3A); or [1,3]-Ts shift and subsequent [1,5]-HAT (route II, Scheme 3A). Comparing the Gibbs free energy of transition states TS I with TS III, a difference of 15.9 kcal mol^−1^ is found, suggesting that route I is thermodynamically unfavorable. The triplet state intermediate ^3^D* is also found to be more energetically stable than intermediate ^3^B*, with a difference in Gibbs energy of 5.7 kcal mol^−1^ (Scheme 3B). Route II involves a direct *N*-to-*C* [1,3]-Ts shift. A frontside or a backside shift of the Ts group on intermediate ^3^A* could take place to form the *E* or *Z* isomer of the diradical triplet intermediate ^3^D* (Scheme S11A†). The reaction mechanism for both isomers has thus been computed and is detailed in the ESI Scheme S11B.† These results show that the *E* isomer of all of the intermediates and transition states is more stable than the corresponding *Z* isomer, contributing to a total stereoselectivity for the *E* isomer. The intermediate ^3^D* could relax *via* ISC to give the singlet state diradical intermediate ^1^D*, and a subsequent *C*-to-*C* [1,5]-HAT proceeds *via* transition state TS IV to produce imino-vinyl sulfone 2. For the latter step, radical–radical coupling for the intermediate ^1^D* was ruled out as a competent mechanism, since it would either lead to the formation of a 4-membered ring with high strain, or, in the case of intramolecular radical–radical coupling, it was expected to be disfavoured with respect to the intramolecular H-transfer reaction that occurs practically spontaneously (with a barrier computed to be roughly 3.0 kcal mol^−1^). The energy profile indicates route II to be the most thermodynamically and kinetically favorable pathway. As no shift in the UV-visible absorption spectra of the reaction mixture in DMSO was detected, the photoactive electron donor–acceptor (EDA) complex between 1a and 3a was negligible (Scheme 2A). Finally, quenching of imino-vinyl sulfone 2 with H-phosphine oxide 3a yields the amino-phosphinoyl alkene 4a, which is further functionalized with a tosyl sulfone.

With an understanding of the reaction mechanism, the viability of the visible-light-induced strategy was explored (Table 2). We examined a series of benzyl groups with different electron-donating groups (EDGs) and EWGs at various positions of the aromatic rings. Substituents at the *para*-position as well as at the *ortho*-position were tolerated, forming the corresponding products (4b–4l) in 68–95% yields. Moreover, substrate 1a was subjected to a scale-up reaction to demonstrate the practicality of the protocol, which provided a satisfactory yield of the target product 4a. X-ray analyses of 4l confirmed the structure of the α-phosphorylated β-sulfonyl enamine (CCDC 2281377 contains the supplementary crystallographic data for the structure, see details in the ESI†).^20^ Disubstituted benzyl-groups (4m–4o) were also compatible with similar efficiency. Additionally, diversification of the process was demonstrated by carrying out the reaction in methanol (2 mL), leading to regio- and stereoselective formation of the corresponding alkoxy-amine 4p in 95% yield. The naphthyl derivative led to the desired product 4q in moderate yield. More interestingly, difluorinated *N*-allenamide 1r was used in this process, providing an interesting drug-like enamine 4r.^21^ Decomposition of non-benzylic *N*-allenamide (1s) was observed under the reaction conditions because the formation of an imine moiety is excluded in this case (Scheme S4†).

**Table 2:** Substrate scope of α-phosphorylated β-sulfonyl enamines^a^^b^^c^^d^

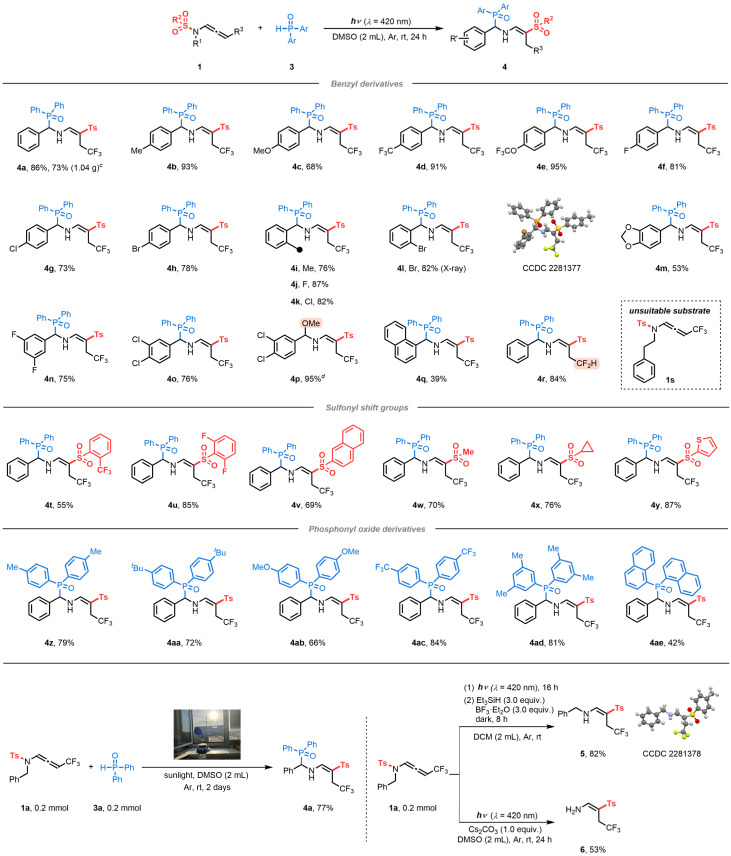

a Reaction condition A: 1 (0.2 mmol), 3 (0.2 mmol, 1.0 equiv.) in DMSO (2 mL) was irradiated for 24 h with blue LEDs (*λ* = 420 nm) under an argon atmosphere at room temperature.

b Isolated yields.

c Scale-up reaction: 1a (2.5 mmol), 3a (2.5 mmol, 1.0 equiv.) in DMSO (15 mL) was irradiated for 24 h with blue LEDs (*λ* = 420 nm) under an argon atmosphere at room temperature.

d Reaction condition B: 1a (0.2 mmol) in MeOH (2 mL) was irradiated for 24 h with blue LEDs (*λ* = 420 nm) under an argon atmosphere at room temperature.

We next focused on the scope of this transformation, using substrates encompassing different sulfonyl amides, including fluorinated sulfonyl groups (1t and 1u), as well as naphthyl (1v), alkyl (1w), and cycloalkyl (1x) derivatives, all of which reacted successfully to provide the corresponding α-phosphorylated β-sulfonyl enamines in high yields. Even thienyl derivatives proved suitable substrates, affording the corresponding product 4y in 87% yield. It was also possible to modify the H-phosphine oxides in this transformation. An array of H-phosphine oxides substituted with different EDGs and EWGs were tested, demonstrating their ability to provide the corresponding phosphorylated enamines (4z–4ad). The naphthyl derivative is also a viable compound, producing carbo-phosphorylated compound 4ae in 42% yield.

To highlight the sustainability of this synthetic process, we performed this reaction under sunlight irradiation for two days.^22^ In this way, the target phosphorylated enamine was obtained in 77% yield. Furthermore, imino-vinyl sulfone 2 was efficiently reduced by Et_3_SiH and BF_3_·Et_2_O, producing secondary enamine 5 in 82% yield.^23^ X-ray analyses of 5 confirmed its structure, and facilitated the assignment of the stereochemistry of the double bond (CCDC 2281378 contains the supplementary crystallographic data for the structure, see details in the ESI†).^24^ In addition, hydrolysis of the imine intermediate under basic conditions provided primary enamine 6 in 53% yield.^25^

**Scheme 4:**
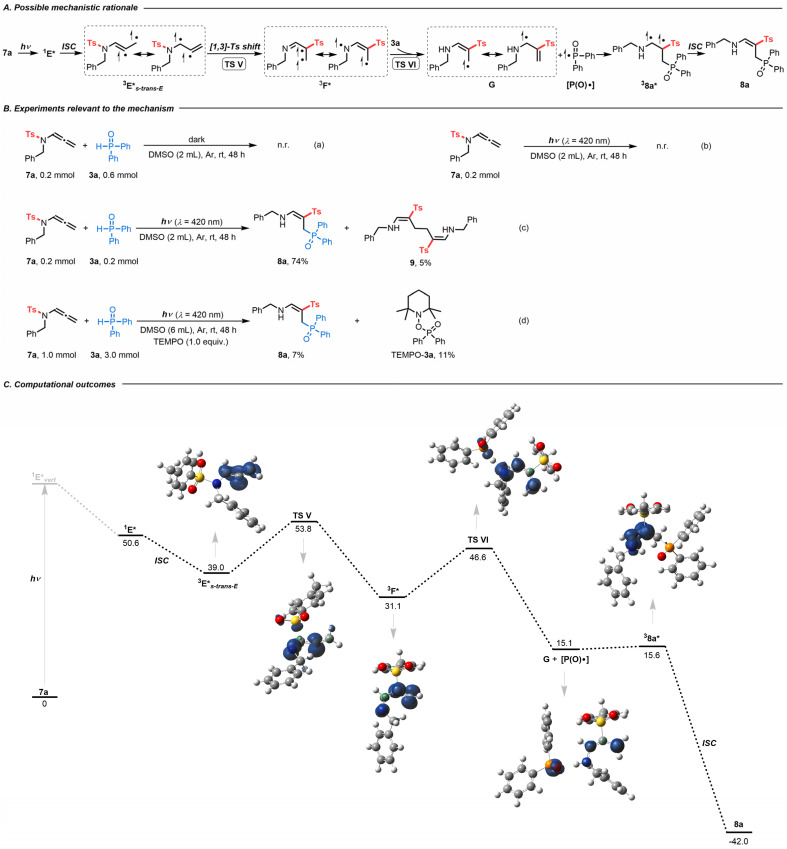
(A) Possible mechanistic rationale. (B) Experiments relevant to the mechanism. (C) Computational outcomes. Gibbs free energies (kcal mol^−1^) computed at the PBE0/6-311++G(d,p) level of theory using DMSO as an implicit solvent with the PCM method. Spin densities are reported for the triplet and singlet diradical states (isocontour value 0.01 au).

After developing an interest in the visible-light-induced *N*-to-*C* [1,3]-sulfonyl shift, we wondered whether this strategy could be expanded beyond CF_3_-substituted *N*-allenamides. Upon employing terminal *N*-allenamides in the presence of H-phosphine oxides, we observed a different outcome, resulting in a completely different hydrophosphorylated product. It is noteworthy that the nature of the phosphorylated product changes based simply on the substitution pattern of the substrates. In this case, we hypothesized that an intermolecular HAT takes place followed by addition of a phosphinoyl radical [P(O)·] to the terminal part of the alkene, affording γ-phosphorylated β-sulfonyl enamines 8 (Scheme 4A). As no spectral shift of the reaction mixture in DMSO could be detected in the UV-visible absorption spectra, formation of a photoactive EDA complex between 7a and 3a seems unlikely (Fig. S2†). In the absence of light irradiation, no reaction took place, which proved its photocatalytic nature [Scheme 4B(a)]. Control experiments suggested the necessity of a hydrogen atom donor. When we carried out the reaction without H-phosphine oxide 3a, starting material 7a was completely recovered, which might be attributed to the diminished reactivity of the allylic radical [Scheme 4B(b)]. With the addition of starting materials 7a (0.2 mmol, 1.0 equiv.) and 3a (1.0 equiv.) in degassed DMSO (2 mL) and irradiation for 48 h at room temperature, the homodimerization compound 9 was obtained in 5% yield, providing evidence for the formation of transient terminal allyl radical G [Scheme 4B(c)].^26^ The radical inhibitor 2,2,6,6-tetramethylpiperidin-1-oxyl (TEMPO) greatly suppressed the reaction, with 8a formed in only about 7% yield. Importantly, compound TEMPO-3a was detected by ^1^H NMR, supporting the generation of phosphinoyl radicals [P(O)·] (Scheme 4B(d)).

DFT calculations provided more details on the regioselective phosphorylation (Scheme 4C). With visible-light irradiation, 7a is promoted vertically to intermediate ^1^E*_vert_, which undergoes vibrational relaxation to produce intermediate ^1^E* (Fig. S4†). Through ISC, the triplet diradical intermediate ^3^E* can be formed (Fig. S6†). At this point a stereoselective frontside *N*-to-*C* [1,3]-Ts shift (TS V) occurs to form intermediate ^3^F*. In this case H-phosphine oxide demonstrated its utility as a hydrogen atom donor, facilitating the formation of intermediate G and phosphinoyl radical [P(O)·] *via* transition state TS VI.^27^ Radical allylic resonance enables the addition of the phosphinoyl radical [P(O)·] to the terminal alkene, affording triplet diradical intermediate ^3^8a*. Finally, ISC led to the formation of γ-phosphorylated β-sulfonyl enamine 8a.

We then explored the generality of this reaction. Under reaction condition C (Table 3), different benzyl-substituted terminal *N*-sulfonyl allenamides showed high reactivity to form the corresponding products (8a–8e) with complete control of regio- and stereoselectivity. X-Ray analyses of 8a confirmed the structure of the γ-phosphorylated β-sulfonyl enamine (CCDC 2310786 contains the supplementary crystallographic data for the structure, see details in the ESI†).^28^ Scale-up reactions led to 8a with a slight erosion of the yield (81 *vs.* 65%). Importantly, this protocol was also compatible with thienyl-substituted substrates producing 8f in 62% yield. Phenyl moieties on the nitrogen atom were utilized to generate desired products (8g–8j) in 70–80% yields. Linear alkyls (7k) and cyclopropyls (7l) proved compatible, allowing the formation of the corresponding phosphorylated enamines in 47 and 73%, respectively. The delocalization of spin densities may explain the preservation of the *N*-cyclopropyl arm of product 8l, with the rate of ring-opening of an α-cyclopropyl radical typically being inversely proportional to the degree of spin delocalization of the nucleus.^29^ Additionally, functionalized side chains (7m, 7n and 7o) underwent a [1,3]-Ts shift, expanding the chemical space of those structures. Terminal *N*-sulfonyl allenamides with different aryl (7p) and heteroaryl (7q) sulfonyls on the nitrogen atom, were also effective for this *N*-to-*C* [1,3]-sulfonyl shift reaction. In addition, H-phosphine oxides substituted with 4-^*t*^Bu– and 3,5-Me– were converted to the corresponding products 8r and 8s in 88 and 84% yields, respectively.

**Table 3:** Substrate scope of γ-phosphorylated β-sulfonyl enamines^,^^a^^b^^c^^d^

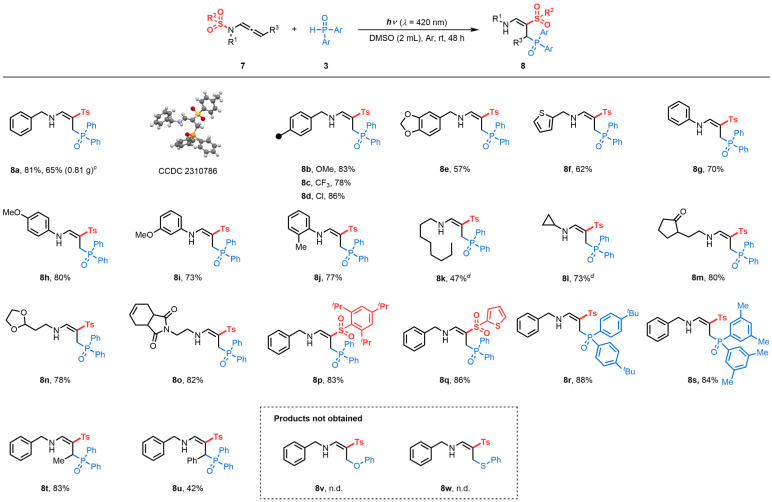

a Reaction condition C: 7 (0.2 mmol), 3 (0.6 mmol, 3.0 equiv.) in DMSO (2 mL) was irradiated for 48 h with blue LEDs (*λ* = 420 nm) under an argon atmosphere at room temperature.

b Isolated yields.

c Scale-up reaction: 7a (2.5 mmol), 3a (2.5 mmol, 1.0 equiv.) in DMSO (15.0 mL) was irradiated for 48 h with blue LEDs (*λ* = 420 nm) under an argon atmosphere at room temperature.

d 72 h.

Conceivably, the outcome of the transformation is closely related to the stability of the allylic radical involved. Similar to terminal *N*-allenamides, the Me–*N*-allenamide 7t led to the formation of γ-phosphorylated β-sulfonyl enamine 8t. Benzylic allylic radicals generated through photoactivation of Ph-*N*-allenamide 7u followed the same trend, yielding the corresponding γ-phosphorylated β-sulfonyl enamine 8u. In contrast, the presence of –CF_3_ or –CF_2_H stabilizes the allylic radical, hence favoring the intramolecular [1,5]-HAT process providing α-phosphorylated β-sulfonyl enamines. Some limitations of the method were also observed, for example, phenol and thiophenol did not lead to formation of the corresponding products 8v and 8w.

## Conclusions

We report herein the first visible-light-induced strategy for the synthesis of α- and γ-phosphorylated β-sulfonyl enamines from *N*-sulfonyl allenamides in the presence of H-phosphine oxides under catalyst- and additive-free conditions. This simple, efficient, and atom-economical process exhibits a broad substrate scope, excellent functional compatibility and complete regio- and stereo-selectivity. In addition, a scale-up reaction and sunlight irradiation make the developed method sustainable and amenable for potentially operational procedures. We anticipate that the unique mechanistic features discovered here, involving the high reactivity of excited *N*-allenamides, will have broad implications beyond this work.

## Author contributions

L. M. and J.-D. G. conceived and designed the experiments. L. M. directed the project. J.-D. G and D. S. performed the experiments. F.-A. K. performed the DFT calculations. I. C. supervised the theoretical work. L. M., J.-D. G., I. C. and F.-A. K. wrote the paper. L. M., J.-D. G., I. C., F.-A. K. and D. S. discussed the results and commented on the manuscript.

## Conflicts of interest

The authors declare no conflict of interest.

## Supplementary Material

SC-015-D4SC05190D-s001

SC-015-D4SC05190D-s002

## Data Availability

All data presented in this manuscript are available in the ESI.†
