# Supplementary material for: Photocatalyst-free, visible-light-induced regio- and stereoselective synthesis of phosphorylated enamines from N-allenamides via [1,3]-sulfonyl shift at room temperature
Source: Chem Sci. 2024 Oct 3;15(43):17962–70. doi: 10.1039/d4sc05190d (PMC11467721; doi:10.1039/d4sc05190d)
Supplement: SC-015-D4SC05190D-s001 [file SC-015-D4SC05190D-s001.pdf]

Supplementary Information (SI) for Chemical Science.
This journal is © The Royal Society of Chemistry 2024

Supporting Information

Photocatalyst-free, visible-light-induced regio- and stereoselective synthesis of phosphorylated enamines from *N*-allenamides *via* [1,3]-sulfonyl shift at room temperature

Jia-Dong Guo,[a] Feven-Alemu Korsaye,[b] Dorian Schutz,[a] Ilaria Ciofini[b]* and Laurence Miesch[a]*

a. Equipe Synthèse Organique et Phytochimie, Institut de Chimie, CNRS-UdS, UMR 7177, 4 rue Blaise Pascal, CS 90032, 67081, Strasbourg.

b. Chimie ParisTech, PSL University, CNRS, Institute of Chemistry for Life and Health Sciences, Chemical Theory and Modelling Group, F-75005 Paris.

Corresponding authors: lmiesch@unistra.fr

ilaria.ciofini@chimieparistech.psl.eu

Table of Contents

1. General information........S3
2. Substrates preparation........S4
 2.1 Synthesis of compounds **1a–1o**, **1q** and **1s–1y**........S4
 2.2 Synthesis of compound **1r**........S4
 2.3 Synthesis of compouds **3b–3g**........S5
 2.4 Synthesis of compouds **7a–7q**........S5
 2.5 Synthesis of compouds **7t**........S6
 2.6 Synthesis of compouds **7u**........S6
3. Reaction optimization........S8
4. General experimental procedure........S9
5. Optical spectroscopic data........S9
 5.1 UV-vis absorption spectra........S9
 5.2 Fluorescence spectra........S10
 5.3 Phosphorescence spectra........S10
6. Initial results........S11
 6.1 Synthesis of compound **2**........S11
 6.2 Sequential synthesis of compound **4a**........S11
7. Scale-up experiment........S12
8. Photoreaction with using **1s** as substrate........S12
9. Sunlight reaction........S12
10. Diverse transformations........S13
 10.1 Synthesis of compound **5**........S13
 10.2 Synthesis of compound **6**........S13
11. Experiments relevant to the mechanism: terminal *N*-sulfonyl allenamide........S14
12. Limitations of the method: terminal *N*-sulfonyl allenamide........S15
13. Light on/off experiment........S16
14. Characterization data for compounds........S17
15. Copies of ^{1}H NMR, ^{13}C NMR, ^{19}F NMR and ^{31}P NMR........S43
16. X-ray crystallography data........S192
 16.1 Crystallography data of **4l** (CCDC 2281377)........S193
 16.2 Crystallography data of **5** (CCDC 2281378)........S206
 16.3 Crystallography data of **8a** (CCDC 2310786)........S217
17. Computational details........S230
18. References........S275

1. General information

All required reagents were purchased from commercial suppliers (TCI, Alfa Aesar, Acros and Aladdin) and were used directly without purification. NMR Spectra (^{1}H, ^{13}C, ^{19}F, ^{31}P) were performed at 298 K. ^{1}H (500 MHz, 400 MHz or 300 MHz) and ^{13}C (126 MHz) NMR chemical shifts are reported relative to residual solvent. DEPT (Distortionless Enhancement by Polarization Transfer) shows positive peaks for primary carbons (CH_3) and tertiary carbons (CH), and negative peaks for secondary carbons (CH_2). However, quaternary carbons (C) do not generate a peak. ^{19}F (282 MHz or 471 MHz) and ^{31}P (121 MHz, 162 MHz or 202 MHz) NMR chemical shifts are reported without any calibration. Data are presented as follows: chemical shift (ppm), multiplicity (s = singlet, d = doublet, t = triplet, q = quartet, m = multiplet), coupling constant *J* (Hz) and integration. HRMS data were recorded on a microTOF spectrometer equipped with an orthogonal electrospray (ESI) interface. Thin layer chromatography was performed using Merck TLC silica gel 60 F254 aluminum sheets using petroleum ether/ethyl acetate as eluant and visualized using permanganate stain, ninhydrin stain, vanillin stain and/or UV light. Merck Geduran® 40–63 µm silica gel was used for column chromatography. Biotage® Isolera™ One system was used for flash chromatography. The wavelength of the UV-detector was calibrated at 254 and 365 nm. Infrared spectra were reported in frequency of absorption using Alpha Bruker Optics spectrometer. Melting points were recorded with a SMP3 Stuart Scientific microscope in open capillary tubes and are uncorrected. UV-vis-NIR absorption spectra were recorded with a Cary 5000 UV-vis-NIR spectrophotometer. Photoluminescence spectra (PL) were obtained using a fluorescence spectrophotometer at 298 K or a phosphorescence spectrophotometer at 77 K (Edinburgh Instrument FLS-920). A four-channel LEDs were employed as the irradiation apparatus (4×4 array, for each channel electrical input 3.2 V × 600 mA × 4 blue LEDs, 420 ± 10 nm, light intensity 200~600 mW/cm^{2}), and Pyrex tubes were used as the reaction vessel. The distance from the LEDs to Pyrex tubes was less than 5 mm. No filter was used during irradiation.

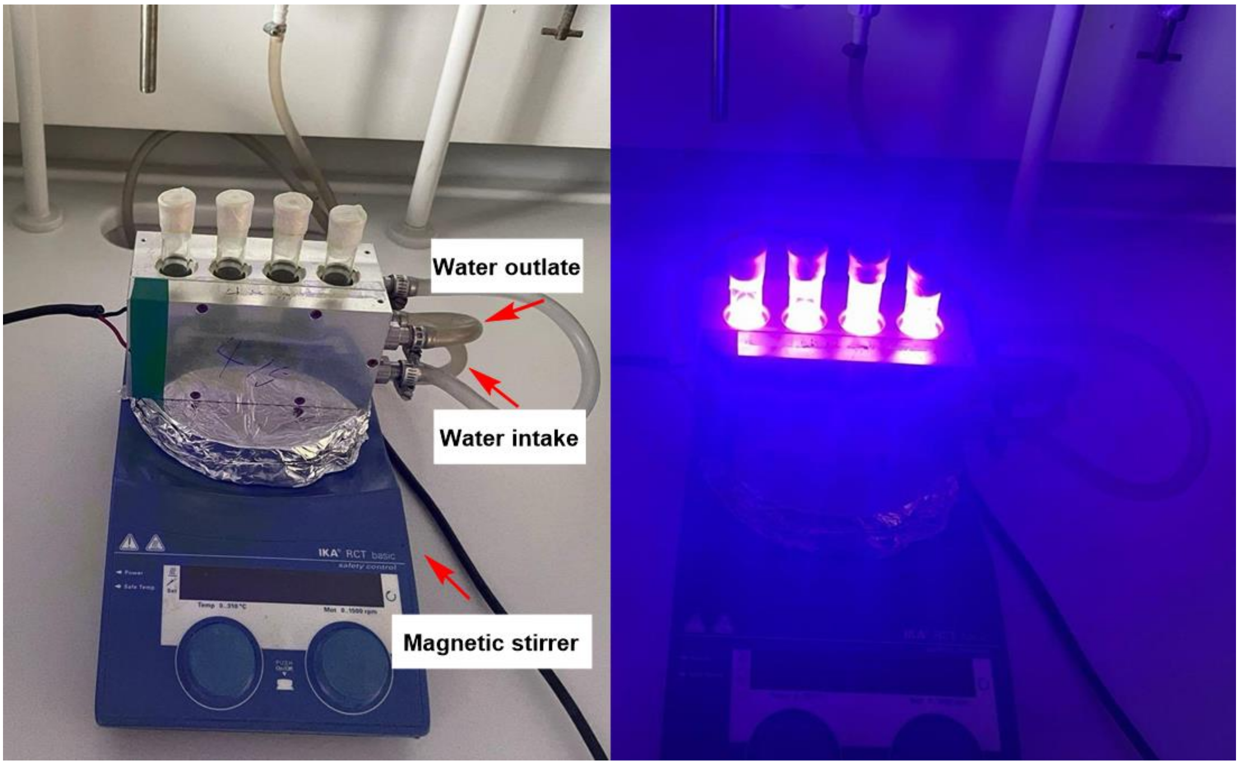

2. Substrates preparation

2.1 Synthesis of compounds 1a–1o, 1q and 1s–1y

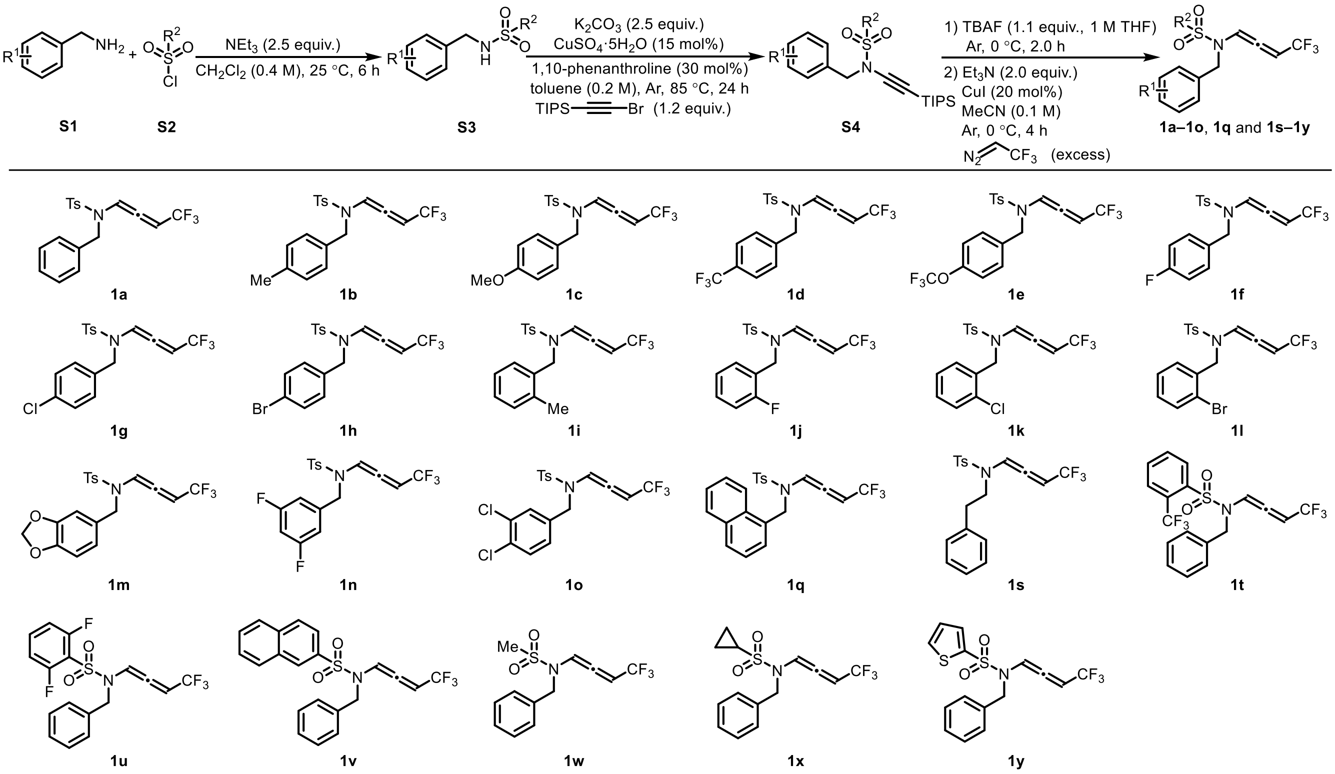

To a solution of amine **S1** (1.0 equiv.) and sulfonyl chloride **S2** (1.2 equiv.) in CH_2Cl_2 (c = 0.4 M) was added dropwise Et_3N (2.5 equiv.). The mixture was stirred at 25 °C during 6 h and then hydrolyzed with an aqueous solution of HCl (1.0 N). The aqueous layer was extracted with CH_2Cl_2 (3 × 30 mL). The organic layers were washed with a saturated solution of NaCl, dried with anhydrous Na_2SO_4, filtered and concentrated under vacuum. The crude sulfonamide **S3** was used without further purification.

In a Schlenk tube was introduced $CuSO_4·5H_2O$ (15 mol%), 1,10-phenanthroline (30 mol%), K_2CO_3 (2.5 equiv.), sulfonamide (1.0 equiv.), anhydrous toluene (c = 0.2 M) and the bromoacetylenic derivative (1.2 equiv.), and bubbled with a stream of argon for 0.5 h. The reaction mixture was heated to 85 °C for 24 h and then cooled down to room temperature, filtered through a pad of Celite® and washed with ethyl acetate. The filtrate was then concentrated under vacuum. The crude material was purified by column chromatography to afford TIPS protected ynamide **S4**.[1]

TIPS protected ynamide (1.0 equiv.) was dissolved in anhydrous THF (c = 0.1 M), and bubbled with a stream of argon for 0.5 h, and cooled to 0 °C. A solution of TBAF (1.1 equiv., 1 M in THF) was added dropwise. The mixture was stirred at 0°C during 2 h, and then hydrolyzed with water. The aqueous layer was extracted with Et_2O (3 × 30 mL). The organic layers were washed with a saturated solution of NaCl, dried with anhydrous Na_2SO_4, filtered and concentrated under vacuum. The crude ynamide was used without further purification.[2] Ynamide (1.0 equiv.) was dissolved in MeCN (c = 0.1 M) in the presence of CuI (20 mol%), and Et_3N (2.0 equiv.), and bubbled with a stream of argon for 0.5 h. 2-diazo-1,1,1-trifluoroethane[3] was added dropwise (in excess) and the reaction was stirred for 4 h at 0 °C. The mixture was concentrated under vacuum and the crude material was purified by flash chromatography treated with Et_3N using a step gradient of ethyl acetate in petroleum ether (ethyl acetate/petroleum ether 0–20%) to afford compounds **1a**–**1o**, **1q** and **1s**–**1y**.[4]

2.2 Synthesis of compound 1r

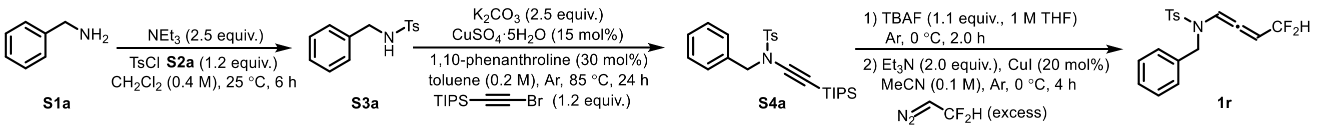

To a solution of phenylmethanamine **S1a** (1.0 equiv.) and 4-methylbenzenesulfonyl chloride **S2a** (1.2 equiv.) in CH_2Cl_2 (c = 0.4 M) was added dropwise Et_3N (2.5 equiv.). The mixture was stirred at 25 °C during 6 h and then hydrolyzed with an aqueous solution of HCl (1.0 N). The aqueous layer was extracted with CH_2Cl_2 (3 × 30 mL). The organic layers were washed with a saturated solution of

NaCl, dried with anhydrous Na_2SO_4, filtered and concentrated under vacuum. The crude *N*-benzyl-4-methylbenzenesulfonamide **S3a** was used without further purification.

In a Schlenk tube was introduced $CuSO_4·5H_2O$ (15 mol%), 1,10-phenanthroline (30 mol%), K_2CO_3 (2.5 equiv.), *N*-benzyl-4-methylbenzenesulfonamide (1.0 equiv.), anhydrous toluene (c = 0.2 M) and the bromoacetylenic derivative (1.2 equiv.), and bubbled with a stream of argon for 0.5 h. The reaction mixture was heated to 85 °C for 24 h and then cooled down to room temperature, filtered through a pad of Celite® and washed with ethyl acetate. The filtrate was then concentrated under vacuum. The crude material was purified by column chromatography to afford compound **S4a**.[1]

S4a (1.0 equiv.) was dissolved in anhydrous THF (c = 0.1 M), and bubbled with a stream of argon for 0.5 h, and cooled to 0 °C. A solution of TBAF (1.1 equiv., 1 M in THF) was added dropwise. The mixture was stirred at 0°C during 2 h, and then hydrolyzed with water. The aqueous layer was extracted with Et_2O (3 × 30 mL). The organic layers were washed with a saturated solution of NaCl, dried with anhydrous Na_2SO_4, filtered and concentrated under vacuum. The crude ynamide was used without further purification.[2] Ynamide (1.0 equiv.) was dissolved in MeCN (c = 0.1 M) in the presence of CuI (20 mol%), and Et_3N (2.0 equiv.), and bubbled with a stream of argon for 0.5 h. 2-diazo-1,1-difluoroethane[3] was added dropwise (in excess) and the reaction was stirred for 4 h at 0 °C. The mixture was concentrated under vacuum and the crude material was purified by flash chromatography treated with Et_3N using a step gradient of ethyl acetate in petroleum ether (ethyl acetate/petroleum ether 0–20%) to afford compound **1r**.[4]

2.3 Synthesis of compouds 3b–3g

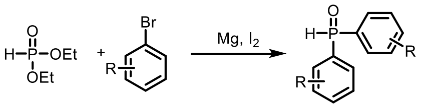

S5 **S6** **3b–3g**

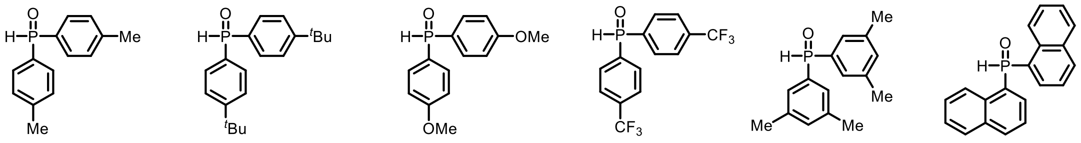

3b **3c** **3d** **3e** **3f** **3g**

Magnesium chips (30 mmol, 3.0 equiv.), catalytic amount of iodine (10 mol%) and 100 mL anhydrous THF solution were added into a 250 mL dry two-necked flask under argon atmosphere. Afterwards, aryl bromide compound **S6** (5 mmol, 0.5 equiv.) was added dropwise. After the initiation, aryl bromide compound **S6** (25 mmol, 2.5 equiv.) was further added dropwise. The reaction mixture was refluxed for 30 minutes before cooled to 0 °C. The solution of diethyl phosphite **S5** (10 mmol, 1.0 equiv.) in 20 mL THF was added dropwise to the reaction system at 0 °C, and the reaction mixture was kept stirring overnight. The reaction was then quenched with saturated NH_4Cl, after which the resulting solution was filtered through Celite, washed with diethyl ether (3 × 10 mL). The filtrate was extracted with diethyl ether (3 × 30 mL), and the organic phase was collected, dried with anhydrous Na_2SO_4, recrystallized with CH_2Cl_2 and petroleum ether to obtain the target pure product **3b**–**3g**.[5]

2.4 Synthesis of compounds 7a–7q

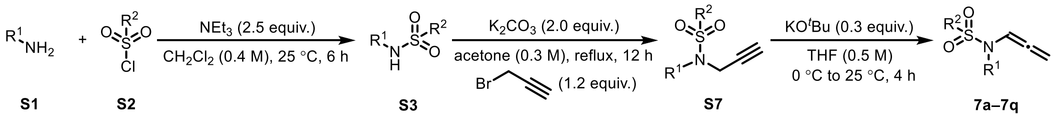

S1 **S2** **S3** **S7** **7a–7q**

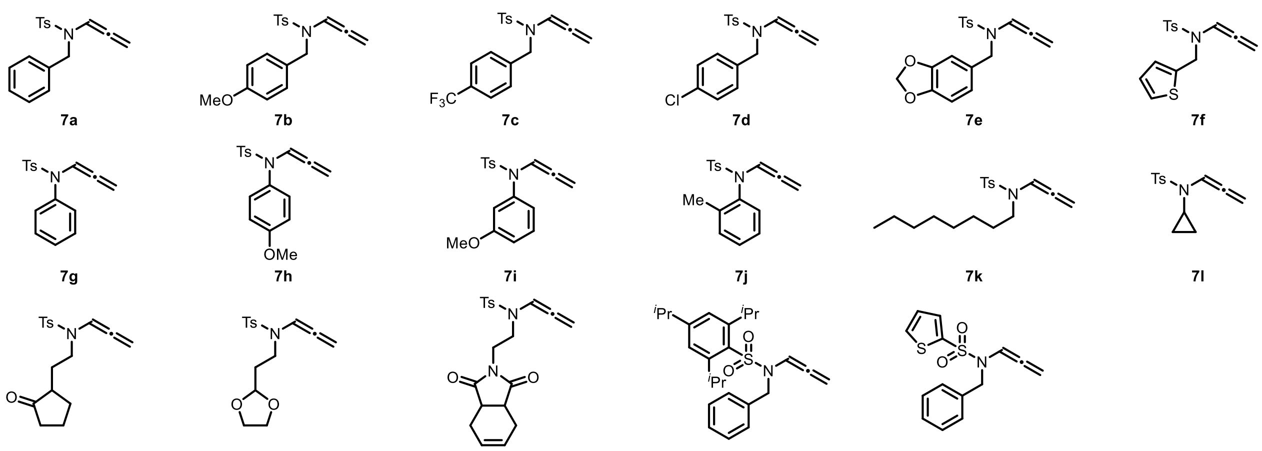

7a **7b** **7c** **7d** **7e** **7f**

7g **7h** **7i** **7j** **7k** **7l**

7m **7n** **7o** **7p** **7q**

To a solution of amine **S1** (1.0 equiv.) and sulfonyl chloride **S2** (1.2 equiv.) in CH_2Cl_2 (c = 0.4 M) was added dropwise Et_3N (2.5 equiv.). The mixture was stirred at 25 °C during 6 h and then hydrolyzed with an aqueous solution of HCl (1.0 N). The aqueous layer was extracted with CH_2Cl_2 (3 × 30 mL). The organic layers were washed with a saturated solution of NaCl, dried with anhydrous Na_2SO_4, filtered and concentrated under vacuum. The crude sulfonamide **S3** was used without further purification.

A flame-dried two-neck 100 mL round-bottomed flask equipped with a magnetic stir bar was charged with sulfonamide **S3** (1.0 equiv.), K_2CO_3 (2.0 equiv.) and acetone (c = 0.3 M), capped with a rubber septum and flushed with argon followed by dropwise addition of 3-bromopropyne (1.2 equiv.). The reaction mixture was refluxed with stirring for 12 h. Then the resulting mixture was quenched by addition of water and extracted with ethyl acetate (3 × 50 mL). The combined organic fractions were washed with brine (50 mL), dried over Na_2SO_4 and filtrated. After the solvent was removed under reduced pressure, the residue was purified by silica gel column chromatography to afford the pure alkynes **S7**.

A flame-dried 100 mL round-bottomed flask equipped with a magnetic stir bar was charged with alkyne (1.0 equiv.), capped with a rubber septum and flushed with argon, was added anhydrous THF (c = 0.5 M) and cooled to 0 ºC. A solution of KO^{t}Bu (0.3 equiv.) in anhydrous THF was added dropwise. The reaction mixture was warmed to room temperature and stirred for 4 h. The resulting mixture was quenched by addition of water and extracted with ethyl acetate (3 × 50 mL). The combined organic fractions were washed with brine (50 mL), dried over Na_2SO_4 and filtrated. After the solvent was removed under reduced pressure, the residue was purified by silica gel column chromatography to afford the allenes **7a**–**7q**.

2.5 Synthesis of compouds 7t

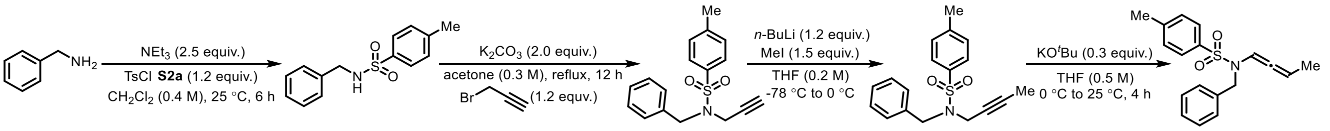

S1a **S3a** **S7a** **S8** **7t**

To a solution of phenylmethanamine **S1a** (1.0 equiv.) and 4-methylbenzenesulfonyl chloride **S2a** (1.2 equiv.) in CH_2Cl_2 (c = 0.4 M) was added dropwise Et_3N (2.5 equiv.). The mixture was stirred at 25 °C during 6 h and then hydrolyzed with an aqueous solution of HCl (1.0 N). The aqueous layer was extracted with CH_2Cl_2 (3 × 30 mL). The organic layers were washed with a saturated solution of NaCl, dried with anhydrous Na_2SO_4, filtered and concentrated under vacuum. The crude *N*-benzyl-4-methylbenzenesulfonamide **S3a** was used without further purification.

A flame-dried two-neck 100 mL round-bottomed flask equipped with a magnetic stir bar was charged with sulfonamide **S3a** (1.0 equiv.), K_2CO_3 (2.0 equiv.) and acetone (c = 0.3 M), capped with a rubber septum and flushed with argon followed by dropwise addition of 3-bromopropyne (1.2 equiv.). The reaction mixture was refluxed with stirring for 12 h. Then the resulting mixture was quenched by addition of water and extracted with ethyl acetate (3 × 50 mL). The combined organic fractions were washed with brine (50 mL), dried over Na_2SO_4 and filtrated. After the solvent was removed under reduced pressure, the residue was purified by silica gel column chromatography to afford the pure alkynes **S7a**.

Under an argon atmosphere, the alkynes **S7a** (1.0 equiv.) was solubilized in THF (c = 0.2 M) and subsequently added dropwise to a solution of *n*-BuLi (1.2 equiv.) at -78°C. Subsequently, the mixture was subjected to stirring for 30 minutes at this cryogenic temperature. Then, MeI (1.5 equiv.) was added dropwise to the reaction system, and the reaction mixture was warmed to 0°C, and stirred for an additional 12 h. The reaction was quenched with water at 0 °C. The organic phase was extracted with ethyl acetate (3 × 30 mL), and the combined organic extracts were dried over Na_2SO_4, filtered, and concentrated under reduced pressure to afford a crude residue. The crude product was purified by silica gel column chromatography to afford the **S8**.

A flame-dried 100 mL round-bottomed flask equipped with a magnetic stir bar was charged with the compound **S8** (1.0 equiv.), capped with a rubber septum and flushed with argon, was added anhydrous THF (c = 0.5 M) and cooled to 0 ºC. A solution of KO^{t}Bu (0.3 equiv.) in anhydrous THF was added dropwise. The reaction mixture was warmed to room temperature and stirred for 4 h. The resulting mixture was quenched by addition of water and extracted with ethyl acetate (3 × 50 mL). The combined organic fractions were washed with brine (50 mL), dried over Na_2SO_4 and filtrated. After the solvent was removed under reduced pressure, the residue was purified by silica gel column chromatography to afford compound **7t**.

2.6 Synthesis of compouds 7u

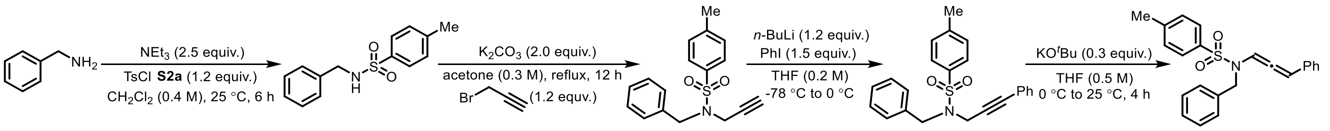

S1a **S3a** **S7a** **S9** **7u**

To a solution of phenylmethanamine **S1a** (1.0 equiv.) and 4-methylbenzenesulfonyl chloride **S2a** (1.2 equiv.) in CH_2Cl_2 (c = 0.4 M) was added dropwise Et_3N (2.5 equiv.). The mixture was stirred at 25 °C during 6 h and then hydrolyzed with an aqueous solution of HCl (1.0 N). The aqueous layer was extracted with CH_2Cl_2 (3 × 30 mL). The organic layers were washed with a saturated solution of NaCl, dried with anhydrous Na_2SO_4, filtered and concentrated under vacuum. The crude *N*-benzyl-4-methylbenzenesulfonamide **S3a** was used without further purification.

A flame-dried two-neck 100 mL round-bottomed flask equipped with a magnetic stir bar was charged with sulfonamide **S3a** (1.0 equiv.), K_2CO_3 (2.0 equiv.) and acetone (c = 0.3 M), capped with a rubber septum and flushed with argon followed by dropwise addition of 3-bromopropyne (1.2 equiv.). The reaction mixture was refluxed with stirring for 12 h. Then the resulting mixture was quenched by addition of water and extracted with ethyl acetate (3 × 50 mL). The combined organic fractions were washed with brine (50 mL), dried over Na_2SO_4 and filtrated. After the solvent was removed under reduced pressure, the residue was purified by silica gel column chromatography to afford the pure alkynes **S7a**.

Under an argon atmosphere, the alkynes **S7a** (1.0 equiv.) was solubilized in THF (c = 0.2 M) and subsequently added dropwise to a solution of *n*-BuLi (1.2 equiv.) at -78°C. Subsequently, the mixture was subjected to stirring for 30 minutes at this cryogenic temperature. Then, PhI (1.5 equiv.) was added dropwise to the reaction system, and the reaction mixture was warmed to 0°C, and stirred for an additional 12 h. The reaction was quenched with water at 0 °C. The organic phase was extracted with ethyl acetate (3 × 30 mL), and the combined organic extracts were dried over Na_2SO_4, filtered, and concentrated under reduced pressure to afford a crude residue. The crude product was purified by silica gel column chromatography to afford the **S9**.

A flame-dried 100 mL round-bottomed flask equipped with a magnetic stir bar was charged with the compound **S9** (1.0 equiv.), capped with a rubber septum and flushed with argon, was added anhydrous THF (c = 0.5 M) and cooled to 0 ºC. A solution of KO^tBu (0.3 equiv.) in anhydrous THF was added dropwise. The reaction mixture was warmed to room temperature and stirred for 4 h. The resulting mixture was quenched by addition of water and extracted with ethyl acetate (3 × 50 mL). The combined organic fractions were washed with brine (50 mL), dried over Na_2SO_4 and filtrated. After the solvent was removed under reduced pressure, the residue was purified by silica gel column chromatography to afford compound **7u**.

3. Reaction optimization

Table S1. Variation from standard conditions[a]

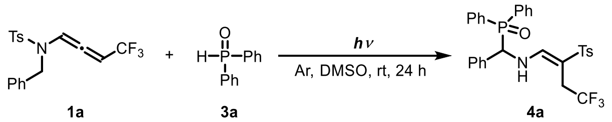

entry	variation from "standard conditions"	yield[b] (%)
1	none	86
2	MeCN	53
3	THF	36
4	ethyl acetate	30
5	CH_2Cl_2	32
6	toluene	60
7	acetone	14
8	in the dark	n.r.
9	in open air	n.r.
10	**3a** (0.2 mmol)	91
11	**3a** (0.3 mmol)	90
12	UV (λ = 360 nm)	73
13	12 h	58
14	DMSO (8 mL)	86
15	*fac*-Ir(ppy)$_3$ as photocatalyst	n.d.[c]
16	dimethyl(phenyl)silane instead of **3a**	n.d.[d]
17	1,3-diphenylpropane-1,3-dione instead of **3a**	n.d.[d]
18	dibenzyl phosphonate instead of **3a**	n.d.[e]

[a]Standard conditions: **1a** (0.2 mmol), **3a** (0.2 mmol, 1.0 equiv.) in DMSO (2 mL) was irradiated for 24 h with blue LEDs (*λ* = 420 nm) under argon atmosphere at room temperature. [b]Isolated yields, n.r. = no reaction, n.d.=not detected. [c]Decomposition of starting material. [d]No corresponding product was detected, and only compound **2** was formed after the reaction. [e]No corresponding product was detected, and only compound **6** was formed after the reaction.

4. General experimental procedure

A 10 mL Pyrex tube equipped with a magnetic stirrer was charged with substrate **1** (0.2 mmol, 1.0 equiv.) and H-phosphine oxide **3** (0.2 mmol, 1.0 equiv.) in DMSO (2 mL). The mixture was bubbled with a stream of argon for 0.5 h. The sample was then irradiated by LEDs (λ = 420 ± 10 nm) for 24 h. After reaction, the solution was extracted with CH_2Cl_2 and washed with brine and dried with anhydrous Na_2SO_4. Upon removal of solvent under vacuum, the residue was purified by column chromatography on silica gel using a mixture petroleum ether/ethyl acetate as eluent or just by washing with diethyl ether and then recrystallization using CH_2Cl_2 and hexane afford the corresponding products **4**.

A 10 mL Pyrex tube equipped with a magnetic stirrer was charged with substrate **7** (0.2 mmol, 1.0 equiv.) and H-phosphine oxide **3** (0.6 mmol, 3.0 equiv.) in DMSO (2 mL). The mixture was bubbled with a stream of argon for 0.5 h. The sample was then irradiated by LEDs (λ = 420 ± 10 nm) for 48 h. After reaction, the solution was extracted with CH_2Cl_2 and washed with brine and dried with anhydrous Na_2SO_4. Upon removal of solvent under vacuum, the residue was purified by column chromatography on silica gel using a mixture petroleum ether/ethyl acetate as eluent afford the corresponding products **8**.

The identity and purity of the product was confirmed by ^{1}H NMR, ^{13}C NMR, ^{19}F NMR and ^{31}P NMR spectroscopic analysis.

5. Optical spectroscopic data

5.1 UV-vis absorption spectra

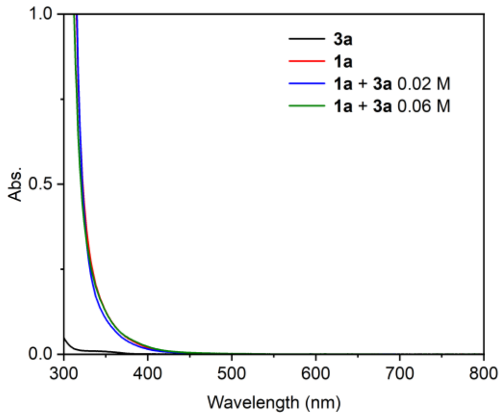

Figure S1. UV-vis absorption spectra of **3a** (2.0×10^{-2} M) in DMSO (black); **1a** (2.0×10^{-2} M) in DMSO (red); **1a** (2.0×10^{-2} M) and **3a** (2.0×10^{-2} M) in DMSO under argon atmosphere (blue); **1a** (2.0×10^{-2} M) and **3a** (6.0×10^{-2} M) in DMSO under argon atmosphere (green).

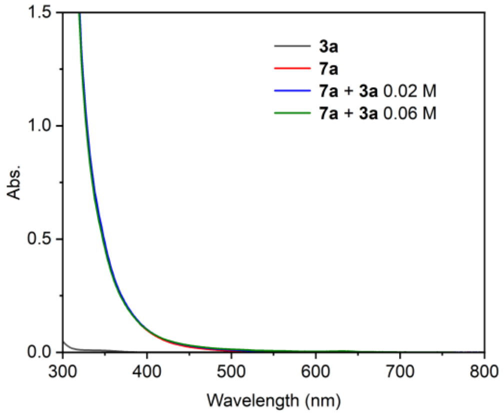

Figure S2. UV-vis absorption spectra of **3a** (2.0×10^{-2} M) in DMSO (black); **7a** (2.0×10^{-2} M) in DMSO (red); **7a** (2.0×10^{-2} M) and **3a** (2.0×10^{-2} M) in DMSO under argon atmosphere (blue); **7a** (2.0×10^{-2} M) and **3a** (6.0×10^{-2} M) in DMSO under argon atmosphere (green).

5.2 Fluorescence spectra

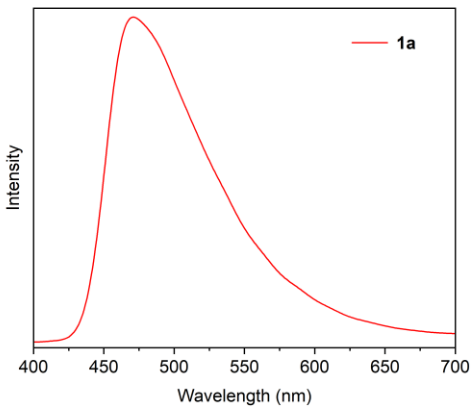

Figure S3. Fluorescence spectra of **1a** (2.0×10^{-2} M) in MeCN under argon atmosphere at 298 K.

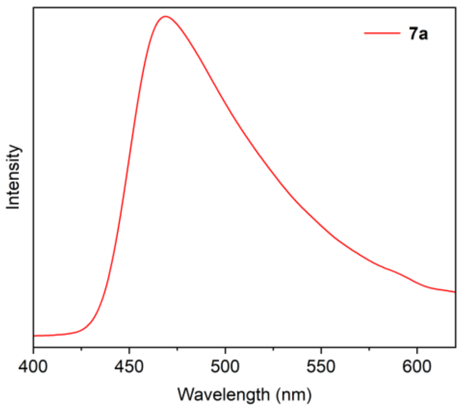

Figure S4. Fluorescence spectra of **7a** (2.0×10^{-2} M) in MeCN under argon atmosphere at 298 K.

5.3 Phosphorescence spectra

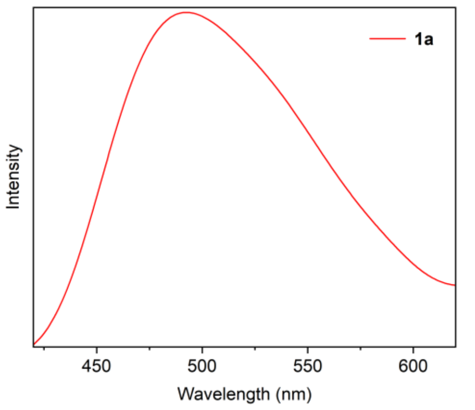

Figure S5. Phosphorescence spectra of **1a** (2.0×10^{-2} M) in 2-methyltetrahydrofuran under argon atmosphere at 77 K with flash delay 1.0 ms.

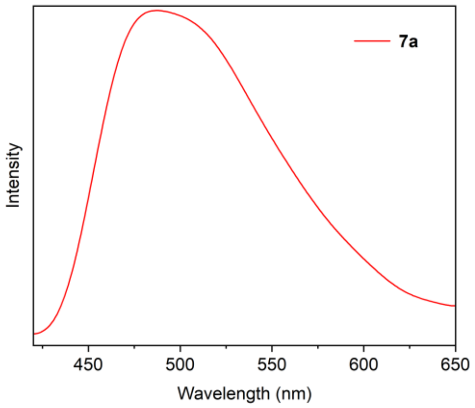

Figure S6. Phosphorescence spectra of **7a** (2.0×10^{-2} M) in 2-methyltetrahydrofuran under argon atmosphere at 77 K with flash delay 1.0 ms.

6. Initial results

6.1 Synthesis of compound 2

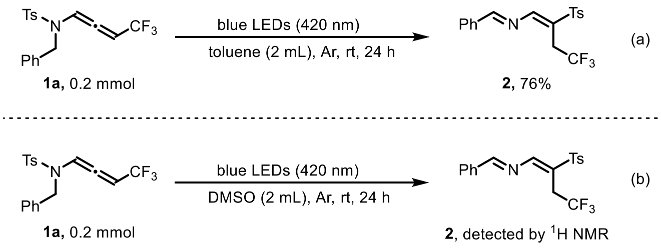

Scheme S1. Synthesis of (*E*)-1-phenyl-*N*-((*E*)-4,4,4-trifluoro-2-tosylbut-1-en-1-yl)methanimine (**2**)

Scheme S1a: A 10 mL Pyrex tube equipped with a magnetic stirrer was charged with substrate **1a** (0.2 mmol, 1.0 equiv.) in toluene (2 mL). The sample was bubbled with a stream of argon for 0.5 h, and then irradiated by LEDs (λ = 420 ± 10 nm) for 24 h. Upon completion of the reaction, the solvent was then removed under vacuum. The residue was purified by flash chromatography afford the corresponding product **2**. ^{1}H NMR yield with hydroquinone as internal standard. White solid; **Melting point**: 98–100 °C; $\mathbf{R_f}$ = 0.26 (petroleum ether : ethyl acetate = 5:1); **^{1}H NMR (500 MHz, DMSO)** δ 8.88 (s, 1H), 8.28 (s, 1H), 7.94 (dd, *J* = 8.5 Hz, *J* = 1.5 Hz, 2H), 7.82 (dt, *J* = 8.5 Hz, *J* = 2.0 Hz, 2H), 7.64–7.60 (m, 1H), 7.56–7.53 (m, 2H), 7.46–7.45 (m, 2H), 3.62 (q, *J* = 11.0 Hz, 2H), 2.40 (s, 3H); **^{13}C NMR (126 MHz, DMSO)** δ 172.2, 154.6, 144.6, 136.9, 135.0, 133.6, 130.1, 129.9, 129.2, 128.8 (q, $J_{C\text{-}F}$ = 2.6 Hz), 127.8, 125.1 (q, $J_{C\text{-}F}$ = 279.3 Hz), 30.4 (q, $J_{C\text{-}F}$ = 31.8 Hz), 21.1; **^{19}F NMR (282 MHz, DMSO)** δ -61.60 (s, 3F); **ESI-HRMS (ESI-TOF):** m/z calcd for $C_{18}H_{16}F_3NNaO_2S^+$ $[M+Na]^+$: 390.0747, found 390.0738.

Scheme S1b: A 10 mL Pyrex tube equipped with a magnetic stirrer was charged with substrate **1a** (0.2 mmol, 1.0 equiv.) in DMSO (2 mL). The sample was bubbled with a stream of argon for 0.5 h, and then irradiated by LEDs (λ = 420 ± 10 nm) for 24 h. After reaction, the solution was extracted with CH_2Cl_2 and washed with brine and dried with anhydrous Na_2SO_4, the solvent was then removed under vacuum. The product **2** was detected by ^{1}H NMR.

6.2 Sequential synthesis of compound 4a

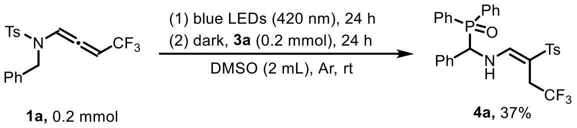

Scheme S2. Sequential synthesis of (*E*)-diphenyl(phenyl((4,4,4-trifluoro-2-tosylbut-1-en-1-yl)amino)methyl)phosphine oxide (**4a**)

A 10 mL Pyrex tube equipped with a magnetic stirrer was charged with substrate **1a** (0.2 mmol, 1.0 equiv.) in DMSO (2 mL). The sample was bubbled with a stream of argon for 0.5 h, and then irradiated by LEDs (λ = 420 ± 10 nm) for 24 h. Upon completion of the reaction, compound **3a** (0.2 mmol, 1.0 equiv.) were added. The mixture was then stirred for 24 h in the dark. After reaction, the solution was extracted with CH_2Cl_2 and washed with brine and dried with anhydrous Na_2SO_4. Upon removal of solvent under vacuum, the

residue was purified just by washing with diethyl ether and then recrystallization using CH_2Cl_2 and hexane afford the corresponding product **4a** (42.2 mg, 37% yield).

7. Scale-up experiment

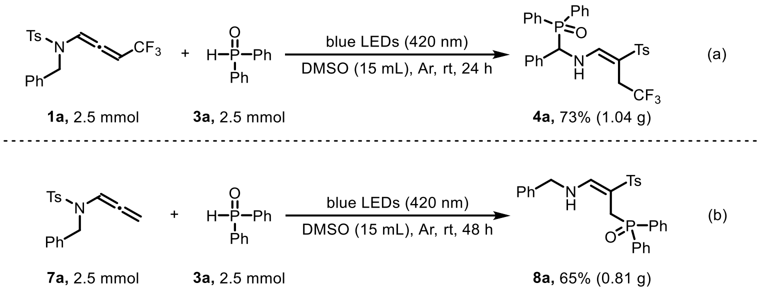

Scheme S3. Scale-up experiment.

Scheme S3a: A 25 mL eggplant-type flask equipped with a magnetic stirrer was charged with substrate **1a** (2.5 mmol, 1.0 equiv.) and **3a** (2.5 mmol, 1.0 equiv.) in DMSO (15 mL). The mixture was bubbled with a stream of argon for 0.5 h. The sample was then irradiated by blue LEDs (λ = 420 nm) 24 h. After reaction, the solution was extracted with CH_2Cl_2 and washed with brine and dried with anhydrous Na_2SO_4. Upon removal of solvent under vacuum, the residue was purified just by washing with diethyl ether and then recrystallization using CH_2Cl_2 and hexane afford the corresponding product **4a** (73%, 1.04 g).

Scheme S3b: A 25 mL eggplant-type flask equipped with a magnetic stirrer was charged with substrate **7a** (2.5 mmol, 1.0 equiv.) and **3a** (2.5 mmol, 1.0 equiv.) in DMSO (15 mL). The mixture was bubbled with a stream of argon for 0.5 h. The sample was then irradiated by blue LEDs (λ = 420 nm) 48 h. After reaction, the solution was extracted with CH_2Cl_2 and washed with brine and dried with anhydrous Na_2SO_4. Upon removal of solvent under vacuum, the residue was purified by column chromatography on silica gel using a mixture petroleum ether/ethyl acetate as eluent afford the corresponding products **8a** (65%, 0.81 g).

8. Photoreaction with using 1s as substrate

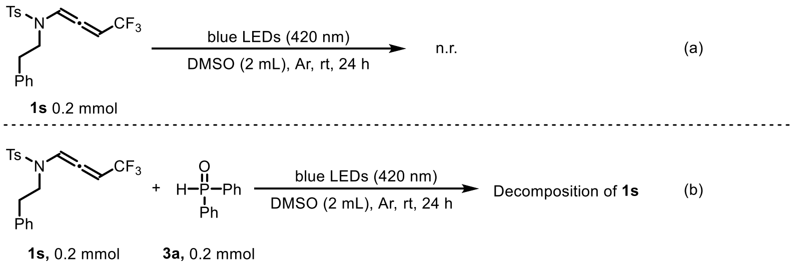

Scheme S4. Photoreaction with using **1s** as substrate.

Scheme S4a: A 10 mL Pyrex tube equipped with a magnetic stirrer was charged with substrate **1s** (0.2 mmol, 1.0 equiv.) in DMSO (2 mL). The sample was bubbled with a stream of argon for 0.5 h, and then irradiated by LEDs (λ = 420 ± 10 nm) for 24 h. After reaction, the solution was extracted with CH_2Cl_2 and washed with brine and dried with anhydrous Na_2SO_4. The residue was purified with chromatography column on silica gel using mixtures of ethyl acetate and petroleum to give the starting material **1s**.

Scheme S4b: A 10 mL Pyrex tube equipped with a magnetic stirrer was charged with substrate **1s** (0.2 mmol, 1.0 equiv.) and H-phosphine oxide **3a** (0.2 mmol, 1.0 equiv.) in DMSO (2 mL). The mixture was bubbled with a stream of argon for 0.5 h. The sample was then irradiated by LEDs (λ = 420 ± 10 nm) for 24 h. After reaction, the solution was extracted with CH_2Cl_2 and washed with brine and dried with anhydrous Na_2SO_4. The starting material **1s** decomposition detected by TLC, and no obvious products that can be separated.

9. Sunlight reaction

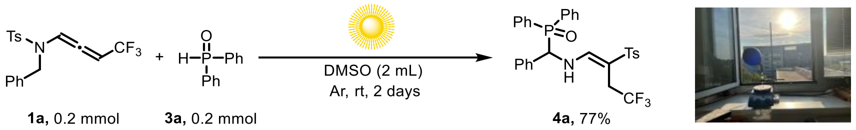

Scheme S5. Sunlight reaction.

A 10 mL Pyrex tube equipped with a magnetic stirrer was charged with substrate **1a** (0.2 mmol, 1.0 equiv.) and **3a** (0.2 mmol, 1.0 equiv.) in DMSO (2 mL). The mixture was bubbled with a stream of argon for 0.5 h. The sample was then irradiated by sunlight for 2 days. After reaction, the solution was extracted with CH_2Cl_2 and washed with brine and dried with anhydrous Na_2SO_4. Upon removal of solvent under vacuum, the residue was purified just by washing with diethyl ether and then recrystallization using CH_2Cl_2 and hexane afford the corresponding product **4a** (87.7 mg, 77% yield). The irradiation was maintained on July 8 and 9, 2023, from 5:00 a.m. till 9:00 p.m. (Longitude: 7.766209, Latitude: 48.580251).

10. Diverse transformations

10.1 Synthesis of compound 5

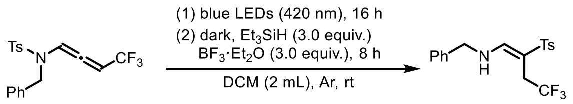

1a, 0.2 mmol **5,** 82%

Scheme S6. Synthesis of (*E*)-*N*-benzyl-4,4,4-trifluoro-2-tosylbut-1-en-1-amine (**5**)

A 10 mL Pyrex tube equipped with a magnetic stirrer was charged with substrate **1a** (0.2 mmol, 1.0 equiv.) in CH_2Cl_2 (2 mL). The sample was bubbled with a stream of argon for 0.5 h, and then irradiated by LEDs (λ = 420 ± 10 nm) for 16 h. Upon completion of the reaction, compound Et_3SiH (3.0 equiv.) and $BF_3\cdot Et_2O$ (3.0 equiv.) were added by syringe under argon. The mixture was then stirred for 8 h in the dark. Upon completion of the reaction, the solvent was then removed under vacuum. The residue was purified with chromatography column on silica gel using mixtures of ethyl acetate and petroleum to give the corresponding products **5** (60.6 mg, 82% yield). White solid; **Melting point**: 88–90°C; **^{1}H NMR (500 MHz, C_6D_6)** δ 7.81 (dt, *J* = 8.5 Hz, *J* = 2.0 Hz, 2H), 7.72 (d, *J* = 14.5 Hz, 1H), 7.08–7.02 (m, 3H), 6.87 (d, *J* = 7.0 Hz, 2H), 6.81 (d, *J* = 8.5 Hz, 2H), 4.53–4.47 (m, 1H), 3.62 (d, *J* = 5.5 Hz, 2H), 2.91 (q, *J* = 11.0 Hz, 2H), 1.88 (s, 3H); **^{13}C NMR (126 MHz, C_6D_6)** δ 149.9, 142.4, 141.3, 138.4, 129.6, 129.0, 127.6, 127.5, 127.2, 126.8 (q, $J_{C\text{-}F}$ = 279.8 Hz), 97.1 (q, $J_{C\text{-}F}$ = 2.8 Hz), 52.2, 30.3 (q, $J_{C\text{-}F}$ = 31.8 Hz), 21.1; **^{19}F NMR (282 MHz, C_6D_6)** δ -63.27 (s, 3F); **ESI-HRMS (ESI-TOF):** m/z calcd for $C_{18}H_{18}F_3NNaO_2S^+$ $[M+Na]^+$: 392.0903, found 392.0897.

10.2 Synthesis of compound 6

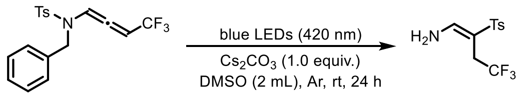

1a, 0.2 mmol **6,** 53%

Scheme S7. (*E*)-4,4,4-trifluoro-2-tosylbut-1-en-1-amine (**6**)

A 10 mL Pyrex tube equipped with a magnetic stirrer was charged with substrate **1a** (0.2 mmol, 1.0 equiv.) and Cs_2CO_3 (0.2 mmol, 1.0 equiv.) in DMSO (2 mL). The mixture was bubbled with a stream of argon for 0.5 h. The sample was then irradiated by LEDs (λ = 420 ± 10 nm) for 24 h. After reaction, the solution was extracted with CH_2Cl_2 and washed with brine and dried with anhydrous Na_2SO_4. Upon removal of solvent under vacuum, the residue was purified with chromatography column on silica gel using mixtures of ethyl acetate and petroleum to give the corresponding products **6** (29.6 mg, 53% yield). Yellow solid; **Melting point**: 92–94°C; **^{1}H NMR (500 MHz, DMSO)** δ 7.61 (dt, *J* = 9.0 Hz, *J* = 2.5 Hz, 2H), 7.53 (t, *J* = 12.0 Hz, 1H), 7.31 (d, *J* = 8.5 Hz, 2H), 6.94 (s, 2H), 3.15 (q, *J* = 11.0 Hz, 2H), 2.34 (s, 3H); **^{13}C NMR (126 MHz, DMSO)** δ 149.4, 142.0, 141.2, 129.4, 126.4 (q, $J_{C\text{-}F}$ = 280.0 Hz), 126.3, 93.7 (q, $J_{C\text{-}F}$ = 2.6 Hz), 28.6 (q, $J_{C\text{-}F}$ = 31.0 Hz), 20.9; **^{19}F NMR (471 MHz, DMSO)** δ -63.09 (s, 3F); **IR (neat):** ν = 3503, 3392, 1645, 1270, 1127, 681, 581 cm^{-1}; **ESI-HRMS (ESI-TOF):** m/z calcd for $C_{11}H_{12}F_3KNO_2S^+$ $[M+K]^+$: 318.0172, found 318.0160.

11. Experiments relevant to the mechanism: terminal *N*-sulfonyl allenamide

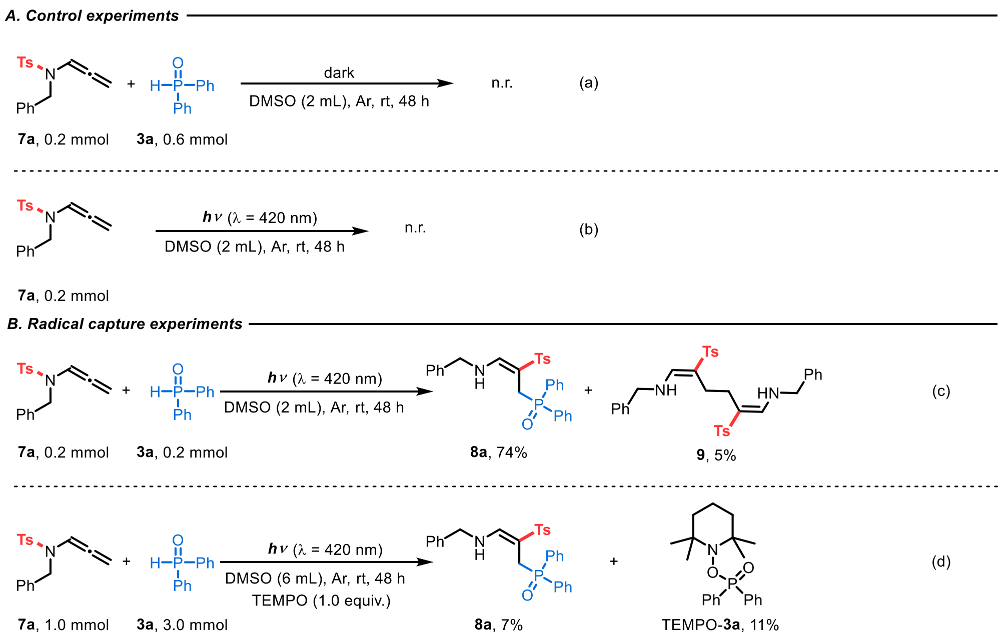

Scheme S8. Experiments relevant to the mechanism: terminal *N*-sulfonyl allenamide

Scheme S8(a): A 10 mL Pyrex tube equipped with a magnetic stirrer was charged with substrate **7a** (0.2 mmol, 1.0 equiv.) and H-phosphine oxide **3a** (0.6 mmol, 3.0 equiv.) in DMSO (2 mL). The mixture was bubbled with a stream of argon for 0.5 h. The sample was then stirred for 48 h in the dark. After reaction, the solution was extracted with CH_2Cl_2 and washed with brine and dried with anhydrous Na_2SO_4. The residue was purified with chromatography column on silica gel using mixtures of ethyl acetate and petroleum to give the starting material **7a**.

Scheme S8(b): A 10 mL Pyrex tube equipped with a magnetic stirrer was charged with substrate **7a** (0.2 mmol) in DMSO (2 mL). The sample was bubbled with a stream of argon for 0.5 h, and then irradiated by LEDs (λ = 420 ± 10 nm) for 48 h. After reaction, the solution was extracted with CH_2Cl_2 and washed with brine and dried with anhydrous Na_2SO_4. The residue was purified with chromatography column on silica gel using mixtures of ethyl acetate and petroleum to give the starting material **7a**.

Scheme S8(c): A 10 mL Pyrex tube equipped with a magnetic stirrer was charged with substrate **7a** (0.2 mmol, 1.0 equiv.) and H-phosphine oxide **3a** (0.2 mmol, 1.0 equiv.) in DMSO (2 mL). The mixture was bubbled with a stream of argon for 0.5 h. The sample was then irradiated by LEDs (λ = 420 ± 10 nm) for 48 h. After reaction, the solution was extracted with CH_2Cl_2 and washed with brine and dried with anhydrous Na_2SO_4. Upon removal of solvent under vacuum, the residue was purified by column chromatography on silica gel using petroleum ether/ethyl acetate mixture as eluent affording the corresponding products **8a** (74.0 mg, 74% yield) and **9** (6.0 mg, 5% yield).

Scheme S8(d): A 10 mL Pyrex tube equipped with a magnetic stirrer was charged with substrate **7a** (1.0 mmol, 1.0 equiv.), H-phosphine oxide **3a** (3.0 mmol, 3.0 equiv.) and TEMPO (1.0 mmol, 1.0 equiv.) in DMSO (6 mL). The mixture was bubbled with a stream of argon for 0.5 h. The sample was then irradiated by LEDs (λ = 420 ± 10 nm) for 48 h. After reaction, the solution was extracted with CH_2Cl_2 and washed with brine and dried with anhydrous Na_2SO_4. Upon removal of solvent under vacuum, the residue was purified by column chromatography on silica gel using petroleum ether/ethyl acetate mixture as eluent affording the corresponding products **8a** (35.0 mg, 7% yield) and TEMPO-**3a** (39.5 mg, 11% yield).

12. Limitations of the method: terminal *N*-sulfonyl allenamide

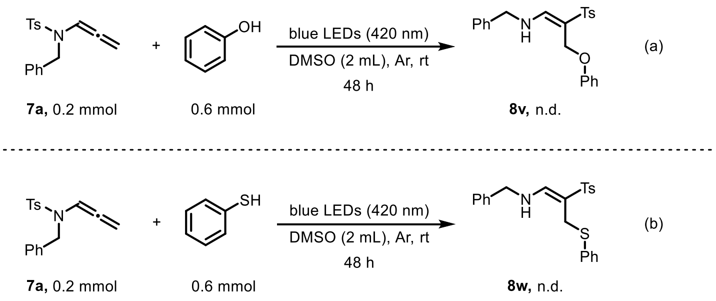

Scheme S9. Experiments relevant to the mechanism: terminal *N*-sulfonyl allenamide

Scheme S9(a): A 10 mL Pyrex tube equipped with a magnetic stirrer was charged with substrate **7a** (0.2 mmol, 1.0 equiv.) and phenol (0.6 mmol, 3.0 equiv.) in DMSO (2 mL). The mixture was bubbled with a stream of argon for 0.5 h, and then irradiated by LEDs (λ = 420 ± 10 nm) for 48 h. After reaction, the solution was extracted with CH_2Cl_2 and washed with brine and dried with anhydrous Na_2SO_4. No corresponding product **8v** were formed.

Scheme S9(b): A 10 mL Pyrex tube equipped with a magnetic stirrer was charged with substrate **7a** (0.2 mmol, 1.0 equiv.) and thiophenol (0.6 mmol, 3.0 equiv.) in DMSO (2 mL). The mixture was bubbled with a stream of argon for 0.5 h, and then irradiated by LEDs (λ = 420 ± 10 nm) for 48 h. After reaction, the solution was extracted with CH_2Cl_2 and washed with brine and dried with anhydrous Na_2SO_4. No corresponding product **8w** were formed.

13. Light on/off experiment

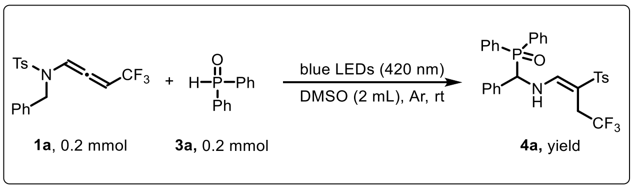

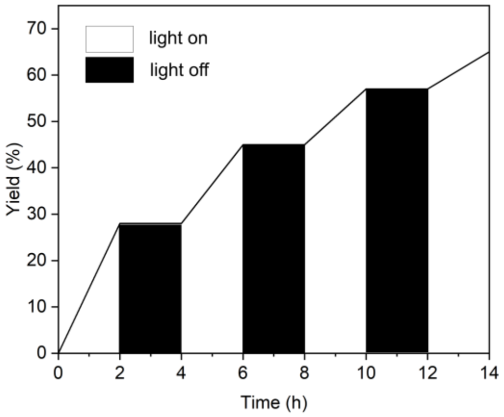

Figure S7. Light on/off experiment.

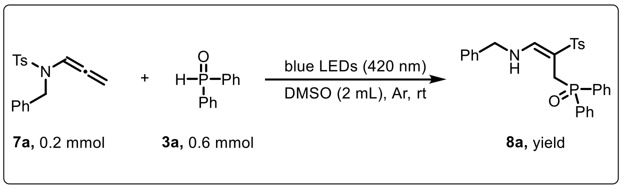

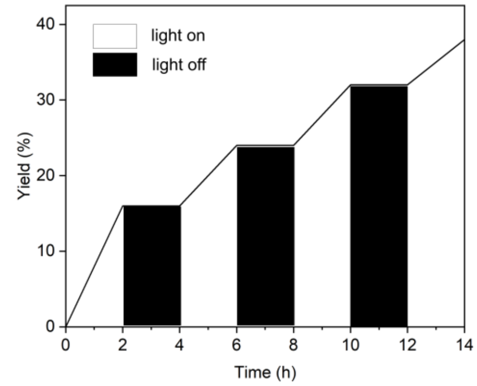

Figure S8. Light on/off experiment.

14. Characterization data for compounds

Compound 1a *N*-benzyl-4-methyl-*N*-(4,4,4-trifluorobuta-1,2-dien-1-yl)benzenesulfonamide

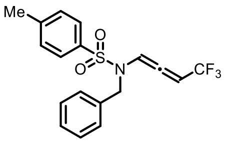

Chemical formula: $C_{18}H_{16}F_3NO_2S$
Molecular weight: 367.39 g.mol^{-1}
White solid
Melting point: 88–90 °C

^{1}H NMR (300 MHz, CDCl$_3$) δ 7.79–7.67 (m, 2H), 7.45–7.38 (m, 1H), 7.38–7.32 (m, 2H), 7.30–7.18 (m, 5H), 5.68 (p, *J* = 5.6 Hz, 1H), 4.46 (d, *J* = 15.3 Hz, 1H), 4.12 (d, *J* = 15.3 Hz, 1H), 2.46 (s, 3H).
Data match with those described in the literature.[6]

Compound 1b 4-Methyl-*N*-(4-methylbenzyl)-*N*-(4,4,4-trifluorobuta-1,2-dien-1-yl)benzenesulfonamide

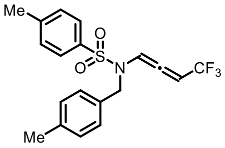

Chemical formula: $C_{19}H_{18}F_3NO_2S$
Molecular weight: 381.41 g.mol^{-1}
White solid
Melting point: 86–88 °C

^{1}H NMR (500 MHz, CDCl$_3$) δ 7.73–7.71 (m, 2H), 7.41–7.37 (m, 1H), 7.36–7.30 (m, 2H), 7.18–7.05 (m, 4H), 5.70 (p, *J* = 5.5 Hz, 1H), 4.47 (d, *J* = 15.0 Hz, 1H), 4.06 (d, *J* = 15.0 Hz, 1H), 2.46 (s, 3H), 2.32 (s, 3H).
^{13}C NMR (126 MHz, CDCl$_3$) δ 199.2 (q, $J_{C\text{-}F}$ = 6.0 Hz), 144.6, 137.7, 135.2, 131.7, 130.1, 129.3, 127.8, 127.3, 121.3 (q, $J_{C\text{-}F}$ = 272.2 Hz), 106.9, 96.4 (q, $J_{C\text{-}F}$ = 39.3 Hz), 50.9, 21.7, 21.2.
^{19}F NMR (282 MHz, CDCl$_3$) δ -62.68 (s, 3F).
IR (neat): ν = 3032, 1428, 1362, 1119, 898, 745, 540 cm^{-1}.
ESI-HRMS (ESI-TOF): m/z calcd for $C_{19}H_{18}F_3NNaO_2S^+$ [M+Na]$^+$: 404.0903, found 404.0904.

Compound 1c *N*-(4-methoxybenzyl)-4-methyl-*N*-(4,4,4-trifluorobuta-1,2-dien-1-yl)benzenesulfonamide

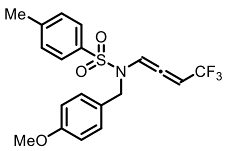

Chemical formula: $C_{19}H_{18}F_3NO_3S$
Molecular weight: 397.41 g.mol^{-1}
White solid
Melting point: 97–99 °C

^{1}H NMR (500 MHz, CDCl$_3$) δ 7.72–7.70 (m, 2H), 7.39–7.33 (m, 3H), 7.18–7.15 (m, 2H), 6.82 (dt, *J* = 9.0 Hz, *J* = 3.0 Hz, 2H), 5.72 (p, *J* = 5.5 Hz, 1H), 4.42 (d, *J* = 16.0 Hz, 1H), 4.04 (d, *J* = 15.0 Hz, 1H), 3.78 (s, 3H), 2.45 (s, 3H).
^{13}C NMR (126 MHz, CDCl$_3$) δ 199.0 (q, $J_{C\text{-}F}$ = 5.9 Hz), 159.3, 144.5, 135.1, 130.0, 129.1, 127.2, 126.6, 121.2 (q, $J_{C\text{-}F}$ = 272.2 Hz), 113.9, 106.6, 96.2 (q, $J_{C\text{-}F}$ = 39.3 Hz), 55.3, 50.5, 21.6.
^{19}F NMR (282 MHz, CDCl$_3$) δ -62.64 (s, 3F).
IR (neat): ν = 3032, 1429, 1362, 1041, 898, 783, 538 cm^{-1}.
ESI-HRMS (ESI-TOF): m/z calcd for $C_{19}H_{18}F_3NNaO_3S^+$ [M+Na]$^+$: 420.0852, found 420.0848.

Compound 1d 4-Methyl-*N*-(4,4,4-trifluorobuta-1,2-dien-1-yl)-*N*-(4-(trifluoromethyl)benzyl)benzenesulfonamide

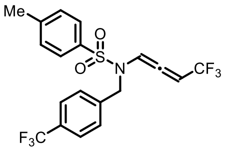

Chemical formula: $C_{19}H_{15}F_6NO_2S$
Molecular weight: 435.38 g.mol^{-1}
White solid
Melting point: 102–104 °C

^{1}H NMR (500 MHz, CDCl$_3$) δ 7.72 (d, *J* = 8.0 Hz, 2H), 7.54 (d, *J* = 8.0 Hz, 2H), 7.45–7.43 (m, 1H), 7.36 (d, *J* = 8.0 Hz, 4H), 5.71 (p, *J* = 5.5 Hz, 1H), 4.45 (d, *J* = 16.0 Hz, 1H), 4.26 (d, *J* = 15.5 Hz, 1H), 2.46 (s, 3H).
^{13}C NMR (126 MHz, CDCl$_3$) δ 198.8 (q, $J_{C\text{-}F}$ = 5.8 Hz), 145.0, 139.0, 134.9, 130.3 (q, $J_{C\text{-}F}$ = 32.6 Hz), 130.3, 127.9, 127.3, 125.7 (q, $J_{C\text{-}F}$ = 3.8 Hz), 124.1 (q, $J_{C\text{-}F}$ = 272.5 Hz), 120.6 (q, $J_{C\text{-}F}$ = 272.2 Hz), 106.9, 96.9 (q, $J_{C\text{-}F}$ = 39.6 Hz), 50.6, 21.8.
^{19}F NMR (471 MHz, CDCl$_3$) δ -62.65 (s, 3F), -62.84 (s, 3F).
IR (neat): ν = 3043, 1451, 1351, 1124, 959, 704, 544 cm^{-1}.
ESI-HRMS (ESI-TOF): m/z calcd for $C_{19}H_{15}F_6NNaO_2S^+$ [M+Na]$^+$: 458.0620, found 458.0613.

Compound 1e 4-Methyl-*N*-(4,4,4-trifluorobuta-1,2-dien-1-yl)-*N*-(4-(trifluoromethoxy)benzyl)benzenesulfonamide

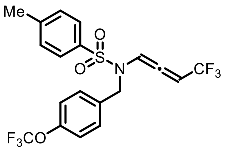

Chemical formula: $C_{19}H_{15}F_6NO_3S$
Molecular weight: 451.38 g.mol^{-1}
White solid
Melting point: 79–81 °C

^{1}H NMR (500 MHz, C_6D_6) δ 7.51 (dt, *J* = 8.5 Hz, *J* = 2.0 Hz, 2H), 7.24–7.21 (m, 1H), 6.95 (dt, *J* = 9.0 Hz, *J* = 3.0 Hz, 2H), 6.83 (d, *J* = 8.0 Hz, 2H), 6.75 (d, *J* = 8.5 Hz, 2H), 5.11 (p, *J* = 5.5 Hz, 1H), 3.97 (d, *J* = 15.5 Hz, 1H), 3.88 (d, *J* = 15.5 Hz, 1H), 1.86 (s, 3H).
^{13}C NMR (126 MHz, C_6D_6) δ 199.0 (q, $J_{C\text{-}F}$ = 5.9 Hz), 149.0 (q, $J_{C\text{-}F}$ = 1.6 Hz), 144.4, 135.8, 134.0, 130.1, 129.3, 127.4, 121.7 (q, $J_{C\text{-}F}$ = 272.2 Hz), 121.3, 121.2 (q, $J_{C\text{-}F}$ = 257.3 Hz), 107.1, 96.2 (q, $J_{C\text{-}F}$ = 39.1 Hz), 50.3, 21.1.
^{19}F NMR (282 MHz, C_6D_6) δ -57.85 (s, 3F), -62.84 (s, 3F).
IR (neat): ν = 3049, 1452, 1342, 1122, 949, 810, 543 cm^{-1}.
ESI-HRMS (ESI-TOF): m/z calcd for $C_{19}H_{16}F_6NO_3S^+$ $[M+H]^+$: 452.0750, found 452.0755.

Compound 1f *N*-(4-fluorobenzyl)-4-methyl-*N*-(4,4,4-trifluorobuta-1,2-dien-1-yl)benzenesulfonamide

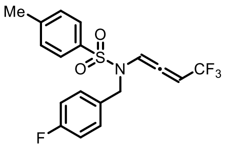

Chemical formula: $C_{18}H_{15}F_4NO_2S$
Molecular weight: 385.38 g.mol^{-1}
White solid
Melting point: 99–101 °C

^{1}H NMR (500 MHz, $CDCl_3$) δ 7.72 (dt, *J* = 8.0 Hz, *J* = 2.5 Hz, 2H), 7.42–7.39 (m, 1H), 7.36 (d, *J* = 11.0 Hz, 2H), 7.23–7.20 (m, 2H), 7.00–6.95 (m, 2H), 5.71 (p, *J* = 6.0 Hz, 1H), 4.37 (d, *J* = 15.0 Hz, 1H), 4.14 (d, *J* = 15.0 Hz, 1H), 2.46 (s, 3H).
Data match with those described in the literature.[7]

Compound 1g *N*-(4-chlorobenzyl)-4-methyl-*N*-(4,4,4-trifluorobuta-1,2-dien-1-yl)benzenesulfonamide

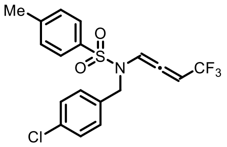

Chemical formula: $C_{18}H_{15}ClF_3NO_2S$
Molecular weight: 401.83 g.mol^{-1}
White solid
Melting point: 94–96 °C

^{1}H NMR (500 MHz, $CDCl_3$) δ 7.72 (dt, *J* = 8.5 Hz, *J* = 2.0 Hz, 2H), 7.42–7.39 (m, 1H), 7.35 (d, *J* = 8.0 Hz, 2H), 7.25 (dt, *J* = 8.5 Hz, *J* = 2.5 Hz, 2H), 7.18 (dt, *J* = 8.5 Hz, *J* = 2.5 Hz, 2H), 5.71 (p, *J* = 5.5 Hz, 1H), 4.39 (d, *J* = 15.5 Hz, 1H), 4.12 (d, *J* = 15.5 Hz, 1H), 2.46 (s, 3H).
^{13}C NMR (126 MHz, $CDCl_3$) δ 198.9 (q, $J_{C\text{-}F}$ = 5.8 Hz), 144.9, 135.0, 133.8, 133.4, 130.3, 129.1, 128.8, 127.3, 121.1 (q, $J_{C\text{-}F}$ = 272.2 Hz), 106.8, 96.7 (q, $J_{C\text{-}F}$ = 39.4 Hz), 50.4, 21.7.
^{19}F NMR (282 MHz, $CDCl_3$) δ -62.73 (s, 3F).
IR (neat): ν = 3046, 1452, 1342, 1122, 947, 815, 542 cm^{-1}.
ESI-HRMS (ESI-TOF): m/z calcd for $C_{18}H_{15}ClF_3NNaO_2S^+$ $[M+Na]^+$: 424.0357, found 424.0353.

Compound 1h *N*-(4-bromobenzyl)-4-methyl-*N*-(4,4,4-trifluorobuta-1,2-dien-1-yl)benzenesulfonamide

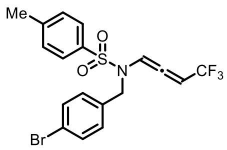

Chemical formula: $C_{18}H_{15}BrF_3NO_2S$
Molecular weight: 446.28 g.mol^{-1}
White solid
Melting point: 95–97 °C

^{1}H NMR (500 MHz, $CDCl_3$) δ 7.71 (dt, *J* = 8.5 Hz, *J* = 2.0 Hz, 2H), 7.42–7.40 (m, 3H), 7.36–7.34 (m, 2H), 7.12 (dt, *J* = 8.5 Hz, *J* = 2.0 Hz, 2H), 5.71 (p, *J* = 5.5 Hz, 1H), 4.38 (d, *J* = 15.5 Hz, 1H), 4.09 (d, *J* = 15.5 Hz, 1H), 2.46 (s, 3H).
^{13}C NMR (126 MHz, $CDCl_3$) δ 198.9 (q, $J_{C\text{-}F}$ = 5.9 Hz), 144.9, 135.0, 133.9, 131.8, 130.3, 129.4, 127.3, 121.9, 121.1 (q, $J_{C\text{-}F}$ = 272.3 Hz), 106.9, 96.7 (q, $J_{C\text{-}F}$ = 39.4 Hz), 50.5, 21.8.
^{19}F NMR (282 MHz, $CDCl_3$) δ -62.71 (s, 3F).
IR (neat): ν = 3039, 1430, 1312, 1119, 898, 665, 595 cm^{-1}.
ESI-HRMS (ESI-TOF): m/z calcd for $C_{18}H_{15}BrF_3NNaO_2S^+$ $[M+Na]^+$: 467.9851, found 467.9851.

Compound 1i 4-Methyl-*N*-(2-methylbenzyl)-*N*-(4,4,4-trifluorobuta-1,2-dien-1-yl)benzenesulfonamide

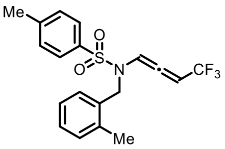

Chemical formula: $C_{19}H_{18}F_3NO_2S$
Molecular weight: 381.41 g.mol^{-1}
White solid
Melting point: 112–114 °C

^{1}H NMR (500 MHz, CDCl$_3$) δ 7.73 (d, *J* = 8.5 Hz, 2H), 7.44–7.40 (m, 1H), 7.36 (d, *J* = 8.0 Hz, 2H), 7.26–7.24 (m, 1H), 7.17–7.08 (m, 3H), 5.61 (p, *J* = 5.5 Hz, 1H), 4.46 (d, *J* = 16.0 Hz, 1H), 4.17 (d, *J* = 15.5 Hz, 1H), 2.47 (s, 3H), 2.22 (s, 3H).
^{13}C NMR (126 MHz, CDCl$_3$) δ 199.0 (q, $J_{C\text{-}F}$ = 5.9 Hz), 144.7, 135.5, 135.0, 132.2, 130.5, 130.2, 127.7, 127.3, 127.0, 126.2, 121.2 (q, $J_{C\text{-}F}$ = 272.2 Hz), 106.8, 96.1 (q, $J_{C\text{-}F}$ = 39.4 Hz), 48.7, 21.7, 19.2.
^{19}F NMR (471 MHz, CDCl$_3$) δ -62.68 (s, 3F).
IR (neat): ν = 3045, 1450, 1343, 1118, 948, 764, 599 cm^{-1}.
ESI-HRMS (ESI-TOF): m/z calcd for $C_{19}H_{18}F_3NNaO_2S^+$ [M+Na]$^+$: 404.0903, found 404.0901.

Compound 1j *N*-(2-fluorobenzyl)-4-methyl-*N*-(4,4,4-trifluorobuta-1,2-dien-1-yl)benzenesulfonamide

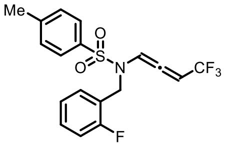

Chemical formula: $C_{18}H_{15}F_4NO_2S$
Molecular weight: 385.38 g.mol^{-1}
White solid
Melting point: 85–87 °C

^{1}H NMR (300 MHz, CDCl$_3$) δ 7.74 (dt, *J* = 8.4 Hz, *J* = 1.8 Hz, 2H), 7.45–7.34 (m, 4H), 7.28–7.21 (m, 1H), 7.14–7.08 (m, 1H), 7.00–6.94 (m, 1H), 5.71 (p, *J* = 5.4 Hz, 1H), 4.44–4.30 (m, 2H), 2.46 (s, 3H).
^{13}C NMR (126 MHz, CDCl$_3$) δ 198.3 (q, $J_{C\text{-}F}$ = 5.8 Hz), 160.4 (d, $J_{C\text{-}F}$ = 247.1 Hz), 144.9, 135.0, 130.3, 129.6 (d, $J_{C\text{-}F}$ = 8.3 Hz), 129.0 (d, $J_{C\text{-}F}$ = 3.4 Hz), 127.3, 124.5 (d, $J_{C\text{-}F}$ = 3.7 Hz), 121.9 (d, $J_{C\text{-}F}$ = 13.4 Hz), 121.1 (q, $J_{C\text{-}F}$ = 272.3 Hz), 115.4 (d, $J_{C\text{-}F}$ = 21.4 Hz), 106.6, 96.6 (q, $J_{C\text{-}F}$ = 39.6 Hz), 44.1 (d, $J_{C\text{-}F}$ = 5.3 Hz), 21.8.
^{19}F NMR (282 MHz, CDCl$_3$) δ -62.90 (s, 3F), -118.63 (s, 1F).
IR (neat): ν = 3036, 1429, 1355, 1102, 945, 759, 545 cm^{-1}.
ESI-HRMS (ESI-TOF): m/z calcd for $C_{18}H_{15}F_4NNaO_2S^+$ [M+Na]$^+$: 408.0652, found 408.0648.

Compound 1k *N*-(2-chlorobenzyl)-4-methyl-*N*-(4,4,4-trifluorobuta-1,2-dien-1-yl)benzenesulfonamide

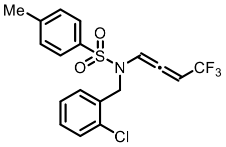

Chemical formula: $C_{18}H_{15}ClF_3NO_2S$
Molecular weight: 401.83 g.mol^{-1}
White solid
Melting point: 86–88 °C

^{1}H NMR (400 MHz, CDCl$_3$) δ 7.75 (dt, *J* = 8.4 Hz, *J* = 2.0 Hz, 2H), 7.46–7.42 (m, 2H), 7.38 (d, *J* = 8.0 Hz, 2H), 7.30 (dd, *J* = 7.6 Hz, *J* = 1.6 Hz, 1H), 7.24–7.17 (m, 2H), 5.68 (p, *J* = 5.6 Hz, 1H), 4.43 (s, 2H), 2.47 (s, 3H).
^{13}C NMR (126 MHz, CDCl$_3$) δ 198.4 (q, $J_{C\text{-}F}$ = 5.9 Hz), 144.9, 135.0, 132.6, 132.1, 130.3, 129.5, 129.0, 128.1, 127.3, 127.2, 121.0 (q, $J_{C\text{-}F}$ = 272.4 Hz), 106.8, 96.6 (q, $J_{C\text{-}F}$ = 39.6 Hz), 48.2, 21.8.
^{19}F NMR (282 MHz, CDCl$_3$) δ -62.80 (s, 3F).
IR (neat): ν = 3019, 1446, 1351, 1110, 921, 753, 540 cm^{-1}.
ESI-HRMS (ESI-TOF): m/z calcd for $C_{18}H_{15}ClF_3NNaO_2S^+$ [M+Na]$^+$: 424.0357, found 424.0353.

Compound 1l *N*-(2-bromobenzyl)-4-methyl-*N*-(4,4,4-trifluorobuta-1,2-dien-1-yl)benzenesulfonamide

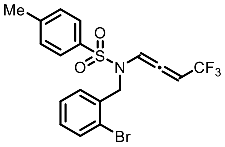

Chemical formula: $C_{18}H_{15}BrF_3NO_2S$
Molecular weight: 446.28 g.mol^{-1}
White solid
Melting point: 94–96 °C

^{1}H NMR (500 MHz, CDCl$_3$) δ 7.75 (dt, *J* = 8.5 Hz, *J* = 2.0 Hz, 2H), 7.49 (dd, *J* = 8.0 Hz, *J* = 1.5 Hz, 1H), 7.46–7.44 (m, 1H), 7.42–7.41 (m, 1H), 7.39–7.36 (m, 2H), 7.30–7.27 (m, 1H), 7.14–7.10 (m, 1H), 5.69 (p, *J* = 5.5 Hz, 1H), 4.41 (s, 2H), 2.47 (s, 3H).
Data match with those described in the literature.[7]

Compound 1m *N*-(benzo[*d*][1,3]dioxol-5-ylmethyl)-4-methyl-*N*-(4,4,4-trifluorobuta-1,2-dien-1-yl)benzenesulfonamide

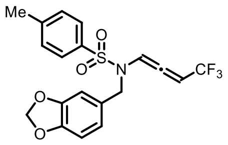

Chemical formula: $C_{19}H_{16}F_3NO_4S$
Molecular weight: 411.39 g.mol^{-1}
White solid
Melting point: 110–112 °C

^{1}H NMR (400 MHz, CDCl$_3$) δ 7.71 (d, *J* = 8.1 Hz, 2H), 7.41–7.37 (m, 1H), 7.35 (d, *J* = 8.1 Hz, 2H), 6.77 (d, *J* = 1.7 Hz, 1H), 6.73–6.62 (m, 2H), 5.93 (s, 2H), 5.75 (p, *J* = 5.6 Hz, 1H), 4.34 (d, *J* = 15.0 Hz, 1H), 4.06 (d, *J* = 15.0 Hz, 1H), 2.45 (s, 3H).
Data match with those described in the literature.[6]

Compound 1n *N*-(3,5-difluorobenzyl)-4-methyl-*N*-(4,4,4-trifluorobuta-1,2-dien-1-yl)benzenesulfonamide

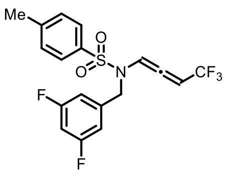

Chemical formula: $C_{18}H_{14}F_5NO_2S$
Molecular weight: 403.37 g.mol^{-1}
White solid
Melting point: 84–86 °C

^{1}H NMR (300 MHz, CDCl$_3$) δ 7.72 (dt, *J* = 8.4 Hz, *J* = 1.8 Hz, 2H), 7.46–7.41 (m, 1H), 7.39–7.35 (m, 2H), 6.79–6.66 (m, 3H), 5.75 (p, *J* = 5.4 Hz, 1H), 4.34–4.19 (m, 2H), 2.47 (s, 3H).
^{13}C NMR (126 MHz, CDCl$_3$) δ 198.7 (q, $J_{C\text{-}F}$ = 5.7 Hz), 163.3 (dd, $J_{C\text{-}F}$ = 249.7 Hz, $J_{C\text{-}F}$ = 12.7 Hz), 145.1, 138.9 (t, $J_{C\text{-}F}$ = 9.1 Hz), 134.9, 130.4, 127.3, 121.0 (q, $J_{C\text{-}F}$ = 272.3 Hz), 110.4 (dd, $J_{C\text{-}F}$ = 20.0 Hz, $J_{C\text{-}F}$ = 6.3 Hz), 106.8, 103.5 (t, $J_{C\text{-}F}$ = 25.3 Hz), 96.9 (q, $J_{C\text{-}F}$ = 39.4 Hz), 50.3 (t, $J_{C\text{-}F}$ = 2.4 Hz), 21.8.
^{19}F NMR (282 MHz, CDCl$_3$) δ -62.91 (s, 3F), -109.28 (s, 2F).
IR (neat): ν = 3020, 1445, 1353, 1119, 917, 684, 541 cm^{-1}.
ESI-HRMS (ESI-TOF): m/z calcd for $C_{18}H_{14}F_5NNaO_2S^+$ [M+Na]$^+$: 426.0558, found 426.0549.

Compound 1o *N*-(3,4-dichlorobenzyl)-4-methyl-*N*-(4,4,4-trifluorobuta-1,2-dien-1-yl)benzenesulfonamide

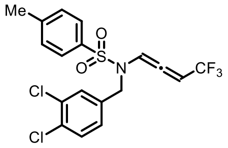

Chemical formula: $C_{18}H_{14}Cl_2F_3NO_2S$
Molecular weight: 436.27 g.mol^{-1}
White solid
Melting point: 100–102 °C

^{1}H NMR (500 MHz, CDCl$_3$) δ 7.70 (dt, *J* = 8.5 Hz, *J* = 2.0 Hz, 2H), 7.44–7.41 (m, 1H), 7.36–7.34 (m, 3H), 7.27 (d, *J* = 2.5 Hz, 1H), 7.07 (dd, *J* = 8.5 Hz, *J* = 2.5 Hz, 1H), 5.75 (p, *J* = 5.5 Hz, 1H), 4.34 (d, *J* = 15.5 Hz, 1H), 4.18 (d, *J* = 15.5 Hz, 1H), 2.46 (s, 3H).
^{13}C NMR (126 MHz, CDCl$_3$) δ 198.7 (q, $J_{C\text{-}F}$ = 5.9 Hz), 145.1, 135.2, 134.9, 132.9, 132.1, 130.6, 130.3, 129.6, 127.2, 127.0, 121.1 (q, $J_{C\text{-}F}$ = 272.3 Hz), 106.9, 96.9 (q, $J_{C\text{-}F}$ = 39.3 Hz), 49.9, 21.7.
^{19}F NMR (471 MHz, CDCl$_3$) δ -62.75 (s, 3F).
IR (neat): ν = 3013, 1428, 1359, 1098, 919, 662, 586 cm^{-1}.
ESI-HRMS (ESI-TOF): m/z calcd for $C_{18}H_{14}Cl_2F_3NNaO_2S^+$ [M+Na]$^+$: 457.9967, found 457.9961.

Compound 1q 4-Methyl-*N*-(naphthalen-1-ylmethyl)-*N*-(4,4,4-trifluorobuta-1,2-dien-1-yl)benzenesulfonamide

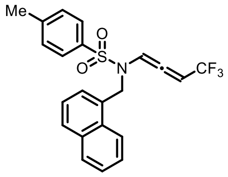

Chemical formula: $C_{22}H_{18}F_3NO_2S$
Molecular weight: 417.45 g.mol^{-1}
White solid
Melting point: 108–110 °C

^{1}H NMR (500 MHz, CDCl$_3$) δ 8.01–7.99 (m, 1H), 7.87–7.85 (m, 1H), 7.79–7.75 (m, 3H), 7.53–7.45 (m, 4H), 7.41–7.38 (m, 1H), 7.35–7.33 (m, 2H), 5.55 (p, *J* = 5.5 Hz, 1H), 4.95 (d, *J* = 15.5 Hz, 1H), 4.67 (d, *J* = 15.5 Hz, 1H), 2.46 (s, 3H).
^{13}C NMR (126 MHz, CDCl$_3$) δ 199.1 (q, $J_{C\text{-}F}$ = 5.9 Hz), 144.7, 134.7, 133.8, 130.9, 130.1, 129.3, 128.9, 128.7, 127.4, 126.4, 125.9, 125.9, 125.2, 122.7, 121.1 (q, $J_{C\text{-}F}$ = 272.4 Hz), 106.8, 96.2 (q, $J_{C\text{-}F}$ = 39.3 Hz), 49.1, 21.7.
^{19}F NMR (471 MHz, CDCl$_3$) δ -62.53 (s, 3F).
IR (neat): ν = 3031, 1442, 1353, 1114, 903, 778, 545 cm^{-1}.
ESI-HRMS (ESI-TOF): m/z calcd for $C_{22}H_{18}F_3NNaO_2S^+$ [M+Na]$^+$: 440.0903, found 440.0896.

Compound 1r *N*-benzyl-*N*-(4,4-difluorobuta-1,2-dien-1-yl)-4-methylbenzenesulfonamide

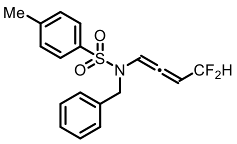

Chemical formula: $C_{18}H_{17}F_2NO_2S$
Molecular weight: 349.40 g.mol^{-1}
Colorless oil

^{1}H NMR (CDCl$_3$, 500 MHz) δ 7.76 (d, *J* = 8.3 Hz, 2H), 7.39 (d, *J* = 8.0 Hz, 2H), 7.35–7.26 (m, 5H), 7.23 (dd, *J* = 11.0 Hz, *J* = 5.7 Hz, 1H), 5.68 (p, *J* = 6.0 Hz, 1H), 5.39 (td, *J* = 56.0 Hz, *J* = 6.0 Hz, 1H), 4.48 (d, *J* = 15.1 Hz, 1H), 4.18 (d, *J* = 15.1 Hz, 1H), 2.49 (s, 3H).
Data match with those described in the literature.[4]

Compound 1s 4-Methyl-*N*-phenethyl-*N*-(4,4,4-trifluorobuta-1,2-dien-1-yl)benzenesulfonamide

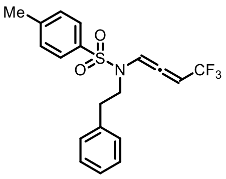

Chemical formula: $C_{19}H_{18}F_3NO_2S$
Molecular weight: 381.41 g.mol^{-1}
White solid
Melting point: 90–92 °C

^{1}H NMR (500 MHz, CDCl$_3$) δ 7.74–7.63 (m, 2H), 7.46 (dq, *J* = 6.2 Hz, *J* = 3.1 Hz, 1H), 7.32 (d, *J* = 8.1 Hz, 2H), 7.31–7.27 (m, 2H), 7.24–7.18 (m, 1H), 7.17–7.09 (m, 2H), 5.99 (p, *J* = 5.6 Hz, 1H), 3.37–3.23 (m, 2H), 2.90–2.77 (m, 2H), 2.43 (s, 3H).
Data match with those described in the literature.[6]

Compound 1t *N*-benzyl-*N*-(4,4,4-trifluorobuta-1,2-dien-1-yl)-2-(trifluoromethyl)benzenesulfonamide

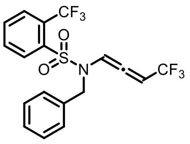

Chemical formula: $C_{18}H_{13}F_6NO_2S$
Molecular weight: 421.36 g.mol^{-1}
White solid
Melting point: 99–101 °C

^{1}H NMR (500 MHz, CDCl$_3$) δ 7.97 (d, *J* = 7.5 Hz, 1H), 7.86 (d, *J* = 6.0 Hz, 1H), 7.68–7.61 (m, 2H), 7.37–7.34 (m, 1H), 7.22–7.15 (m, 5H), 5.68 (p, *J* = 5.5 Hz, 1H), 4.57 (d, *J* = 15.5 Hz, 1H), 4.30 (d, *J* = 15.5 Hz, 1H).
^{13}C NMR (126 MHz, CDCl$_3$) δ 198.2 (q, $J_{C\text{-}F}$ = 5.9 Hz), 137.9, 134.4, 133.5, 132.7, 131.1, 129.0 (q, $J_{C\text{-}F}$ = 6.4 Hz), 128.7, 128.3 (q, $J_{C\text{-}F}$ = 33.5 Hz), 128.1, 127.6, 122.5 (q, $J_{C\text{-}F}$ = 274.7 Hz), 121.2 (q, $J_{C\text{-}F}$ = 272.2 Hz), 106.8, 96.9 (q, $J_{C\text{-}F}$ = 39.4 Hz), 51.5.
^{19}F NMR (282 MHz, CDCl$_3$) δ -57.64 (s, 3F), -62.73 (s, 3F).
IR (neat): ν = 3018, 1455, 1350, 1028, 878, 734, 646 cm^{-1}.
ESI-HRMS (ESI-TOF): m/z calcd for $C_{18}H_{13}F_6NNaO_2S^+$ [M+Na]$^+$: 444.0463, found 444.0464.

Compound 1u *N*-benzyl-2,6-difluoro-*N*-(4,4,4-trifluorobuta-1,2-dien-1-yl)benzenesulfonamide

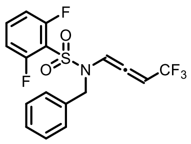

Chemical formula: $C_{17}H_{12}F_5NO_2S$
Molecular weight: 389.34 g.mol^{-1}
Colorless oil

^{1}H NMR (500 MHz, CDCl$_3$) δ 7.56–7.50 (m, 1H), 7.43–7.41 (m, 1H), 7.27–7.23 (m, 5H), 7.04–7.00 (m, 2H), 5.77 (p, *J* = 5.5 Hz, 1H), 4.67 (d, *J* = 15.5 Hz, 1H), 4.41 (d, *J* = 15.5 Hz, 1H).
^{13}C NMR (126 MHz, CDCl$_3$) δ 198.4 (q, $J_{C\text{-}F}$ = 6.0 Hz), 159.6 (dd, $J_{C\text{-}F}$ = 260.8 Hz, $J_{C\text{-}F}$ = 3.7 Hz), 135.6 (t, $J_{C\text{-}F}$ = 11.1 Hz), 134.4, 128.7, 128.1, 127.8, 121.2 (q, $J_{C\text{-}F}$ = 272.2 Hz), 116.8 (t, $J_{C\text{-}F}$ = 16.0 Hz), 113.5 (dd, $J_{C\text{-}F}$ = 22.9 Hz, $J_{C\text{-}F}$ = 4.2 Hz), 106.5, 96.9 (q, $J_{C\text{-}F}$ = 39.4 Hz), 51.2 (t, $J_{C\text{-}F}$ = 1.5 Hz).
^{19}F NMR (282 MHz, CDCl$_3$) δ -62.70 (s, 3F), -105.45 (s, 2F).
IR (neat): ν = 3062, 1469, 1374, 1124, 933, 789, 597 cm^{-1}.
ESI-HRMS (ESI-TOF): m/z calcd for $C_{17}H_{12}F_5NNaO_2S^+$ [M+Na]$^+$: 412.0402, found 412.0395.

Compound 1v *N*-benzyl-*N*-(4,4,4-trifluorobuta-1,2-dien-1-yl)naphthalene-2-sulfonamide

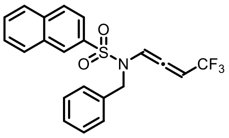

Chemical Formula: $C_{21}H_{16}F_3NO_2S$
Molecular weight: 403.42 g.mol^{-1}
White solid
Melting point: 100–102 °C

^{1}H NMR (500 MHz, CDCl$_3$) δ 8.42 (d, *J* = 2.0 Hz, 1H), 8.01–7.93 (m, 3H), 7.79 (dd, *J* = 8.5 Hz, *J* = 2.0 Hz, 1H), 7.71–7.63 (m, 2H), 7.52–7.49 (m, 1H), 7.29–7.22 (m, 5H), 5.69 (p, *J* = 5.5 Hz, 1H), 4.52 (d, *J* = 15.0 Hz, 1H), 4.23 (d, *J* = 15.0 Hz, 1H).
^{13}C NMR (126 MHz, CDCl$_3$) δ 199.0 (q, J_{C-F} = 5.9 Hz), 135.2, 135.0, 134.7, 132.3, 130.0, 129.5, 129.5, 129.0, 128.7, 128.1, 128.0, 128.0, 127.7, 122.0, 121.2 (q, J_{C-F} = 272.3 Hz), 106.9, 96.6 (q, J_{C-F} = 39.3 Hz), 51.2.
^{19}F NMR (471 MHz, CDCl$_3$) δ -62.71 (s, 3F).
IR (neat): ν = 3034, 1446, 1353, 1114, 923, 743, 587 cm^{-1}.
ESI-HRMS (ESI-TOF): m/z calcd for $C_{21}H_{16}F_3NNaO_2S^+$ [M+Na]$^+$: 426.0747, found 426.0739.

Compound 1w *N*-benzyl-*N*-(4,4,4-trifluorobuta-1,2-dien-1-yl)methanesulfonamide

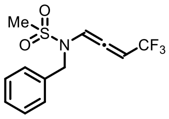

Chemical formula: $C_{12}H_{12}F_3NO_2S$
Molecular weight: 291.29 g.mol^{-1}
White solid
Melting point: 64–67 °C

^{1}H NMR (500 MHz, CDCl$_3$) δ 7.56–7.13 (m, 6H), 5.85 (p, *J* = 5.6 Hz, 1H), 4.66 (d, *J* = 15.3 Hz, 1H), 4.41 (d, *J* = 15.3 Hz, 1H), 2.93 (s, 3H).
Data match with those described in the literature.[6]

Compound 1x *N*-benzyl-*N*-(4,4,4-trifluorobuta-1,2-dien-1-yl)cyclopropanesulfonamide

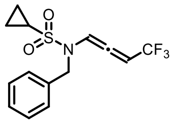

Chemical formula: $C_{14}H_{14}F_3NO_2S$
Molecular weight: 317.33 g.mol^{-1}
White solid
Melting point: 73–75 °C

^{1}H NMR (500 MHz, CDCl$_3$) δ 7.30–7.25 (m, 4H), 7.24–7.20 (m, 2H), 5.73 (p, *J* = 5.5 Hz, 1H), 4.64 (d, *J* = 15.5 Hz, 1H), 4.38 (d, *J* = 15.5 Hz, 1H), 2.35–2.30 (m, 1H), 1.22–1.18 (m, 2H), 1.00–0.96 (m, 2H).
Data match with those described in the literature.[7]

Compound 1y *N*-benzyl-*N*-(4,4,4-trifluorobuta-1,2-dien-1-yl)thiophene-2-sulfonamide

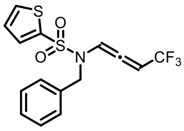

Chemical formula: $C_{15}H_{12}F_3NO_2S_2$
Molecular weight: 359.38 g.mol^{-1}
White solid
Melting point: 68–70 °C

^{1}H NMR (500 MHz, CDCl$_3$) δ 7.67 (dd, *J* = 5.0 Hz, *J* = 1.5 Hz, 1H), 7.62 (dd, *J* = 4.0 Hz, *J* = 1.5 Hz, 1H), 7.35–7.32 (m, 1H), 7.31–7.24 (m, 5H), 7.14 (dd, *J* = 5.0 Hz, *J* = 3.5 Hz, 1H), 5.72 (p, *J* = 6.0 Hz, 1H), 4.53 (d, *J* = 15.0 Hz, 1H), 4.21 (d, *J* = 15.0 Hz, 1H).
Data match with those described in the literature.[7]

Compound 4a (*E*)-diphenyl(phenyl((4,4,4-trifluoro-2-tosylbut-1-en-1-yl)amino)methyl)phosphine oxide

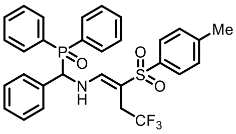

Chemical formula: $C_{30}H_{27}F_3NO_3PS$
Molecular weight: 569.58 g.mol^{-1}
White solid
Melting point: 242–244 °C
Yield: 86%

^{1}H NMR (500 MHz, DMSO) δ 8.10–8.04 (m, 3H), 7.83–7.75 (m, 3H), 7.65–7.62 (m, 1H), 7.57–7.54 (m, 2H), 7.50–7.40 (m, 5H), 7.32–7.28 (m, 4H), 7.24–7.19 (m, 3H), 6.17 (dd, *J* = 9.5 Hz, *J* = 7.0 Hz, 1H) 3.32–3.25 (m, 1H), 3.10–3.00 (m, 1H), 2.37 (s, 3H).
^{13}C NMR (126 MHz, DMSO) δ 151.5 (d, J_{C-P} = 5.8 Hz), 141.9, 140.7, 135.5, 131.9 (d, J_{C-P} = 2.5 Hz), 131.8 (d, J_{C-P} = 2.5 Hz), 131.4 (d, J_{C-P} = 94.5 Hz),131.3 (d, J_{C-P} = 94.5 Hz), 131.1 (d, J_{C-P} = 8.7 Hz), 130.9 (d, J_{C-P} = 8.9 Hz), 129.2, 128.9 (d, J_{C-P} = 4.9 Hz), 128.6 (d, J_{C-P}

= 11.3 Hz), 128.4 (d, J_{C-P} = 11.3 Hz), 127.9, 127.7, 126.0, 125.9 (q, J_{C-F} = 280.5 Hz), 94.1, 61.5 (d, J_{C-P} = 74.1 Hz), 28.4 (q, J_{C-F} = 30.9 Hz), 20.9.
^{19}F NMR (471 MHz, DMSO) δ -63.12 (s, 3F).
^{31}P NMR (202 MHz, DMSO) δ 29.16 (s, 1P).
IR (neat): ν = 3270, 1638, 1254, 1131, 1082, 700, 546 cm^{-1}.
ESI-HRMS (ESI-TOF): m/z calcd for $C_{30}H_{27}F_3NNaO_3PS^+$ $[M+Na]^+$: 592.1294, found 592.1289.

Compound 4b (*E*)-diphenyl(*p*-tolyl((4,4,4-trifluoro-2-tosylbut-1-en-1-yl)amino)methyl)phosphine oxide

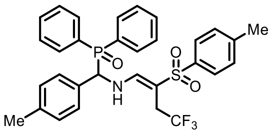

Chemical Formula: $C_{31}H_{29}F_3NO_3PS$
Molecular weight: 583.61 $g.mol^{-1}$
White solid
Melting point: 265–267 °C
Yield: 93%

^{1}H NMR (500 MHz, DMSO) δ 8.06–7.99 (m, 3H), 7.85–7.81 (m, 2H), 7.74 (d, *J* = 13.5 Hz, 1H), 7.64–7.61 (m, 1H), 7.56–7.52 (m, 2H), 7.50–7.41 (m, 3H), 7.35–7.28 (m, 6H), 7.02 (d, *J* = 8.0 Hz, 2H), 6.12 (dd, *J* = 9.5 Hz, *J* = 7.0 Hz, 1H), 3.31–3.23 (m, 1H), 3.09–3.00 (m, 1H), 2.36 (s, 3H), 2.20 (s, 3H).
^{13}C NMR (126 MHz, DMSO) δ 151.4 (d, J_{C-P} = 5.8 Hz), 142.0, 140.7, 137.0, 132.5, 131.9 (d, J_{C-P} = 2.5 Hz), 131.8 (d, J_{C-P} = 2.5 Hz), 131.6 (d, J_{C-P} = 96.3 Hz), 131.5 (d, J_{C-P} = 96.3 Hz), 131.1 (d, J_{C-P} = 8.9 Hz), 130.9 (d, J_{C-P} = 8.9 Hz), 129.3, 128.9 (d, J_{C-P} = 4.9 Hz), 128.6, 128.5 (d, J_{C-P} = 4.5 Hz), 128.4, 126.1, 125.9 (q, J_{C-F} = 280.1 Hz), 94.0, 61.1 (d, J_{C-P} = 74.6 Hz), 28.4 (q, J_{C-F} = 31.4 Hz), 21.0, 20.7.
^{19}F NMR (282 MHz, DMSO) δ -63.11 (s, 3F).
^{31}P NMR (121 MHz, DMSO) δ 29.01 (s, 1P).
IR (neat): ν = 3228, 1634, 1270, 1123, 1087, 660, 538 cm^{-1}.
ESI-HRMS (ESI-TOF): m/z calcd for $C_{31}H_{29}F_3NNaO_3PS^+$ $[M+Na]^+$: 606.1450, found 606.1437.

Compound 4c (*E*)-((4-methoxyphenyl)((4,4,4-trifluoro-2-tosylbut-1-en-1-yl)amino)methyl)diphenylphosphine oxide

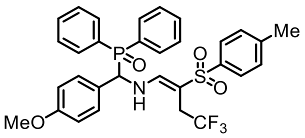

Chemical formula: $C_{31}H_{29}F_3NO_4PS$
Molecular weight: 599.61 $g.mol^{-1}$
White solid
Melting point: 233–235 °C
Yield: 68%

^{1}H NMR (500 MHz, DMSO) δ 8.08–8.02 (m, 3H), 7.86–7.82 (m, 2H), 7.77 (d, *J* = 13.5 Hz, 1H), 7.64–7.61 (m, 1H), 7.57–7.53 (m, 2H), 7.49–7.40 (m, 5H), 7.33–7.28 (m, 4H), 6.79 (d, *J* = 8.5 Hz, 2H), 6.12 (dd, *J* = 9.5 Hz, *J* = 6.5 Hz, 1H), 3.67 (s, 3H), 3.34–3.27 (m, 1H), 3.11–3.01 (m, 1H), 2.36 (s, 3H).
^{13}C NMR (126 MHz, DMSO) δ 158.7 (d, J_{C-P} = 1.6 Hz), 151.5 (d, J_{C-P} = 5.8 Hz), 141.9, 140.8, 131.8 (d, J_{C-P} = 2.5 Hz), 131.7 (d, J_{C-P} = 95.8 Hz), 131.7 (d, J_{C-P} = 2.5 Hz), 131.7 (d, J_{C-P} = 94.4 Hz), 131.0 (d, J_{C-P} = 8.9 Hz), 130.9 (d, J_{C-P} = 8.8 Hz), 130.3 (d, J_{C-P} = 5.0 Hz), 129.2, 128.5 (d, J_{C-P} = 11.2 Hz), 128.4 (d, J_{C-P} = 11.3 Hz), 127.5, 126.0, 125.9 (q, J_{C-F} = 280.4 Hz), 113.4, 93.8, 60.7 (d, J_{C-P} = 75.6 Hz), 55.0, 28.4 (q, J_{C-F} = 30.9 Hz), 20.9.
^{19}F NMR (471 MHz, DMSO) δ -63.09 (s, 3F).
^{31}P NMR (202 MHz, DMSO) δ 29.09 (s, 1P).
IR (neat): ν = 3236, 1639, 1249, 1125, 1085, 692, 545 cm^{-1}.
ESI-HRMS (ESI-TOF): m/z calcd for $C_{31}H_{29}F_3NNaO_4PS^+$ $[M+Na]^+$: 622.1399, found 622.1394.

Compound 4d (*E*)-diphenyl(((4,4,4-trifluoro-2-tosylbut-1-en-1-yl)amino)(4-(trifluoromethyl)phenyl)methyl)phosphine oxide

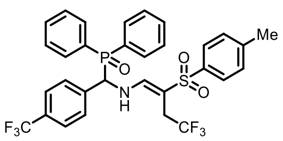

Chemical formula: $C_{31}H_{26}F_6NO_3PS$
Molecular weight: 637.58 $g.mol^{-1}$
White solid
Melting point: 237–239 °C
Yield: 91%

^{1}H NMR (500 MHz, DMSO) δ 8.22–8.17 (m, 1H), 8.11–8.07 (m, 2H), 7.86–7.82 (m, 2H), 7.79 (d, *J* = 13.5 Hz, 1H), 7.68–7.56 (m, 7H), 7.52–7.43 (m, 3H), 7.32 (dd, *J* = 22.0 Hz, *J* = 8.5 Hz, 4H), 6.37 (dd, *J* = 9.5 Hz, *J* = 7.0 Hz, 1H), 3.38–3.31 (m, 1H), 3.11–3.02 (m, 1H), 2.36 (s, 3H).
^{13}C NMR (126 MHz, DMSO) δ 151.3 (d, J_{C-P} = 5.7 Hz), 142.0, 140.5, 140.2, 132.1 (d, J_{C-P} = 2.5 Hz), 132.0 (d, J_{C-P} = 2.5 Hz), 131.1 (d, J_{C-P} = 9.1 Hz), 131.0 (d, J_{C-P} = 97.8 Hz), 131.0 (d, J_{C-P} = 94.5 Hz), 130.9 (d, J_{C-P} = 9.1 Hz), 129.6 (d, J_{C-P} = 4.5 Hz), 129.3, 128.7 (d, J_{C-P} = 11.5 Hz), 128.6 (d, J_{C-P} = 11.3 Hz), 128.3 (q, J_{C-F} = 32.3 Hz), 126.1, 125.9 (q, J_{C-F} = 280.1 Hz), 124.9 (q, J_{C-F} = 3.9 Hz), 124.1 (q, J_{C-F} = 272.7 Hz), 94.8, 61.3 (d, J_{C-P} = 72.6 Hz), 28.4 (q, J_{C-F} = 30.7 Hz), 20.9.
^{19}F NMR (471 MHz, DMSO) δ -61.05 (s, 3F), -63.13 (s, 3F).
^{31}P NMR (202 MHz, DMSO) δ 29.25 (s, 1P).
IR (neat): ν = 3256, 1643, 1328, 1115, 1070, 720, 653, 550 cm^{-1}.
ESI-HRMS (ESI-TOF): m/z calcd for $C_{31}H_{26}F_6KNO_3PS^+$ $[M+K]^+$: 676.0907, found 676.0885.

Compound 4e (*E*)-diphenyl(((4,4,4-trifluoro-2-tosylbut-1-en-1-yl)amino)(4-(trifluoromethoxy)phenyl)methyl)phosphine oxide

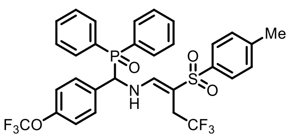

Chemical formula: $C_{31}H_{26}F_6NO_4PS$
Molecular weight: 653.58 g.mol^{-1}
White solid
Melting point: 210–212 °C
Yield: 95%

^{1}H NMR (500 MHz, DMSO) δ 8.14–8.06 (m, 3H), 7.83–7.75 (m, 3H), 7.67–7.64 (m, 1H), 7.59–7.56 (m, 4H), 7.51–7.47 (m, 1H), 7.45–7.41 (m, 2H), 7.33–7.24 (m, 6H), 6.28 (dd, *J* = 9.5 Hz, *J* = 6.5 Hz, 1H), 3.45–3.29 (m, 1H), 3.10–3.00 (m, 1H), 2.36 (s, 3H).
^{13}C NMR (126 MHz, DMSO) δ 151.3 (d, $J_{C\text{-}P}$ = 5.7 Hz), 147.9, 142.1, 140.6, 135.0, 132.1 (d, $J_{C\text{-}P}$ = 2.5 Hz), 132.0 (d, $J_{C\text{-}P}$ = 2.5 Hz), 131.1 (d, $J_{C\text{-}P}$ = 8.9 Hz), 131.1 (d, $J_{C\text{-}P}$ = 94.8 Hz), 131.0 (d, $J_{C\text{-}P}$ = 97.3 Hz), 130.9 (d, $J_{C\text{-}P}$ = 2.6 Hz), 130.8 (d, $J_{C\text{-}P}$ = 2.6 Hz), 129.3, 128.7 (d, $J_{C\text{-}P}$ = 11.5 Hz), 128.6 (d, $J_{C\text{-}P}$ = 11.3 Hz), 126.1, 125.9 (q, $J_{C\text{-}F}$ = 280.2 Hz), 120.6, 120.0 (q, $J_{C\text{-}F}$ = 256.9 Hz), 94.6, 60.9 (d, $J_{C\text{-}P}$ = 73.6 Hz), 28.4 (q, $J_{C\text{-}F}$ = 30.9 Hz), 20.9.
^{19}F NMR (282 MHz, DMSO) δ -56.81 (s, 3F), -63.12 (s, 3F).
^{31}P NMR (121 MHz, DMSO) δ 29.15 (s, 1P).
IR (neat): ν = 3250, 1640, 1249, 1171, 1126, 692, 548 cm^{-1}.
ESI-HRMS (ESI-TOF): m/z calcd for $C_{31}H_{26}F_6KNO_4PS^+$ [M+K]$^+$: 692.0856, found 692.0840.

Compound 4f (*E*)-((4-fluorophenyl)((4,4,4-trifluoro-2-tosylbut-1-en-1-yl)amino)methyl)diphenylphosphine oxide

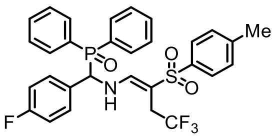

Chemical formula: $C_{30}H_{26}F_4NO_3PS$
Molecular weight: 587.57 g.mol^{-1}
White solid
Melting point: 250–252 °C
Yield: 81%

^{1}H NMR (500 MHz, DMSO) δ 8.08–8.04 (m, 3H), 7.82–7.74 (m, 3H), 7.66–7.63 (m, 1H), 7.58–7.41 (m, 7H), 7.30 (dd, *J* = 15.0 Hz, 8.5 Hz, 4H), 7.07 (t, *J* = 8.5 Hz, 2H), 6.21 (dd, *J* = 10.0 Hz, *J* = 7.0 Hz, 1H), 3.36–3.26 (m, 1H), 3.08–3.02 (m, 1H), 2.36 (s, 3H).
^{13}C NMR (126 MHz, DMSO) δ 161.7 (d, $J_{C\text{-}F}$ = 245.2 Hz), 151.4 (d, $J_{C\text{-}P}$ = 5.8Hz), 142.1, 140.6, 132.0, 131.8 (d, $J_{C\text{-}F}$ = 3.0 Hz), 131.2 (d, $J_{C\text{-}P}$ = 96.6 Hz), 131.2 (d, $J_{C\text{-}P}$ = 96.6 Hz), 131.1 (d, $J_{C\text{-}P}$ = 8.9 Hz), 131.0 (d, $J_{C\text{-}F}$ = 5.0 Hz), 130.9 (d, $J_{C\text{-}P}$ = 8.9 Hz), 129.3, 128.7 (d, $J_{C\text{-}P}$ = 11.3 Hz), 128.6 (d, $J_{C\text{-}P}$ = 11.3 Hz), 126.1, 125.9 (q, $J_{C\text{-}F}$ = 280.1 Hz), 115.0 (d, $J_{C\text{-}F}$ = 21.4 Hz), 94.4, 60.8 (d, $J_{C\text{-}P}$ = 74.3 Hz), 28.5 (q, $J_{C\text{-}F}$ = 30.6 Hz), 21.0.
^{19}F NMR (471 MHz, DMSO) δ -63.11 (s, 3F), -114.13 (s, 1F).
^{31}P NMR (121 MHz, DMSO) δ 29.18 (s, 1P).
IR (neat): ν = 3231, 1639, 1279, 1134, 1086, 659, 537 cm^{-1}.
ESI-HRMS (ESI-TOF): m/z calcd for $C_{30}H_{26}F_4KNO_3PS^+$ [M+K]$^+$: 626.0939, found 626.0932.

Compound 4g (*E*)-((4-chlorophenyl)((4,4,4-trifluoro-2-tosylbut-1-en-1-yl)amino)methyl)diphenylphosphine oxide

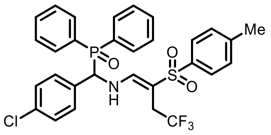

Chemical formula: $C_{30}H_{26}ClF_3NO_3PS$
Molecular weight: 604.02 g.mol^{-1}
White solid
Melting point: 257–259 °C
Yield: 73%

^{1}H NMR (500 MHz, DMSO) δ 8.13–8.05 (m, 3H), 7.85–7.81 (m, 2H), 7.76 (d, *J* = 13.0 Hz, 1H), 7.66–7.63 (m, 1H), 7.59–7.55 (m, 2H), 7.51–7.42 (m, 5H), 7.33–7.28 (m, 6H), 6.24 (dd, *J* = 9.5 Hz, *J* = 7.0 Hz, 1H), 3.38–3.29 (m, 1H), 3.11–3.01 (m, 1H), 2.36 (s, 3H).
^{13}C NMR (126 MHz, DMSO) δ 151.3 (d, $J_{C\text{-}P}$ = 5.7 Hz), 142.0, 140.6, 134.6, 132.1 (d, $J_{C\text{-}P}$ = 137.7 Hz), 132.1 (d, $J_{C\text{-}P}$ = 137.5 Hz), 132.0 (d, $J_{C\text{-}P}$ = 2.6 Hz), 131.9 (d, $J_{C\text{-}P}$ = 2.4 Hz), 131.1 (d, $J_{C\text{-}P}$ = 8.9 Hz), 130.9 (d, $J_{C\text{-}P}$ = 9.1 Hz), 130.7, 130.7 (d, $J_{C\text{-}P}$ = 8.3 Hz), 129.3, 128.7 (d, $J_{C\text{-}P}$ = 11.5 Hz), 128.5 (d, $J_{C\text{-}P}$ = 11.3 Hz), 128.0, 126.1, 125.9 (q, $J_{C\text{-}F}$ = 280.4 Hz), 94.5, 60.9 (d, $J_{C\text{-}P}$ = 73.8 Hz), 28.4 (q, $J_{C\text{-}F}$ = 30.5 Hz), 20.9.
^{19}F NMR (471 MHz, DMSO) δ -63.11 (s, 3F).
^{31}P NMR (202 MHz, DMSO) δ 29.16 (s, 1P).
IR (neat): ν = 3230, 1639, 1250, 1129, 1087, 660, 542 cm^{-1}.
ESI-HRMS (ESI-TOF): m/z calcd for $C_{30}H_{26}ClF_3KNO_3PS^+$ [M+K]$^+$: 642.0644, found 642.0623.

Compound 4h (*E*)-((4-bromophenyl)((4,4,4-trifluoro-2-tosylbut-1-en-1-yl)amino)methyl)diphenylphosphine oxide

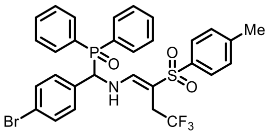

Chemical formula: $C_{30}H_{26}BrF_3NO_3PS$
Molecular weight: 648.48 g.mol^{-1}
White solid
Melting point: 251–253 °C
Yield: 78%

^{1}H NMR (500 MHz, DMSO) δ 8.10–8.03 (m, 3H), 7.84–7.80 (m, 2H), 7.74 (d, *J* = 13.0 Hz, 1H), 7.66–7.63 (m, 1H), 7.58–7.55 (m, 2H), 7.52–7.39 (m, 7H), 7.32–7.28 (m, 4H), 6.22 (dd, *J* = 9.5 Hz, *J* = 7.0 Hz, 1H), 3.44–3.27 (m, 1H), 3.09–3.00 (m, 1H), 2.36 (s, 3H).
^{13}C NMR (126 MHz, DMSO) δ 151.3 (d, $J_{C\text{-}P}$ = 5.8 Hz), 142.1, 140.6, 135.0, 132.1 (d, $J_{C\text{-}P}$ = 2.4 Hz), 132.0 (d, $J_{C\text{-}P}$ = 2.4 Hz), 131.1 (d, $J_{C\text{-}P}$ = 97.6 Hz), 131.1 (d, $J_{C\text{-}P}$ = 94.2 Hz), 131.1 (d, $J_{C\text{-}P}$ = 4.0 Hz), 131.0 (d, $J_{C\text{-}P}$ = 5.4 Hz), 130.9 (d, $J_{C\text{-}P}$ = 8.9 Hz), 129.3, 128.7, 128.6, 128.5, 126.1, 125.9 (q, $J_{C\text{-}F}$ = 280.4 Hz), 121.3 (d, $J_{C\text{-}P}$ = 2.4 Hz), 94.6, 60.9 (d, $J_{C\text{-}P}$ = 73.7 Hz), 28.4 (q, $J_{C\text{-}F}$ = 30.7 Hz), 20.9.
^{19}F NMR (282 MHz, DMSO) δ -63.11 (s, 3F).
^{31}P NMR (121 MHz, DMSO) δ 28.92 (s, 1P).
IR (neat): ν = 3266, 1641, 1251, 1124, 1076, 685, 596, 541 cm^{-1}.
ESI-HRMS (ESI-TOF): m/z calcd for $C_{30}H_{26}BrF_3KNO_3PS^+$ $[M+K]^+$: 686.0138, found 686.0120.

Compound 4i (*E*)-diphenyl(*o*-tolyl((4,4,4-trifluoro-2-tosylbut-1-en-1-yl)amino)methyl)phosphine oxide

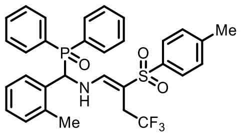

Chemical formula: $C_{31}H_{29}F_3NO_3PS$
Molecular weight: 583.61 $g.mol^{-1}$
White solid
Melting point: 252–254 °C
Yield: 76%

^{1}H NMR (500 MHz, DMSO) δ 8.14–8.10 (m, 2H), 8.01–7.99 (m, 1H), 7.93–7.88 (m, 1H), 7.84 (d, *J* = 13.5 Hz, 1H), 7.68–7.62 (m, 3H), 7.59–7.55 (m, 2H), 7.49–7.46 (m, 1H), 7.40–7.37 (m, 4H), 7.30 (d, *J* = 8.0 Hz, 2H), 7.17–7.12 (m, 2H), 7.04–7.03 (m, 1H), 5.84 (dd, *J* = 8.5 Hz, *J* = 7.0 Hz, 1H), 3.27–3.17 (m, 1H), 3.08–2.99 (m, 1H), 2.37 (s, 3H), 2.11 (s, 3H).
^{13}C NMR (126 MHz, DMSO) δ 151.3 (d, $J_{C\text{-}P}$ = 4.7 Hz), 141.9, 140.7, 136.7 (d, $J_{C\text{-}P}$ = 7.3 Hz), 133.8, 132.0 (d, $J_{C\text{-}P}$ = 2.6 Hz), 131.7 (d, $J_{C\text{-}P}$ = 93.7 Hz), 131.7 (d, $J_{C\text{-}P}$ = 8.9 Hz), 130.8 (d, $J_{C\text{-}P}$ = 9.1 Hz), 130.8 (d, $J_{C\text{-}P}$ = 97.8 Hz), 130.2, 130.2, 129.2, 128.6, 128.5 (d, $J_{C\text{-}P}$ = 1.8 Hz), 128.4, 128.0, 126.2, 125.9 (q, $J_{C\text{-}F}$ = 280.1 Hz), 125.6, 94.2, 57.4 (d, $J_{C\text{-}P}$ = 75.2 Hz), 28.4 (q, $J_{C\text{-}F}$ = 30.9 Hz), 20.9, 19.2.
^{19}F NMR (471 MHz, DMSO) δ -63.14 (s, 3F).
^{31}P NMR (202 MHz, DMSO) δ 29.75 (s, 1P).
IR (neat): ν = 3245, 1634, 1252, 1132, 1085, 663, 546 cm^{-1}.
ESI-HRMS (ESI-TOF): m/z calcd for $C_{31}H_{29}F_3KNO_3PS^+$ $[M+K]^+$: 622.1189, found 622.1165.

Compound 4j (*E*)-((2-fluorophenyl)((4,4,4-trifluoro-2-tosylbut-1-en-1-yl)amino)methyl)diphenylphosphine oxide

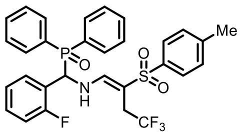

Chemical formula: $C_{30}H_{26}F_4NO_3PS$
Molecular weight: 587.57 $g.mol^{-1}$
White solid
Melting point: 219–221 °C
Yield: 87%

^{1}H NMR (500 MHz, DMSO) δ 8.15 (dd, *J* = 11.0 Hz, *J* = 7.5 Hz, 2H), 8.06–7.97 (m, 2H), 7.90 (d, *J* = 13.5 Hz, 1H), 7.79–7.75 (m, 2H), 7.62 (t, *J* = 7.5 Hz, 1H), 7.55–7.47 (m, 3H), 7.43–7.39 (m, 4H), 7.32–7.26 (m, 3H), 7.18 (t, *J* = 7.5 Hz, 1H), 7.00 (t, *J* = 9.5 Hz, 1H), 6.29 (dd, *J* = 9.5 Hz, *J* = 7.0 Hz, 1H), 3.36–3.26 (m, 1H), 3.11–3.02 (m, 1H), 2.37 (s, 3H).
^{13}C NMR (126 MHz, DMSO) δ 159.0 (d, $J_{C\text{-}F}$ = 247.2 Hz), 151.5 (d, $J_{C\text{-}P}$ = 5.5 Hz), 142.0, 140.7, 132.1 (d, $J_{C\text{-}P}$ = 2.5 Hz), 132.0 (d, $J_{C\text{-}P}$ = 2.5 Hz), 131.5 (d, $J_{C\text{-}F}$ = 3.3 Hz), 131.4 (d, $J_{C\text{-}P}$ = 8.7 Hz), 131.0 (d, $J_{C\text{-}P}$ = 98.4 Hz), 130.8 (d, $J_{C\text{-}P}$ = 9.1 Hz), 130.7 (d, $J_{C\text{-}P}$ = 98.4 Hz), 130.2 (d, $J_{C\text{-}P}$ = 8.2 Hz), 129.2, 128.6 (d, $J_{C\text{-}P}$ = 11.6 Hz), 128.4 (d, $J_{C\text{-}P}$ = 11.5 Hz), 126.2, 125.9 (q, $J_{C\text{-}F}$ = 280.2 Hz), 124.2, 122.5 (d, $J_{C\text{-}F}$ = 14.9 Hz), 115.0 (d, $J_{C\text{-}F}$ = 22.1 Hz), 95.0, 54.6 (d, $J_{C\text{-}P}$ = 75.5 Hz), 28.4 (q, $J_{C\text{-}F}$ = 30.9 Hz), 20.9.
^{19}F NMR (471 MHz, DMSO) δ -63.20 (s, 3F), -115.07 (s, 1F).
^{31}P NMR (202 MHz, DMSO) δ 29.33 (s, 1P).
IR (neat): ν = 3226, 1637, 1260, 1135, 1083, 694, 658, 549 cm^{-1}.
ESI-HRMS (ESI-TOF): m/z calcd for $C_{30}H_{26}F_4KNO_3PS^+$ $[M+K]^+$: 626.0939, found 626.0923.

Compound 4k (*E*)-((2-chlorophenyl)((4,4,4-trifluoro-2-tosylbut-1-en-1-yl)amino)methyl)diphenylphosphine oxide

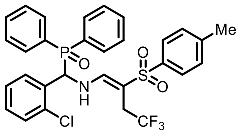

Chemical formula: $C_{30}H_{26}ClF_3NO_3PS$
Molecular weight: 604.02 $g.mol^{-1}$
White solid
Melting point: 224–226 °C
Yield: 82%

^{1}H NMR (500 MHz, DMSO) δ 8.17 (d, *J* = 8.0 Hz, 1H), 8.13–8.09 (m, 2H), 8.06–8.02 (m, 1H), 7.79 (d, *J* = 13.5 Hz, 1H), 7.69–7.65 (m, 3H), 7.60–7.56 (m, 2H), 7.50–7.47 (m, 1H), 7.41–7.25 (m, 9H), 6.03 (dd, *J* = 9.0 Hz, *J* = 7.0 Hz, 1H), 3.34–3.24 (m, 1H), 3.08–2.98 (m, 1H), 2.36 (s, 3H).
^{13}C NMR (126 MHz, DMSO) δ 151.1 (d, $J_{C\text{-}P}$ = 5.0 Hz), 142.1, 140.5, 133.1 (d, $J_{C\text{-}P}$ = 2.8 Hz), 132.9 (d, $J_{C\text{-}P}$ = 7.4 Hz), 132.2 (d, $J_{C\text{-}P}$ = 2.6 Hz), 131.8 (d, $J_{C\text{-}P}$ = 3.8 Hz), 131.6 (d, $J_{C\text{-}P}$ = 9.1 Hz), 130.9 (d, $J_{C\text{-}P}$ = 9.2 Hz), 130.9 (d, $J_{C\text{-}P}$ = 95.0 Hz), 130.3 (d, $J_{C\text{-}P}$ = 99.0 Hz), 130.0, 129.3, 129.3, 128.7 (d, $J_{C\text{-}P}$ = 11.6 Hz), 128.5 (d, $J_{C\text{-}P}$ = 11.5 Hz), 127.1, 126.3, 125.9 (q, $J_{C\text{-}F}$ = 280.4 Hz), 95.2, 57.9 (d, $J_{C\text{-}P}$ = 75.0 Hz), 28.5 (q, $J_{C\text{-}F}$ = 30.8 Hz), 21.0.
^{19}F NMR (471 MHz, DMSO) δ -63.09 (s, 3F).

^{31}**P NMR (202 MHz, DMSO)** δ 29.73 (s, 1P).
IR (neat): ν = 3236, 1637, 1251, 1122, 1090, 662, 565 cm^{-1}.
ESI-HRMS (ESI-TOF): m/z calcd for $C_{30}H_{26}ClF_3KNO_3PS^+$ [M+K]$^+$: 642.0643, found 642.0631.

Compound 4l (*E*)-((2-bromophenyl)((4,4,4-trifluoro-2-tosylbut-1-en-1-yl)amino)methyl)diphenylphosphine oxide

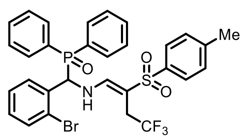

Chemical formula: $C_{30}H_{26}BrF_3NO_3PS$
Molecular weight: 648.48 g.mol^{-1}
White solid
Melting point: 225–227 °C
Yield: 82%

^{1}**H NMR (500 MHz, DMSO)** δ 8.18 (d, *J* = 7.5 Hz, 1H), 8.08–8.03 (m, 3H), 7.73–7.58 (m, 6H), 7.49 (t, *J* = 8.5 Hz, 2H), 7.41–7.37 (m, 5H), 7.30 (d, *J* = 8.0 Hz, 2H), 7.20 (t, *J* = 8.0 Hz, 1H), 5.88–5.85 (m, 1H), 3.33–3.24 (m, 1H), 3.07–2.97 (m, 1H), 2.36 (s, 3H).
^{13}**C NMR (126 MHz, DMSO)** δ 150.8 (d, $J_{C\text{-}P}$ = 4.8 Hz), 142.1, 140.4, 134.7 (d, $J_{C\text{-}P}$ = 3.0 Hz), 132.7, 132.3 (d, $J_{C\text{-}P}$ = 2.6 Hz), 132.3 (d, $J_{C\text{-}P}$ = 2.5 Hz), 131.8 (d, $J_{C\text{-}P}$ = 3.6 Hz), 131.5 (d, $J_{C\text{-}P}$ = 8.9 Hz), 130.9 (d, $J_{C\text{-}P}$ = 9.1 Hz), 130.7 (d, $J_{C\text{-}P}$ = 95.4 Hz), 130.4, 130.1 (d, $J_{C\text{-}P}$ = 99.0 Hz), 129.3, 128.8 (d, $J_{C\text{-}P}$ = 11.6 Hz), 128.6 (d, $J_{C\text{-}P}$ = 15.2 Hz), 127.8, 126.3, 125.9 (q, $J_{C\text{-}F}$ = 280.2 Hz), 124.1 (d, $J_{C\text{-}P}$ = 8.2 Hz), 95.4, 60.5 (d, $J_{C\text{-}P}$ = 75.0 Hz), 28.4 (q, $J_{C\text{-}F}$ = 30.9 Hz), 20.9.
^{19}**F NMR (471 MHz, DMSO)** δ -63.04 (s, 3F).
^{31}**P NMR (202 MHz, DMSO)** δ 29.76 (s, 1P).
IR (neat): ν = 3242, 1639, 1252, 1134, 1090, 690, 545 cm^{-1}.
ESI-HRMS (ESI-TOF): m/z calcd for $C_{30}H_{26}BrF_3KNO_3PS^+$ [M+K]$^+$: 686.0138, found 686.0122.

Compound 4m (*E*)-(benzo[*d*][1,3]dioxol-5-yl((4,4,4-trifluoro-2-tosylbut-1-en-1-yl)amino)methyl)diphenylphosphine oxide

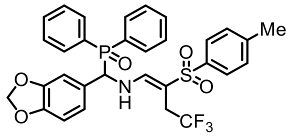

Chemical formula: $C_{31}H_{27}F_3NO_5PS$
Molecular weight: 613.59 g.mol^{-1}
White solid
Melting point: 242–244 °C
Yield: 53%

^{1}**H NMR (500 MHz, DMSO)** δ 8.05–8.01 (m, 2H), 7.99–7.94 (m, 1H), 7.85–7.81 (m, 2H), 7.72 (d, *J* = 13.0 Hz, 1H), 7.65–7.61 (m, 1H), 7.57–7.53 (m, 2H), 7.50–7.42 (m, 3H), 7.32–7.28 (m, 4H), 7.19–7.18 (m, 1H), 6.88–6.86 (m, 1H), 6.74 (d, *J* = 8.0 Hz, 1H), 6.08 (dd, *J* = 9.5 Hz, *J* = 6.5 Hz, 1H), 5.95 (dd, *J* = 3.5 Hz, *J* = 1.0 Hz, 2H), 3.35–3.25 (m, 1H), 3.08–2.99 (m, 1H), 2.36 (s, 3H).
^{13}**C NMR (126 MHz, DMSO)** δ 151.4 (d, $J_{C\text{-}P}$ = 5.9 Hz), 147.0 (d, $J_{C\text{-}P}$ = 1.1 Hz), 146.8 (d, $J_{C\text{-}P}$ = 1.8 Hz), 142.0, 140.7, 131.9 (d, $J_{C\text{-}P}$ = 1.0 Hz), 131.9 (d, $J_{C\text{-}P}$ = 1.0 Hz), 131.5 (d, $J_{C\text{-}P}$ = 95.0 Hz), 131.4 (d, $J_{C\text{-}P}$ = 94.8 Hz), 131.1 (d, $J_{C\text{-}P}$ = 8.9 Hz), 130.9 (d, $J_{C\text{-}P}$ = 8.8 Hz), 129.3, 129.2, 128.7 (d, $J_{C\text{-}P}$ = 11.5 Hz), 128.5 (d, $J_{C\text{-}P}$ = 11.3 Hz), 126.1, 126.0 (q, $J_{C\text{-}F}$ = 280.4 Hz), 122.7 (d, $J_{C\text{-}P}$ = 6.0 Hz), 109.4 (d, $J_{C\text{-}P}$ = 4.3 Hz), 107.8, 101.1, 94.1, 61.1 (d, $J_{C\text{-}P}$ = 75.5 Hz), 28.5 (q, $J_{C\text{-}F}$ = 31.0 Hz), 21.0.
^{19}**F NMR (282 MHz, DMSO)** δ -63.09 (s, 3F).
^{31}**P NMR (121 MHz, DMSO)** δ 29.18 (s, 1P).
IR (neat): ν = 3226, 1635, 1274, 1250, 1181, 1123, 1083, 694, 583, 545, 520 cm^{-1}.
ESI-HRMS (ESI-TOF): m/z calcd for $C_{31}H_{27}F_3KNO_5PS^+$ [M+K]$^+$: 652.0931, found 652.0923.

Compound 4n (*E*)-((3,5-difluorophenyl)((4,4,4-trifluoro-2-tosylbut-1-en-1-yl)amino)methyl)diphenylphosphine oxide

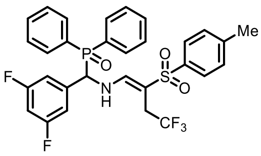

Chemical formula: $C_{30}H_{25}F_5NO_3PS$
Molecular weight: 605.56 g.mol^{-1}
White solid
Melting point: 227–229 °C
Yield: 75%

^{1}**H NMR (500 MHz, DMSO)** δ 8.11–8.03 (m, 3H), 7.83–7.79 (m, 2H), 7.71–7.66 (m, 2H), 7.61–7.57 (m, 2H), 7.55–7.51 (m, 1H), 7.49–7.46 (m, 2H), 7.32–7.28 (m, 4H), 7.14–7.07 (m, 3H), 6.27 (dd, *J* = 9.5 Hz, *J* = 7.0 Hz, 1H), 3.39–3.31 (m, 1H), 3.09–3.00 (m, 1H), 2.36 (s, 3H).
^{13}**C NMR (126 MHz, DMSO)** δ 161.8 (d, $J_{C\text{-}F}$ = 246.8 Hz), 151.1 (d, $J_{C\text{-}P}$ = 5.5 Hz), 142.2, 140.4, 139.7 (t, $J_{C\text{-}F}$ = 9.3 Hz), 132.3 (d, $J_{C\text{-}P}$ = 2.6 Hz), 132.2 (d, $J_{C\text{-}P}$ = 2.6 Hz), 131.1 (d, $J_{C\text{-}P}$ = 9.1 Hz), 130.9 (d, $J_{C\text{-}P}$ = 9.3 Hz), 130.6 (d, $J_{C\text{-}P}$ = 95.0 Hz), 130.5 (d, $J_{C\text{-}P}$ = 95.0 Hz), 129.4, 128.8 (d, $J_{C\text{-}P}$ = 11.6 Hz), 128.7 (d, $J_{C\text{-}P}$ = 11.5 Hz), 126.1, 125.9 (q, $J_{C\text{-}F}$ = 280.2 Hz), 112.1 (d, $J_{C\text{-}F}$ = 26.1 Hz), 103.4 (t, $J_{C\text{-}F}$ = 25.8 Hz), 95.2, 61.2 (d, $J_{C\text{-}P}$ = 72.6 Hz), 28.5 (q, $J_{C\text{-}F}$ = 30.9 Hz), 21.0.
^{19}**F NMR (471 MHz, DMSO)** δ -63.10 (s, 3F), -109.81 (s, 2F).
^{31}**P NMR (202 MHz, DMSO)** δ 29.30 (s, 1P).
IR (neat): ν = 3245, 1642, 1275, 1119, 1089, 695, 660, 582, 552 cm^{-1}.
ESI-HRMS (ESI-TOF): m/z calcd for $C_{30}H_{25}F_5KNO_3PS^+$ [M+K]$^+$: 644.0845, found 644.0838.

Compound 4o (*E*)-((3,4-dichlorophenyl)((4,4,4-trifluoro-2-tosylbut-1-en-1-yl)amino)methyl)diphenylphosphine oxide

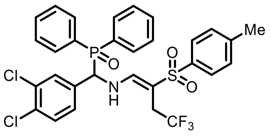

Chemical formula: $C_{30}H_{25}Cl_2F_3NO_3PS$
Molecular weight: 638.46 g.mol^{-1}
White solid
Melting point: 245–247 °C
Yield: 76%

^{1}H NMR (500 MHz, DMSO) δ 8.13–8.02 (m, 3H), 7.83–7.77 (m, 3H), 7.72–7.65 (m, 2H), 7.60–7.45 (m, 6H), 7.32–7.28 (m, 5H), 6.27 (dd, *J* = 9.5 Hz, *J* = 7.0 Hz, 1H), 3.44–3.30 (m, 1H), 3.07–2.98 (m, 1H), 2.36 (s, 3H).
^{13}C NMR (126 MHz, DMSO) δ 151.1 (d, $J_{C\text{-}P}$ = 5.7 Hz), 142.1, 140.5, 136.5, 132.3 (d, $J_{C\text{-}P}$ = 2.5 Hz), 132.2 (d, $J_{C\text{-}P}$ = 2.5 Hz), 131.1 (d, $J_{C\text{-}P}$ = 9.1 Hz), 130.9 (d, $J_{C\text{-}P}$ = 9.1 Hz), 130.8 (d, $J_{C\text{-}P}$ = 1.9 Hz), 130.8, 130.7 (d, $J_{C\text{-}P}$ = 95.0 Hz), 130.7 (d, $J_{C\text{-}P}$ = 97.8 Hz), 130.6 (d, $J_{C\text{-}P}$ = 2.3 Hz), 130.2, 129.3, 129.0 (d, $J_{C\text{-}P}$ = 4.6 Hz), 128.7 (d, $J_{C\text{-}P}$ = 22.4 Hz), 128.7, 126.1, 125.9 (q, $J_{C\text{-}F}$ = 280.2 Hz), 95.0, 60.7 (d, $J_{C\text{-}P}$ = 73.08 Hz), 28.5 (q, $J_{C\text{-}F}$ = 29.4 Hz), 21.0.
^{19}F NMR (471 MHz, DMSO) δ -63.11 (s, 3F).
^{31}P NMR (202 MHz, DMSO) δ 29.32 (s, 1P).
IR (neat): ν = 3222, 1637, 1279, 1131, 1088, 693, 661, 561 cm^{-1}.
ESI-HRMS (ESI-TOF): m/z calcd for $C_{30}H_{25}Cl_2F_3KNO_3PS^+$ $[M+K]^+$: 676.0253, found 676.0246.

Compound 4p (*E*)-*N*-((3,4-dichlorophenyl)(methoxy)methyl)-4,4,4-trifluoro-2-tosylbut-1-en-1-amine

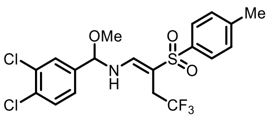

Chemical formula: $C_{19}H_{18}Cl_2F_3NO_3S$
Molecular weight: 468.31 g.mol^{-1}
White solid
R_f = 0.18 (petroleum ether : ethyl acetate = 5:1)
Yield: 95%

^{1}H NMR (500 MHz, DMSO) δ 8.00–7.96 (m, 1H), 7.80 (d, *J* = 13.5 Hz, 1H), 7.69 (d, *J* = 8.0 Hz, 1H), 7.64–7.62 (m, 3H), 7.39 (dd, *J* = 8.5 Hz, *J* = 2.0 Hz, 1H), 7.35 (d, *J* = 8.0 Hz, 2H), 5.68 (d, *J* = 8.0 Hz, 1H), 3.35 (s, 3H), 3.25 (q, *J* = 10.5 Hz, 2H), 2.36 (s, 3H).
^{13}C NMR (126 MHz, DMSO) δ 149.4, 142.4, 140.4, 140.3, 131.0, 130.9, 130.6, 129.5, 128.3, 126.8, 126.4, 126.2 (q, $J_{C\text{-}F}$ = 280.1 Hz), 96.3 (q, $J_{C\text{-}F}$ = 2.8 Hz), 88.5, 54.5, 28.9 (q, $J_{C\text{-}F}$ = 30.9 Hz), 20.9.
^{19}F NMR (471 MHz, DMSO) δ -63.10 (s, 3F).
ESI-HRMS (ESI-TOF): m/z calcd for $C_{19}H_{18}Cl_2F_3NNaO_3S^+$ $[M+Na]^+$: 490.0229, found 490.0224.

Compound 4q (*E*)-(naphthalen-1-yl((4,4,4-trifluoro-2-tosylbut-1-en-1-yl)amino)methyl)diphenylphosphine oxide

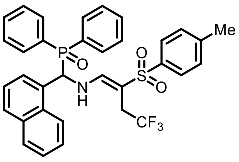

Chemical formula: $C_{34}H_{29}F_3NO_3PS$
Molecular weight: 619.64 g.mol^{-1}
White solid
Melting point: 248–250 °C
Yield: 39%

^{1}H NMR (500 MHz, DMSO) δ 8.44–8.42 (m, 1H), 8.33 (d, *J* = 7.5 Hz, 1H), 8.21–8.17 (m, 2H), 8.06–8.01 (m, 1H), 7.93 (d, *J* = 13.5 Hz, 1H), 7.87–7.83 (m, 2H), 7.71–7.65 (m, 3H), 7.60–7.56 (m, 2H), 7.49–7.45 (m, 3H), 7.35–7.22 (m, 7H), 6.60 (dd, *J* = 9.0 Hz, *J* = 6.5 Hz, 1H), 3.24–3.14 (m, 1H), 3.03–2.93 (m, 1H), 2.36 (s, 3H).
^{13}C NMR (126 MHz, DMSO) δ 151.1 (d, $J_{C\text{-}P}$ = 4.4 Hz), 142.0, 140.7, 133.3, 132.1, 131.9, 131.7 (d, $J_{C\text{-}P}$ = 9.1 Hz), 131.6, 131.5 (d, $J_{C\text{-}P}$ = 93.7 Hz), 131.2 (d, $J_{C\text{-}P}$ = 98.4 Hz), 130.9 (d, $J_{C\text{-}P}$ = 7.1 Hz), 130.7 (d, $J_{C\text{-}P}$ = 8.9 Hz), 129.3, 128.9, 128.7 (d, $J_{C\text{-}P}$ = 4.8 Hz), 128.6, 128.6 (d, $J_{C\text{-}P}$ = 11.5 Hz), 128.4 (d, $J_{C\text{-}P}$ = 11.3 Hz), 126.2, 126.2, 125.9 (q, $J_{C\text{-}F}$ = 280.6 Hz), 125.8, 125.1, 123.9, 94.3, 56.2 (d, $J_{C\text{-}P}$ = 74.8 Hz), 28.5 (q, $J_{C\text{-}F}$ = 30.7 Hz), 21.0.
^{19}F NMR (471 MHz, DMSO) δ -63.13 (s, 3F).
^{31}P NMR (202 MHz, DMSO) δ 30.14 (s, 1P).
IR (neat): ν = 3219, 1637, 1261, 1129, 1088, 695, 665, 546 cm^{-1}.
ESI-HRMS (ESI-TOF): m/z calcd for $C_{34}H_{29}F_3KNO_3PS^+$ $[M+K]^+$: 658.1189, found 658.1174.

Compound 4r (*E*)-(((4,4-difluoro-2-tosylbut-1-en-1-yl)amino)(phenyl)methyl)diphenylphosphine oxide

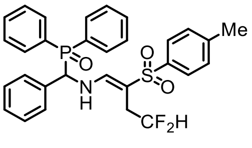

Chemical formula: $C_{30}H_{28}F_2NO_3PS$
Molecular weight: 551.59 g.mol^{-1}
White solid
Melting point: 193–195 °C
Yield: 84%

^{1}H NMR (500 MHz, DMSO) δ 8.07–8.02 (m, 2H), 7.88–7.79 (m, 3H), 7.64–7.60 (m, 2H), 7.57–7.53 (m, 2H), 7.49–7.40 (m, 5H), 7.33–7.29 (m, 4H), 7.23–7.17 (m, 3H), 6.10 (dd, *J* = 9.5 Hz, *J* = 6.5 Hz, 1H), 5.59–5.34 (m, 1H), 2.72–2.56 (m, 2H), 2.36 (s, 3H).

^{13}**C NMR (126 MHz, DMSO)** δ 149.5 (d, J_{C-P} = 5.3 Hz), 142.4, 139.9, 135.6, 132.0, 131.6 (d, J_{C-P} = 94.2 Hz), 131.3 (d, J_{C-P} = 94.2 Hz), 131.1 (d, J_{C-P} = 8.6 Hz), 131.0 (d, J_{C-P} = 8.9 Hz), 129.5, 129.0 (d, J_{C-P} = 4.9 Hz), 128.7 (d, J_{C-P} = 11.3 Hz), 128.5 (d, J_{C-P} = 11.3 Hz), 128.1, 127.8, 126.4, 115.7 (t, J_{C-F} = 241.7 Hz), 96.4, 61.3 (d, J_{C-P} = 73.7 Hz), 29.0 (t, J_{C-F} = 24.2 Hz), 21.0.
^{19}**F NMR (471 MHz, DMSO)** δ -113.80 (d, *J* = 274.1 Hz, 1F), -114.19 (d, *J* = 274.1 Hz, 1F).
^{31}**P NMR (202 MHz, DMSO)** δ 29.62 (s, 1P).
IR (neat): ν = 3197, 1639, 1268, 1105, 722, 694, 543 cm^{-1}.
ESI-HRMS (ESI-TOF): m/z calcd for $C_{30}H_{28}F_2KNO_3PS^+$ $[M+K]^+$: 590.1127, found 590.1104.

Compound 4t (*E*)-diphenyl(phenyl((4,4,4-trifluoro-2-((2-(trifluoromethyl)phenyl)sulfonyl)but-1-en-1-yl)amino)methyl)phosphine oxide

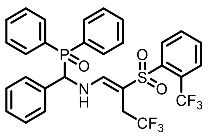

Chemical formula: $C_{30}H_{24}F_6NO_3PS$
Molecular weight: 623.55 $g.mol^{-1}$
White solid
Melting point: 236–238 °C
Yield: 55%

^{1}**H NMR (500 MHz, DMSO)** δ 8.32–8.27 (m, 1H), 8.07–8.03 (m, 2H), 7.88–7.86 (m, 1H), 7.83–7.71 (m, 6H), 7.62–7.59 (m, 1H), 7.55–7.51 (m, 2H), 7.49–7.45 (m, 3H), 7.43–7.39 (m, 2H), 7.23–7.18 (m, 3H), 6.21 (dd, *J* = 9.5 Hz, *J* = 7.5 Hz, 1H), 3.53–3.42 (m, 1H), 3.13–3.03 (m, 1H).
^{13}**C NMR (126 MHz, DMSO)** δ 152.8 (d, J_{C-P} = 6.9 Hz), 141.9, 135.6, 132.9, 132.6, 131.9 (d, J_{C-P} = 2.5 Hz), 131.9 (d, J_{C-P} = 2.5 Hz), 131.4 (d, J_{C-P} = 95.6 Hz), 131.3 (d, J_{C-P} = 95.6 Hz), 131.1 (d, J_{C-P} = 6.5 Hz), 131.0 (d, J_{C-P} = 6.5 Hz), 130.9, 129.0 (d, J_{C-P} = 4.8 Hz), 128.6 (d, J_{C-P} = 11.5 Hz), 128.5 (d, J_{C-P} = 11.5 Hz), 128.0 (q, J_{C-F} = 6.7 Hz), 128.0, 127.8, 126.0 (q, J_{C-F} = 280.4 Hz), 125.8 (q, J_{C-F} = 32.8 Hz), 122.9 (q, J_{C-F} = 274.7 Hz), 93.5, 61.6 (d, J_{C-P} = 74.2 Hz), 28.5 (q, J_{C-F} = 30.6 Hz).
^{19}**F NMR (282 MHz, DMSO)** δ -55.74 (s, 3F), -63.30 (s, 3F).
^{31}**P NMR (121 MHz, DMSO)** δ 29.14 (s, 1P).
IR (neat): ν = 3065, 1634, 1308, 1127, 1035, 695, 603, 542 cm^{-1}.
ESI-HRMS (ESI-TOF): m/z calcd for $C_{30}H_{24}F_6KNO_3PS^+$ $[M+K]^+$: 662.0750, found 662.0747.

Compound 4u (*E*)-(((2-((2,6-difluorophenyl)sulfonyl)-4,4,4-trifluorobut-1-en-1-yl)amino)(phenyl)methyl)diphenylphosphine oxide

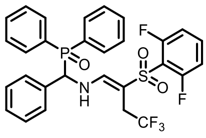

Chemical formula: $C_{29}H_{23}F_5NO_3PS$
Molecular weight: 591.53 $g.mol^{-1}$
White solid
Melting point: 257–259 °C
Yield: 85%

^{1}**H NMR (500 MHz, DMSO)** δ 8.40–8.35 (m, 1H), 8.03–7.99 (m, 2H), 7.84–7.80 (m, 3H), 7.63–7.58 (m, 1H), 7.55–7.45 (m, 6H), 7.43–7.39 (m, 2H), 7.23–7.14 (m, 5H), 6.25 (dd, *J* = 9.5 Hz, *J* = 7.0 Hz, 1H), 3.59–3.50 (m, 1H), 3.26–3.16 (m, 1H).
^{13}**C NMR (126 MHz, DMSO)** δ 158.7 (d, J_{C-F} = 256.8 Hz), 153.1 (d, J_{C-P} = 6.4 Hz), 135.5, 134.7 (t, J_{C-F} = 11.2 Hz), 131.8 (d, J_{C-P} = 2.5 Hz), 131.7 (d, J_{C-P} = 2.5 Hz), 131.3 (d, J_{C-P} = 96.9 Hz), 131.2 (d, J_{C-P} = 96.9 Hz), 131.0, 130.9, 128.9 (d, J_{C-P} = 4.8 Hz), 128.5 (d, J_{C-P} = 11.5 Hz), 128.4 (d, J_{C-P} = 11.5 Hz), 127.9, 127.7, 126.1 (q, J_{C-F} = 280.1 Hz), 120.6 (t, J_{C-F} = 15.5 Hz), 113.1 (d, J_{C-F} = 23.7 Hz), 95.4, 61.7 (d, J_{C-P} = 74.0 Hz), 28.3 (q, J_{C-F} = 30.9 Hz).
^{19}**F NMR (282 MHz, DMSO)** δ -63.90 (s, 3F), -108.33 (s, 2F).
^{31}**P NMR (121 MHz, DMSO)** δ 29.02 (s, 1P).
IR (neat): ν = 3216, 1639, 1464, 1184, 1125, 1098, 725 692, 546 cm^{-1}.
ESI-HRMS (ESI-TOF): m/z calcd for $C_{29}H_{23}F_5KNO_3PS^+$ $[M+K]^+$: 630.0688, found 630.0665.

Compound 4v (*E*)-diphenyl(phenyl((4,4,4-trifluoro-2-(naphthalen-2-ylsulfonyl)but-1-en-1-yl)amino)methyl)phosphine oxide

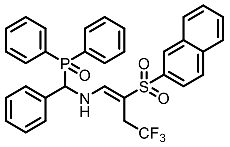

Chemical formula: $C_{33}H_{27}F_3NO_3PS$
Molecular weight: 605.61 $g.mol^{-1}$
White solid
Melting point: 233–235 °C
Yield: 69%

^{1}**H NMR (500 MHz, DMSO)** δ 8.34 (s, 1H), 8.27–8.22 (m, 1H), 8.15–8.02 (m, 5H), 7.97 (d, *J* = 13.0 Hz, 1H), 7.88–7.84 (m, 2H), 7.68–7.36 (m, 11H), 7.25–7.17 (m, 3H), 6.26 (dd, *J* = 9.5 Hz, *J* = 7.0 Hz, 1H), 3.47–3.37 (m, 1H), 3.24–3.14 (m, 1H).
^{13}**C NMR (126 MHz, DMSO)** δ 152.0 (d, J_{C-P} = 5.9 Hz), 140.4, 135.5, 133.9, 132.0 (d, J_{C-P} = 2.5 Hz), 131.9 (d, J_{C-P} = 2.5 Hz), 131.7, 131.4 (d, J_{C-P} = 96.6 Hz), 131.4 (d, J_{C-P} = 96.6 Hz), 131.2 (d, J_{C-P} = 8.9 Hz), 131.0 (d, J_{C-P} = 8.9 Hz), 129.2, 129.0 (d, J_{C-P} = 4.8 Hz), 128.7, 128.6, 128.6, 128.5, 128.5, 128.1, 127.8, 127.5, 126.7, 126.1 (q, J_{C-F} = 280.4 Hz), 122.3, 94.1, 61.6 (d, J_{C-P} = 74.0 Hz), 28.5 (q, J_{C-F} = 30.7 Hz).
^{19}**F NMR (471 MHz, DMSO)** δ -63.15 (s, 3F).
^{31}**P NMR (202 MHz, DMSO)** δ 29.48 (s, 1P).
IR (neat): ν = 3300, 1639, 1287, 1143, 1115, 694, 656, 546 cm^{-1}.
ESI-HRMS (ESI-TOF): m/z calcd for $C_{33}H_{27}F_3KNO_3PS^+$ $[M+K]^+$: 644.1033, found 644.1021.

Compound 4w (*E*)-diphenyl(phenyl((4,4,4-trifluoro-2-(methylsulfonyl)but-1-en-1-yl)amino)methyl)phosphine oxide

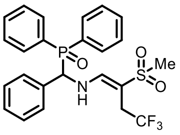

Chemical formula: $C_{24}H_{23}F_3NO_3PS$
Molecular weight: 493.48 g.mol^{-1}
White solid
Melting point: 205–207 °C
Yield: 70%

^{1}H NMR (500 MHz, DMSO) δ 8.05–8.01 (m, 2H), 7.89–7.79 (m, 3H), 7.59–7.52 (m, 3H), 7.49–7.40 (m, 6H), 7.22–7.16 (m, 3H), 6.06 (dd, *J* = 9.5 Hz, *J* = 6.5 Hz, 1H), 3.51–3.44 (m, 1H), 3.24–3.14 (m, 1H), 2.62 (s, 3H).
^{13}C NMR (126 MHz, DMSO) δ 150.8 (d, $J_{C\text{-}P}$ = 5.2 Hz), 135.4, 131.9 (d, $J_{C\text{-}P}$ = 2.6 Hz), 131.9 (d, $J_{C\text{-}P}$ = 2.6 Hz), 131.5 (d, $J_{C\text{-}P}$ = 96.4 Hz), 131.2 (d, $J_{C\text{-}P}$ = 94.0 Hz), 131.2 (d, $J_{C\text{-}P}$ = 9.1 Hz), 131.0 (d, $J_{C\text{-}P}$ = 8.9 Hz), 129.0 (d, $J_{C\text{-}P}$ = 4.9 Hz), 128.6 (d, $J_{C\text{-}P}$ = 8.1 Hz), 128.5 (d, $J_{C\text{-}P}$ = 8.1 Hz), 128.0 (d, $J_{C\text{-}P}$ = 1.3 Hz), 127.8 (d, $J_{C\text{-}P}$ = 1.9 Hz), 126.4 (q, $J_{C\text{-}F}$ = 280.0 Hz), 95.2, 61.7 (d, $J_{C\text{-}P}$ = 73.5 Hz), 44.4, 28.5 (q, $J_{C\text{-}F}$ = 30.4 Hz).
^{19}F NMR (471 MHz, DMSO) δ -63.21 (s, 3F).
^{31}P NMR (202 MHz, DMSO) δ 29.52 (s, 1P).
IR (neat): ν = 3224, 1637, 1248, 1115, 1025, 693, 543 cm^{-1}.
ESI-HRMS (ESI-TOF): m/z calcd for $C_{24}H_{23}F_3KNO_3PS^+$ [M+K]$^+$: 532.0720, found 532.0711.

Compound 4x (*E*)-(((2-(cyclopropylsulfonyl)-4,4,4-trifluorobut-1-en-1-yl)amino)(phenyl)methyl)diphenylphosphine oxide

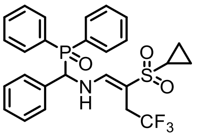

Chemical formula: $C_{26}H_{25}F_3NO_3PS$
Molecular weight: 519.52 g.mol^{-1}
White solid
Melting point: 161–163 °C
Yield: 76%

^{1}H NMR (500 MHz, DMSO) δ 8.04–8.00 (m, 2H), 7.90–7.85 (m, 1H), 7.81–7.77 (m, 2H), 7.58–7.52 (m, 3H), 7.49–7.45 (m, 1H), 7.43–7.37 (m, 5H), 7.22–7.17 (m, 3H), 6.05 (dd, *J* = 9.5 Hz, *J* = 7.5 Hz, 1H), 3.53–3.43 (m, 1H), 3.25–3.14 (m, 1H), 2.28–2.23 (m, 1H), 0.89–0.79 (m, 2H), 0.78–0.68 (m, 2H).
^{13}C NMR (126 MHz, DMSO) δ 150.7 (d, $J_{C\text{-}P}$ = 6.0 Hz), 135.6, 131.8 (d, $J_{C\text{-}P}$ = 2.6 Hz), 131.7 (d, $J_{C\text{-}P}$ = 2.6 Hz), 131.5 (d, $J_{C\text{-}P}$ = 96.5 Hz), 131.4 (d, $J_{C\text{-}P}$ = 93.9 Hz), 131.1 (d, $J_{C\text{-}P}$ = 9.1 Hz), 130.9 (d, $J_{C\text{-}P}$ = 8.9 Hz), 128.9 (d, $J_{C\text{-}P}$ = 4.9 Hz), 128.5 (d, $J_{C\text{-}P}$ = 11.5 Hz), 128.4 (d, $J_{C\text{-}P}$ = 11.3 Hz), 127.9, 127.6 (d, $J_{C\text{-}P}$ = 1.8 Hz), 126.3 (q, $J_{C\text{-}F}$ = 279.7 Hz), 94.7, 61.4 (d, $J_{C\text{-}P}$ = 74.1 Hz), 32.7, 28.7 (q, $J_{C\text{-}F}$ = 31.2 Hz), 5.2, 5.1.
^{19}F NMR (471 MHz, DMSO) δ -63.34 (s, 3F).
^{31}P NMR (202 MHz, DMSO) δ 29.38 (s, 1P).
IR (neat): ν = 3228, 2927, 1637, 1253, 1116, 692, 540 cm^{-1}.
ESI-HRMS (ESI-TOF): m/z calcd for $C_{26}H_{25}F_3KNO_3PS^+$ [M+K]$^+$: 558.0876, found 558.0855.

Compound 4y (*E*)-diphenyl(phenyl((4,4,4-trifluoro-2-(thiophen-2-ylsulfonyl)but-1-en-1-yl)amino)methyl)phosphine oxide

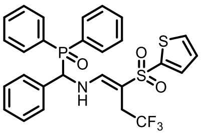

Chemical formula: $C_{27}H_{23}F_3NO_3PS_2$
Molecular weight: 561.57 g.mol^{-1}
White solid
Melting point: 242–244 °C
Yield: 87%

^{1}H NMR (500 MHz, DMSO) δ 8.20–8.15 (m, 1H), 8.04–8.00 (m, 2H), 7.84–7.76 (m, 4H), 7.60–7.40 (m, 8H), 7.23–7.17 (m, 4H), 7.09–7.07 (m, 1H), 6.17 (dd, *J* = 9.5 Hz, *J* = 7.0 Hz, 1H), 3.38–3.31 (m, 1H), 3.19–3.09 (m, 1H).
^{13}C NMR (126 MHz, DMSO) δ 151.6 (d, $J_{C\text{-}P}$ = 6.2 Hz), 145.8, 135.5, 132.2, 131.9, 131.4 (d, $J_{C\text{-}P}$ = 95.0 Hz), 131.3 (d, $J_{C\text{-}P}$ = 95.0 Hz), 131.1 (d, $J_{C\text{-}P}$ = 8.9 Hz), 130.9 (d, $J_{C\text{-}P}$ = 8.9 Hz), 130.9, 128.9 (d, $J_{C\text{-}P}$ = 4.8 Hz), 128.7 (d, $J_{C\text{-}P}$ = 11.5 Hz), 128.5 (d, $J_{C\text{-}P}$ = 11.3 Hz), 128.0, 127.8, 127.5, 126.0 (q, $J_{C\text{-}F}$ = 280.5 Hz), 95.3, 61.5 (d, $J_{C\text{-}P}$ = 73.7 Hz), 28.5 (q, $J_{C\text{-}F}$ = 30.6 Hz).
^{19}F NMR (471 MHz, DMSO) δ -63.31 (s, 3F).
^{31}P NMR (202 MHz, DMSO) δ 29.30 (s, 1P).
IR (neat): ν = 3307, 3063, 1639, 1117, 684, 606, 539 cm^{-1}.
ESI-HRMS (ESI-TOF): m/z calcd for $C_{27}H_{24}F_3NO_3PS_2^+$ [M+H]$^+$: 562.0882, found 562.0875.

Compound 4z (*E*)-(phenyl((4,4,4-trifluoro-2-tosylbut-1-en-1-yl)amino)methyl)di-*p*-tolylphosphine oxide

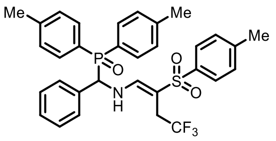

Chemical formula: $C_{32}H_{31}F_3NO_3PS$
Molecular weight: 597.63 g.mol^{-1}
White solid
Melting point: 253–255 °C
Yield: 79%

^{1}H NMR (300 MHz, DMSO) δ 8.07–7.99 (m, 1H), 7.93–7.87 (m, 2H), 7.77 (d, *J* = 13.2 Hz, 1H), 7.67–7.61 (m, 2H), 7.45–7.41 (m, 2H), 7.37–7.32 (m, 4H), 7.29–7.20 (m, 7H), 6.09 (dd, *J* = 9.3 Hz, *J* = 6.9 Hz, 1H), 3.27–3.15 (m, 1H), 3.12–2.99 (m, 1H), 2.37 (s, 3H), 2.35 (s, 3H), 2.28 (s, 3H).
^{13}C NMR (126 MHz, DMSO) δ 151.5 (d, $J_{C\text{-}P}$ = 5.7 Hz), 141.8, 141.7 (d, $J_{C\text{-}P}$ = 2.6 Hz), 141.5 (d, $J_{C\text{-}P}$ = 2.6 Hz), 140.8, 135.7, 131.0 (d, $J_{C\text{-}P}$ = 9.2 Hz), 130.8 (d, $J_{C\text{-}P}$ = 9.3 Hz), 129.2, 129.1 (d, $J_{C\text{-}P}$ = 11.7 Hz), 128.9 (d, $J_{C\text{-}P}$ = 12.2 Hz), 128.5 (d, $J_{C\text{-}P}$ = 100.0 Hz) x 2, 127.9, 127.6, 126.1, 125.9 (q, $J_{C\text{-}F}$ = 280.2 Hz), 94.1, 61.5 (d, $J_{C\text{-}P}$ = 74.0 Hz), 28.3 (q, $J_{C\text{-}F}$ = 30.4 Hz), 21.1, 21.0, 20.9.
^{19}F NMR (471 MHz, DMSO) δ -63.12 (s, 3F).
^{31}P NMR (202 MHz, DMSO) δ 29.77 (s, 1P).
IR (neat): ν = 3224, 2970, 1638, 1247, 1133, 1024, 660, 537 cm^{-1}.
ESI-HRMS (ESI-TOF): m/z calcd for $C_{32}H_{31}F_3KNO_3PS^+$ $[M+K]^+$: 636.1346, found 636.1360.

Compound 4aa (*E*)-bis(4-(*tert*-butyl)phenyl)(phenyl((4,4,4-trifluoro-2-tosylbut-1-en-1-yl)amino)methyl)phosphine oxide

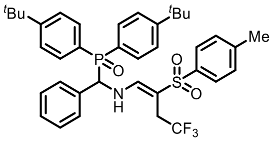

Chemical formula: $C_{38}H_{43}F_3NO_3PS$
Molecular weight: 681.79 g.mol^{-1}
White solid
Melting point: 230–232 °C
Yield: 72%

^{1}H NMR (500 MHz, DMSO) δ 8.01–7.96 (m, 1H), 7.92–7.88 (m, 2H), 7.86 (d, *J* = 13.0 Hz, 1H), 7.73–7.69 (m, 2H), 7.53–7.51 (m, 2H), 7.46–7.43 (m, 4H), 7.41–7.39 (m, 2H), 7.34–7.32 (m, 2H), 7.22–7.21 (m, 3H), 6.12 (dd, *J* = 9.5 Hz, *J* = 7.5 Hz, 1H), 3.33–3.28 (m, 1H), 3.07–2.97 (m, 1H), 1.23 (s, 10H), 1.20 (s, 10H).
^{13}C NMR (126 MHz, DMSO) δ 154.7 (d, $J_{C\text{-}P}$ = 2.6 Hz), 154.4 (d, $J_{C\text{-}P}$ = 2.6 Hz), 151.7 (d, $J_{C\text{-}P}$ = 6.5 Hz), 141.8, 141.2, 135.9, 130.8 (d, $J_{C\text{-}P}$ = 9.1 Hz), 130.7 (d, $J_{C\text{-}P}$ = 9.3 Hz), 129.3, 128.9 (d, $J_{C\text{-}P}$ = 4.7 Hz), 128.7 (d, $J_{C\text{-}P}$ = 97.3 Hz), 128.5 (d, $J_{C\text{-}P}$ = 99.7 Hz), 128.0, 127.7, 126.3, 125.9 (q, $J_{C\text{-}F}$ = 280.5 Hz), 125.4 (d, $J_{C\text{-}P}$ = 4.5 Hz), 125.3 (d, $J_{C\text{-}P}$ = 4.5 Hz), 94.8, 61.2 (d, $J_{C\text{-}P}$ = 73.8 Hz), 34.7, 34.6, 30.8, 30.7, 28.4 (q, $J_{C\text{-}F}$ = 31.2 Hz), 20.9.
^{19}F NMR (471 MHz, DMSO) δ -63.50 (s, 3F).
^{31}P NMR (202 MHz, DMSO) δ 29.48 (s, 1P).
IR (neat): ν = 3220, 2965, 1638, 1251, 1128, 663, 588 cm^{-1}.
ESI-HRMS (ESI-TOF): m/z calcd for $C_{38}H_{44}F_3NO_3PS^+$ $[M+H]^+$: 682.2726, found 682.2705.

Compound 4ab (*E*)-bis(4-methoxyphenyl)(phenyl((4,4,4-trifluoro-2-tosylbut-1-en-1-yl)amino)methyl)phosphine oxide

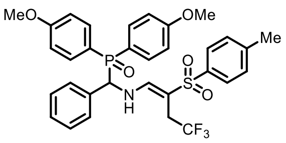

Chemical formula: $C_{32}H_{31}F_3NO_5PS$
Molecular weight: 629.63 g.mol^{-1}
White solid
Melting point: 227–229 °C
Yield: 66%

^{1}H NMR (500 MHz, DMSO) δ 8.00–7.90 (m, 3H), 7.74 (d, *J* = 13.0 Hz, 1H), 7.68–7.64 (m, 2H), 7.44–7.41 (m, 2H), 7.35–7.33 (m, 2H), 7.27–7.18 (m, 5H), 7.08–7.05 (m, 2H), 6.97–6.94 (m, 2H), 6.02 (dd, *J* = 9.5 Hz, *J* = 7.0 Hz, 1H), 3.79 (s, 3H), 3.74 (s, 3H), 3.32–3.22 (m, 1H), 3.14–3.04 (m, 1H), 2.35 (s, 3H).
^{13}C NMR (126 MHz, DMSO) δ 161.9 (d, $J_{C\text{-}P}$ = 2.6 Hz), 161.9 (d, $J_{C\text{-}P}$ = 2.8 Hz), 151.6 (d, $J_{C\text{-}P}$ = 5.5 Hz), 142.0, 140.9, 135.9, 133.0 (d, $J_{C\text{-}P}$ = 10.2 Hz), 132.8 (d, $J_{C\text{-}P}$ = 10.3 Hz), 129.3, 128.9 (d, $J_{C\text{-}P}$ = 4.8 Hz), 128.0, 127.7, 126.2, 126.0 (q, $J_{C\text{-}F}$ = 280.5 Hz), 122.8 (d, $J_{C\text{-}P}$ = 103.4 Hz), 122.7 (d, $J_{C\text{-}P}$ = 101.9 Hz), 114.3 (d, $J_{C\text{-}P}$ = 12.3 Hz), 114.1 (d, $J_{C\text{-}P}$ = 12.3 Hz), 94.1, 61.9 (d, $J_{C\text{-}P}$ = 74.1 Hz), 55.4, 55.4, 28.5 (q, $J_{C\text{-}F}$ = 31.0 Hz), 21.0.
^{19}F NMR (282 MHz, DMSO) δ -63.08 (s, 3F).
^{31}P NMR (121 MHz, DMSO) δ 29.60 (s, 1P).
IR (neat): ν = 3214, 1638, 1598, 1250, 1171, 1130, 1080, 772, 661, 541 cm^{-1}.
ESI-HRMS (ESI-TOF): m/z calcd for $C_{32}H_{32}F_3NO_5PS^+$ $[M+H]^+$: 630.1685, found 630.1668.

Compound 4ac (*E*)-(phenyl((4,4,4-trifluoro-2-tosylbut-1-en-1-yl)amino)methyl)bis(4-(trifluoromethyl)phenyl)phosphine oxide

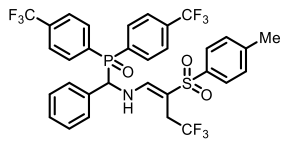

Chemical formula: $C_{32}H_{25}F_9NO_3PS$
Molecular weight: 705.57 g.mol^{-1}
White solid
Melting point: 228–230 °C
Yield: 84%

^{1}H NMR (500 MHz, DMSO) δ 8.34–8.31 (m, 2H), 8.22–8.17 (m, 1H), 8.10–8.07 (m, 2H), 7.91–7.89 (m, 2H), 7.86–7.83 (m, 3H), 7.46–7.43 (m, 4H), 7.31–7.21 (m, 5H), 6.42 (dd, *J* = 9.5 Hz, *J* = 7.5 Hz, 1H), 3.39–3.30 (m, 1H), 3.09–3.00 (m, 1H), 2.34 (s, 3H).
^{13}C NMR (126 MHz, DMSO) δ 151.2 (d, J_{C-P} = 6.7 Hz), 142.1, 140.7, 135.7 (d, J_{C-P} = 91.4 Hz), 135.7 (d, J_{C-P} = 94.1 Hz), 134.6, 132.2 (q, J_{C-F} = 32.3 Hz), 132.1 (q, J_{C-F} = 32.3 Hz), 132.1 (d, J_{C-P} = 9.3 Hz), 131.9 (d, J_{C-P} = 9.4 Hz), 129.3, 128.9 (d, J_{C-P} = 4.9 Hz), 128.3, 128.2, 126.2, 125.9 (q, J_{C-F} = 280.4 Hz), 125.6 (q, J_{C-F} = 3.9 Hz), 125.5 (q, J_{C-F} = 4.8 Hz), 123.7 (q, J_{C-F} = 273.4 Hz), 123.6 (q, J_{C-F} = 273.4 Hz), 95.7, 61.2 (d, J_{C-P} = 75.8 Hz), 28.4 (q, J_{C-F} = 31.1 Hz), 20.9.
^{19}F NMR (471 MHz, DMSO) δ -61.77 (s, 3F), -61.82 (s, 3F), -63.50 (s, 3F).
^{31}P NMR (202 MHz, DMSO) δ 27.86 (s, 1P).
IR (neat): ν = 3250, 1638, 1391, 1127, 1016, 700, 546 cm^{-1}.
ESI-HRMS (ESI-TOF): m/z calcd for $C_{32}H_{25}F_9KNO_3PS^+$ [M+K]$^+$: 744.0781, found 744.0759.

Compound 4ad (*E*)-bis(3,5-dimethylphenyl)(phenyl((4,4,4-trifluoro-2-tosylbut-1-en-1-yl)amino)methyl)phosphine oxide

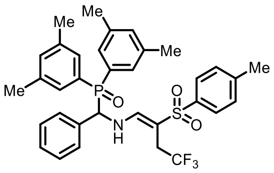

Chemical formula: $C_{34}H_{35}F_3NO_3PS$
Molecular weight: 625.69 g.mol^{-1}
White solid
Melting point: 248–250 °C
Yield: 81%

^{1}H NMR (500 MHz, DMSO) δ 7.96–7.91 (m, 1H), 7.70 (d, *J* = 13.5 Hz, 1H), 7.60–7.58 (m, 2H), 7.49–7.47 (m, 2H), 7.43–7.41 (m, 2H), 7.31–7.29 (m, 2H), 7.26–7.17 (m, 6H), 7.09 (s, 1H), 6.06 (dd, *J* = 9.5 Hz, *J* = 6.5 Hz, 1H), 3.30–3.21 (m, 1H), 3.14–3.04 (m, 1H), 2.35 (s, 3H), 2.29 (s, 6H), 2.23 (s, 6H).
^{13}C NMR (126 MHz, DMSO) δ 151.4 (d, J_{C-P} = 5.4 Hz), 142.1, 140.7, 137.8 (d, J_{C-P} = 12.1 Hz), 137.7 (d, J_{C-P} = 12.1 Hz), 135.8, 133.3 (d, J_{C-P} = 2.6 Hz), 133.2 (d, J_{C-P} = 2.6 Hz), 131.4 (d, J_{C-P} = 95.9 Hz), 131.2 (d, J_{C-P} = 94.0 Hz), 129.3, 129.1 (d, J_{C-P} = 4.9 Hz), 128.7 (d, J_{C-P} = 8.9 Hz), 128.5 (d, J_{C-P} = 8.8 Hz), 128.0, 127.7, 126.1, 126.0 (q, J_{C-F} = 280.5 Hz), 94.0, 61.5 (d, J_{C-P} = 73.5 Hz), 28.5 (q, J_{C-F} = 31.0 Hz), 21.0, 21.0, 20.9.
^{19}F NMR (282 MHz, DMSO) δ -63.07 (s, 3F).
^{31}P NMR (121 MHz, DMSO) δ 29.68 (s, 1P).
IR (neat): ν = 3221, 1636, 1251, 1133, 1027, 662, 578 cm^{-1}.
ESI-HRMS (ESI-TOF): m/z calcd for $C_{34}H_{36}F_3NO_3PS^+$ [M+H]$^+$: 626.2100, found 626.2058.

Compound 4ae (*E*)-di(naphthalen-1-yl)(phenyl((4,4,4-trifluoro-2-tosylbut-1-en-1-yl)amino)methyl)phosphine oxide

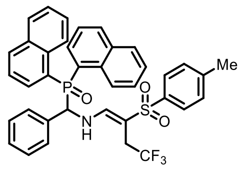

Chemical formula: $C_{38}H_{31}F_3NO_3PS$
Molecular weight: 669.70 g.mol^{-1}
White solid
Melting point: 185–187 °C
Yield: 42%

^{1}H NMR (500 MHz, DMSO) δ 8.67 (d, *J* = 9.0 Hz, 1H), 8.61 (d, *J* = 8.5 Hz, 1H), 8.42–8.38 (m, 1H), 8.20 (d, *J* = 8.0 Hz, 1H), 8.10–7.97 (m, 4H), 7.89–7.84 (m, 2H), 7.67–7.63 (m, 1H), 7.49–7.41 (m, 5H), 7.39–7.30 (m, 6H), 7.07–7.03 (m, 3H), 6.48–6.45 (m, 1H), 3.38–3.35 (m, 1H), 3.18–3.08 (m, 1H), 2.37 (s, 3H).
^{13}C NMR (126 MHz, DMSO) δ 151.1 (d, J_{C-P} = 7.7 Hz), 142.2, 140.7, 136.1, 133.6 (d, J_{C-P} = 8.7 Hz), 133.4 (d, J_{C-P} = 7.2 Hz), 133.3, 133.2 (d, J_{C-P} = 1.1 Hz), 133.1 (d, J_{C-P} = 1.1 Hz), 133.0 (d, J_{C-P} = 8.9 Hz), 132.5 (d, J_{C-P} = 10.4 Hz), 132.2 (d, J_{C-P} = 9.8 Hz), 129.4, 129.2, 128.9 (d, J_{C-P} = 4.8 Hz), 128.7, 127.9 (d, J_{C-P} = 93.7 Hz), 127.8 (d, J_{C-P} = 92.1 Hz), 127.8, 127.7, 127.1, 126.8, 126.3, 126.2, 126.2, 126.1 (q, J_{C-F} = 280.2 Hz), 125.7 (d, J_{C-P} = 3.9 Hz), 124.6 (d, J_{C-P} = 9.2 Hz), 124.5 (d, J_{C-P} = 9.4 Hz), 95.9, 61.1 (d, J_{C-P} = 76.2 Hz), 28.5 (q, J_{C-F} = 30.9 Hz), 21.0.
^{19}F NMR (471 MHz, DMSO) δ -62.98 (s, 3F).
^{31}P NMR (202 MHz, DMSO) δ 35.59 (s, 1P).
IR (neat): ν = 3318, 3044, 1635, 1252, 1126, 1080, 772, 685, 553 cm^{-1}.
ESI-HRMS (ESI-TOF): m/z calcd for $C_{38}H_{32}F_3NO_3PS^+$ [M+H]$^+$: 670.1787, found 670.1745.

Compound 7a *N*-benzyl-4-methyl-N-(propa-1,2-dien-1-yl)benzenesulfonamide

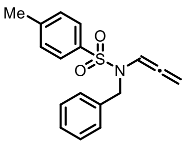

Chemical formula: $C_{17}H_{17}NO_2S$
Molecular weight: 299.39 g.mol^{-1}

^{1}H NMR (400 MHz, CDCl$_3$) δ 7.73 (d, *J* = 8.0 Hz, 2H), 7.33 (m, 7H), 6.84 (t, *J* = 6.0 Hz, 1H), 5.15 (d, *J* = 6.0 Hz, 2H), 4.30 (s, 2H), 2.45 (s, 3H).
Data match with those described in the literature.[8]

Compound 7b *N*-(4-methoxybenzyl)-4-methyl-*N*-(propa-1,2-dien-1-yl)benzenesulfonamide

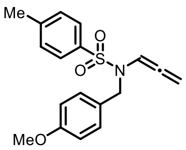

Chemical formula: $C_{18}H_{19}NO_3S$
Molecular weight: 329.41 g.mol^{-1}

^{1}H NMR (400 MHz, CDCl$_3$) δ 7.72 (d, *J* = 7.6 Hz, 2H), 7.33 (d, *J* = 7.7 Hz, 2H), 7.24 (d, *J* = 8.0 Hz, 2H), 6.83–6.78 (m, 3H), 5.17 (d, *J* = 6.1 Hz, 2H), 4.23 (s, 2H), 3.79 (s, 3H), 2.44 (s, 3H).
Data match with those described in the literature.[8]

Compound 7c 4-Methyl-*N*-(propa-1,2-dien-1-yl)-*N*-(4-(trifluoromethyl)benzyl)benzenesulfonamide

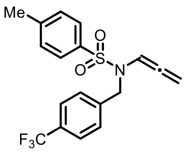

Chemical formula: $C_{18}H_{16}F_3NO_2S$
Molecular weight: 367.39 g.mol^{-1}

^{1}H NMR (400 MHz, CDCl$_3$) δ 7.71 (d, *J* = 8.3 Hz, 2H), 7.54 (d, *J* = 8.2 Hz, 2H), 7.41 (d, *J* = 8.1 Hz, 2H), 7.33 (d, *J* = 8.2 Hz, 2H), 6.87 (t, *J* = 6.2 Hz, 1H), 5.15 (d, *J* = 6.2 Hz, 2H), 4.35 (s, 2H), 2.44 (s, 3H).
Data match with those described in the literature.[8]

Compound 7d *N*-(4-chlorobenzyl)-4-methyl-*N*-(propa-1,2-dien-1-yl)benzenesulfonamide

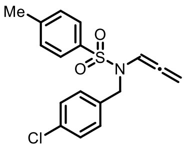

Chemical formula: $C_{17}H_{16}ClNO_2S$
Molecular weight: 333.83 g.mol^{-1}

^{1}H NMR (500 MHz, CDCl$_3$) δ 7.59–7.57 (m, 2H), 7.19–7.11 (m, 6H), 6.70 (t, *J* = 6.0 Hz, 1H), 5.01 (d, *J* = 6.0 Hz, 2H), 4.13 (s, 2H), 2.28 (s, 3H).
Data match with those described in the literature.[9]

Compound 7e *N*-(benzo[*d*][1,3]dioxol-5-ylmethyl)-4-methyl-*N*-(propa-1,2-dien-1-yl)benzenesulfonamide

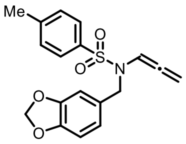

Chemical formula: $C_{18}H_{17}NO_4S$
Molecular weight: 343.40 g.mol^{-1}

^{1}H NMR (400 MHz, CDCl$_3$) δ 7.71 (d, *J* = 8.2 Hz, 2H), 7.33 (d, *J* = 8.0 Hz, 2H), 6.84 (s, 1H), 6.80 (t, *J* = 6.2 Hz, 1H), 6.72 (q, *J* = 14.4 Hz, *J* = 8.1 Hz, 2H), 5.93 (s, 2H), 5.19 (d, *J* = 6.2 Hz, 2H), 4.19 (s, 2H), 2.44 (s, 3H).
Data match with those described in the literature.[8]

Compound 7f 4-Methyl-*N*-(propa-1,2-dien-1-yl)-*N*-(thiophen-2-ylmethyl)benzenesulfonamide

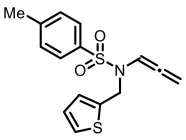

Chemical formula: $C_{15}H_{15}NO_2S_2$
Molecular weight: 305.41 g.mol^{-1}
Yellow liquid

^{1}H NMR (500 MHz, CDCl$_3$) δ 7.66–7.64 (m, 2H), 7.28–7.26 (m, 2H), 7.19–7.17 (m, 1H), 6.95–6.94 (m, 1H), 6.89–6.88 (m, 1H), 6.82–6.79 (m, 1H), 5.32 (d, *J* = 6.0 Hz, 2H), 4.54 (s, 2H), 2.42 (s, 3H).
^{13}C NMR (126 MHz, CDCl$_3$) δ 201.6, 143.9, 138.6, 135.6, 129.8, 127.4, 127.3, 126.5, 125.8, 100.0, 88.9, 45.1, 21.7.
IR (neat): ν = 2924, 1347, 1158, 1090, 663, 544 cm^{-1}.
ESI-HRMS (ESI-TOF): m/z calcd for $C_{15}H_{15}NNaO_2S_2^+$ [M+Na]$^+$: 328.0436, found 328.0441.

Compound 7g 4-Methyl-*N*-phenyl-*N*-(propa-1,2-dien-1-yl)benzenesulfonamide

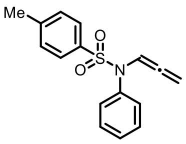

Chemical formula: $C_{16}H_{15}NO_2S$
Molecular weight: 285.36 g.mol^{-1}

^{1}H NMR (300 MHz, CDCl$_3$) δ = 7.56 (d, *J* = 8.3 Hz, 2H), 7.32–7.22 (m, 5H), 7.11 (t, *J* = 6.3 Hz, 1H), 7.06–6.96 (m, 2H), 5.02 (d, *J* = 6.4 Hz, 2H), 2.44 (s, 3H).
Data match with those described in the literature.[10]

Compound 7h *N*-(4-methoxyphenyl)-4-methyl-*N*-(propa-1,2-dien-1-yl)benzenesulfonamide

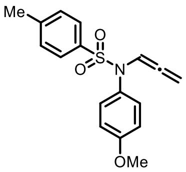

Chemical formula: $C_{17}H_{17}NO_3S$
Molecular weight: 315.39 g.mol^{-1}

^{1}H NMR (300 MHz, CDCl$_3$) δ = 7.55 (d, *J* = 8.0 Hz, 2H), 7.27 (d, *J* = 8.0 Hz, 2H), 7.12 (t, *J* = 6.2 Hz, 1H), 6.93–6.85 (m, 2H), 6.82–6.74 (m, 2H), 5.03 (d, *J* = 6.2 Hz, 2H), 3.79 (s, 3H), 2.44 (s, 3H).
Data match with those described in the literature.[10]

Compound 7i *N*-(3-methoxyphenyl)-4-methyl-*N*-(propa-1,2-dien-1-yl)benzenesulfonamide

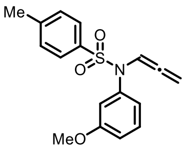

Chemical formula: $C_{17}H_{17}NO_3S$
Molecular weight: 315.39 g.mol^{-1}
Yellow liquid

^{1}H NMR (500 MHz, CDCl$_3$) δ 7.59–7.57 (m, 2H), 7.29–7.27 (m, 2H), 7.18–7.15 (m, 1H), 7.08 (t, *J* = 6.5 Hz, 1H), 6.86–6.84 (m, 1H), 6.58–6.55 (m, 2H), 5.04 (d, *J* = 6.0 Hz, 2H), 3.71 (s, 3H), 2.44 (s, 3H).
^{13}C NMR (126 MHz, CDCl$_3$) δ 201.3, 159.9, 144.1, 138.4, 135.5, 129.7, 129.4, 127.9, 121.8, 115.1, 114.9, 102.4, 87.6, 55.4, 21.8.
IR (neat): ν = 2960, 1601, 1487, 1358, 1167, 691, 578 cm^{-1}.
ESI-HRMS (ESI-TOF): m/z calcd for $C_{17}H_{17}KNO_3S^+$ [M+K]$^+$: 354.0561, found 354.0545.

Compound 7j 4-Methyl-*N*-(propa-1,2-dien-1-yl)-*N*-(o-tolyl)benzenesulfonamide

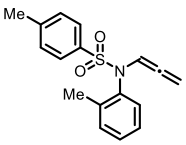

Chemical formula: $C_{17}H_{17}NO_2S$
Molecular weight: 299.39 g.mol^{-1}
Yellow liquid

^{1}H NMR (500 MHz, CDCl$_3$) δ 7.65–7.63 (m, 2H), 7.30 (d, *J* = 9.0 Hz, 2H), 7.25–7.20 (m, 2H), 7.14 (t, *J* = 6.0 Hz, 1H), 7.03–7.00 (m, 1H), 6.55 (d, *J* = 8.0 Hz, 1H), 4.98 (d, *J* = 7.0 Hz, 2H), 2.45 (s, 3H), 2.27 (s, 3H).

^{13}C NMR (126 MHz, CDCl$_3$) δ 201.2, 144.0, 139.3, 136.4, 136.1, 131.3, 129.8, 129.4, 129.1, 127.8, 126.1, 102.4, 87.4, 21.8, 18.1.
IR (neat): ν = 2977, 1491, 1356, 1164, 663, 583 cm^{-1}.
ESI-HRMS (ESI-TOF): m/z calcd for $C_{17}H_{18}NO_2S^+$ [M+H]$^+$: 300.1053, found 300.1040.

Compound 7k 4-Methyl-*N*-octyl-*N*-(propa-1,2-dien-1-yl)benzenesulfonamide

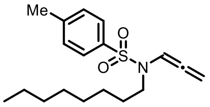

Chemical formula: $C_{18}H_{27}NO_2S$
Molecular weight: 321.48 g.mol^{-1}
Yellow liquid

^{1}H NMR (500 MHz, CDCl$_3$) δ 7.68–7.66 (m, 2H), 7.30–7.28 (m, 2H), 6.82–6.80 (m, 1H), 5.27 (d, *J* = 6.5 Hz, 2H), 3.09–3.06 (m, 2H), 2.41 (s, 3H), 1.53–1.50 (m, 2H), 1.28–1.24 (m, 10H), 0.86 (t, *J* = 6.5 Hz, 3H).
^{13}C NMR (126 MHz, CDCl$_3$) δ 201.6, 143.7, 135.7, 129.8, 127.2, 100.2, 87.4, 46.7, 31.9, 29.3, 29.3, 28.0, 26.6, 22.7, 21.6, 14.2.
IR (neat): ν = 2925, 2855, 1356, 1163, 1094, 664, 590, 546 cm^{-1}.
ESI-HRMS (ESI-TOF): m/z calcd for $C_{18}H_{27}KNO_2S^+$ [M+K]$^+$: 360.1394, found 360.1374.

Compound 7l *N*-cyclopropyl-4-methyl-*N*-(propa-1,2-dien-1-yl)benzenesulfonamide

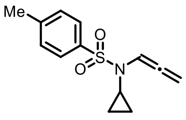

Chemical formula: $C_{13}H_{15}NO_2S$
Molecular weight: 249.33 g.mol^{-1}

^{1}H NMR (300 MHz, CDCl$_3$) δ = 7.74 (d, *J* = 8.2 Hz, 2H), 7.33 (d, *J* = 8.0 Hz, 2H), 6.76 (t, *J* = 6.2 Hz, 1H), 5.23 (d, *J* = 6.2 Hz, 2H), 2.44 (s, 3H), 1.73–1.69 (m, 1H), 0.98–0.95 (m, 2H), 0.72–0.69 (m, 2H).
Data match with those described in the literature.[4]

Compound 7m 4-Methyl-*N*-(2-(2-oxocyclopentyl)ethyl)-*N*-(propa-1,2-dien-1-yl)benzenesulfonamide

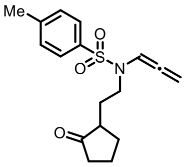

Chemical formula: $C_{17}H_{21}NO_3S$
Molecular weight: 319.42 g.mol^{-1}
Yellow liquid

^{1}H NMR (500 MHz, CDCl$_3$) δ 7.68–7.66 (m, 2H), 7.32–7.29 (m, 2H), 6.77 (t, *J* = 6.5 Hz, 1H), 5.33–5.26 (m, 2H), 3.27–3.12 (m, 2H), 2.42 (s, 3H), 2.40–2.27 (m, 2H), 2.17–1.97 (m, 4H), 1.84–1.74 (m, 1H), 1.53–1.36 (m, 2H).
^{13}C NMR (126 MHz, CDCl$_3$) δ 220.8, 201.7, 143.9, 135.2, 129.9, 127.3, 99.9, 87.8, 46.9, 45.0, 38.0, 29.9, 28.0, 21.7, 20.8.
IR (neat): ν = 2961, 1733, 1351, 1162, 1093, 664, 590, 545 cm^{-1}.
ESI-HRMS (ESI-TOF): m/z calcd for $C_{17}H_{21}NNaO_3S^+$ [M+Na]$^+$: 342.1134, found 342.1137.

Compound 7n *N*-(2-(1,3-dioxolan-2-yl)ethyl)-4-methyl-*N*-(propa-1,2-dien-1-yl)benzenesulfonamide

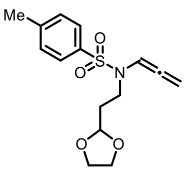

Chemical formula: $C_{15}H_{19}NO_4S$
Molecular weight: 309.38 g.mol^{-1}

^{1}H NMR (300 MHz, CDCl$_3$) δ = 7.69 (d, *J* = 8.3 Hz, 2H), 7.30 (d, *J* = 8.1 Hz, 2H), 6.83 (t, *J* = 6.2 Hz, 1H), 5.32 (d, *J* = 6.2 Hz, 2H), 4.90 (t, *J* = 4.7 Hz, 1H), 3.95–3.92 (m, 2H), 3.85–3.82 (m, 2H), 3.27–3.24 (m, 2H), 2.42 (s, 3H), 1.92–1.88 (m, 2H).
Data match with those described in the literature.[4]

Compound 7o *N*-(2-(1,3-dioxo-1,3,3a,4,7,7a-hexahydro-2*H*-isoindol-2-yl)ethyl)-4-methyl-*N*-(propa-1,2-dien-1-yl)benzenesulfonamide

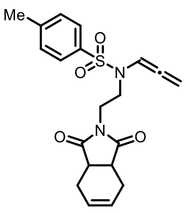

Chemical formula: $C_{20}H_{22}N_2O_4S$
Molecular weight: 386.47 g.mol^{-1}

^{1}H NMR (300 MHz, CDCl$_3$) δ = 7.63 (d, *J* = 7.9 Hz, 2H), 7.29 (d, *J* = 7.7 Hz, 2H), 6.81 (t, *J* = 5.9 Hz, 1H), 5.91–5.89 (m, 2H), 5.39 (d, *J* = 6.1 Hz, 2H), 3.60–3.59 (m, 2H), 3.27–3.24 (m, 2H), 3.18–3.15 (m, 2H), 2.60–2.57 (m, 2H), 2.41 (s, 3H), 2.28–2.26 (m, 2H).

Data match with those described in the literature.[4]

Compound 7p *N*-benzyl-2,4,6-triisopropyl-*N*-(propa-1,2-dien-1-yl)benzenesulfonamide

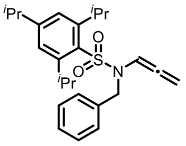

Chemical formula: $C_{25}H_{33}NO_2S$
Molecular weight: 411.60 g.mol^{-1}

^{1}H NMR (400 MHz, CDCl$_3$) δ 7.28–7.22 (m, 5H), 7.19 (s, 2H), 6.68 (t, *J* = 6.2 Hz, 1H), 5.15 (d, *J* = 6.2 Hz, 2H), 4.47 (s, 2H), 4.19–4.13 (m, 2H), 2.95–2.88 (m, 1H), 1.27 (t, *J* = 7.0 Hz, 18H).
Data match with those described in the literature.[8]

Compound 7q *N*-benzyl-*N*-(propa-1,2-dien-1-yl)thiophene-2-sulfonamide

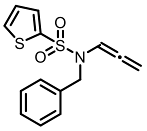

Chemical formula: $C_{14}H_{13}NO_2S_2$
Molecular weight: 291.38 g.mol^{-1}
Yellow liquid

^{1}H NMR (500 MHz, CDCl$_3$) δ 7.55 (dd, *J* = 5.5 Hz, *J* = 1.5 Hz, 1H), 7.52 (dd, *J* = 3.5 Hz, *J* = 1.0 Hz, 1H), 7.25–7.18 (m, 5H), 7.06–7.04 (m, 1H), 6.71–6.68 (m, 1H), 5.12 (d, *J* = 6.0 Hz, 2H), 4.29 (s, 2H).
^{13}C NMR (126 MHz, CDCl$_3$) δ 202.6, 138.4, 136.0, 132.5, 132.4, 128.5, 128.1, 127.7, 127.6, 99.7, 88.3, 50.4.
IR (neat): ν = 3093, 1358, 1160, 603, 570 cm^{-1}.
ESI-HRMS (ESI-TOF): m/z calcd for $C_{14}H_{14}NO_2S_2^+$ [M+H]$^+$: 292.0460, found 292.0447.

Compound 7t *N*-benzyl-*N*-(buta-1,2-dien-1-yl)-4-methylbenzenesulfonamide

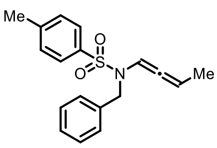

Chemical formula: $C_{18}H_{19}NO_2S$
Molecular weight: 313.42 g.mol^{-1}
Yellow liquid

^{1}H NMR (500 MHz, CDCl$_3$) δ 7.73–7.70 (m, 2H), 7.33–7.30 (m, 2H), 7.29–7.19 (m, 5H), 6.72–6.69 (m, 1H), 5.48–5.43 (m, 1H), 4.46 (d, *J* = 15.0 Hz, 1H), 4.08 (d, *J* = 15.0 Hz, 1H), 2.43 (s, 3H), 1.32–1.30 (m, 3H).
^{13}C NMR (126 MHz, CDCl$_3$) δ 197.3, 143.8, 136.4, 135.6, 129.8, 128.4, 127.9, 127.4, 127.4, 99.3, 99.1, 50.2, 21.7, 15.8.
IR (neat): ν = 3087, 2897, 1349, 1164, 1091, 664, 588 cm^{-1}.
ESI-HRMS (ESI-TOF): m/z calcd for $C_{18}H_{19}NNaO_2S^+$ [M+Na]$^+$: 336.1025, found 336.1039.

Compound 7u *N*-benzyl-4-methyl-*N*-(3-phenylpropa-1,2-dien-1-yl)benzenesulfonamide

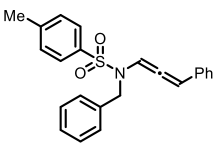

Chemical formula: $C_{23}H_{21}NO_2S$
Molecular weight: 375.49 g.mol^{-1}
Yellow liquid

Data match with those described in the literature.[11]

Compound 8a (*E*)-(3-(benzylamino)-2-tosylallyl)diphenylphosphine oxide

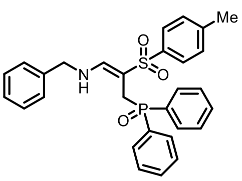

Chemical formula: $C_{29}H_{28}NO_3PS$
Molecular weight: 501.58 g.mol^{-1}
White solid
Melting point: 72–74 °C
Yield: 81%

^{1}H NMR (500 MHz, CDCl$_3$) δ 8.64–8.59 (m, 1H), 7.62–7.58 (m, 4H), 7.47–7.39 (m, 5H), 7.33–7.29 (m, 4H), 7.23–7.16 (m, 3H), 7.11–7.06 (m, 4H), 4.27 (d, *J* = 6.0 Hz, 2H), 3.16 (d, *J* = 13.5 Hz, 2H), 2.28 (s, 3H).

^{13}C NMR (126 MHz, CDCl$_3$) δ 149.9 (d, J_{C-P} = 2.9 Hz), 142.4, 140.2, 138.6, 132.2 (d, J_{C-P} = 2.8 Hz), 131.0 (d, J_{C-P} = 100.3 Hz), 130.9 (d, J_{C-P} = 9.6 Hz), 129.6, 128.7 (d, J_{C-P} = 12.1 Hz), 128.7, 127.5, 127.2, 126.6, 94.0 (d, J_{C-P} = 4.4 Hz), 52.8, 29.2 (d, J_{C-P} = 68.3 Hz), 21.5.
^{31}P NMR (121 MHz, CDCl$_3$) δ 35.01 (s, 1P).
IR (neat): ν = 3060, 1647, 1283, 1136, 737, 659 cm^{-1}.
ESI-HRMS (ESI-TOF): m/z calcd for $C_{29}H_{28}KNO_3PS^+$ [M+K]$^+$: 540.1159, found 540.1120.

Compound 8b (*E*)-(3-((4-methoxybenzyl)amino)-2-tosylallyl)diphenylphosphine oxide

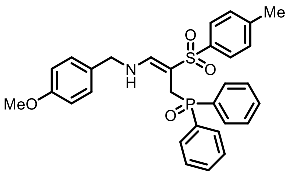

Chemical formula: $C_{30}H_{30}NO_4PS$
Molecular weight: 531.61 g.mol^{-1}
White solid
Melting point: 78–80 °C
Yield: 83%

^{1}H NMR (500 MHz, CDCl$_3$) δ 8.64–8.59 (m, 1H), 7.69–7.64 (m, 4H), 7.55–7.46 (m, 5H), 7.40–7.36 (m, 4H), 7.15–7.10 (m, 4H), 6.84–6.81 (m, 2H), 4.29 (d, *J* = 6.0 Hz, 2H), 3.79 (s, 3H), 3.22 (d, *J* = 14.0 Hz, 2H), 2.35 (s, 3H).
^{13}C NMR (126 MHz, CDCl$_3$) δ 159.0, 149.7 (d, J_{C-P} = 2.8 Hz), 142.3, 140.2, 132.1 (d, J_{C-P} = 2.8 Hz), 130.9 (d, J_{C-P} = 9.6 Hz), 130.6, 130.4 (d, J_{C-P} = 100.3 Hz), 129.6, 128.7 (d, J_{C-P} = 12.1 Hz), 128.6, 126.6, 114.1, 93.7 (d, J_{C-P} = 4.3 Hz), 55.3, 52.3, 29.2 (d, J_{C-P} = 68.3 Hz), 21.5.
^{31}P NMR (121 MHz, CDCl$_3$) δ 34.96 (s, 1P).
IR (neat): ν = 3059, 1643, 1512, 1135, 735, 658, 515 cm^{-1}.
ESI-HRMS (ESI-TOF): m/z calcd for $C_{30}H_{30}NNaO_4PS^+$ [M+Na]$^+$: 554.1525, found 554.1533.

Compound 8c (*E*)-diphenyl(2-tosyl-3-((4-(trifluoromethyl)benzyl)amino)allyl)phosphine oxide

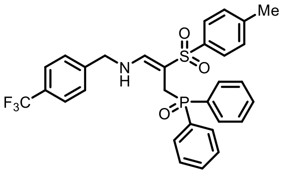

Chemical formula: $C_{30}H_{27}F_3NO_3PS$
Molecular weight: 569.58 g.mol^{-1}
White solid
Melting point: 70–72 °C
Yield: 78%

^{1}H NMR (500 MHz, CDCl$_3$) δ 8.80–8.75 (m, 1H), 7.70–7.65 (m, 4H), 7.56–7.49 (m, 7H), 7.43–7.39 (m, 4H), 7.28–7.26 (m, 2H), 7.19–7.17 (m, 2H), 4.41 (d, *J* = 6.0 Hz, 2H), 3.23 (d, *J* = 14.0 Hz, 2H), 2.38 (s, 3H).
^{13}C NMR (126 MHz, CDCl$_3$) δ 149.6 (d, J_{C-P} = 3.0 Hz), 142.8, 142.7, 140.0, 132.4 (d, J_{C-P} = 2.9 Hz), 131.0 (d, J_{C-P} = 9.7 Hz), 130.9 (d, J_{C-P} = 100.5 Hz), 129.7, 128.9 (d, J_{C-P} = 12.1 Hz), 127.5, 126.8, 125.7 (q, J_{C-F} = 3.8 Hz), 124.2 (q, J_{C-F} = 272.5 Hz), 95.2 (d, J_{C-P} = 4.5 Hz), 52.4, 29.2 (d, J_{C-P} = 67.8 Hz), 21.6.
^{19}F NMR (471 MHz, CDCl$_3$) δ -62.47 (s, 3F).
^{31}P NMR (202 MHz, CDCl$_3$) δ 35.43 (s, 1P).
IR (neat): ν = 2925, 1648, 1325, 1067, 738, 660, 543 cm^{-1}.
ESI-HRMS (ESI-TOF): m/z calcd for $C_{30}H_{27}F_3NNaO_3PS^+$ [M+Na]$^+$: 592.1294, found 592.1287.

Compound 8d (*E*)-(3-((4-chlorobenzyl)amino)-2-tosylallyl)diphenylphosphine oxide

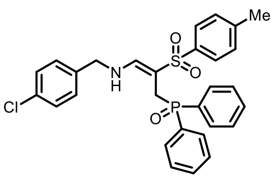

Chemical formula: $C_{29}H_{27}ClNO_3PS$
Molecular weight: 536.02 g.mol^{-1}
White solid
Melting point: 70–72 °C
Yield: 86%

^{1}H NMR (500 MHz, CDCl$_3$) δ 8.72–8.67 (m, 1H), 7.69–7.65 (m, 4H), 7.55–7.48 (m, 5H), 7.42–7.38 (m, 4H), 7.25–7.23 (m, 2H), 7.18–7.16 (m, 2H), 7.10–7.08 (m, 2H), 4.31 (d, *J* = 5.5 Hz, 2H), 3.22 (d, *J* = 14.0 Hz, 2H), 2.37 (s, 3H).
^{13}C NMR (126 MHz, CDCl$_3$) δ 149.6 (d, J_{C-P} = 3.0 Hz), 142.5, 140.0, 137.2, 133.3, 132.3 (d, J_{C-P} = 2.8 Hz), 130.9 (d, J_{C-P} = 9.7 Hz), 130.9 (d, J_{C-P} = 100.5 Hz), 129.7, 128.9, 128.8 (d, J_{C-P} = 12.1 Hz), 128.6, 126.7, 94.7 (d, J_{C-P} = 5.8 Hz), 52.2, 29.2 (d, J_{C-P} = 67.9 Hz), 21.5.
^{31}P NMR (121 MHz, CDCl$_3$) δ 35.12 (s, 1P).
IR (neat): ν = 3060, 1647, 1137, 659, 514 cm^{-1}.
ESI-HRMS (ESI-TOF): m/z calcd for $C_{29}H_{27}ClNNaO_3PS^+$ [M+Na]$^+$: 558.1030, found 558.1020.

Compound 8e (*E*)-(3-((benzo[*d*][1,3]dioxol-5-ylmethyl)amino)-2-tosylallyl)diphenylphosphine oxide

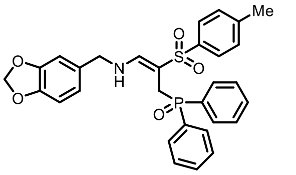

Chemical formula: $C_{30}H_{28}NO_5PS$
Molecular weight: 545.59 g.mol^{-1}
White solid
Melting point: 83–85 °C
Yield: 57%

^{1}H NMR (300 MHz, CDCl$_3$) δ 8.66–8.57 (m, 1H), 7.72–7.65 (m, 4H), 7.56–7.37 (m, 9H), 7.18–7.15 (m, 2H), 6.73–6.62 (m, 3H), 5.95 (s, 2H), 4.25 (d, J = 5.4 Hz, 2H), 3.22 (d, J = 13.5 Hz, 2H), 2.37 (s, 3H).
^{13}C NMR (126 MHz, CDCl$_3$) δ 149.7 (d, $J_{C\text{-}P}$ = 2.9 Hz), 148.0, 147.1, 142.4, 140.2, 132.5, 132.3 (d, $J_{C\text{-}P}$ = 2.9 Hz), 131.0 (d, $J_{C\text{-}P}$ = 9.7 Hz), 131.0 (d, $J_{C\text{-}P}$ = 100.4 Hz), 129.7, 128.8 (d, $J_{C\text{-}P}$ = 12.1 Hz), 126.7, 120.7, 108.4, 107.9, 101.2, 94.1 (d, $J_{C\text{-}P}$ = 4.4 Hz), 52.7, 29.3 (d, $J_{C\text{-}P}$ = 68.2 Hz), 21.6.
^{31}P NMR (121 MHz, CDCl$_3$) δ 35.00 (s, 1P).
IR (neat): ν = 3055, 1641, 1437, 1195, 658, 537 cm^{-1}.
ESI-HRMS (ESI-TOF): m/z calcd for $C_{30}H_{28}KNO_5PS^+$ [M+K]$^+$: 584.1057, found 584.1060.

Compound 8f (*E*)-diphenyl(3-((thiophen-2-ylmethyl)amino)-2-tosylallyl)phosphine oxide

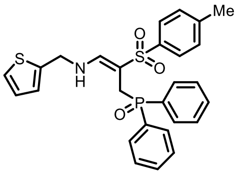

Chemical formula: $C_{27}H_{26}NO_3PS_2$
Molecular weight: 507.60 g.mol^{-1}
White solid
Melting point: 85–87 °C
Yield: 62%

^{1}H NMR (500 MHz, CDCl$_3$) δ 8.76–8.72 (m, 1H), 7.69–7.65 (m, 4H), 7.56–7.48 (m, 5H), 7.42–7.38 (m, 4H), 7.23 (dd, J = 5.0 Hz, J = 1.0 Hz, 1H), 7.16–7.14 (m, 2H), 6.95–6.93 (m, 1H), 6.91–6.90 (m, 1H), 4.52 (d, J = 5.5 Hz, 2H), 3.21 (d, J = 13.5 Hz, 2H), 2.37 (s, 3H).
^{13}C NMR (126 MHz, CDCl$_3$) δ 149.3 (d, $J_{C\text{-}P}$ = 2.9 Hz), 142.5, 141.8, 140.0, 132.2 (d, $J_{C\text{-}P}$ = 2.8 Hz), 131.0 (d, $J_{C\text{-}P}$ = 9.6 Hz), 130.9 (d, $J_{C\text{-}P}$ = 100.4 Hz), 129.7, 128.8 (d, $J_{C\text{-}P}$ = 12.1 Hz), 127.2, 126.8, 125.7, 125.3, 95.0 (d, $J_{C\text{-}P}$ = 4.5 Hz), 47.5, 29.2 (d, $J_{C\text{-}P}$ = 68.2 Hz), 21.6.
^{31}P NMR (121 MHz, CDCl$_3$) δ 35.01 (s, 1P).
IR (neat): ν = 3072, 1645, 1284, 1136, 737, 659, 541 cm^{-1}.
ESI-HRMS (ESI-TOF): m/z calcd for $C_{27}H_{26}NNaO_3PS_2^+$ [M+Na]$^+$: 530.0984, found 530.0983.

Compound 8g (*E*)-diphenyl(3-(phenylamino)-2-tosylallyl)phosphine oxide

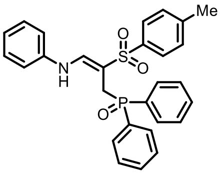

Chemical formula: $C_{28}H_{26}NO_3PS$
Molecular weight: 487.55 g.mol^{-1}
White solid
Melting point: 73–75 °C
Yield: 70%

^{1}H NMR (500 MHz, CDCl$_3$) δ 10.78 (d, J = 13.0 Hz, 1H), 8.09 (dd, J = 13.0 Hz, J = 2.5 Hz, 1H), 7.75–7.70 (m, 4H), 7.60–7.58 (m, 2H), 7.53–7.49 (m, 2H), 7.44–7.40 (m, 4H), 7.33–7.29 (m, 2H), 7.18–7.15 (m, 2H), 7.12–7.09 (m, 2H), 7.03–7.00 (m, 1H), 3.36 (d, J = 13.5 Hz, 2H), 2.38 (s, 3H).
^{13}C NMR (126 MHz, CDCl$_3$) δ 142.9, 141.4 (d, $J_{C\text{-}P}$ = 3.3 Hz), 141.2, 139.4, 132.4 (d, $J_{C\text{-}P}$ = 2.9 Hz), 131.0 (d, $J_{C\text{-}P}$ = 9.7 Hz), 130.6 (d, $J_{C\text{-}P}$ = 101.0 Hz), 129.8, 129.7, 128.9 (d, $J_{C\text{-}P}$ = 12.2 Hz), 126.9, 122.8, 115.9, 100.2 (d, $J_{C\text{-}P}$ = 5.3 Hz), 29.4 (d, $J_{C\text{-}P}$ = 67.5 Hz), 21.6.
^{31}P NMR (202 MHz, CDCl$_3$) δ 35.78 (s, 1P).
IR (neat): ν = 2926, 1655, 1591, 1498, 1286, 1137, 1085, 735, 659, 527 cm^{-1}.
ESI-HRMS (ESI-TOF): m/z calcd for $C_{28}H_{27}NO_3PS^+$ [M+H]$^+$: 488.1444, found 488.1413.

Compound 8h (*E*)-(3-((4-methoxyphenyl)amino)-2-tosylallyl)diphenylphosphine oxide

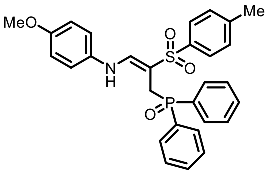

Chemical formula: $C_{29}H_{28}NO_4PS$
Molecular weight: 517.58 g.mol^{-1}
White solid
Melting point: 75–77 °C
Yield: 80%

^{1}H NMR (500 MHz, CDCl$_3$) δ 10.65 (d, J = 13.5 Hz, 1H), 8.00 (dd, J = 13.0 Hz, J = 2.5 Hz, 1H), 7.74–7.70 (m, 4H), 7.59–7.57 (m, 2H), 7.52–7.48 (m, 2H), 7.43–7.39 (m, 4H), 7.16–7.14 (m, 2H), 7.04–7.03 (m, 2H), 6.87–6.84 (m, 2H), 3.77 (s, 3H), 3.36 (d, J = 13.5 Hz, 2H), 2.36 (s, 3H).

^{13}C NMR (126 MHz, CDCl$_3$) δ 155.7, 142.7, 142.2 (d, J_{C-P} = 3.0 Hz), 139.6, 134.8, 132.3 (d, J_{C-P} = 2.9 Hz), 130.9 (d, J_{C-P} = 9.7 Hz), 130.6 (d, J_{C-P} = 101.3 Hz), 129.7, 128.8 (d, J_{C-P} = 12.1 Hz), 126.8, 117.3, 114.9, 98.6 (d, J_{C-P} = 5.3 Hz), 55.6, 29.4 (d, J_{C-P} = 67.8 Hz), 21.5.
^{31}P NMR (121 MHz, CDCl$_3$) δ 35.62 (s, 1P).
IR (neat): ν = 2933, 1652, 1508, 1287, 1245, 1136, 1084, 733, 585 cm^{-1}.
ESI-HRMS (ESI-TOF): m/z calcd for $C_{29}H_{28}NNaO_4PS^+$ [M+Na]$^+$: 540.1369, found 540.1356.

Compound 8i (*E*)-(3-((3-methoxyphenyl)amino)-2-tosylallyl)diphenylphosphine oxide

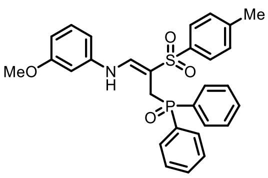

Chemical formula: $C_{29}H_{28}NO_4PS$
Molecular weight: 517.58 g.mol^{-1}
White solid
Melting point: 96–98 °C
Yield: 73%

^{1}H NMR (500 MHz, CDCl$_3$) δ 10.76 (d, *J* = 13.0 Hz, 1H), 8.06 (dd, *J* = 12.5 Hz, *J* = 2.0 Hz, 1H), 7.74–7.69 (m, 4H), 7.59–7.57 (m, 2H), 7.53–7.50 (m, 2H), 7.44–7.40 (m, 4H), 7.20 (t, *J* = 8.0 Hz, 1H), 7.17 (d, *J* = 8.0 Hz, 2H), 6.71 (dd, *J* = 8.0 Hz, *J* = 2.0 Hz, 1H), 6.63 (t, *J* = 2.5 Hz, 1H), 6.57 (dd, *J* = 8.5 Hz, *J* = 2.5 Hz, 1H), 3.81 (s, 3H), 3.36 (d, *J* = 13.5 Hz, 2H), 2.38 (s, 3H).
^{13}C NMR (126 MHz, CDCl$_3$) δ 161.0, 142.9, 142.5, 141.4 (d, J_{C-P} = 3.4 Hz), 139.3, 132.5 (d, J_{C-P} = 2.8 Hz), 131.0 (d, J_{C-P} = 9.7 Hz), 130.5 (d, J_{C-P} = 101.2 Hz), 130.5, 129.8, 128.9 (d, J_{C-P} = 12.1 Hz), 127.0, 108.5, 108.4, 101.8, 100.4 (d, J_{C-P} = 5.3 Hz), 55.5, 29.4 (d, J_{C-P} = 67.5 Hz), 21.6.
^{31}P NMR (202 MHz, CDCl$_3$) δ 35.82 (s, 1P).
IR (neat): ν = 2933, 1658, 1594, 1286, 1137, 1084, 733, 659, 527 cm^{-1}.
ESI-HRMS (ESI-TOF): m/z calcd for $C_{29}H_{28}NNaO_4PS^+$ [M+Na]$^+$: 540.1369, found 540.1379.

Compound 8j (*E*)-diphenyl(3-(*o*-tolylamino)-2-tosylallyl)phosphine oxide

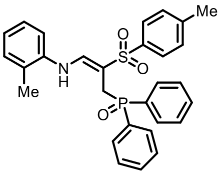

Chemical formula: $C_{29}H_{28}NO_3PS$
Molecular weight: 501.58 g.mol^{-1}
White solid
Melting point: 73–75 °C
Yield: 77%

^{1}H NMR (500 MHz, CDCl$_3$) δ 10.25 (d, *J* = 12.5 Hz, 1H), 8.07 (dd, *J* = 12.5 Hz, *J* = 2.5 Hz, 1H), 7.74–7.70 (m, 4H), 7.61–7.58 (m, 2H), 7.54–7.50 (m, 2H), 7.44–7.40 (m, 4H), 7.20–7.16 (m, 4H), 7.03–6.96 (m, 2H), 3.39 (d, *J* = 13.5 Hz, 2H), 2.48 (s, 3H), 2.39 (s, 3H).
^{13}C NMR (126 MHz, CDCl$_3$) δ 142.9 (d, J_{C-P} = 3.3 Hz), 142.8, 140.3, 139.5, 132.4 (d, J_{C-P} = 2.9 Hz), 131.2, 131.1 (d, J_{C-P} = 9.6 Hz), 130.8 (d, J_{C-P} = 100.7 Hz), 129.8, 128.8 (d, J_{C-P} = 12.1 Hz), 127.6, 127.1, 127.0, 123.3, 115.8, 100.6 (d, J_{C-P} = 5.4 Hz), 29.5 (d, J_{C-P} = 68.2 Hz), 21.6, 18.5.
^{31}P NMR (202 MHz, CDCl$_3$) δ 35.36 (s, 1P).
IR (neat): ν = 2933, 1647, 1289, 1138, 1085, 737 cm^{-1}.
ESI-HRMS (ESI-TOF): m/z calcd for $C_{29}H_{28}NNaO_3PS^+$ [M+Na]$^+$: 524.1420, found 524.1418.

Compound 8k (*E*)-(3-(octylamino)-2-tosylallyl)diphenylphosphine oxide

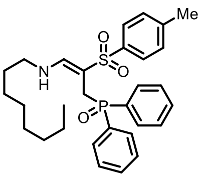

Chemical formula: $C_{30}H_{38}NO_3PS$
Molecular weight: 523.67 g.mol^{-1}
Colorless liquid
Yield: 47%

^{1}H NMR (500 MHz, CDCl$_3$) δ 8.23–8.18 (m, 1H), 7.73–7.68 (m, 4H), 7.55–7.53 (m, 2H), 7.50–7.46 (m, 2H), 7.44–7.38 (m, 5H), 7.14 (d, *J* = 8.0 Hz, 2H), 3.20 (d, *J* = 13.5 Hz, 2H), 3.15 (q, *J* = 7.0 Hz, 2H), 2.35 (s, 3H), 1.52–1.46 (m, 2H), 1.29–1.24 (m, 10H), 0.88 (t, *J* = 7.0 Hz, 3H).
^{13}C NMR (126 MHz, CDCl$_3$) δ 150.0 (d, J_{C-P} = 2.6 Hz), 142.2, 140.5, 132.1 (d, J_{C-P} = 2.9 Hz), 131.1 (d, J_{C-P} = 100.0 Hz), 130.9 (d, J_{C-P} = 9.7 Hz), 129.6, 128.7 (d, J_{C-P} = 12.1 Hz), 126.5, 92.1 (d, J_{C-P} = 4.2 Hz), 49.5, 31.9, 31.2, 29.3, 29.3, 29.2 (d, J_{C-P} = 68.4 Hz), 26.6, 22.7, 21.5, 14.2.
^{31}P NMR (202 MHz, CDCl$_3$) δ 35.13 (s, 1P).
IR (neat): ν = 2926, 2855, 1645, 1283, 1136, 738, 657 cm^{-1}.
ESI-HRMS (ESI-TOF): m/z calcd for $C_{30}H_{39}NO_3PS^+$ [M+H]$^+$: 524.2383, found 524.2382.

Compound 8l (*E*)-(3-(cyclopropylamino)-2-tosylallyl)diphenylphosphine oxide

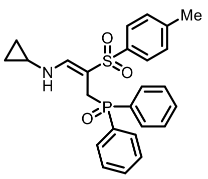

Chemical formula: $C_{25}H_{26}NO_3PS$
Molecular weight: 451.52 g.mol^{-1}
White solid
Melting point: 170–172 °C
Yield: 73%

^{1}H NMR (500 MHz, CDCl$_3$) δ 8.38–8.37 (m, 1H), 7.72–7.67 (m, 4H), 7.58–7.54 (m, 3H), 7.52–7.48 (m, 2H), 7.43–7.39 (m, 4H), 7.16 (d, *J* = 8.5 Hz, 2H), 3.17 (d, *J* = 13.5 Hz, 2H), 2.71–2.67 (m, 1H), 2.37 (s, 3H), 0.69–0.63 (m, 2H), 0.62–0.56 (m, 2H).
^{13}C NMR (126 MHz, CDCl$_3$) δ 150.1 (d, $J_{C\text{-}P}$ = 2.9 Hz), 142.4, 140.3, 132.2 (d, $J_{C\text{-}P}$ = 2.9 Hz), 131.1 (d, $J_{C\text{-}P}$ = 100.2 Hz), 130.9 (d, $J_{C\text{-}P}$ = 9.6 Hz), 129.7, 128.8 (d, $J_{C\text{-}P}$ = 12.1 Hz), 126.7, 94.3 (d, $J_{C\text{-}P}$ = 4.5 Hz), 29.2 (d, $J_{C\text{-}P}$ = 68.3 Hz), 29.2, 21.5, 6.9.
^{31}P NMR (202 MHz, CDCl$_3$) δ 35.03 (s, 1P).
IR (neat): ν = 3197, 3060, 1644, 1284, 1135, 1086, 737, 656, 584 cm^{-1}.
ESI-HRMS (ESI-TOF): m/z calcd for $C_{25}H_{26}NNaO_3PS^+$ [M+Na]$^+$: 474.1263, found 474.1261.

Compound 8m (*E*)-2-(2-((3-(diphenylphosphoryl)-2-tosylprop-1-en-1-yl)amino)ethyl)cyclopentan-1-one

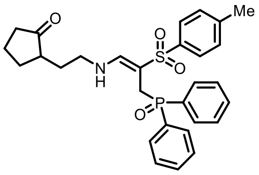

Chemical formula: $C_{29}H_{32}NO_4PS$
Molecular weight: 521.61 g.mol^{-1}
White solid
Melting point: 68–70 °C
Yield: 80%

^{1}H NMR (500 MHz, CDCl$_3$) δ 8.31–8.26 (m, 1H), 7.73–7.68 (m, 4H), 7.57–7.54 (m, 2H), 7.52–7.48 (m, 2H), 7.44–7.39 (m, 5H), 7.17–7.16 (m, 2H), 3.34–3.23 (m, 2H), 3.19 (d, *J* = 13.5 Hz, 2H), 2.37 (s, 3H), 2.33–2.16 (m, 2H), 2.13–1.94 (m, 4H), 1.77–1.68 (m, 1H), 1.49–1.41 (m, 2H).
^{13}C NMR (126 MHz, CDCl$_3$) δ 220.4, 149.8 (d, $J_{C\text{-}P}$ = 2.8 Hz), 142.4, 140.4, 132.3 (d, $J_{C\text{-}P}$ = 2.9 Hz), 131.2 (d, $J_{C\text{-}P}$ = 100.3 Hz), 131.0 (d, $J_{C\text{-}P}$ = 9.6 Hz), 129.7, 128.8 (d, $J_{C\text{-}P}$ = 12.0 Hz), 126.6, 93.2 (d, $J_{C\text{-}P}$ = 4.3 Hz), 47.6, 46.3, 38.0, 31.5, 29.9, 29.2 (d, $J_{C\text{-}P}$ = 68.3 Hz), 21.6, 20.9.
^{31}P NMR (202 MHz, CDCl$_3$) δ 35.23 (s, 1P).
IR (neat): ν = 2956, 1733, 1646, 1282, 1135, 738, 658 cm^{-1}.
ESI-HRMS (ESI-TOF): m/z calcd for $C_{29}H_{32}NNaO_4PS^+$ [M+Na]$^+$: 544.1682, found 544.1680.

Compound 8n (*E*)-(3-((2-(1,3-dioxolan-2-yl)ethyl)amino)-2-tosylallyl)diphenylphosphine oxide

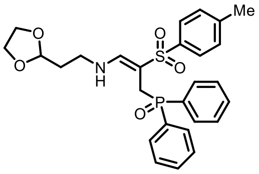

Chemical formula: $C_{27}H_{30}NO_5PS$
Molecular weight: 511.57 g.mol^{-1}
White solid
Melting point: 68–70 °C
Yield: 78%

^{1}H NMR (500 MHz, CDCl$_3$) δ 8.25–8.20 (m, 1H), 7.72–7.67 (m, 4H), 7.55–7.52 (m, 2H), 7.50–7.38 (m, 7H), 7.14–7.12 (m, 2H), 4.82 (t, *J* = 4.5 Hz, 1H), 3.96–3.90 (m, 2H), 3.83–3.76 (m, 2H), 3.33 (q, *J* = 6.5 Hz, 2H), 3.20 (d, *J* = 13.5 Hz, 2H), 2.34 (s, 3H), 1.91–1.87 (m, 2H).
^{13}C NMR (126 MHz, CDCl$_3$) δ 150.0 (d, $J_{C\text{-}P}$ = 2.8 Hz), 142.2, 140.4, 132.1 (d, $J_{C\text{-}P}$ = 2.8 Hz), 131.1 (d, $J_{C\text{-}P}$ = 99.9 Hz), 130.9 (d, $J_{C\text{-}P}$ = 9.5 Hz), 129.6, 128.7 (d, $J_{C\text{-}P}$ = 12.0 Hz), 126.5, 102.1, 92.9 (d, $J_{C\text{-}P}$ = 4.4 Hz), 65.0, 44.5, 35.2, 29.2 (d, $J_{C\text{-}P}$ = 68.4 Hz), 21.5.
^{31}P NMR (202 MHz, CDCl$_3$) δ 35.04 (s, 1P).
IR (neat): ν = 2959, 1645, 1282, 1135, 738, 657, 540 cm^{-1}.
ESI-HRMS (ESI-TOF): m/z calcd for $C_{27}H_{30}NNaO_5PS^+$ [M+Na]$^+$: 534.1475, found 534.1484.

Compound 8o (*E*)-2-(2-((3-(diphenylphosphoryl)-2-tosylprop-1-en-1-yl)amino)ethyl)-3a,4,7,7a-tetrahydro-1*H*-isoindole-1,3(2*H*)-dione

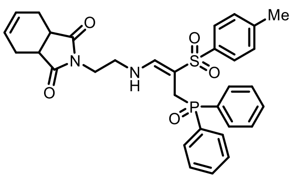

Chemical formula: $C_{32}H_{33}N_2O_5PS$
Molecular weight: 588.66 g.mol^{-1}
White solid
Melting point: 80–82 °C
Yield: 82%

^{1}H NMR (500 MHz, CDCl$_3$) δ 8.36–8.31 (m, 1H), 7.65–7.61 (m, 4H), 7.50–7.44 (m, 4H), 7.39–7.35 (m, 4H), 7.31 (dd, *J* = 13.0 Hz, *J* = 2.0 Hz, 1H), 7.11–7.09 (m, 2H), 5.85–5.83 (m, 2H), 3.61 (t, *J* = 6.0 Hz, 2H), 3.38 (q, *J* = 6.0 Hz, 2H),, 3.19 (d, *J* = 13.5 Hz, 2H),, 3.09–3.07 (m, 2H), 2.55–2.51 (m, 2H), 2.34 (s, 3H), 2.18–2.14 (m, 2H).
^{13}C NMR (126 MHz, CDCl$_3$) δ 180.2, 149.9 (d, J_{C-P} = 2.8 Hz), 142.3, 140.1, 132.1 (d, J_{C-P} = 2.8 Hz), 131.1 (d, J_{C-P} = 100.0 Hz), 130.9 (d, J_{C-P} = 9.6 Hz), 129.6, 128.7 (d, J_{C-P} = 12.0 Hz), 127.9, 126.5, 94.7 (d, J_{C-P} = 4.8 Hz), 46.0, 39.7, 39.1, 29.1 (d, J_{C-P} = 68.8 Hz), 23.5, 21.5.
^{31}P NMR (202 MHz, CDCl$_3$) δ 35.06 (s, 1P).
IR (neat): ν = 2948, 1698, 1644, 1136, 735, 657 cm^{-1}.
ESI-HRMS (ESI-TOF): m/z calcd for $C_{32}H_{33}N_2NaO_5PS^+$ [M+Na]$^+$: 611.1740, found 611.1737.

Compound 8p (*E*)-(3-(benzylamino)-2-((2,4,6-triisopropylphenyl)sulfonyl)allyl)diphenylphosphine oxide

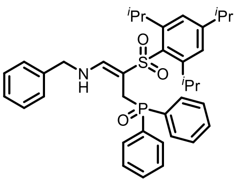

Chemical formula: $C_{37}H_{44}NO_3PS$
Molecular weight: 613.80 g.mol^{-1}
White solid
Melting point: 71–73 °C
Yield: 83%

^{1}H NMR (500 MHz, CDCl$_3$) δ 8.53–8.48 (m, 1H), 7.71–7.67 (m, 4H), 7.52–7.48 (m, 2H), 7.42–7.38 (m, 4H), 7.25–7.22 (m, 4H), 7.13–7.11 (m, 2H), 7.07 (s, 2H), 4.28 (d, *J* = 6.0 Hz, 2H), 4.06 (q, *J* = 6.5 Hz, 2H), 3.29 (d, *J* = 13.5 Hz, 2H), 2.88 (q, *J* = 7.0 Hz, 1H), 1.25 (d, *J* = 7.0 Hz, 6H), 1.08 (d, *J* = 7.0 Hz, 12H).
^{13}C NMR (126 MHz, CDCl$_3$) δ 152.6, 150.9, 147.1 (d, J_{C-P} = 3.2 Hz), 139.0, 133.6, 132.3 (d, J_{C-P} = 2.8 Hz), 131.3 (d, J_{C-P} = 100.4 Hz), 131.0 (d, J_{C-P} = 9.6 Hz), 128.8 (d, J_{C-P} = 12.1 Hz), 128.7, 127.5, 127.3, 124.1, 97.9 (d, J_{C-P} = 4.7 Hz), 52.5, 34.2, 29.4, 28.6 (d, J_{C-P} = 68.2 Hz), 25.0, 23.8.
^{31}P NMR (121 MHz, CDCl$_3$) δ 34.43 (s, 1P).
IR (neat): ν = 2957, 1651, 1284, 1129, 737, 695, 582 cm^{-1}.
ESI-HRMS (ESI-TOF): m/z calcd for $C_{37}H_{44}NNaO_3PS^+$ [M+Na]$^+$: 636.2672, found 636.2663.

Compound 8q (*E*)-(3-(benzylamino)-2-(thiophen-2-ylsulfonyl)allyl)diphenylphosphine oxide

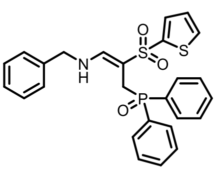

Chemical formula: $C_{26}H_{24}NO_3PS_2$
Molecular weight: 493.58 g.mol^{-1}
White solid
Melting point: 180–182 °C
Yield: 86%

^{1}H NMR (500 MHz, CDCl$_3$) δ 8.76–8.71 (m, 1H), 7.65–7.61 (m, 4H), 7.50–7.42 (m, 3H), 7.37–7.32 (m, 5H), 7.28 (dd, *J* = 4.0 Hz, *J* = 1.5 Hz, 1H), 7.24–7.17 (m, 3H), 7.12–7.10 (m, 2H), 6.86 (dd, *J* = 5.0 Hz, *J* = 3.5 Hz, 1H), 4.29 (d, *J* = 6.0 Hz, 2H), 3.25 (d, *J* = 13.5 Hz, 2H).
^{13}C NMR (126 MHz, CDCl$_3$) δ 150.2 (d, J_{C-P} = 2.6 Hz), 145.7, 138.4, 132.3 (d, J_{C-P} = 2.8 Hz), 131.3, 131.0 (d, J_{C-P} = 9.6 Hz), 130.9 (d, J_{C-P} = 100.5 Hz), 130.8, 128.8 (d, J_{C-P} = 12.0 Hz), 128.8, 127.6, 127.4, 127.3, 94.7 (d, J_{C-P} = 4.0 Hz), 53.0, 29.3 (d, J_{C-P} = 68.2 Hz).
^{31}P NMR (121 MHz, CDCl$_3$) δ 35.09 (s, 1P).
IR (neat): ν = 3058, 1644, 1282, 1197, 1127, 1011, 692, 550 cm^{-1}.
ESI-HRMS (ESI-TOF): m/z calcd for $C_{26}H_{24}NNaO_3PS_2^+$ [M+Na]$^+$: 516.0827, found 516.0825.

Compound 8r (*E*)-(3-(benzylamino)-2-tosylallyl)bis(4-(*tert*-butyl)phenyl)phosphine oxide

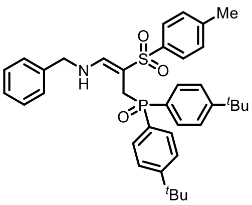

Chemical formula: $C_{37}H_{44}NO_3PS$
Molecular weight: 613.80 g.mol^{-1}
White solid
Melting point: 88–90 °C
Yield: 88%

^{1}H NMR (500 MHz, CDCl$_3$) δ 8.79–8.74 (m, 1H), 7.57–7.50 (m, 6H), 7.42 (dd, *J* = 13.5 Hz, *J* = 2.0 Hz, 1H), 7.35–7.33 (m, 4H), 7.20–7.15 (m, 3H), 7.13–7.10 (m, 2H), 7.08–7.06 (m, 2H), 4.27 (d, *J* = 5.5 Hz, 2H), 3.10 (d, *J* = 14.0 Hz, 2H), 2.30 (s, 3H), 1.23 (s, 18H).
^{13}C NMR (126 MHz, CDCl$_3$) δ 155.5 (d, J_{C-P} = 2.9 Hz), 149.8 (d, J_{C-P} = 2.9 Hz), 142.3, 140.3, 138.7, 130.8 (d, J_{C-P} = 9.9 Hz), 129.6, 128.7, 128.0 (d, J_{C-P} = 101.8 Hz), 127.4, 127.2, 126.8, 125.8 (d, J_{C-P} = 12.3 Hz), 94.1 (d, J_{C-P} = 4.2 Hz), 52.8, 35.1, 31.2, 29.4 (d, J_{C-P} = 67.8 Hz), 21.6.
^{31}P NMR (121 MHz, CDCl$_3$) δ 34.80 (s, 1P).
IR (neat): ν = 2963, 1649, 1093, 611 cm^{-1}.
ESI-HRMS (ESI-TOF): m/z calcd for $C_{37}H_{44}NNaO_3PS^+$ [M+Na]$^+$: 636.2672, found 636.2675.

Compound 8s (*E*)-(3-(benzylamino)-2-tosylallyl)bis(3,5-dimethylphenyl)phosphine oxide

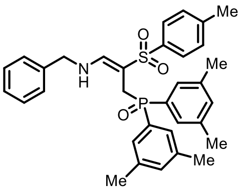

Chemical formula: $C_{33}H_{36}NO_3PS$
Molecular weight: 557.69 g.mol^{-1}
White solid
Melting point: 95–97 °C
Yield: 84%

^{1}H NMR (500 MHz, CDCl$_3$) δ 8.78–8.73 (m, 1H), 7.46–7.43 (m, 3H), 7.26–7.18 (m, 7H), 7.13–7.11 (m, 2H), 7.07–7.03 (m, 4H), 4.29 (d, *J* = 6.0 Hz, 2H), 3.13 (d, *J* = 13.5 Hz, 2H), 2.28 (s, 3H), 2.23 (s, 12H).
^{13}C NMR (126 MHz, CDCl$_3$) δ 150.2 (d, J_{C-P} = 2.9 Hz), 142.2, 140.5, 138.8, 138.5 (d, J_{C-P} = 12.7 Hz), 134.0 (d, J_{C-P} = 2.9 Hz), 131.0 (d, J_{C-P} = 99.4 Hz), 129.5, 128.7, 128.5 (d, J_{C-P} = 9.6 Hz), 127.5, 127.2, 126.5, 94.1 (d, J_{C-P} = 4.4 Hz), 52.8, 29.4 (d, J_{C-P} = 67.7 Hz), 21.5, 21.4.
^{31}P NMR (121 MHz, CDCl$_3$) δ 35.41 (s, 1P).
IR (neat): ν = 2921, 1648, 1283, 1196, 1137, 736, 659 cm^{-1}.
ESI-HRMS (ESI-TOF): m/z calcd for $C_{33}H_{36}NNaO_3PS^+$ [M+Na]$^+$: 580.2046, found 580.2045.

Compound 8t (*E*)-(4-(benzylamino)-3-tosylbut-3-en-2-yl)diphenylphosphine oxide

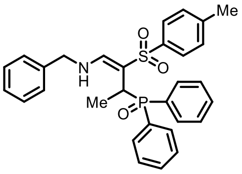

Chemical formula: $C_{30}H_{30}NO_3PS$
Molecular weight: 515.61 g.mol^{-1}
White solid
Melting point: 90–92 °C
Yield: 83%

^{1}H NMR (500 MHz, CDCl$_3$) δ 8.69–8.64 (m, 1H), 7.77–7.73 (m, 2H), 7.67–7.63 (m, 2H), 7.57 (d, *J* = 8.5 Hz, 2H), 7.51–7.41 (m, 5H), 7.33–7.27 (m, 5H), 7.20–7.16 (m, 4H), 4.36 (t, *J* = 5.5 Hz, 2H), 3.57–3.50 (m, 1H), 2.39 (s, 3H), 1.06–1.02 (m, 3H).
^{13}C NMR (126 MHz, CDCl$_3$) δ 148.6, 142.4, 140.7, 138.8, 131.9 (d, J_{C-P} = 16.5 Hz), 131.1 (d, J_{C-P} = 9.3 Hz), 130.9 (d, J_{C-P} = 8.9 Hz), 129.3 (d, J_{C-P} = 108.2 Hz), 129.0 (d, J_{C-P} = 11.3 Hz), 128.5 (d, J_{C-P} = 12.0 Hz), 127.6, 127.3, 127.0, 100.5, 53.1, 32.6 (d, J_{C-P} = 67.0 Hz), 21.6, 11.3.
^{31}P NMR (162 MHz, CDCl$_3$) δ 38.40 (s, 1P).
IR (neat): ν = 3034, 2922, 1640, 1077, 663, 540, 452 cm^{-1}.
ESI-HRMS (ESI-TOF): m/z calcd for $C_{30}H_{30}NNaO_3PS^+$ [M+Na]$^+$: 538.1585, found 538.1592.

Compound 8u (*E*)-(3-(benzylamino)-1-phenyl-2-tosylallyl)diphenylphosphine oxide

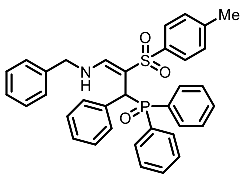

Chemical formula: $C_{35}H_{32}NO_3PS$
Molecular weight: 577.68 g.mol^{-1}
White solid
Melting point: 87–89 °C
Yield: 42%

^{1}H NMR (500 MHz, CDCl$_3$) δ 9.35–9.30 (m, 1H), 7.95–7.91 (m, 2H), 7.51–7.49 (m, 2H), 7.43–7.39 (m, 2H), 7.34–7.14 (m, 10H), 7.01–6.99 (m, 2H), 6.85–6.82 (m, 1H), 6.74–6.69 (m, 4H), 6.56–6.54 (m, 2H), 4.68 (d, *J* = 14.5 Hz, 1H), 4.36–4.21 (m, 2H), 2.11 (s, 3H).
^{13}C NMR (126 MHz, CDCl$_3$) δ 149.1 (d, $J_{C\text{-}P}$ = 4.2 Hz), 141.9, 140.1, 138.6, 134.8 (d, $J_{C\text{-}P}$ = 4.5 Hz), 132.3 (d, $J_{C\text{-}P}$ = 2.8 Hz), 131.9 (d, $J_{C\text{-}P}$ = 2.9 Hz), 131.5 (d, $J_{C\text{-}P}$ = 9.1 Hz), 131.3 (d, $J_{C\text{-}P}$ = 9.5 Hz), 129.9 (d, $J_{C\text{-}P}$ = 4.5 Hz), 128.8 (d, $J_{C\text{-}P}$ = 10.2 Hz), 128.6 (d, $J_{C\text{-}P}$ = 92.6 Hz), 127.9 (d, $J_{C\text{-}P}$ = 102.2 Hz), 127.6 (d, $J_{C\text{-}P}$ = 2.1 Hz), 127.3, 127.0, 126.5 (d, $J_{C\text{-}P}$ = 2.6 Hz), 101.1, 53.2, 46.0 (d, $J_{C\text{-}P}$ = 61.5 Hz), 21.3.
^{31}P NMR (121 MHz, CDCl$_3$) δ 35.83 (s, 1P).
IR (neat): ν = 3205, 3058, 1646, 1438, 1283, 1139, 1081, 697, 561 cm^{-1}.
ESI-HRMS (ESI-TOF): m/z calcd for $C_{35}H_{32}KNO_3PS^+$ $[M+K]^+$: 616.1464, found 616.1478.

Compound 9 (1*E*,5*E*)-*N*^{1},*N*^{6}-dibenzyl-2,5-ditosylhexa-1,5-diene-1,6-diamine

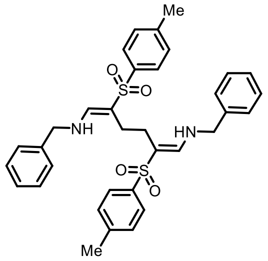

Chemical formula: $C_{34}H_{36}N_2O_4S_2$
Molecular weight: 600.79 g.mol^{-1}
White solid
Melting point: 110–112 °C
Yield: 5%

^{1}H NMR (500 MHz, CDCl$_3$) δ 7.63–7.61 (m, 4H), 7.40–7.29 (m, 12H), 7.23–7.21 (m, 4H), 6.51–6.46 (m, 2H), 4.32 (d, *J* = 6.0 Hz, 4H), 2.38 (s, 6H), 2.26 (s, 4H).
^{13}C NMR (126 MHz, CDCl$_3$) δ 148.0, 142.9, 138.7, 138.5, 129.6, 128.8, 127.7, 127.7, 127.3, 101.5, 52.2, 24.4, 21.6.
IR (neat): ν = 3334, 1640, 1273, 1131, 1080, 726, 664, 571 cm^{-1}.
ESI-HRMS (ESI-TOF): m/z calcd for $C_{34}H_{37}N_2O_4S_2^+$ $[M+H]^+$: 601.2189, found 601.2222.

Compound TEMPO-3a 2,2,6,6-Tetramethylpiperidin-1-yl diphenylphosphinate

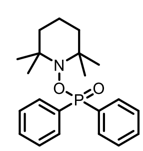

Chemical formula: $C_{21}H_{28}NO_2P$
Molecular weight: 357.43 g.mol^{-1}
Light yellow solid
Yield: 11%

^{1}H NMR (500 MHz, CDCl$_3$) δ 7.88–7.83 (m, 4H), 7.49–7.40 (m, 6H), 1.70–1.41 (m, 6H), 1.38–0.85 (m, 12H).
^{13}C NMR (126 MHz, CDCl$_3$) δ 134.0 (d, $J_{C\text{-}P}$ = 135.3 Hz), 131.8 (d, $J_{C\text{-}P}$ = 9.5 Hz), 131.7 (d, $J_{C\text{-}P}$ = 2.8 Hz), 128.4 (d, $J_{C\text{-}P}$ = 13.0 Hz), 61.7, 61.7, 40.2, 17.0.
^{31}P NMR (202 MHz, CDCl$_3$) δ 33.71 (s, 1P).
ESI-HRMS (ESI-TOF): m/z calcd for $C_{21}H_{29}NO_2P^+$ $[M+H]^+$: 358.1930, found 358.1924.

15. Copies of ^{1}H NMR, ^{13}C NMR, ^{19}F NMR and ^{31}P NMR

1b – ^{1}H NMR (500 MHz, $CDCl_3$)

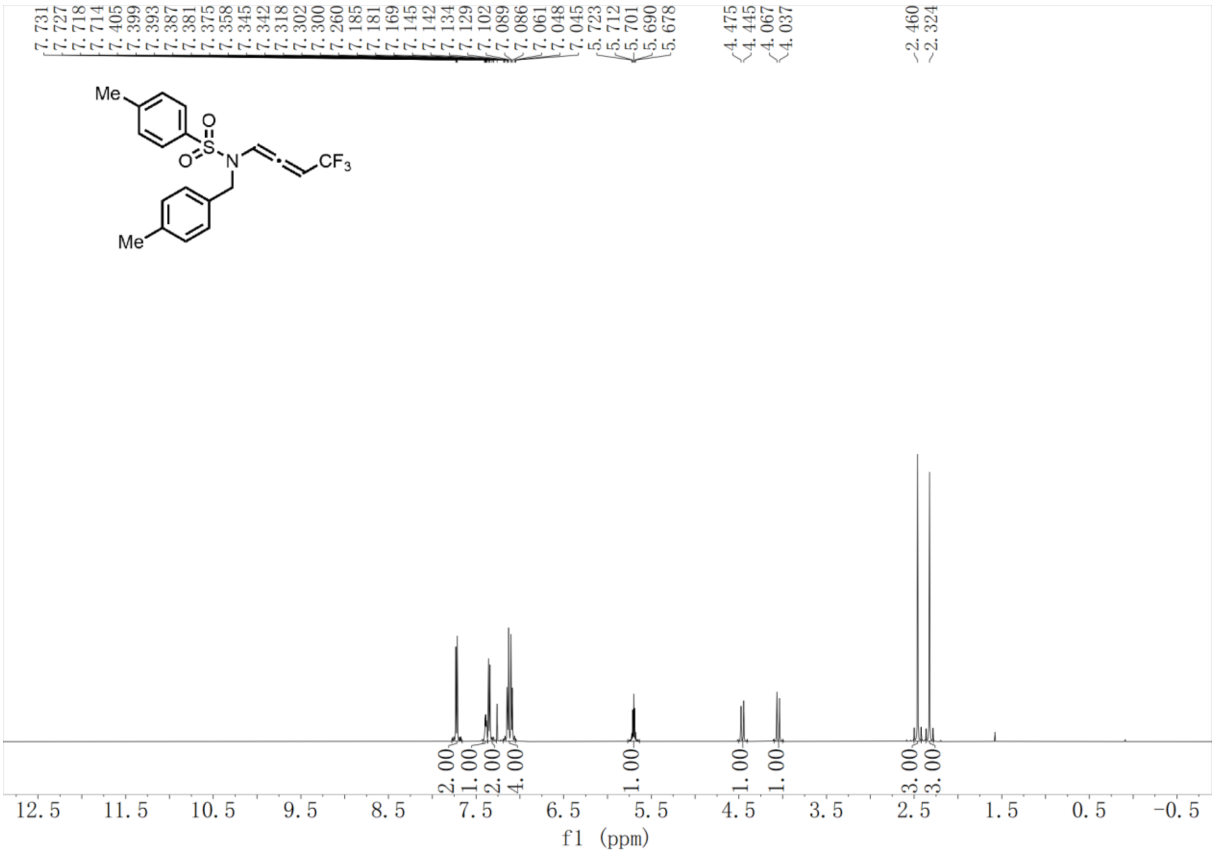

1b – ^{13}C NMR (126 MHz, $CDCl_3$)

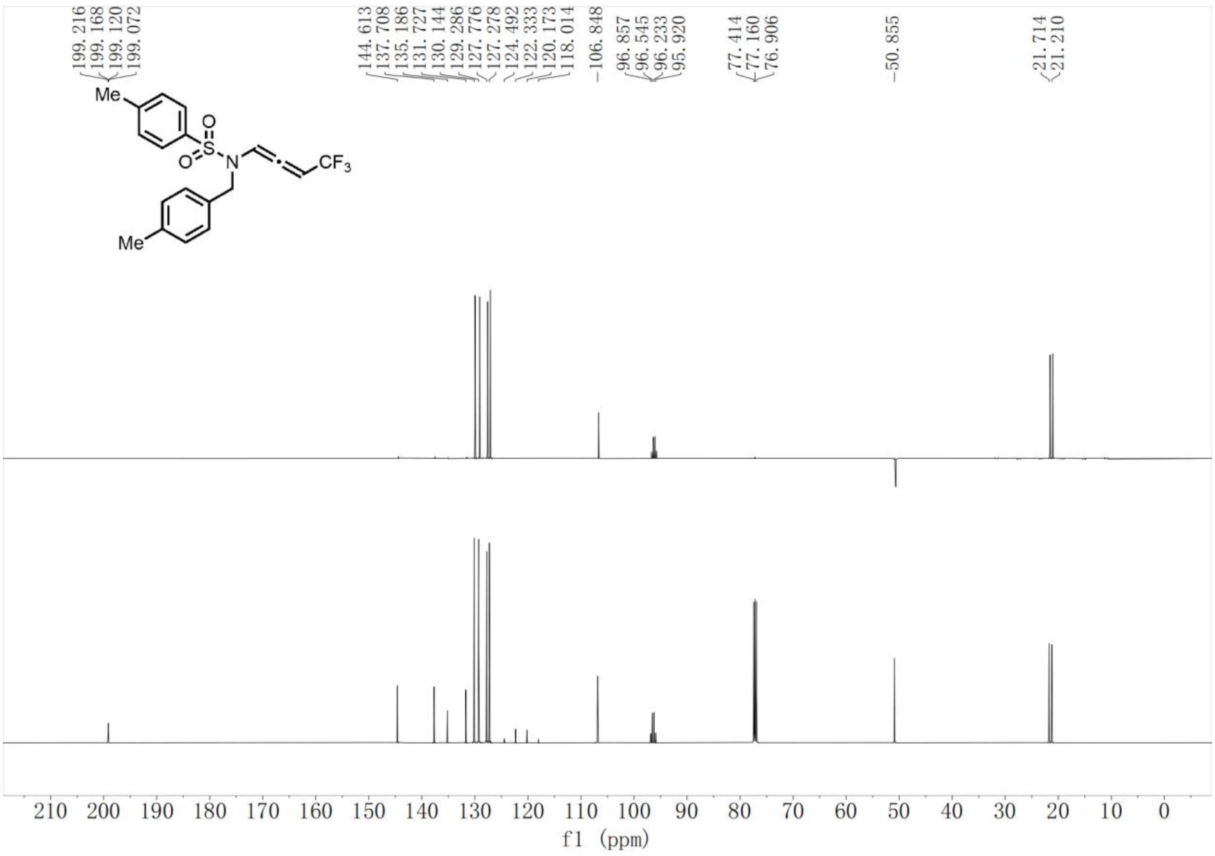

1b – ^{19}F NMR (282 MHz, $CDCl_3$)

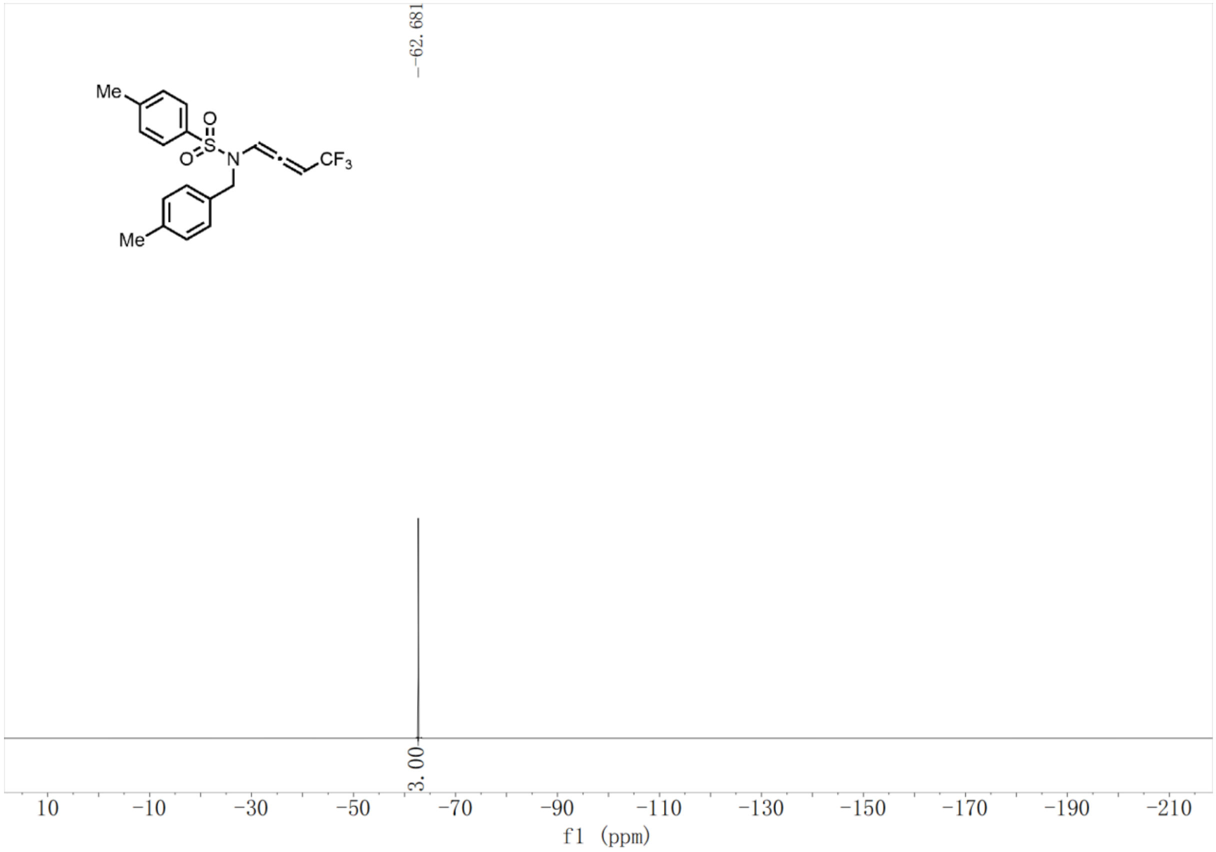

1c – ^{1}H NMR (500 MHz, $CDCl_3$)

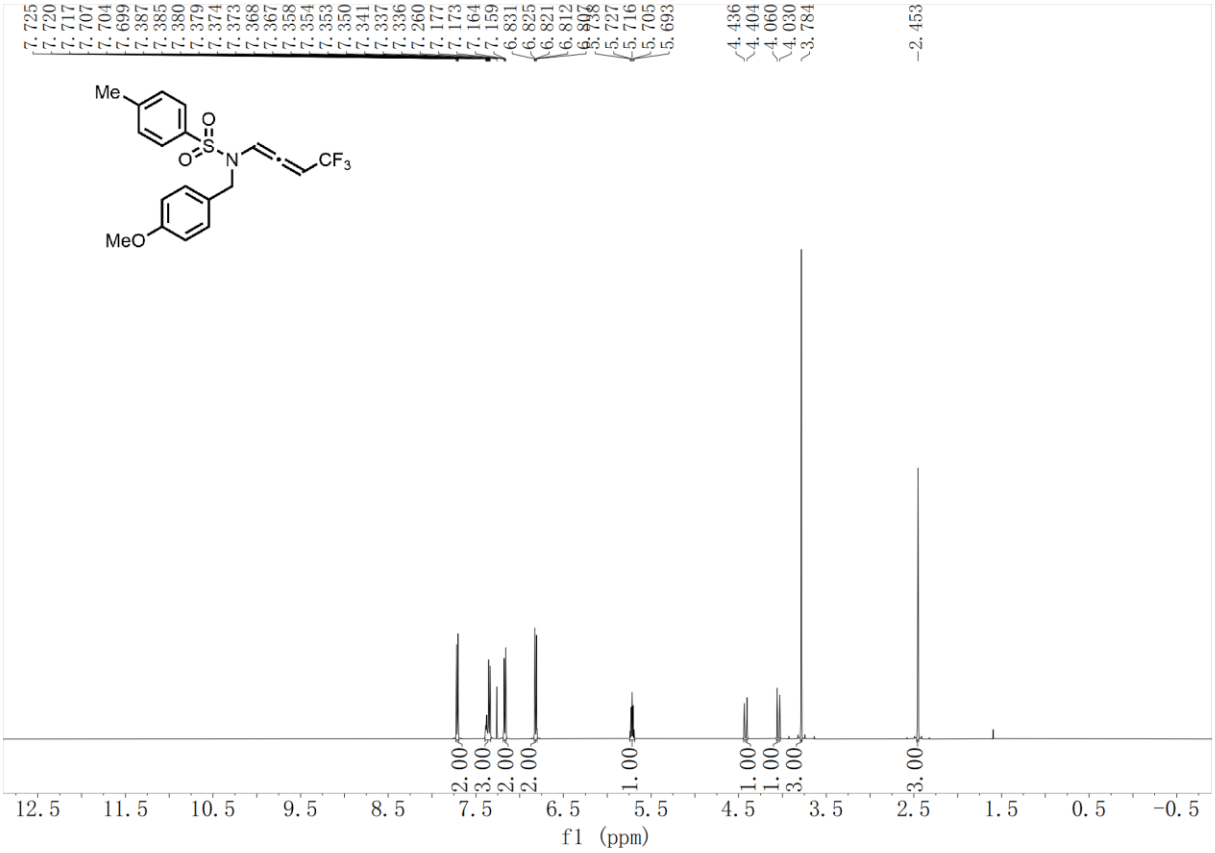

1c – ^{13}C NMR (126 MHz, $CDCl_3$)

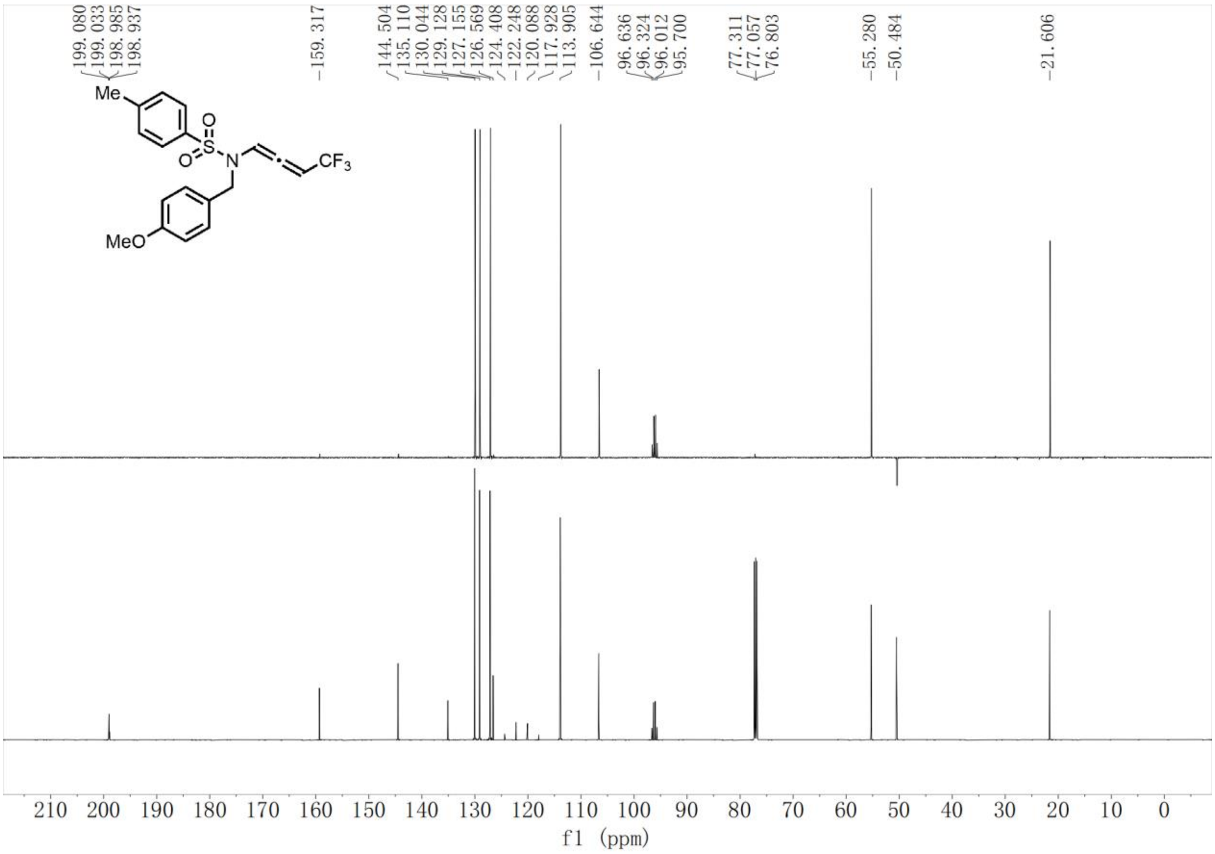

1c – ^{19}F NMR (282 MHz, $CDCl_3$)

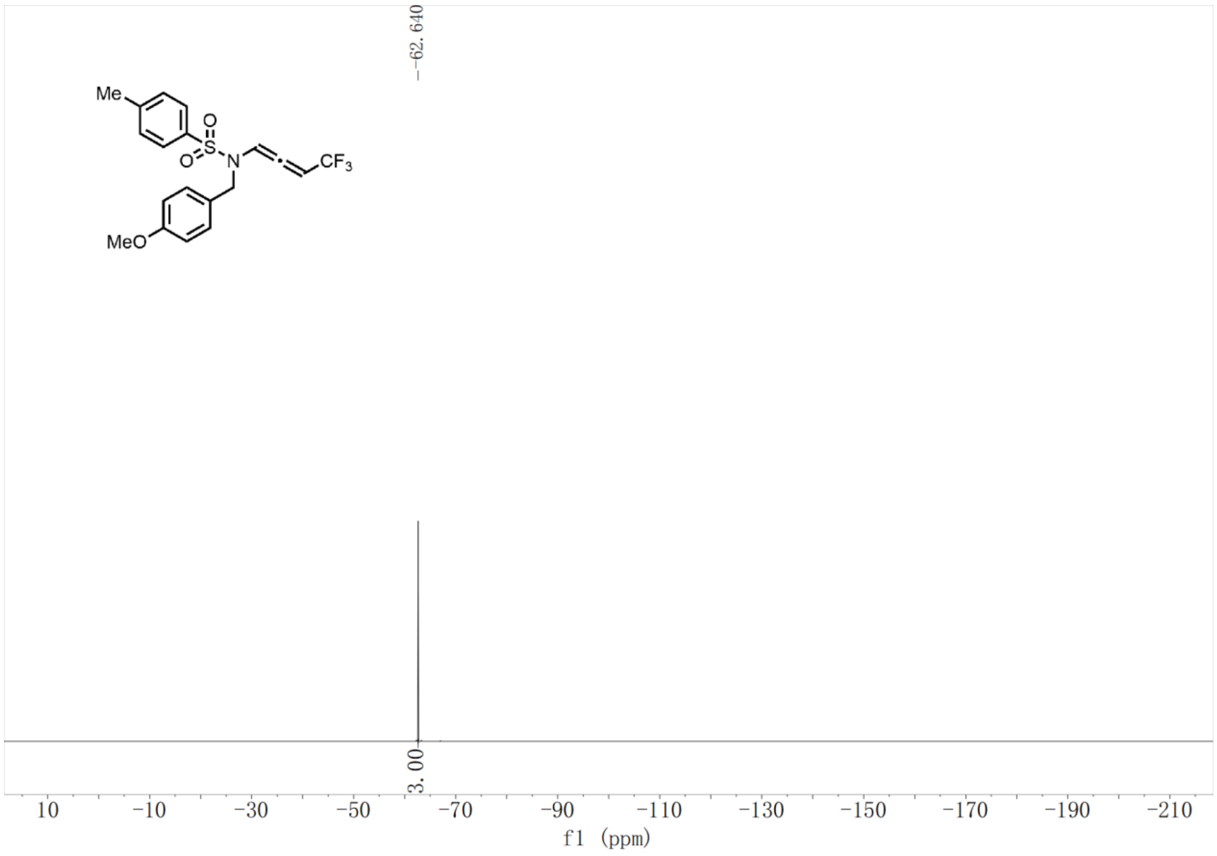

1d – ^{1}H NMR (500 MHz, $CDCl_3$)

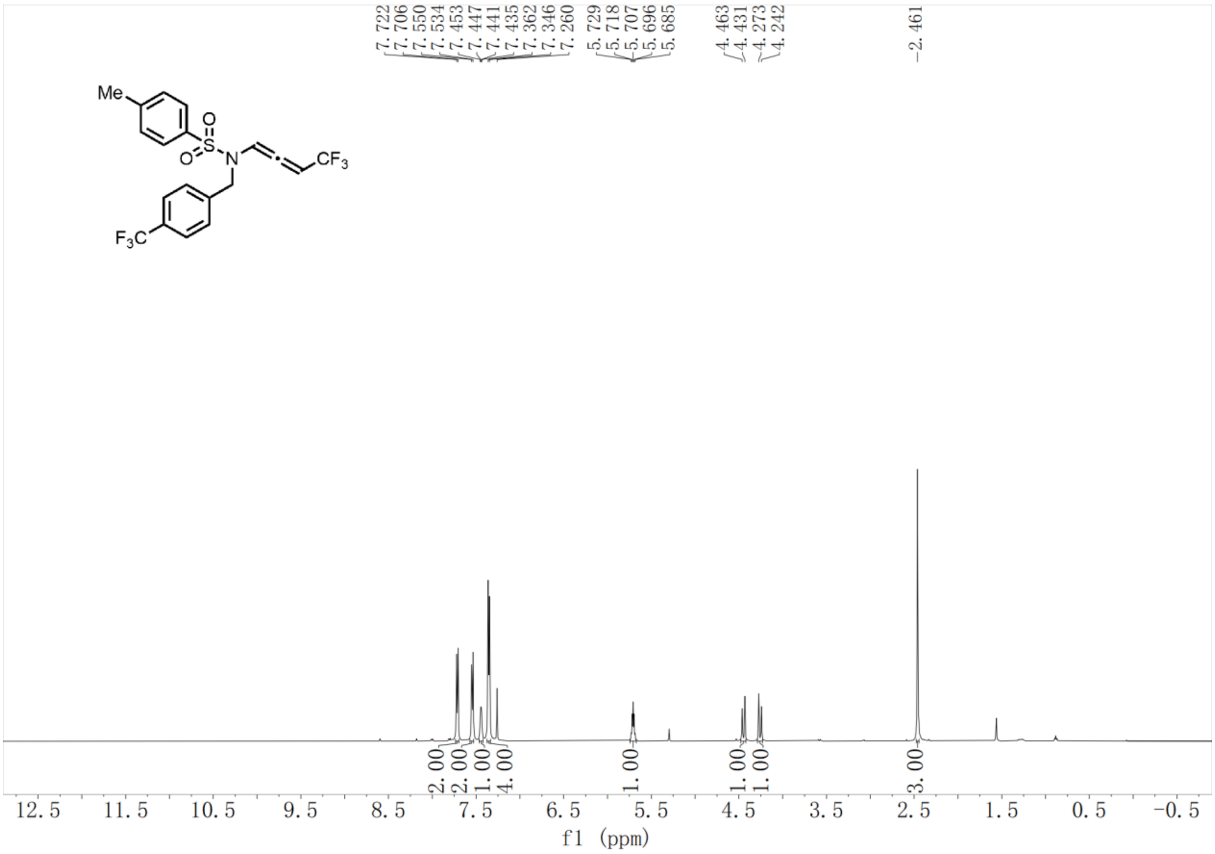

1d – ^{13}C NMR (126 MHz, $CDCl_3$)

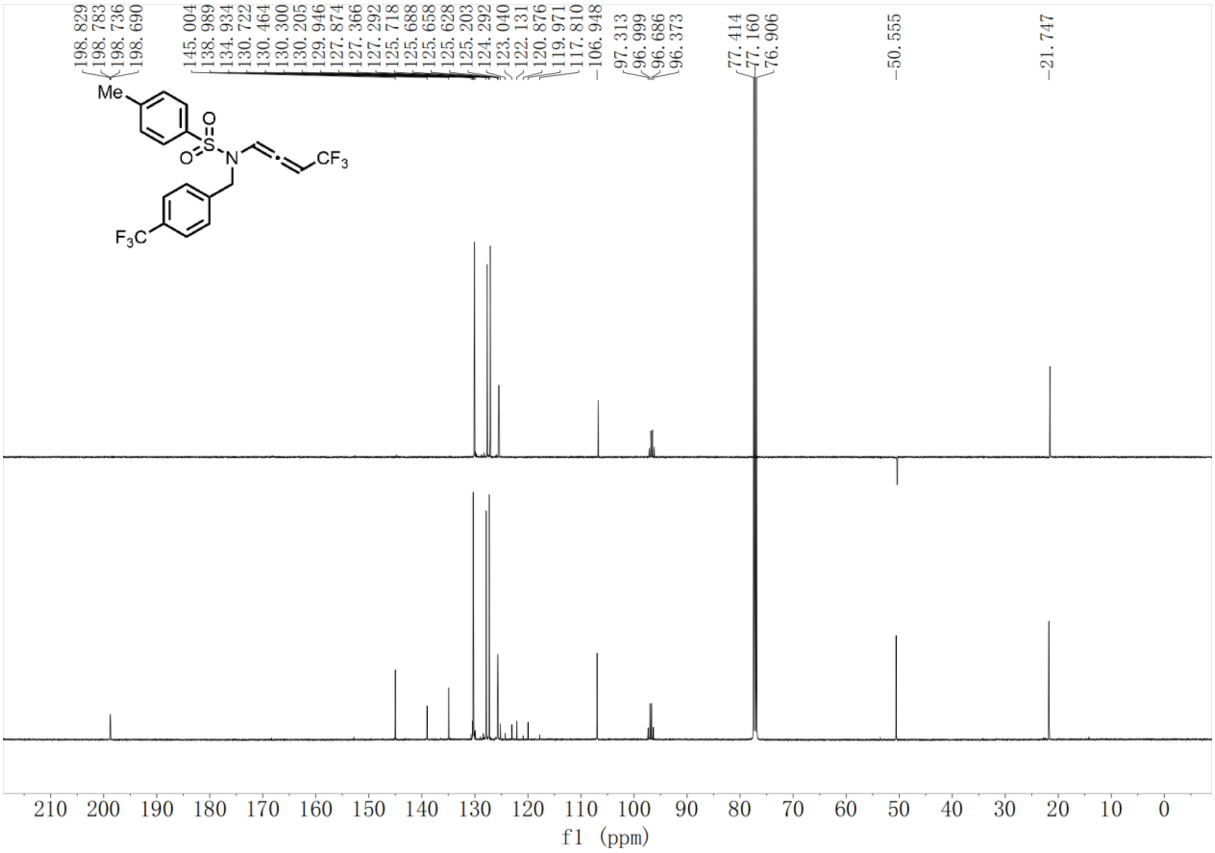

1d – ^{19}F NMR (471 MHz, $CDCl_3$)

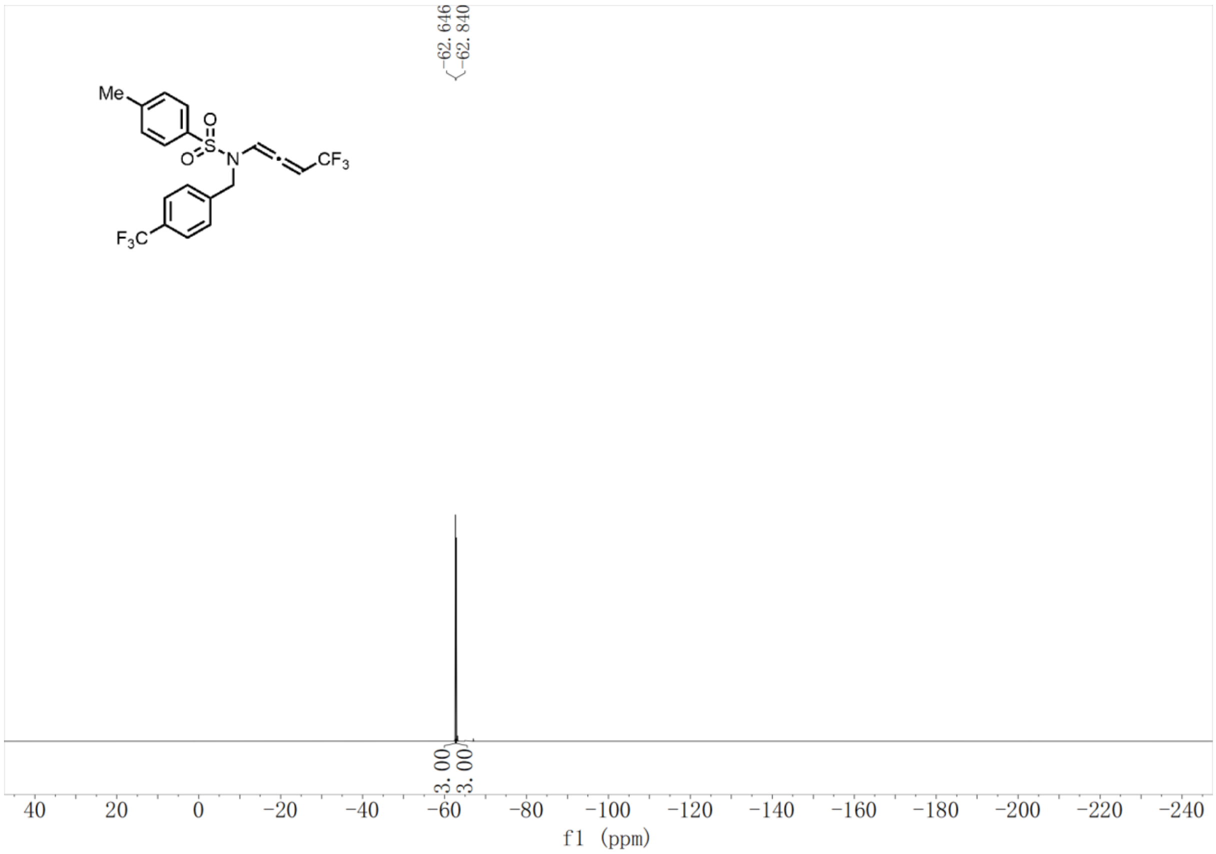

1e – ^{1}H NMR (500 MHz, C_6D_6)

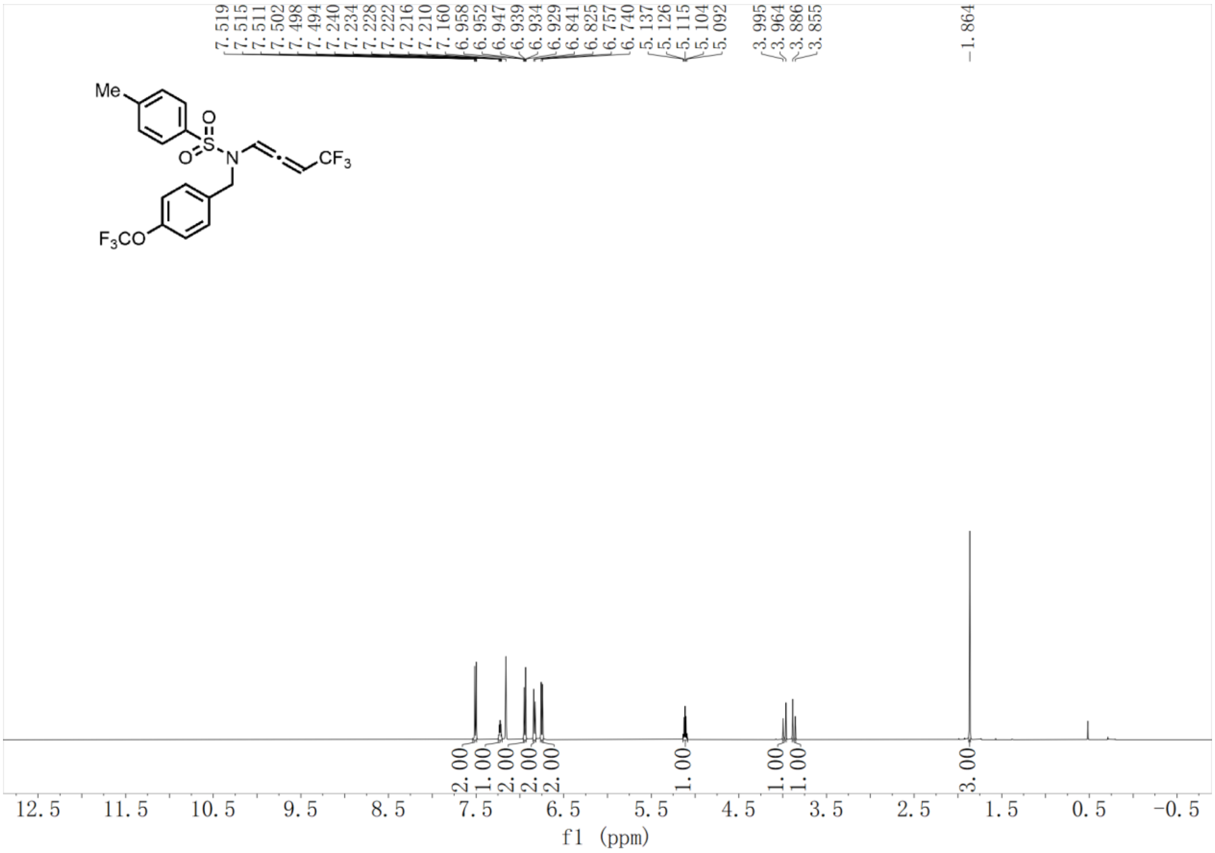

1e – ^{13}C NMR (126 MHz, C_6D_6)

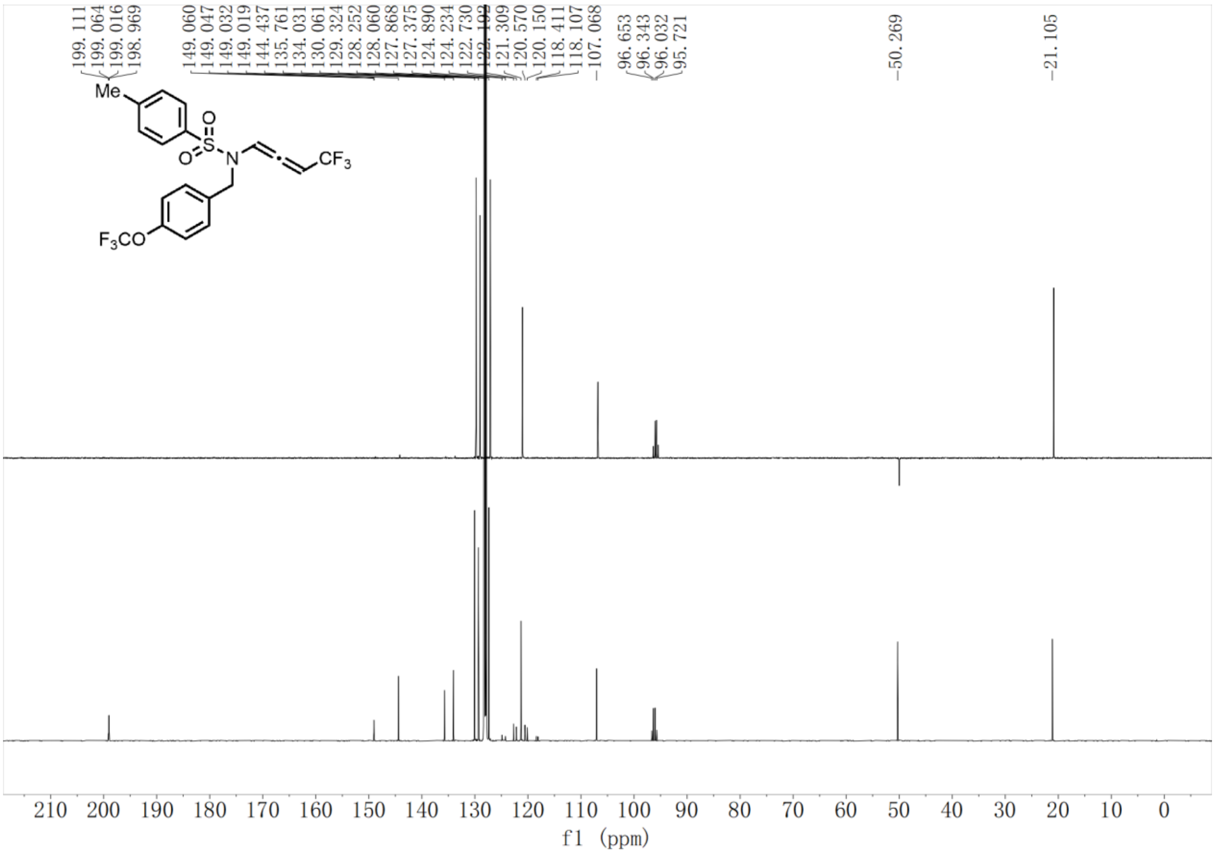

1e – ^{19}F NMR (282 MHz, C_6D_6)

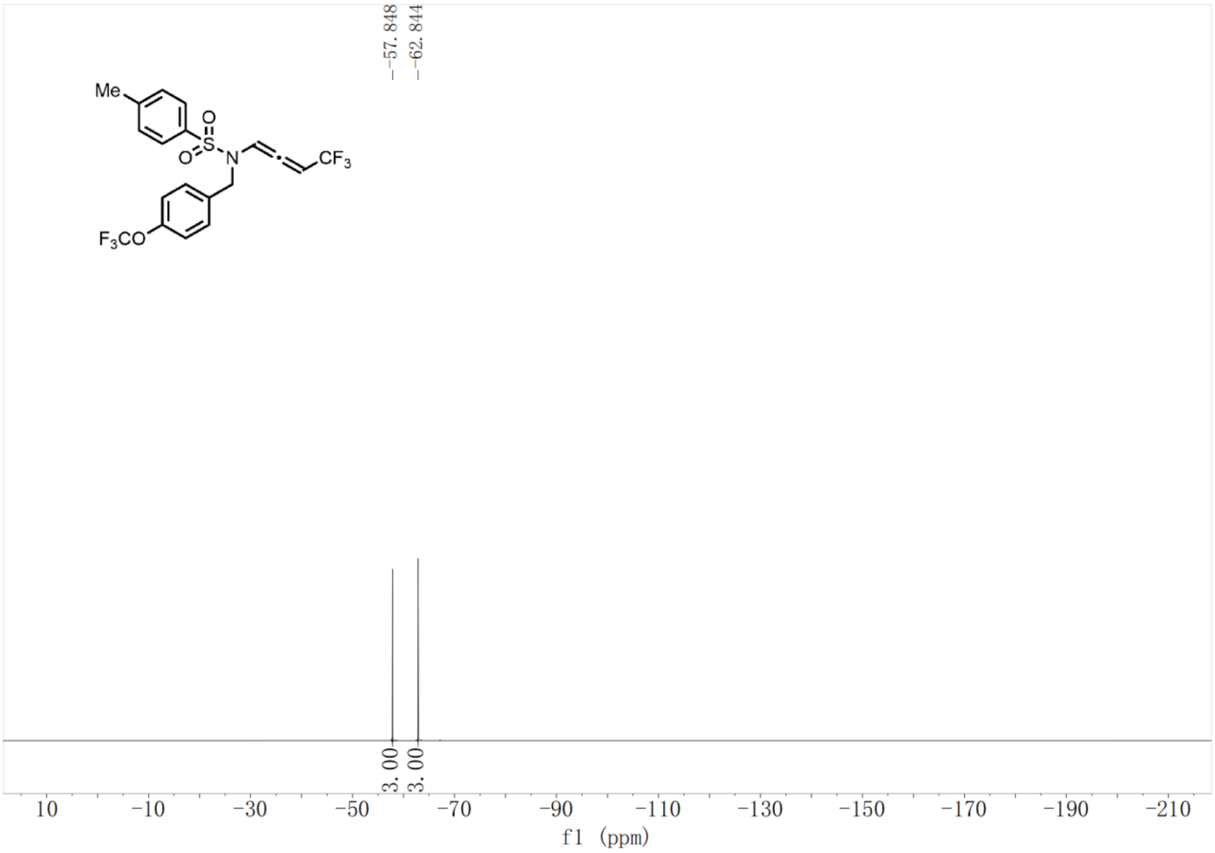

1g – ^{1}H NMR (500 MHz, $CDCl_3$)

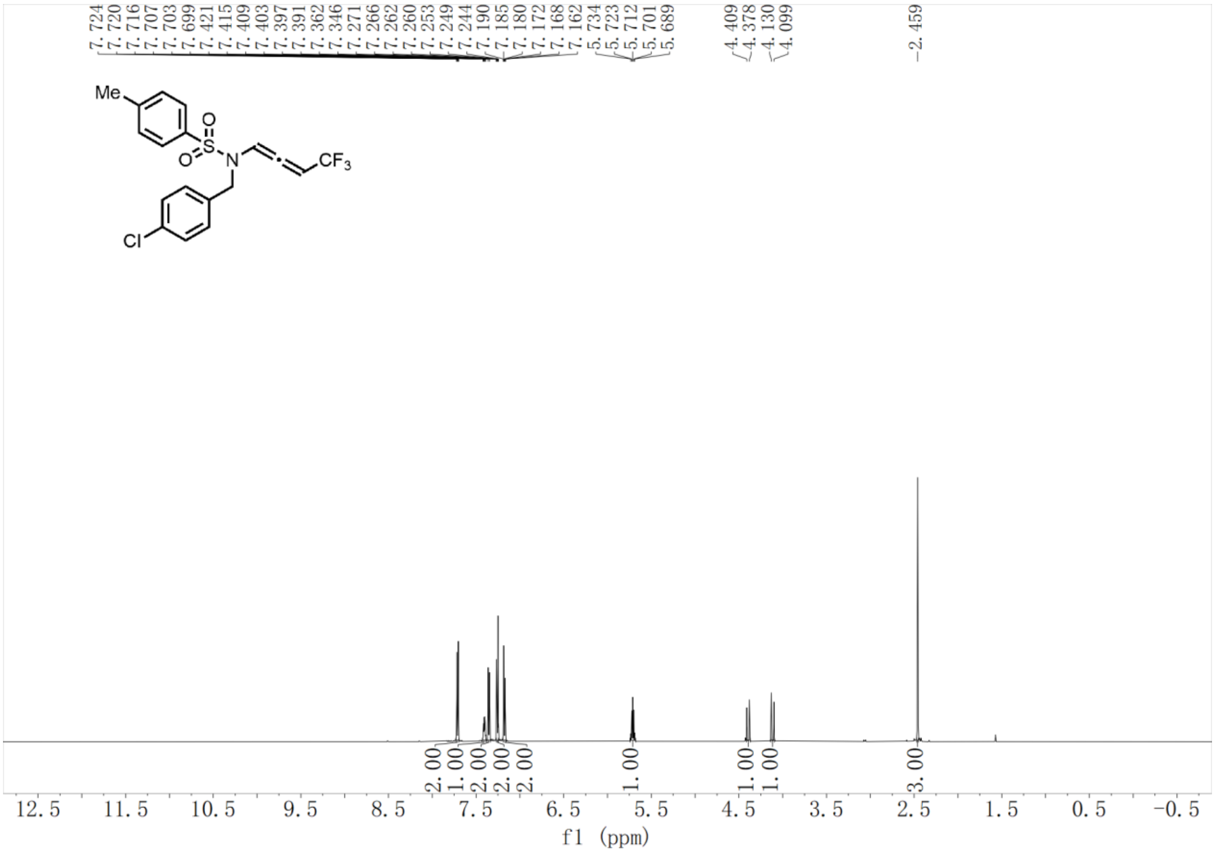

1g – ^{13}C NMR (126 MHz, $CDCl_3$)

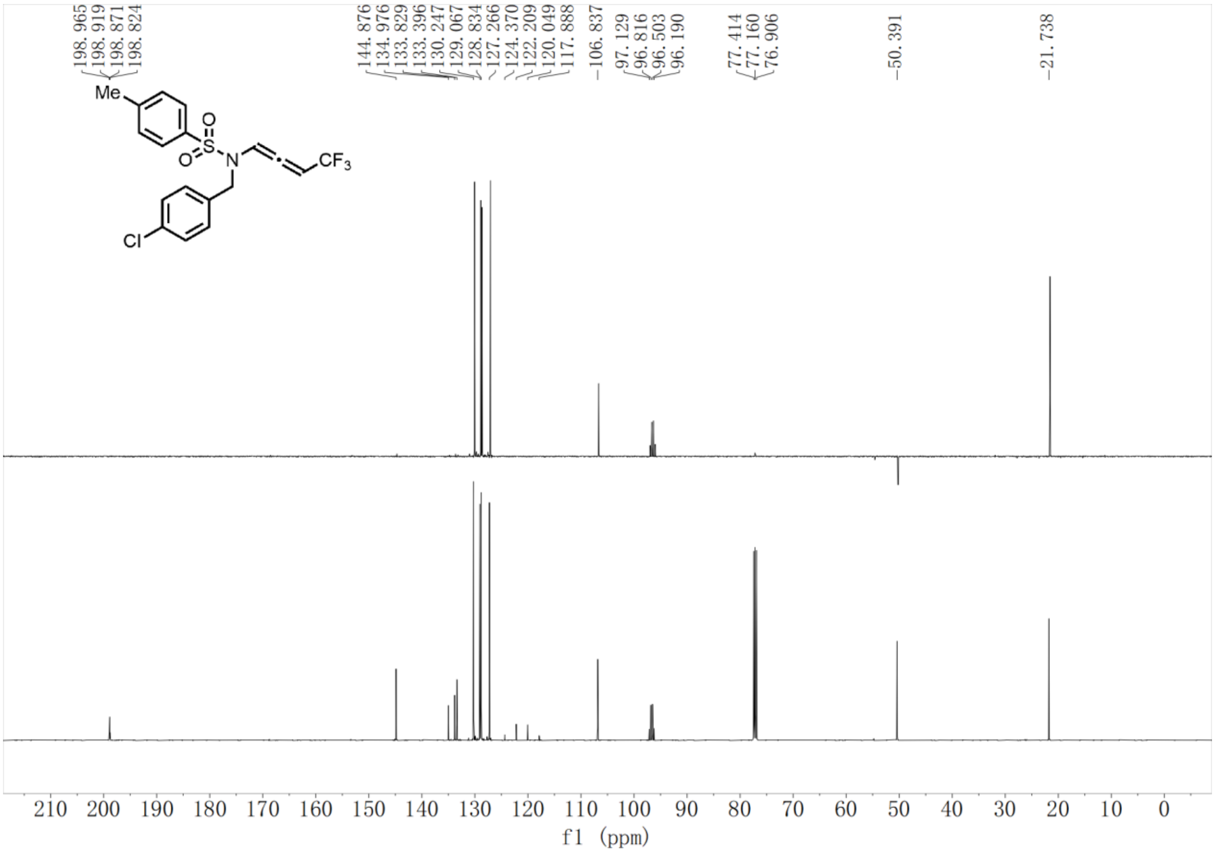

1g – ^{19}F NMR (282 MHz, $CDCl_3$)

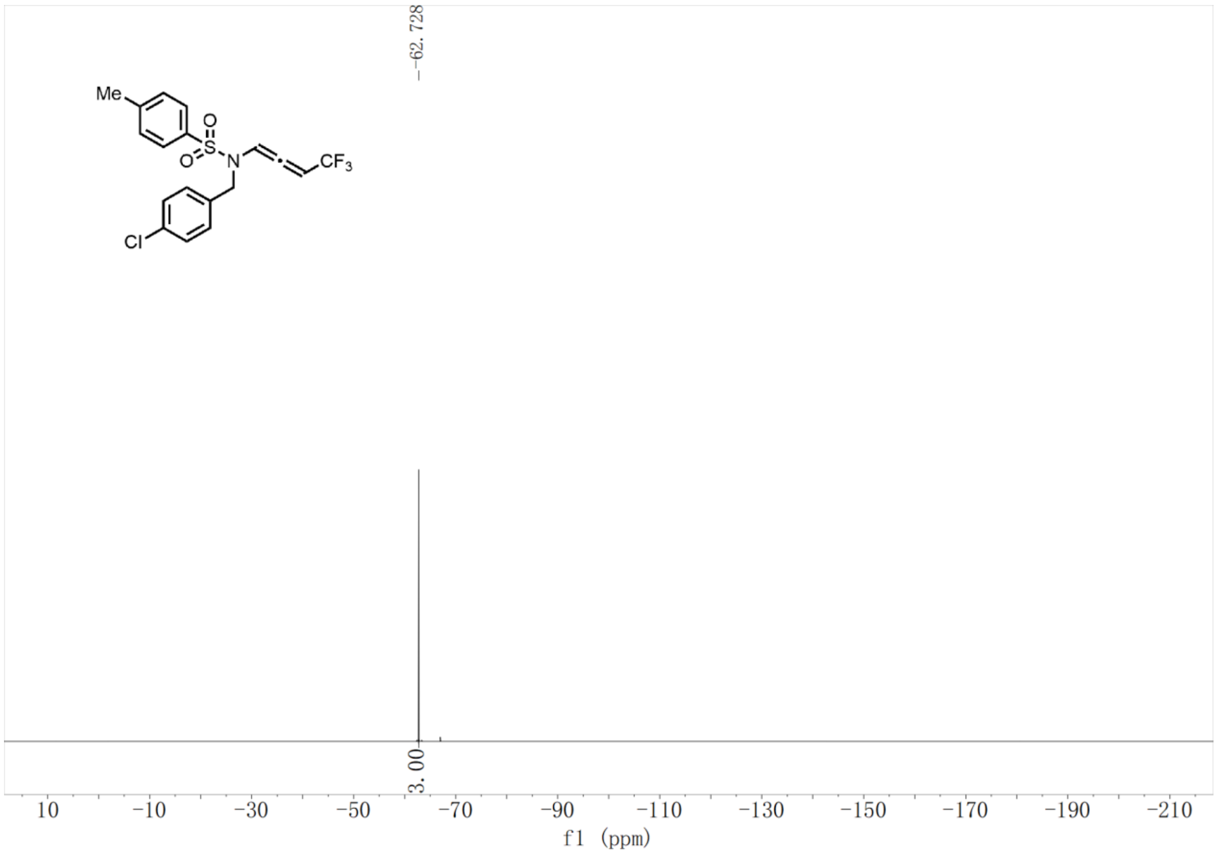

1h – ^{1}H NMR (500 MHz, $CDCl_3$)

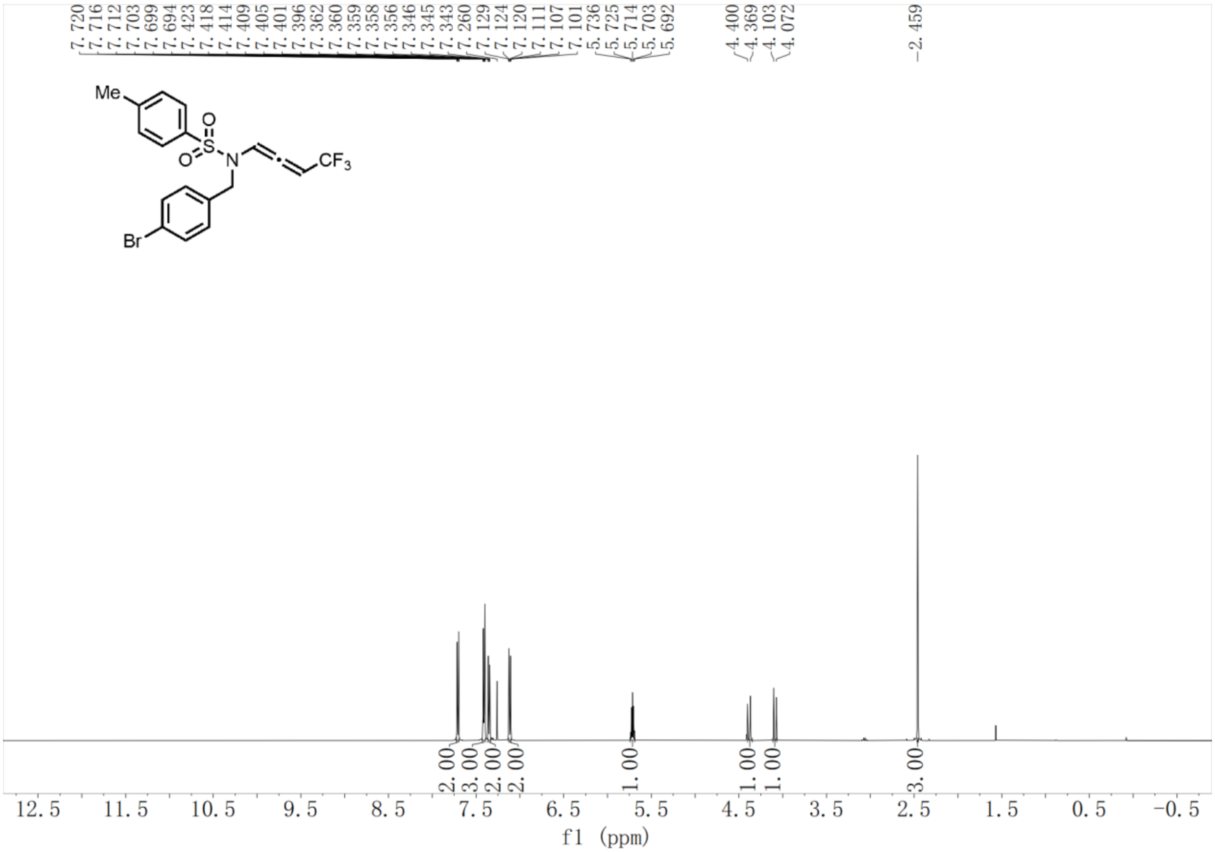

1h – ^{13}C NMR (126 MHz, $CDCl_3$)

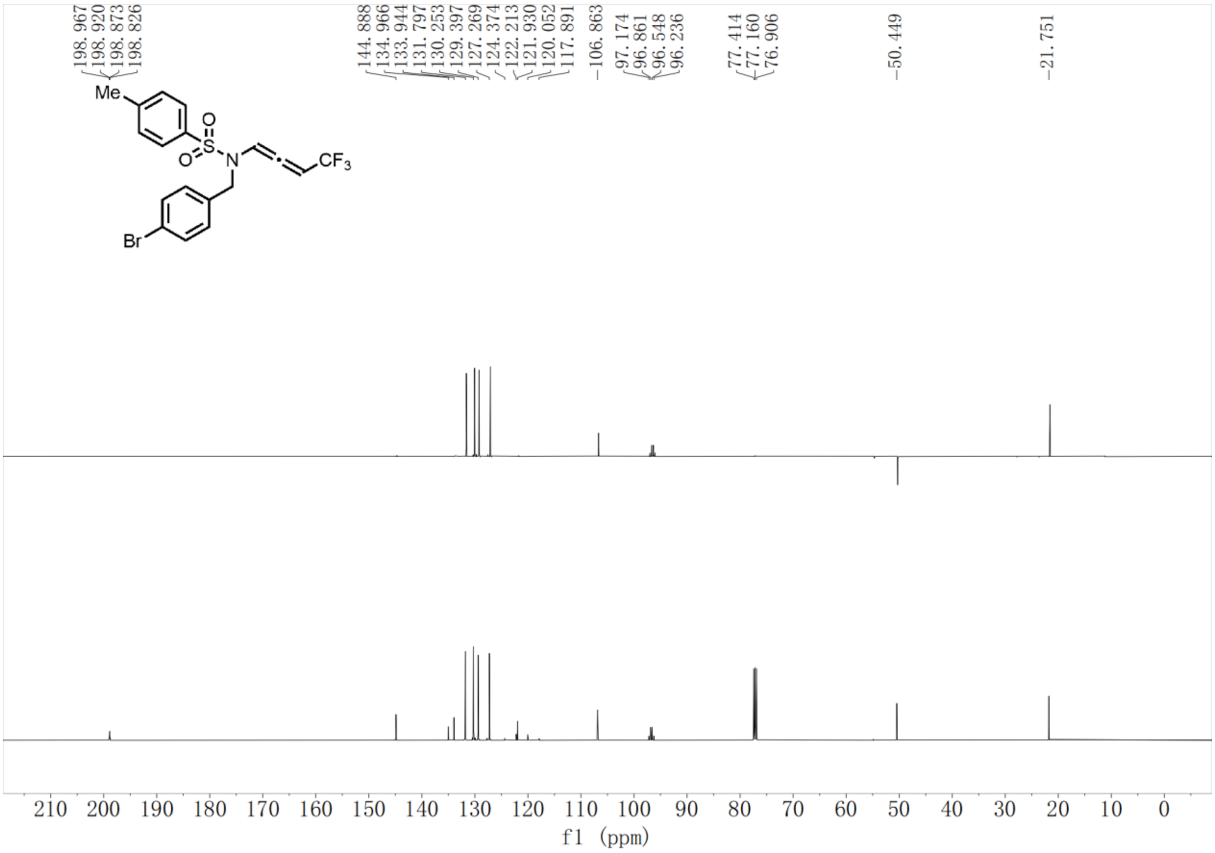

1h – ^{19}F NMR (282 MHz, $CDCl_3$)

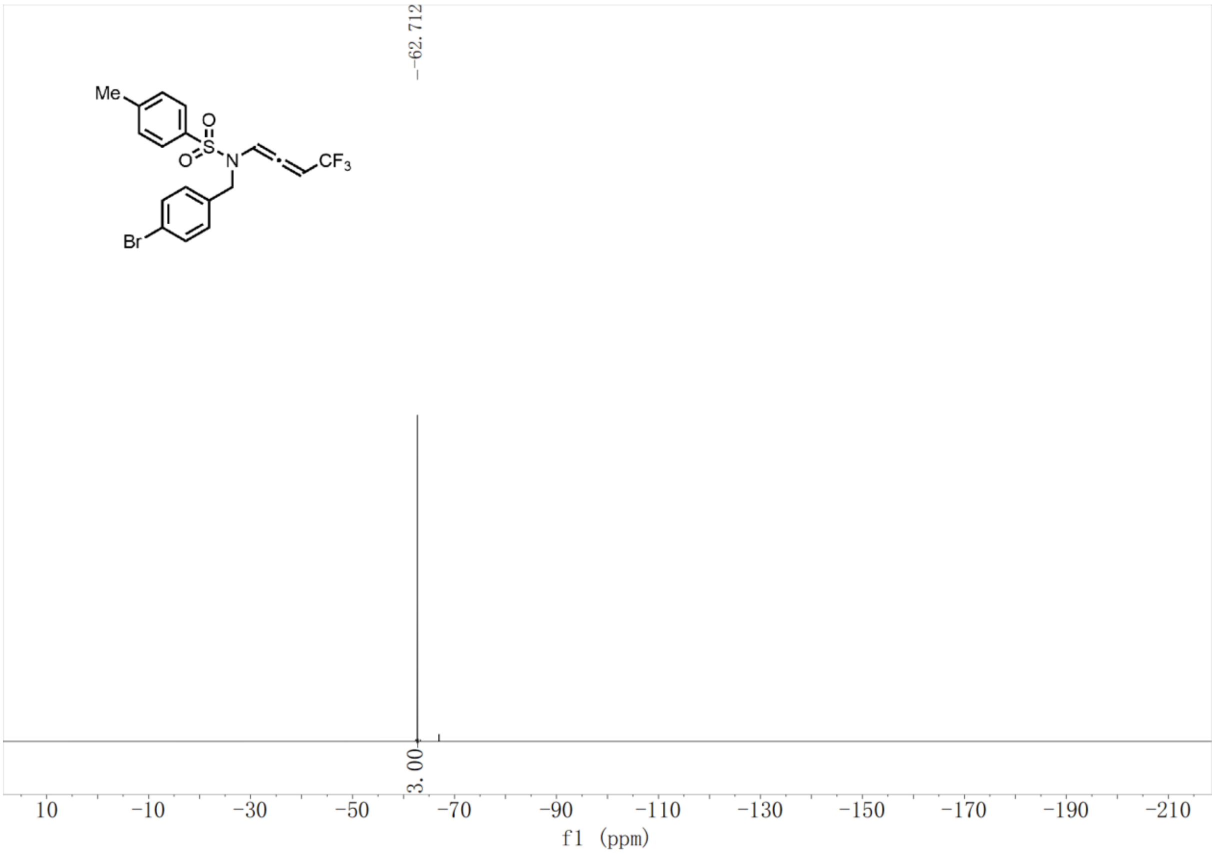

1i – ^{1}H NMR (500 MHz, $CDCl_3$)

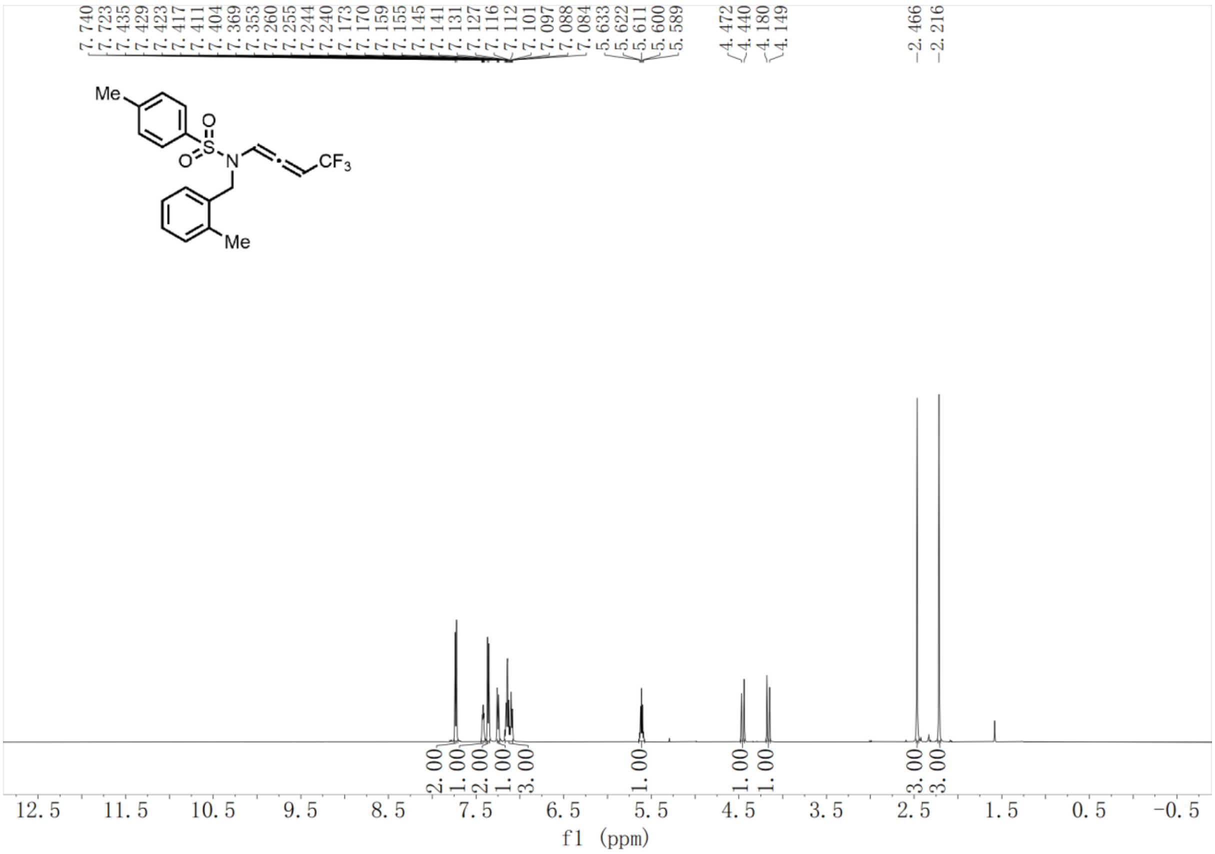

1i – ^{13}C NMR (126 MHz, $CDCl_3$)

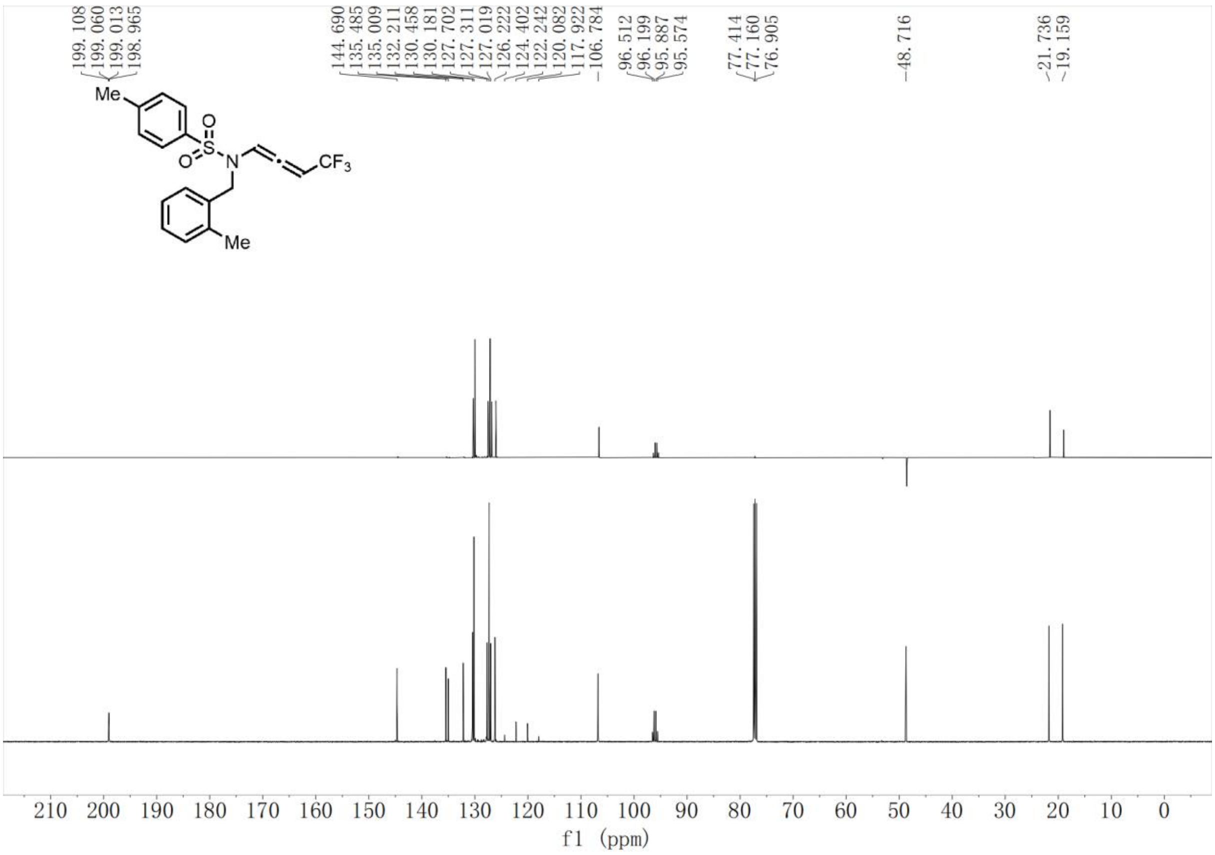

1i – ^{19}F NMR (471 MHz, $CDCl_3$)

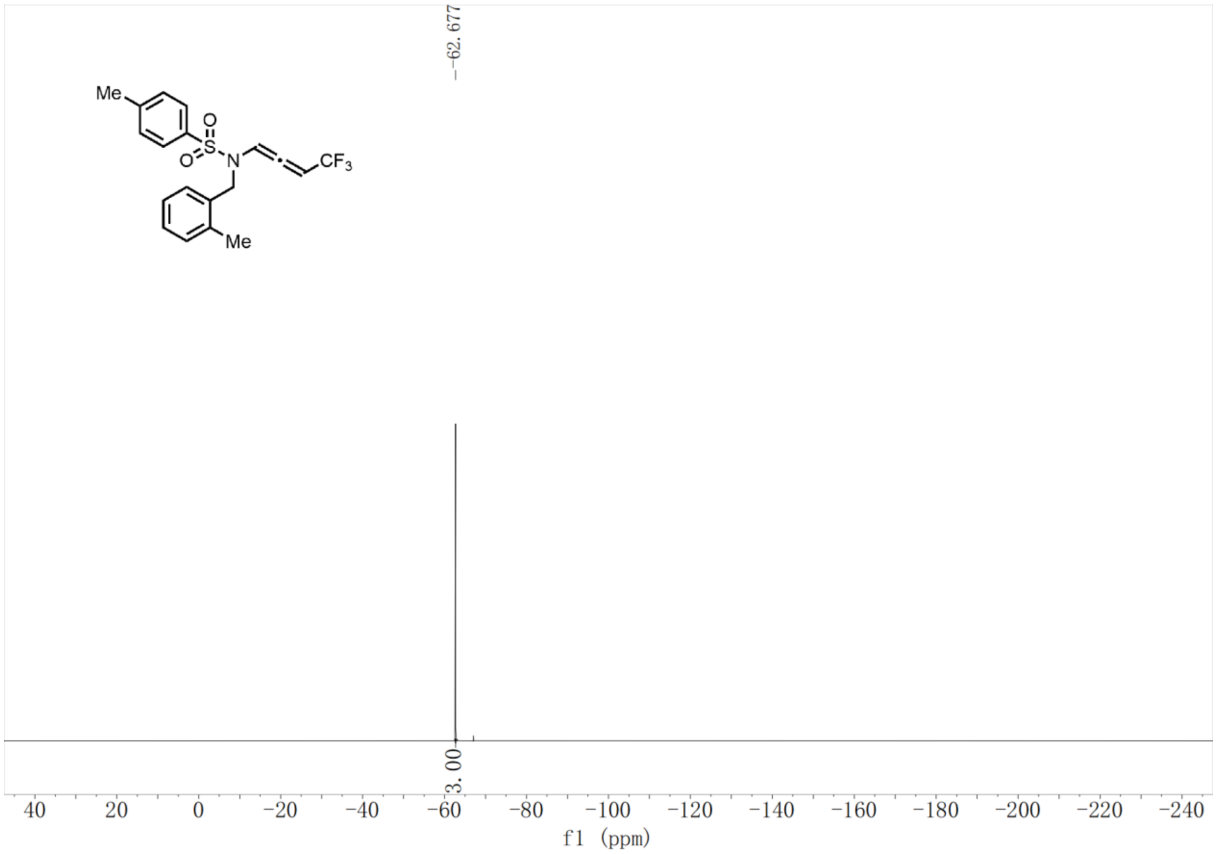

1j – ^{1}H NMR (300 MHz, $CDCl_3$)

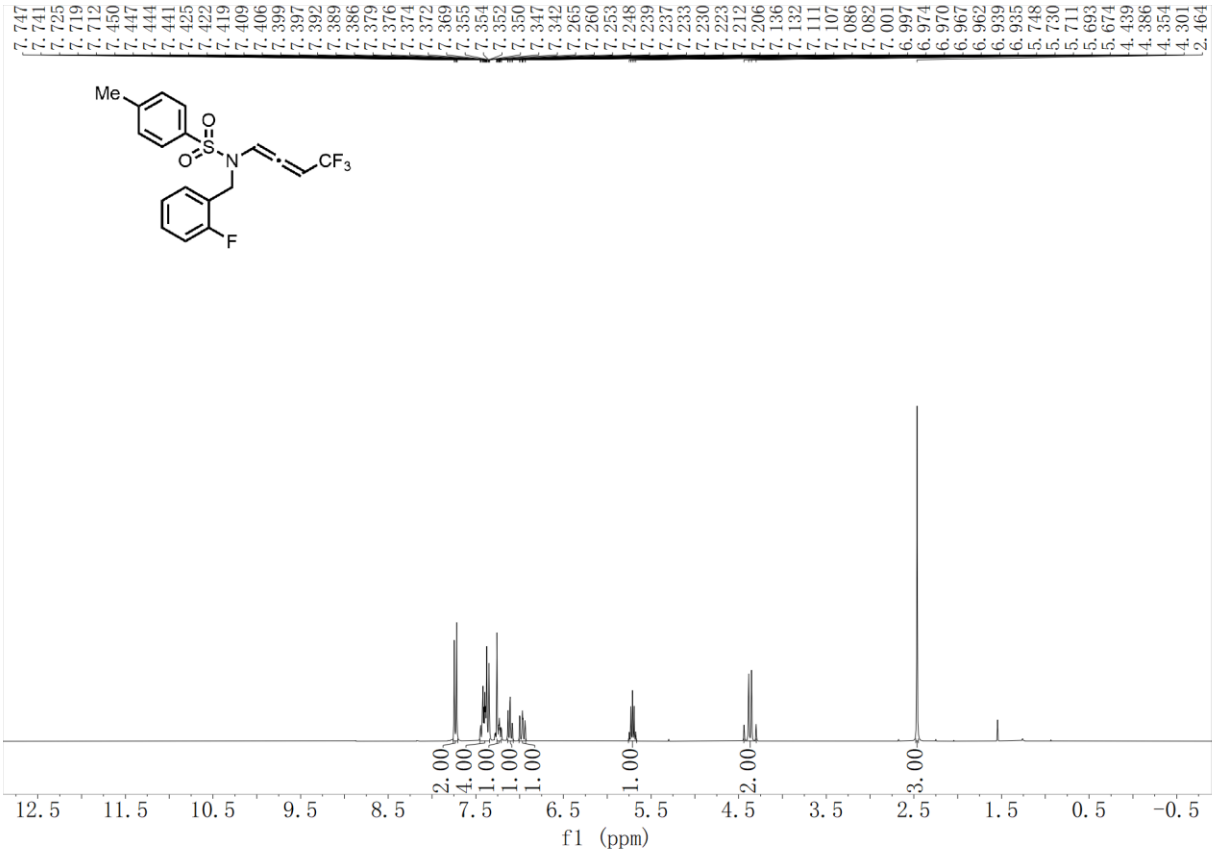

1j – ^{13}C NMR (126 MHz, $CDCl_3$)

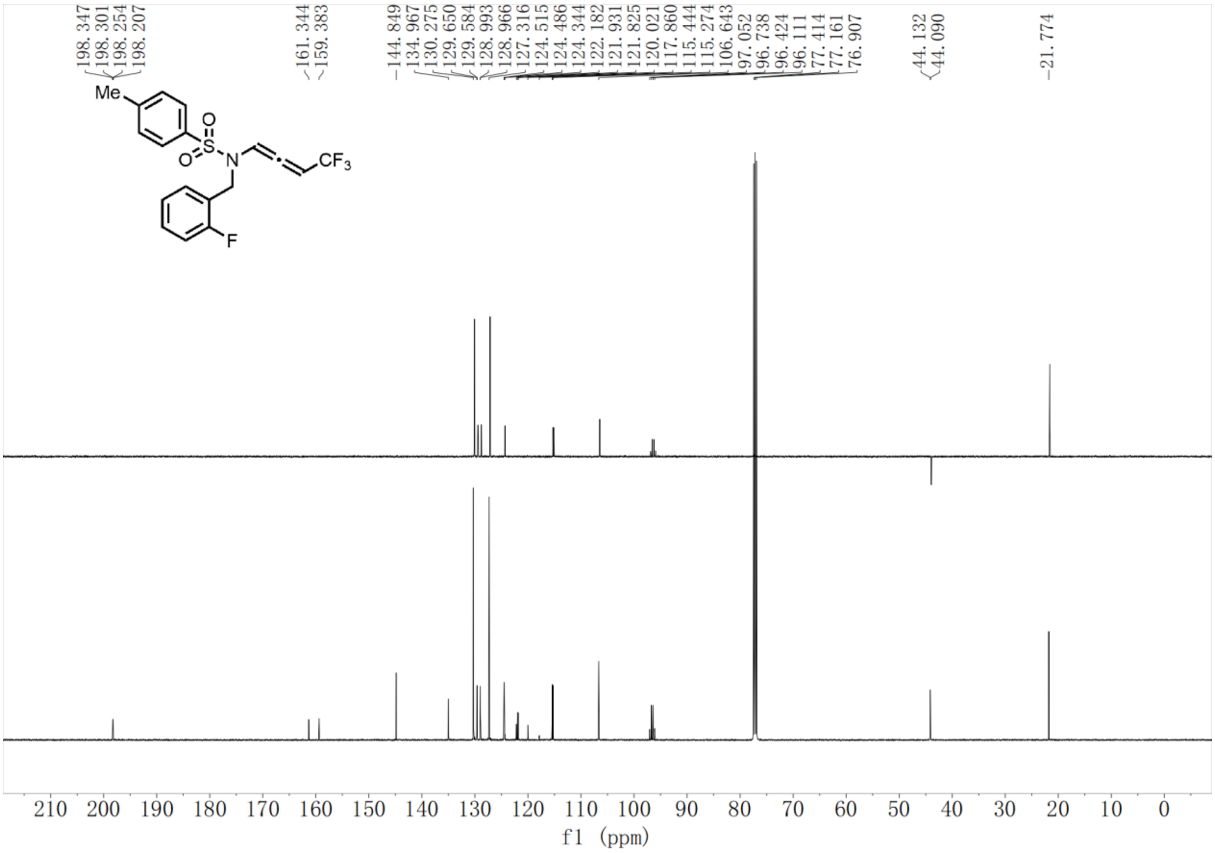

1j – ^{19}F NMR (282 MHz, $CDCl_3$)

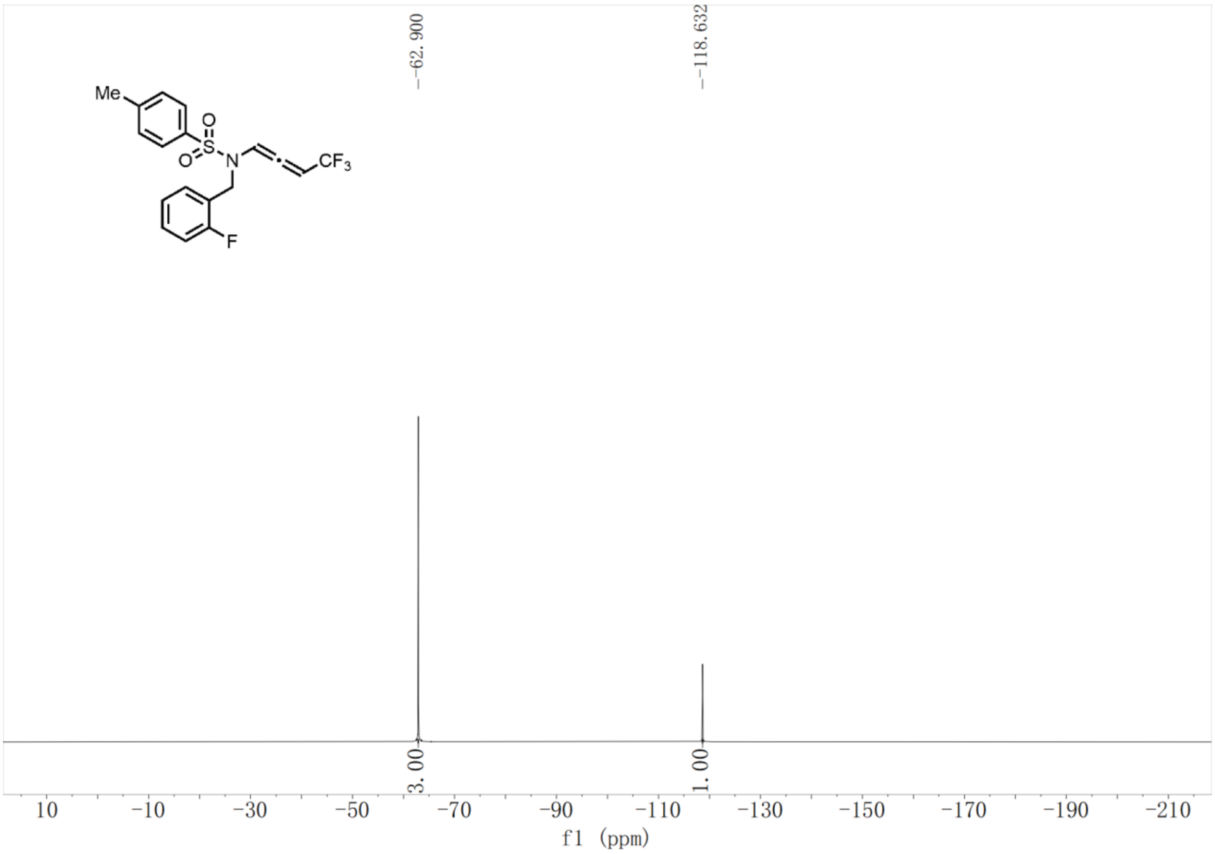

1k – ^{1}H NMR (400 MHz, $CDCl_3$)

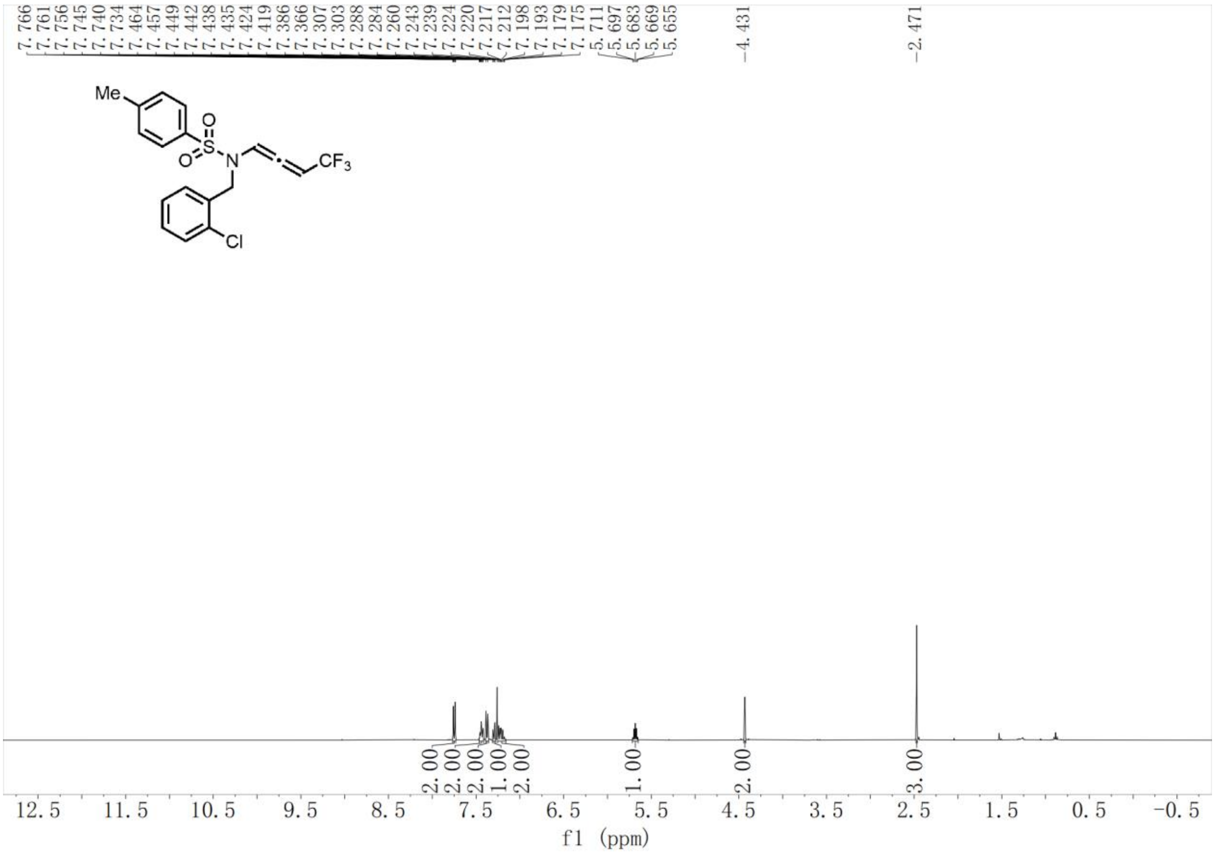

1k – ^{13}C NMR (126 MHz, $CDCl_3$)

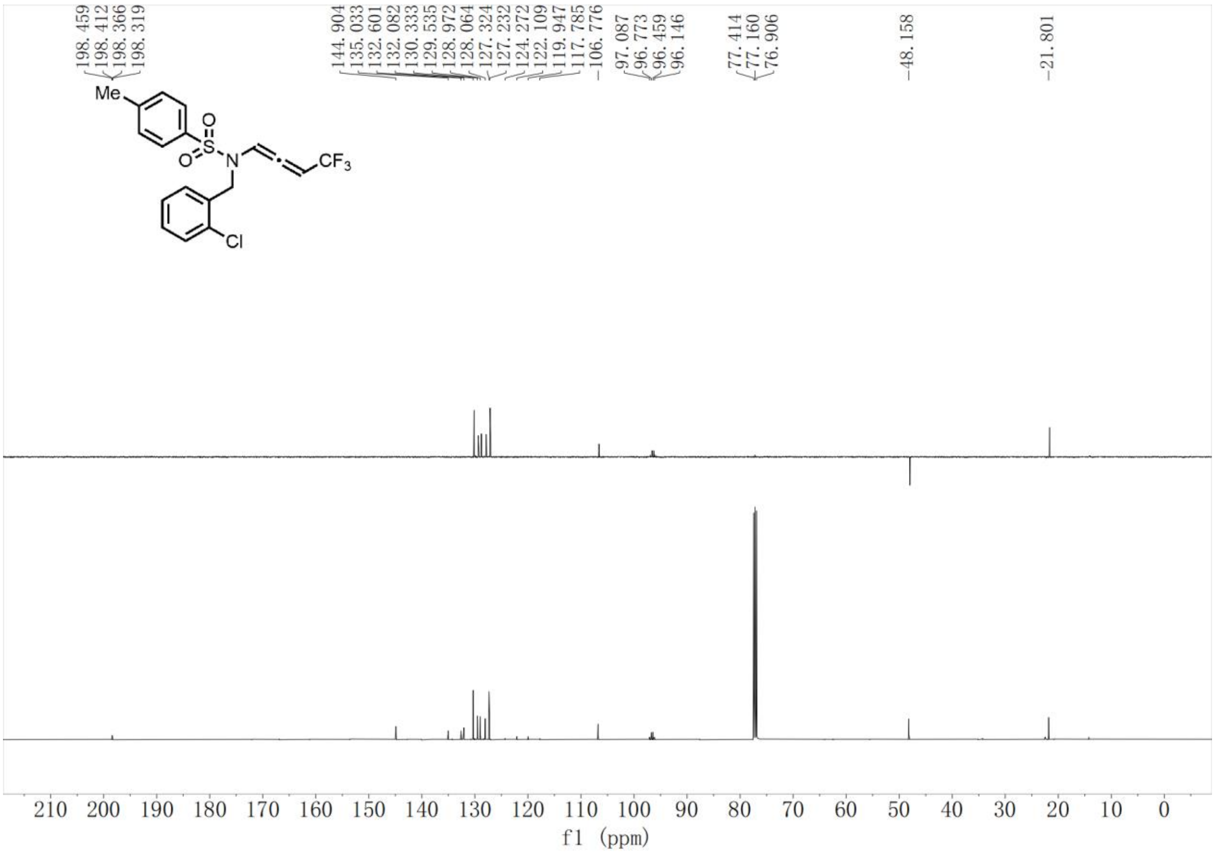

1k – ^{19}F NMR (282 MHz, $CDCl_3$)

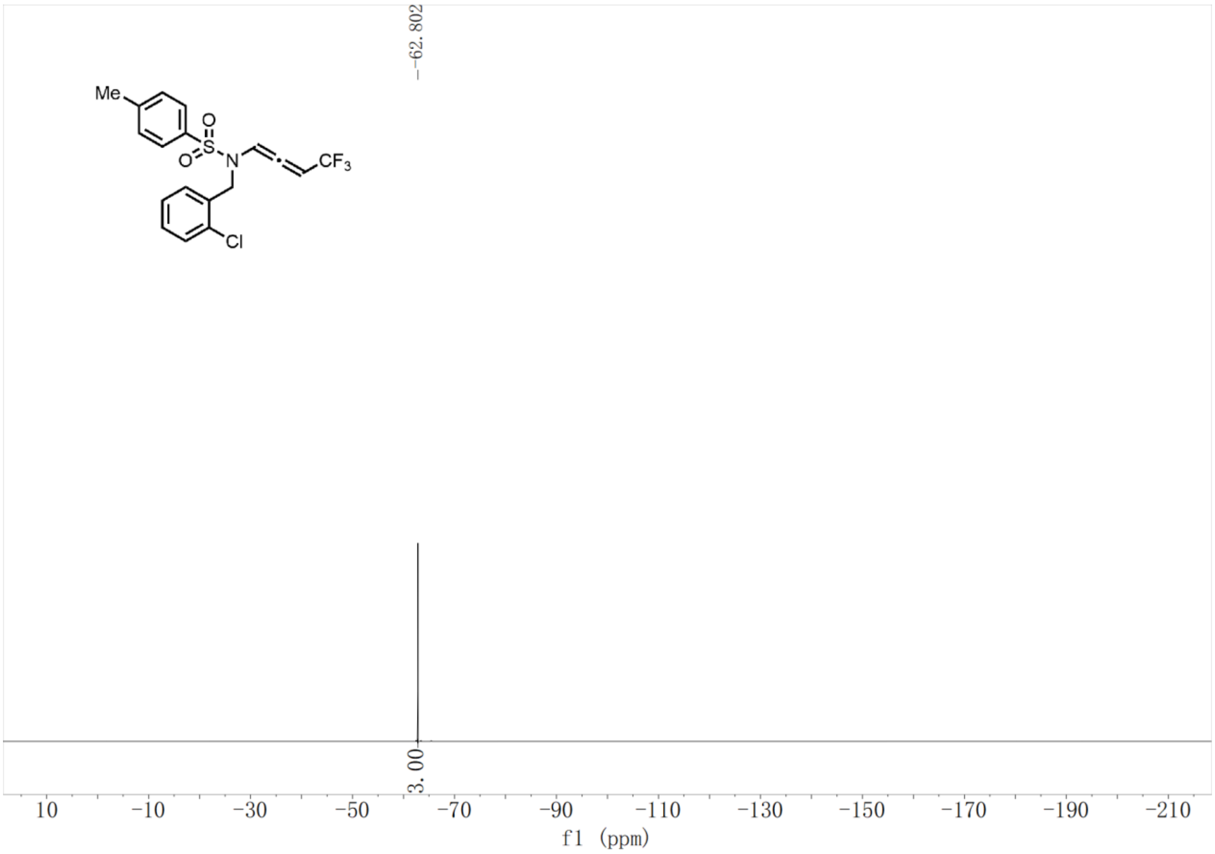

1n – ^{1}H NMR (300 MHz, $CDCl_3$)

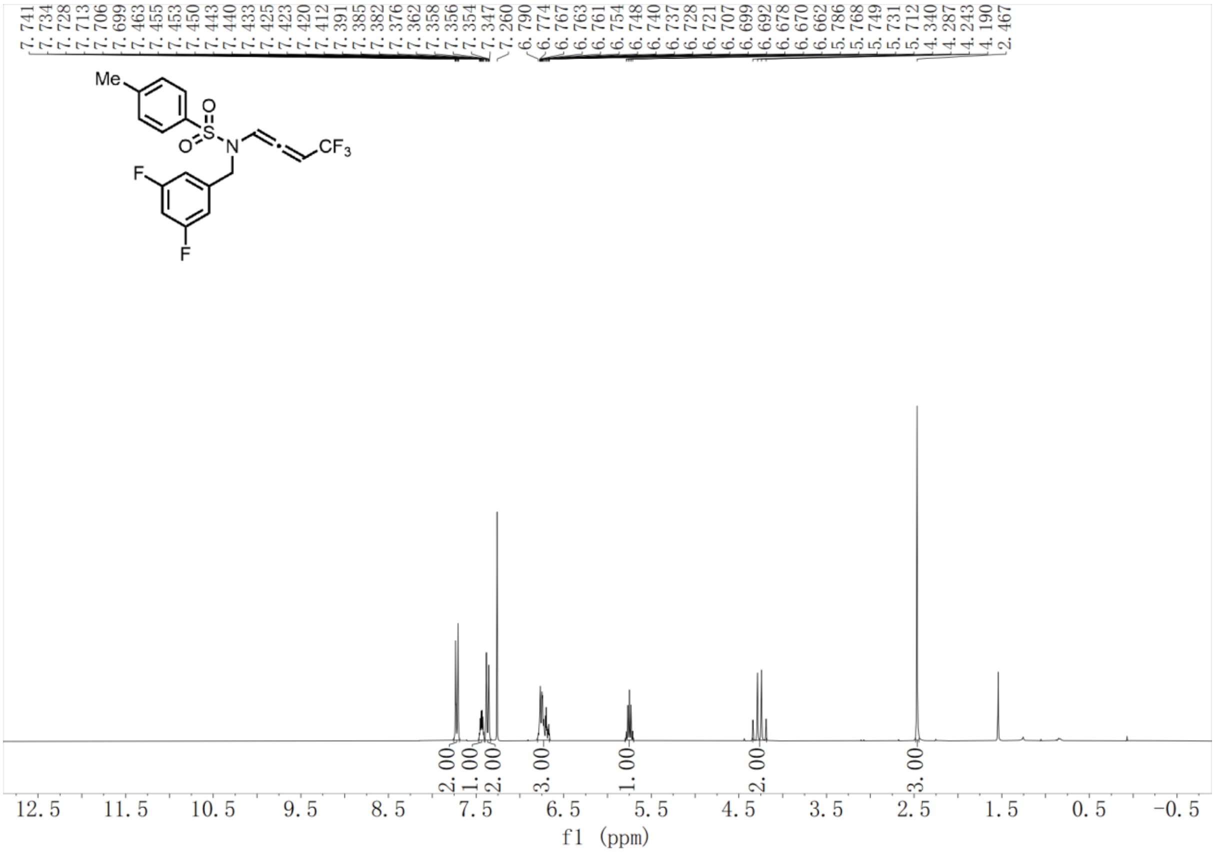

1n – ^{13}C NMR (126 MHz, $CDCl_3$)

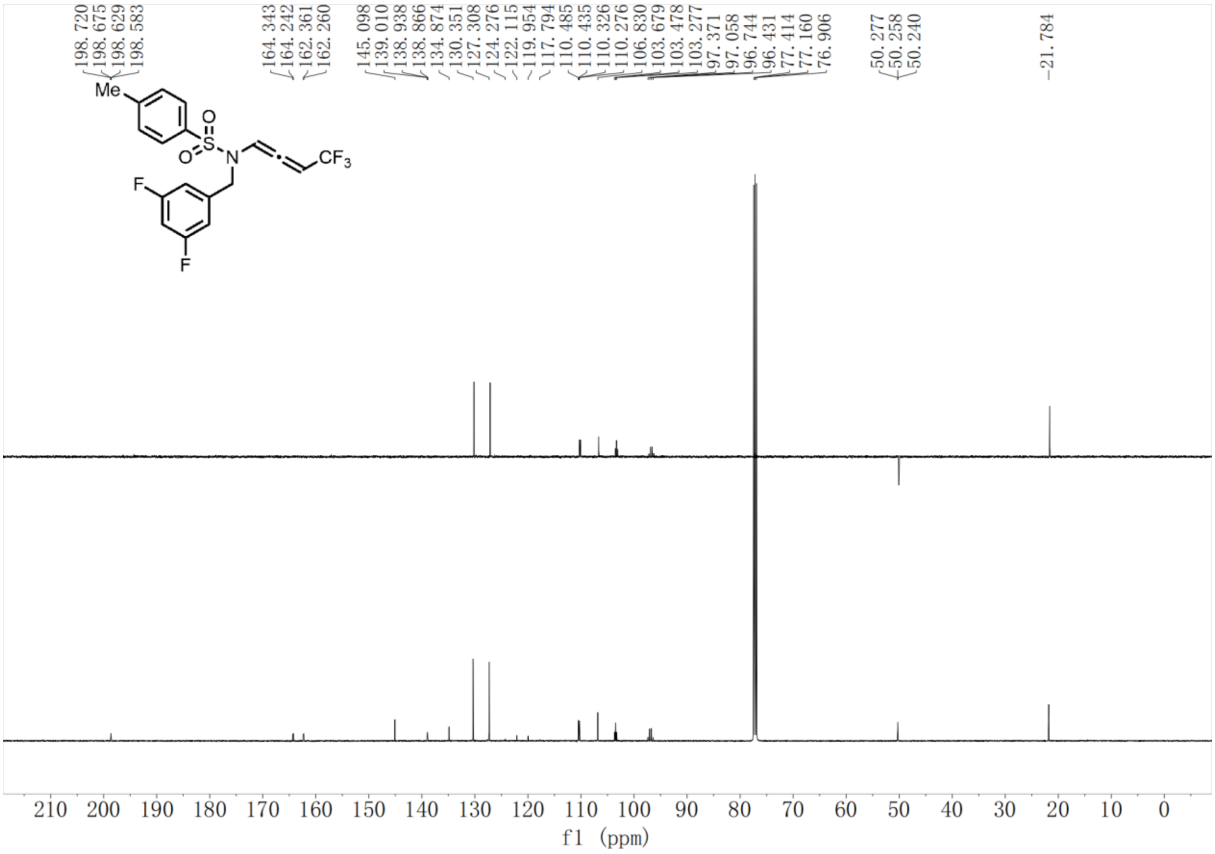

1n – ^{19}F NMR (282 MHz, $CDCl_3$)

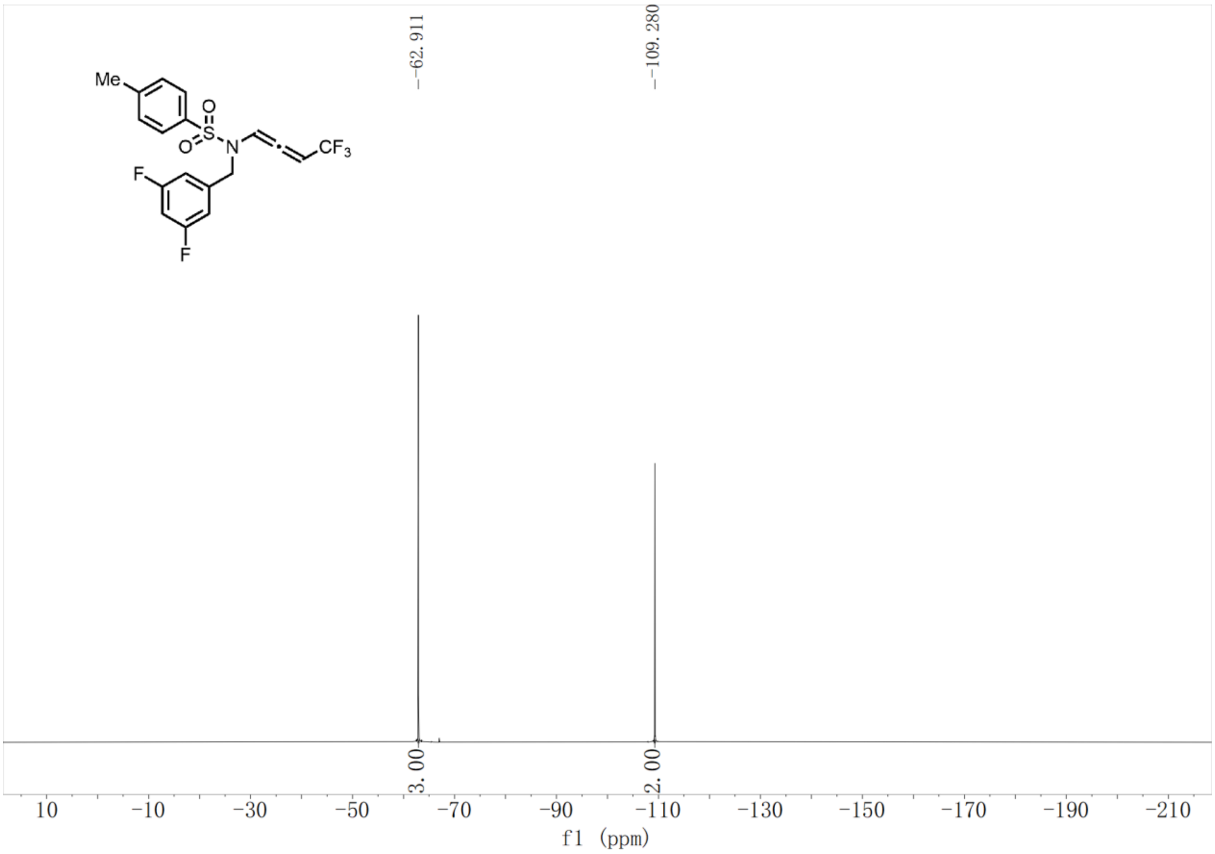

1o – ^{1}H NMR (500 MHz, $CDCl_3$)

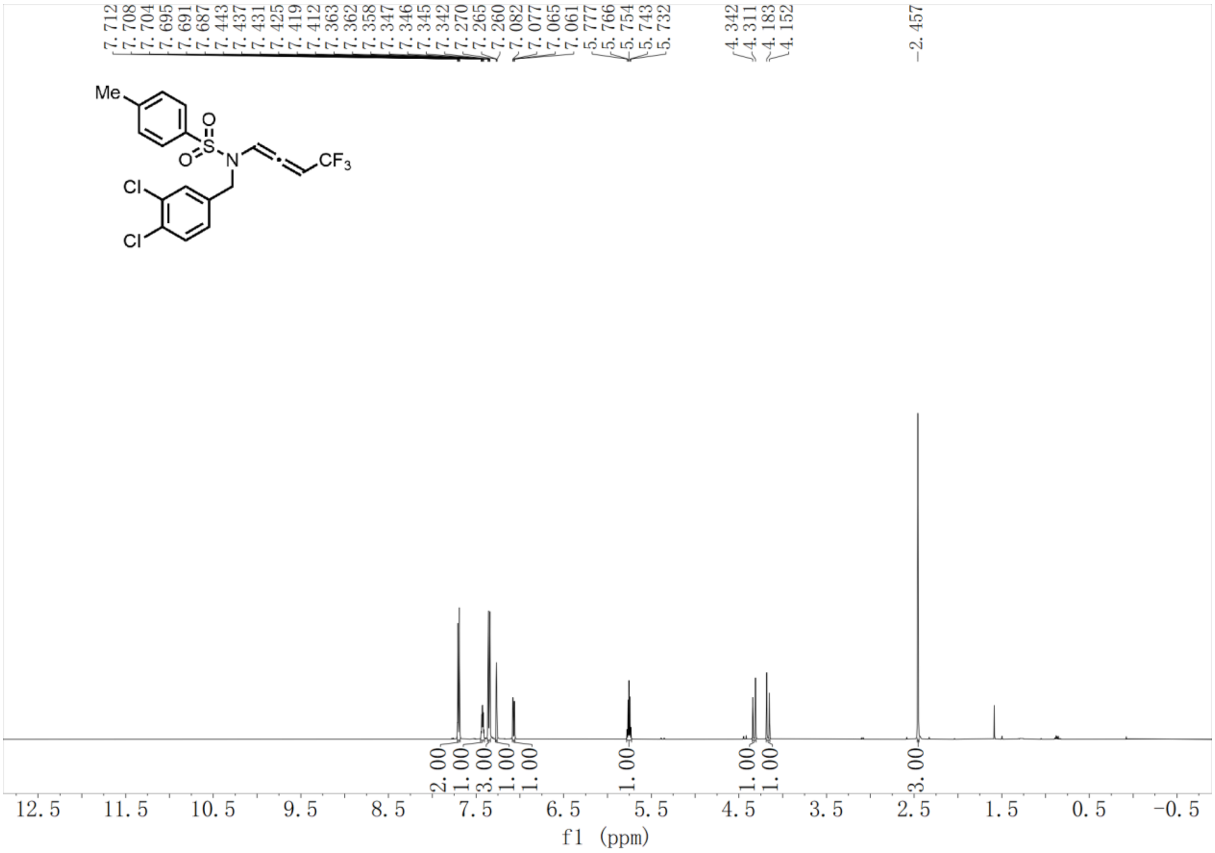

1o – ^{13}C NMR (126 MHz, $CDCl_3$)

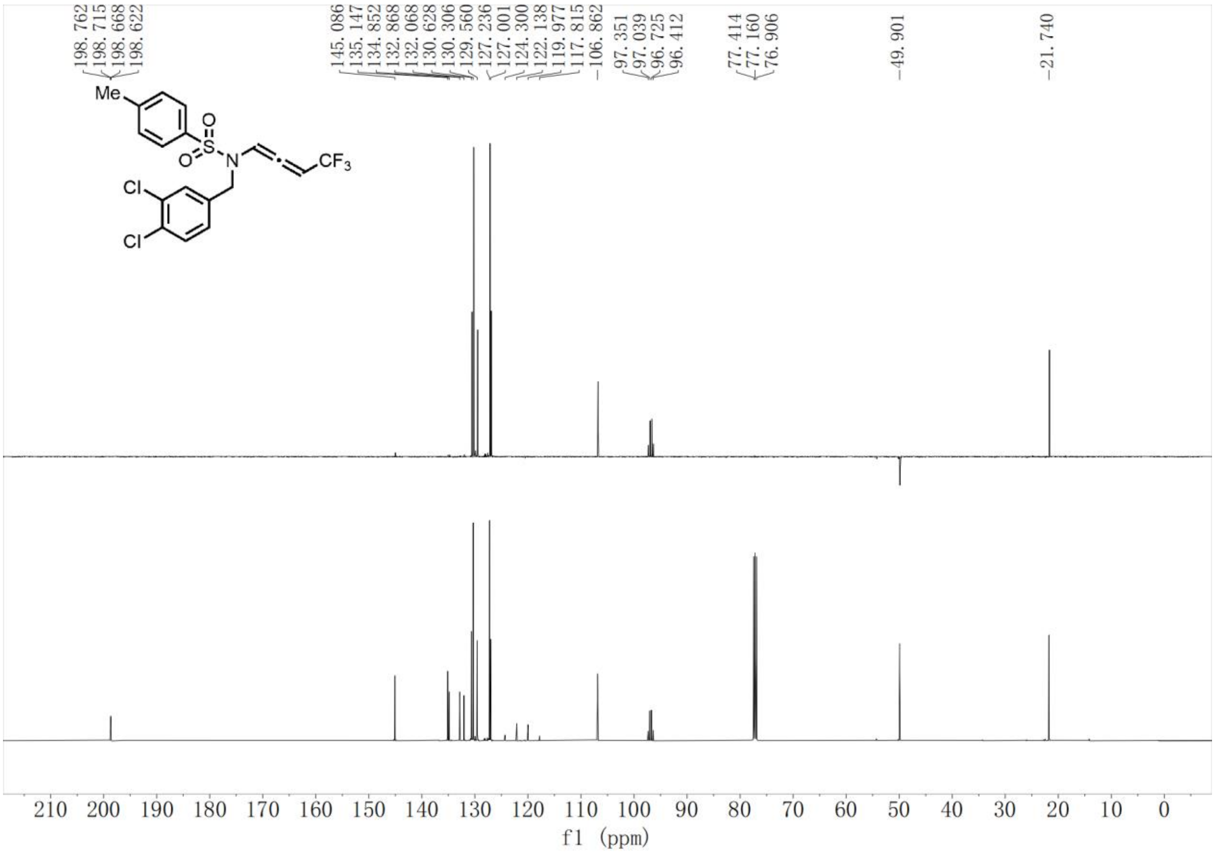

1o – ^{19}F NMR (471 MHz, $CDCl_3$)

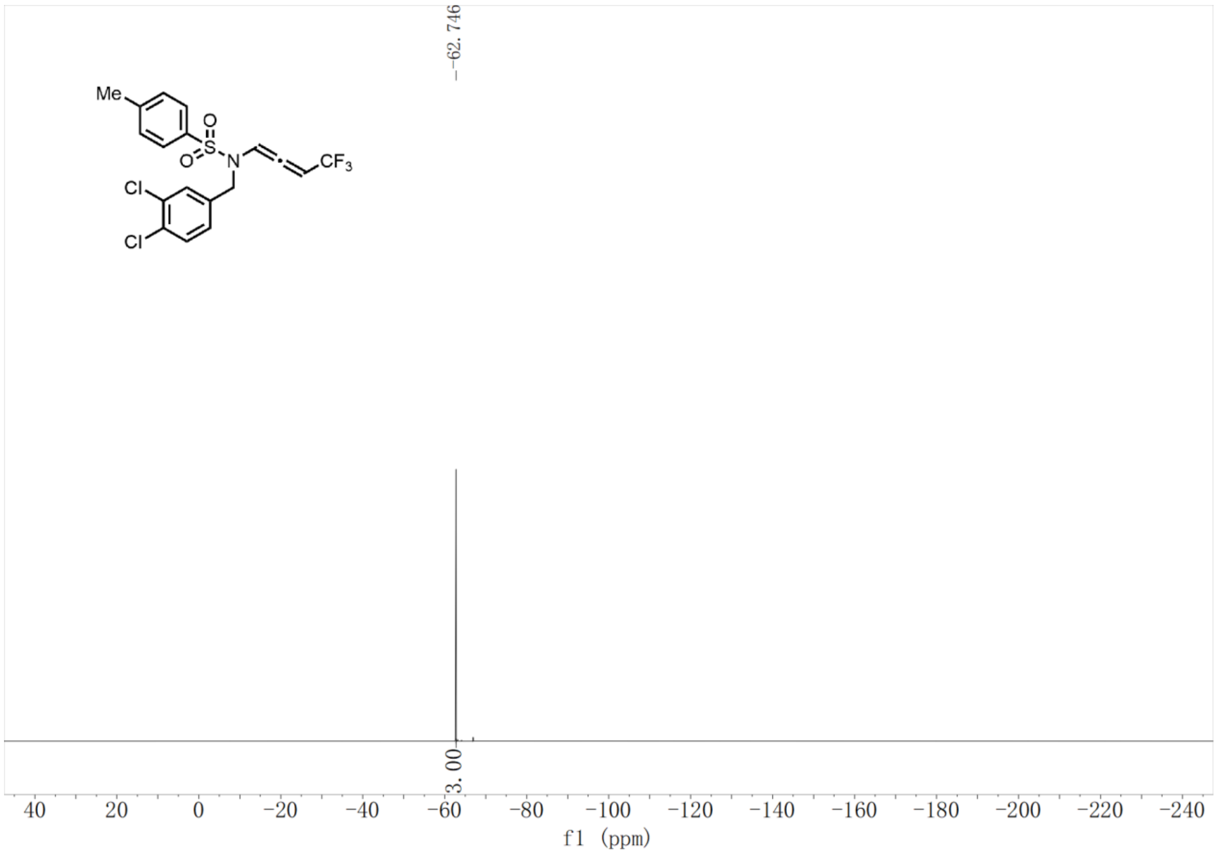

1q – ^{1}H NMR (500 MHz, $CDCl_3$)

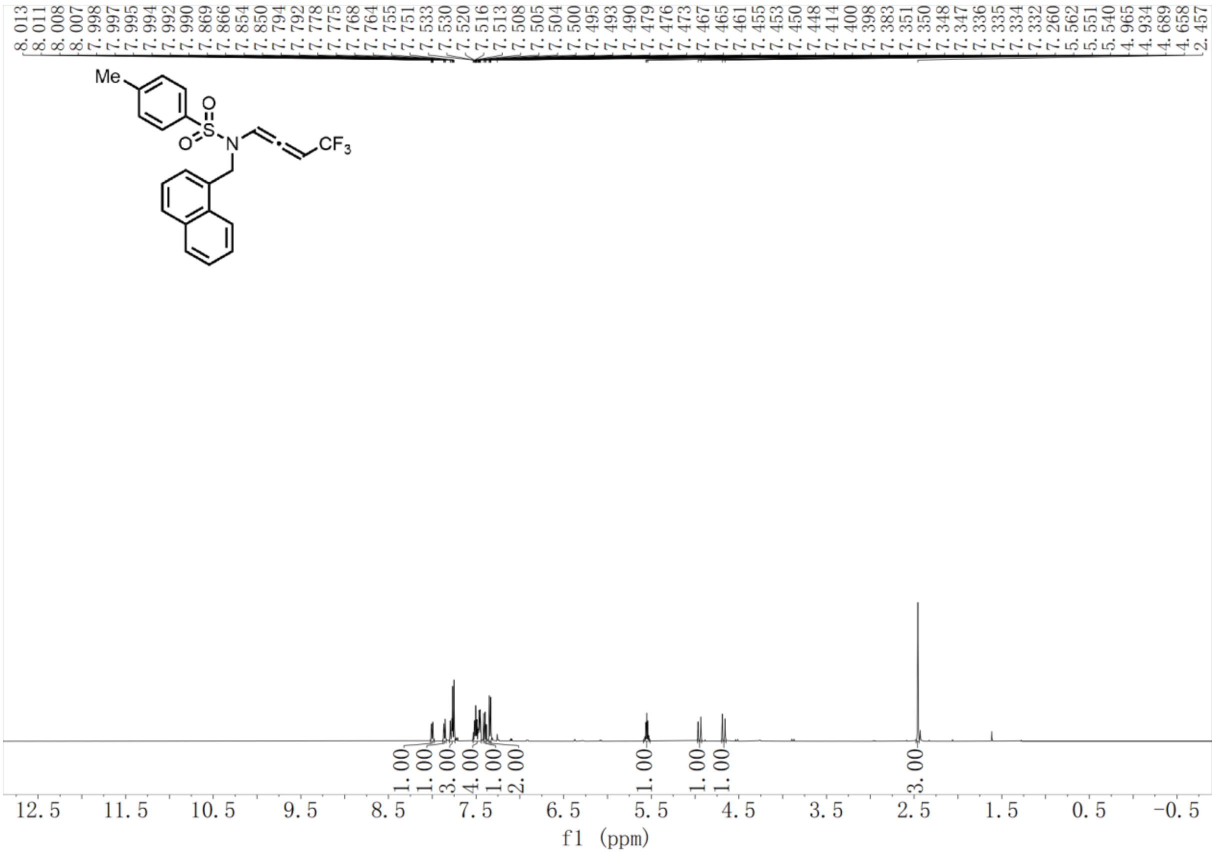

1q – ^{13}C NMR (126 MHz, $CDCl_3$)

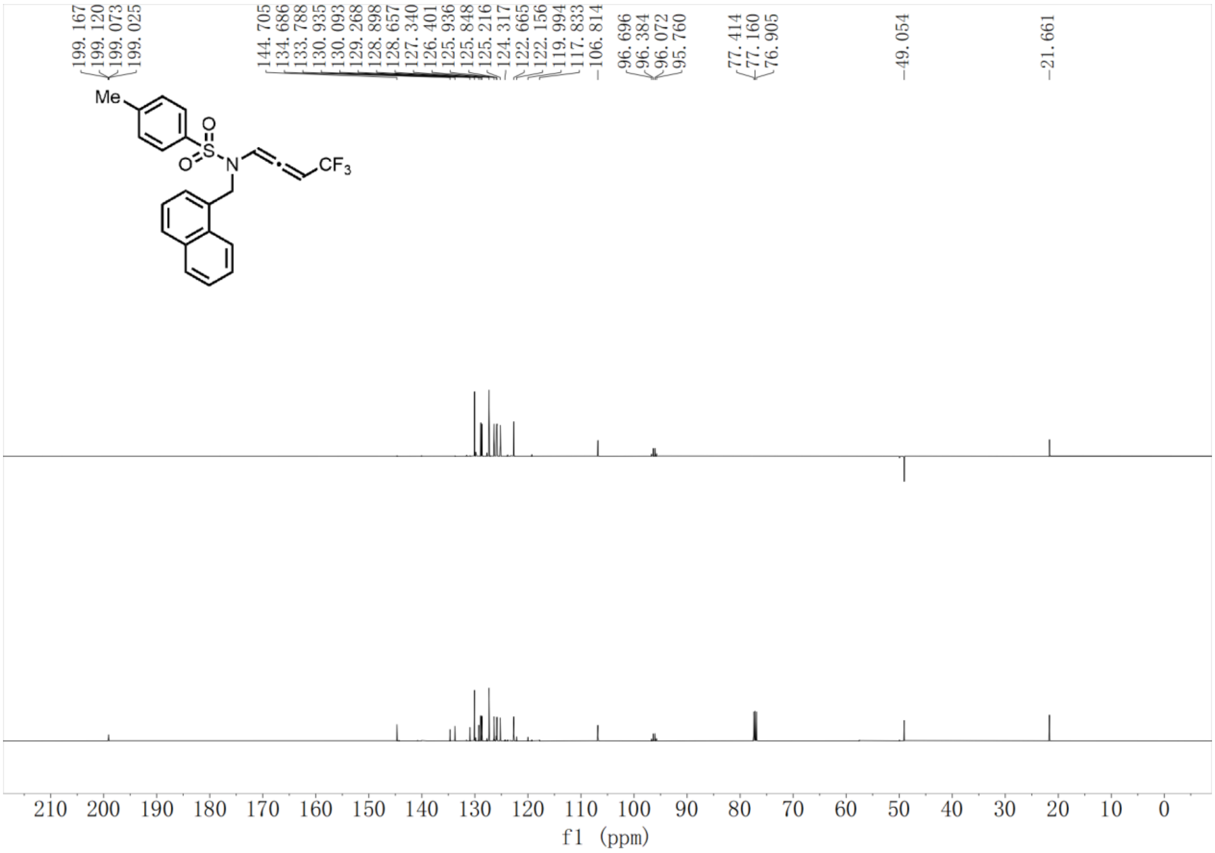

1q – ^{19}F NMR (471 MHz, $CDCl_3$)

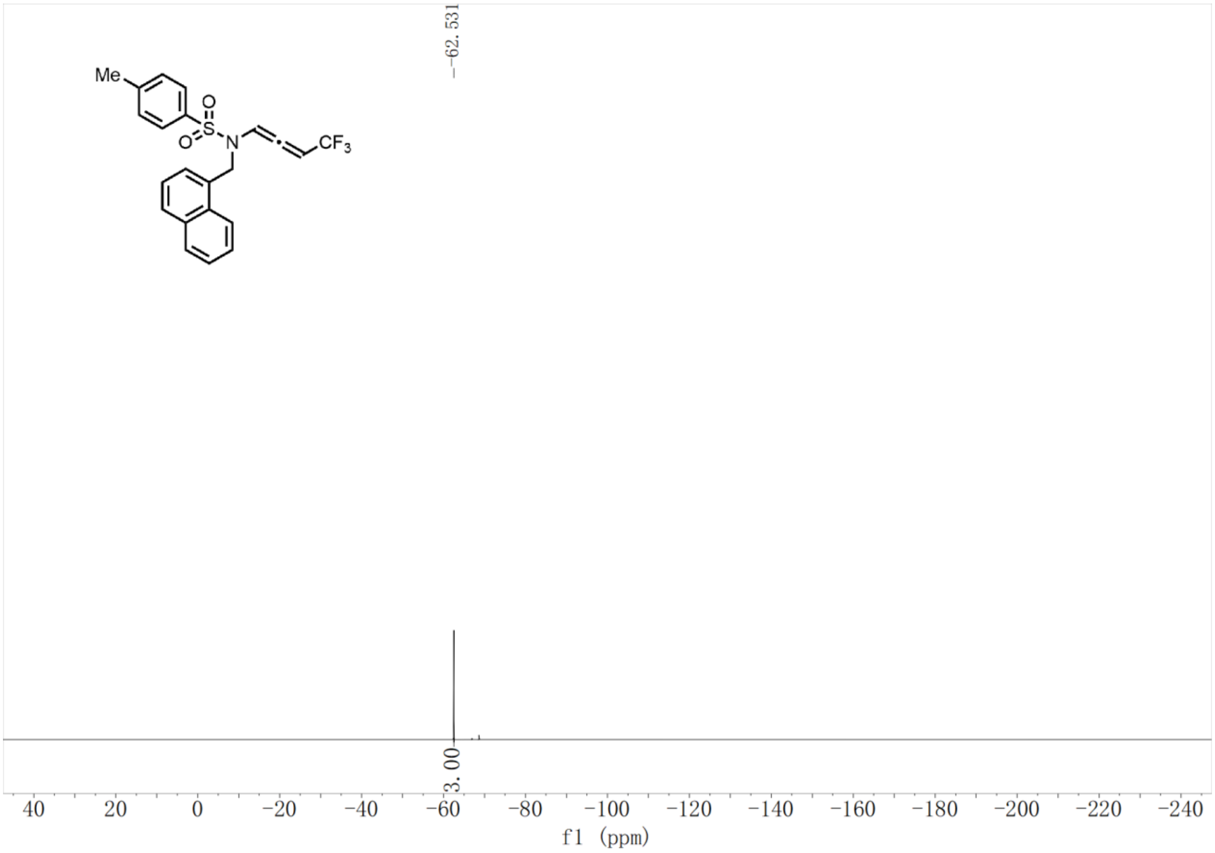

1t – ^{1}H NMR (500 MHz, $CDCl_3$)

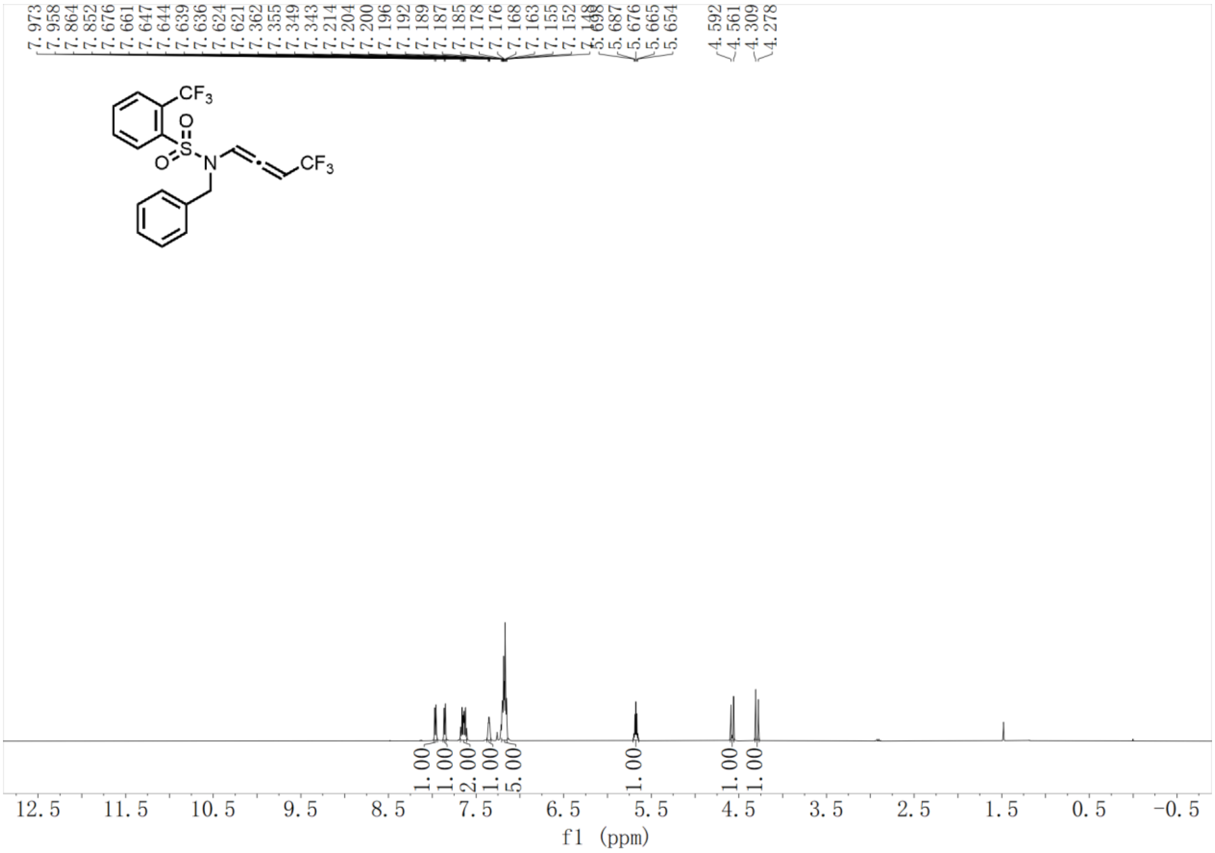

1t – ^{13}C NMR (126 MHz, $CDCl_3$)

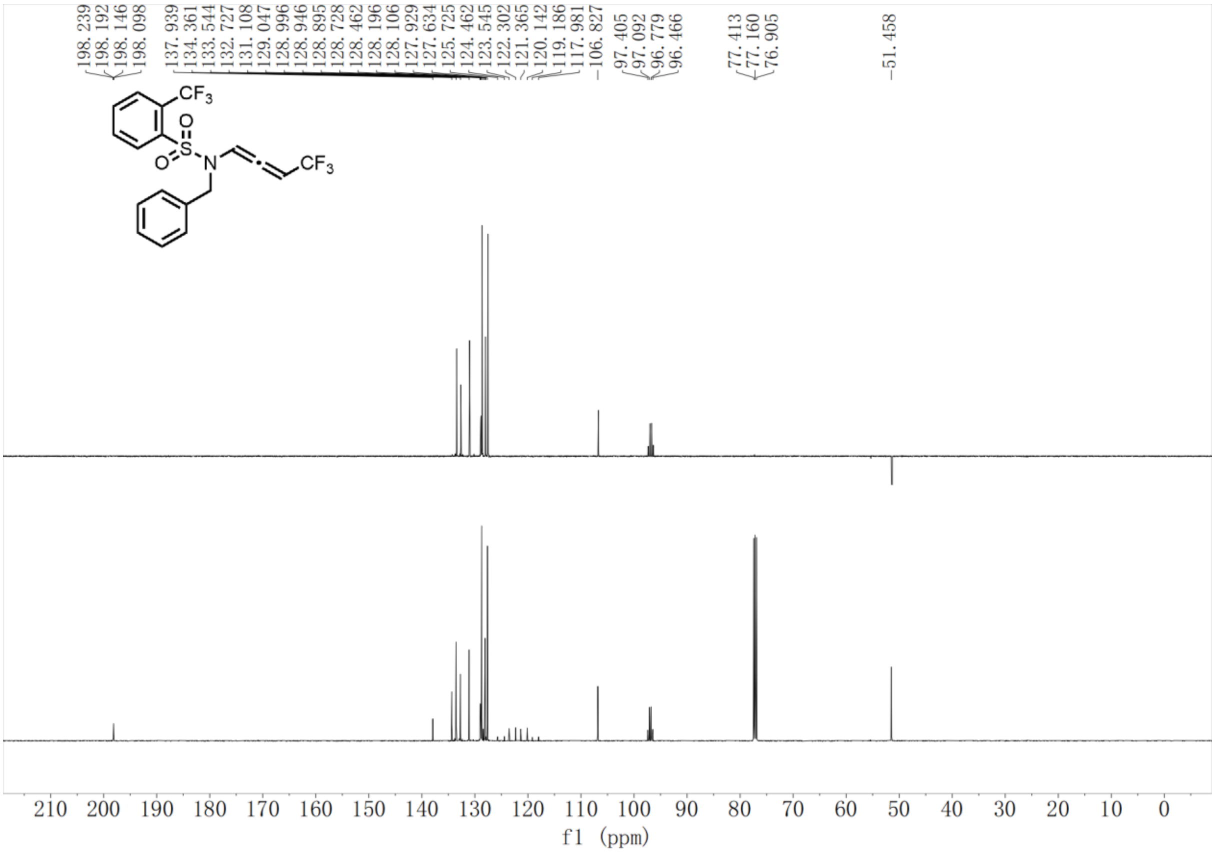

1t – ^{19}F NMR (282 MHz, $CDCl_3$)

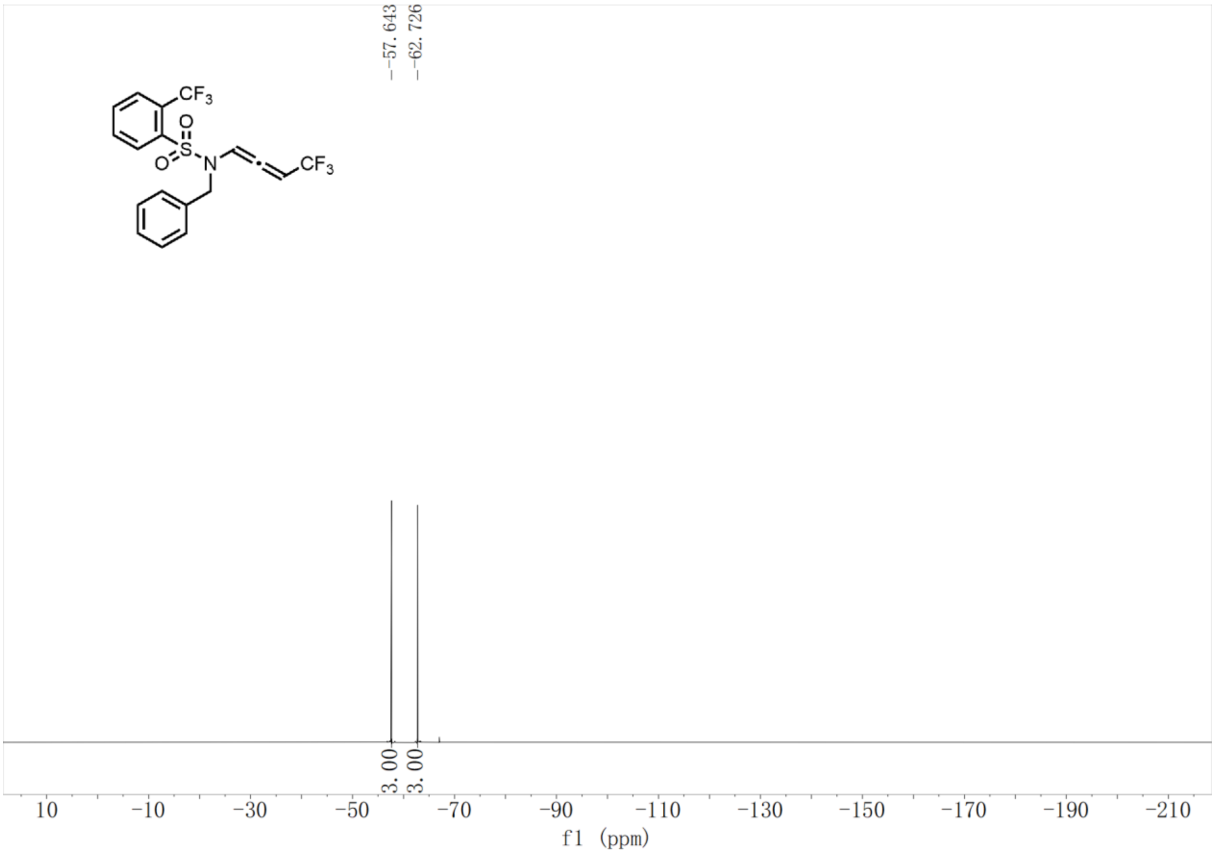

1u – ^{1}H NMR (500 MHz, $CDCl_3$)

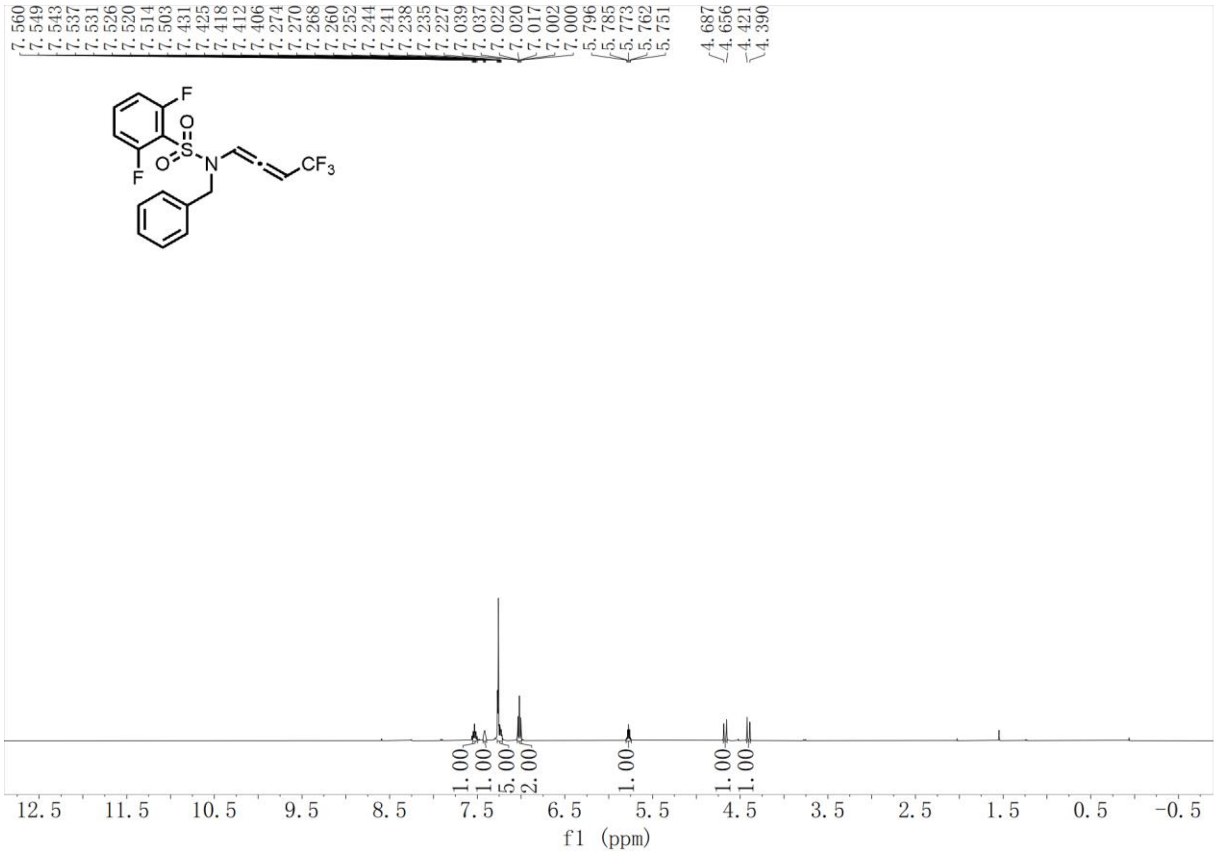

1u – ^{13}C NMR (126 MHz, $CDCl_3$)

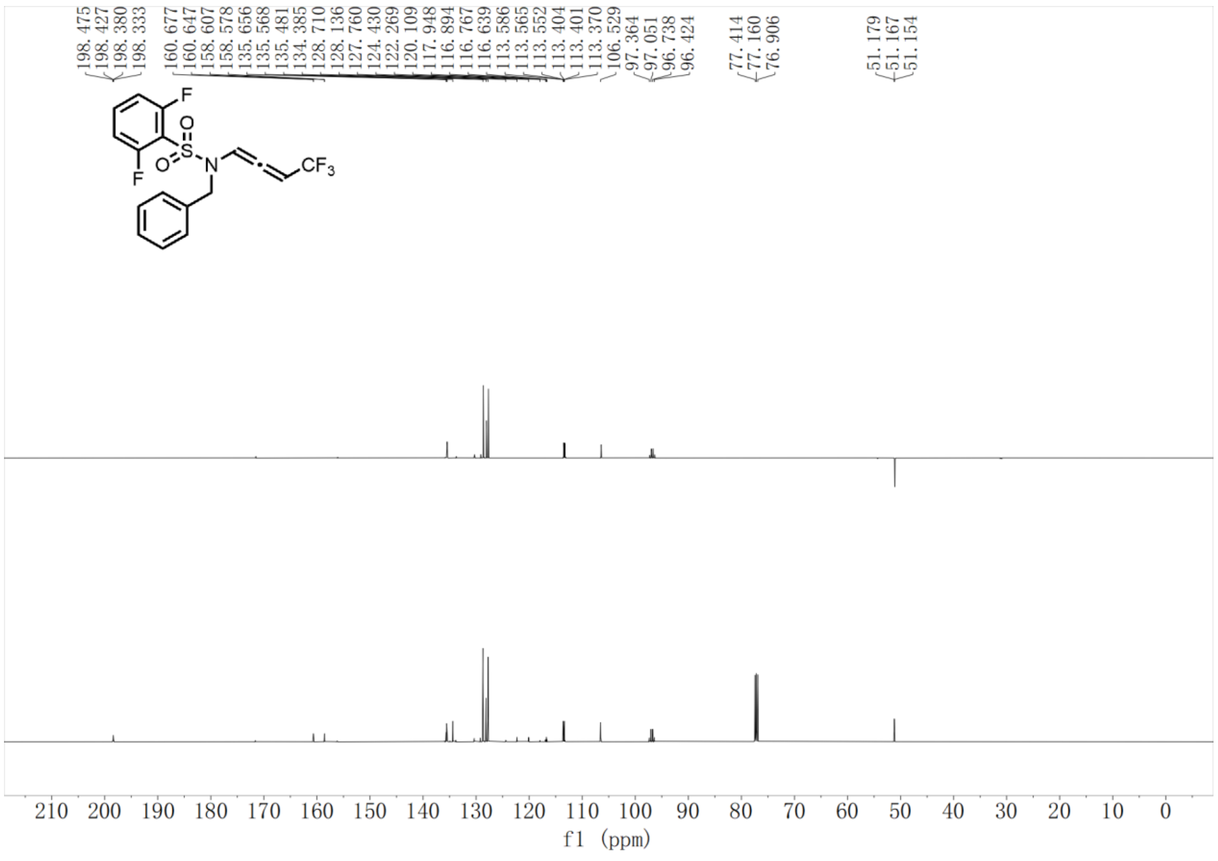

1u – ^{19}F NMR (282 MHz, $CDCl_3$)

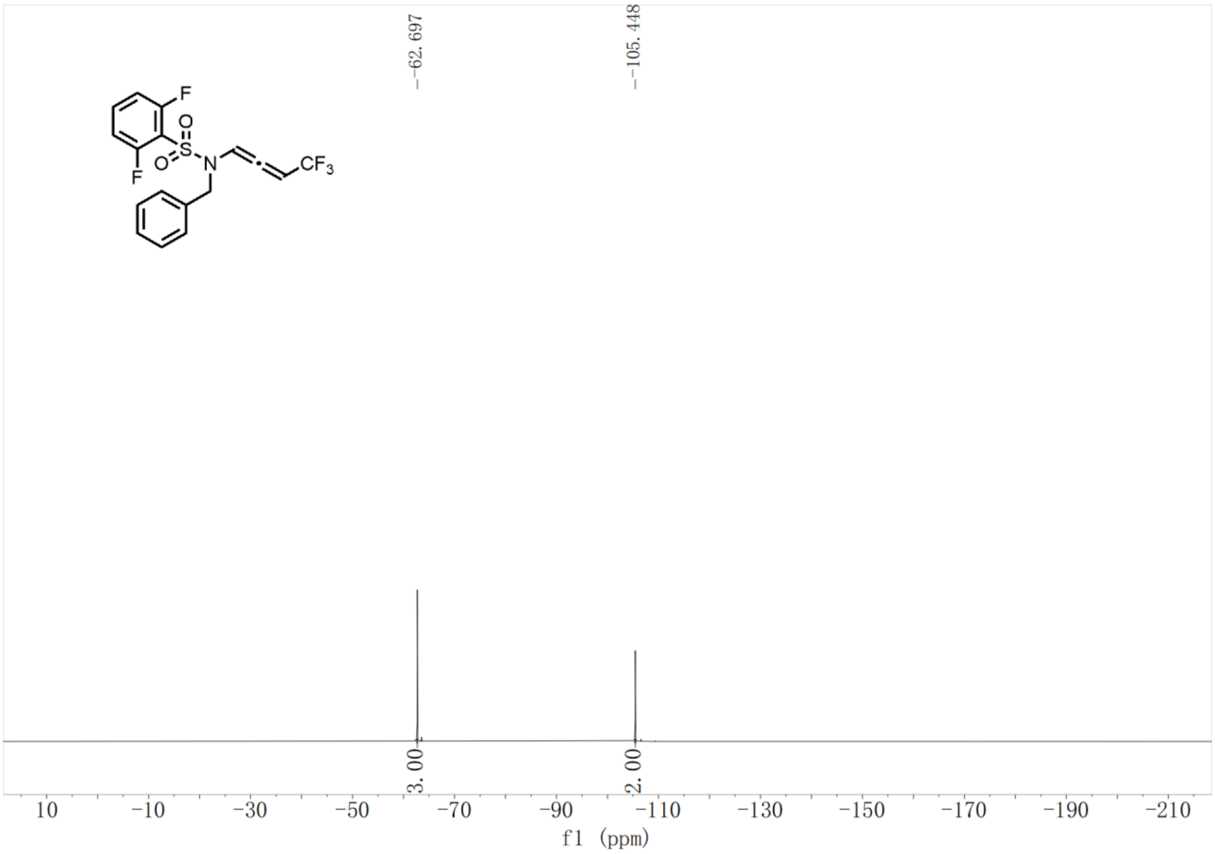

1v – ^{1}H NMR (500 MHz, $CDCl_3$)

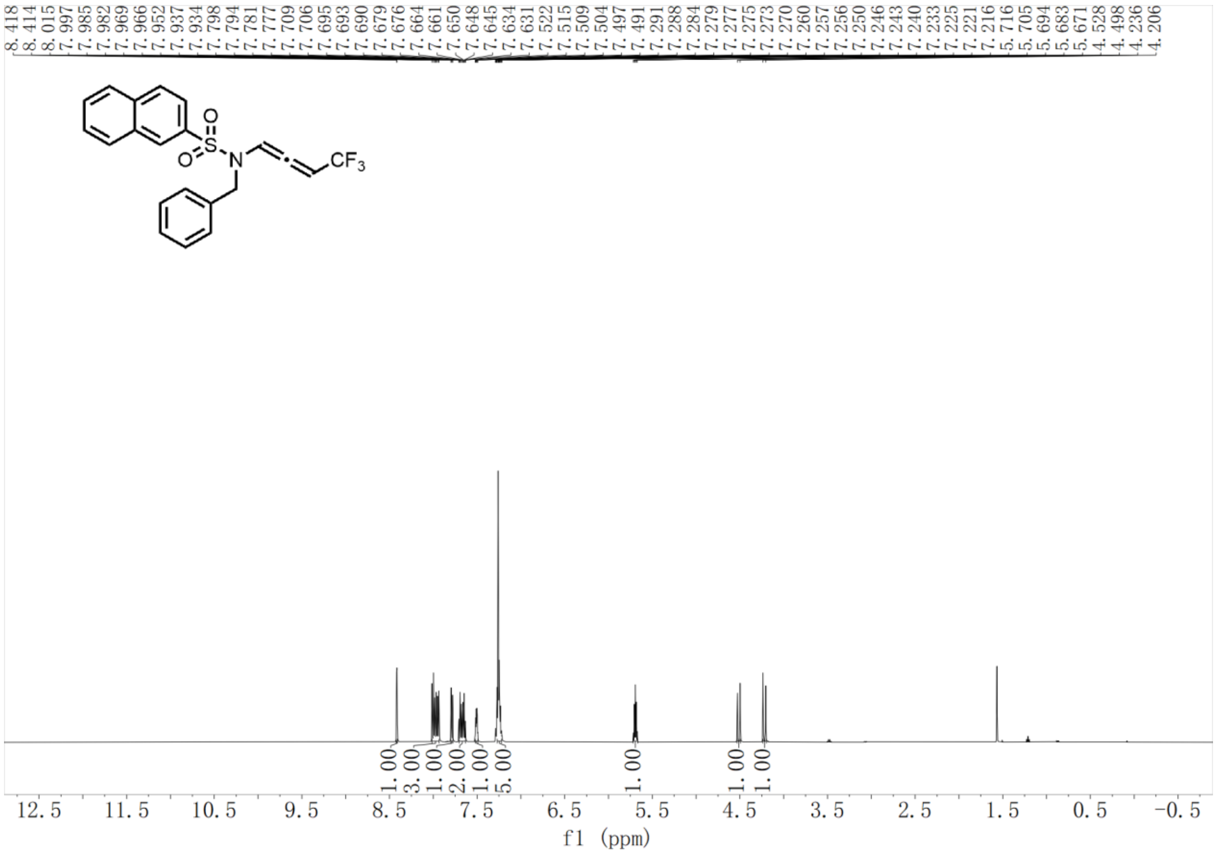

1v – ^{13}C NMR (126 MHz, $CDCl_3$)

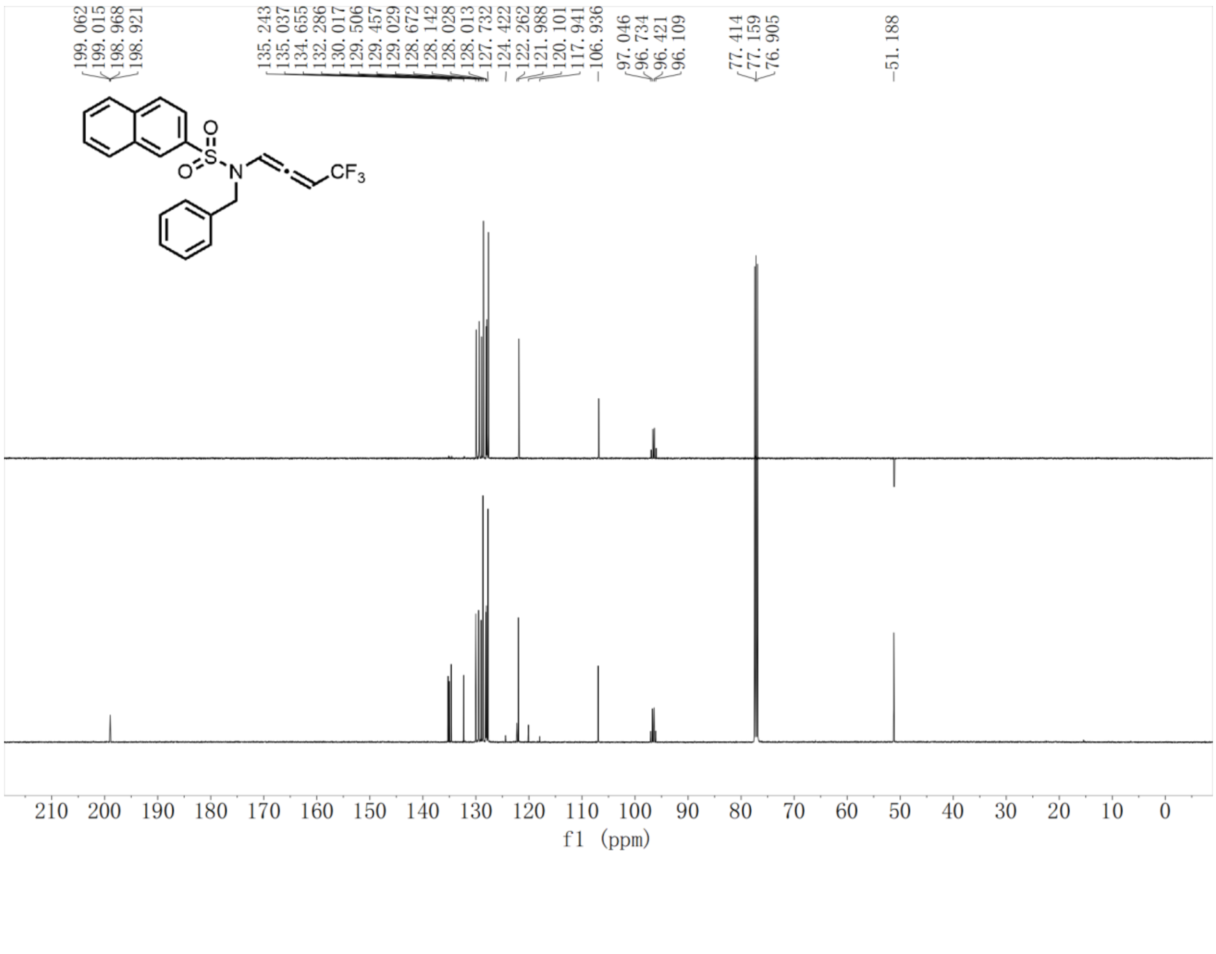

1v – ^{19}F NMR (471 MHz, $CDCl_3$)

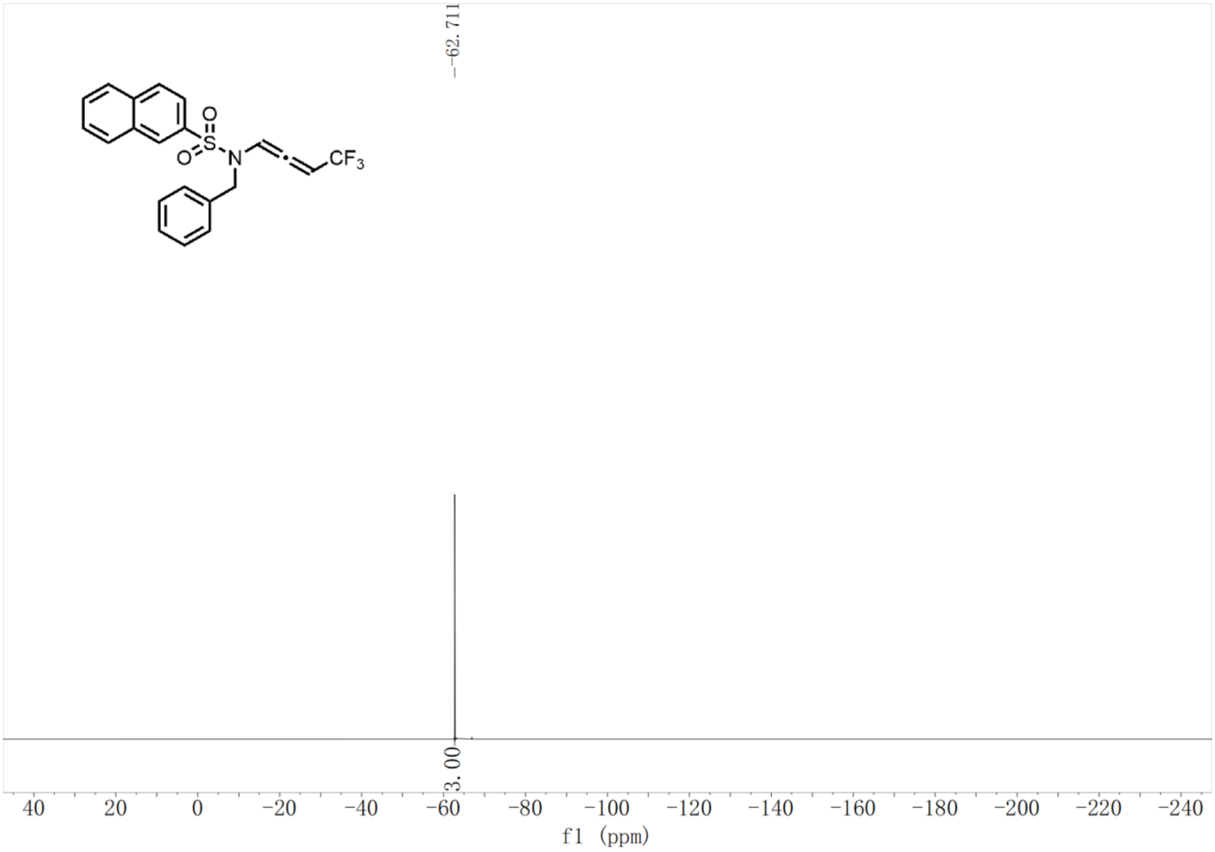

2 – ^{1}H NMR (500 MHz, DMSO)

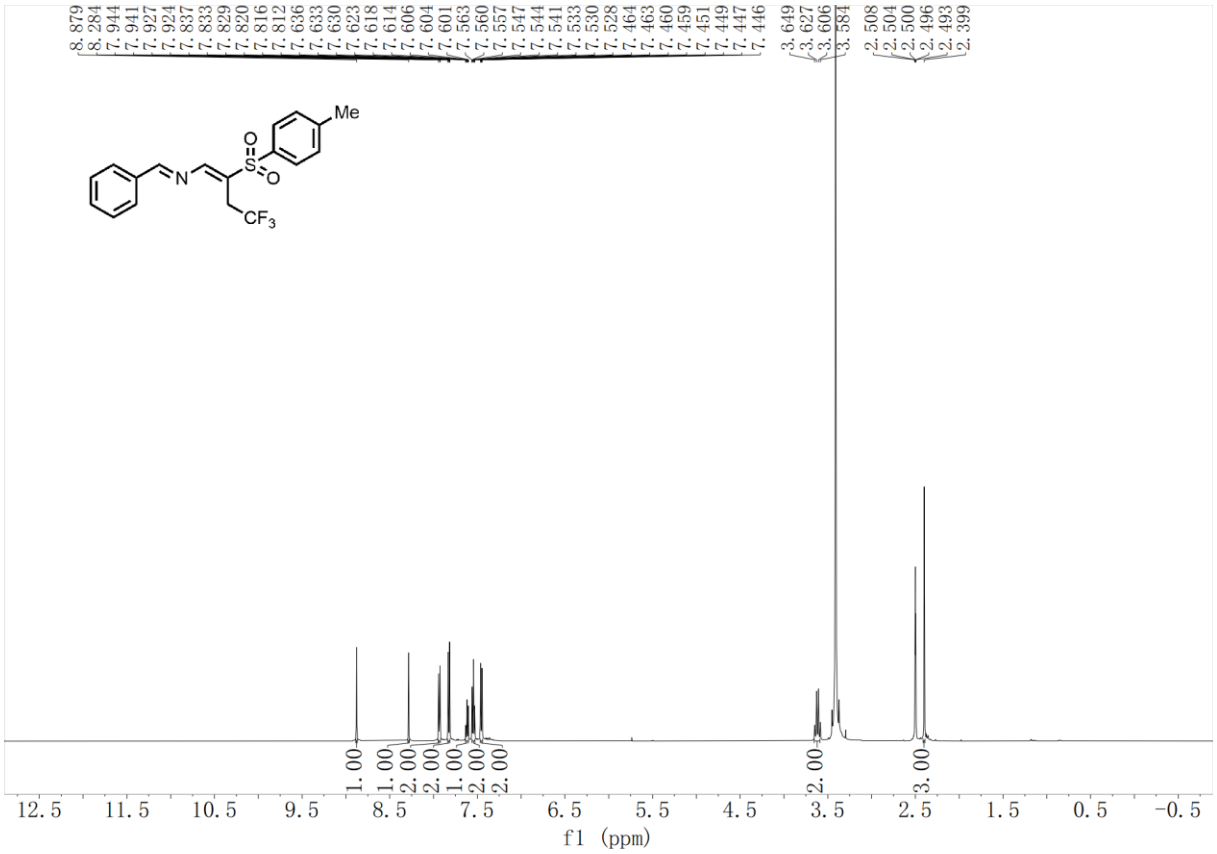

2 – ^{13}C NMR (126 MHz, DMSO)

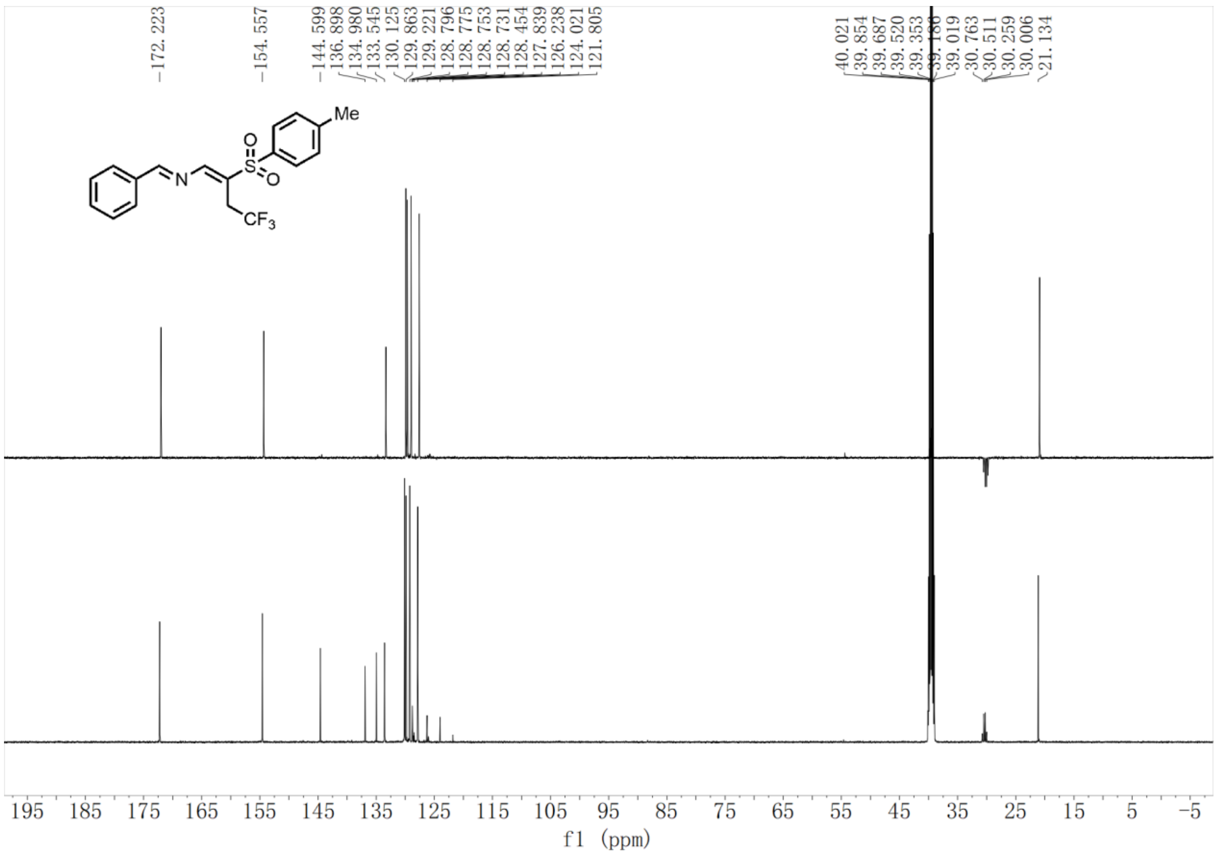

2 – ^{19}F NMR (282 MHz, DMSO)

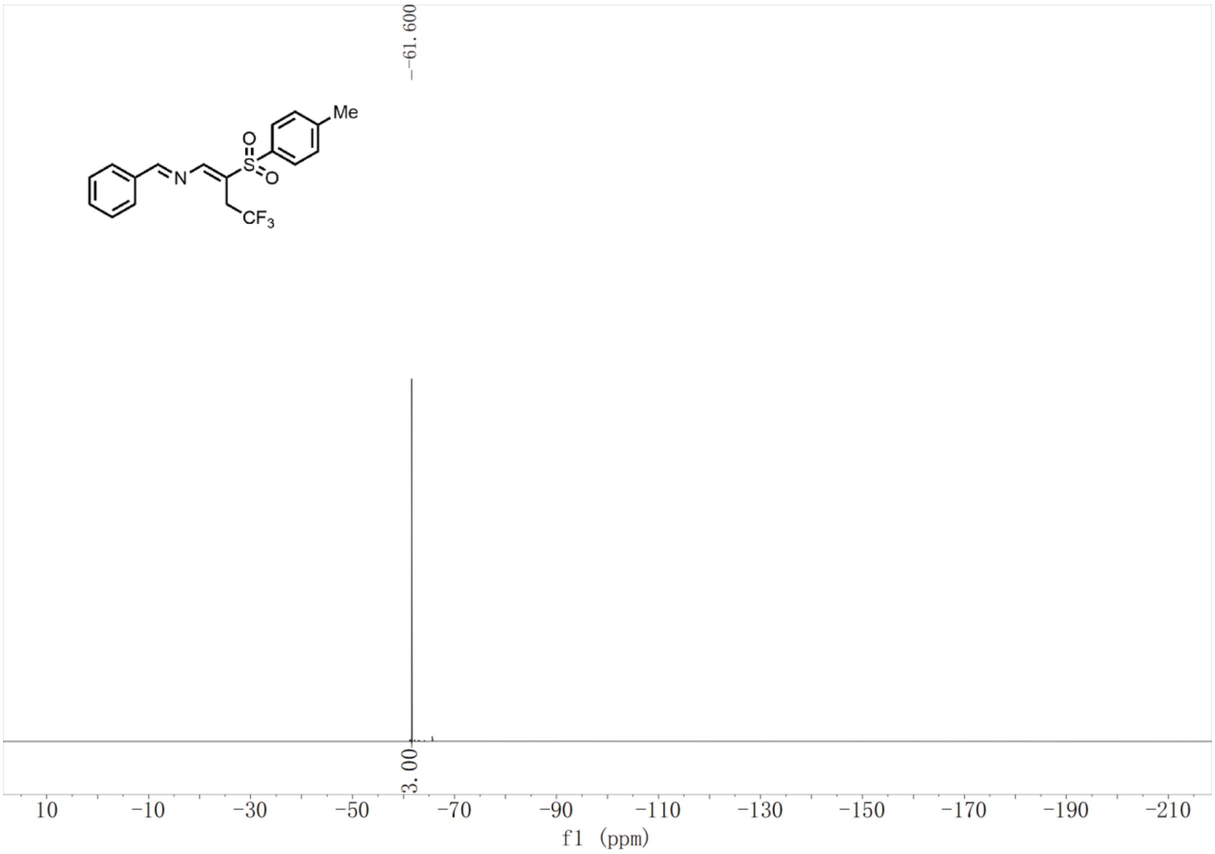

4a – ^{1}H NMR (500 MHz, DMSO)

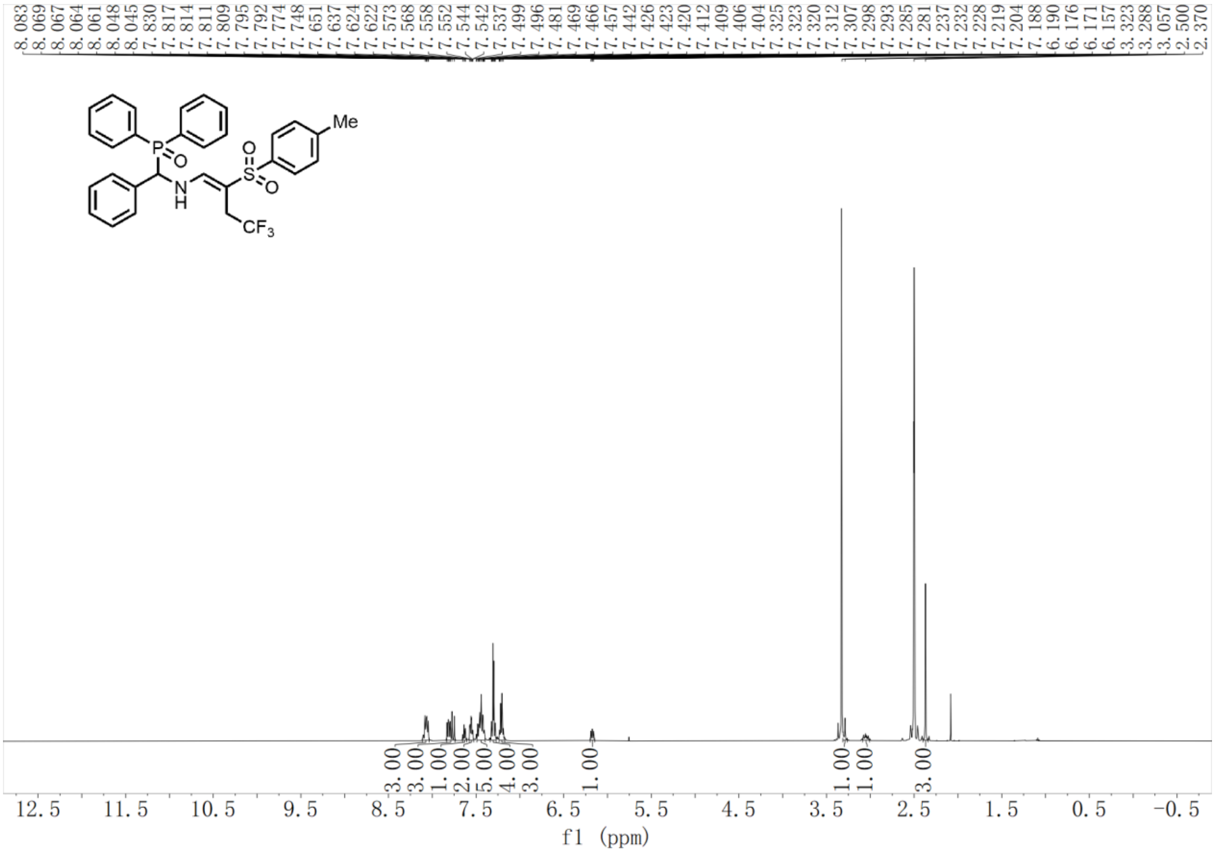

4a – ^{13}C NMR (126 MHz, DMSO)

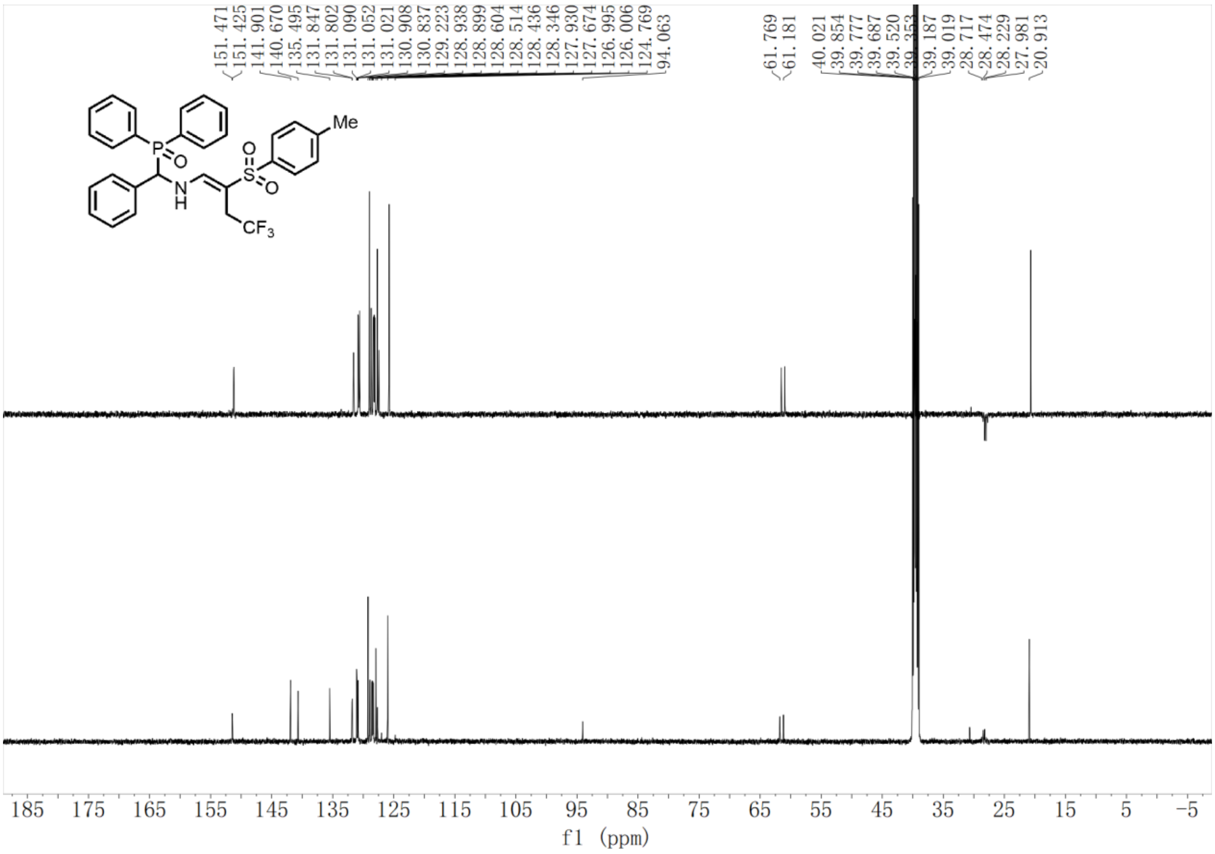

4a – ^{19}F NMR (471 MHz, DMSO)

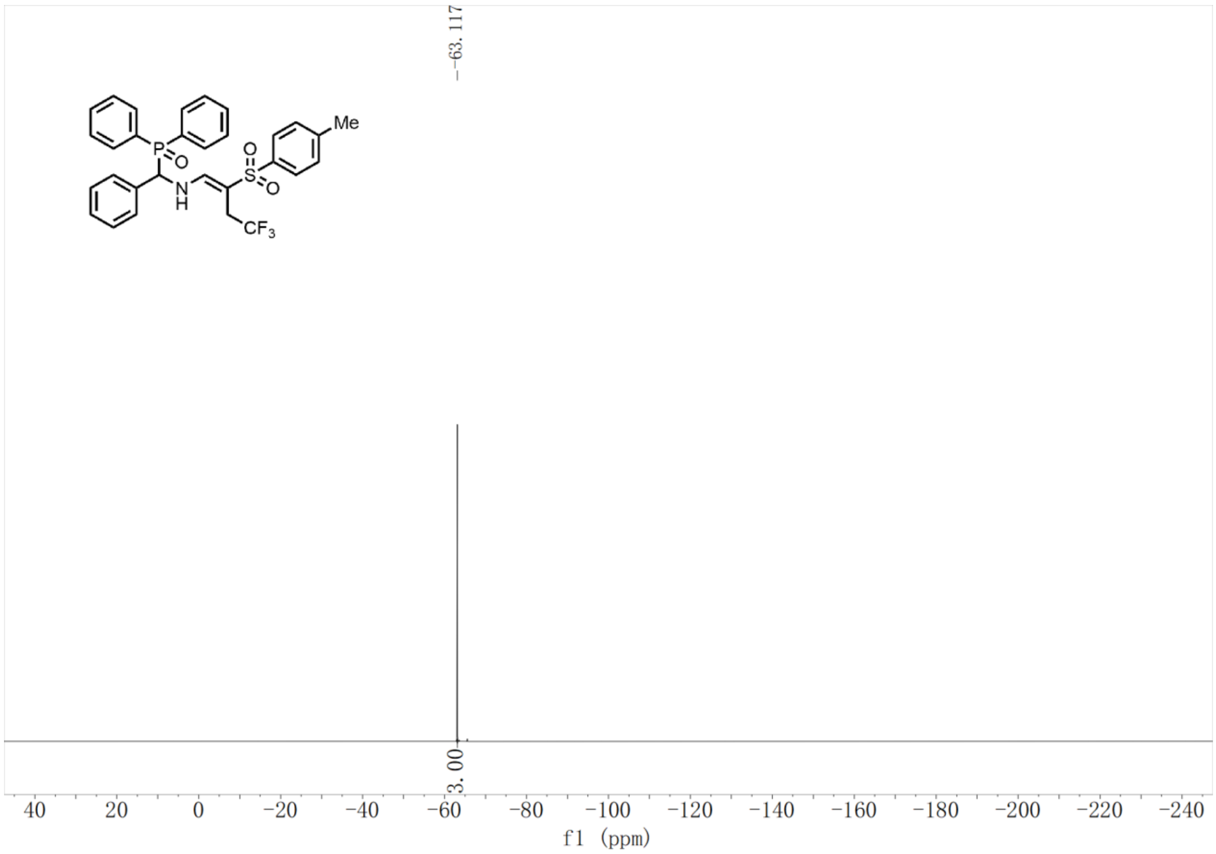

4a – ^{31}P NMR (202 MHz, DMSO)

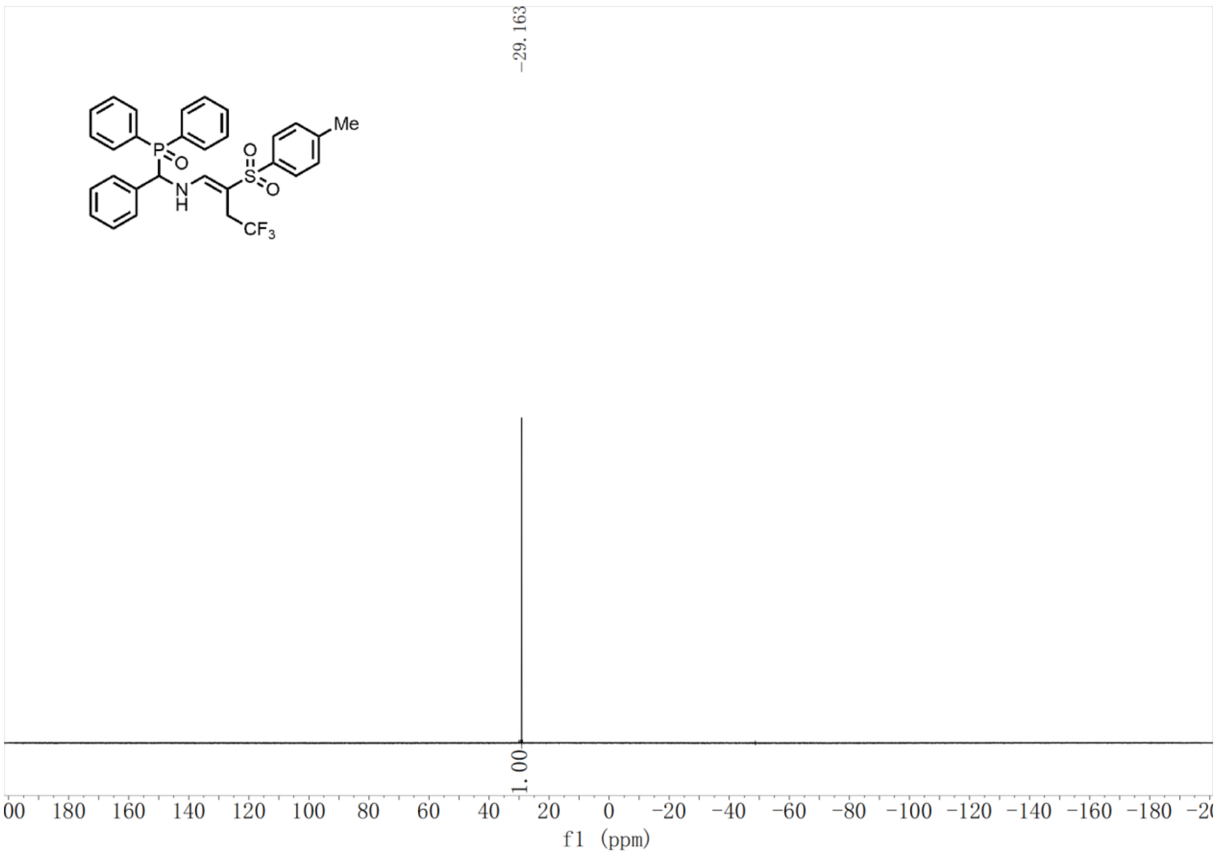

4b – ^{1}H NMR (500 MHz, DMSO)

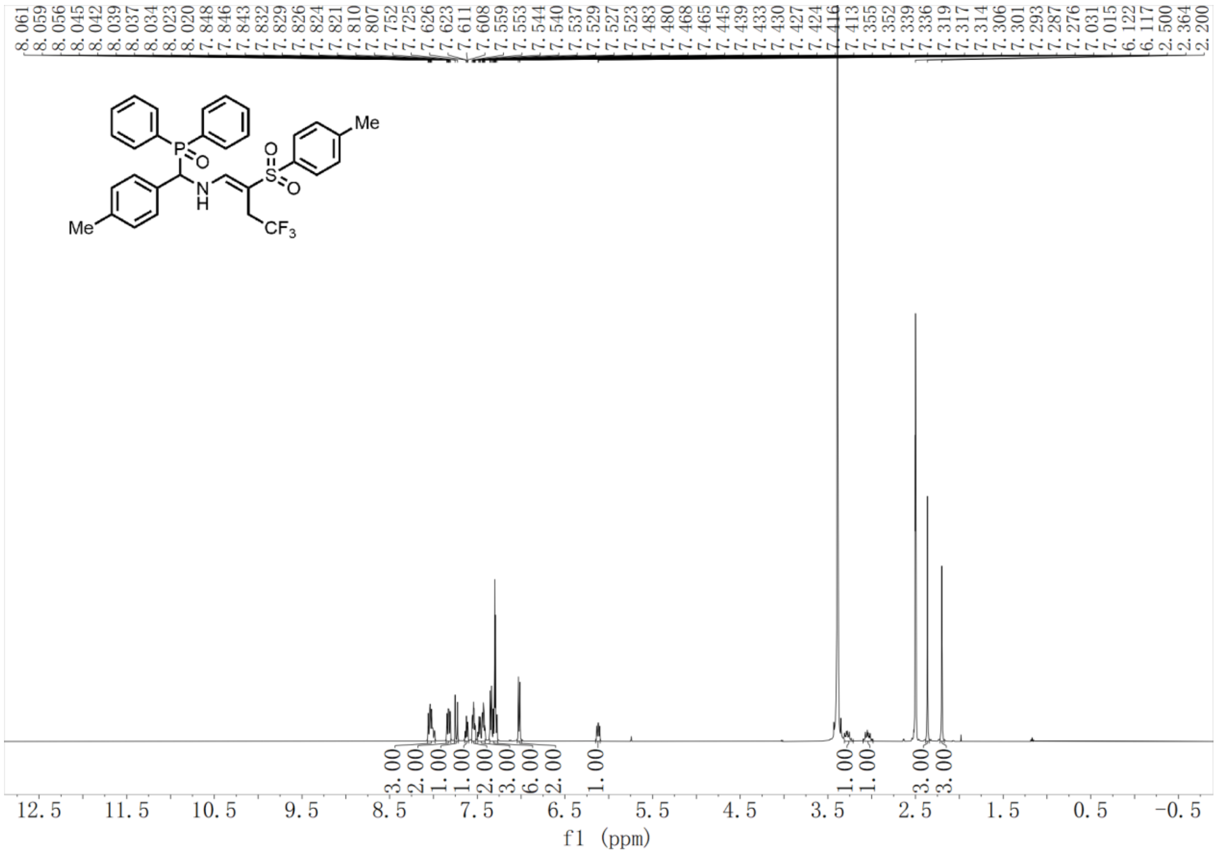

4b – ^{13}C NMR (126 MHz, DMSO)

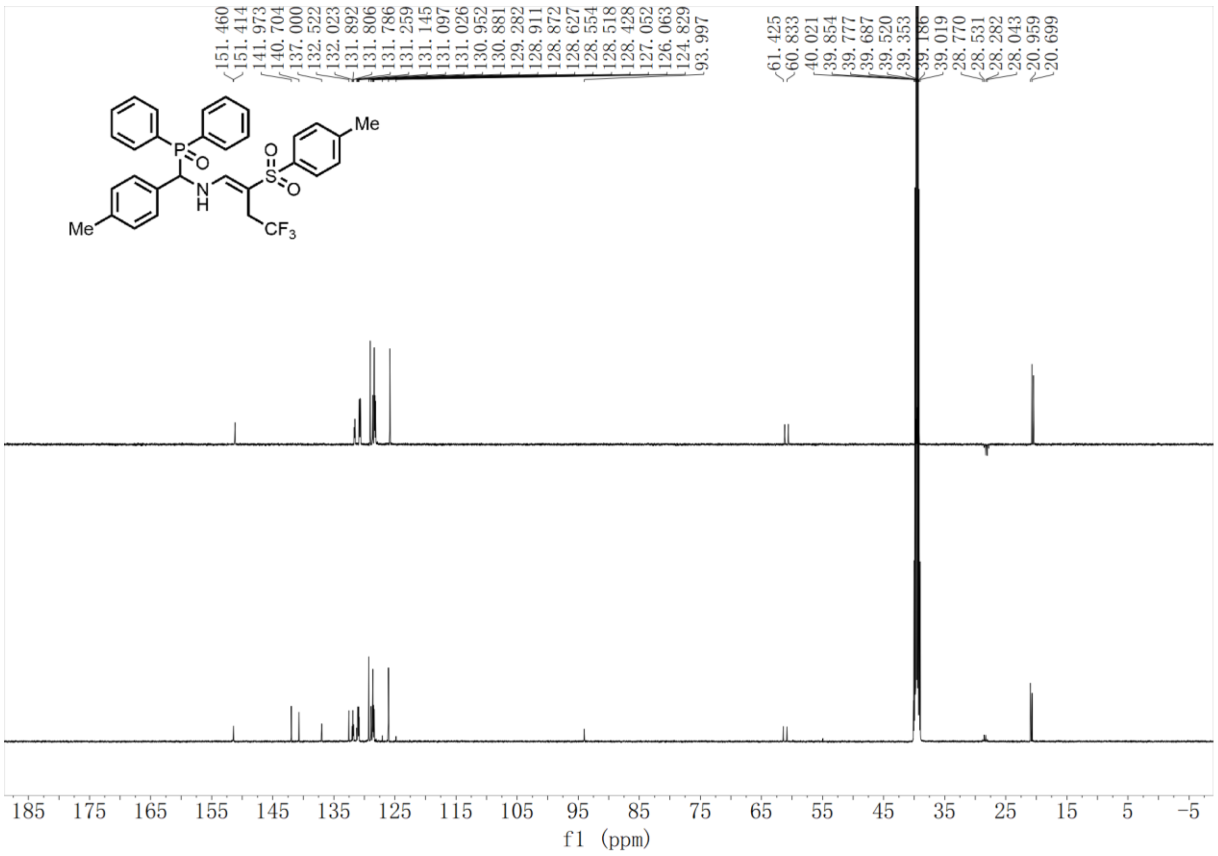

4b – ^{19}F NMR (282 MHz, DMSO)

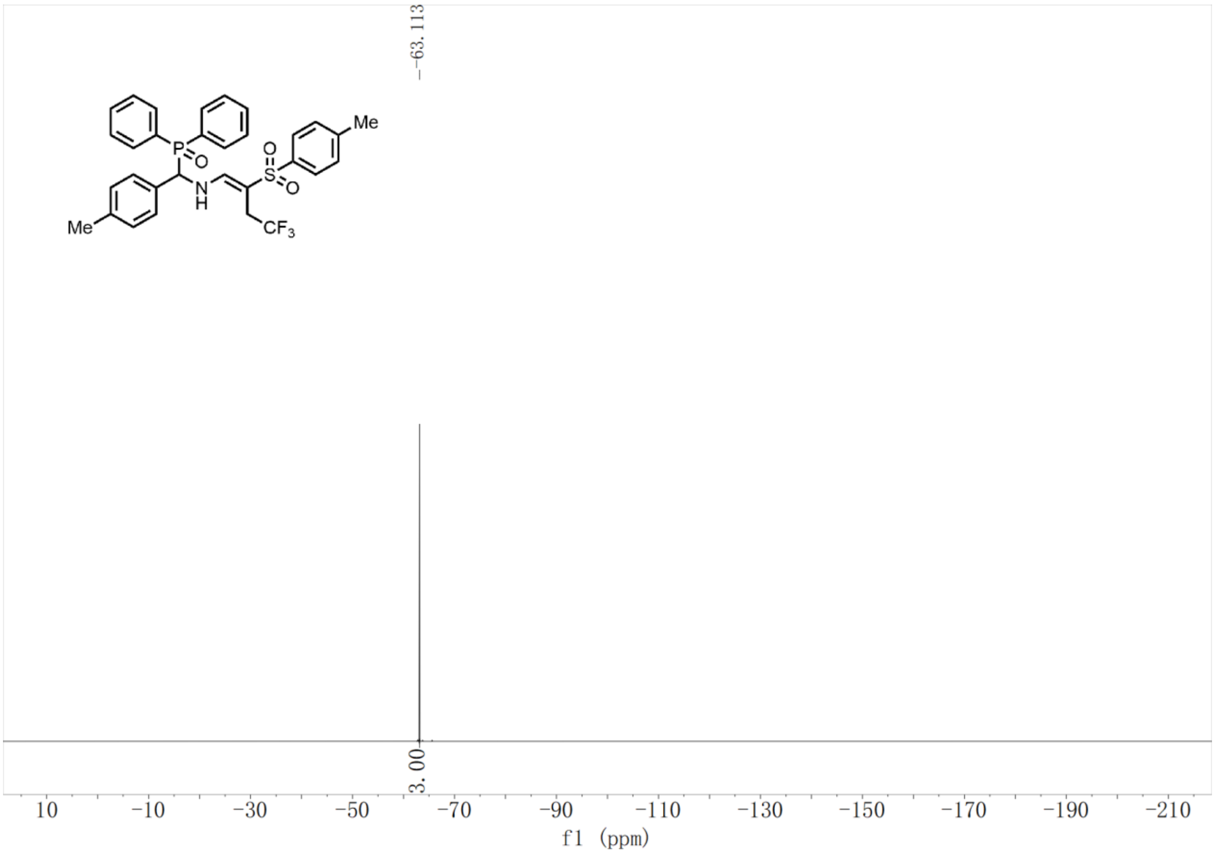

4b – ^{31}P NMR (121 MHz, DMSO)

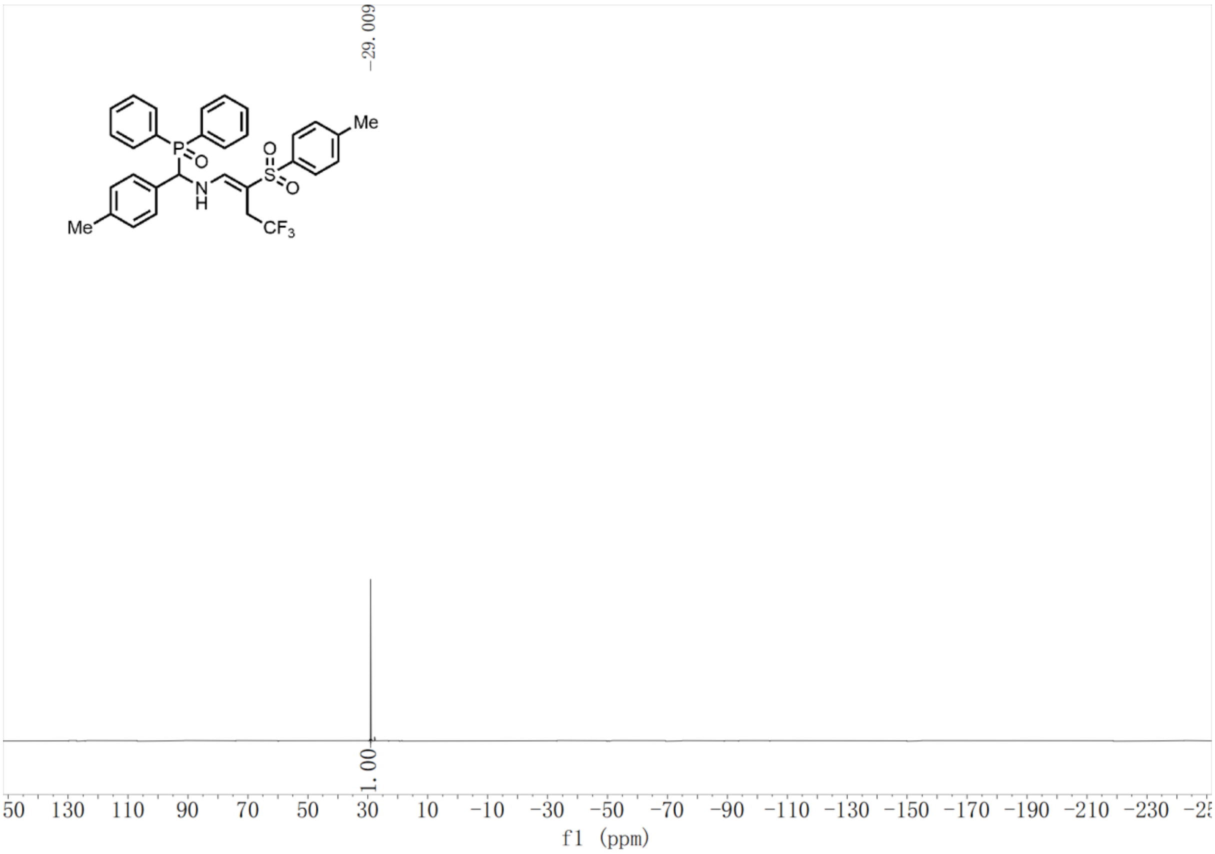

4c – ^{1}H NMR (500 MHz, DMSO)

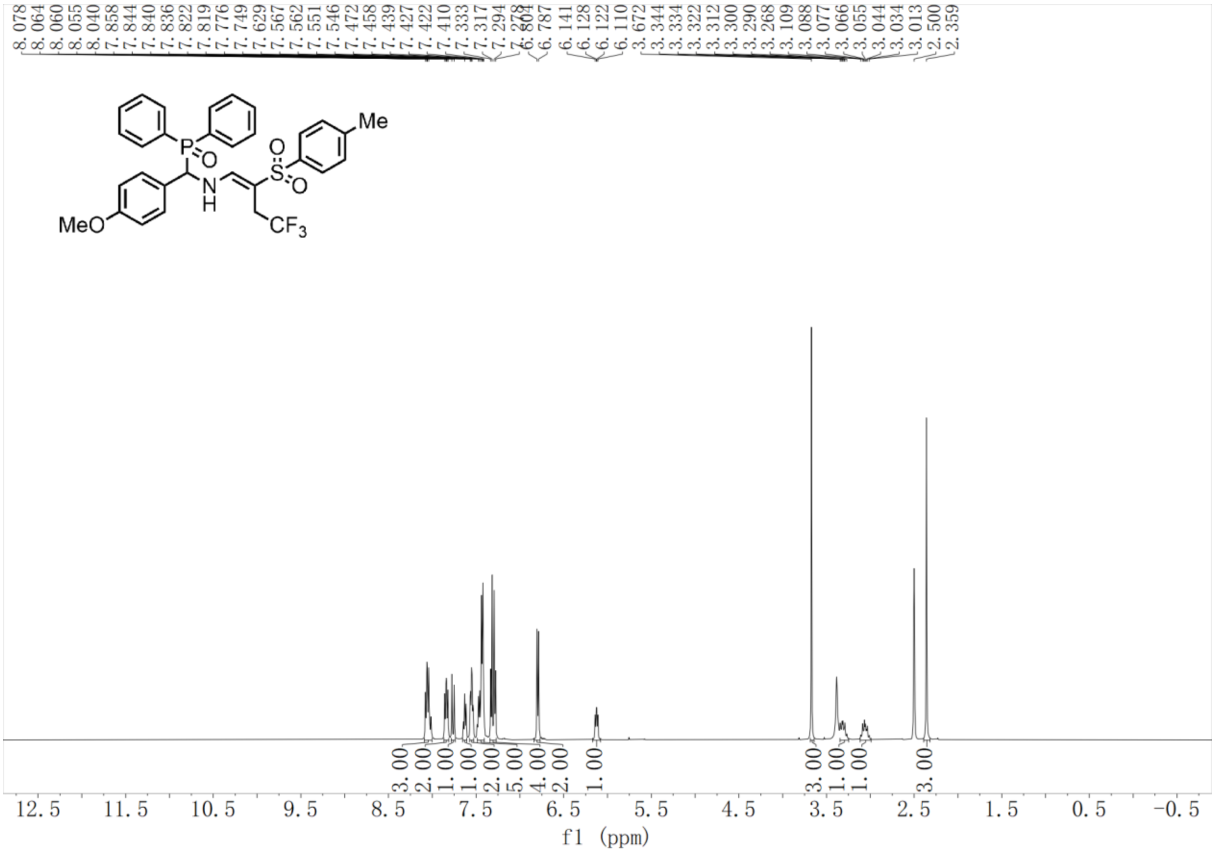

4c – ^{13}C NMR (126 MHz, DMSO)

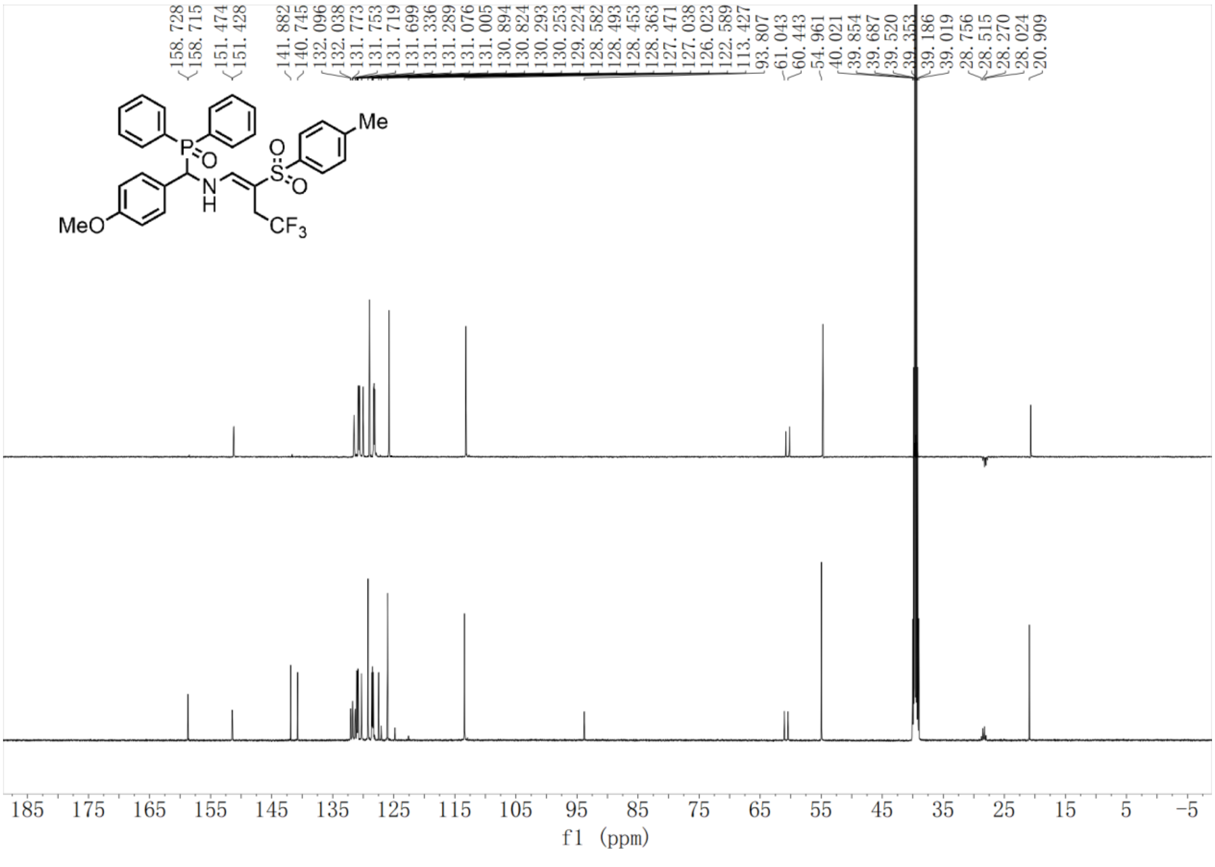

4c – ^{19}F NMR (471 MHz, DMSO)

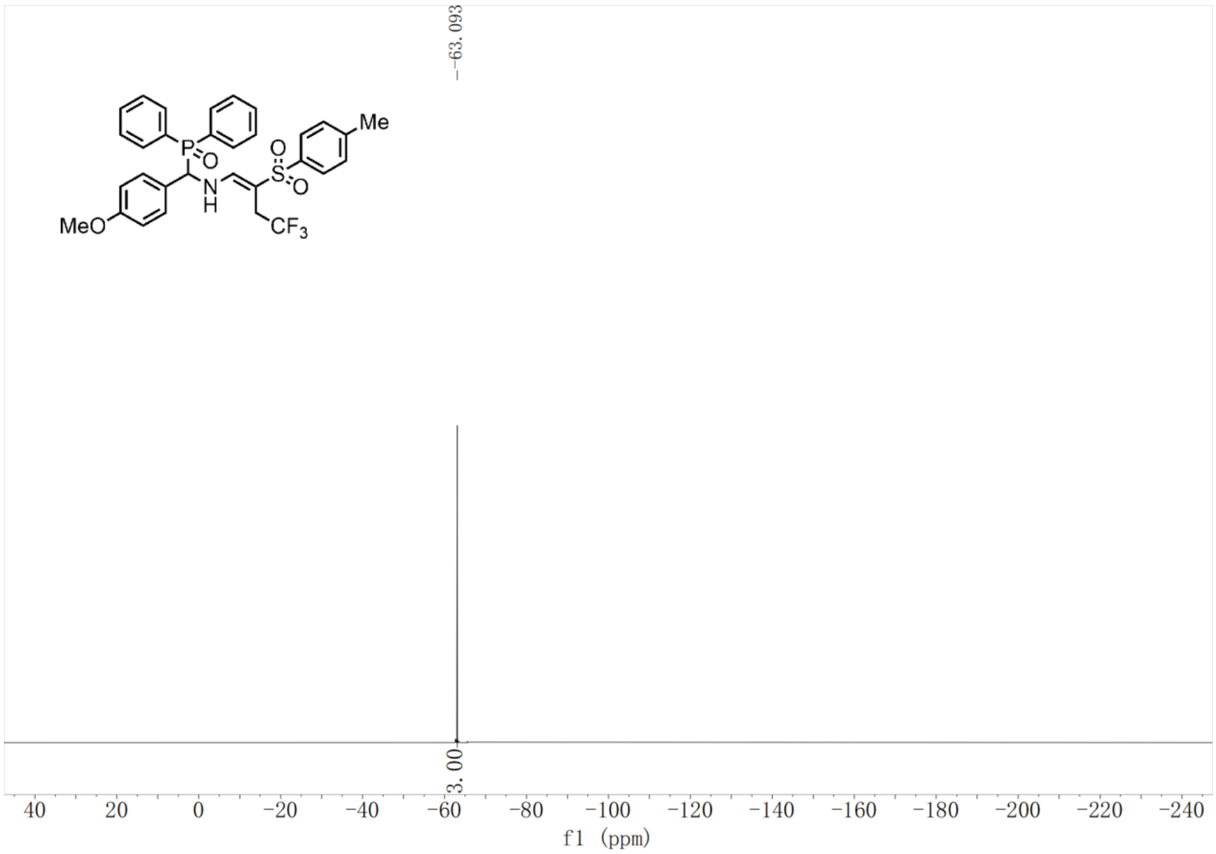

4c – ^{31}P NMR (202 MHz, DMSO)

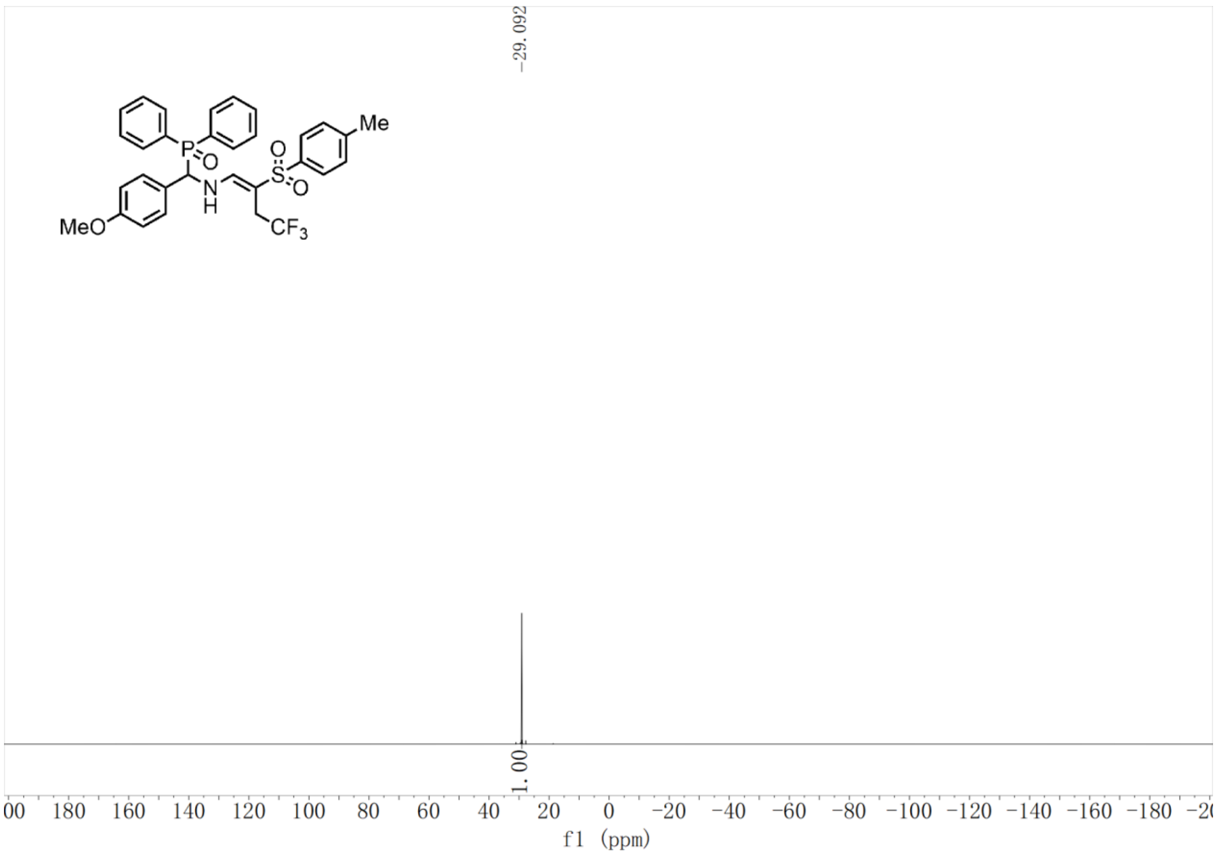

4d – ^{1}H NMR (500 MHz, DMSO)

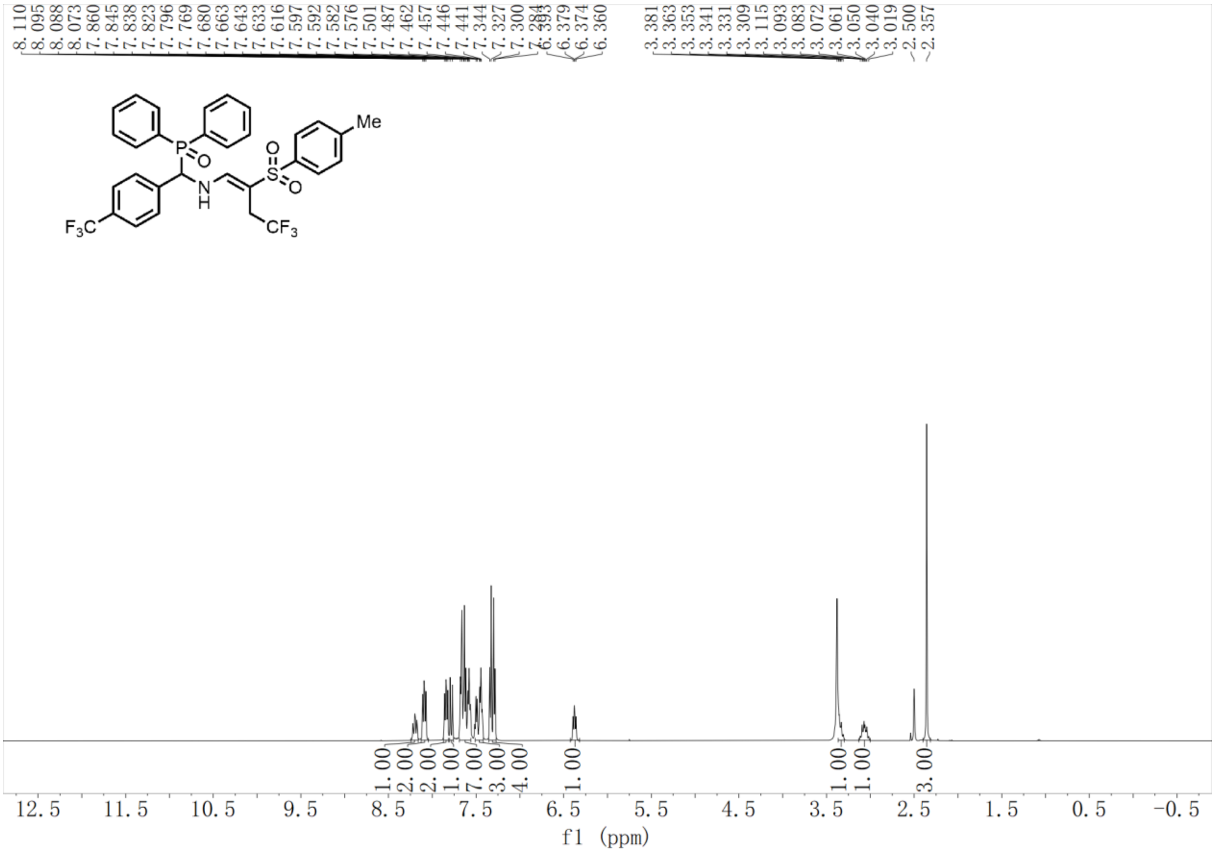

4d – ^{13}C NMR (126 MHz, DMSO)

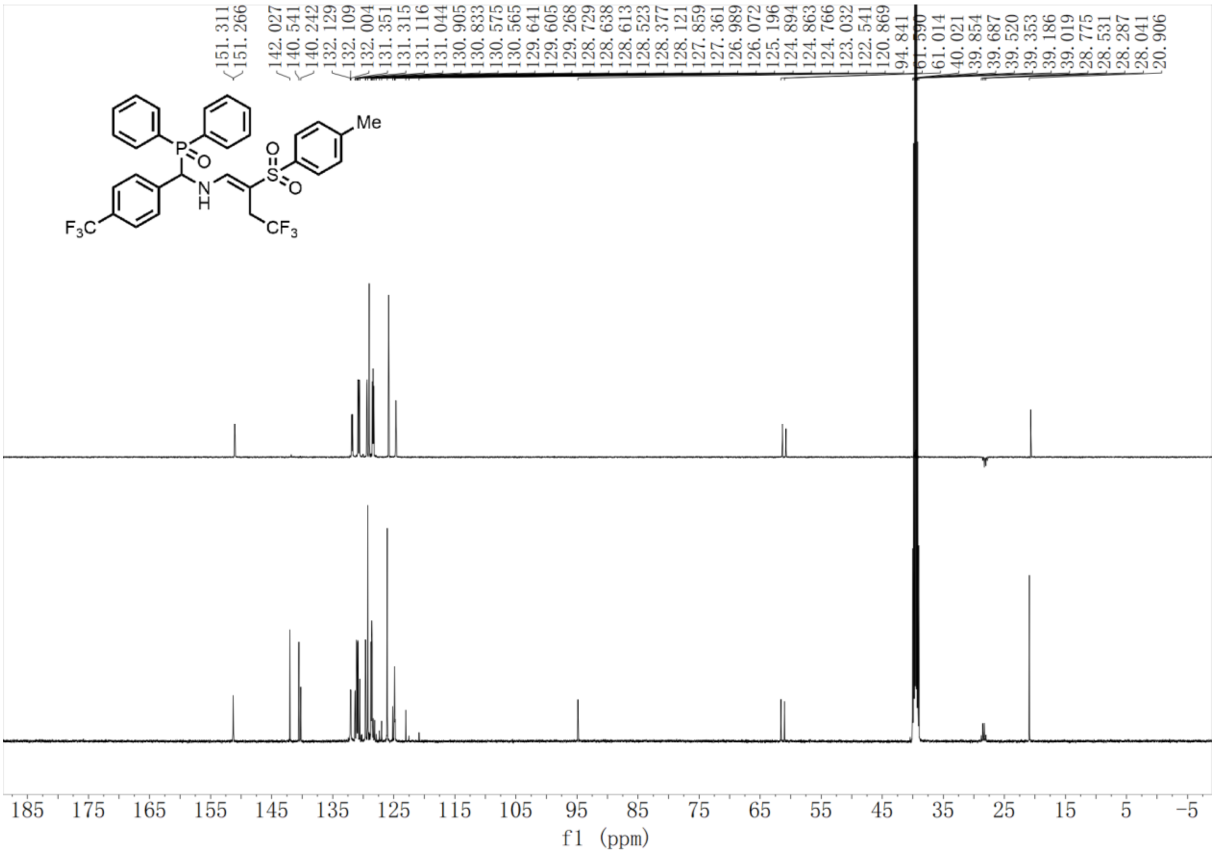

4d – ^{19}F NMR (471 MHz, DMSO)

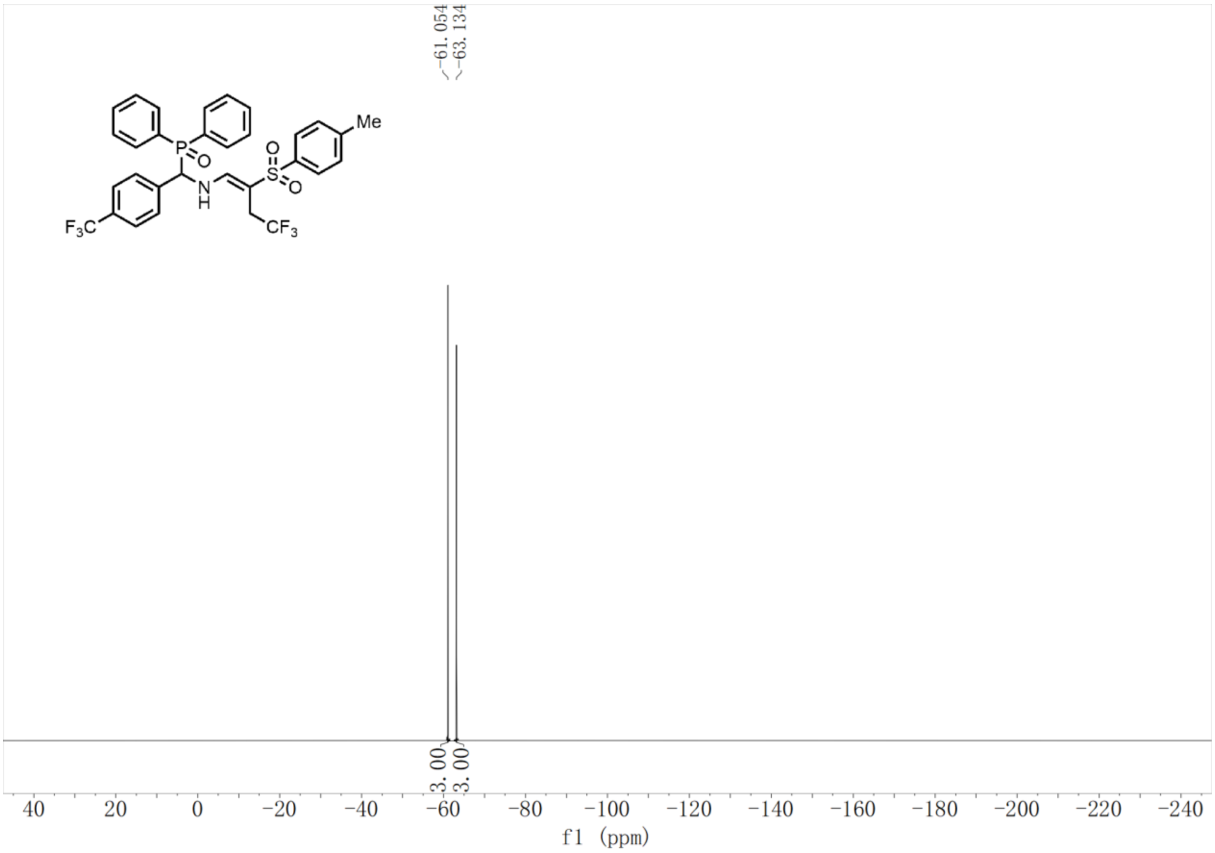

4d – ^{31}P NMR (202 MHz, DMSO)

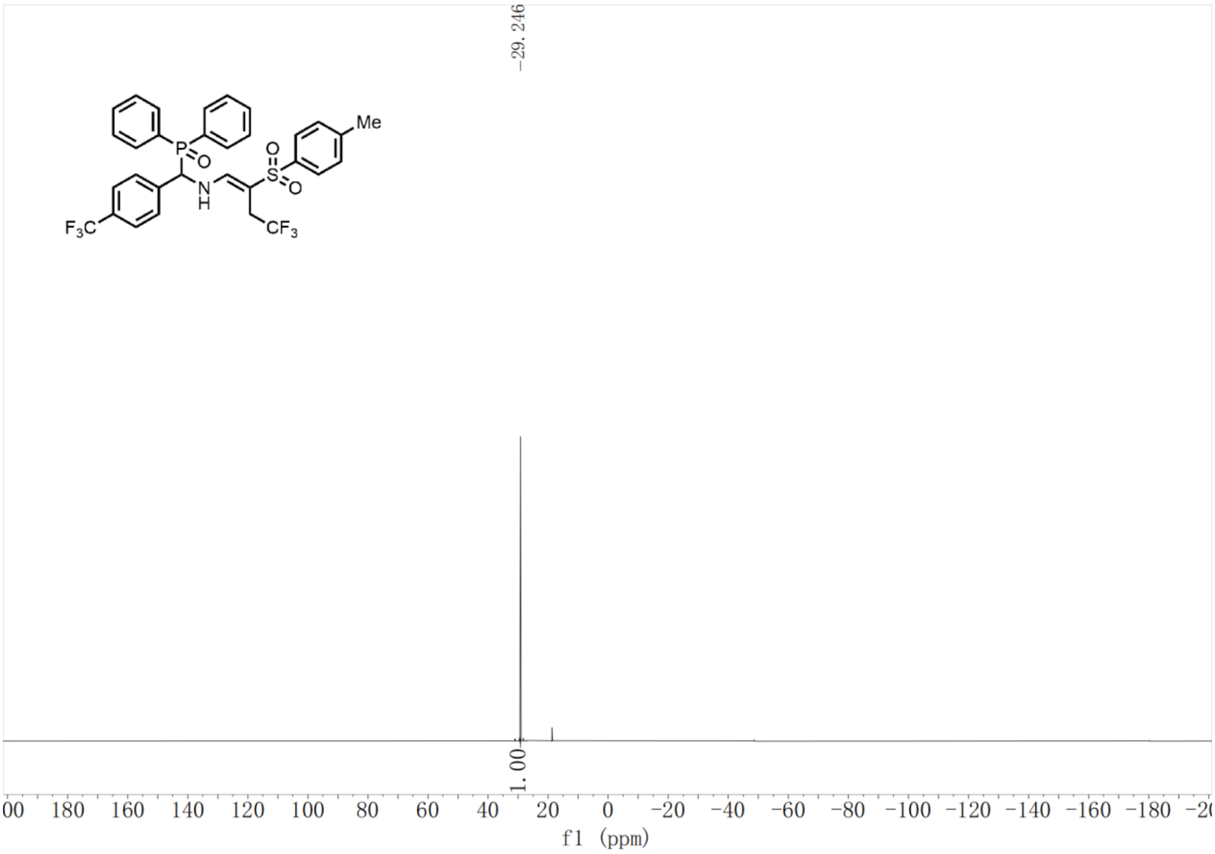

4e – ^{1}H NMR (500 MHz, DMSO)

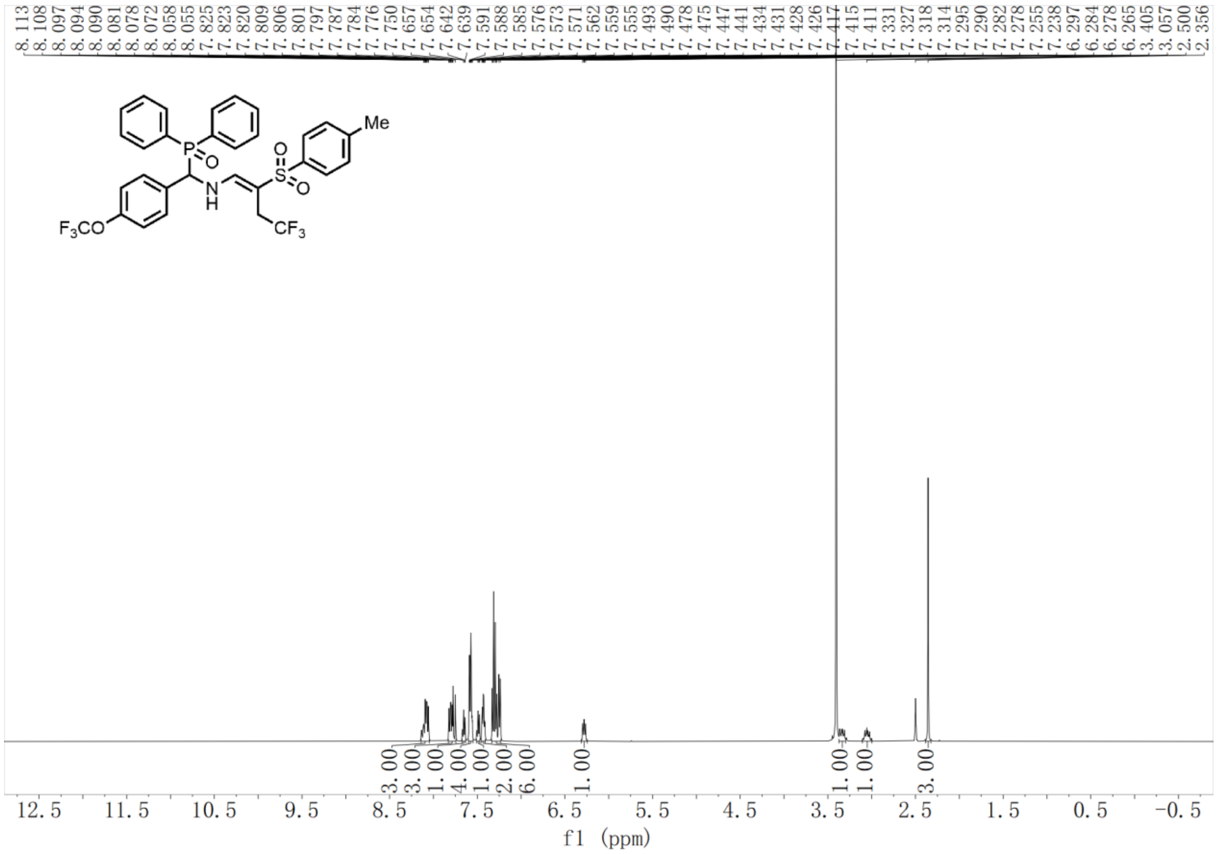

4e – ^{13}C NMR (126 MHz, DMSO)

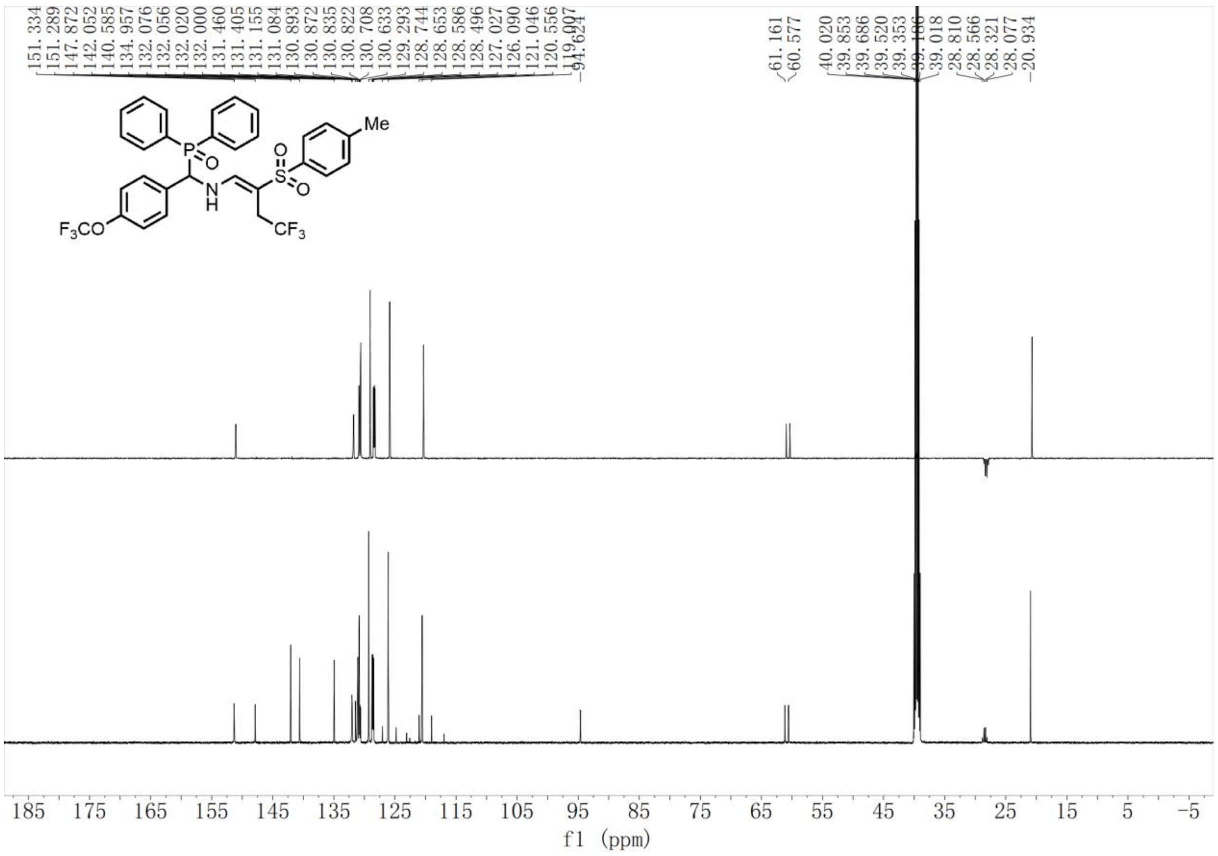

4e – ^{19}F NMR (282 MHz, DMSO)

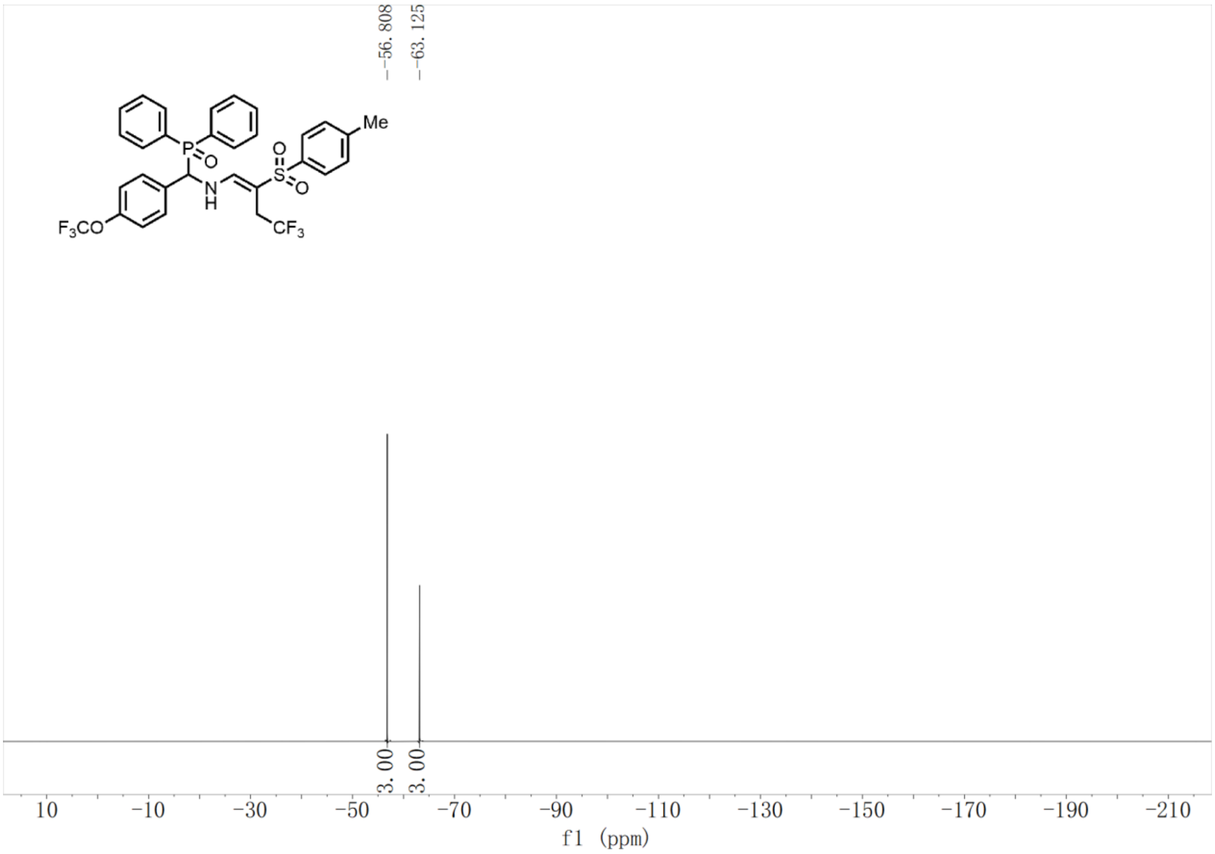

4e – ^{31}P NMR (121 MHz, DMSO)

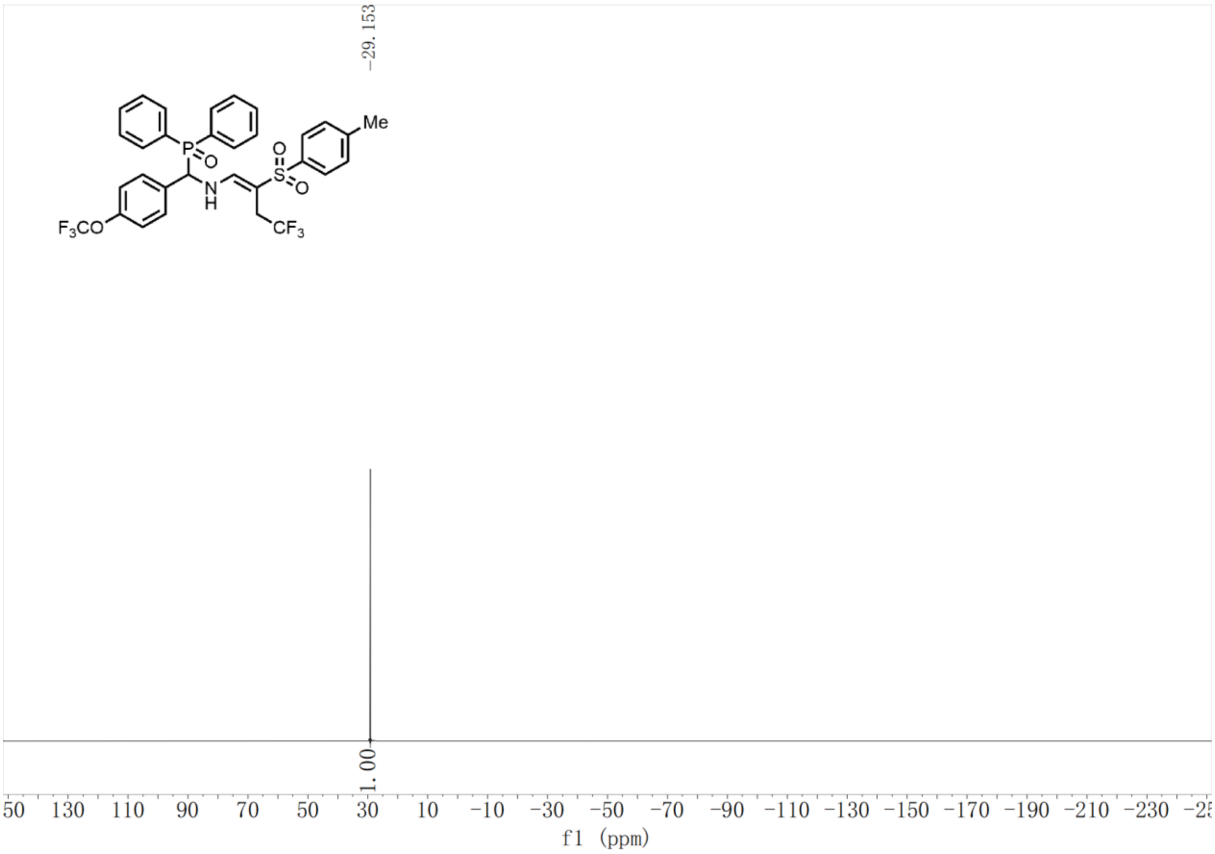

4f – ^{1}H NMR (500 MHz, DMSO)

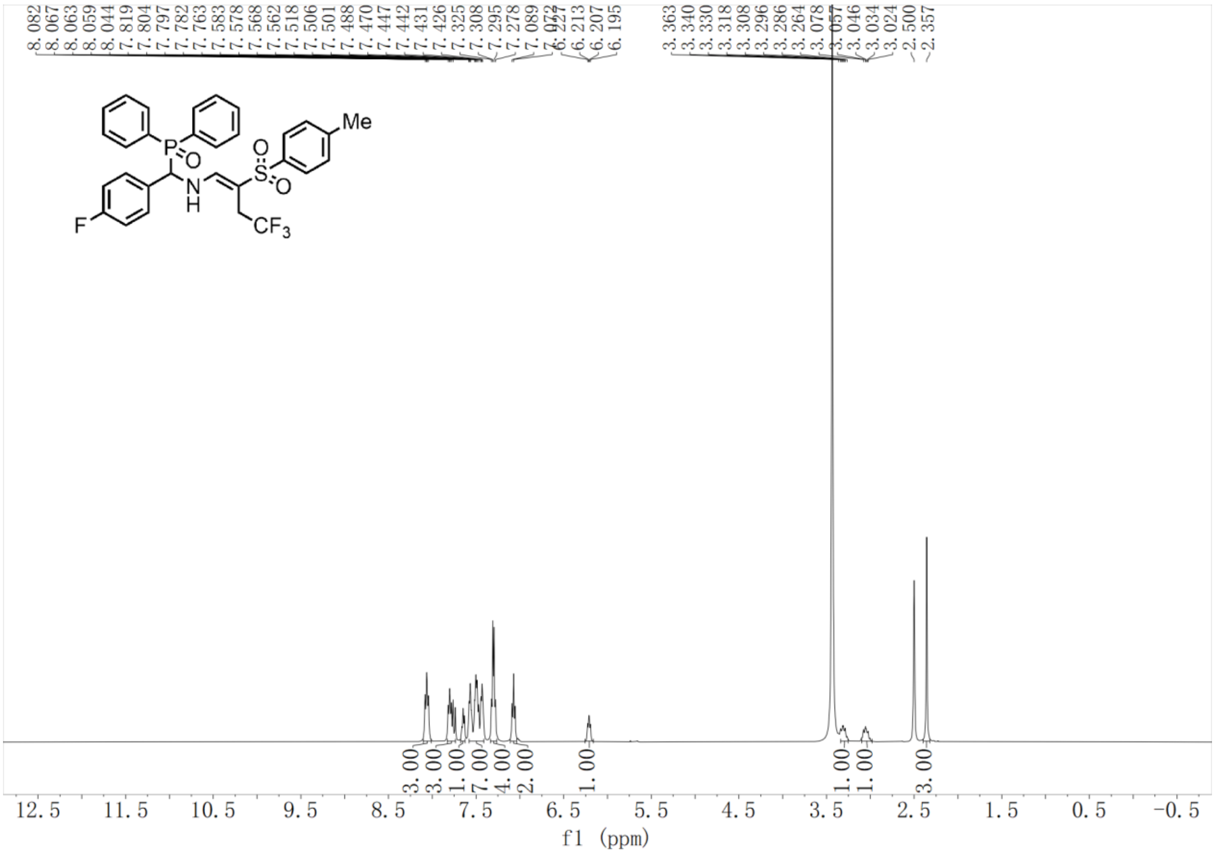

4f – ^{13}C NMR (126 MHz, DMSO)

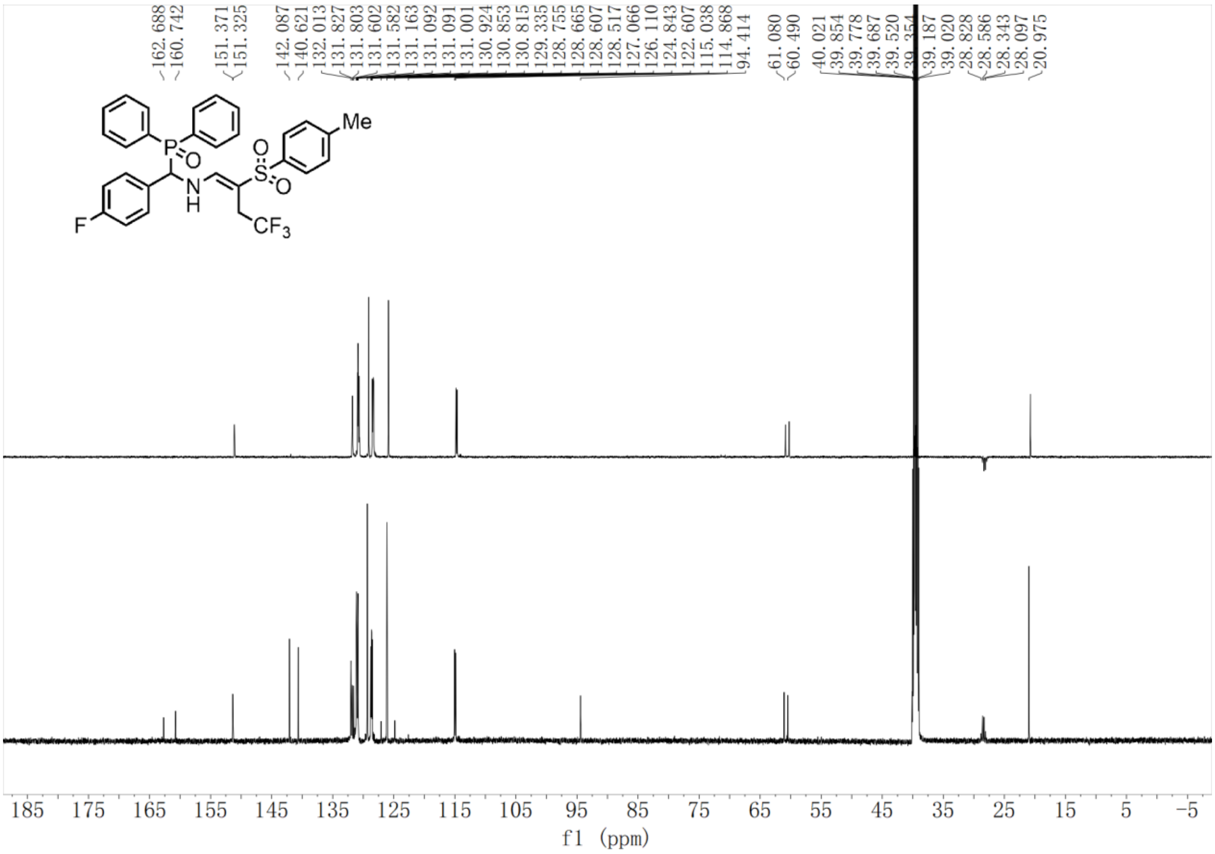

4f – ^{19}F NMR (471 MHz, DMSO)

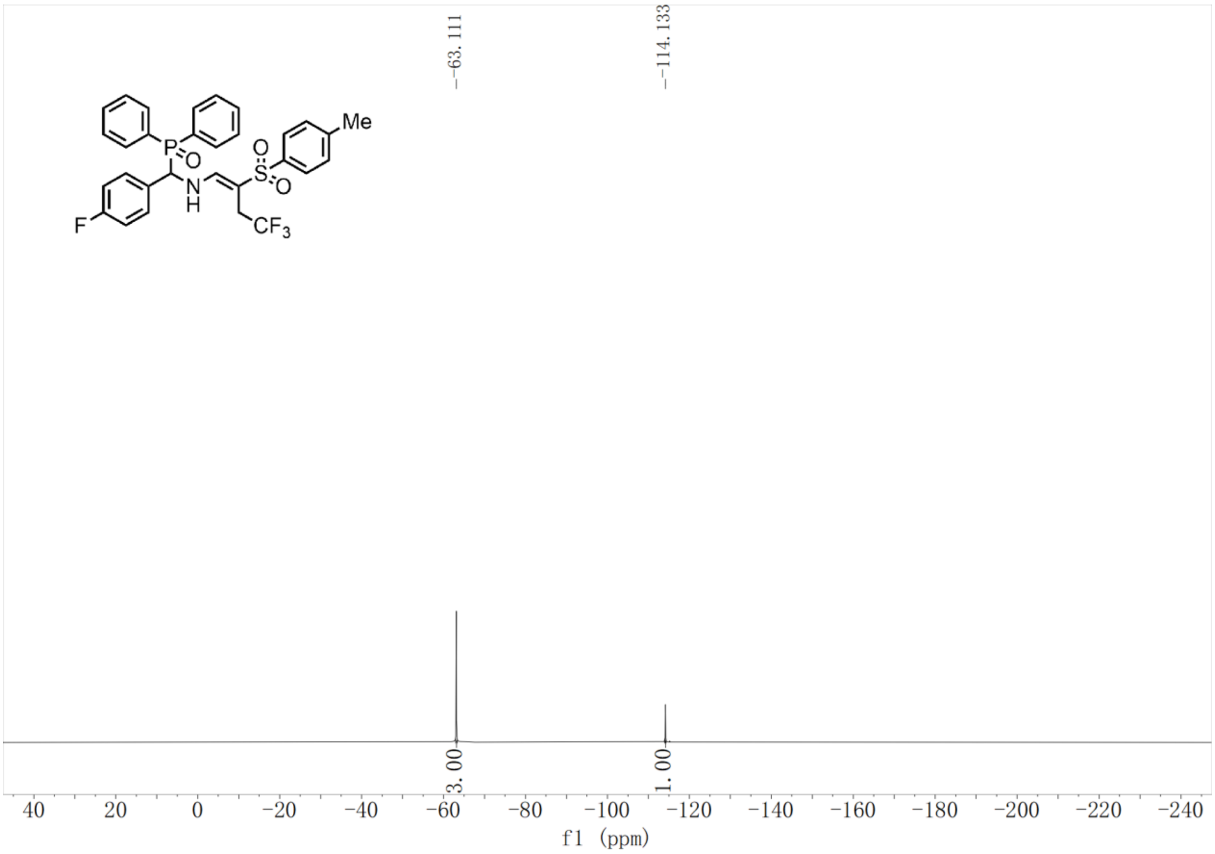

4f – ^{31}P NMR (121 MHz, DMSO)

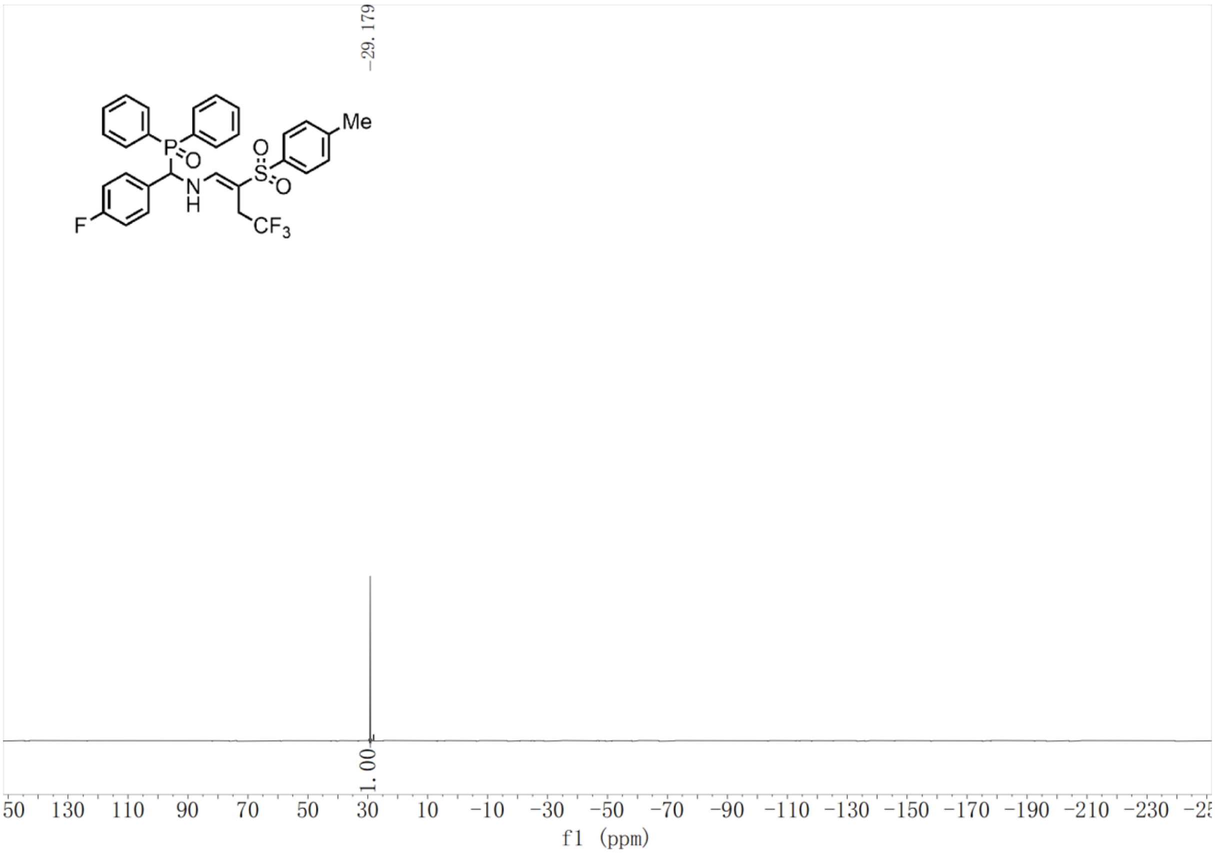

4g – ^{1}H NMR (500 MHz, DMSO)

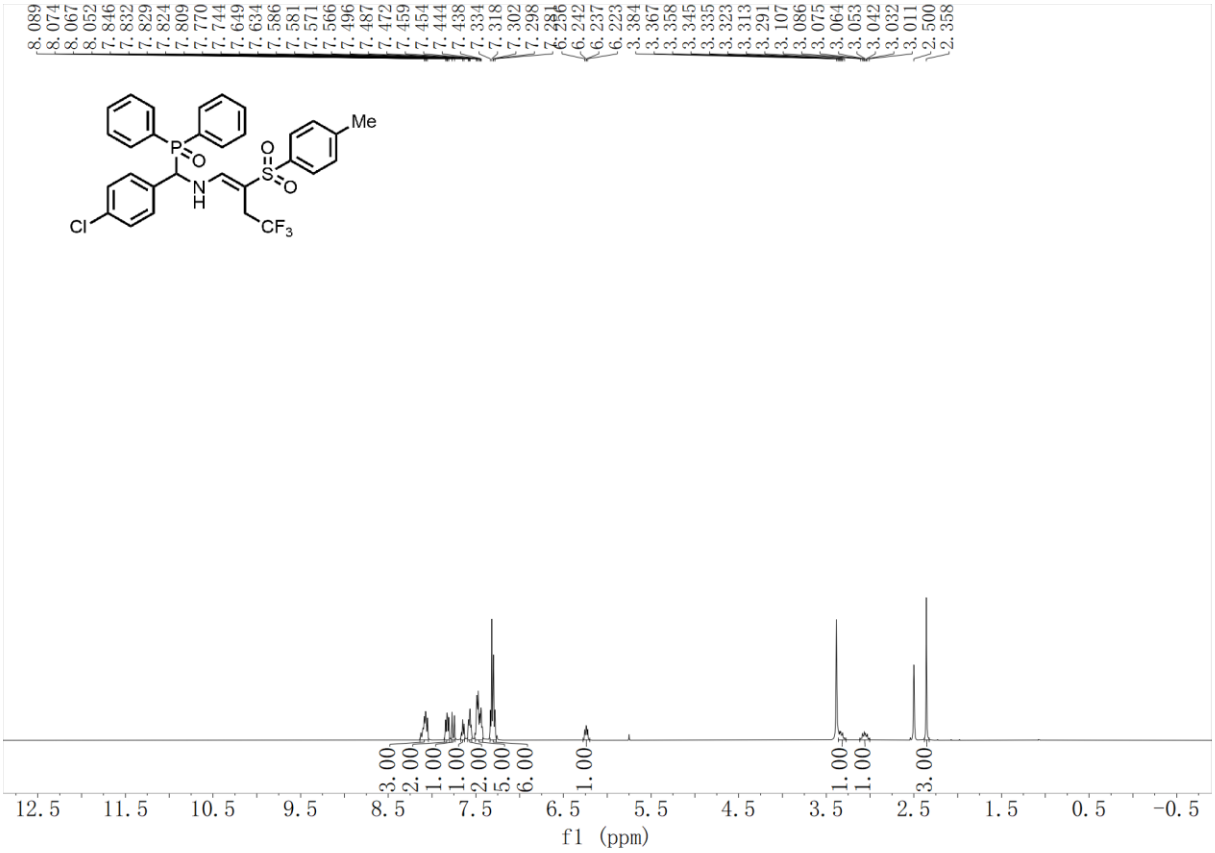

4g – ^{13}C NMR (126 MHz, DMSO)

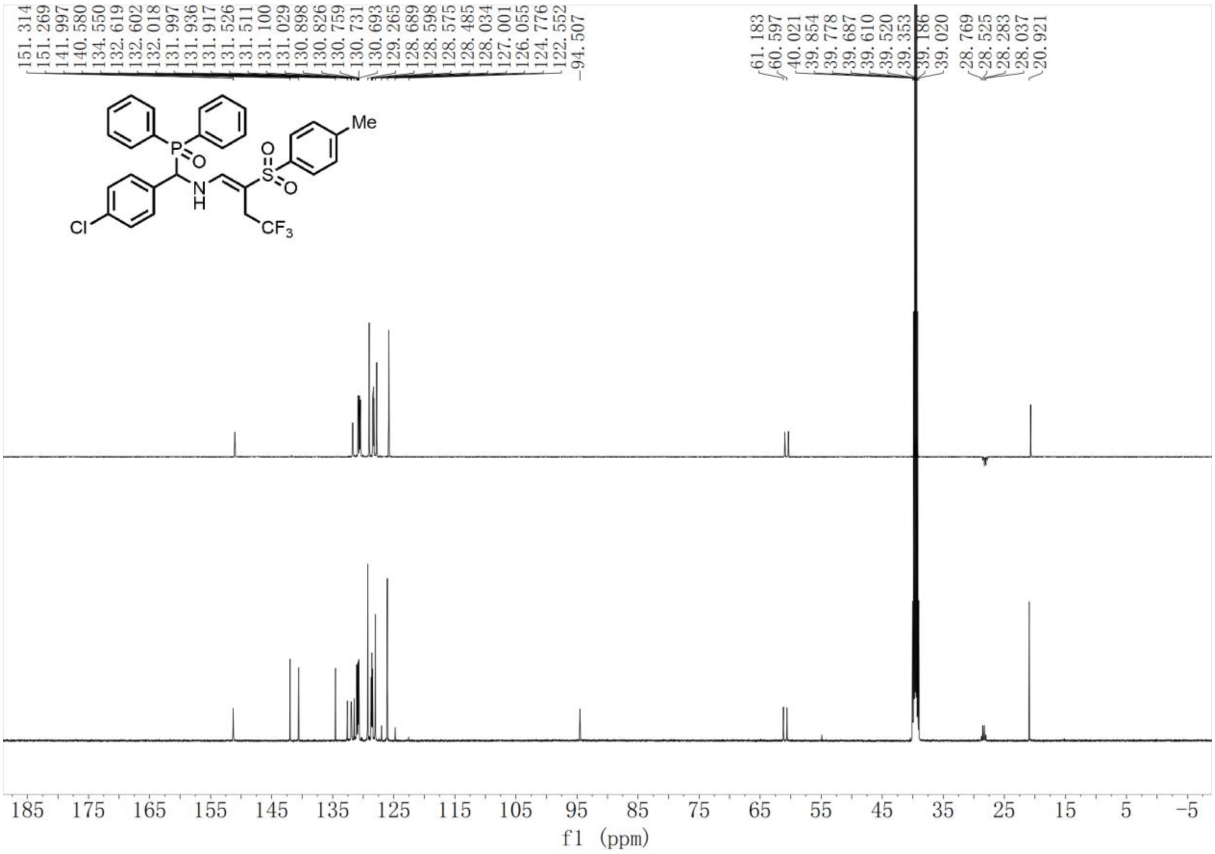

4g – ^{19}F NMR (471 MHz, DMSO)

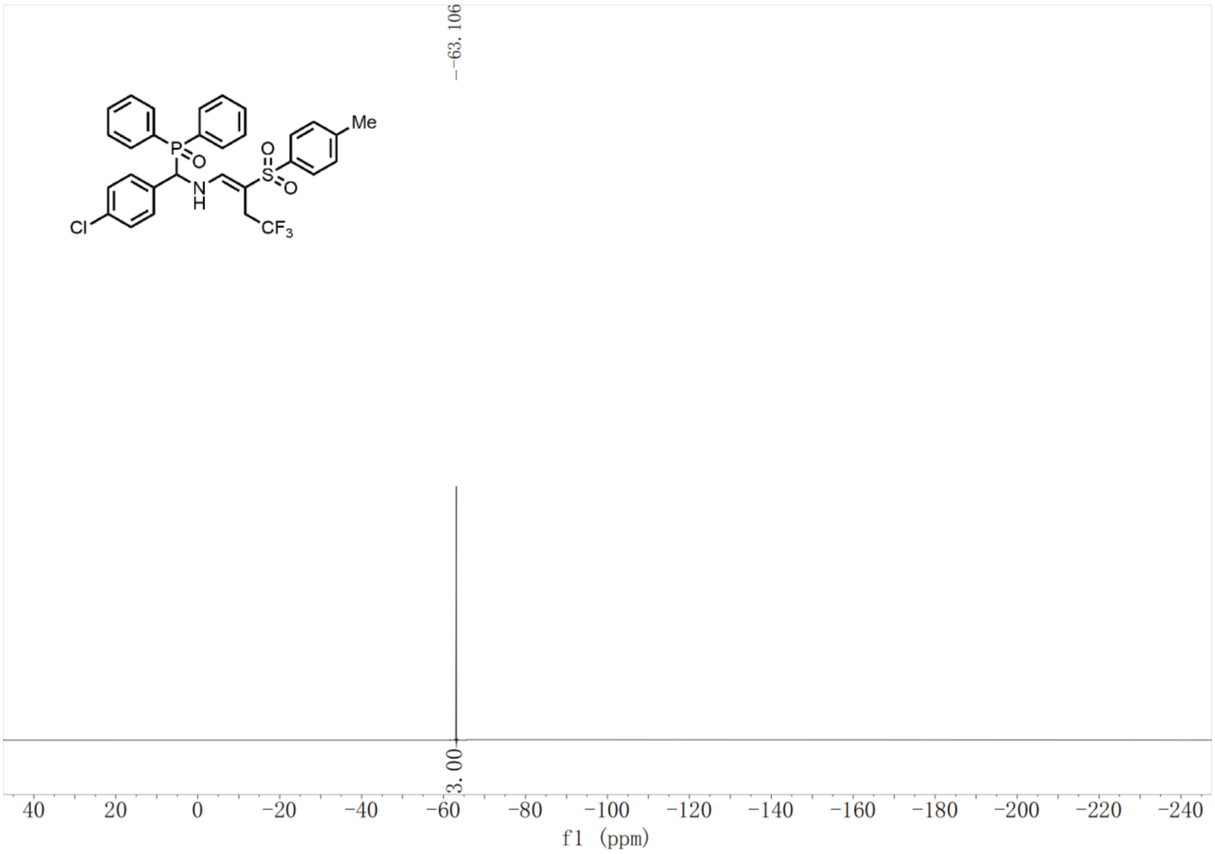

4g – ^{31}P NMR (202 MHz, DMSO)

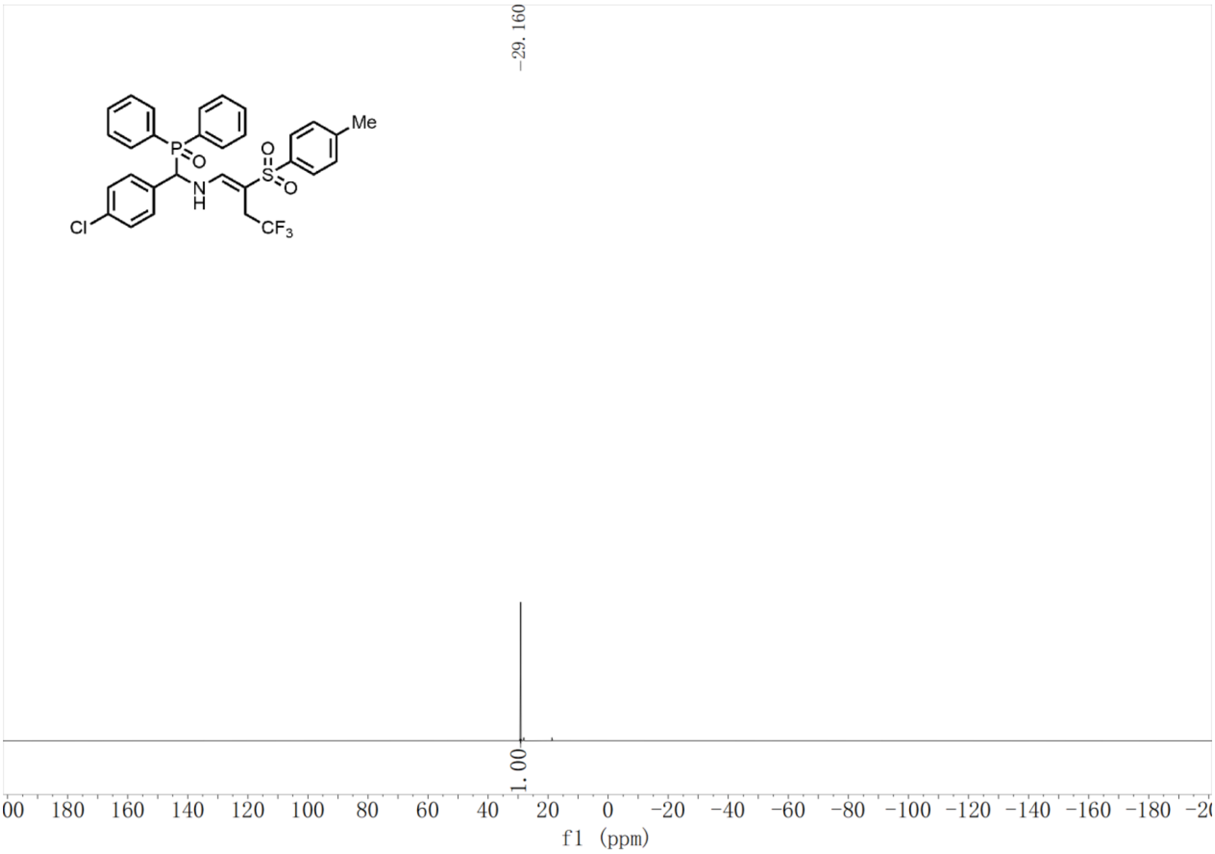

4h – ^{1}H NMR (500 MHz, DMSO)

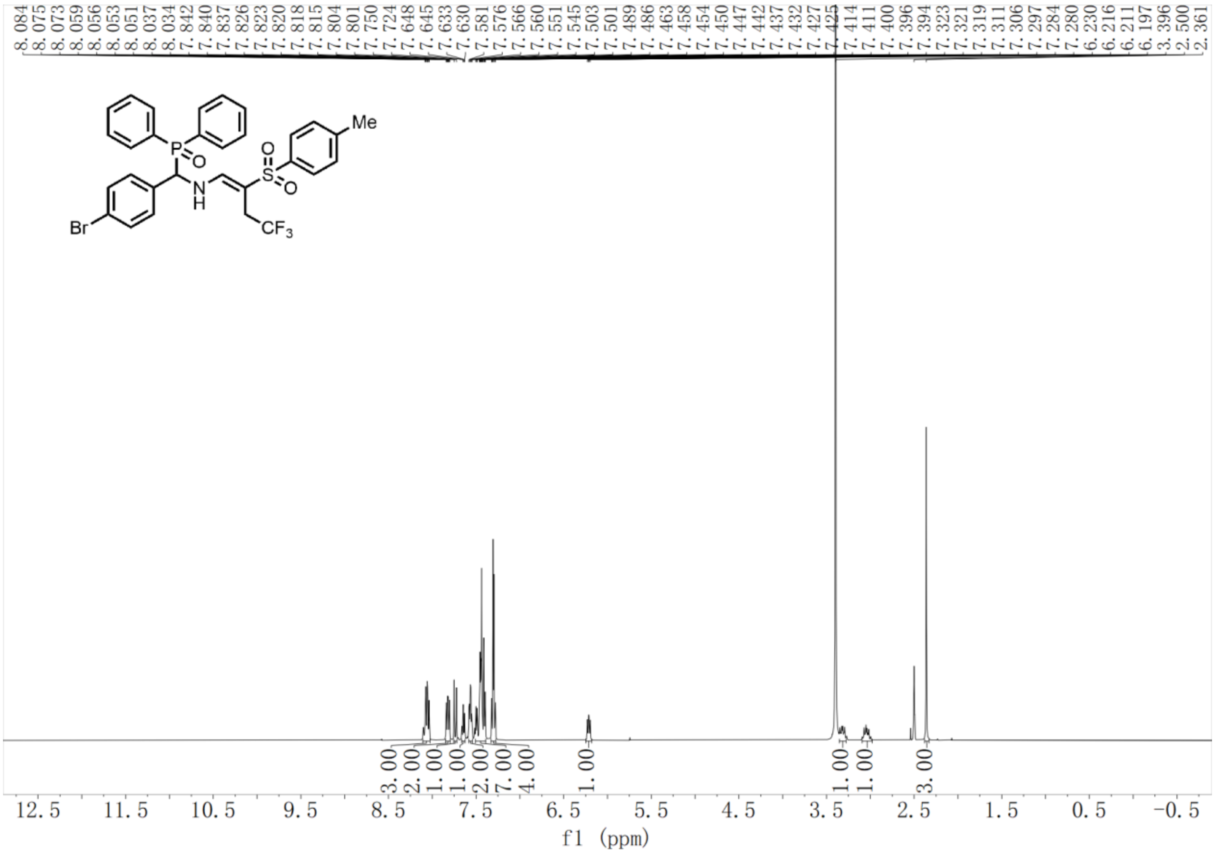

4h – ^{13}C NMR (126 MHz, DMSO)

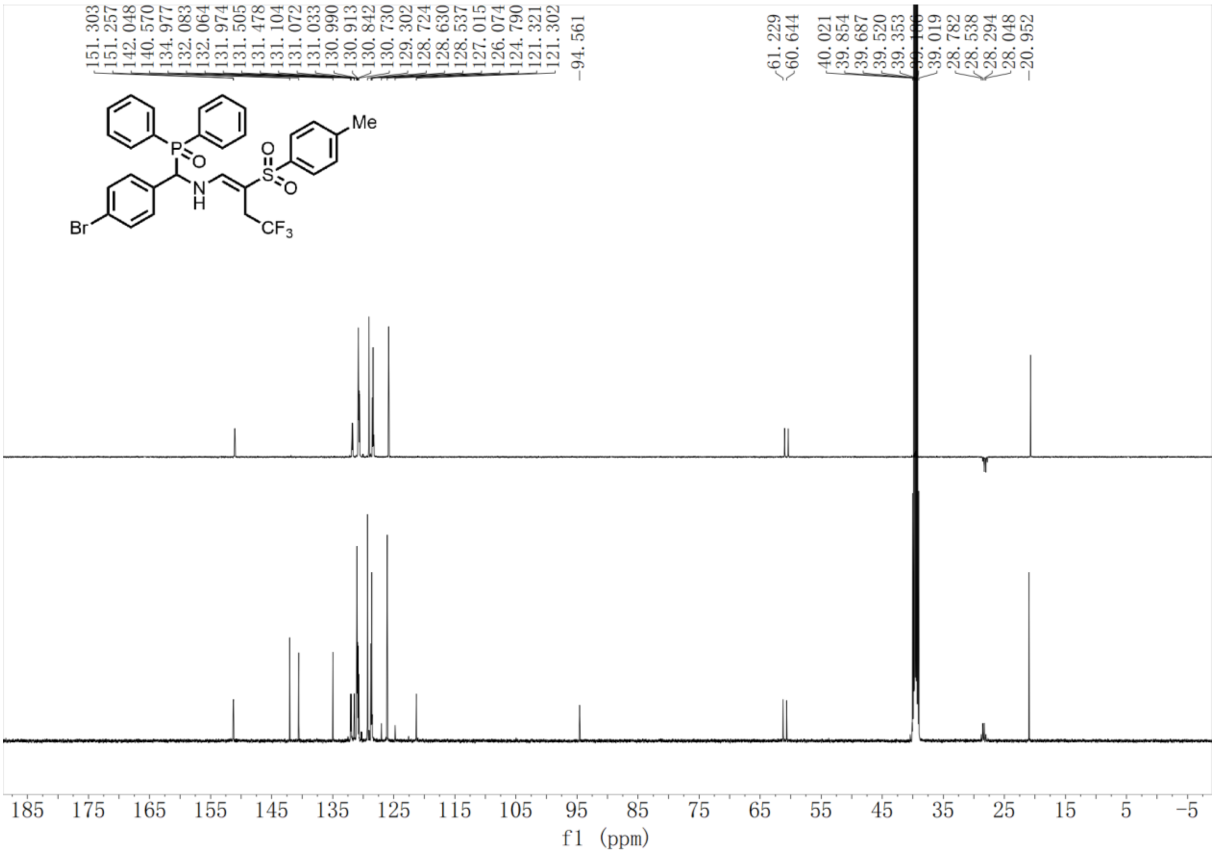

4h – ^{19}F NMR (282 MHz, DMSO)

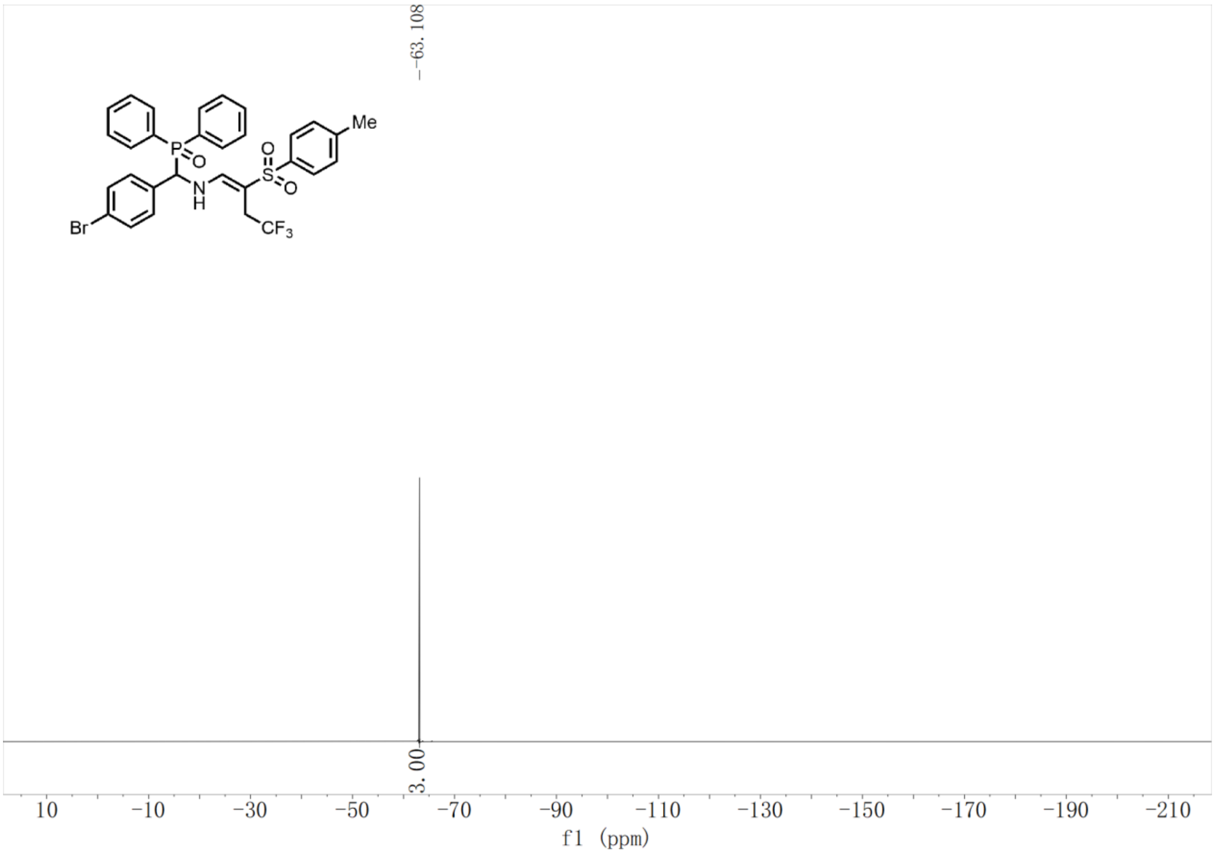

4h – ^{31}P NMR (121 MHz, DMSO)

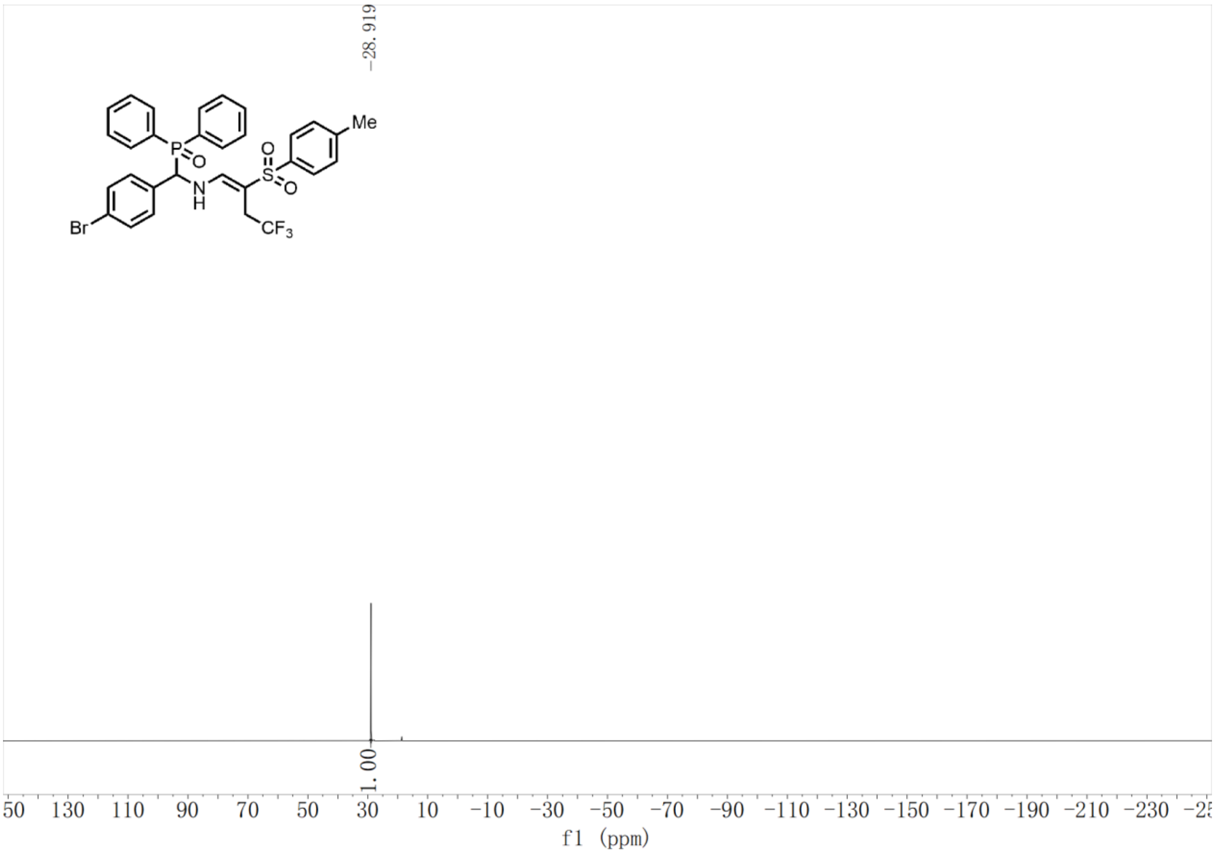

4i – ^{1}H NMR (500 MHz, DMSO)

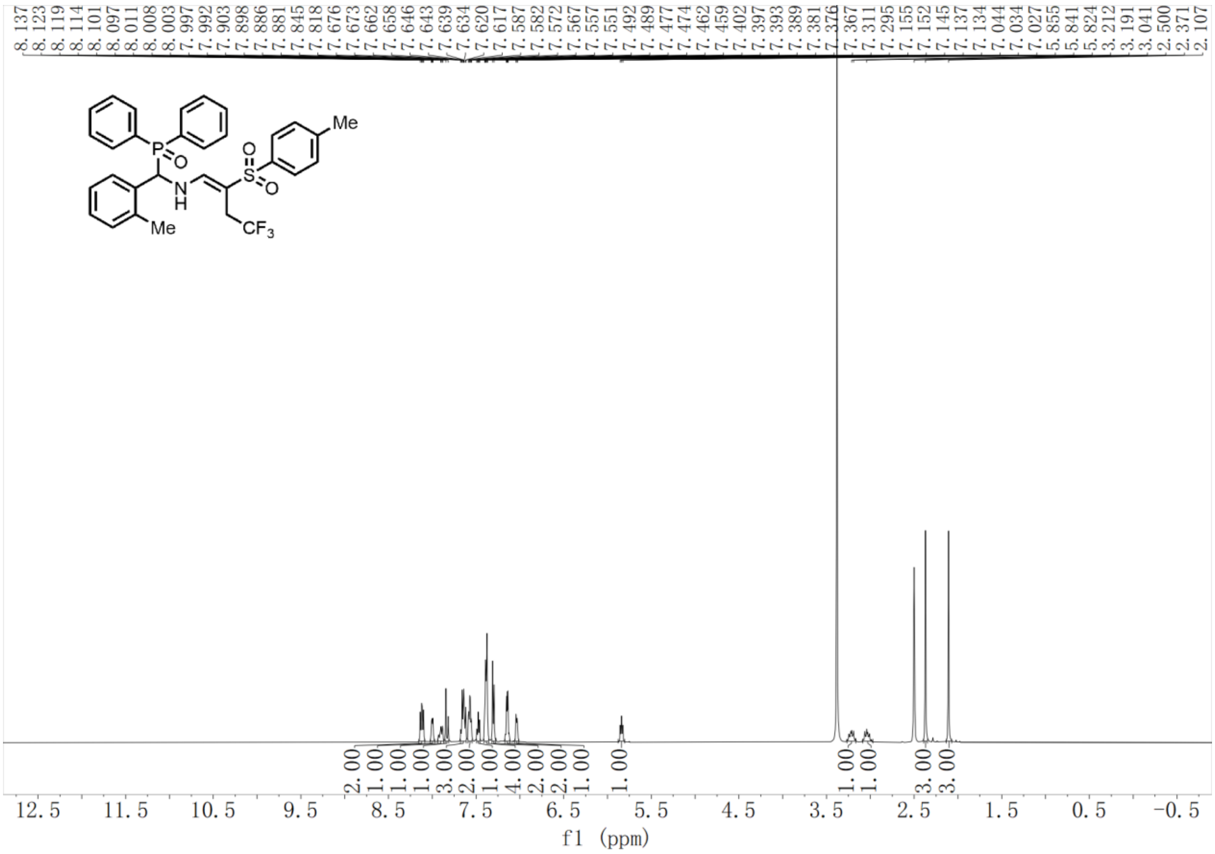

4i – ^{13}C NMR (126 MHz, DMSO)

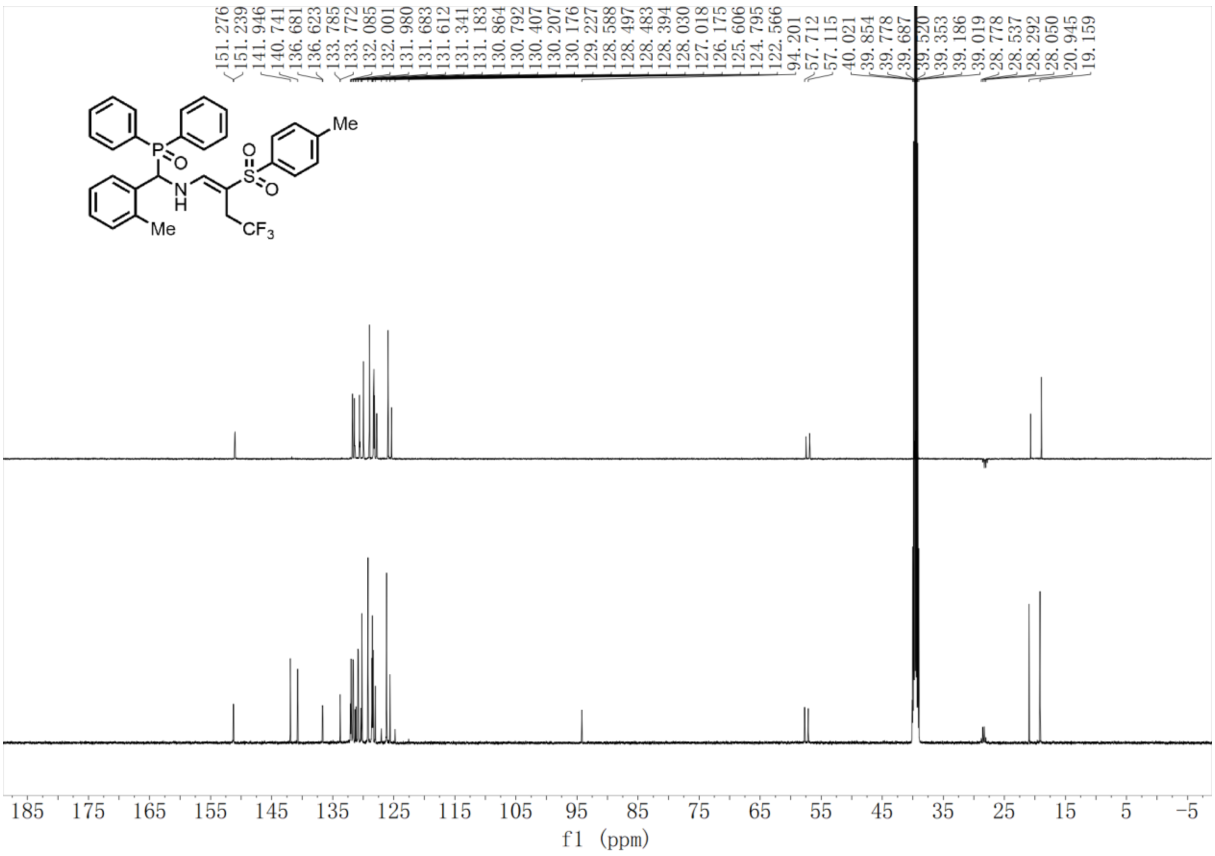

4i – ^{19}F NMR (471 MHz, DMSO)

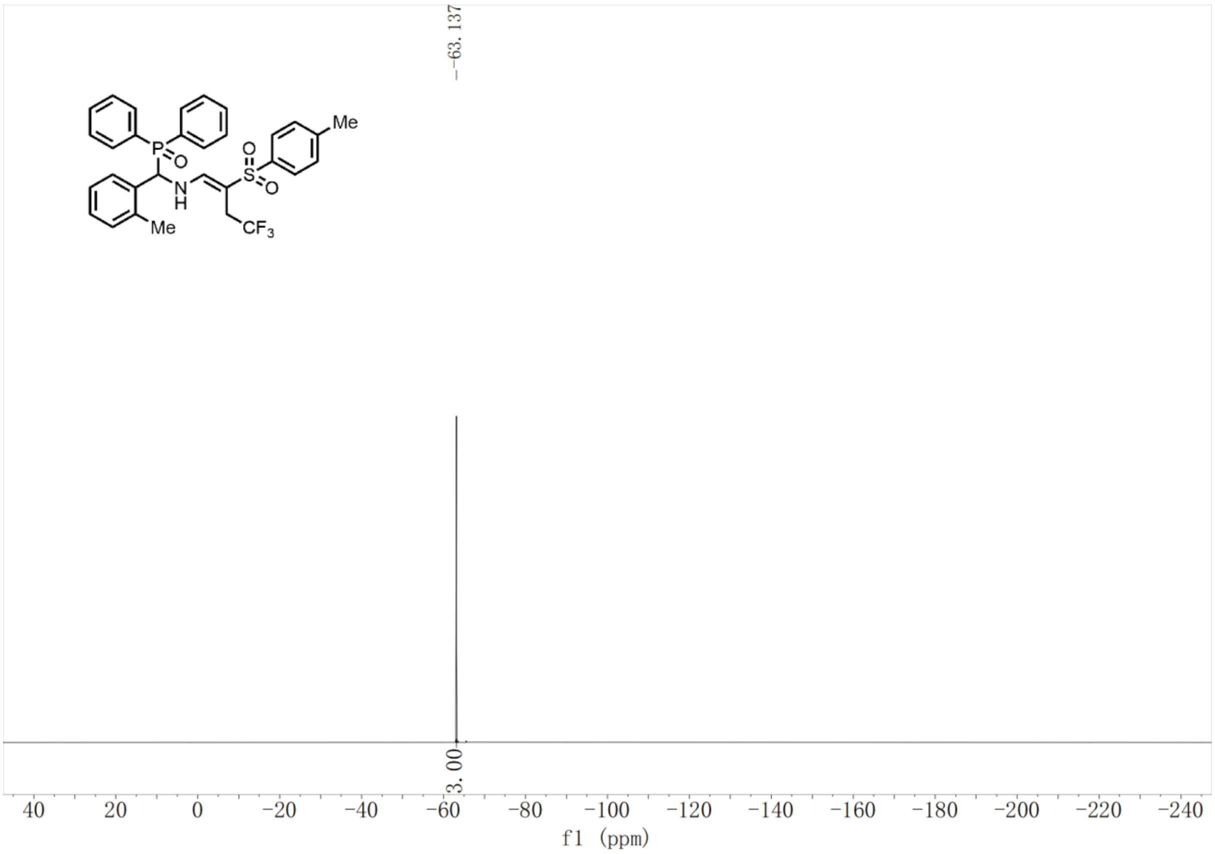

4i – ^{31}P NMR (202 MHz, DMSO)

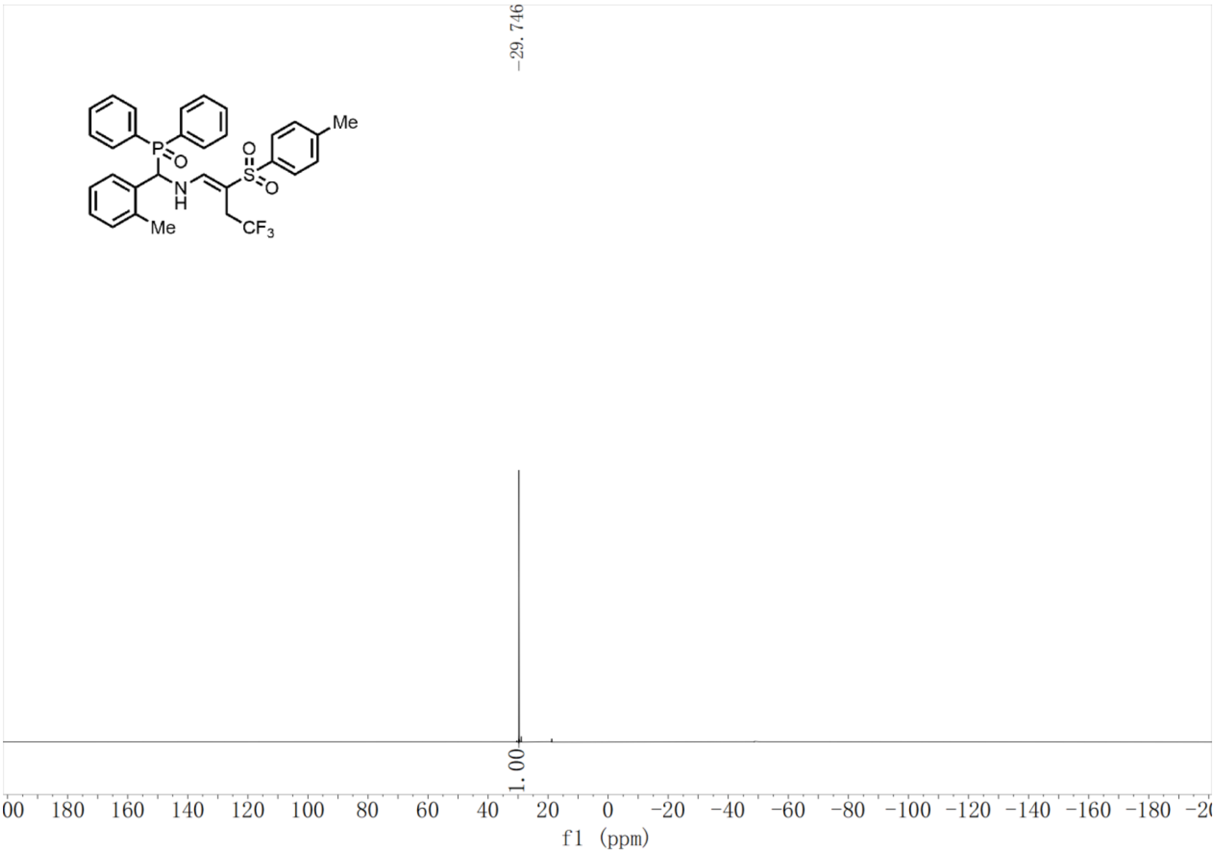

4j – ^{1}H NMR (500 MHz, DMSO)

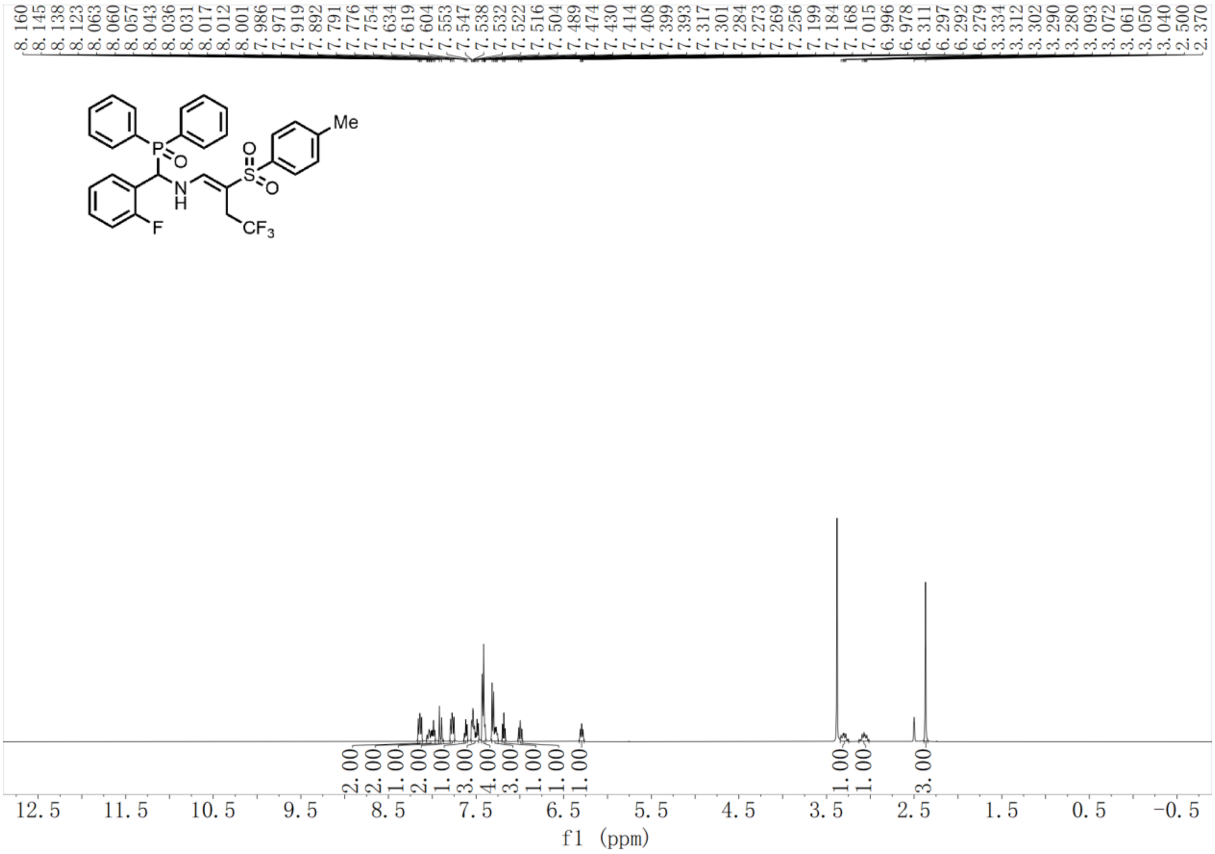

4j – ^{13}C NMR (126 MHz, DMSO)

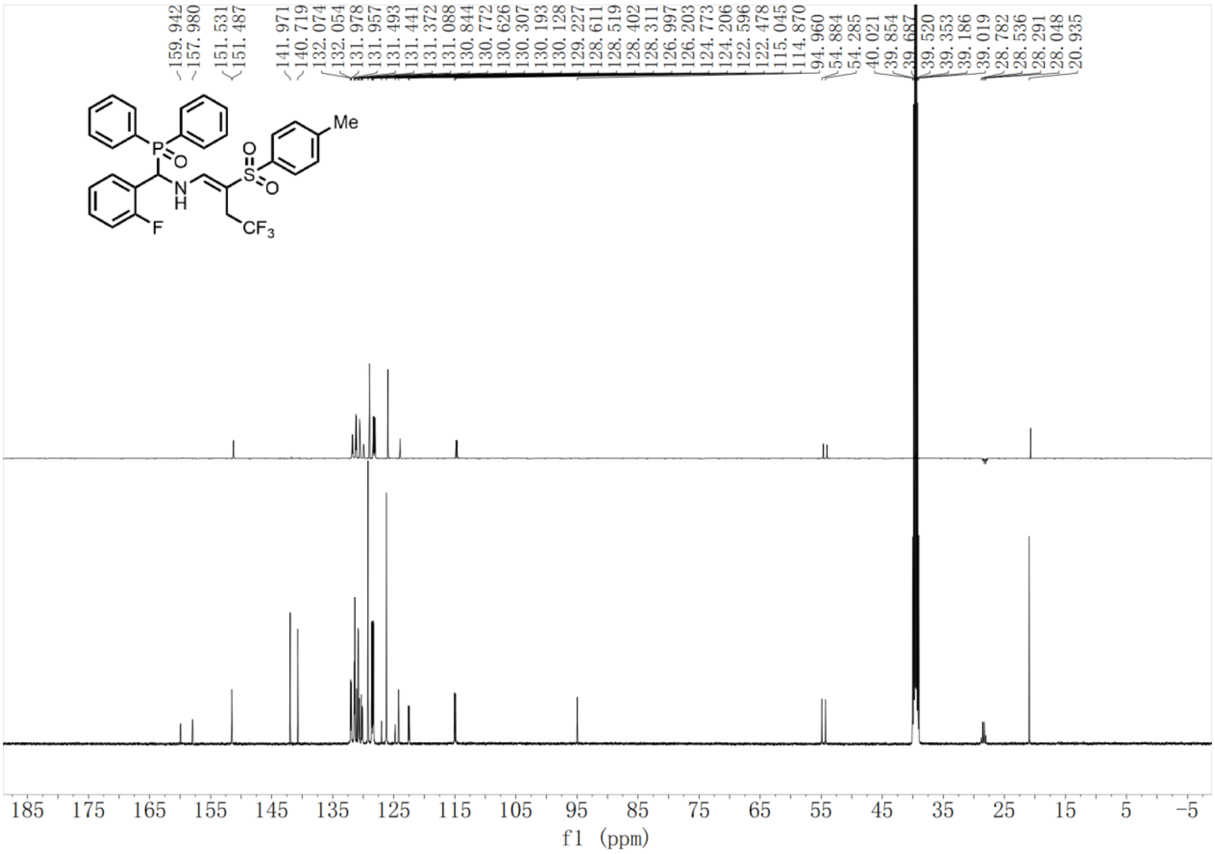

4j – ^{19}F NMR (471 MHz, DMSO)

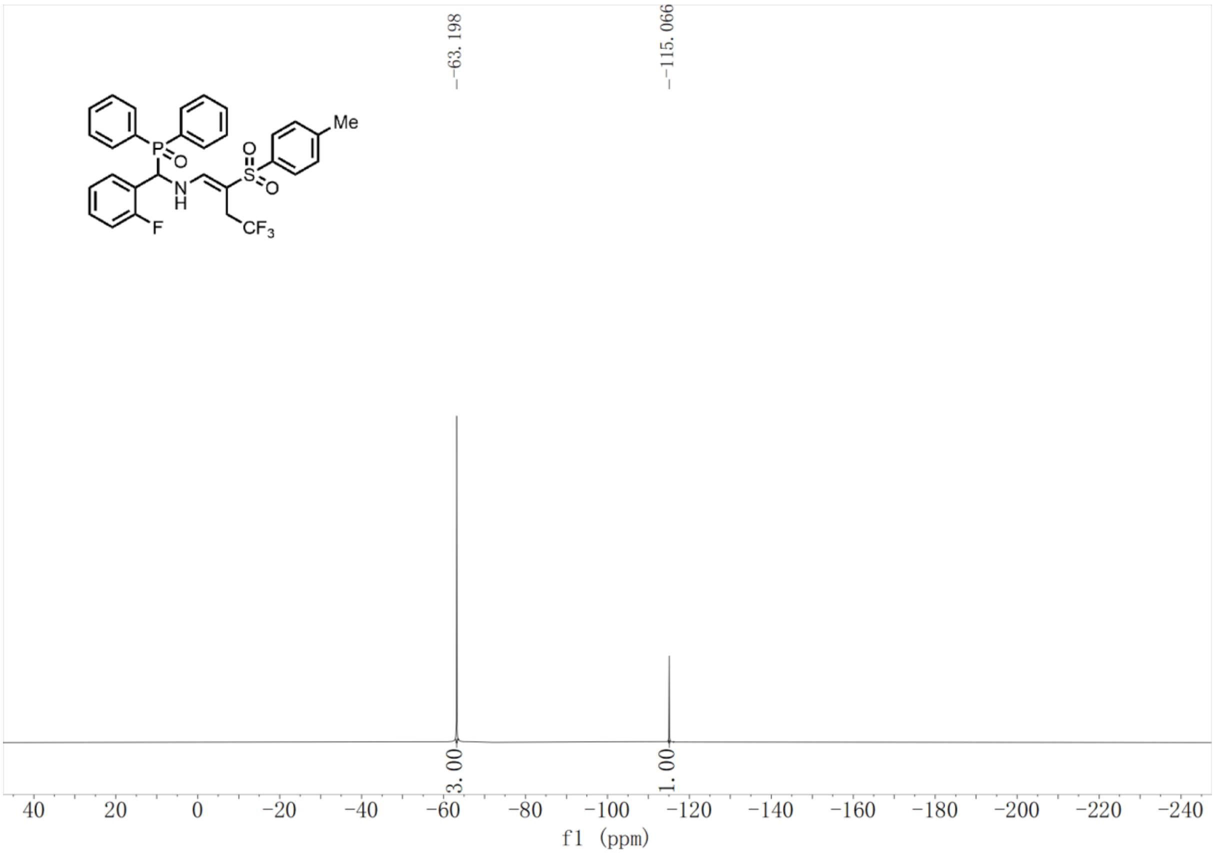

4j – ^{31}P NMR (202 MHz, DMSO)

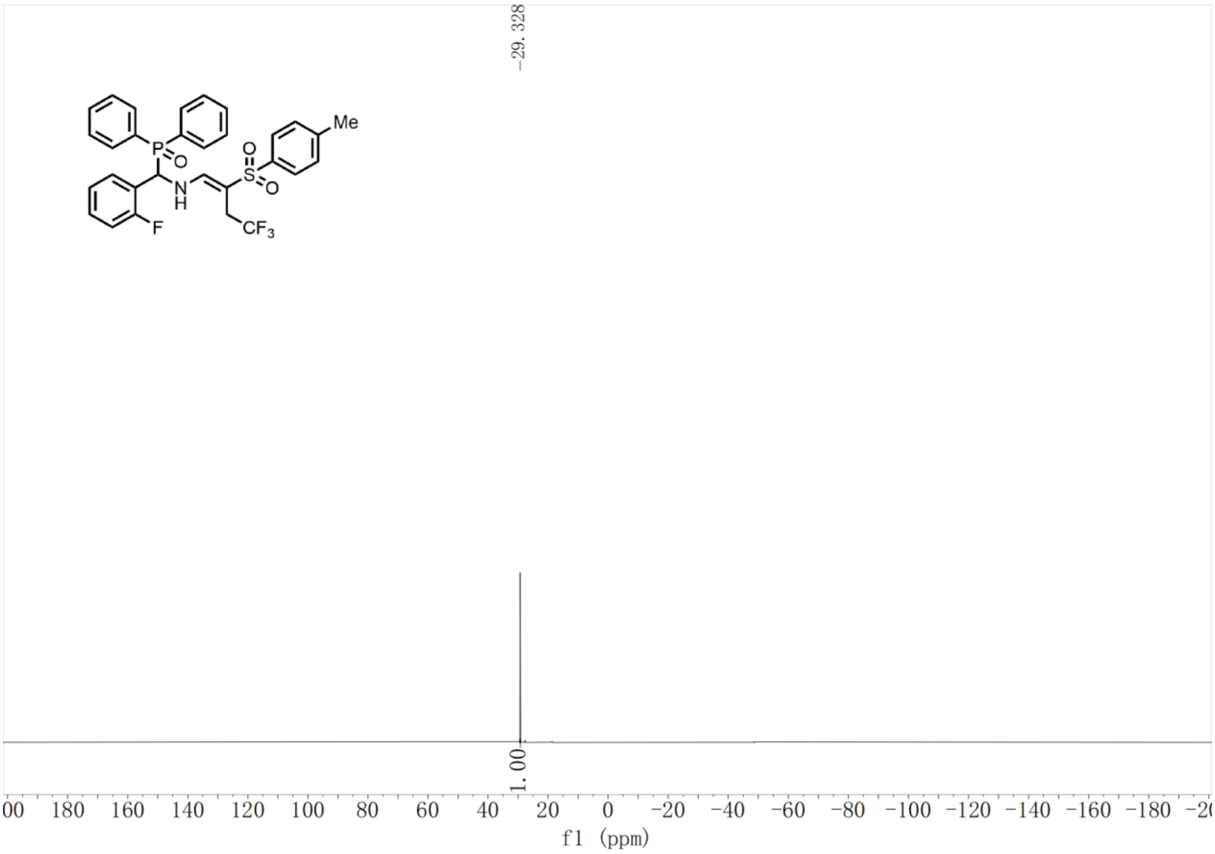

4k – ^{1}H NMR (500 MHz, DMSO)

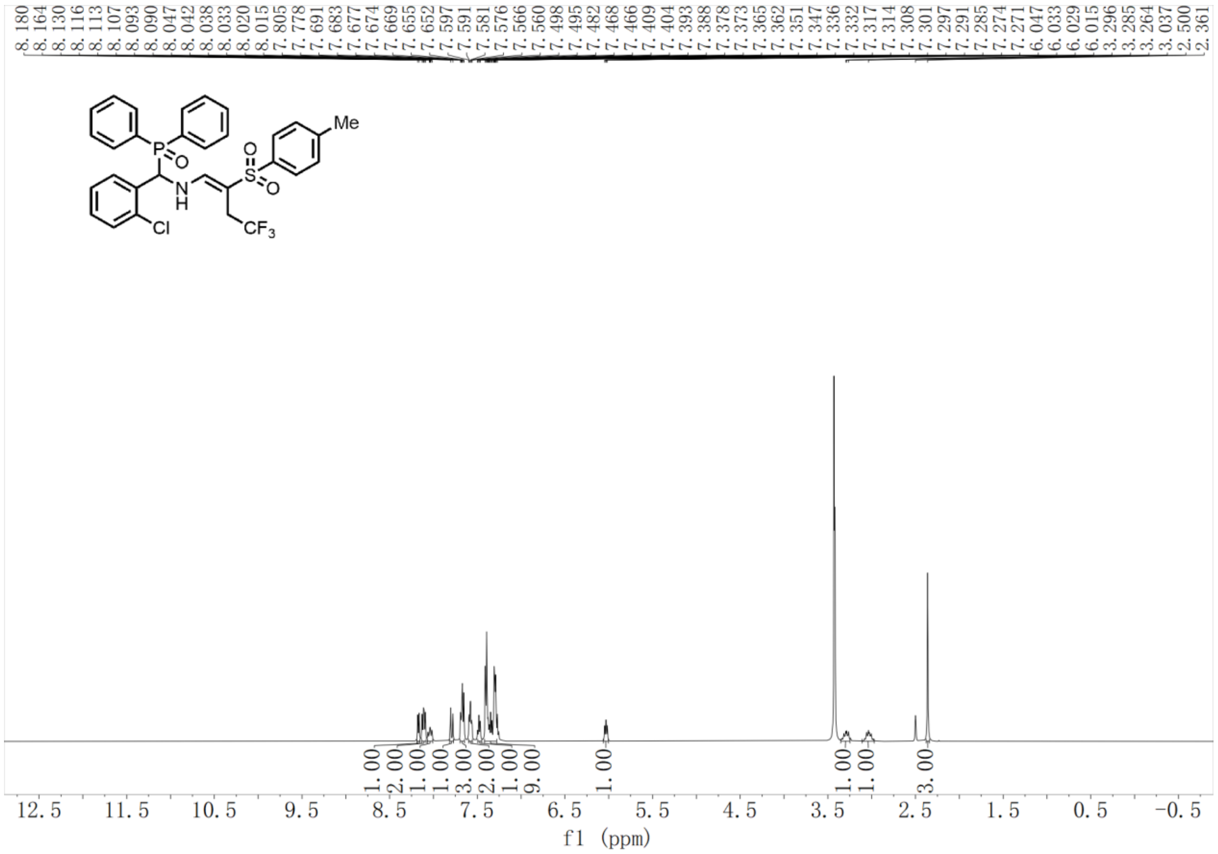

4k – ^{13}C NMR (126 MHz, DMSO)

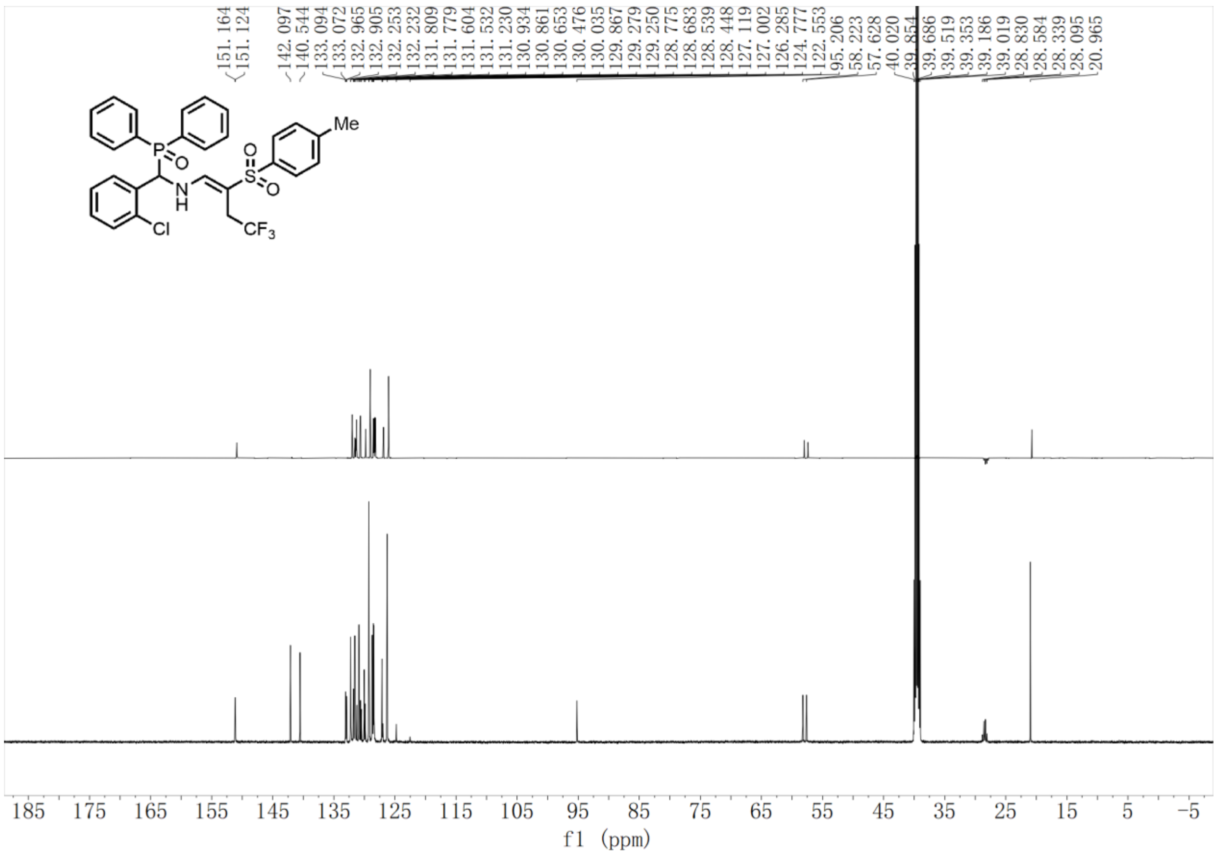

4k – ^{19}F NMR (471 MHz, DMSO)

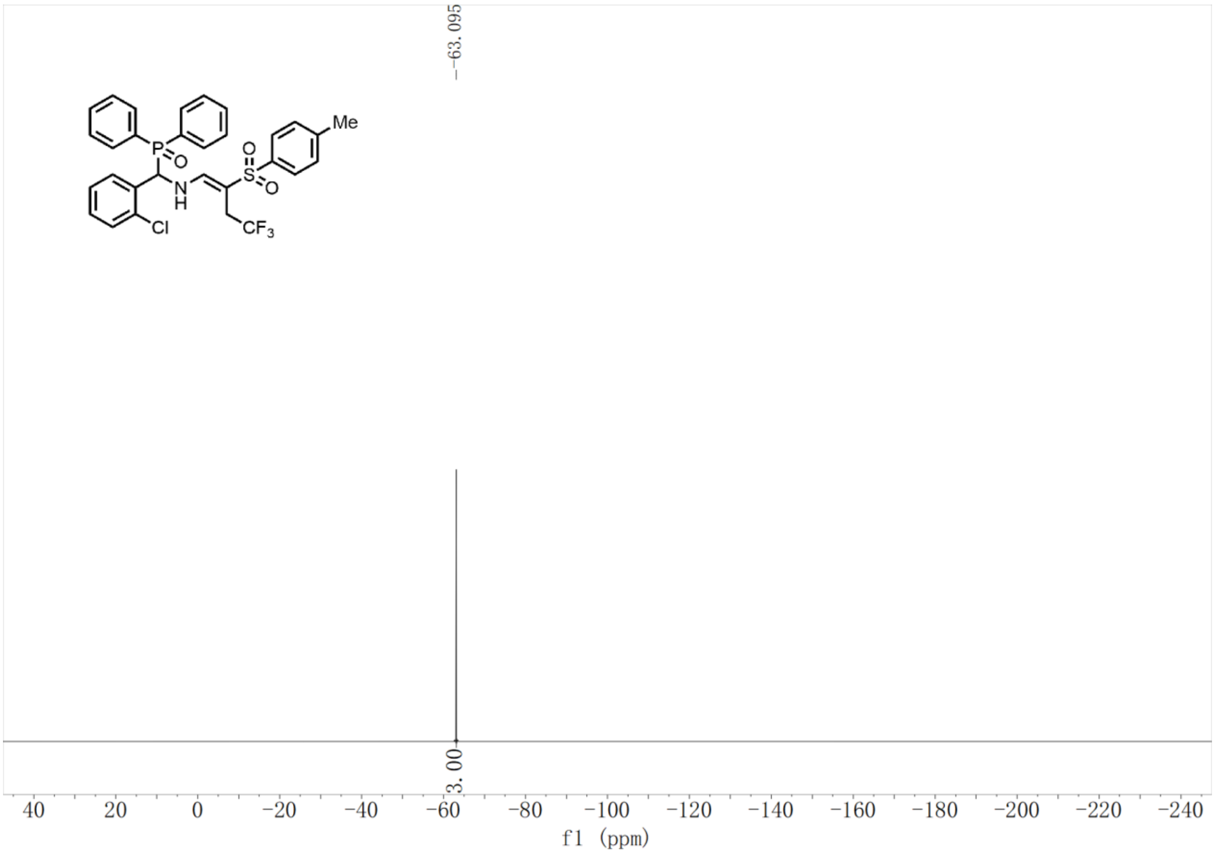

4k – ^{31}P NMR (202 MHz, DMSO)

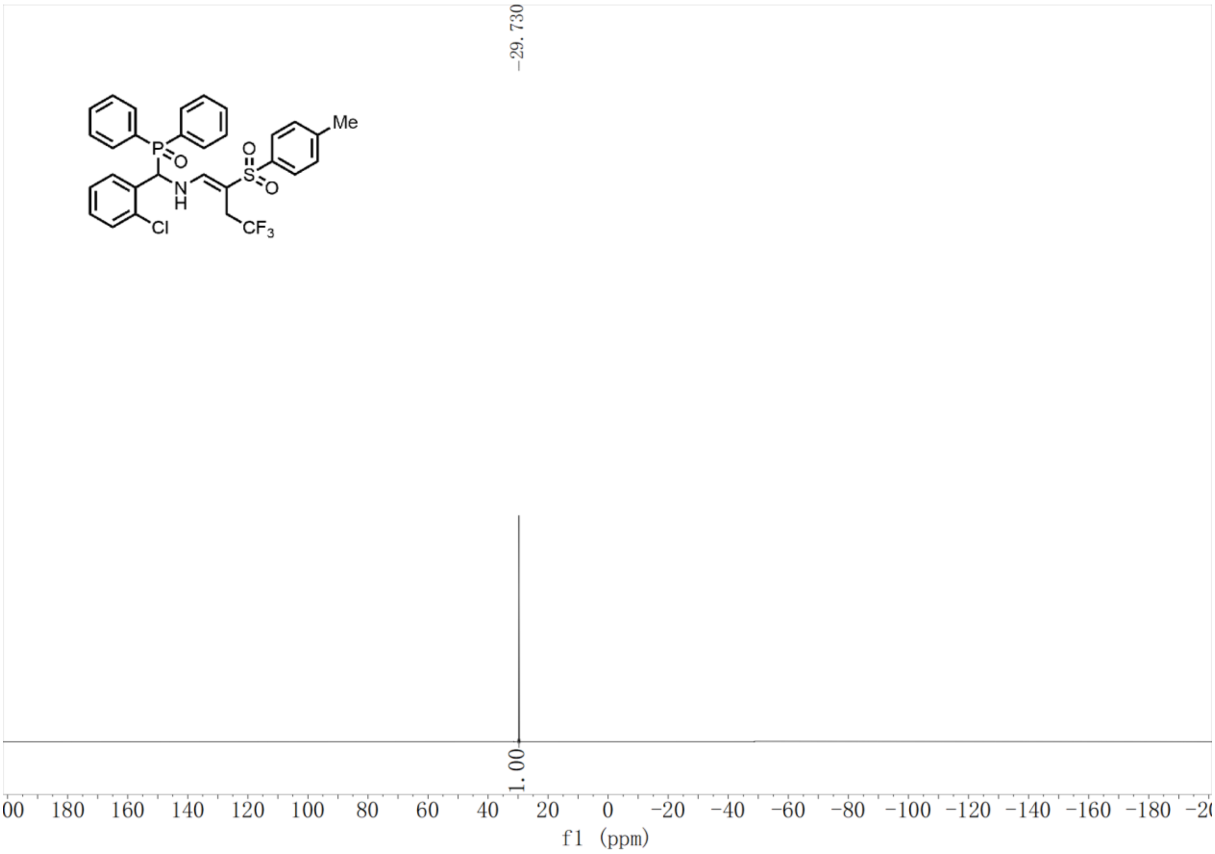

4l – ^{1}H NMR (500 MHz, DMSO)

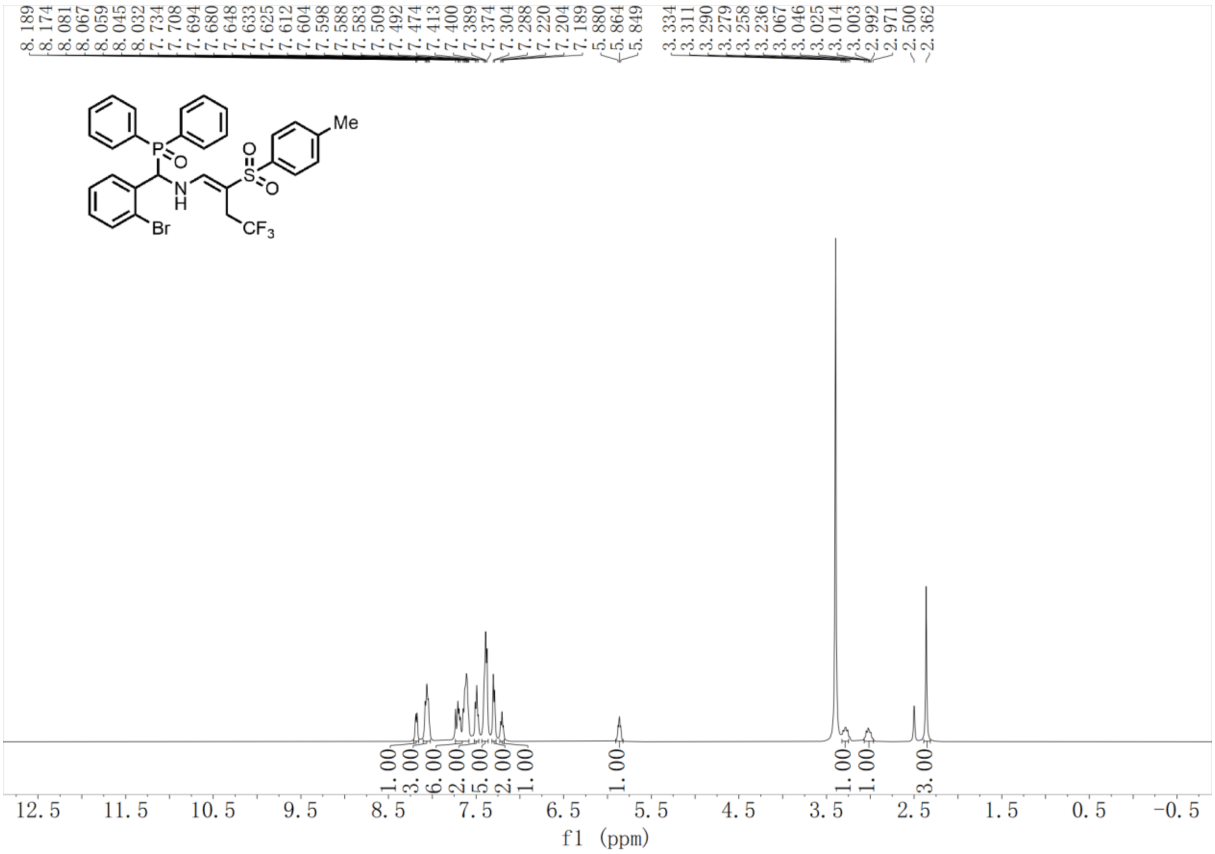

4l – ^{13}C NMR (126 MHz, DMSO)

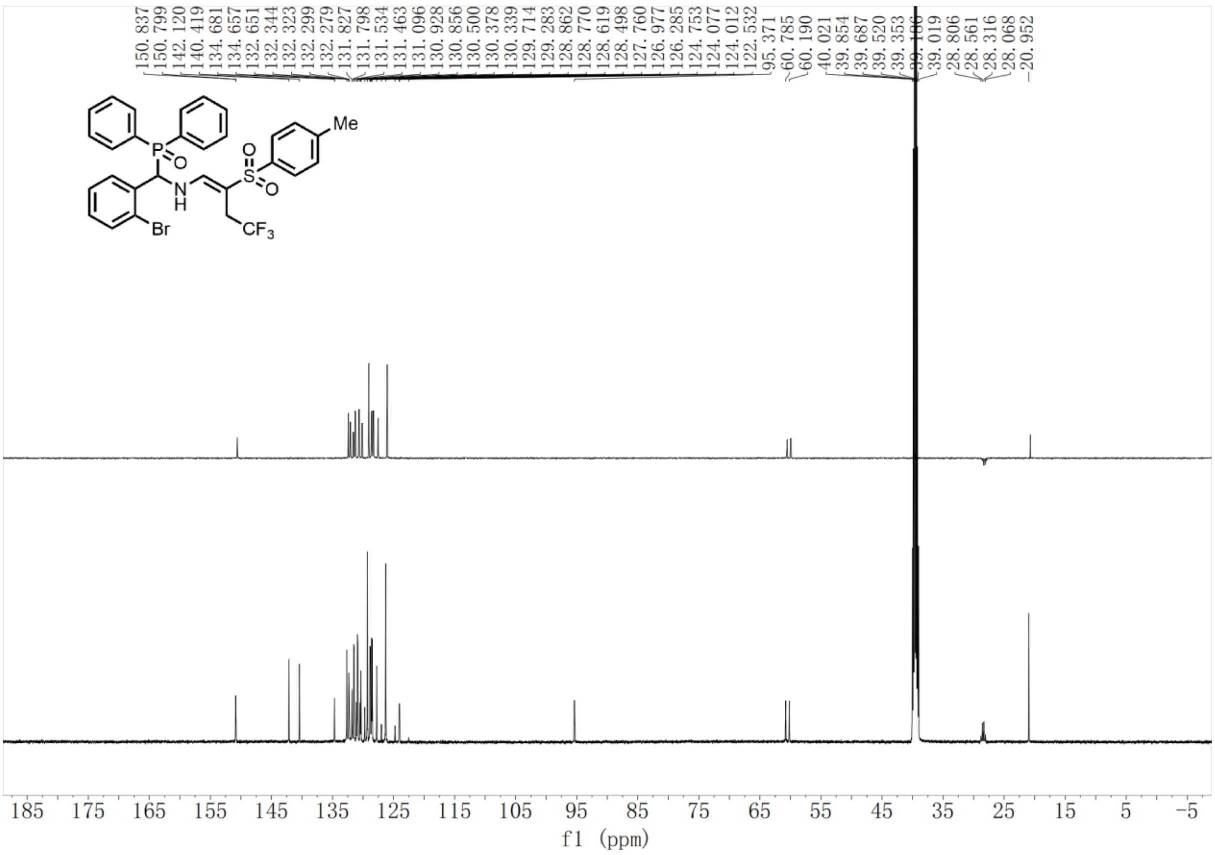

4l – ^{19}F NMR (471 MHz, DMSO)

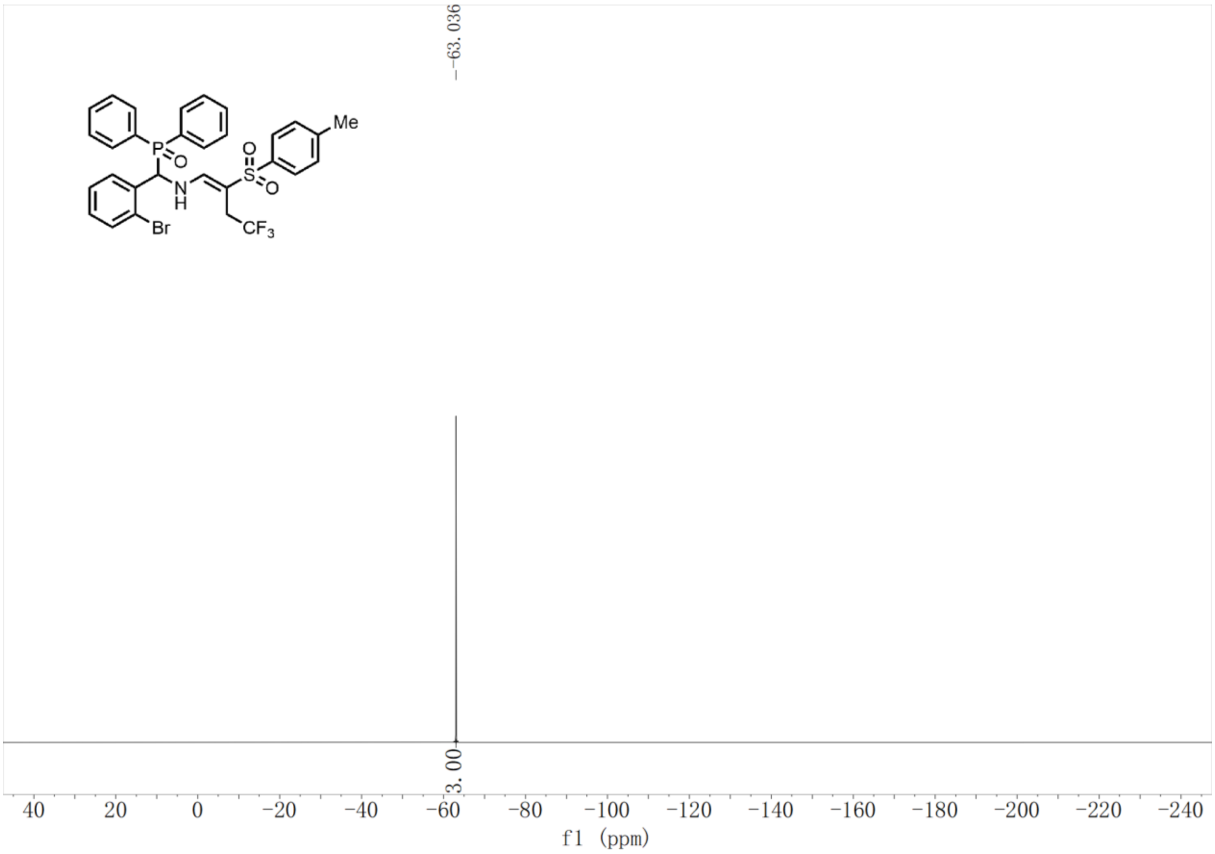

4l – ^{31}P NMR (202 MHz, DMSO)

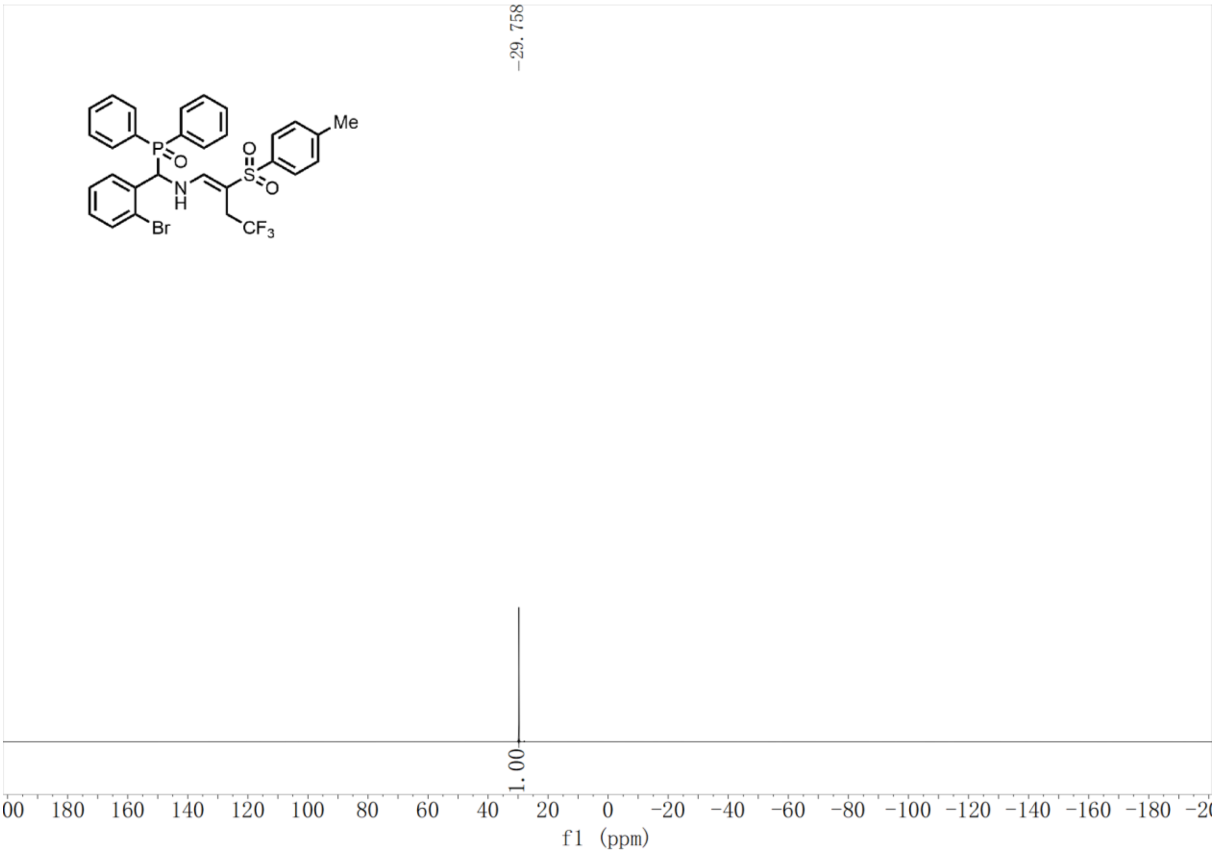

4m – ^{1}H NMR (500 MHz, DMSO)

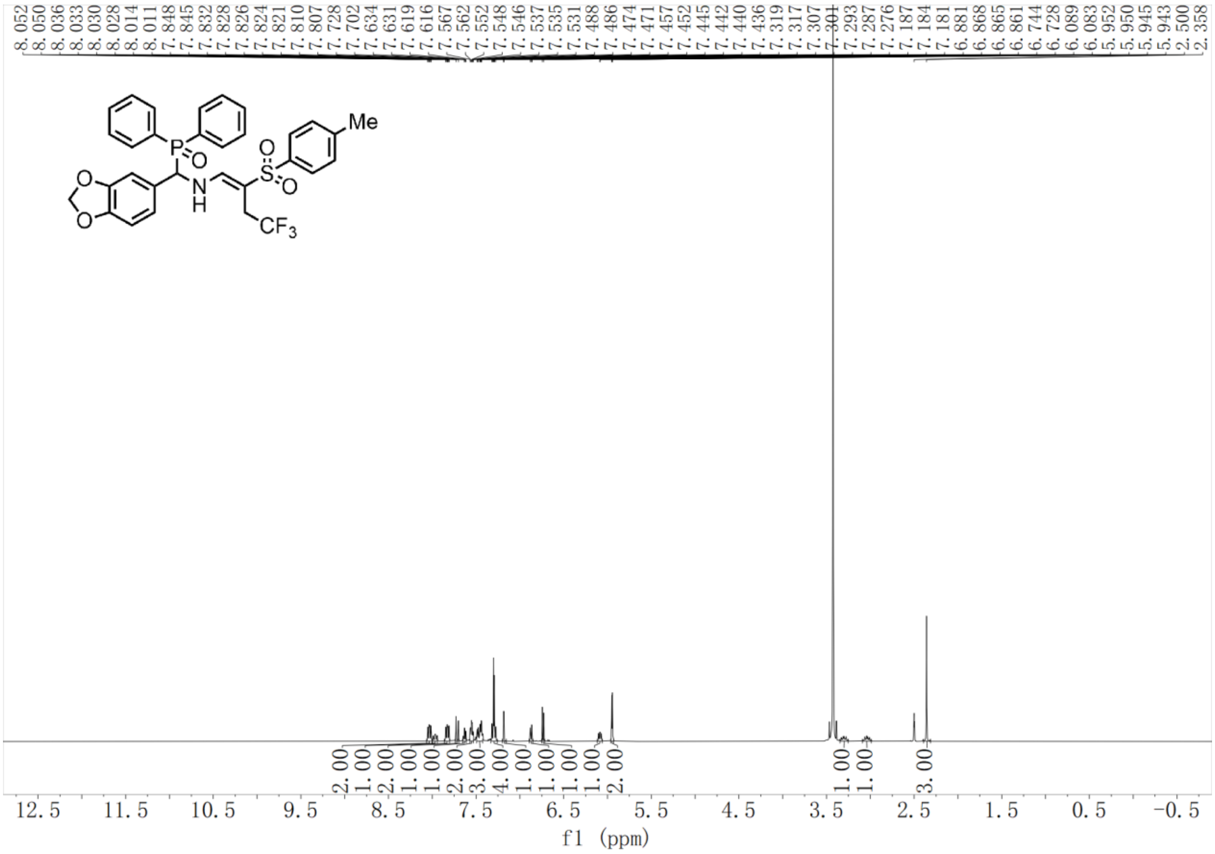

4m – ^{13}C NMR (126 MHz, DMSO)

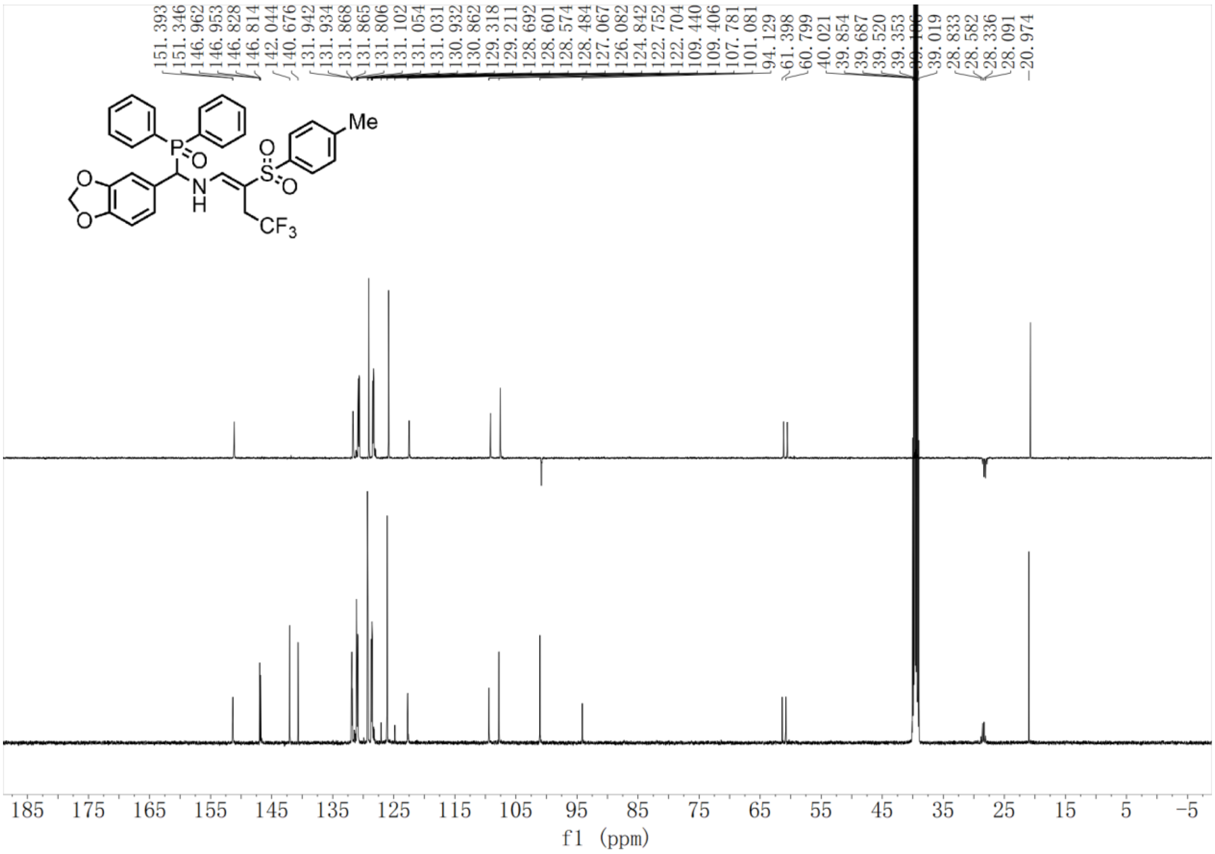

4m – ^{19}F NMR (282 MHz, DMSO)

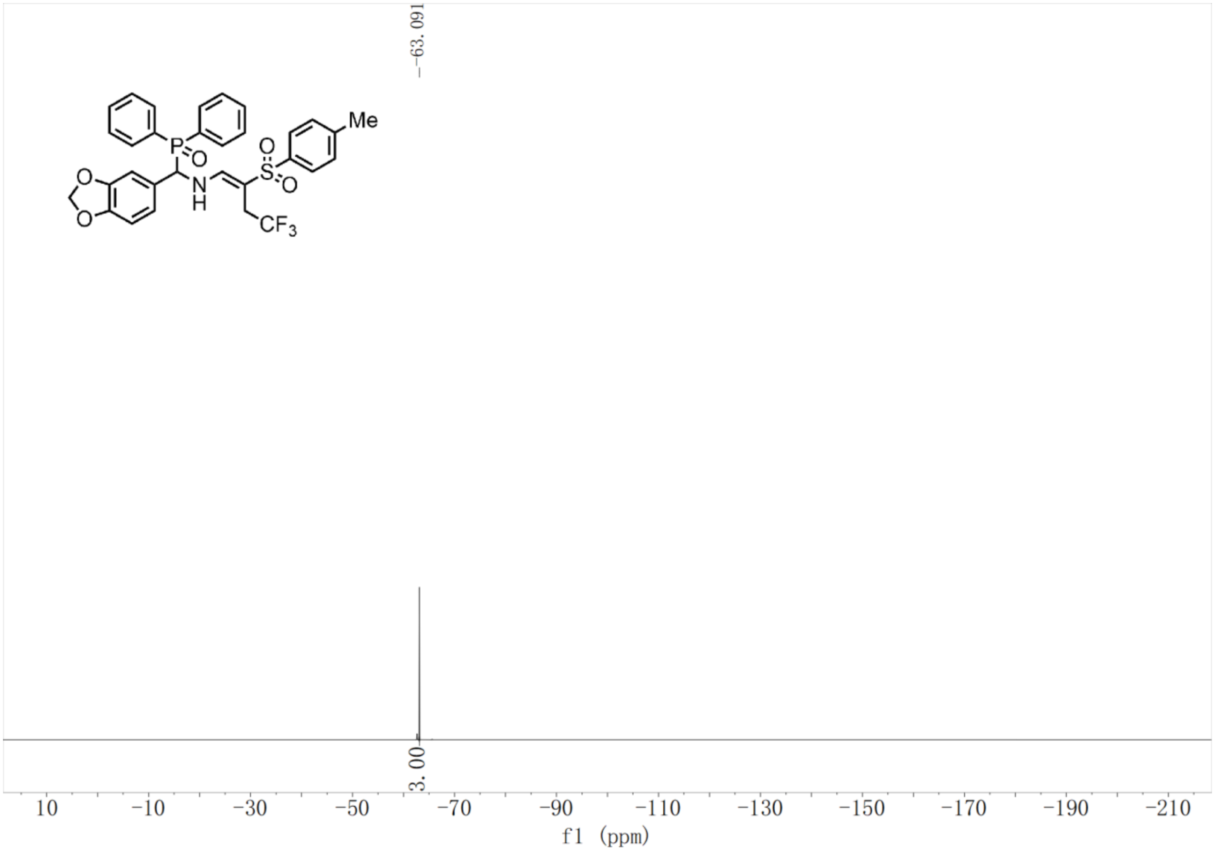

4m – ^{31}P NMR (121 MHz, DMSO)

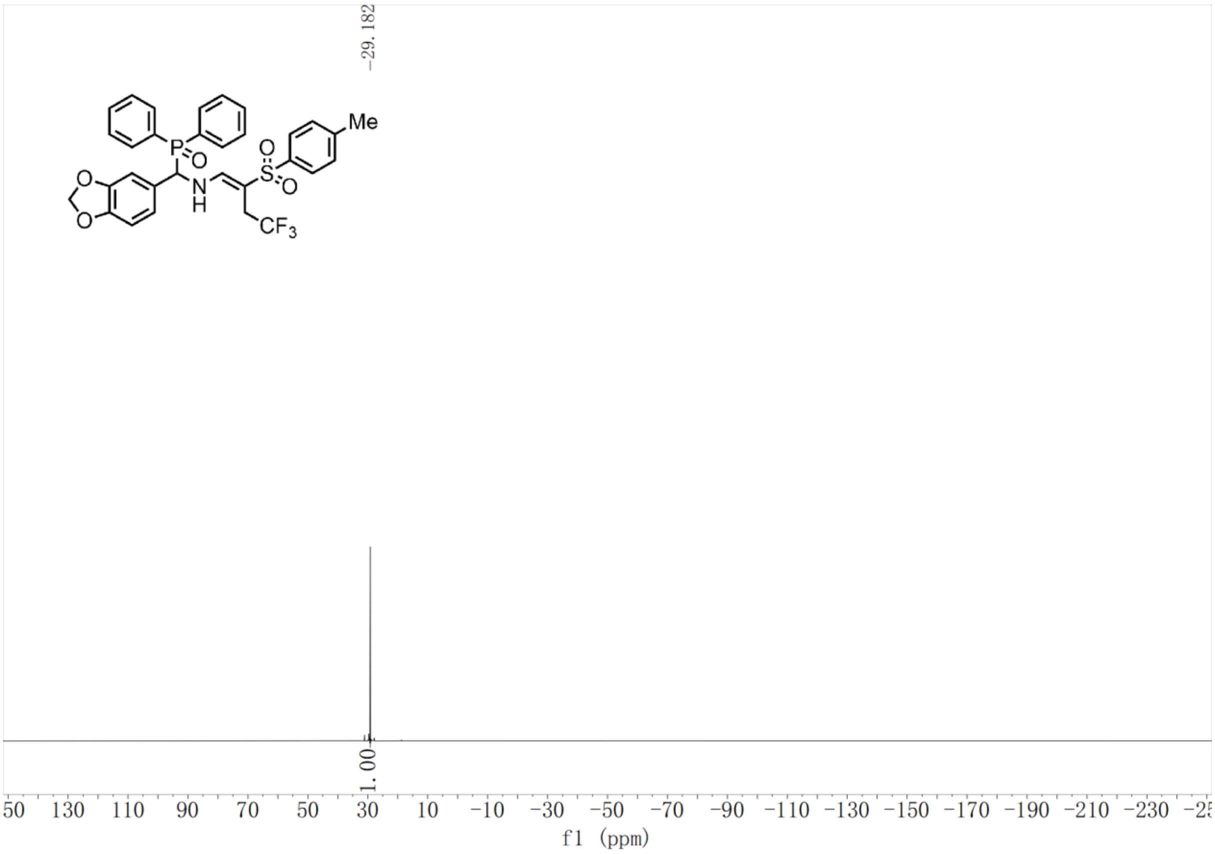

4n – ^{1}H NMR (500 MHz, DMSO)

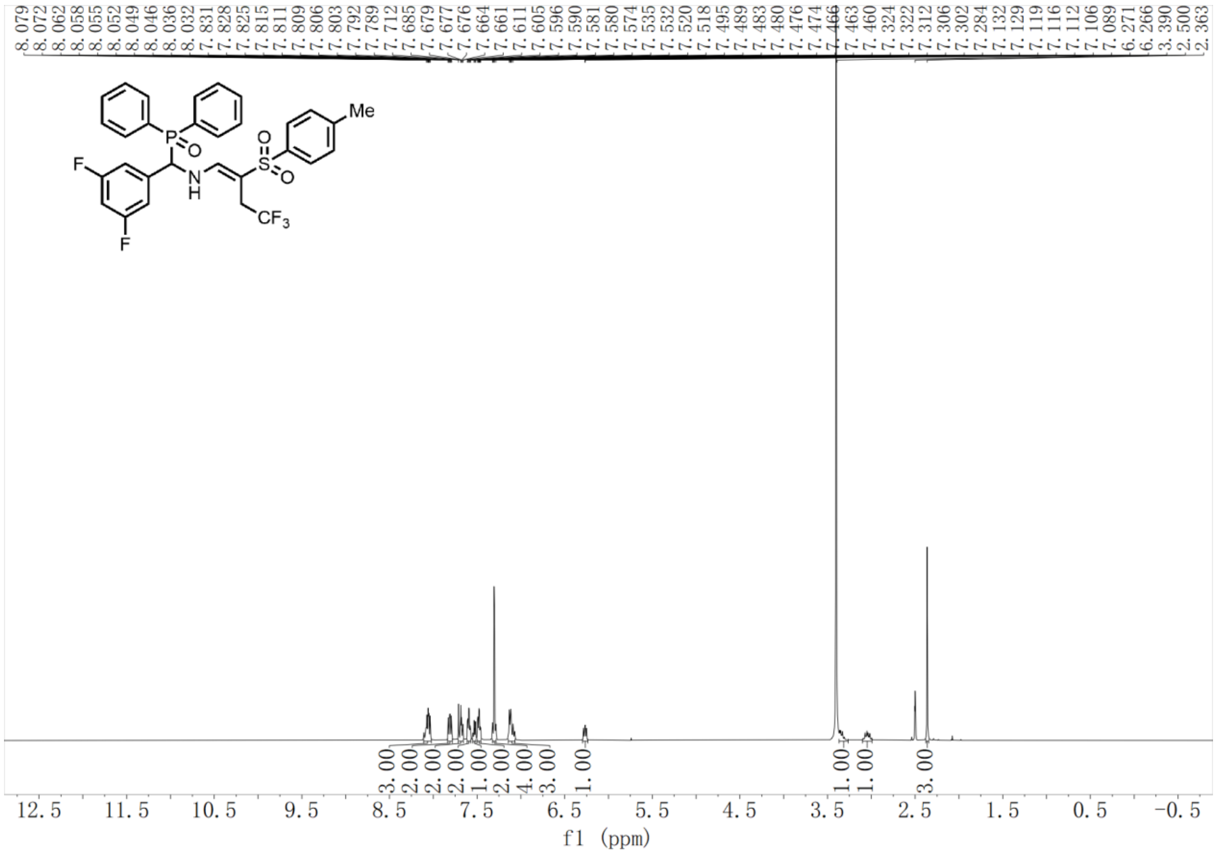

4n – ^{13}C NMR (126 MHz, DMSO)

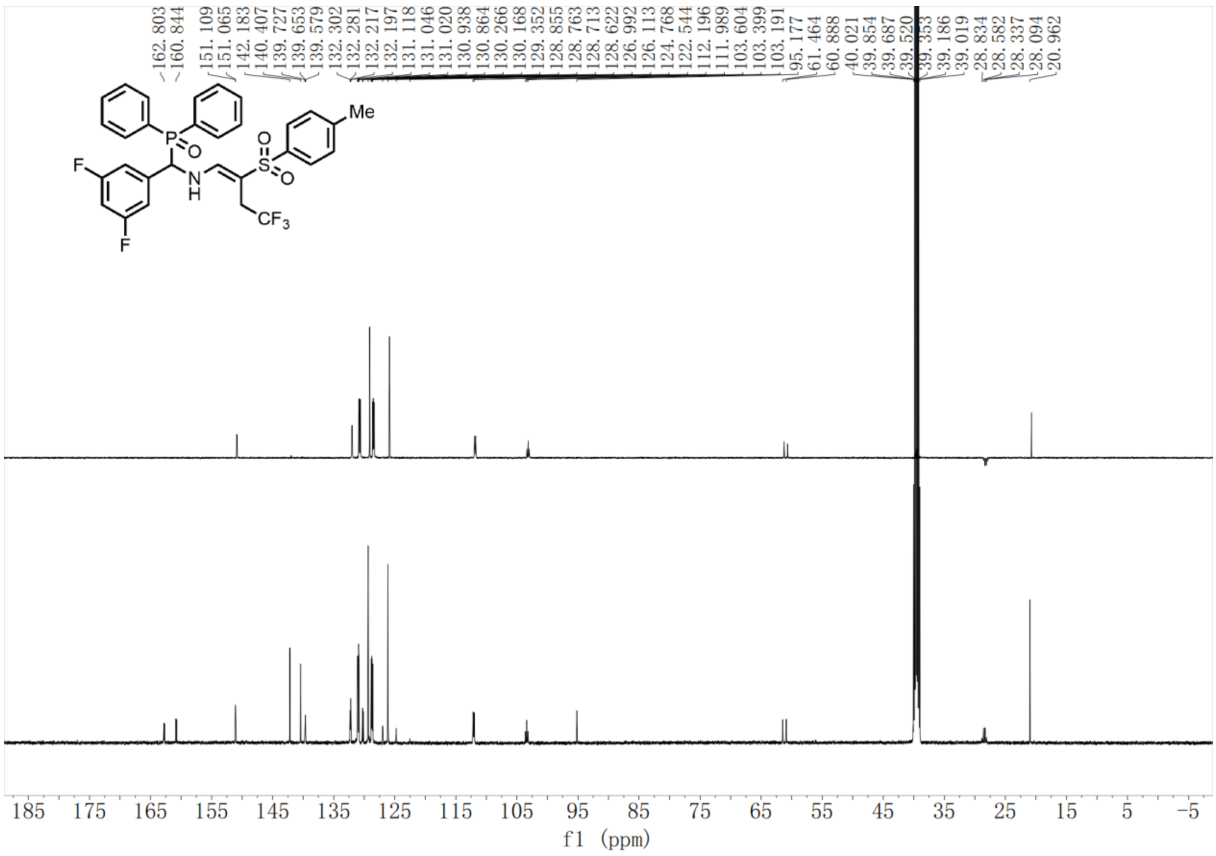

4n – ^{19}F NMR (471 MHz, DMSO)

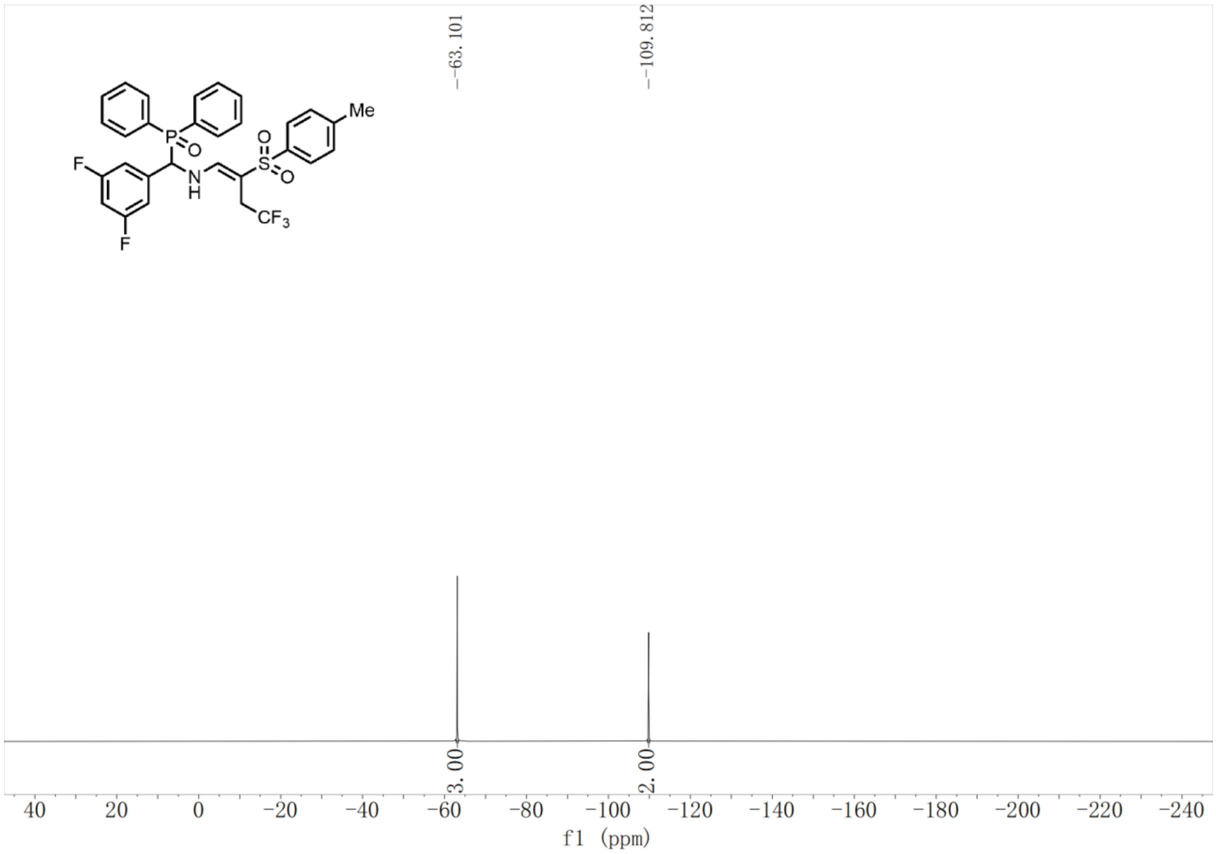

4n – ^{31}P NMR (202 MHz, DMSO)

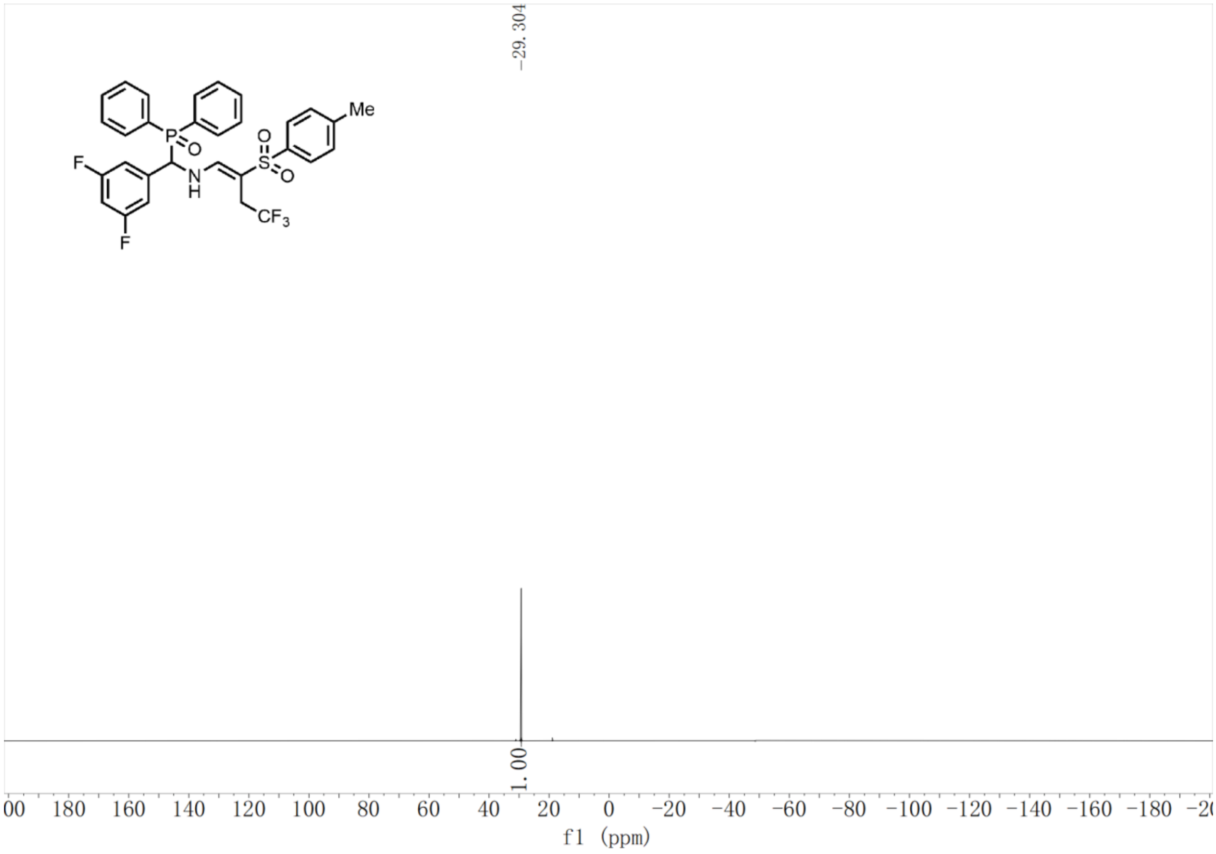

4o – ^{1}H NMR (500 MHz, DMSO)

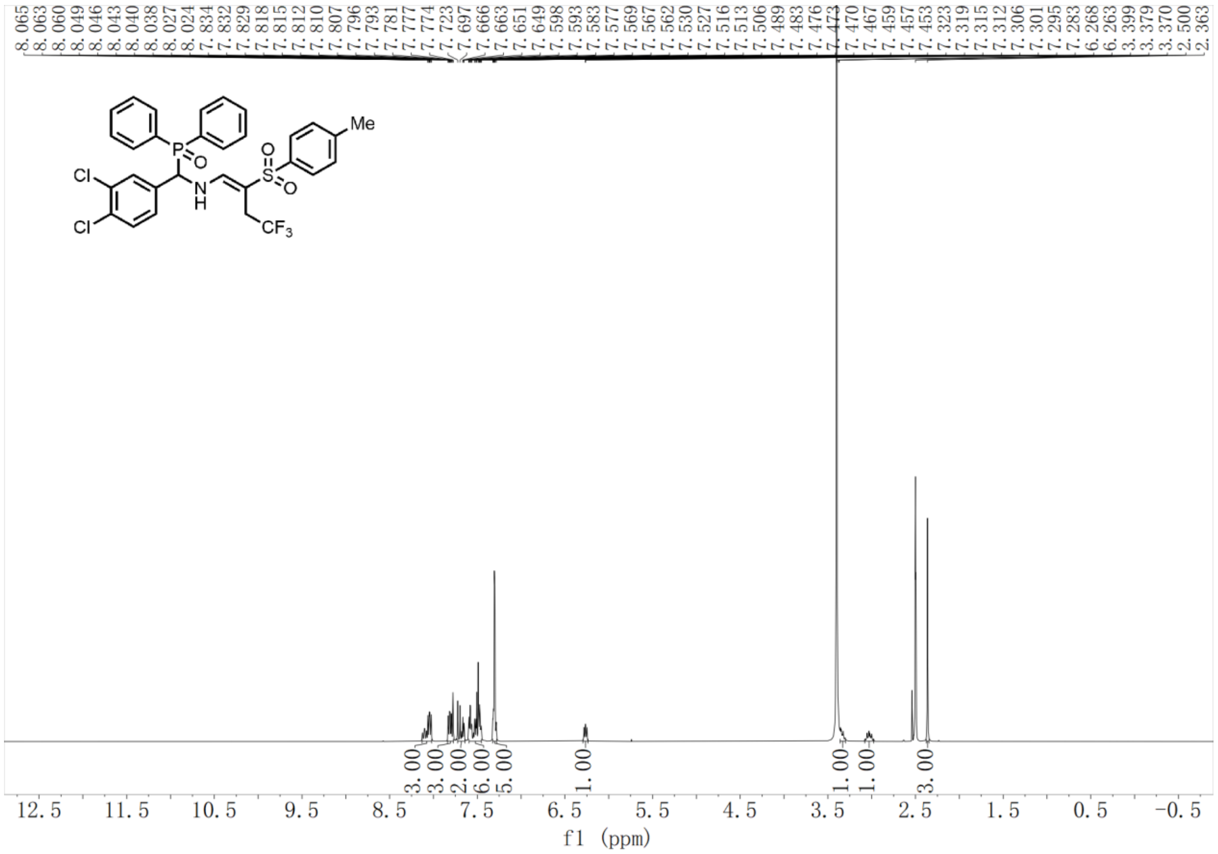

4o – ^{13}C NMR (126 MHz, DMSO)

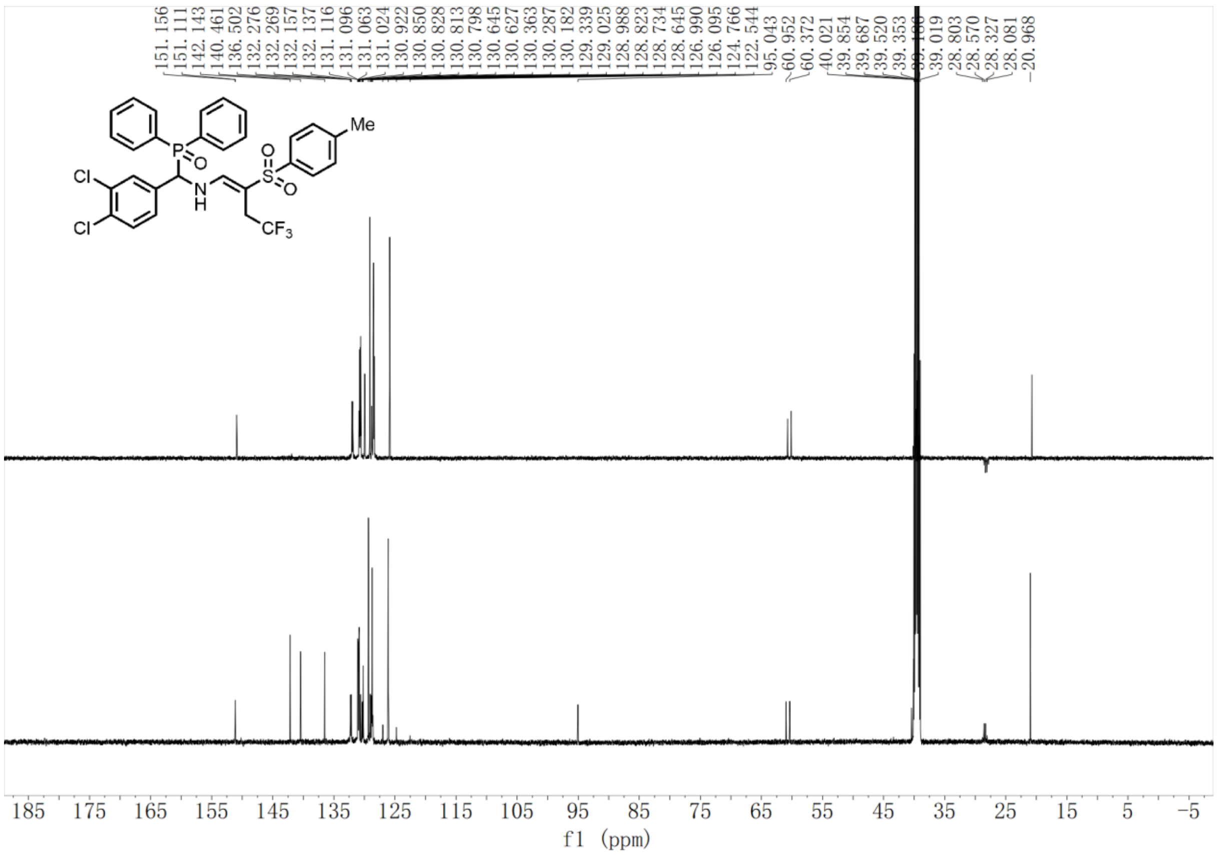

4o – ^{19}F NMR (471 MHz, DMSO)

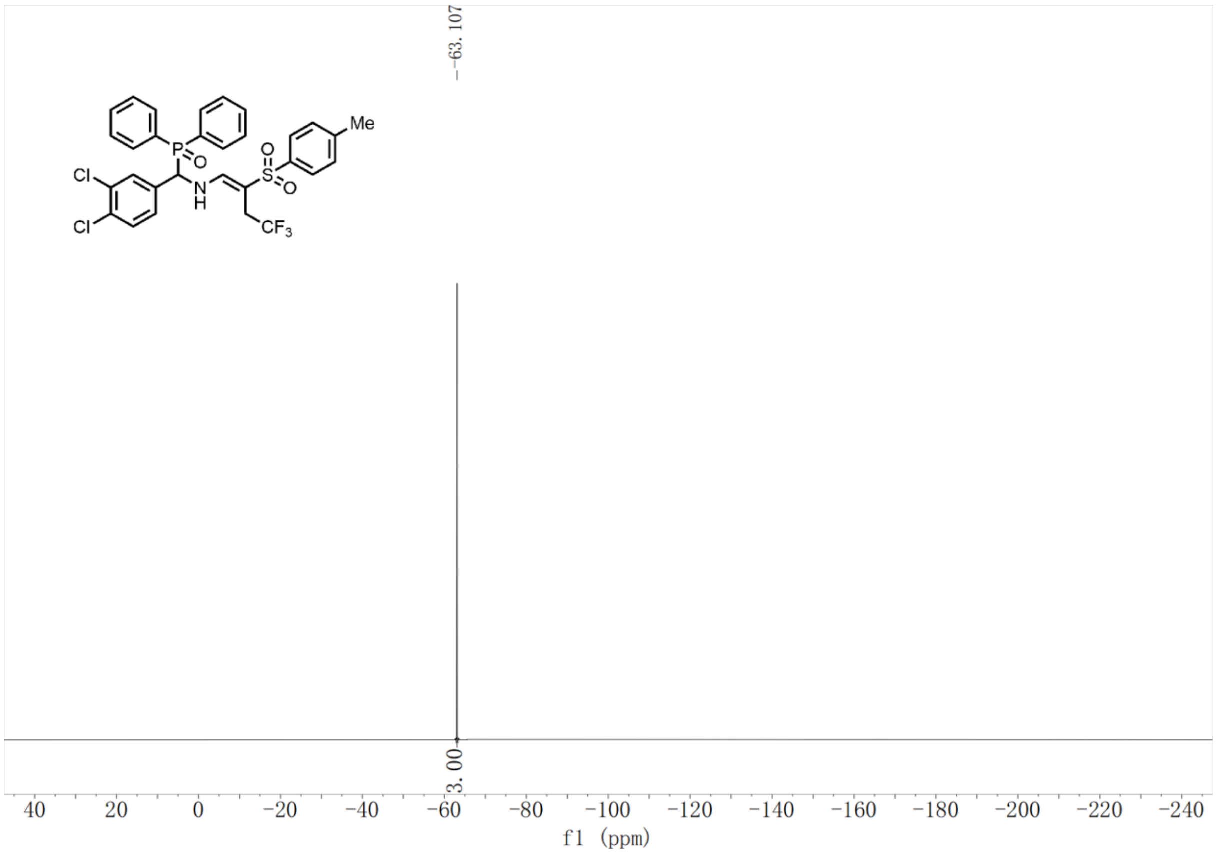

4o – ^{31}P NMR (202 MHz, DMSO)

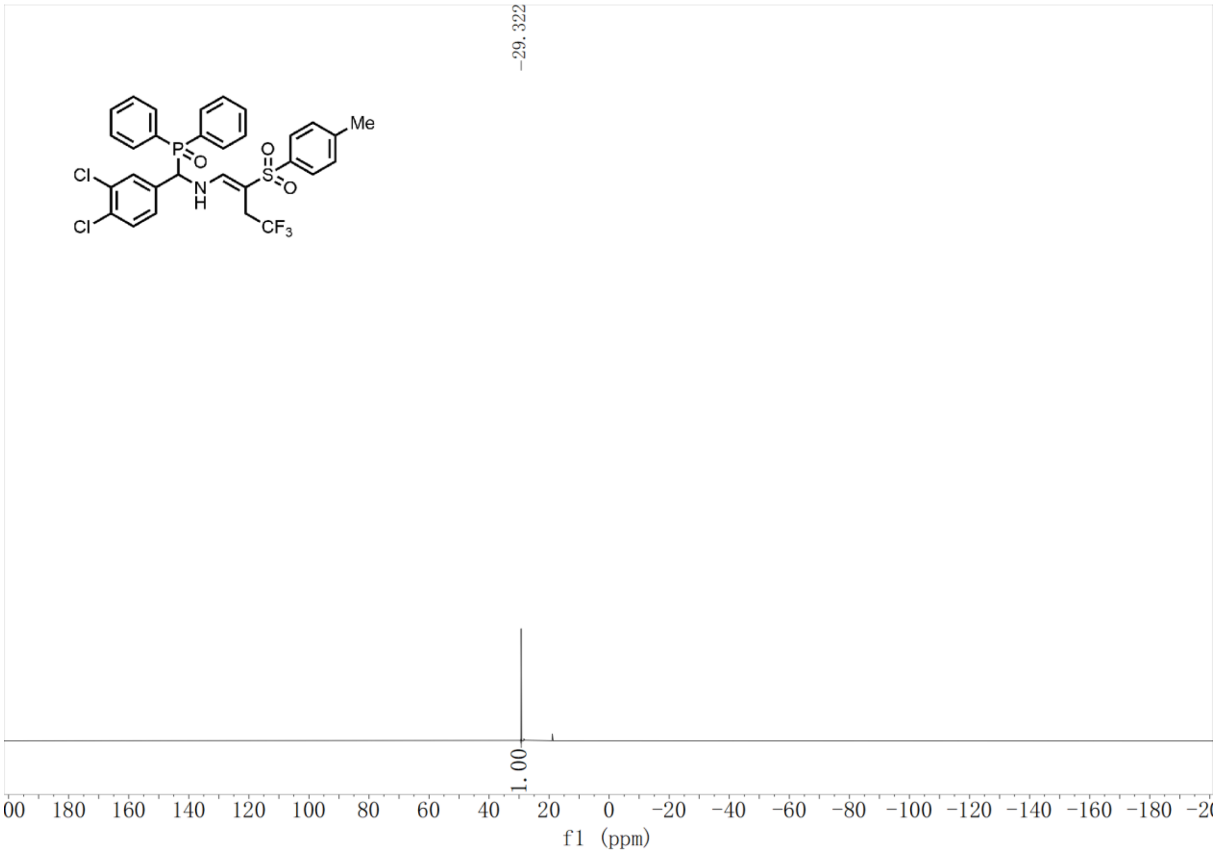

4p – ^{1}H NMR (500 MHz, DMSO)

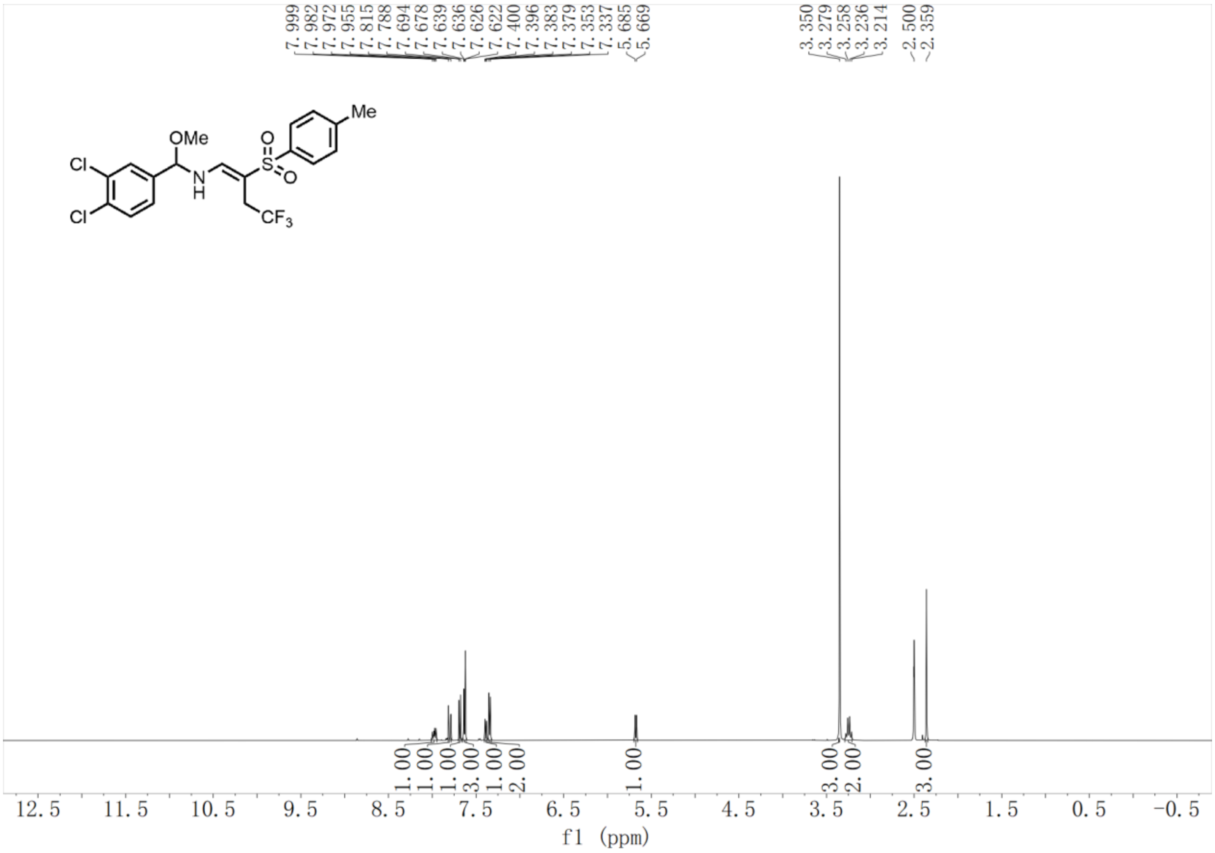

4p – ^{13}C NMR (126 MHz, DMSO)

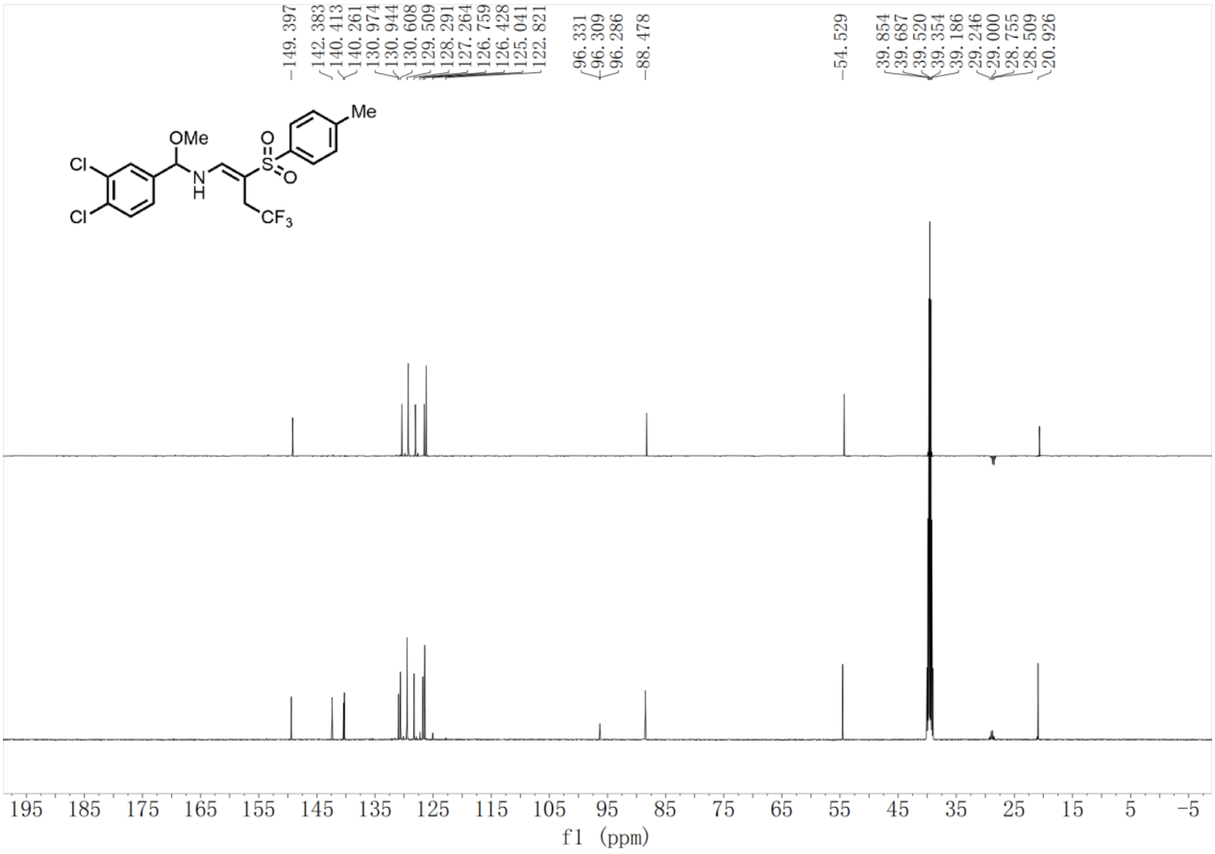

4p – ^{19}F NMR (471 MHz, DMSO)

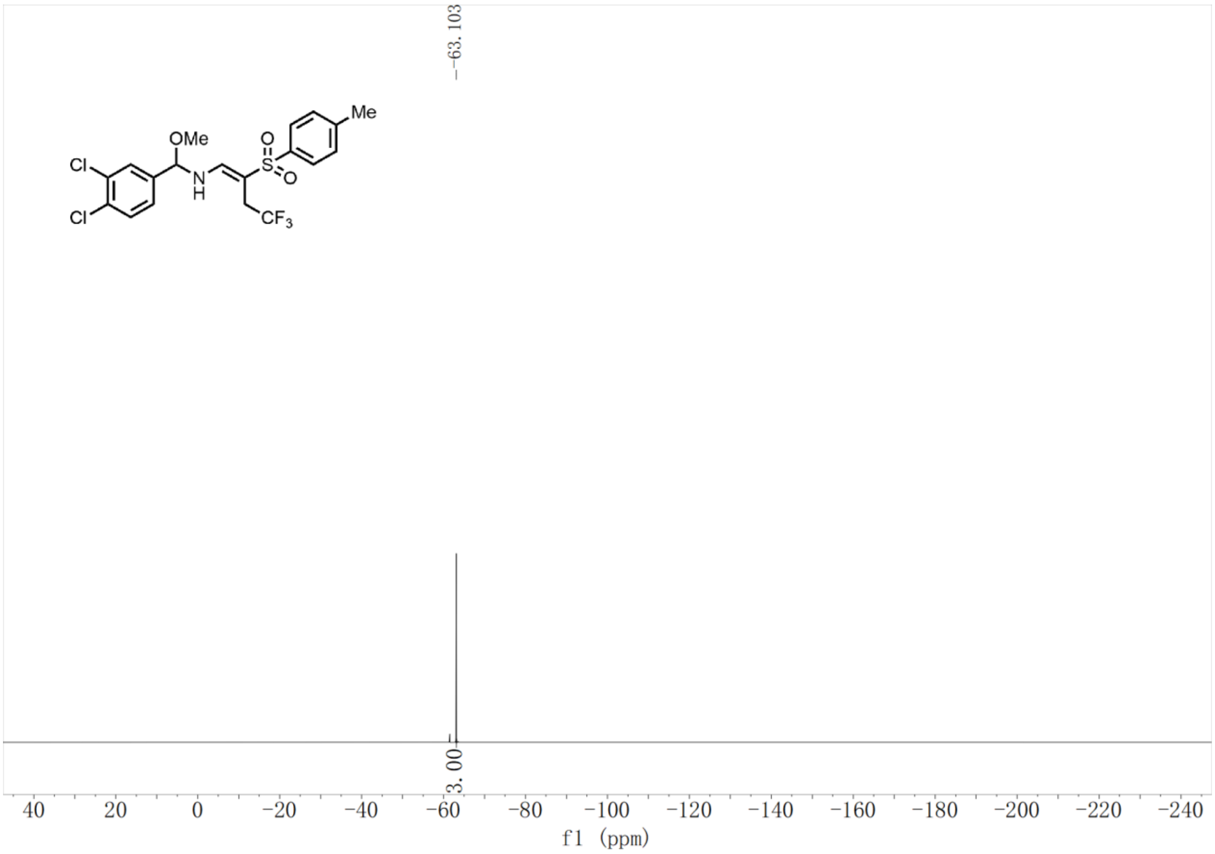

4q – ^{1}H NMR (500 MHz, DMSO)

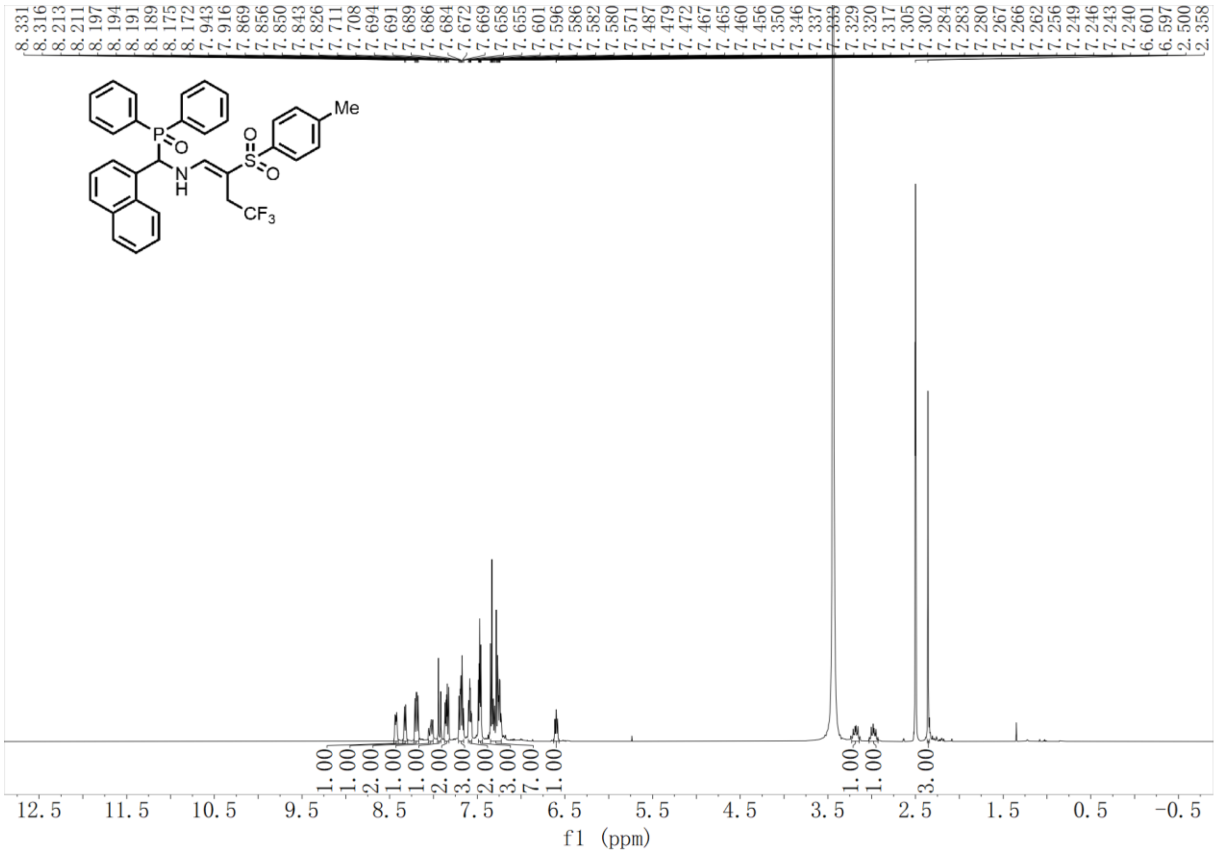

4q – ^{13}C NMR (126 MHz, DMSO)

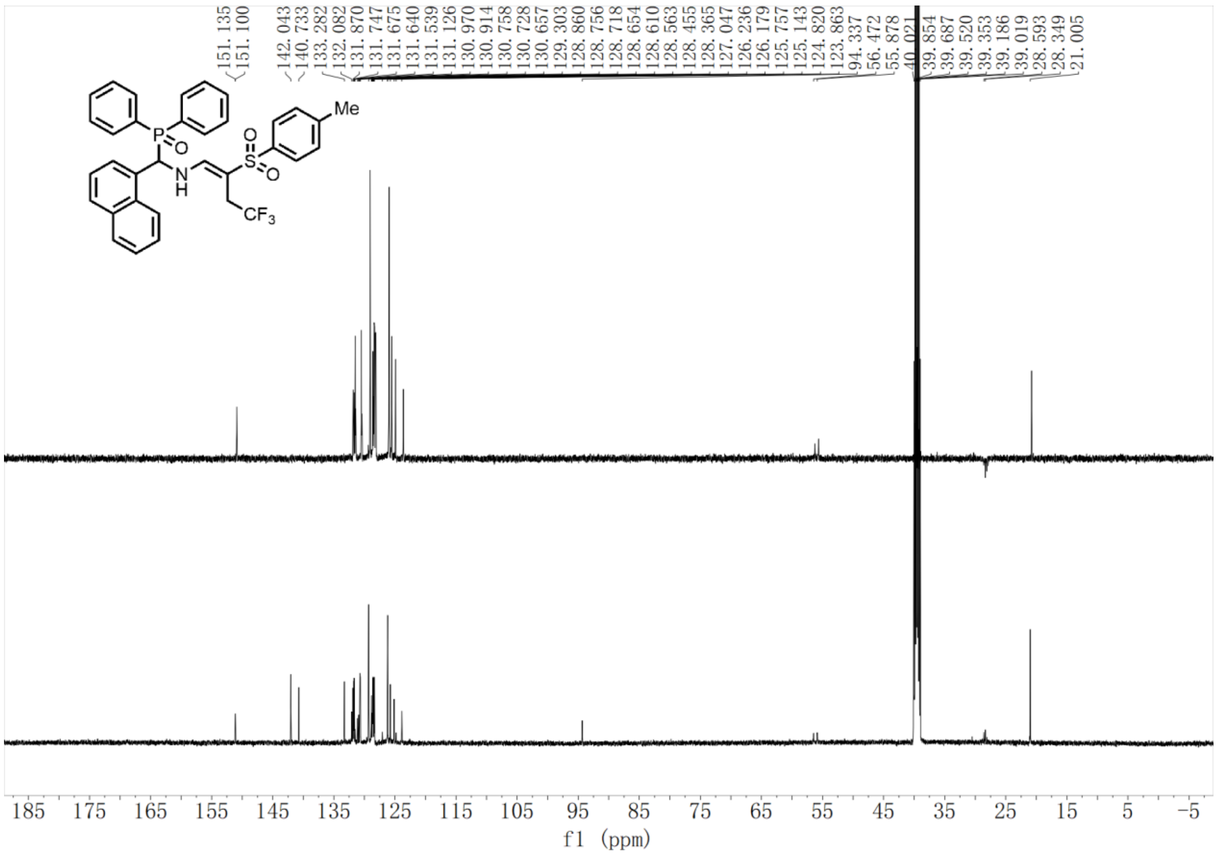

4q – ^{19}F NMR (471 MHz, DMSO)

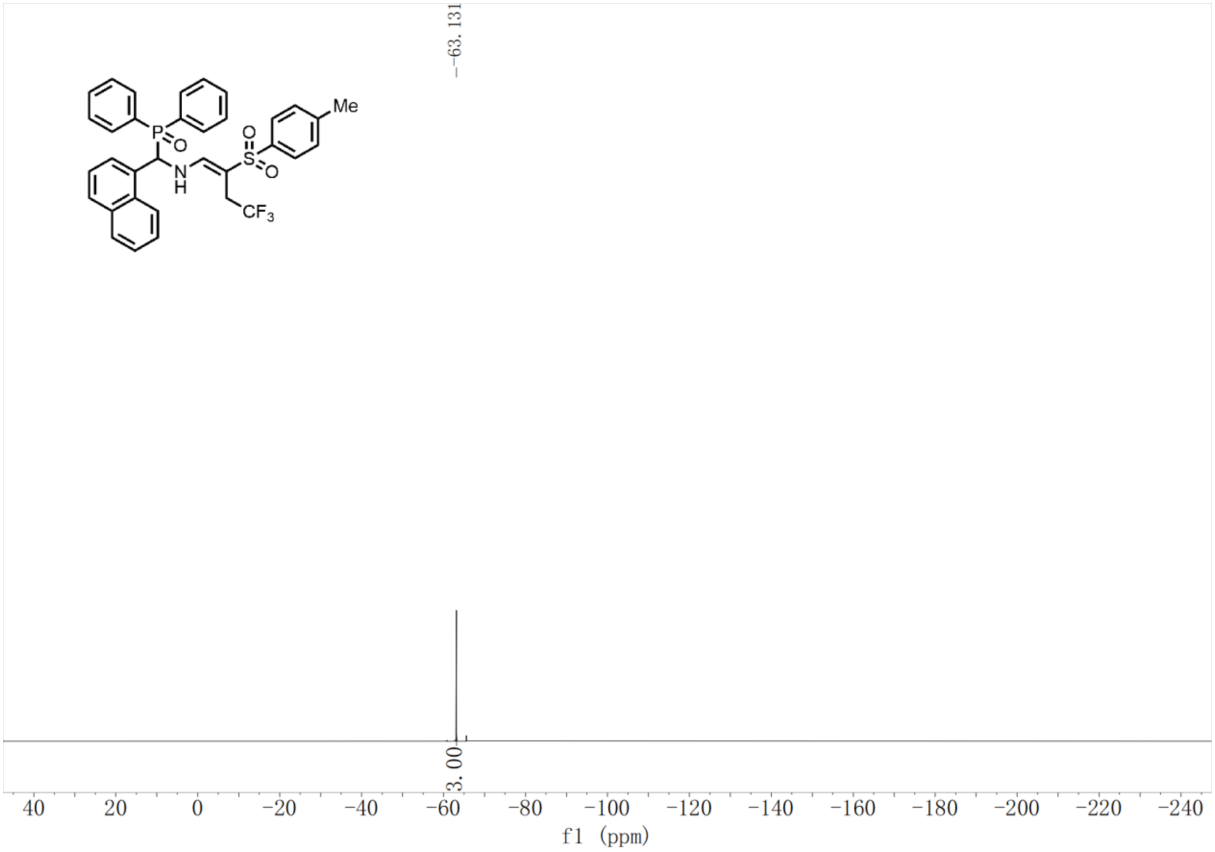

4q – ^{31}P NMR (202 MHz, DMSO)

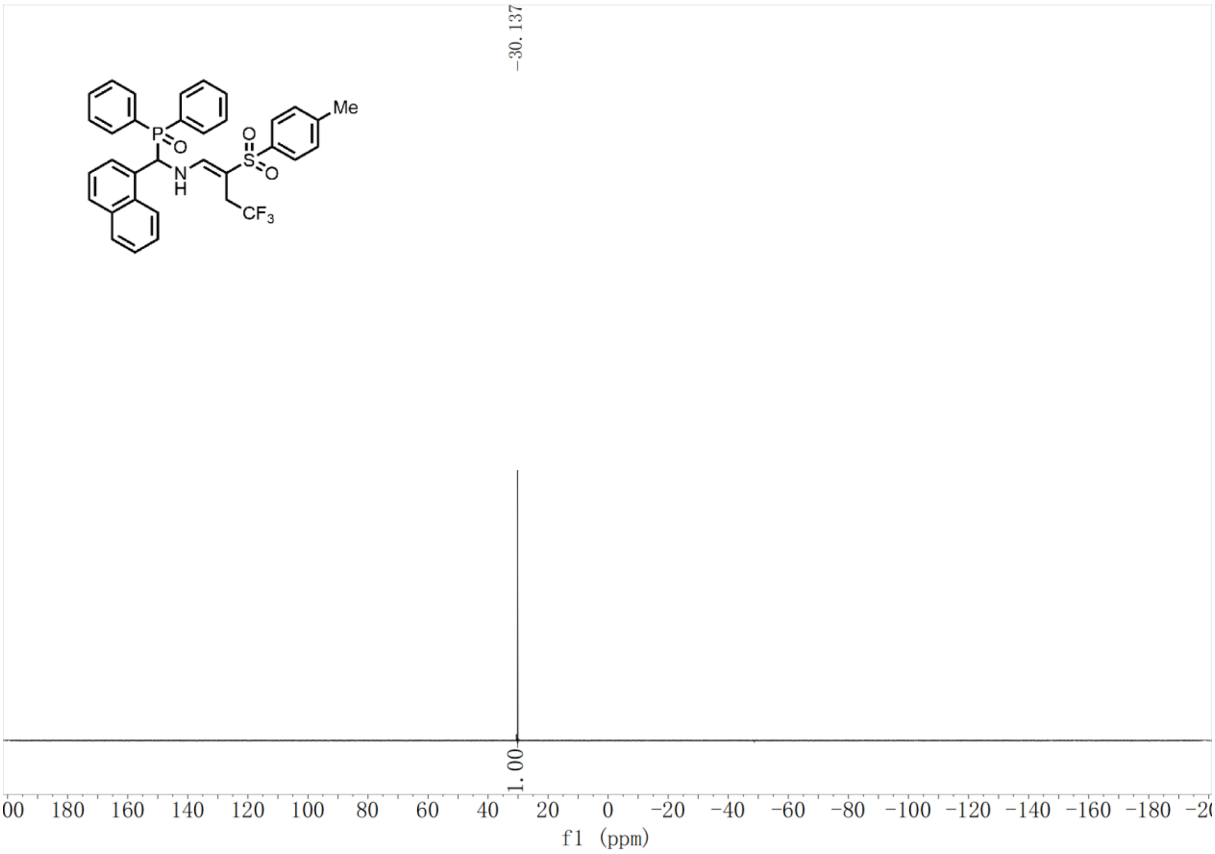

4r – ^{1}H NMR (500 MHz, DMSO)

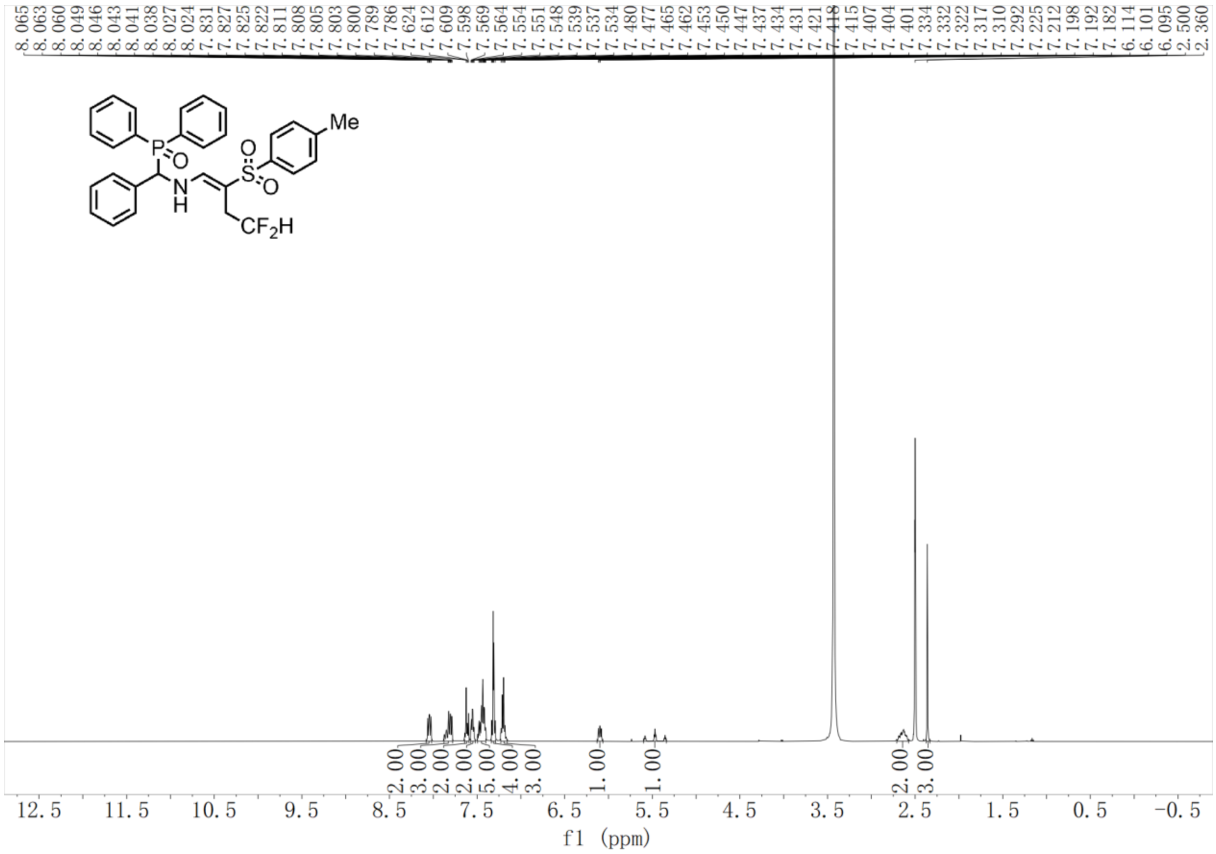

4r – ^{13}C NMR (126 MHz, DMSO)

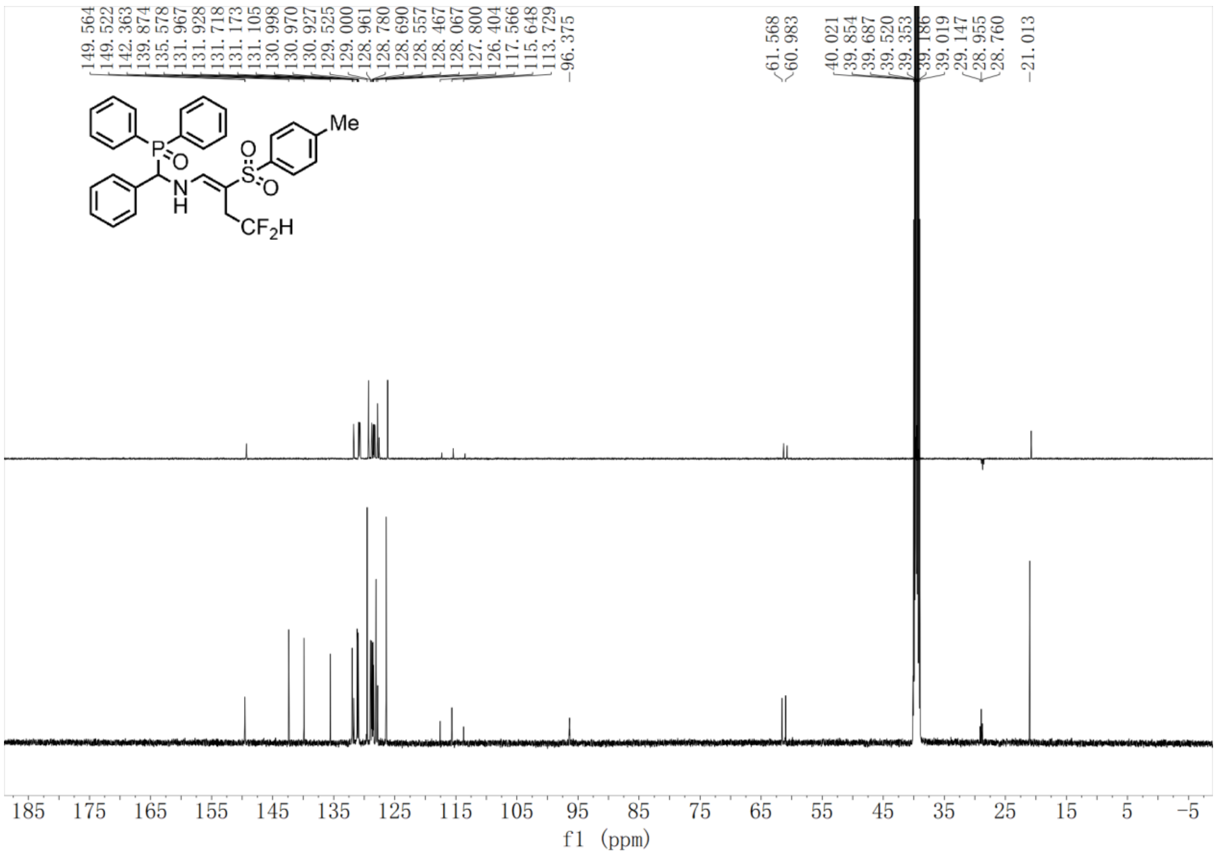

4r – ^{19}F NMR (471 MHz, DMSO)

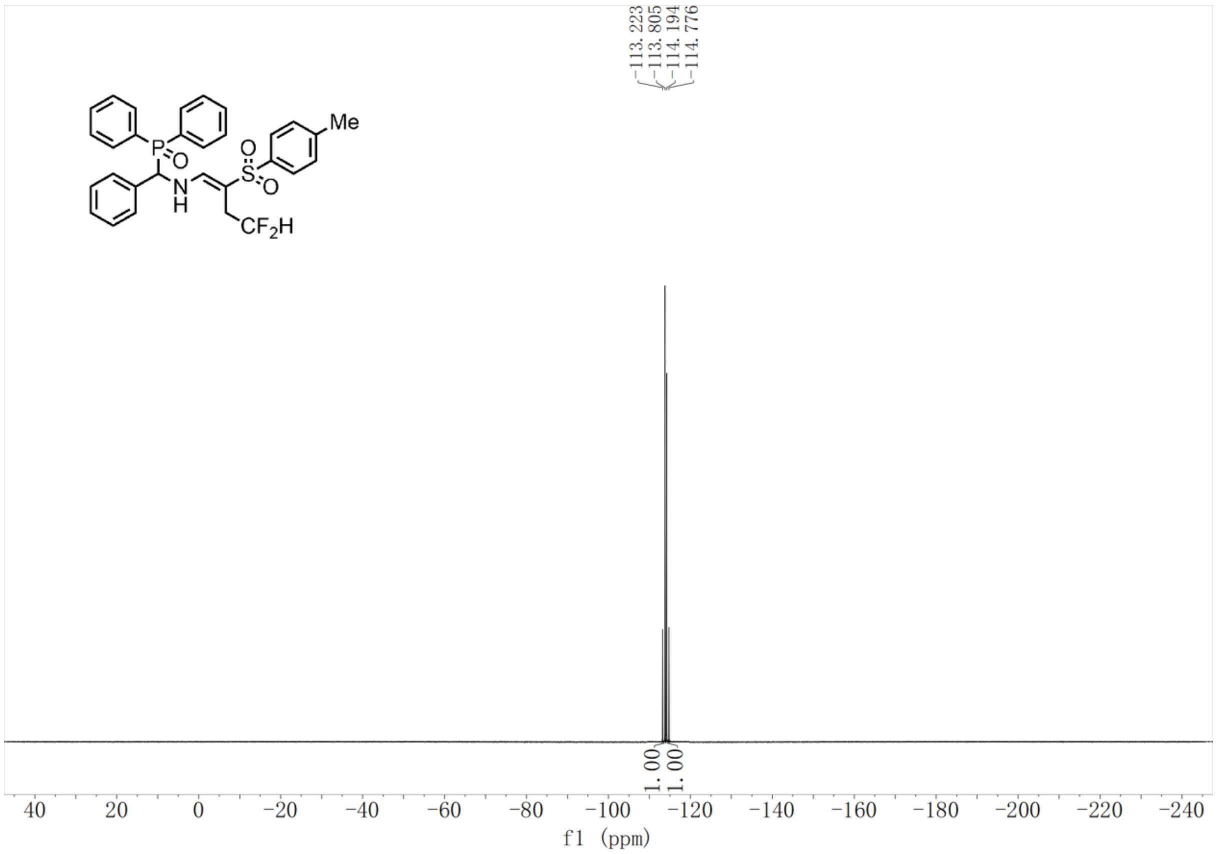

4r – ^{31}P NMR (202 MHz, DMSO)

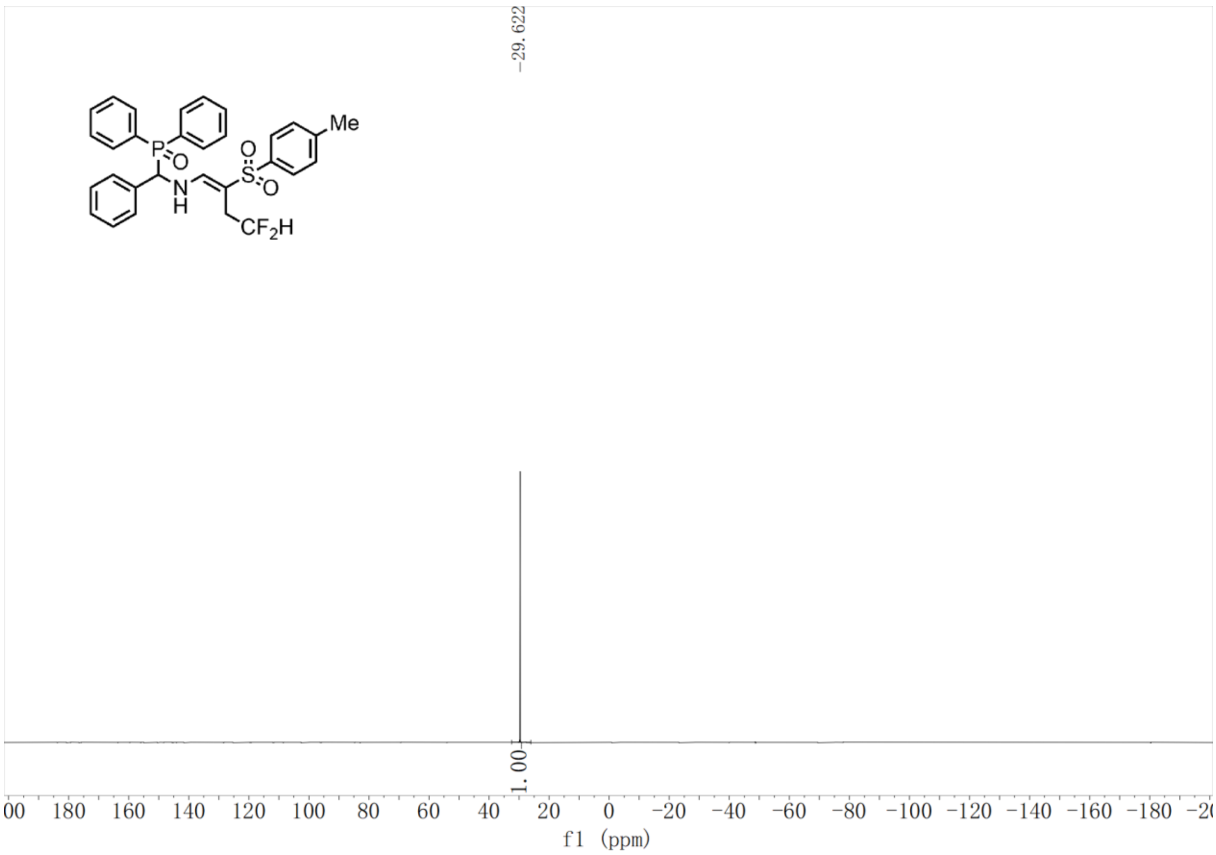

4t – ^{1}H NMR (500 MHz, DMSO)

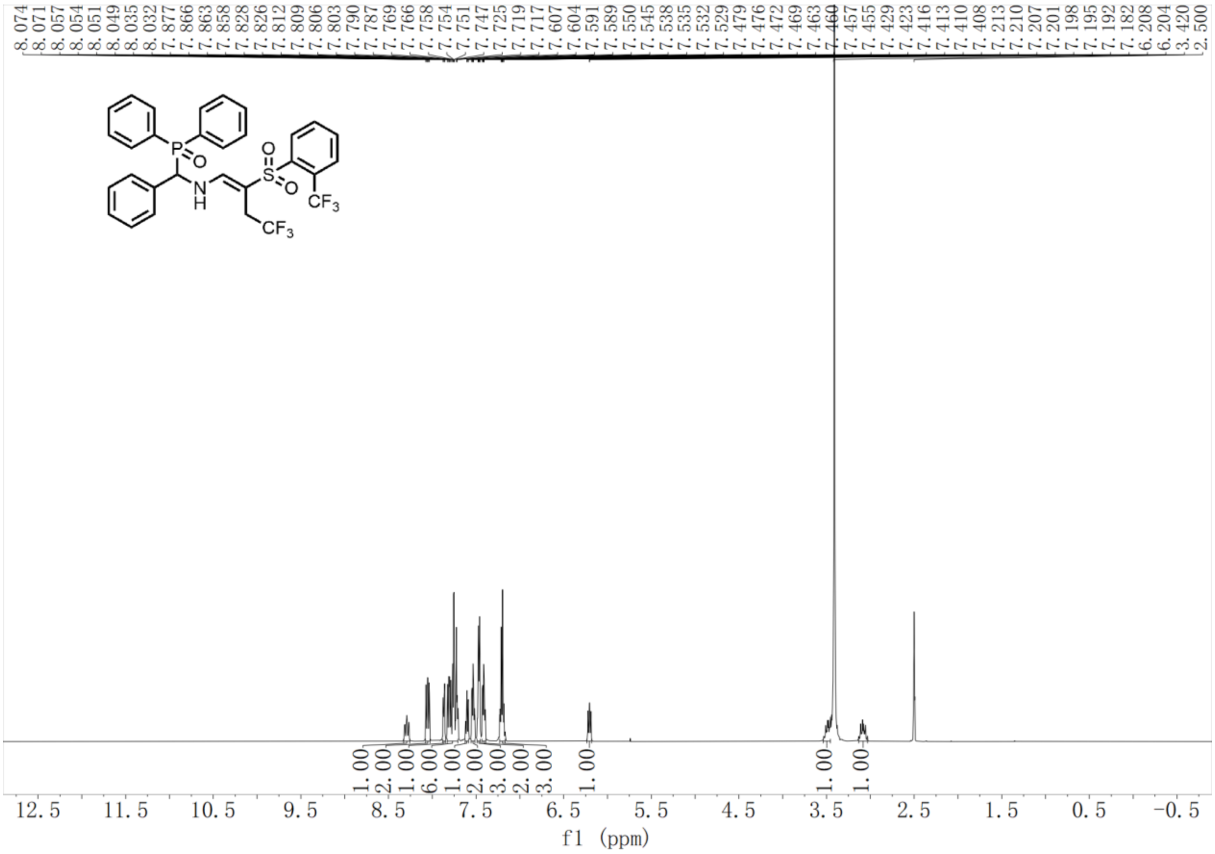

4t – ^{13}C NMR (126 MHz, DMSO)

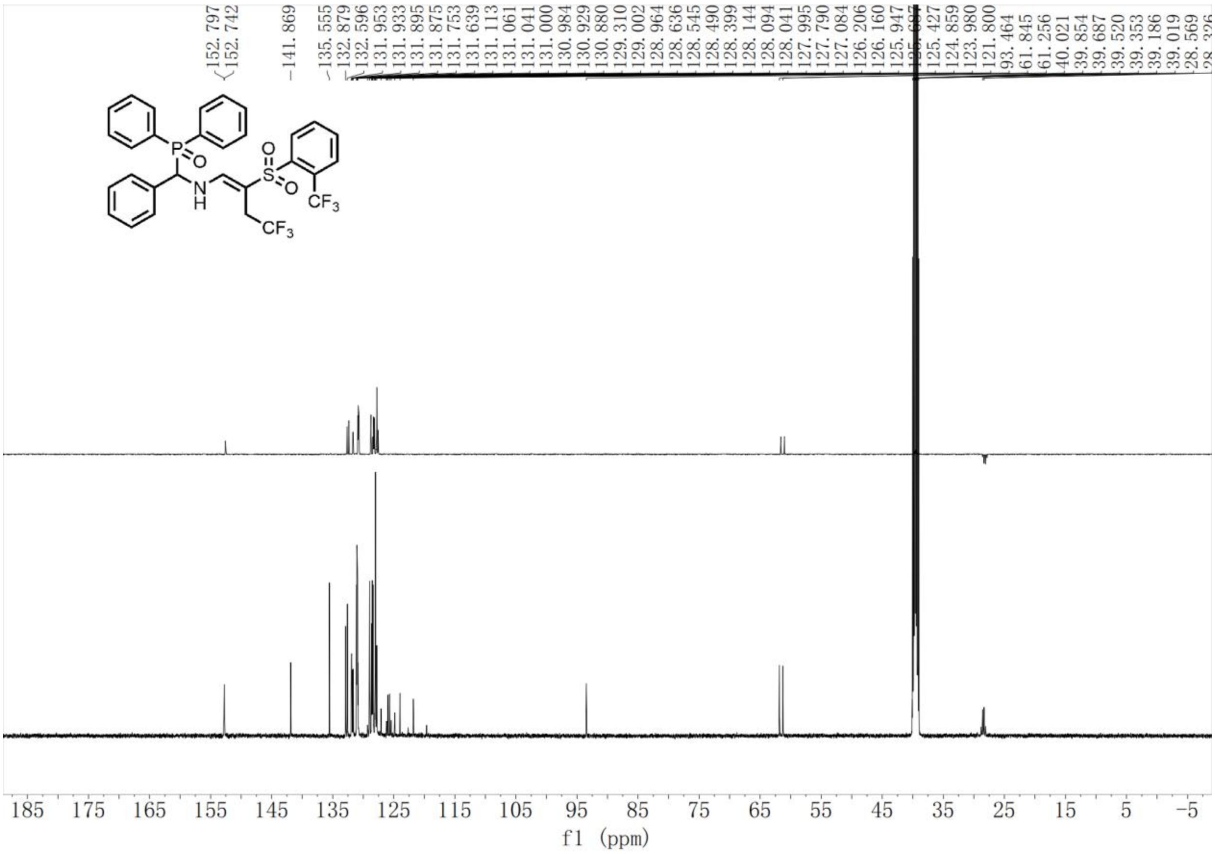

4t – ^{19}F NMR (282 MHz, DMSO)

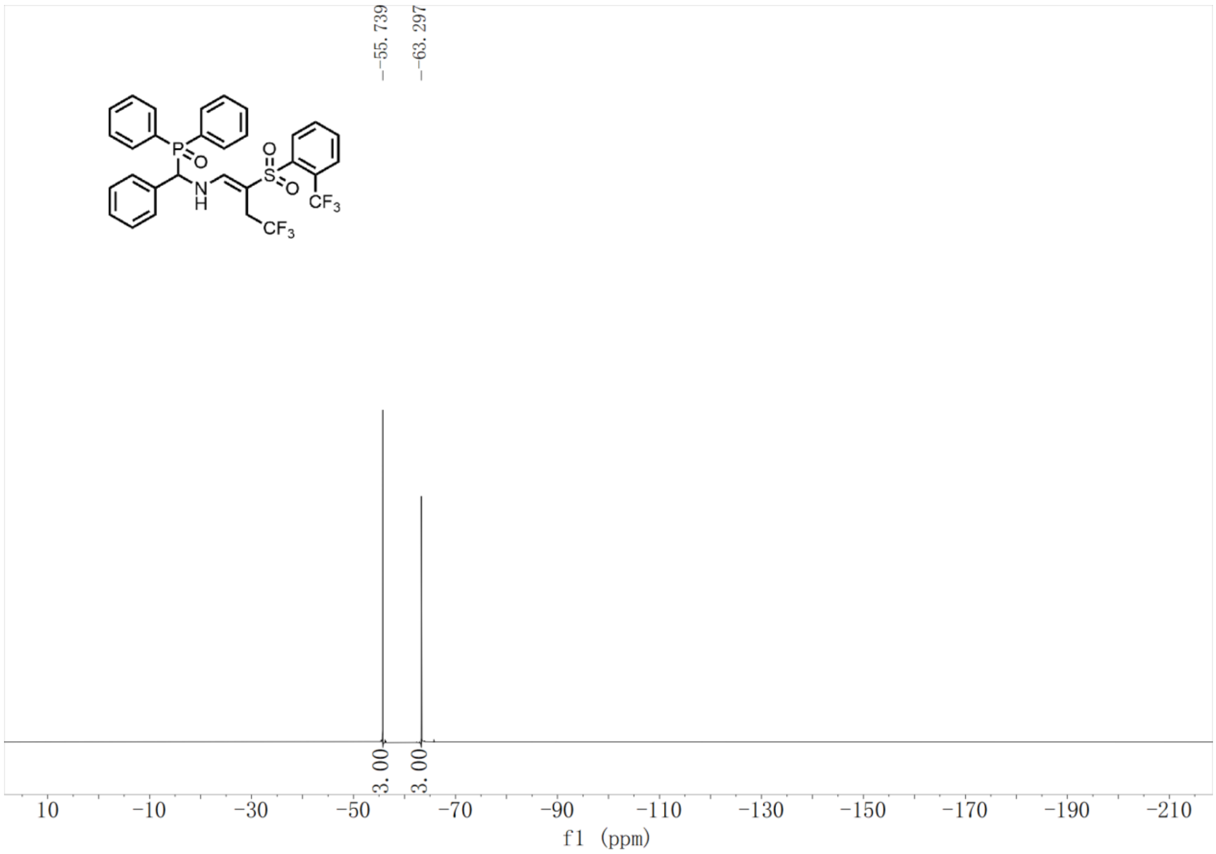

4t – ^{31}P NMR (121 MHz, DMSO)

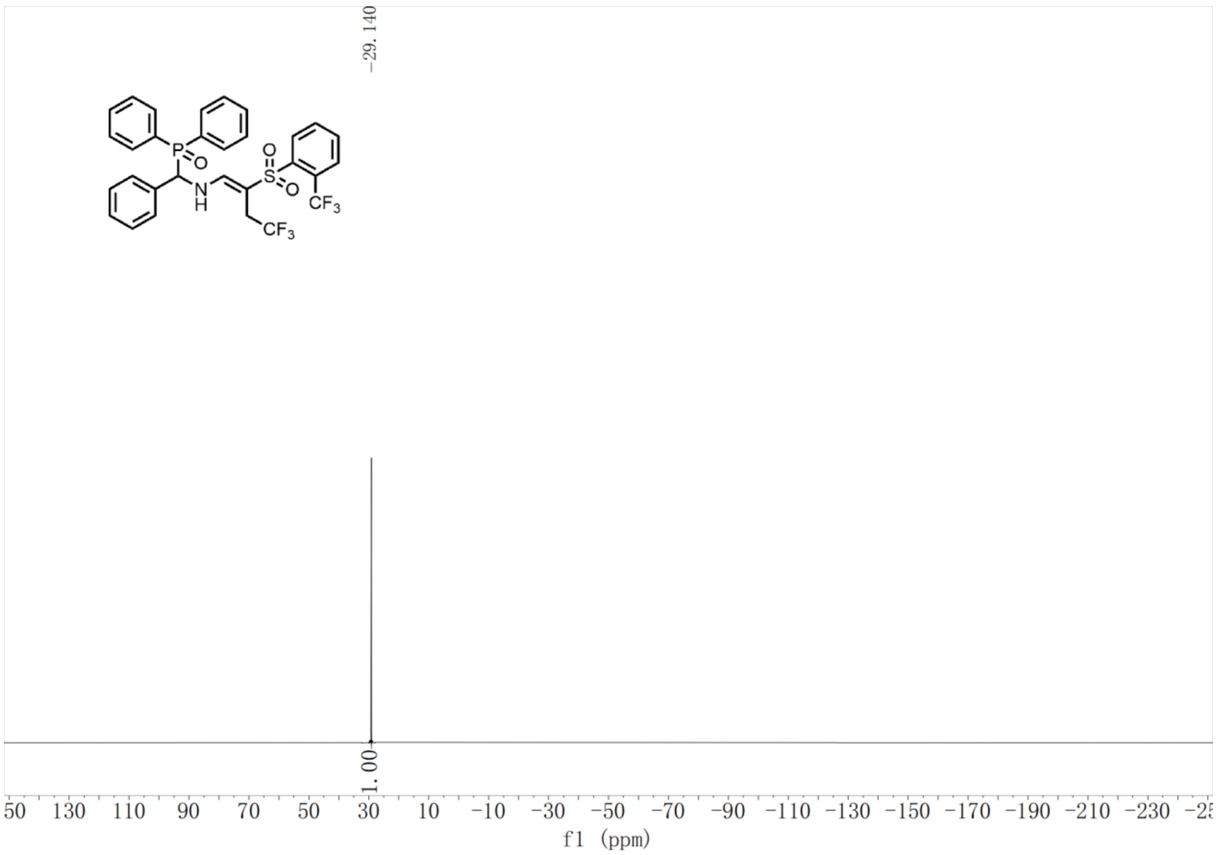

4u – ^{1}H NMR (500 MHz, DMSO)

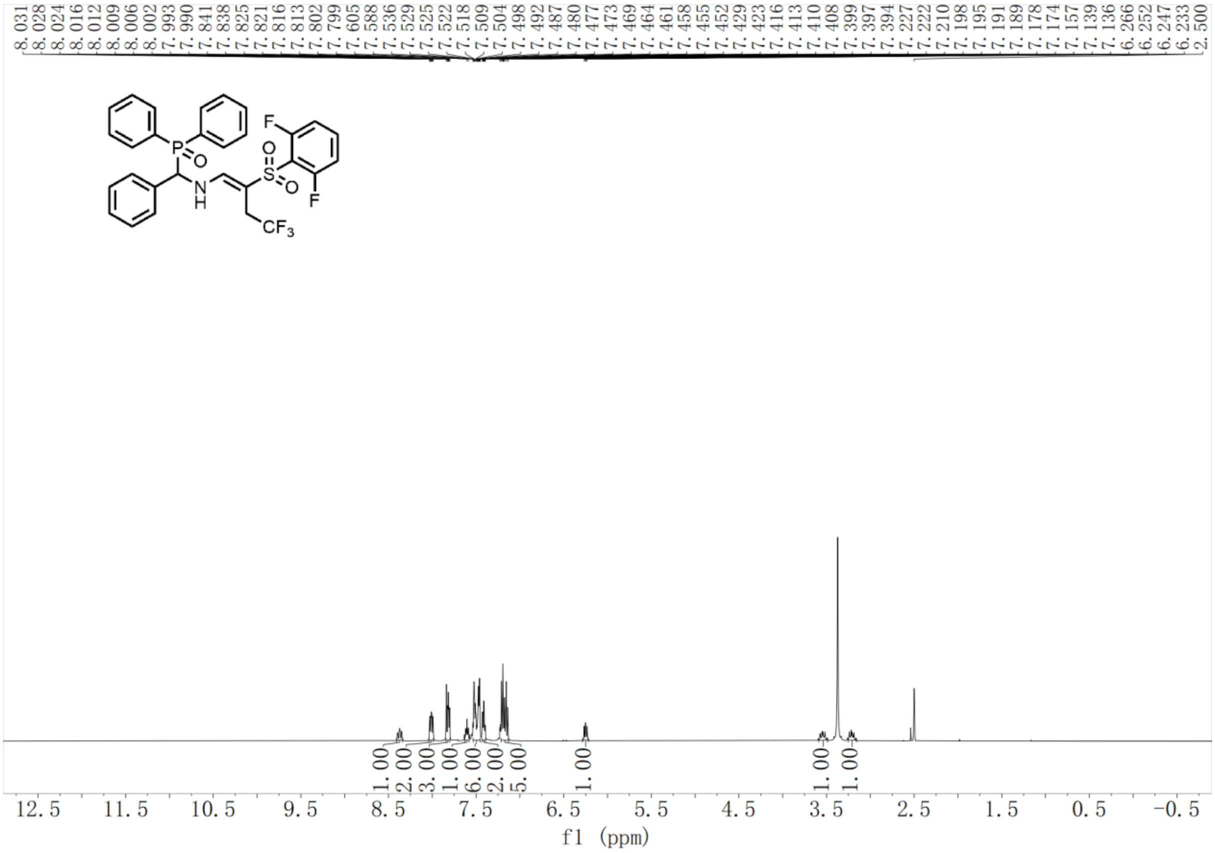

4u – ^{13}C NMR (126 MHz, DMSO)

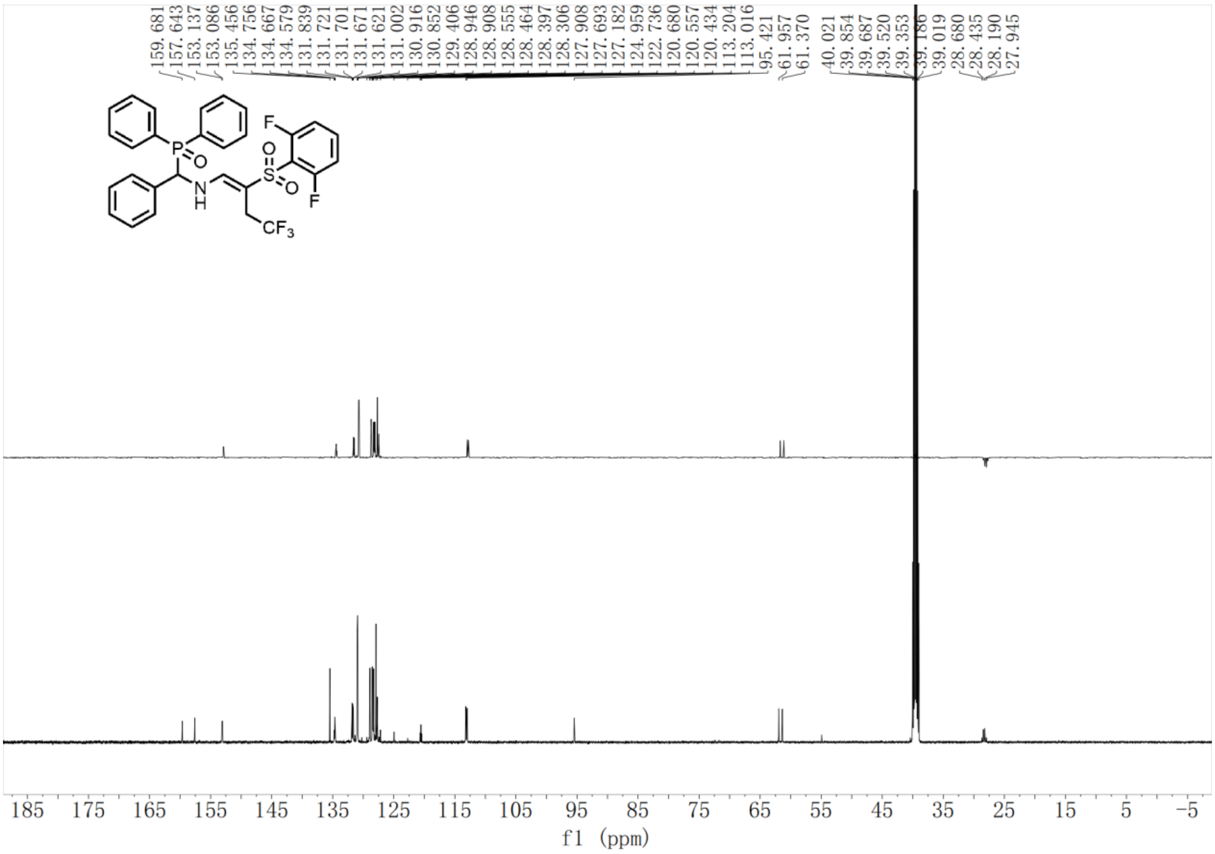

4u – ^{19}F NMR (282 MHz, DMSO)

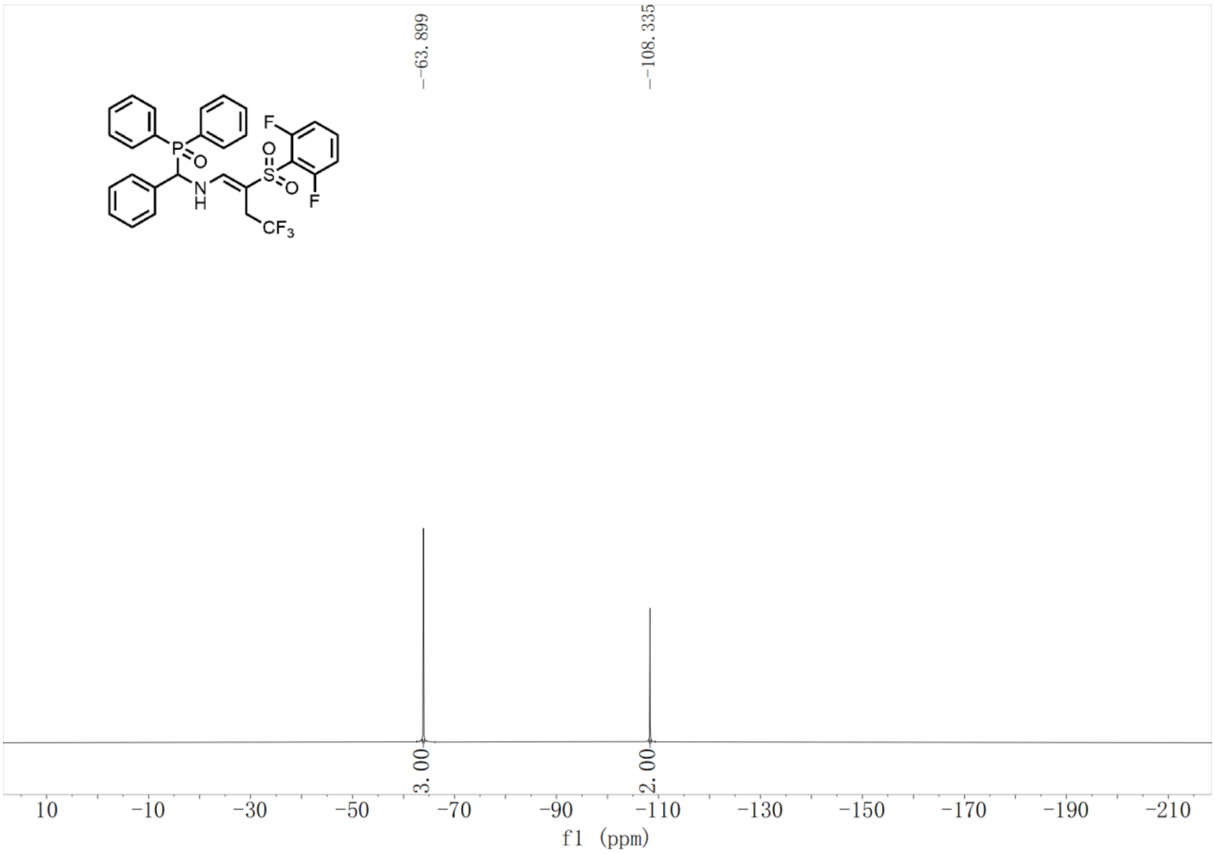

4u – ^{31}P NMR (121 MHz, DMSO)

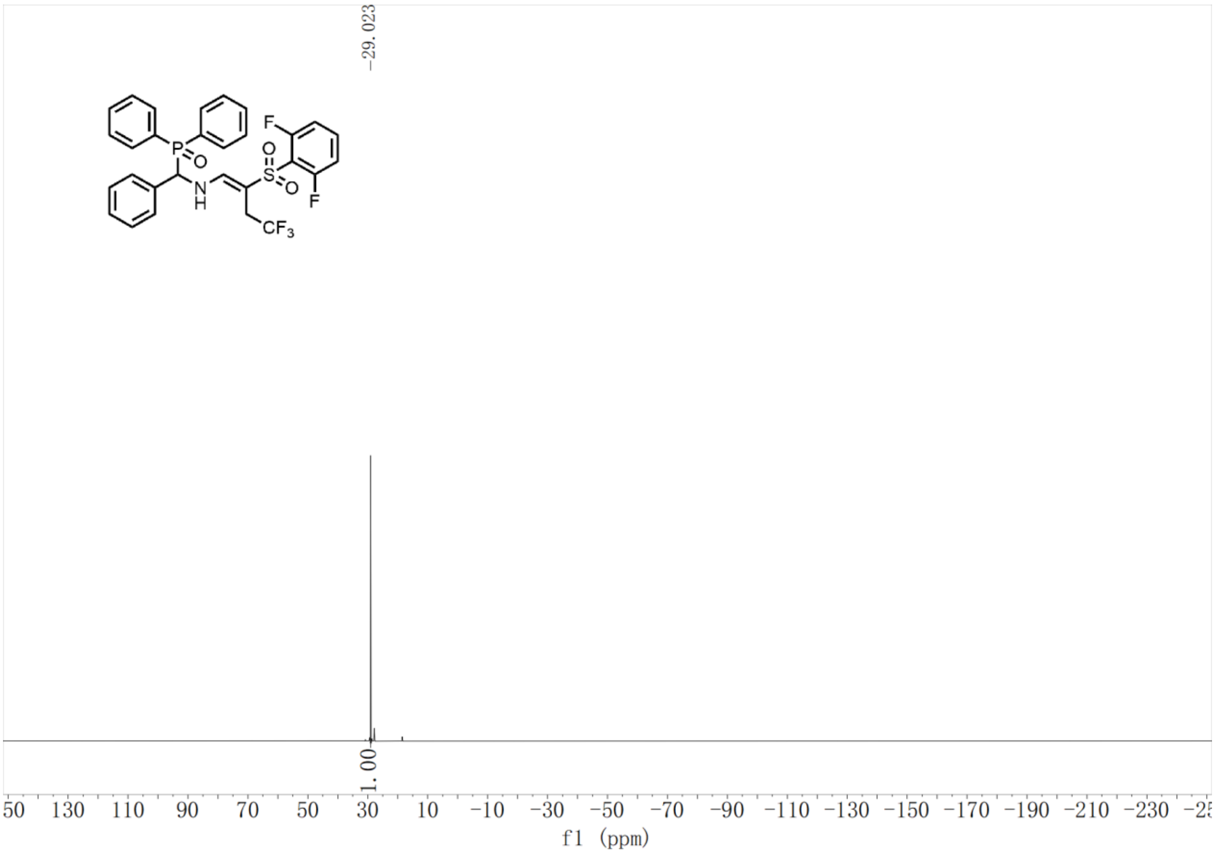

4v – ^{1}H NMR (500 MHz, DMSO)

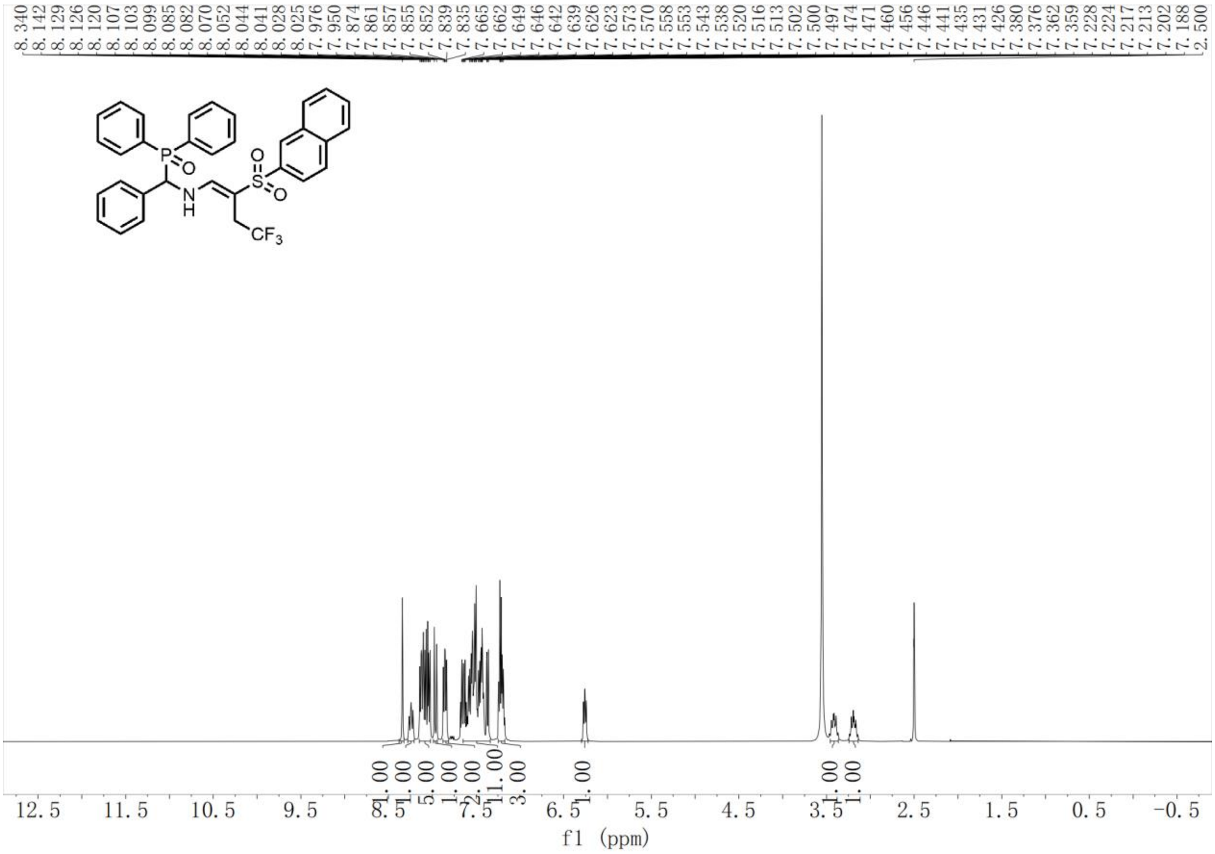

4v – ^{13}C NMR (126 MHz, DMSO)

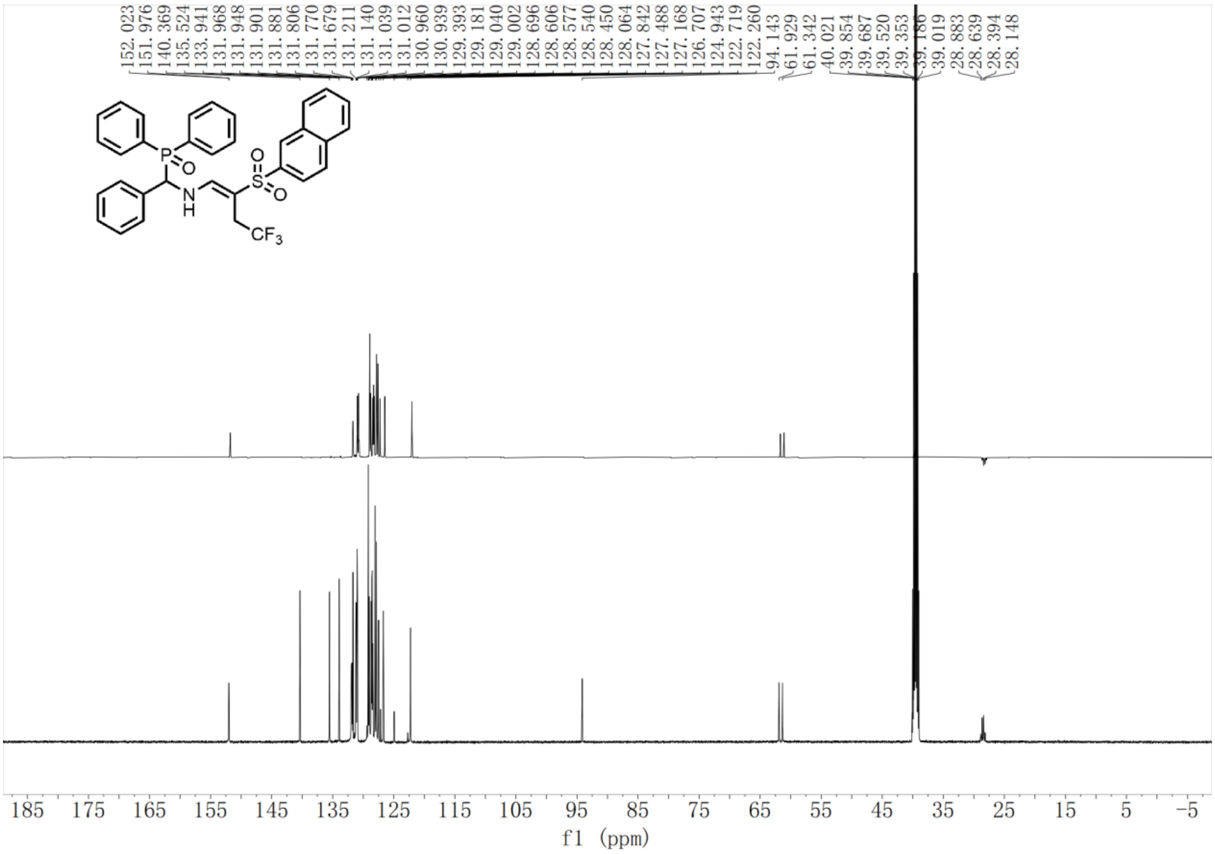

4v – ^{19}F NMR (471 MHz, DMSO)

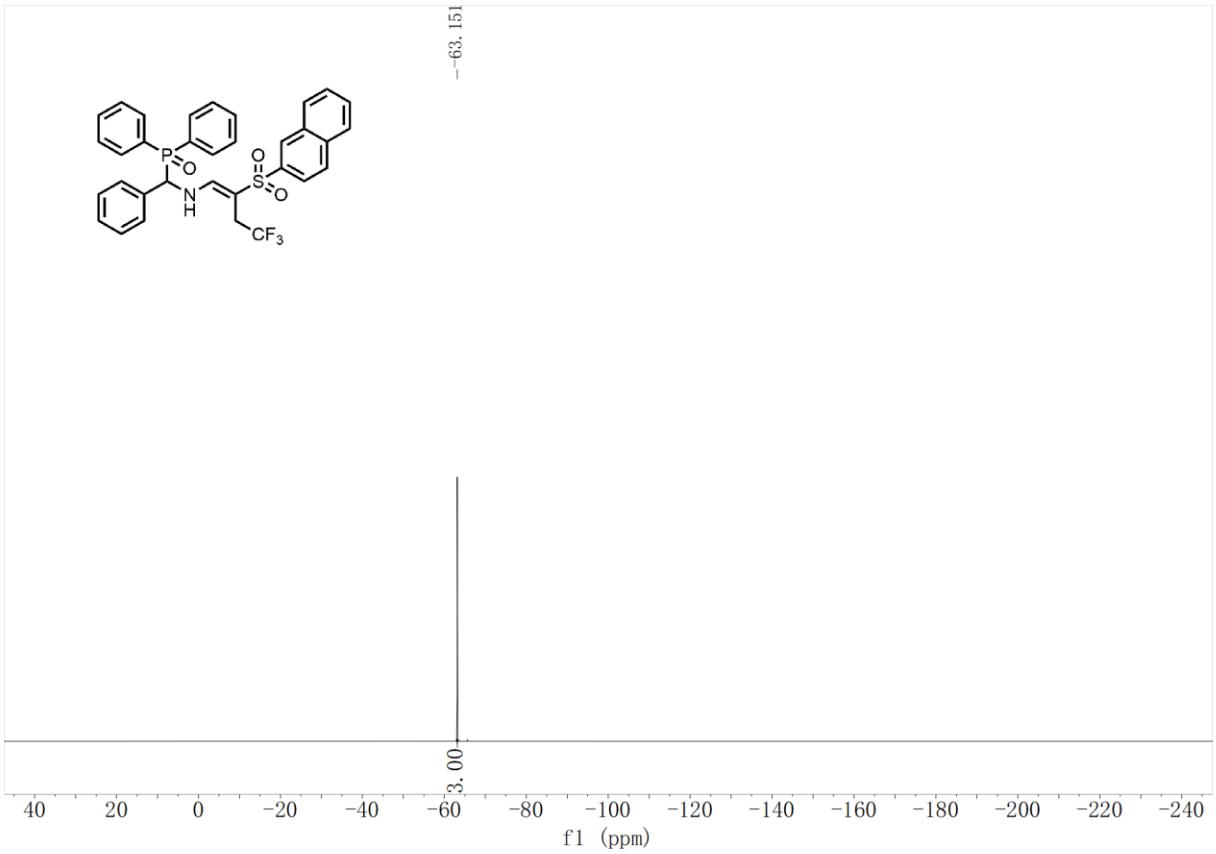

4v – ^{31}P NMR (202 MHz, DMSO)

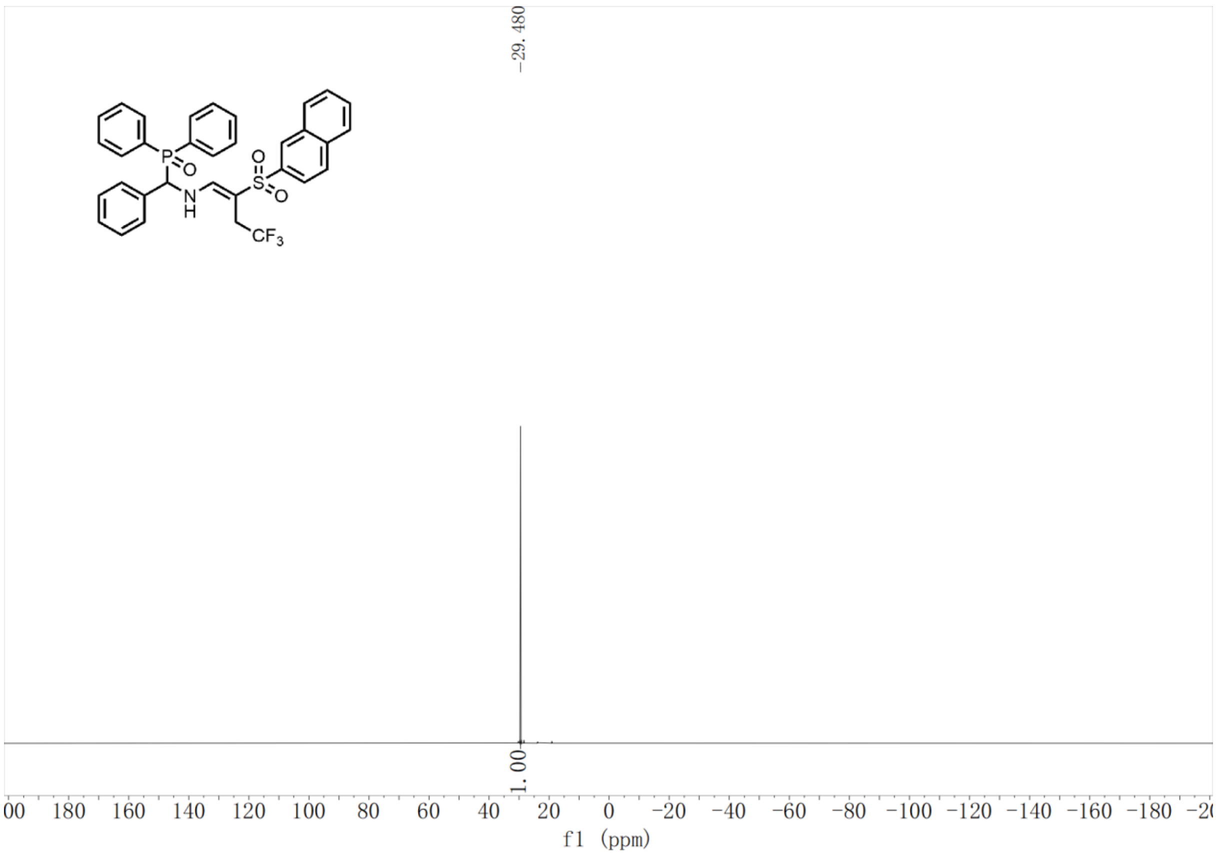

4w – ^{1}H NMR (500 MHz, DMSO)

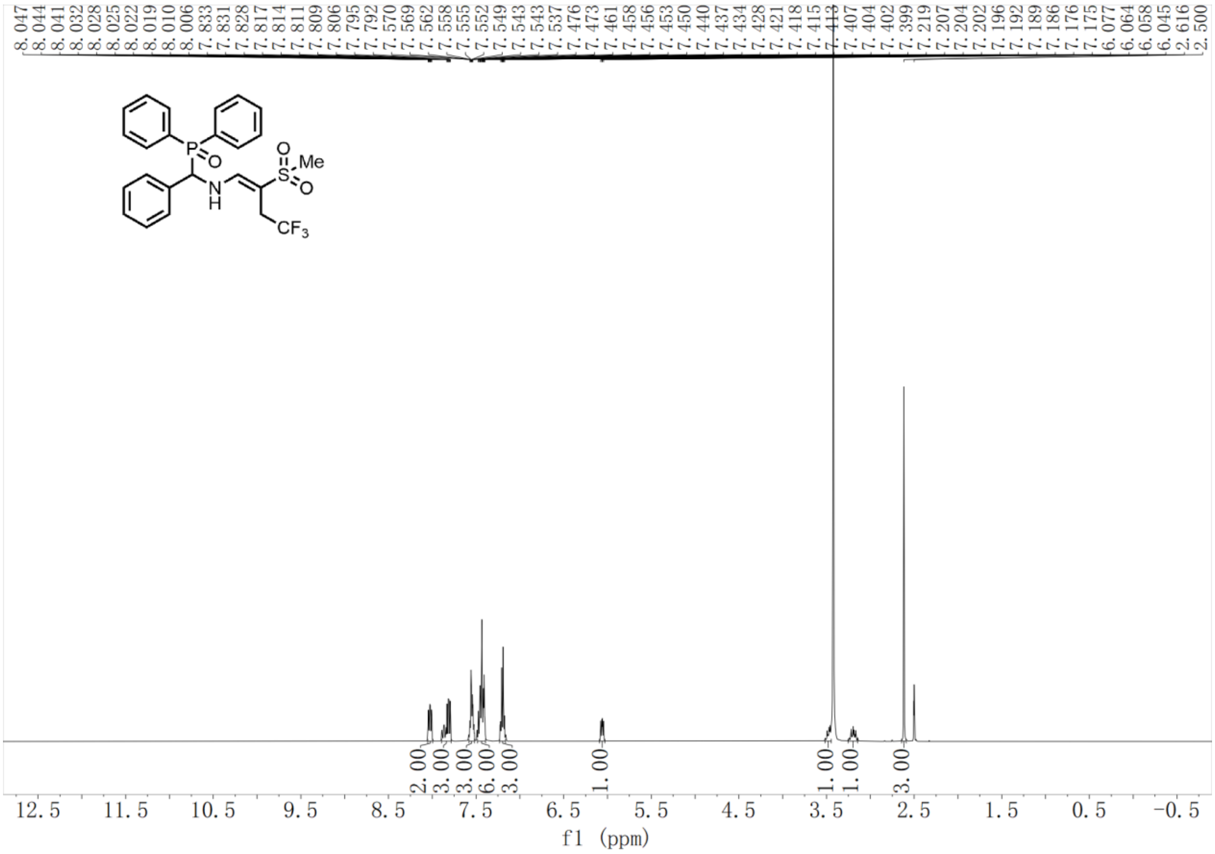

4w – ^{13}C NMR (126 MHz, DMSO)

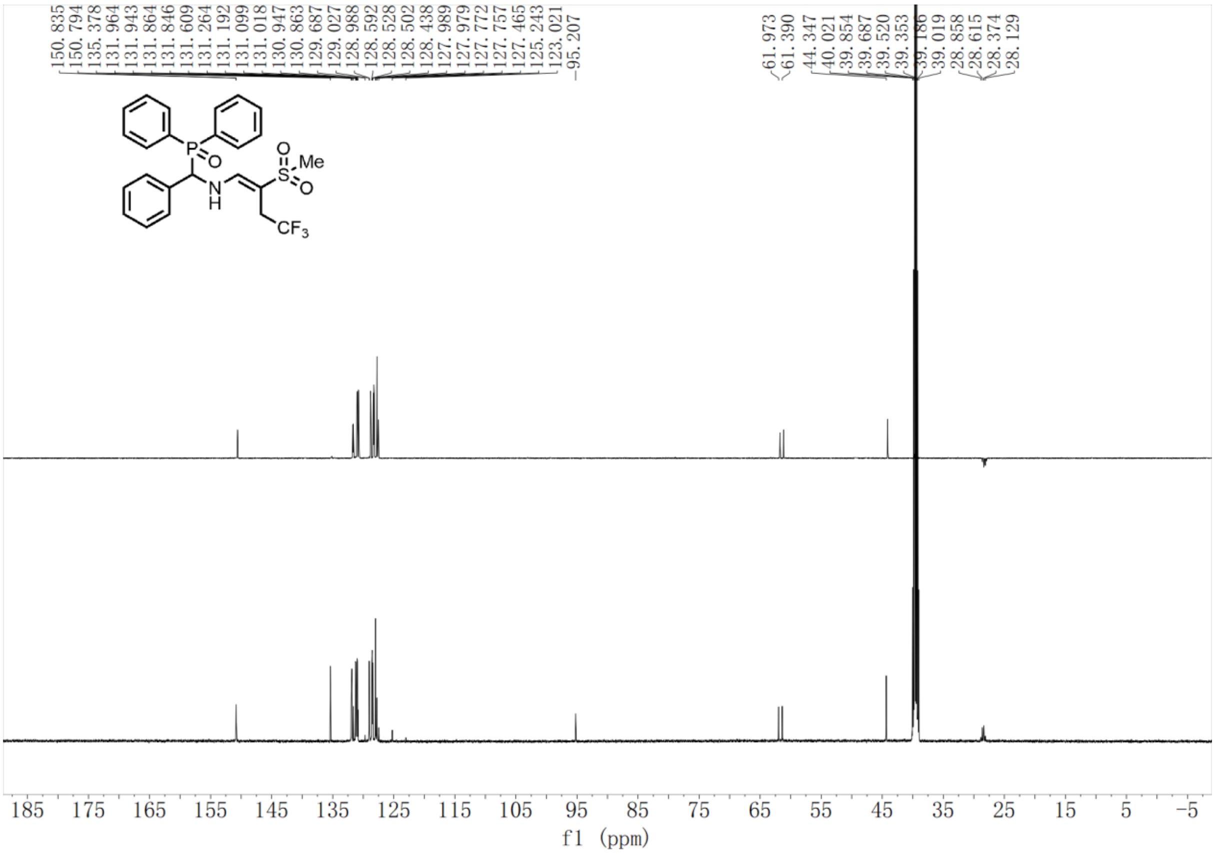

4w – ^{19}F NMR (471 MHz, DMSO)

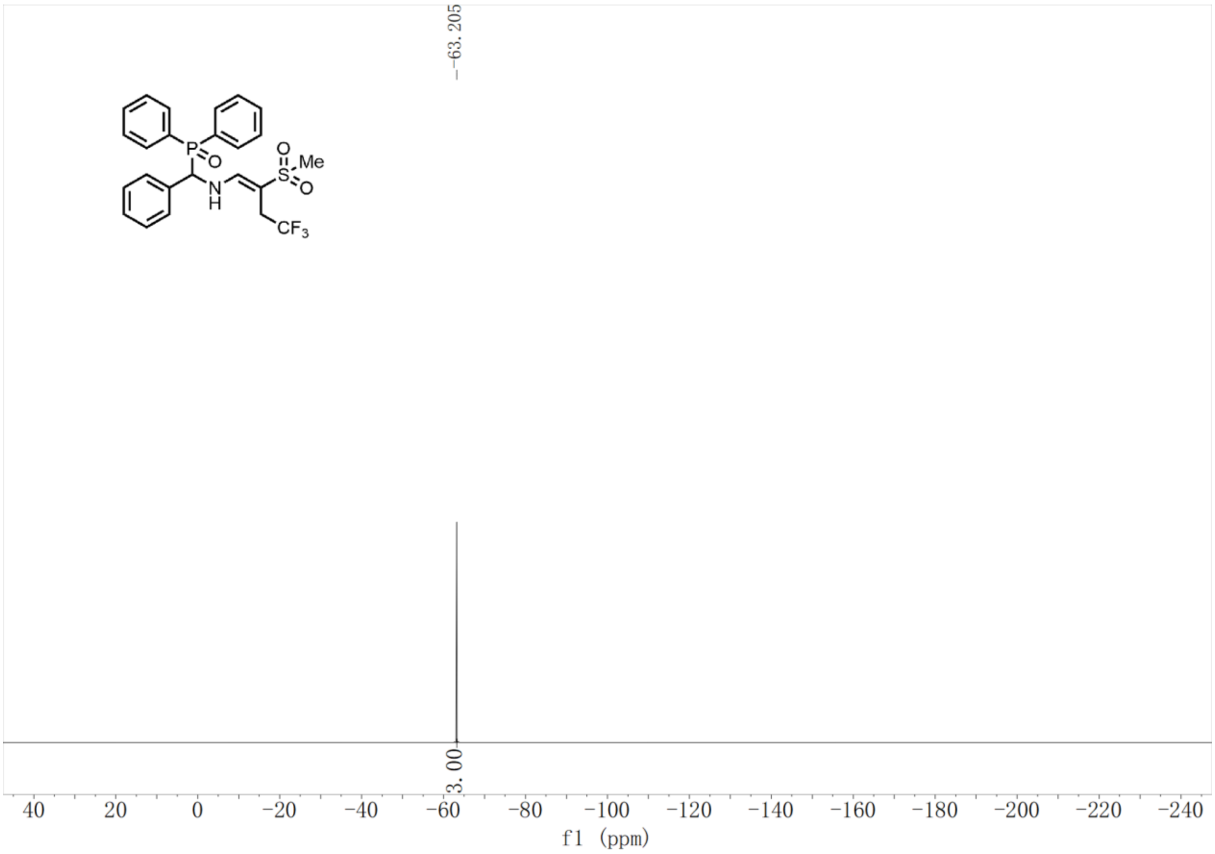

4w – ^{31}P NMR (202 MHz, DMSO)

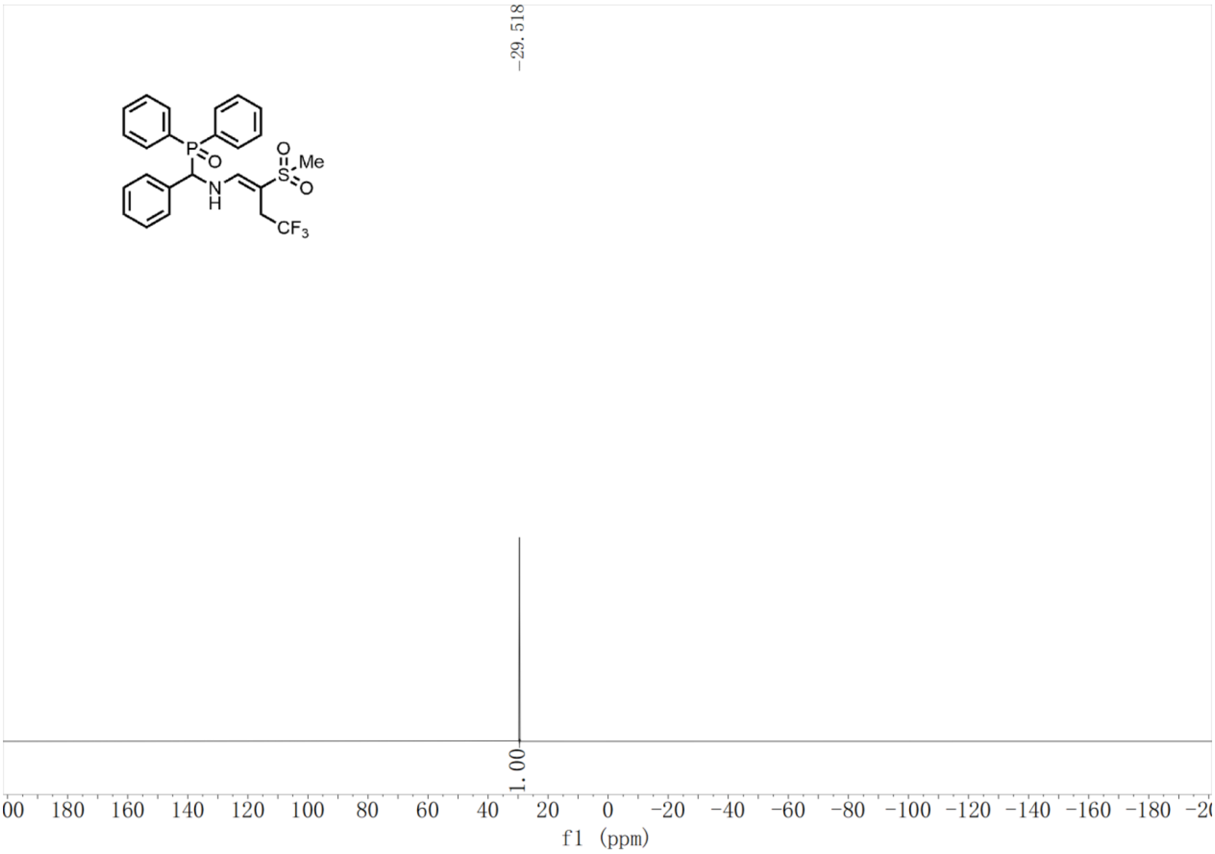

4x – ^{1}H NMR (500 MHz, DMSO)

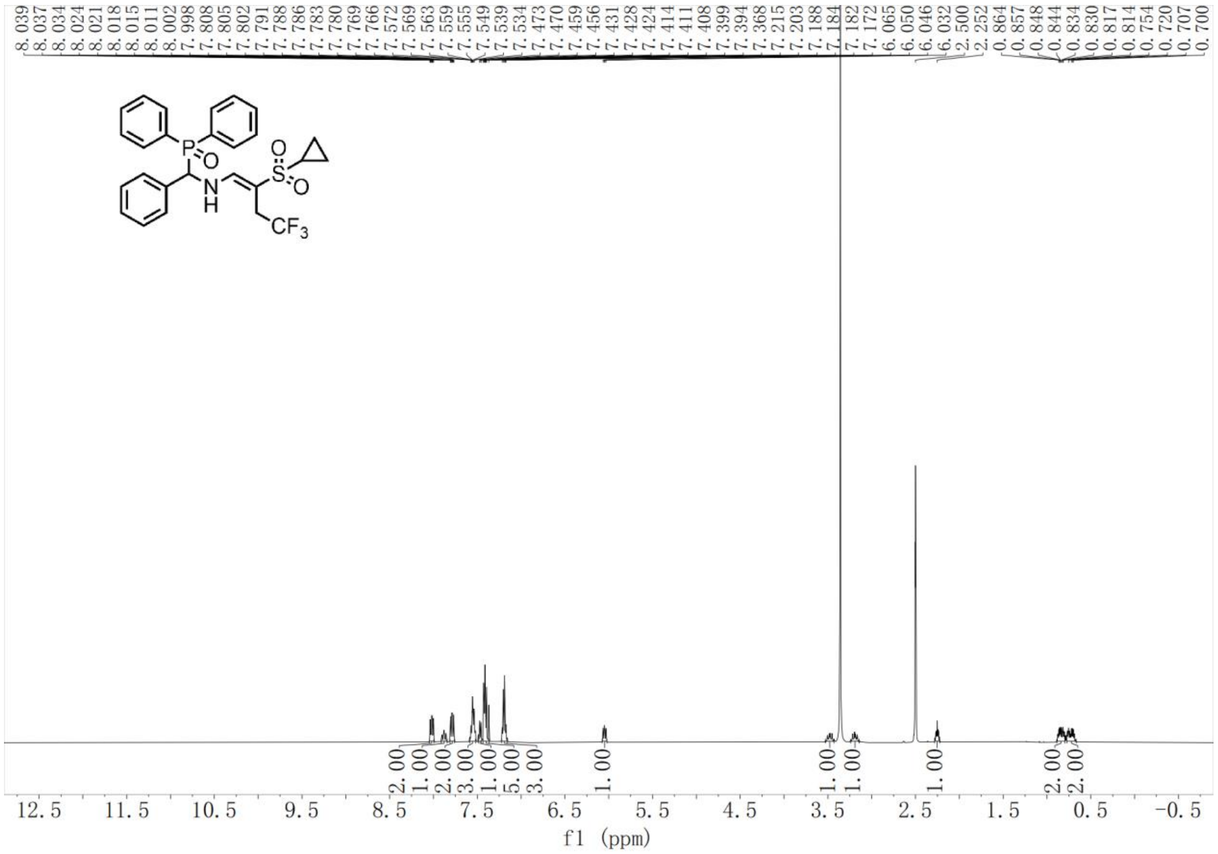

4x – ^{13}C NMR (126 MHz, DMSO)

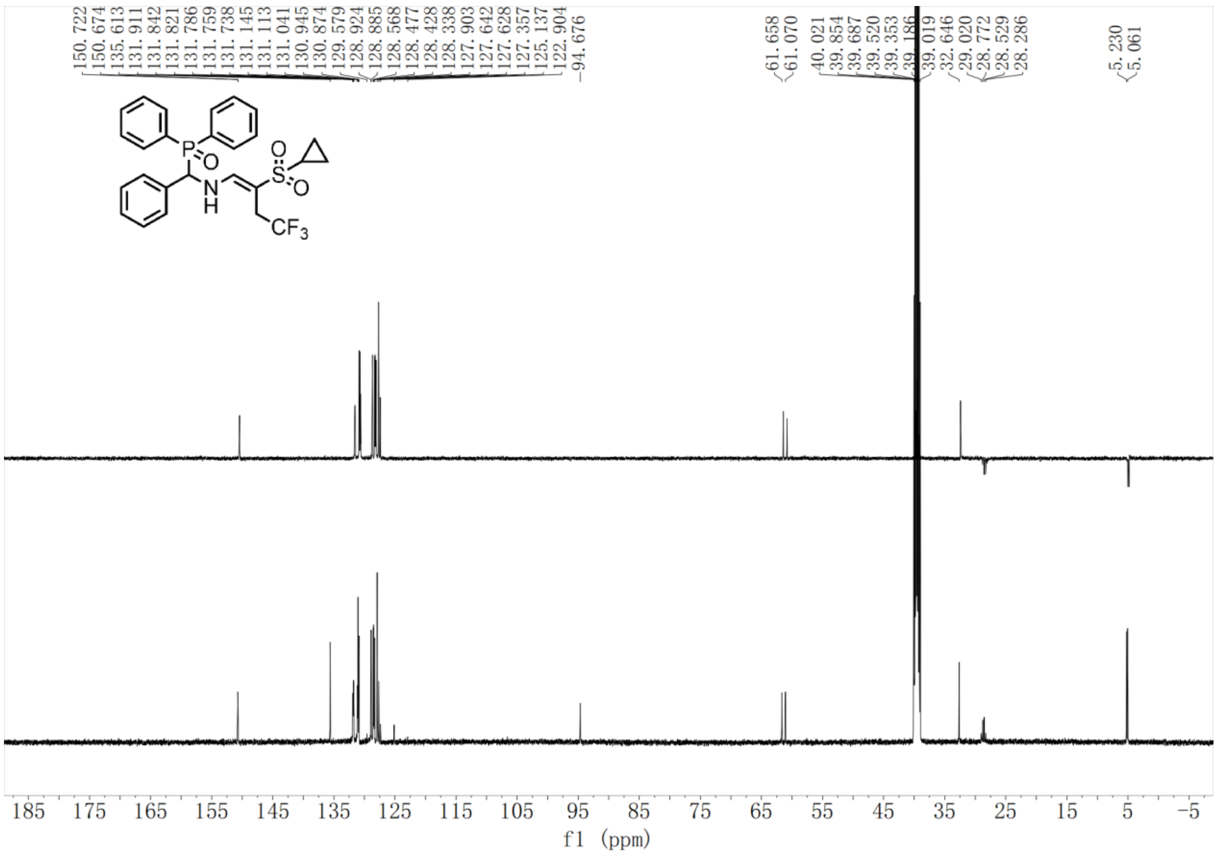

4x – ^{19}F NMR (471 MHz, DMSO)

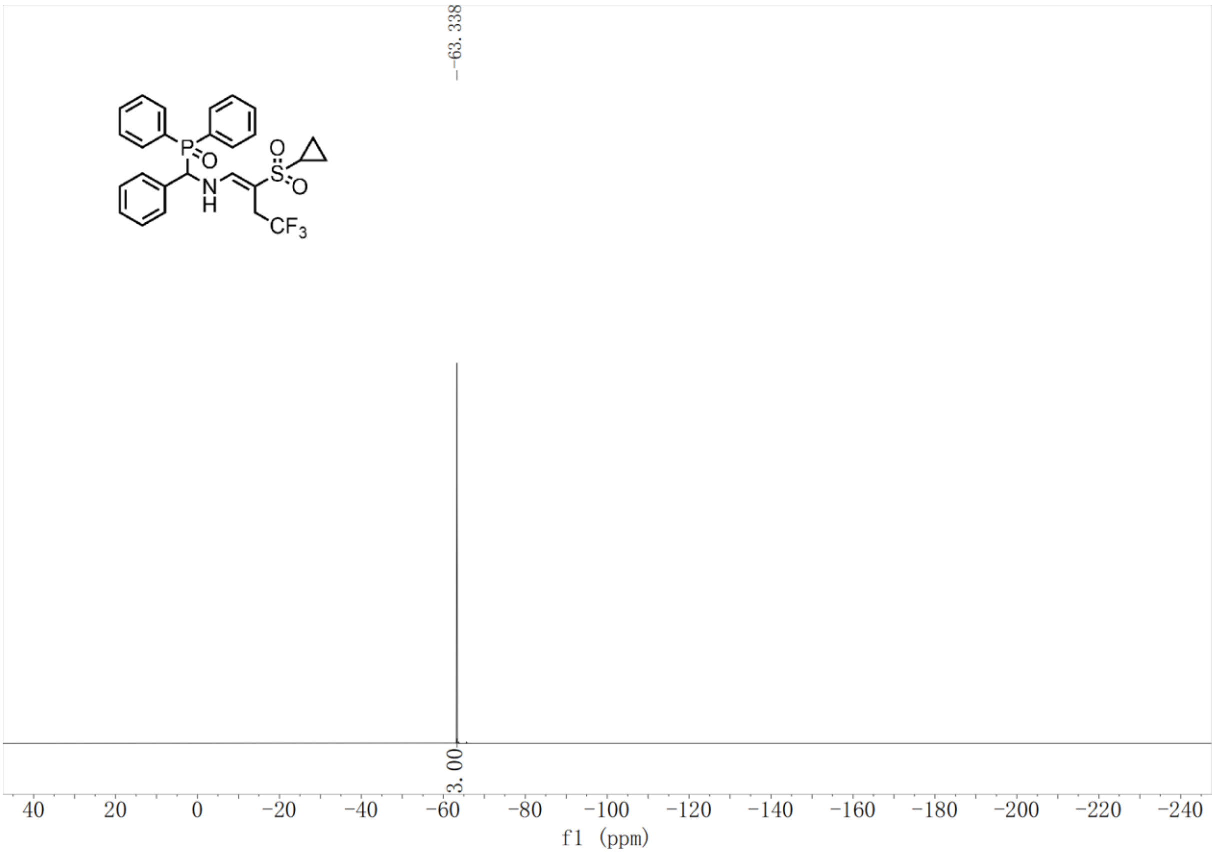

4x – ^{31}P NMR (202 MHz, DMSO)

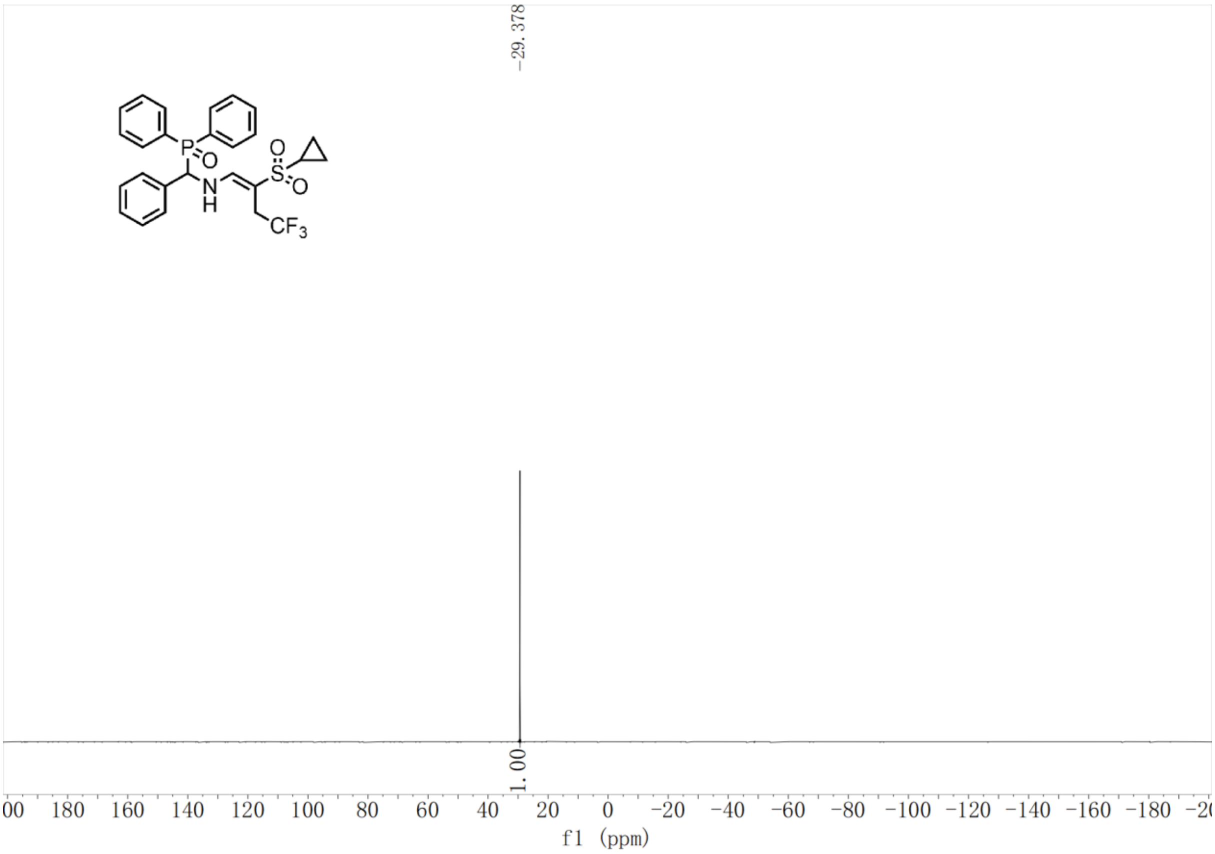

4y – ^{1}H NMR (500 MHz, DMSO)

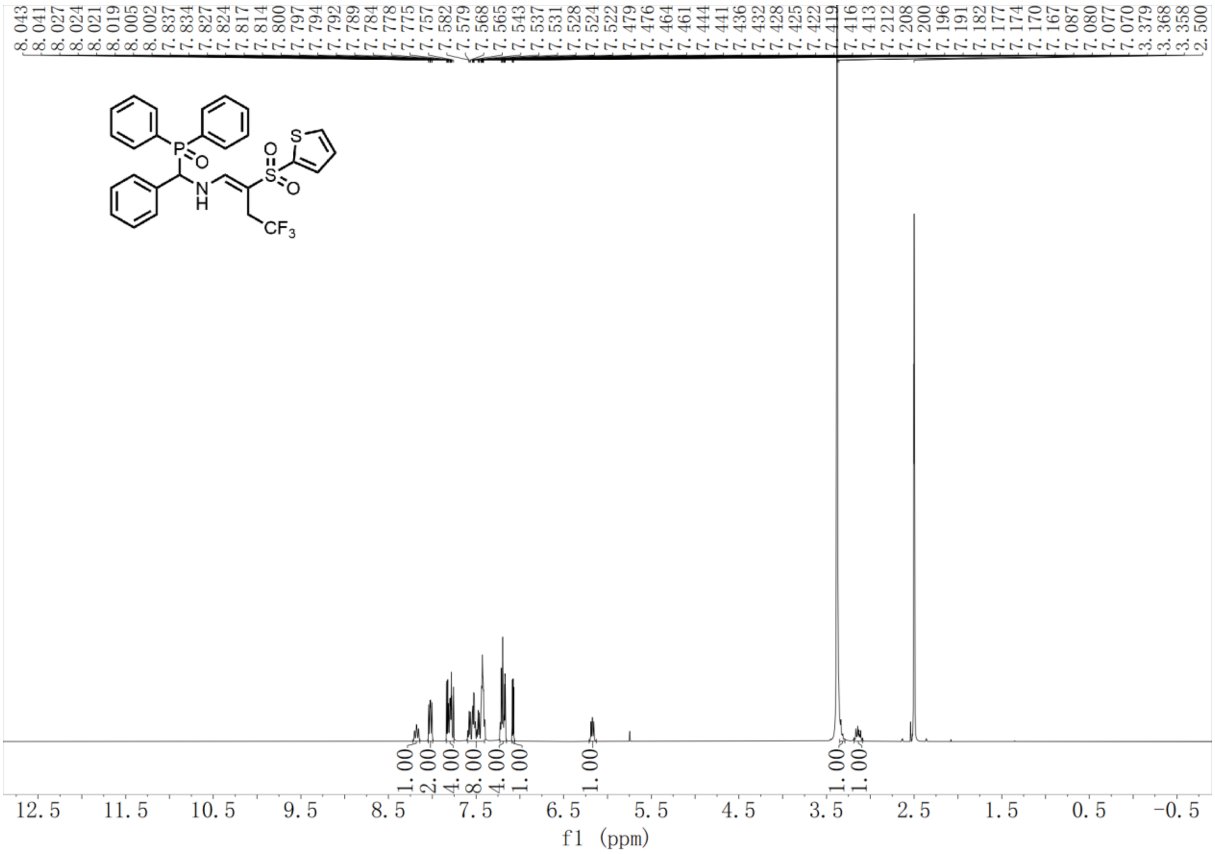

4y – ^{13}C NMR (126 MHz, DMSO)

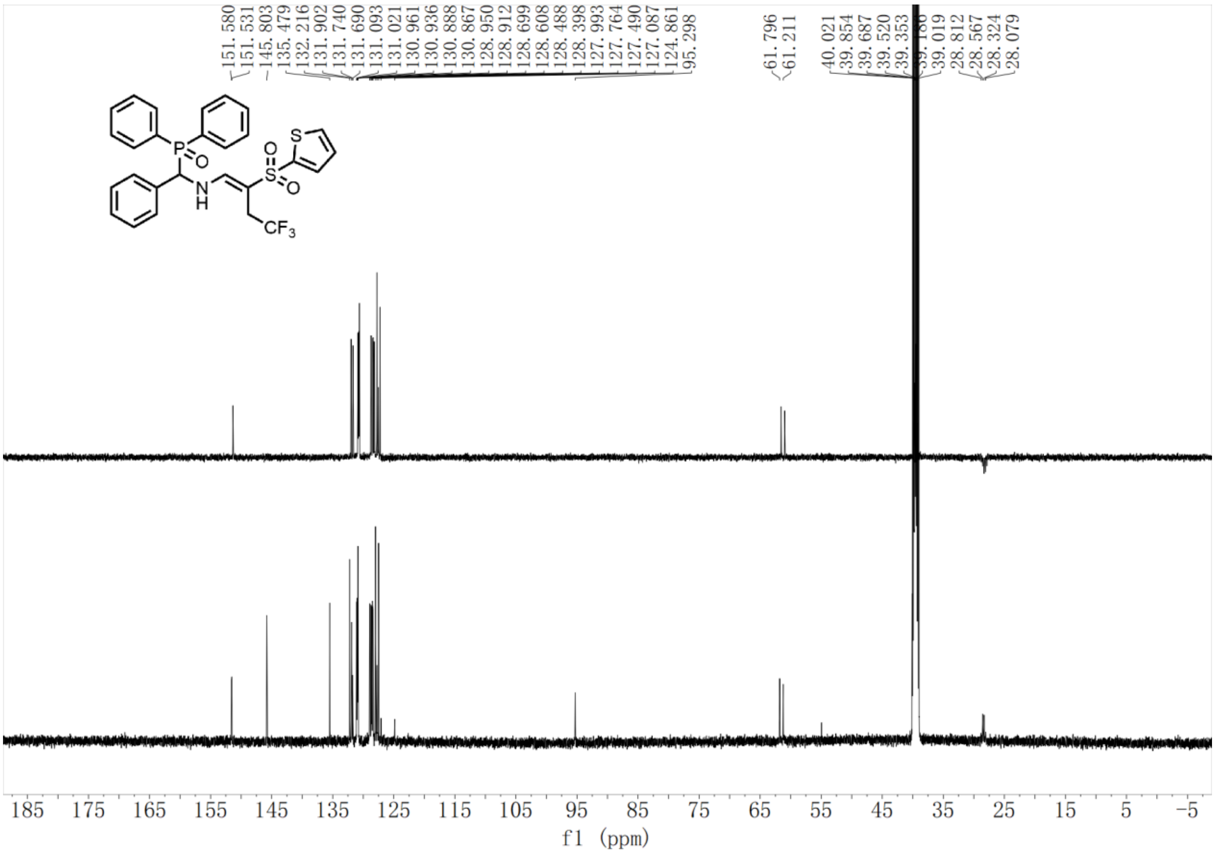

4y – ^{19}F NMR (471 MHz, DMSO)

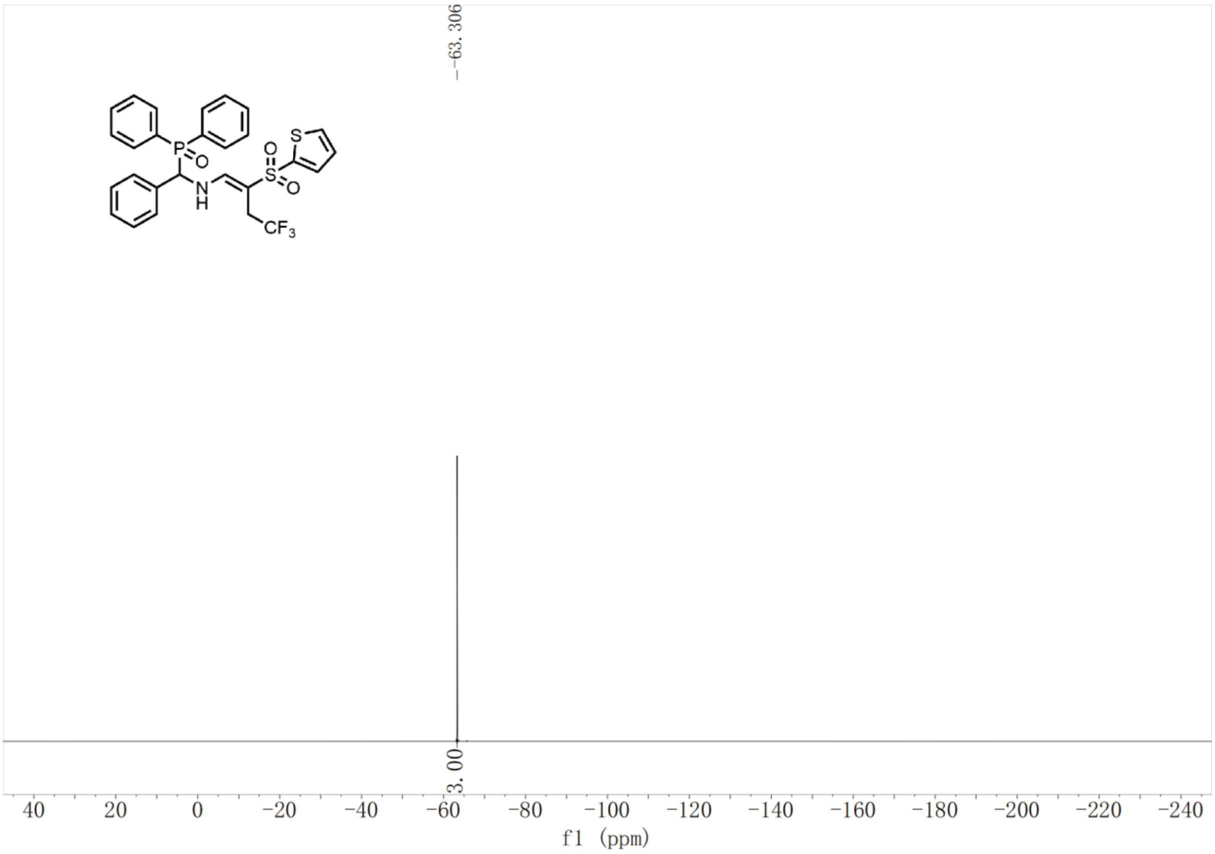

4y – ^{31}P NMR (202 MHz, DMSO)

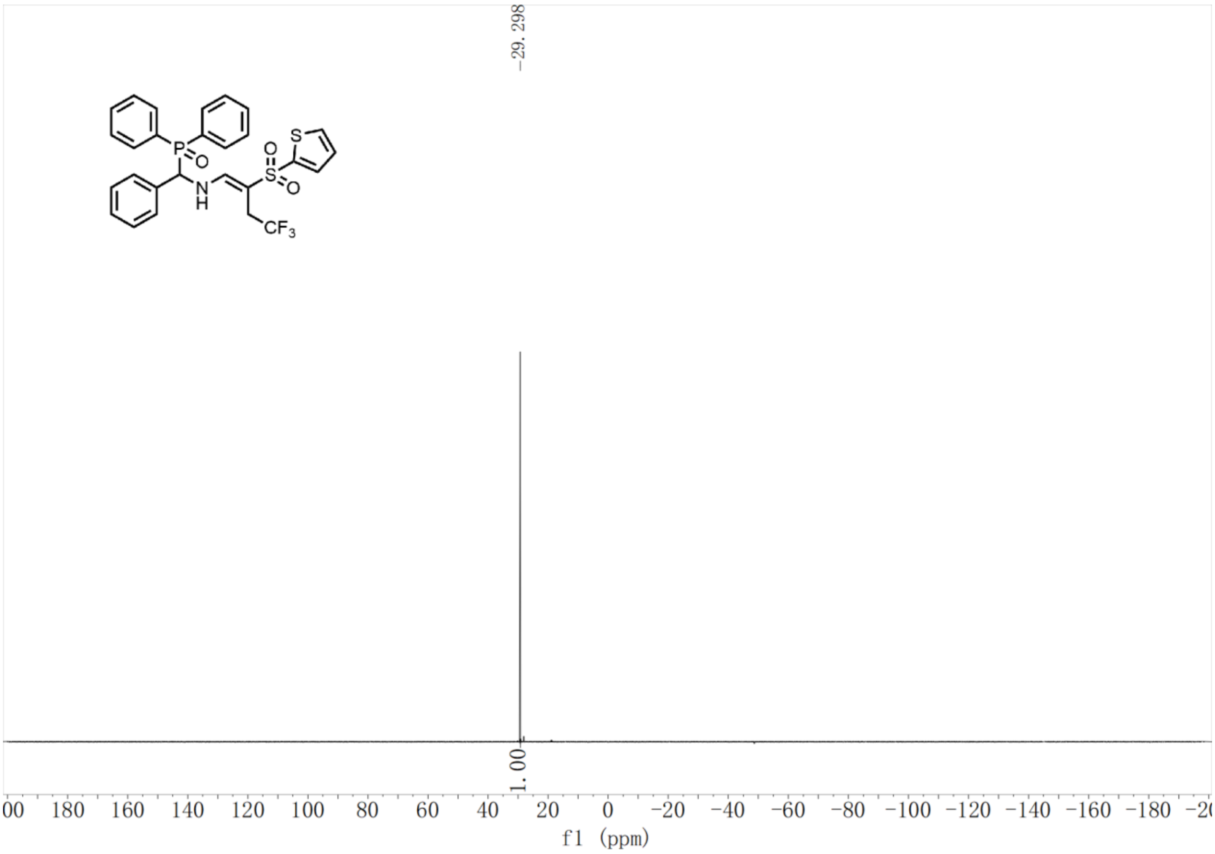

4z – ^{1}H NMR (300 MHz, DMSO)

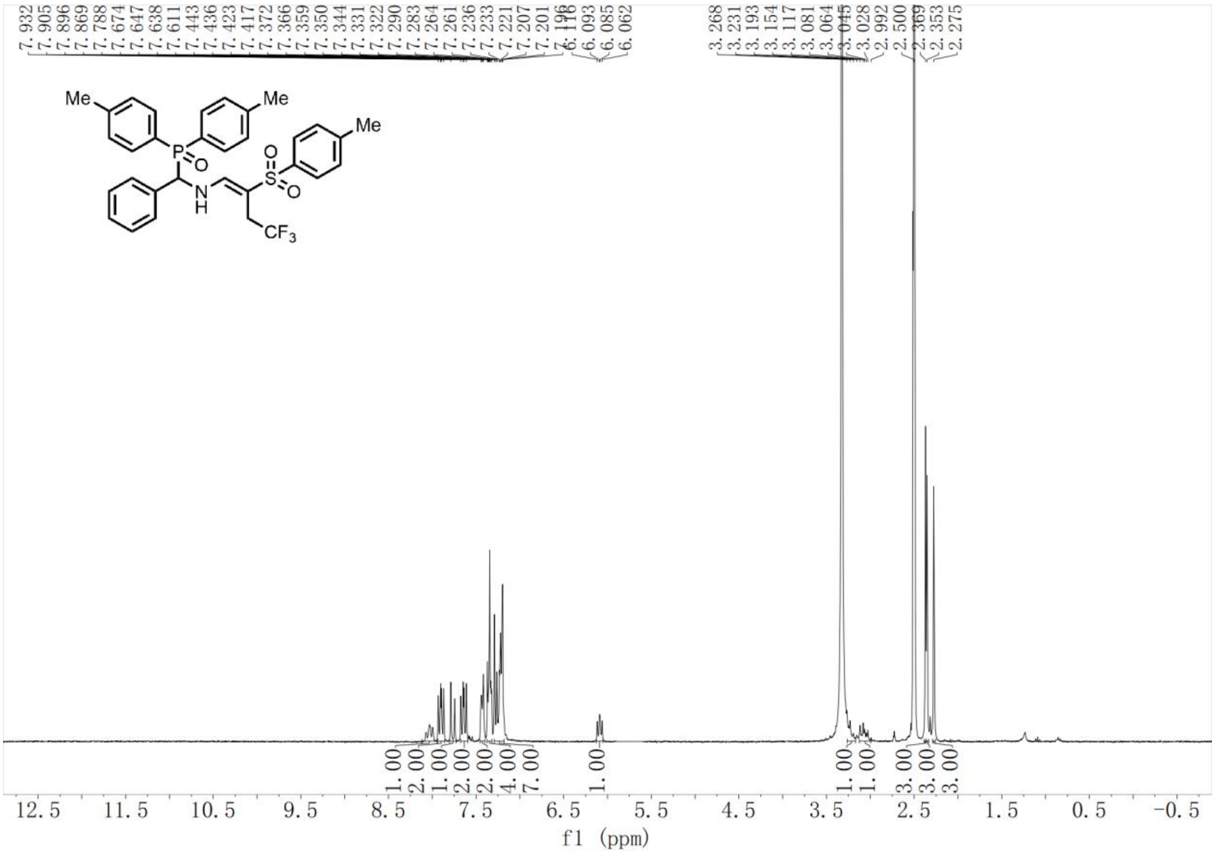

4z – ^{13}C NMR (126 MHz, DMSO)

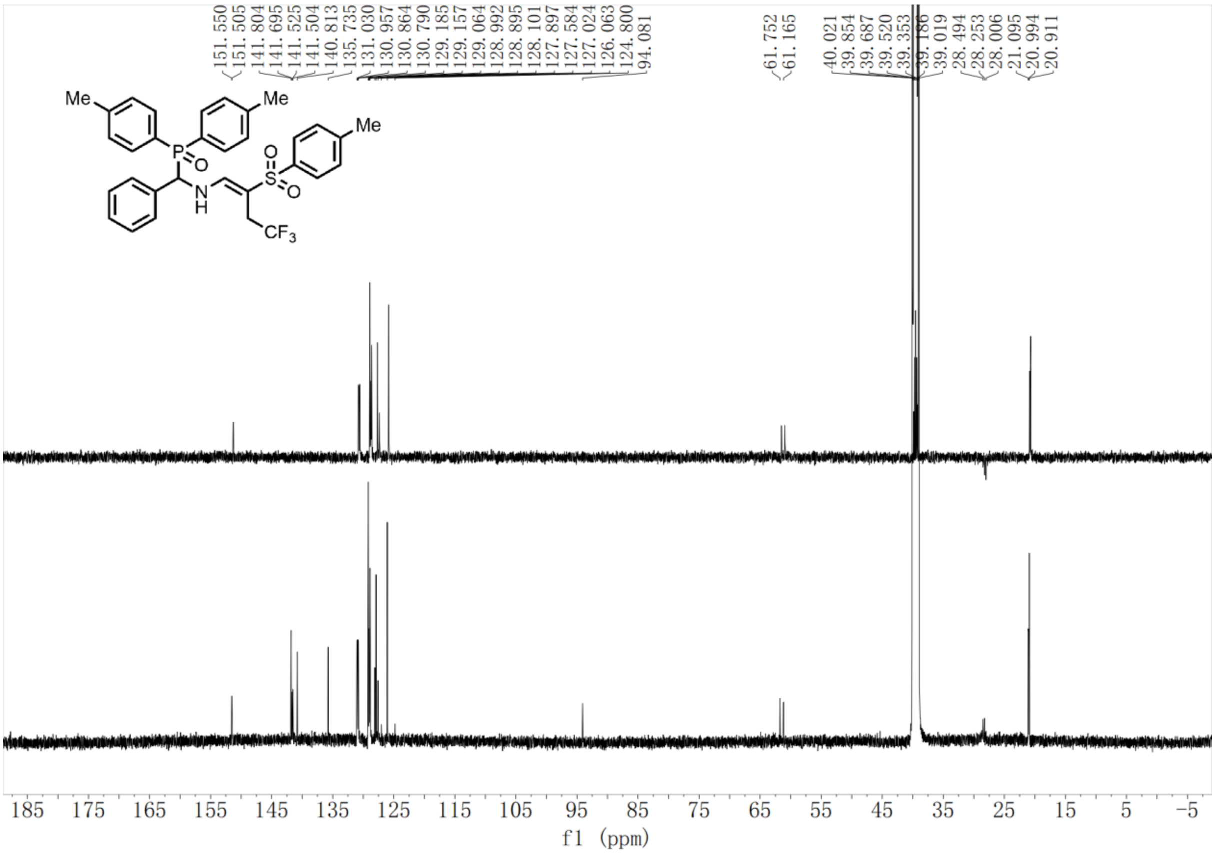

4z – ^{19}F NMR (471 MHz, DMSO)

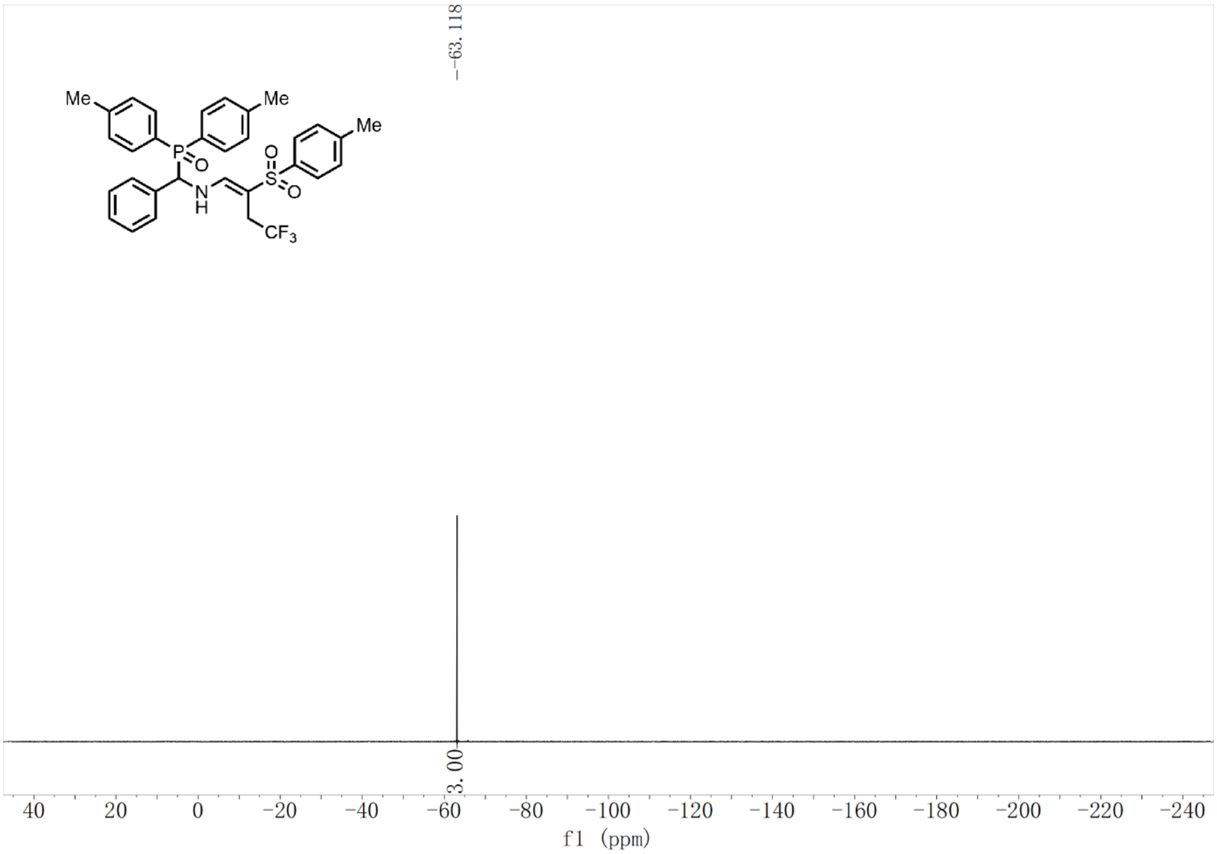

4z – ^{31}P NMR (202 MHz, DMSO)

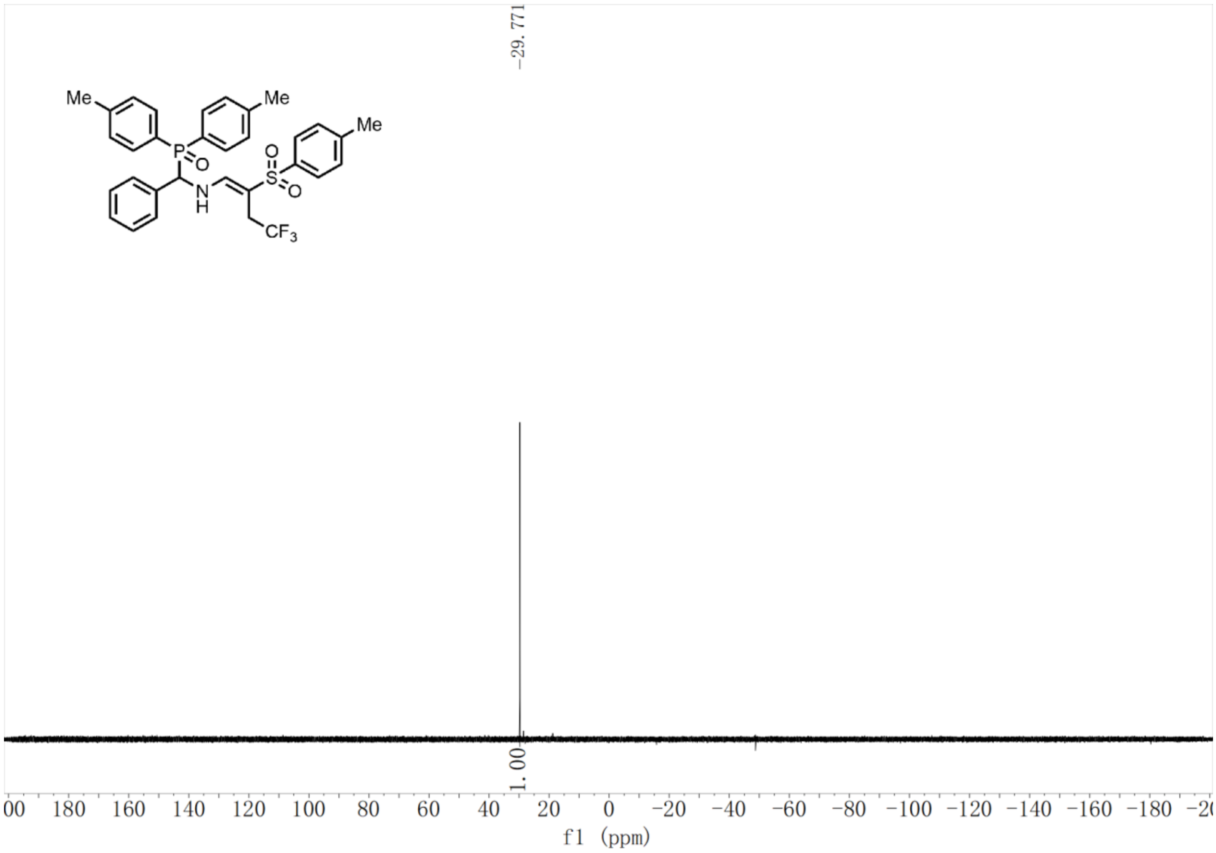

4aa – ^{1}H NMR (500 MHz, DMSO)

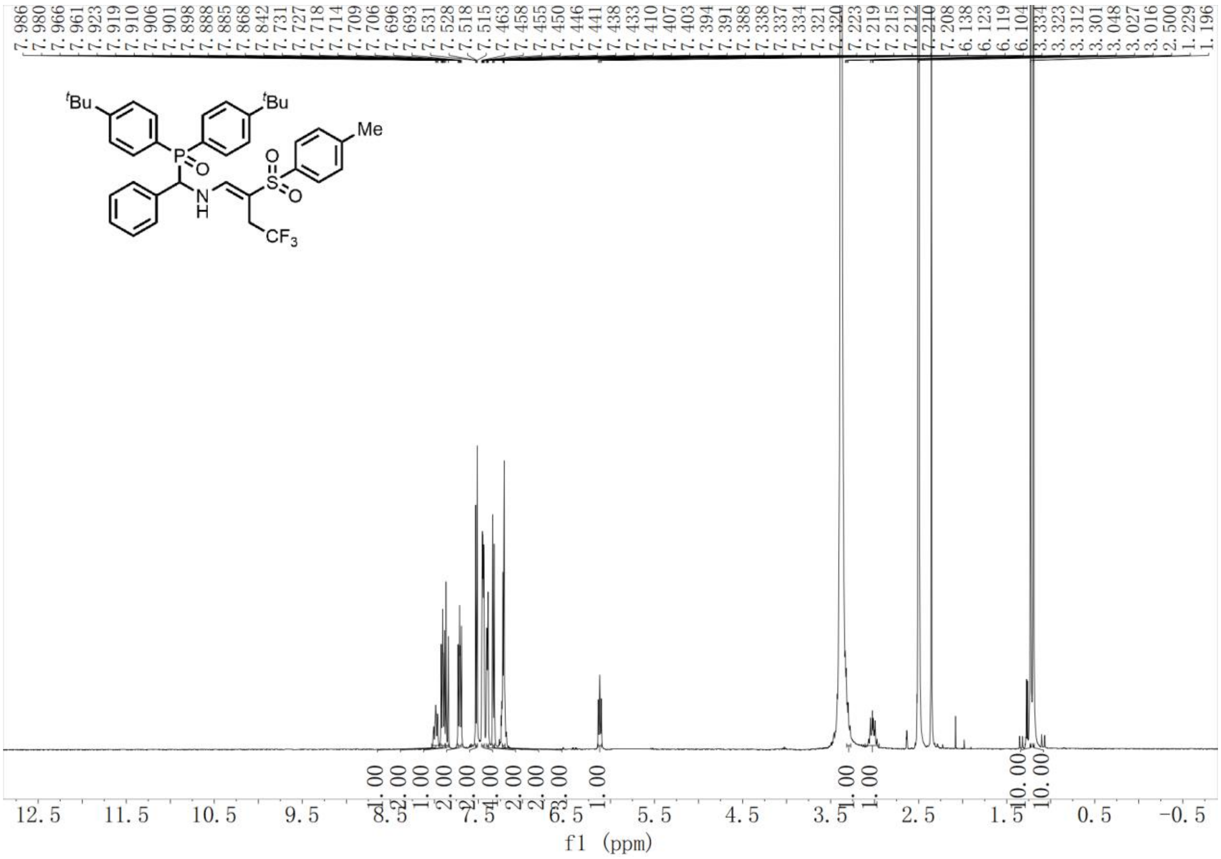

4aa – ^{13}C NMR (126 MHz, DMSO)

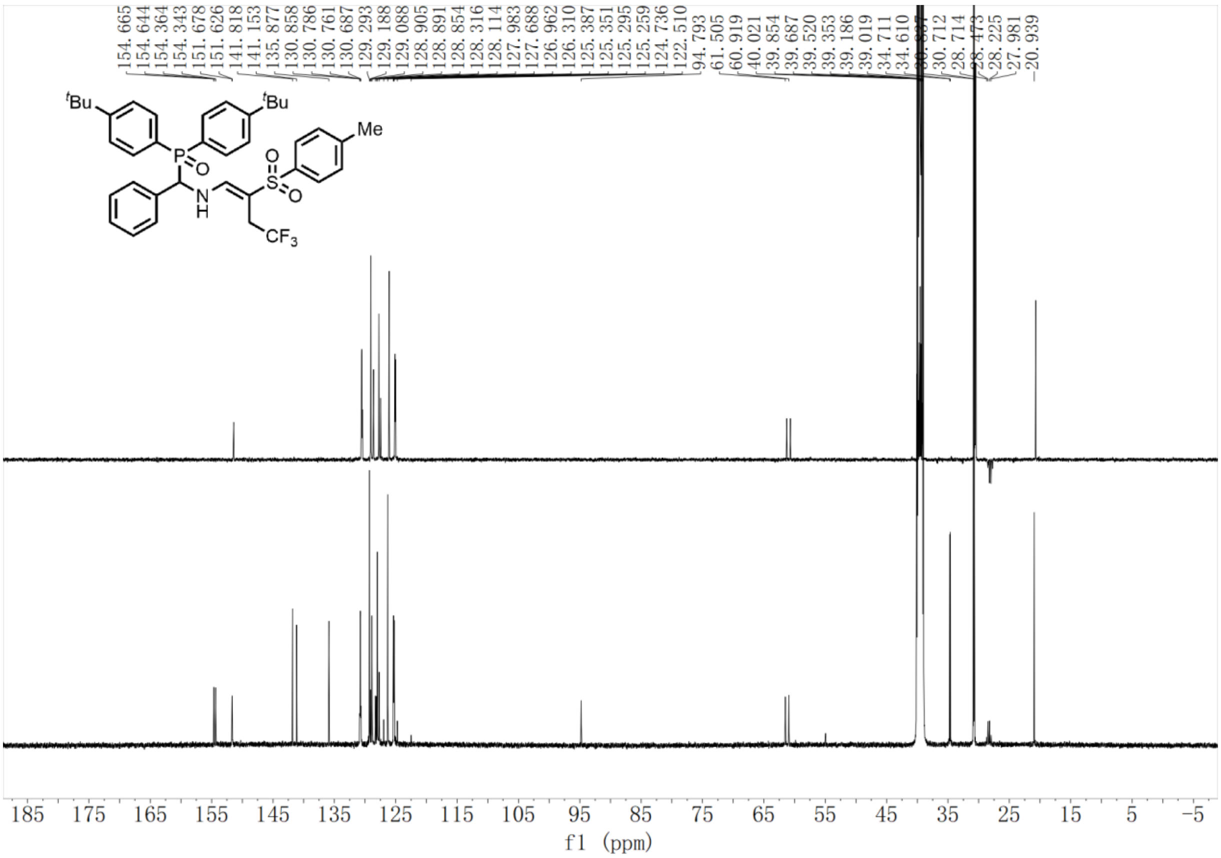

4aa – ^{19}F NMR (471 MHz, DMSO)

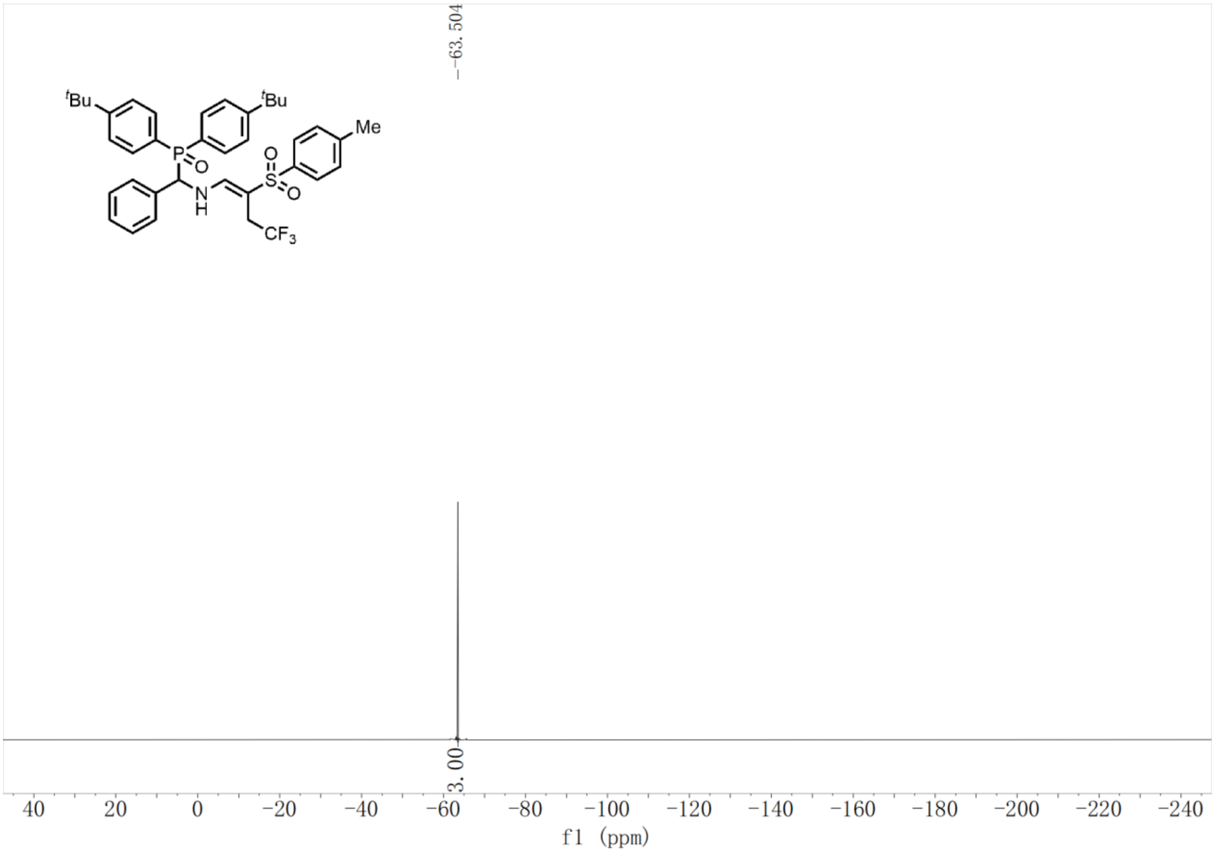

4aa – ^{31}P NMR (202 MHz, DMSO)

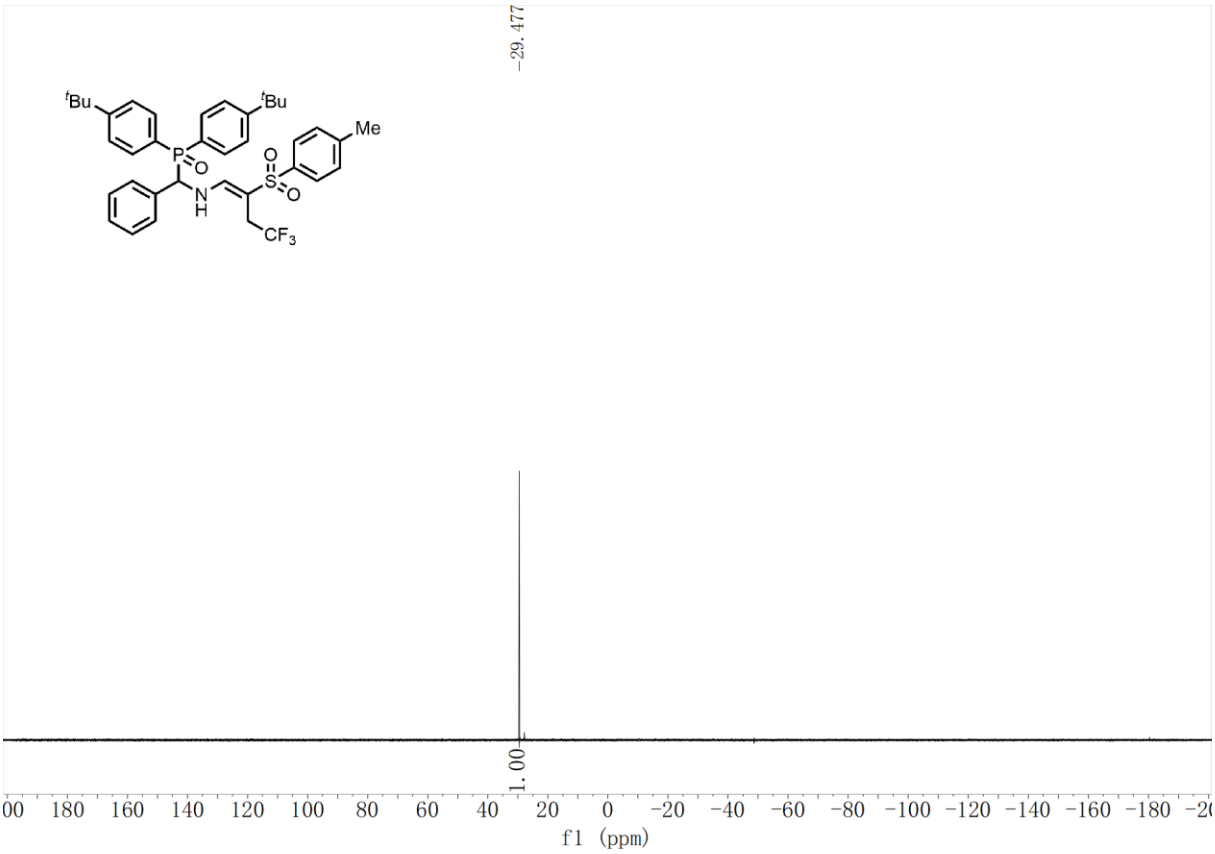

4ab – ^{1}H NMR (500 MHz, DMSO)

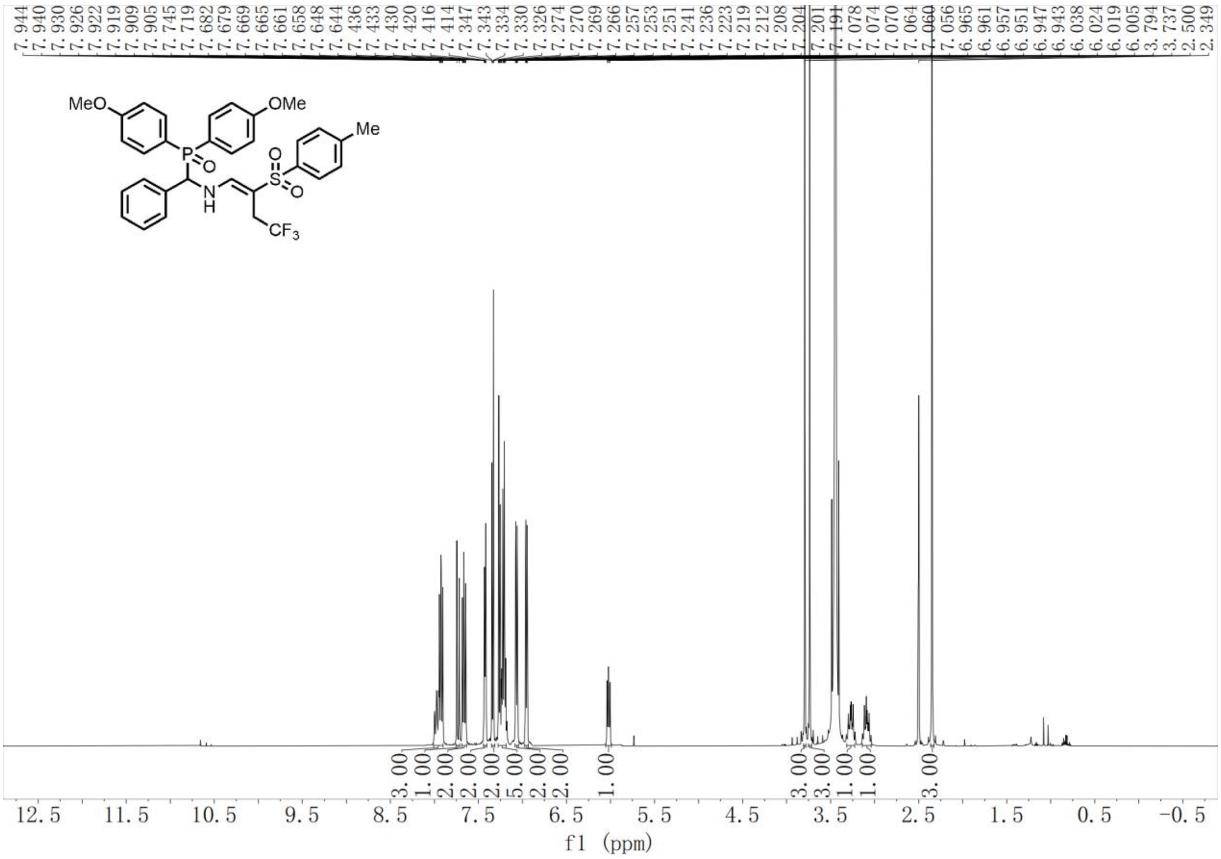

4ab – ^{13}C NMR (126 MHz, DMSO)

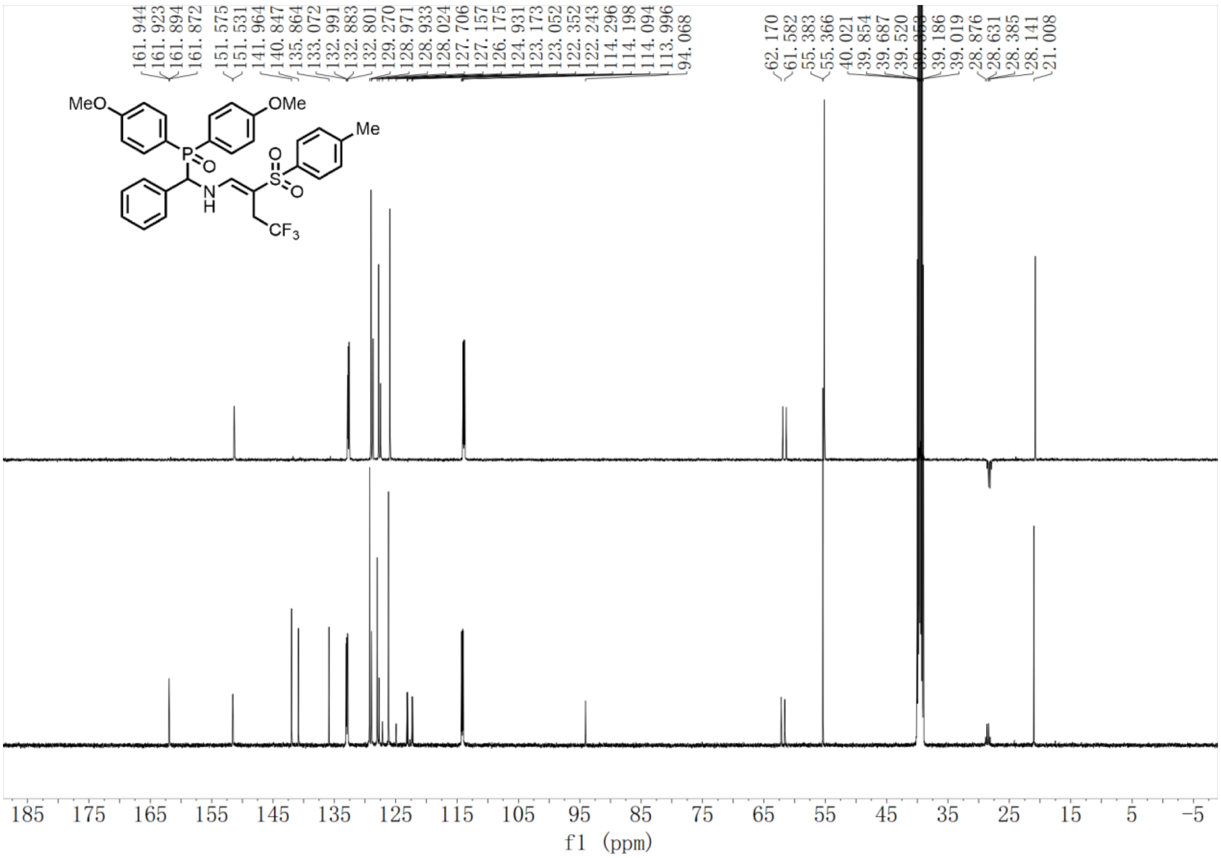

4ab – ^{19}F NMR (282 MHz, DMSO)

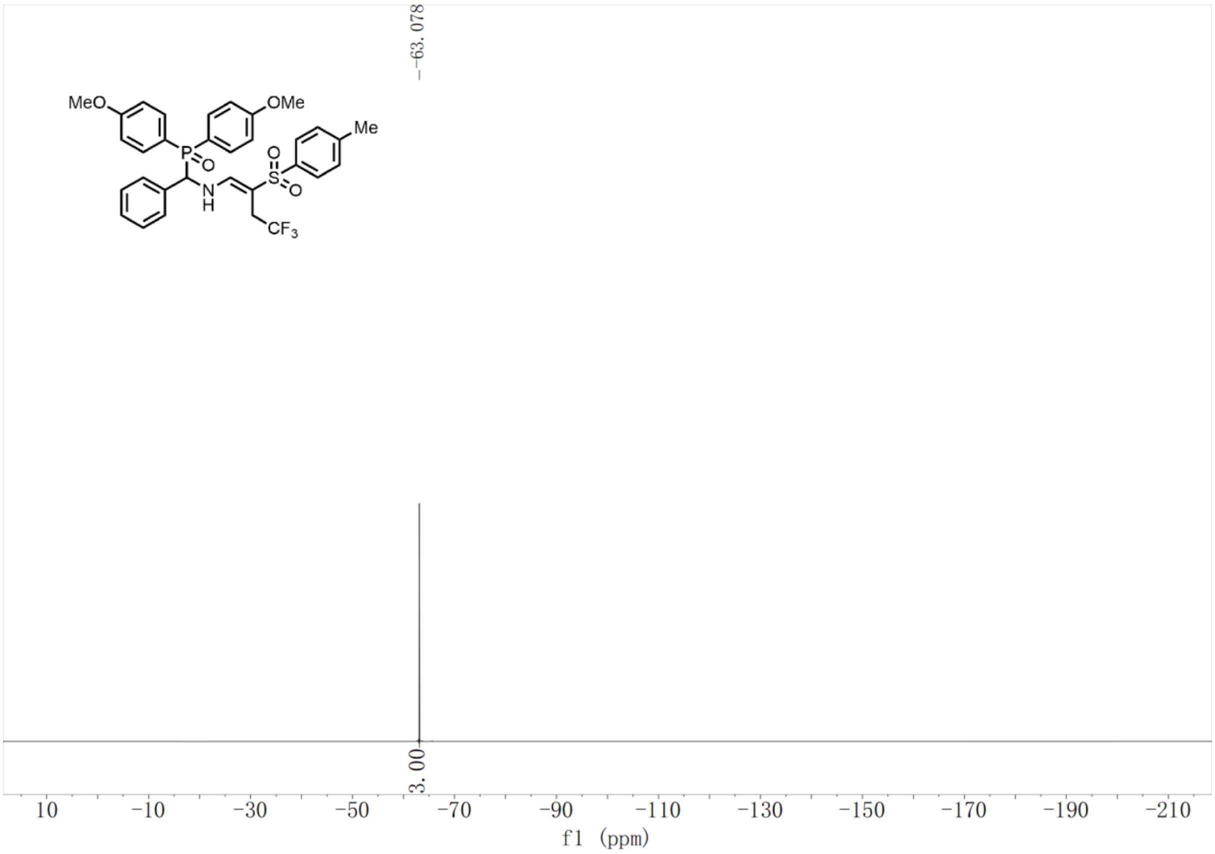

4ab – ^{31}P NMR (121 MHz, DMSO)

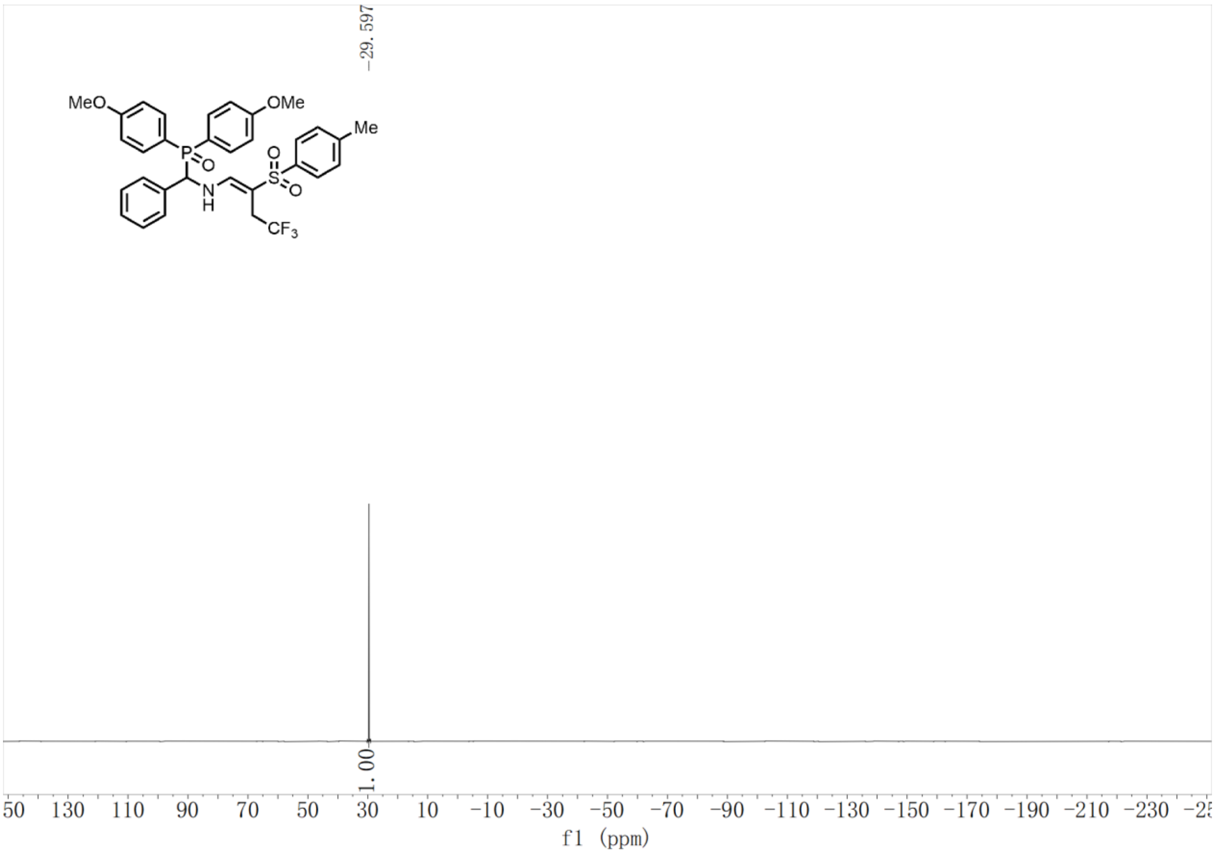

4ac – ^{1}H NMR (500 MHz, DMSO)

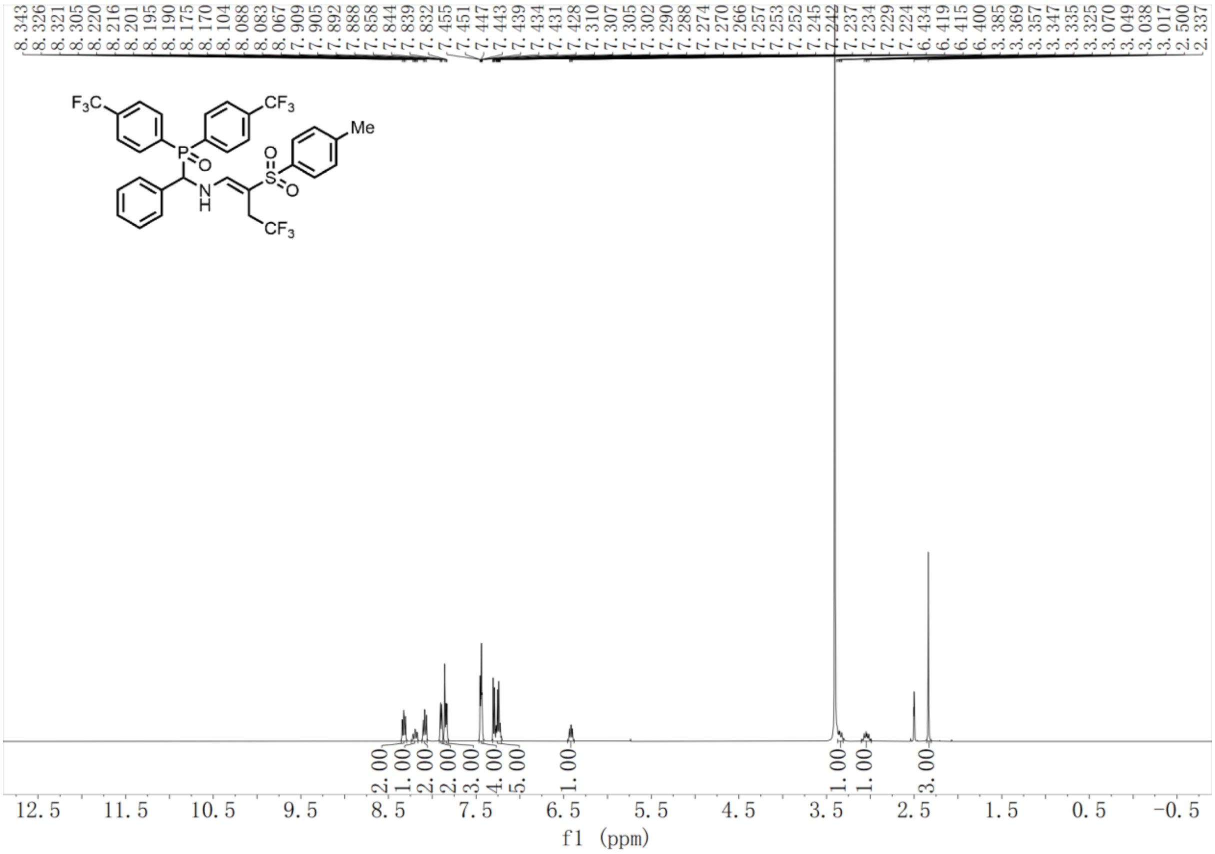

4ac – ^{13}C NMR (126 MHz, DMSO)

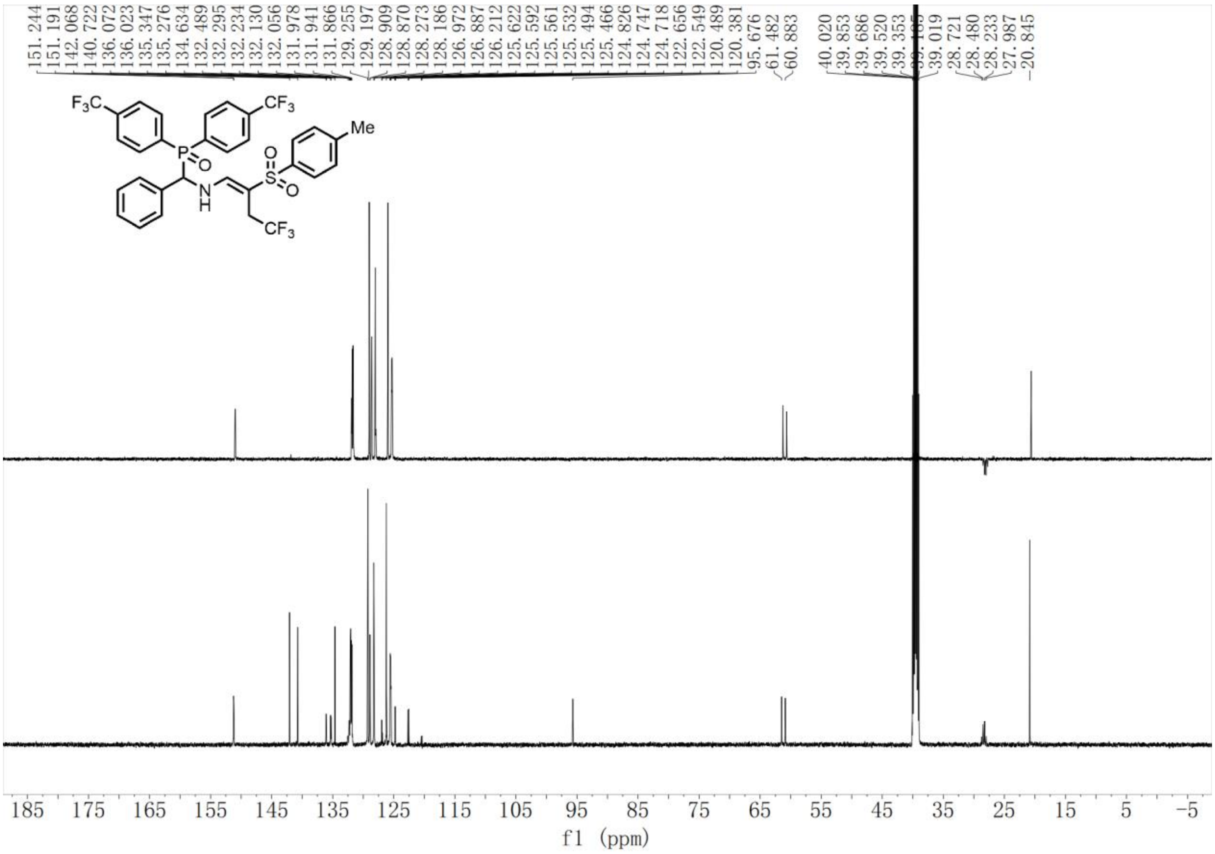

4ac – ^{19}F NMR (471 MHz, DMSO)

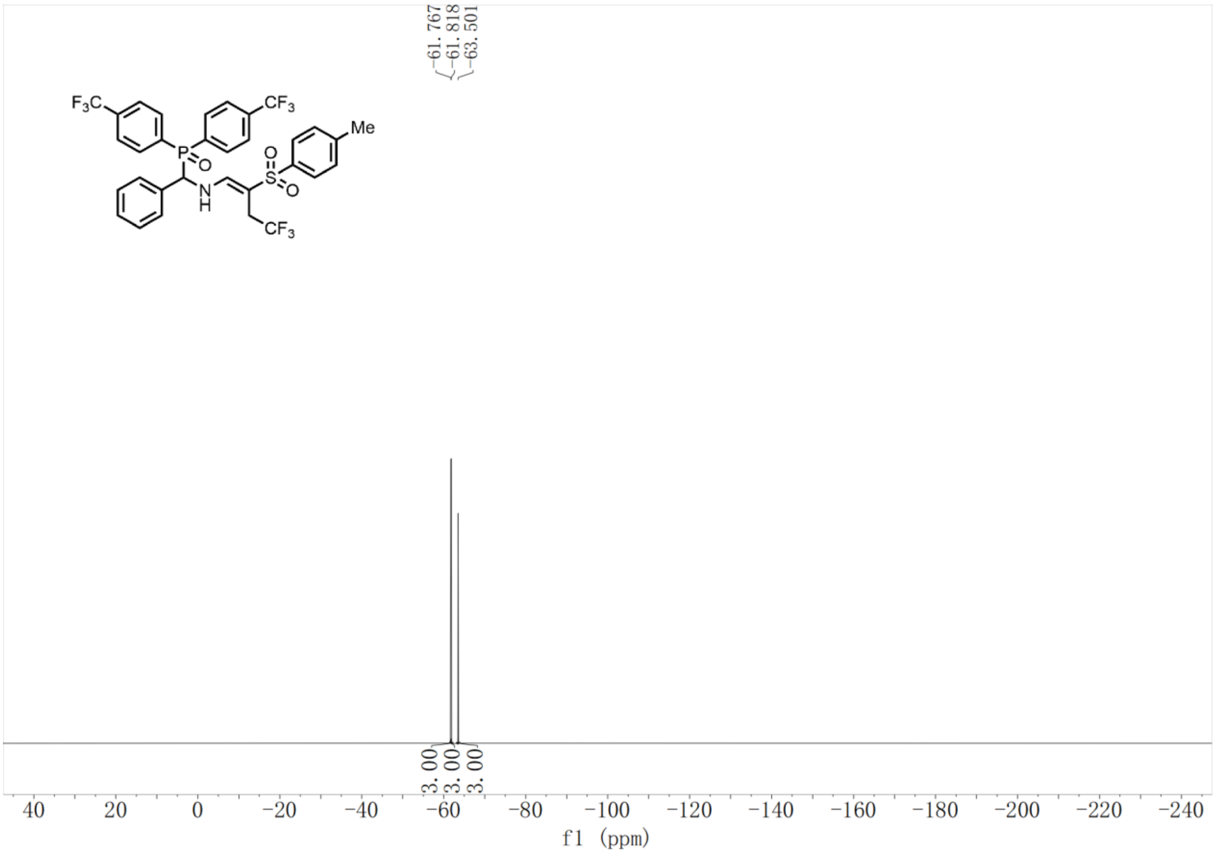

4ac – ^{31}P NMR (202 MHz, DMSO)

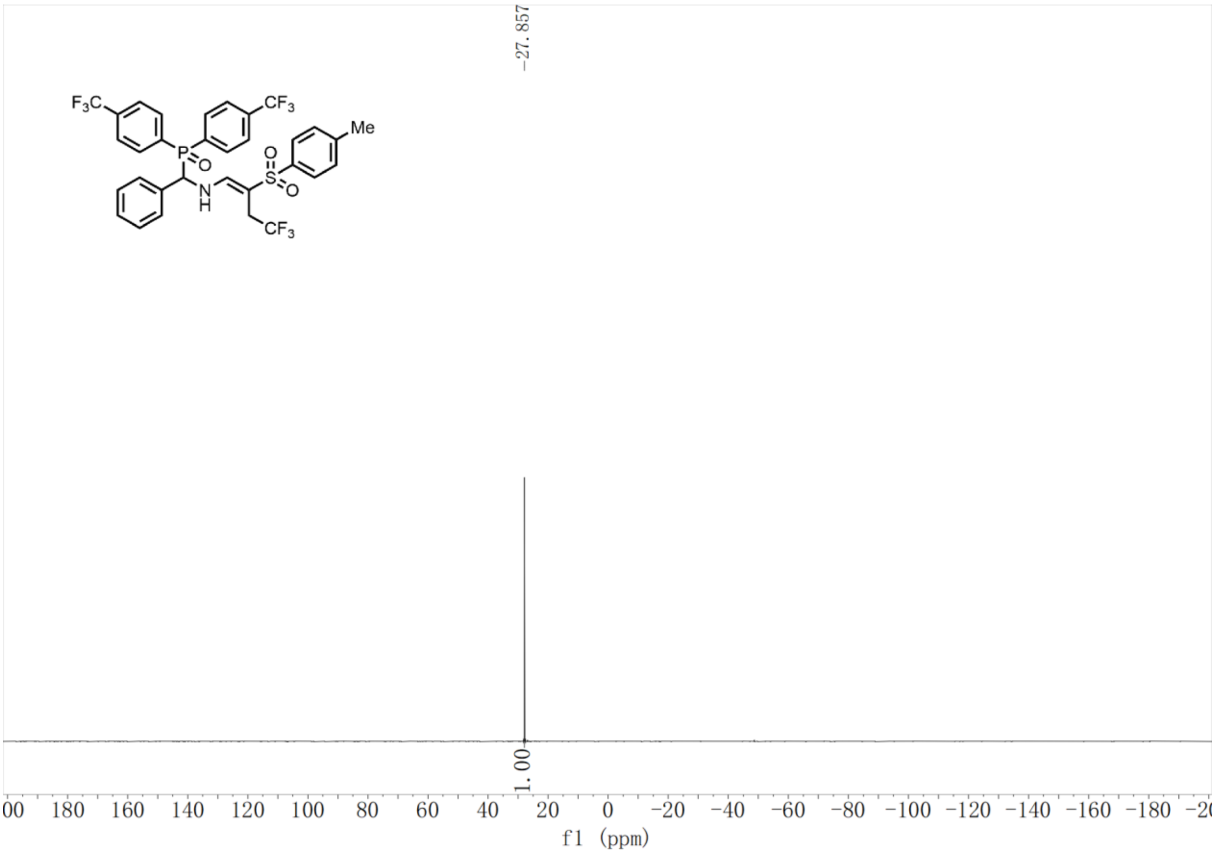

4ad – ^{1}H NMR (500 MHz, DMSO)

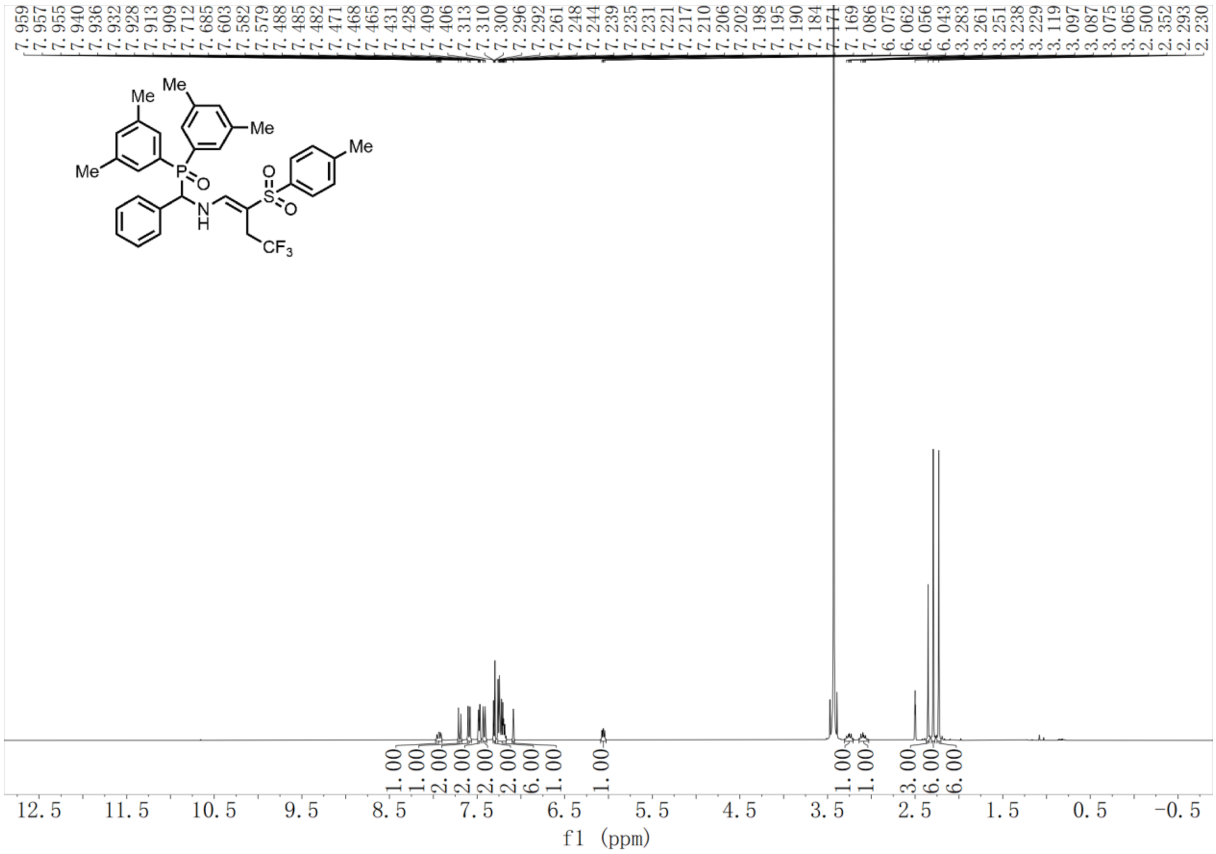

4ad – ^{13}C NMR (126 MHz, DMSO)

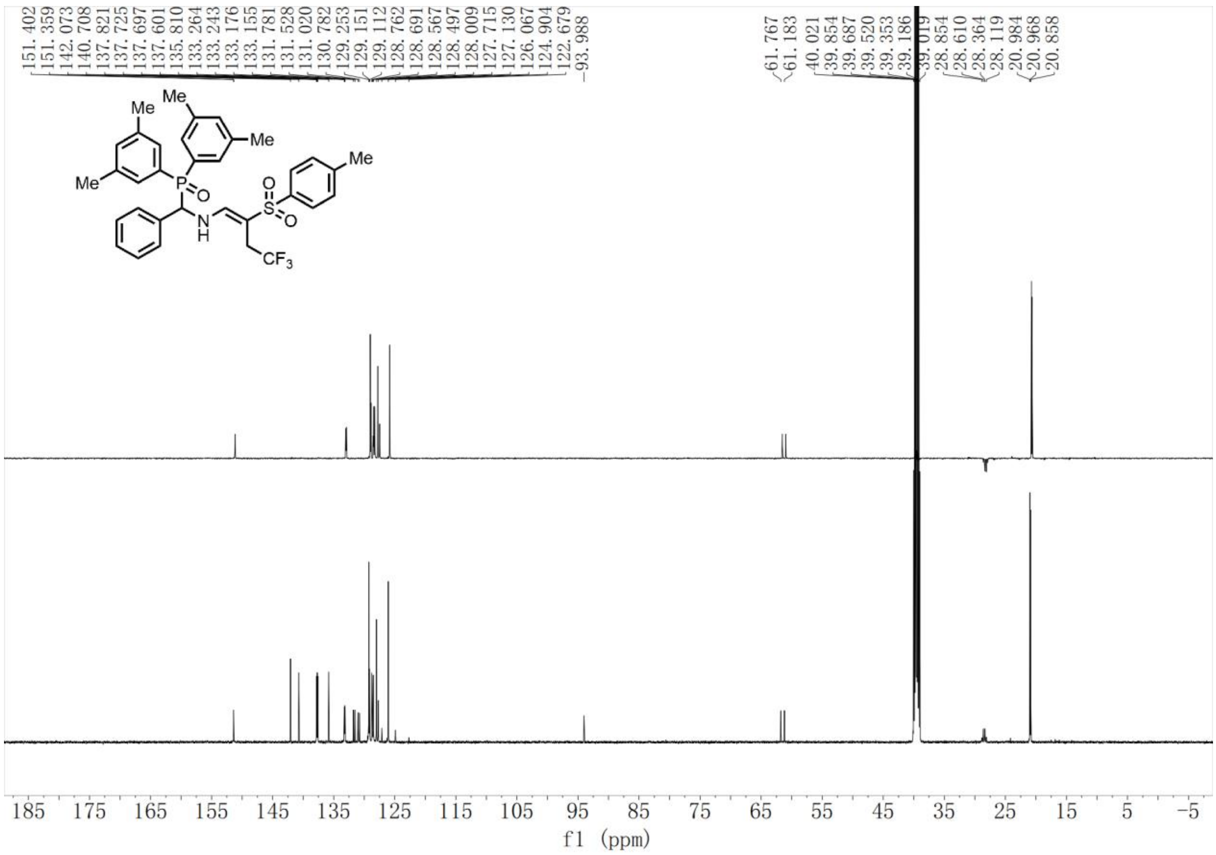

4ad – ^{19}F NMR (282 MHz, DMSO)

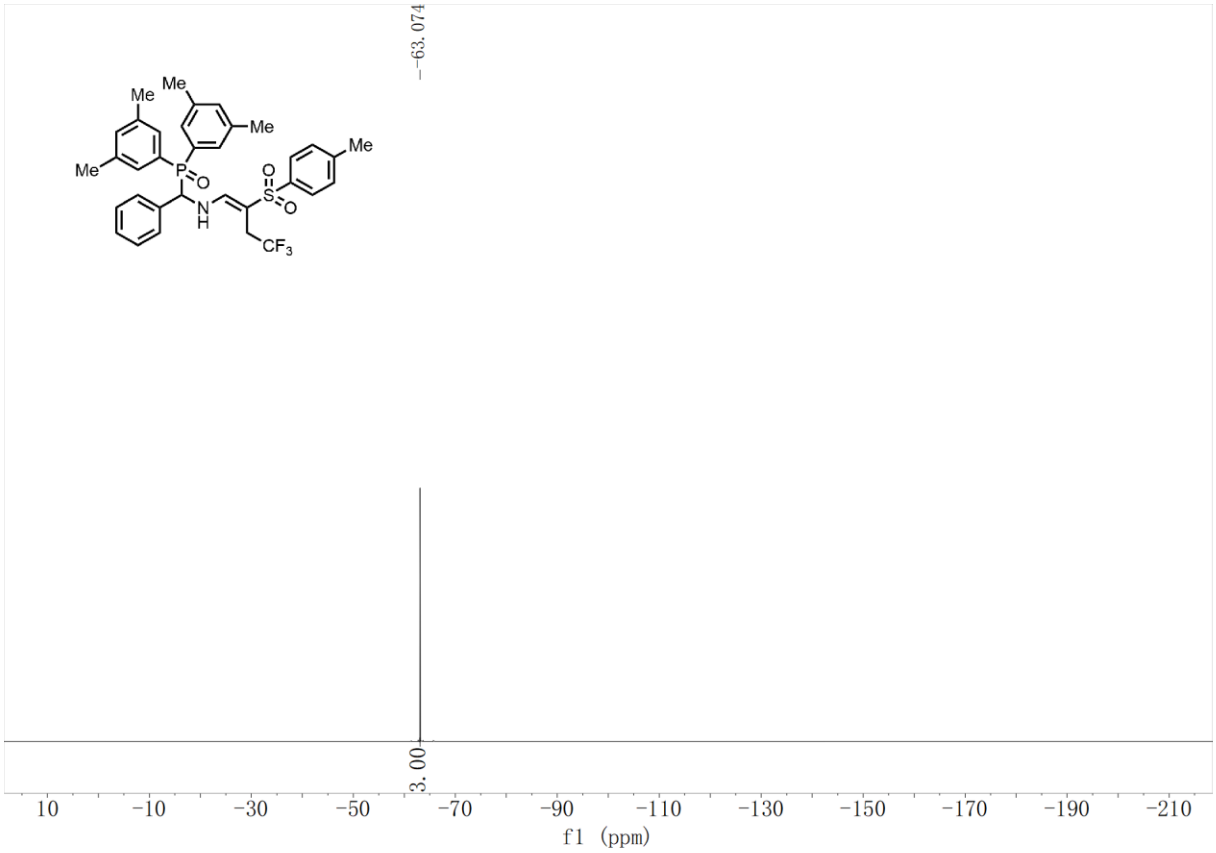

4ad – ^{31}P NMR (121 MHz, DMSO)

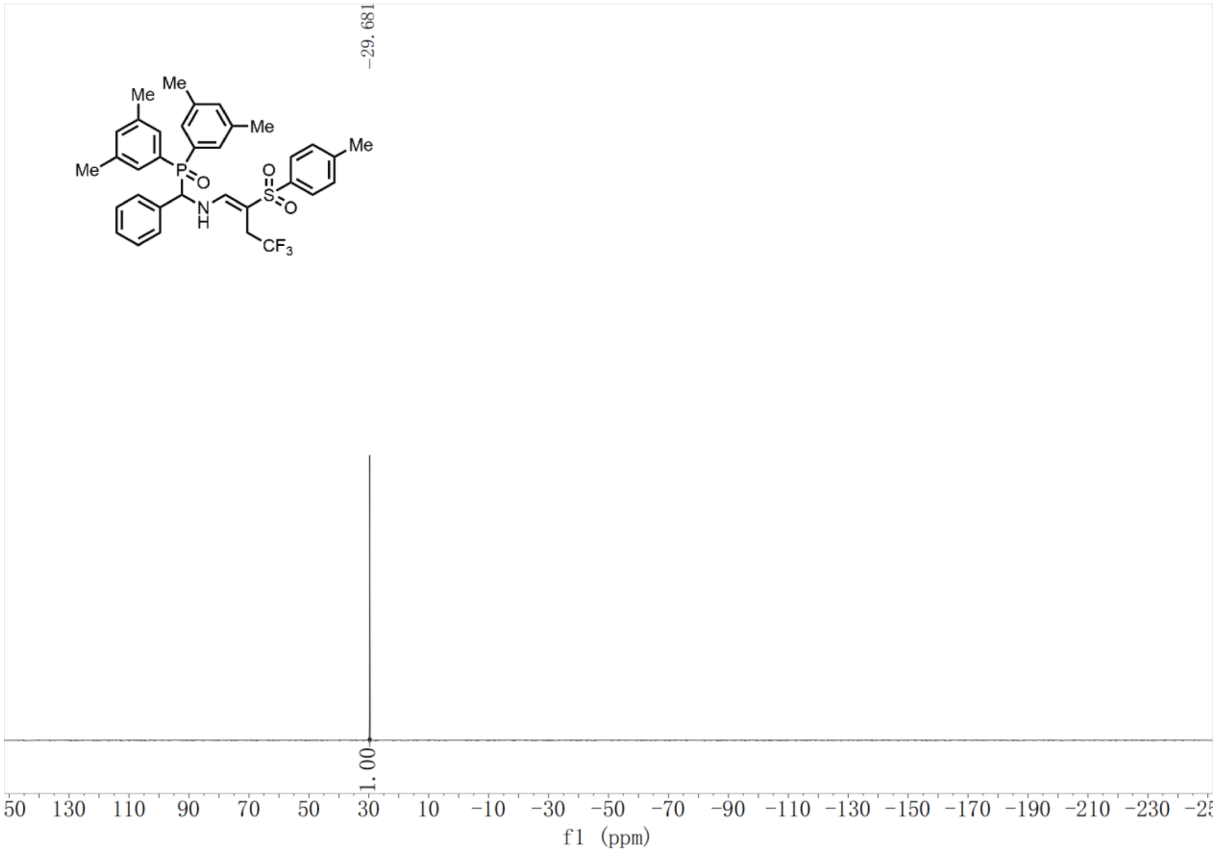

4ae – ^{1}H NMR (500 MHz, DMSO)

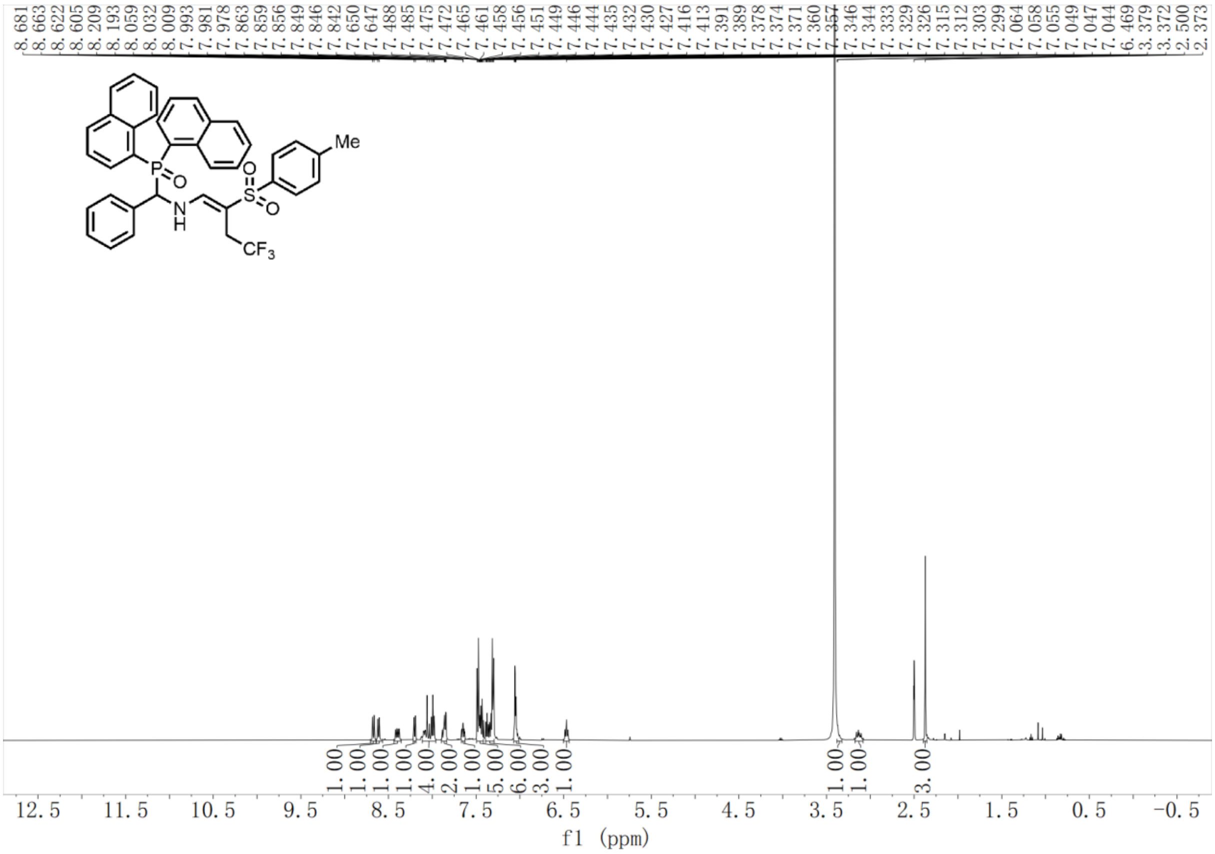

4ae – ^{13}C NMR (126 MHz, DMSO)

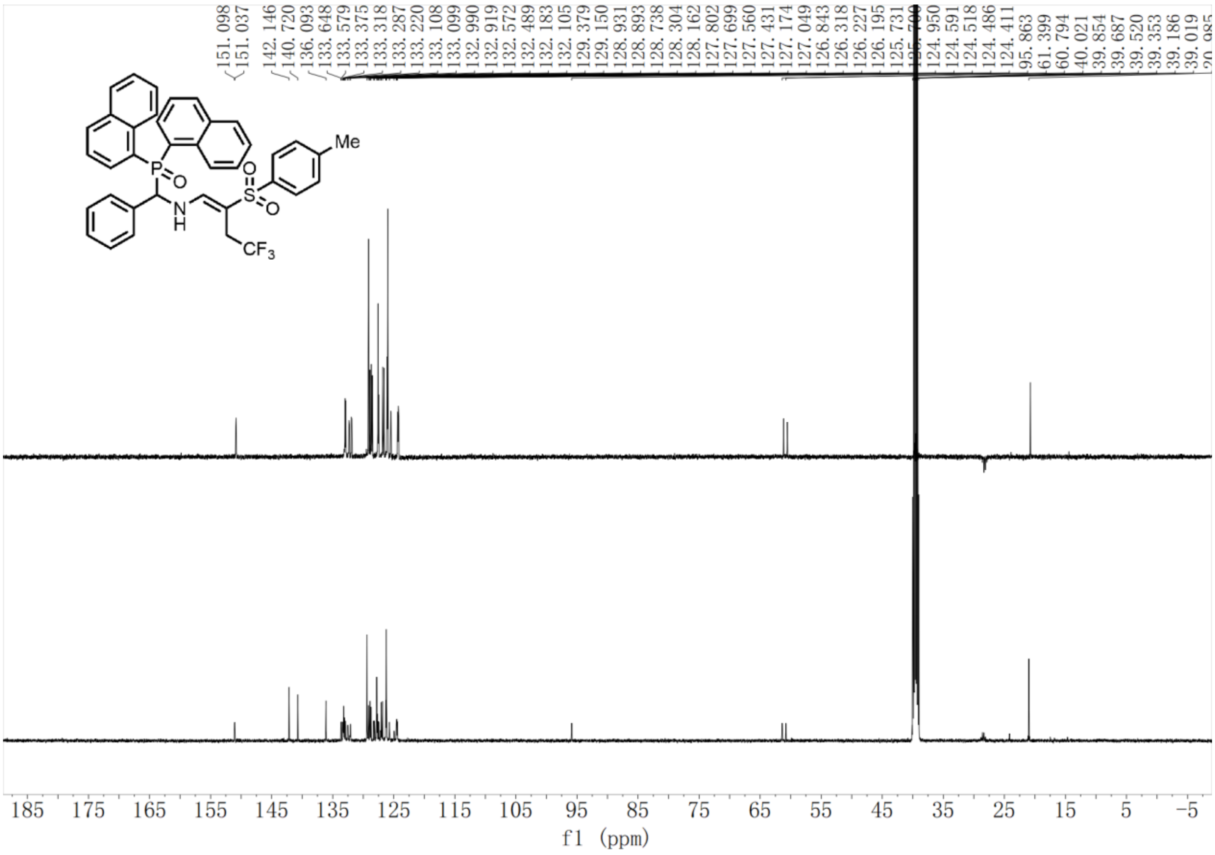

4ae – ^{19}F NMR (471 MHz, DMSO)

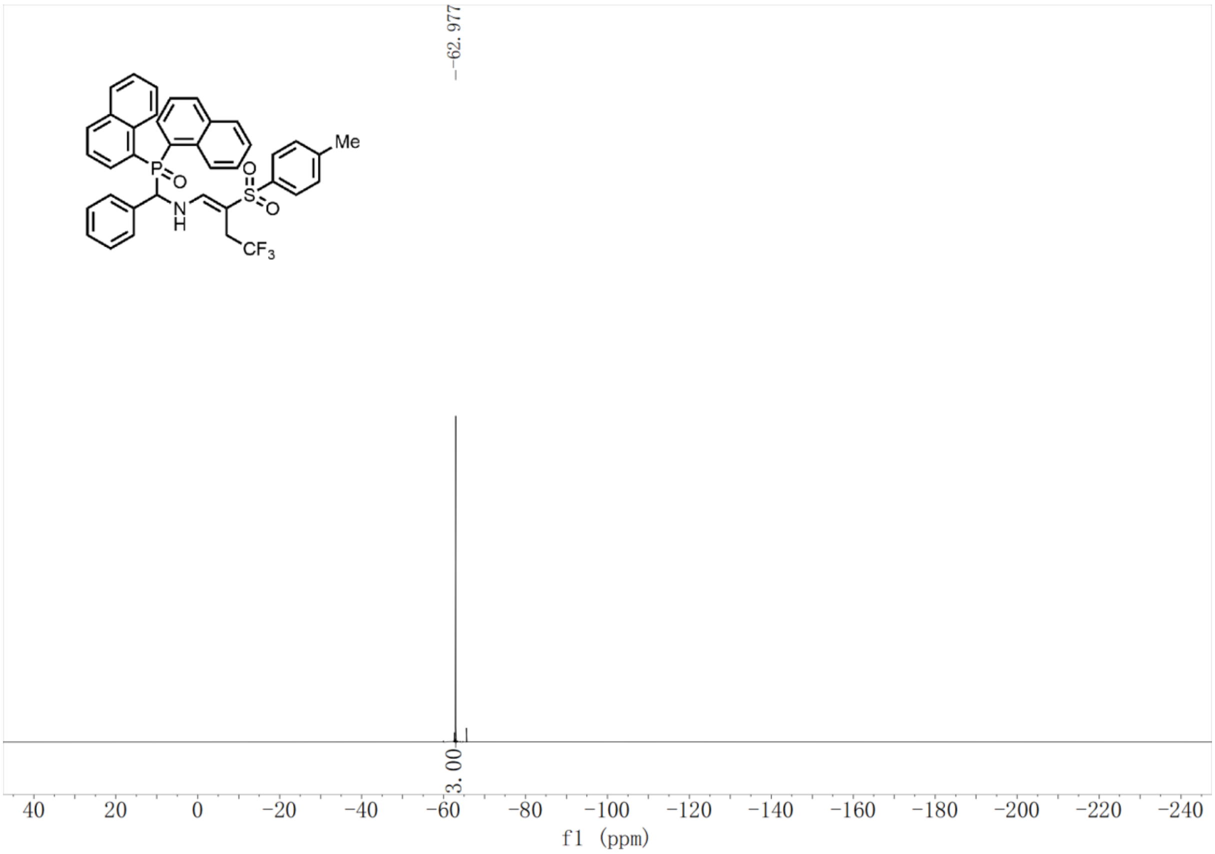

4ae – ^{31}P NMR (202 MHz, DMSO)

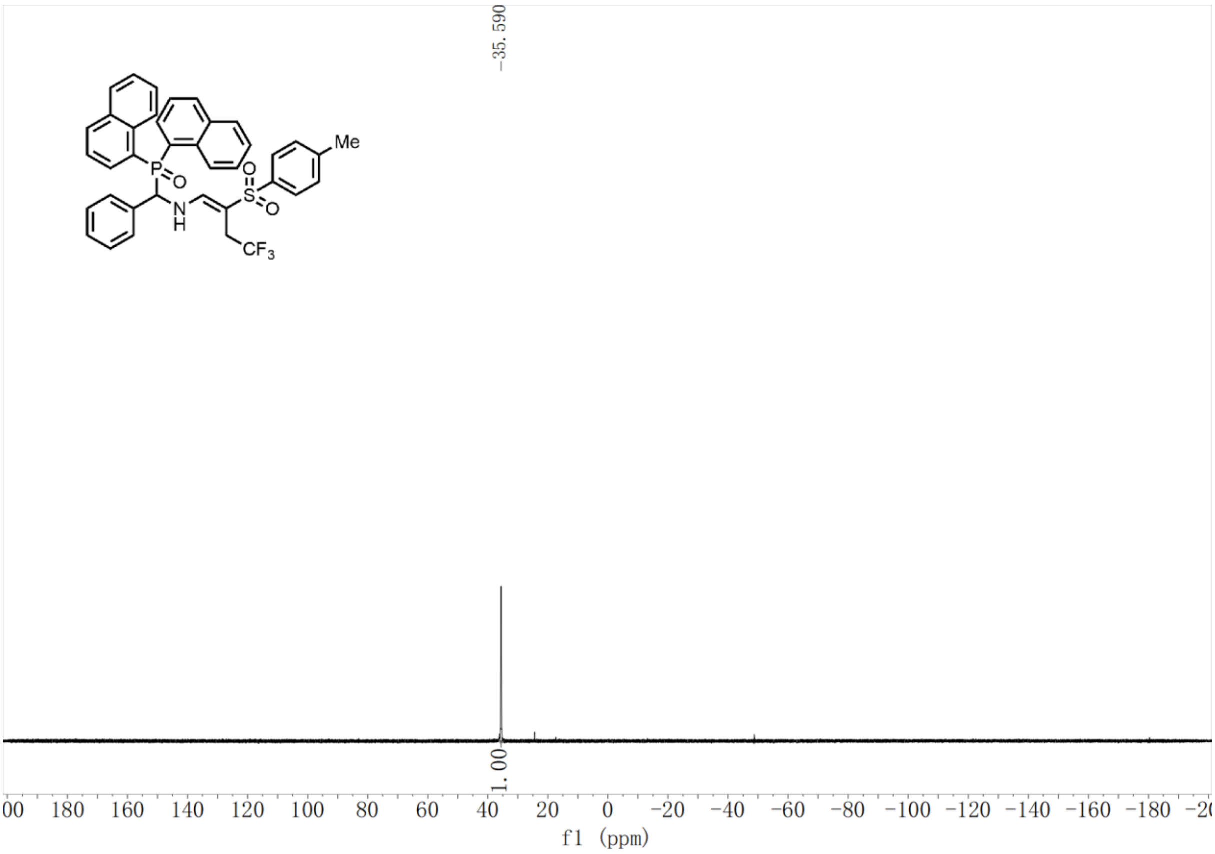

5 – ^{1}H NMR (500 MHz, C_6D_6)

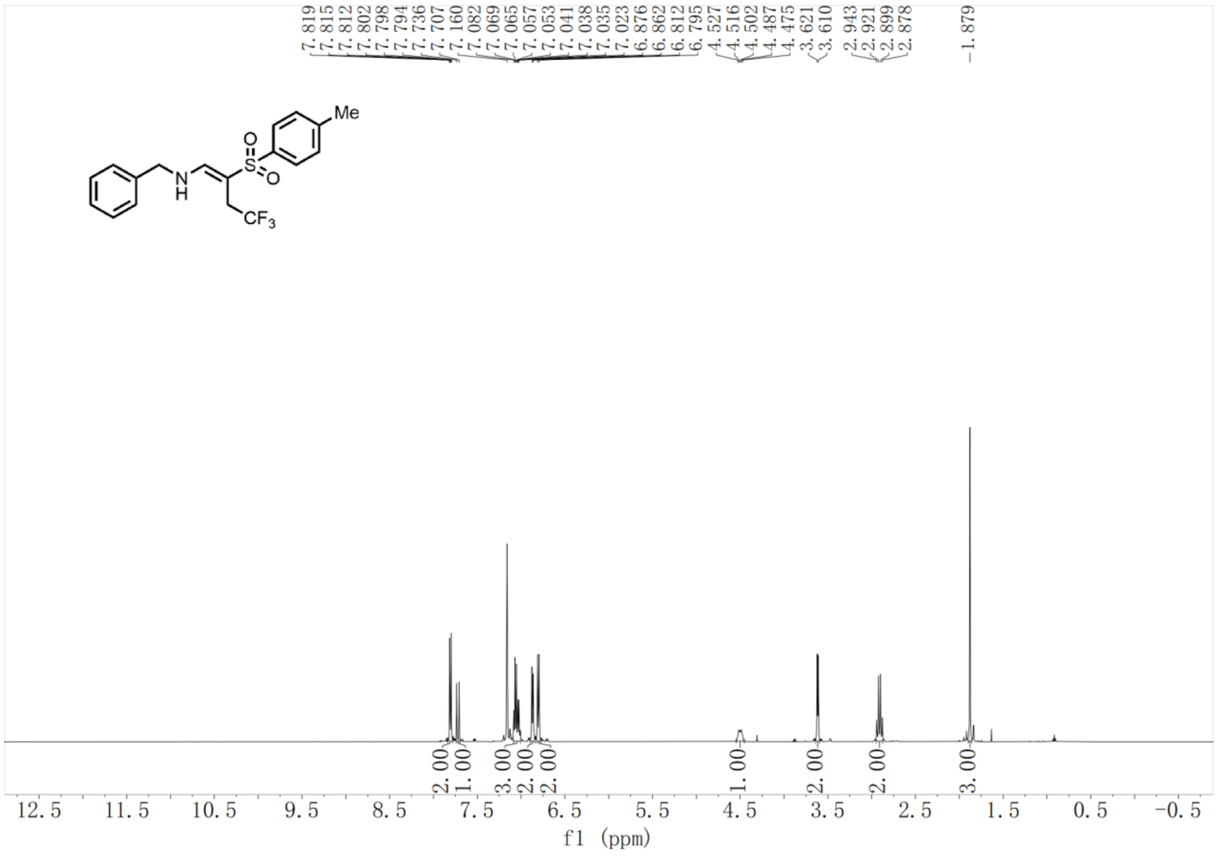

5 – ^{13}C NMR (126 MHz, C_6D_6)

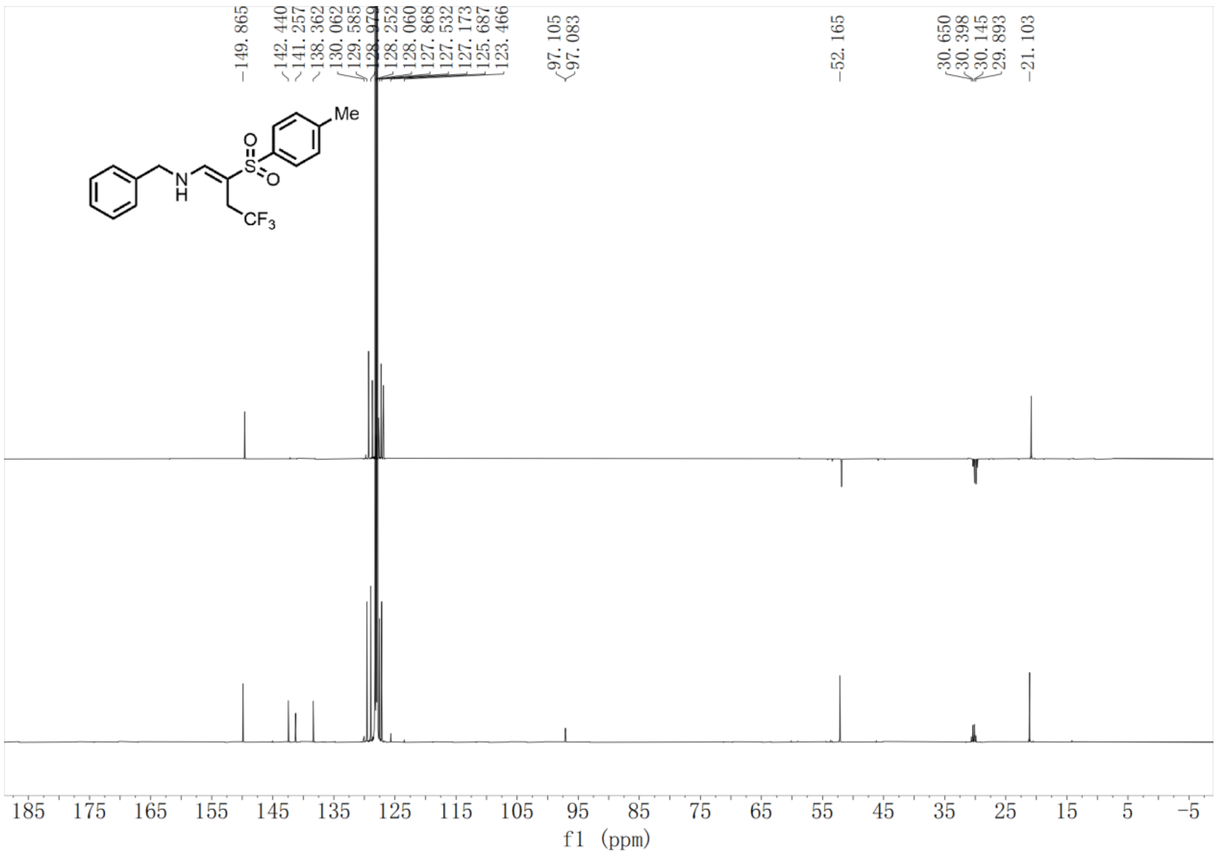

5 – ^{19}F NMR (282 MHz, C_6D_6)

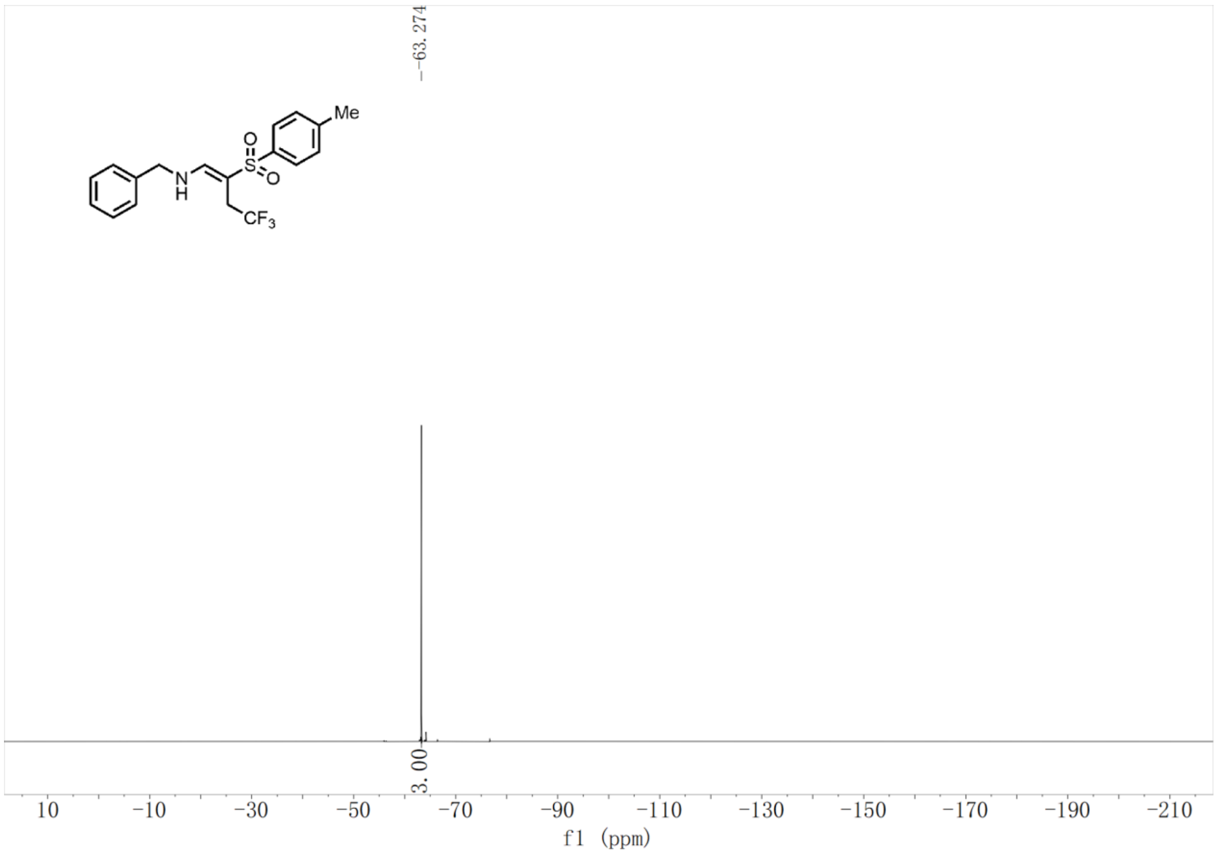

6 – ^{1}H NMR (500 MHz, DMSO)

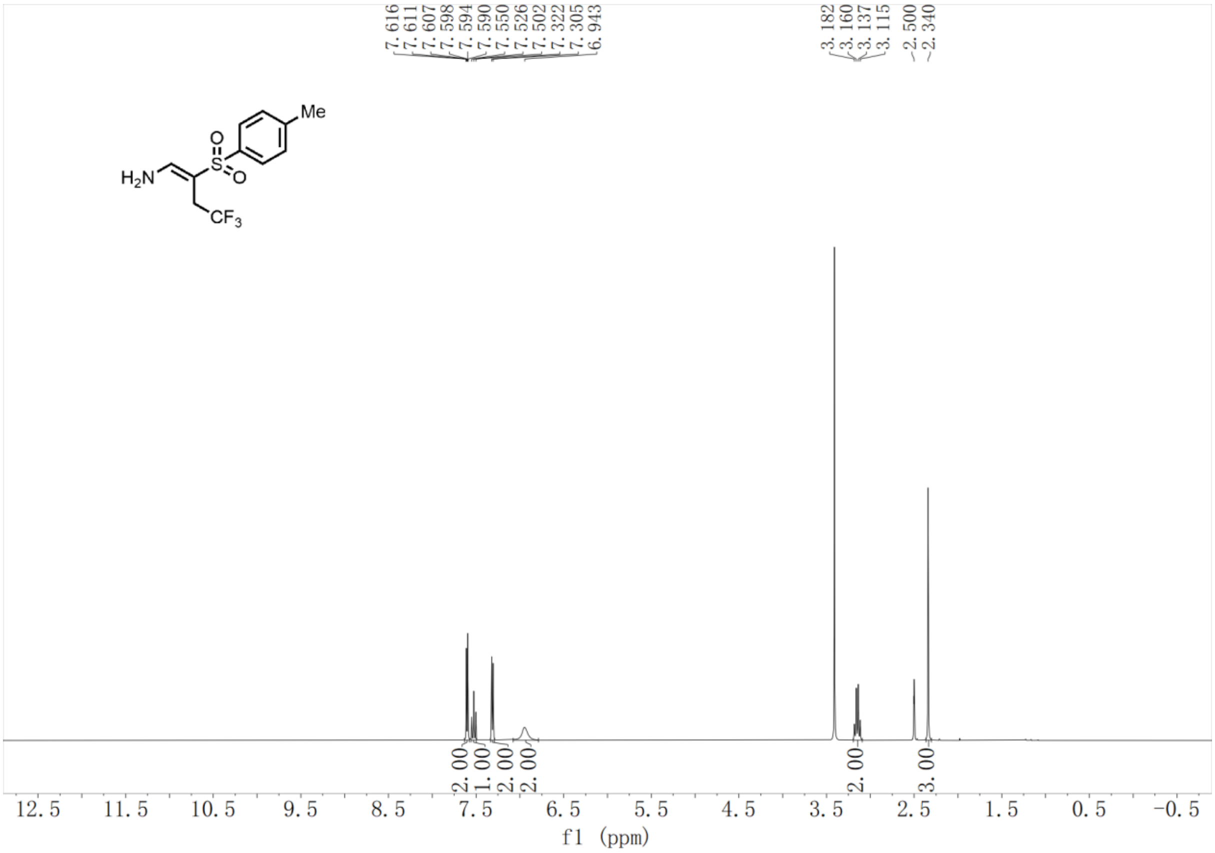

6 – ^{13}C NMR (126 MHz, DMSO)

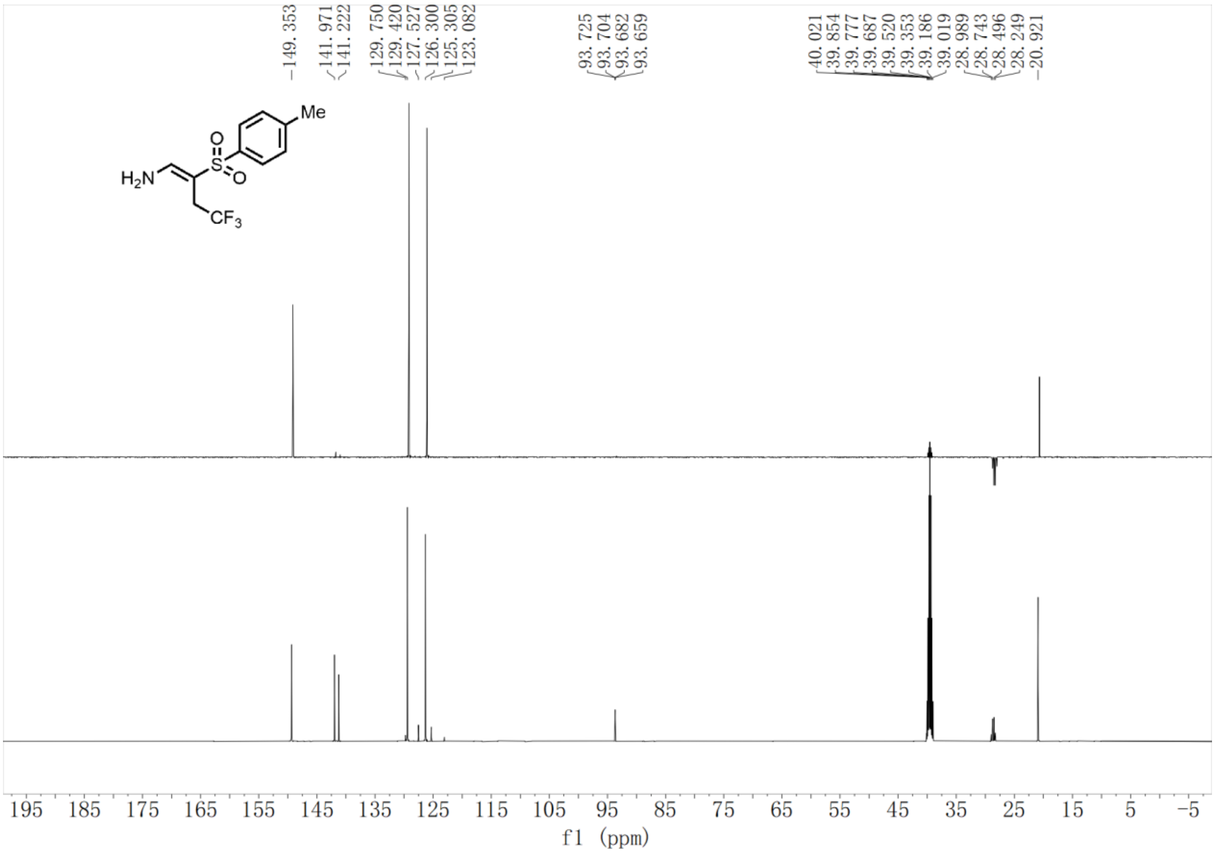

6 – ^{19}F NMR (471 MHz, DMSO)

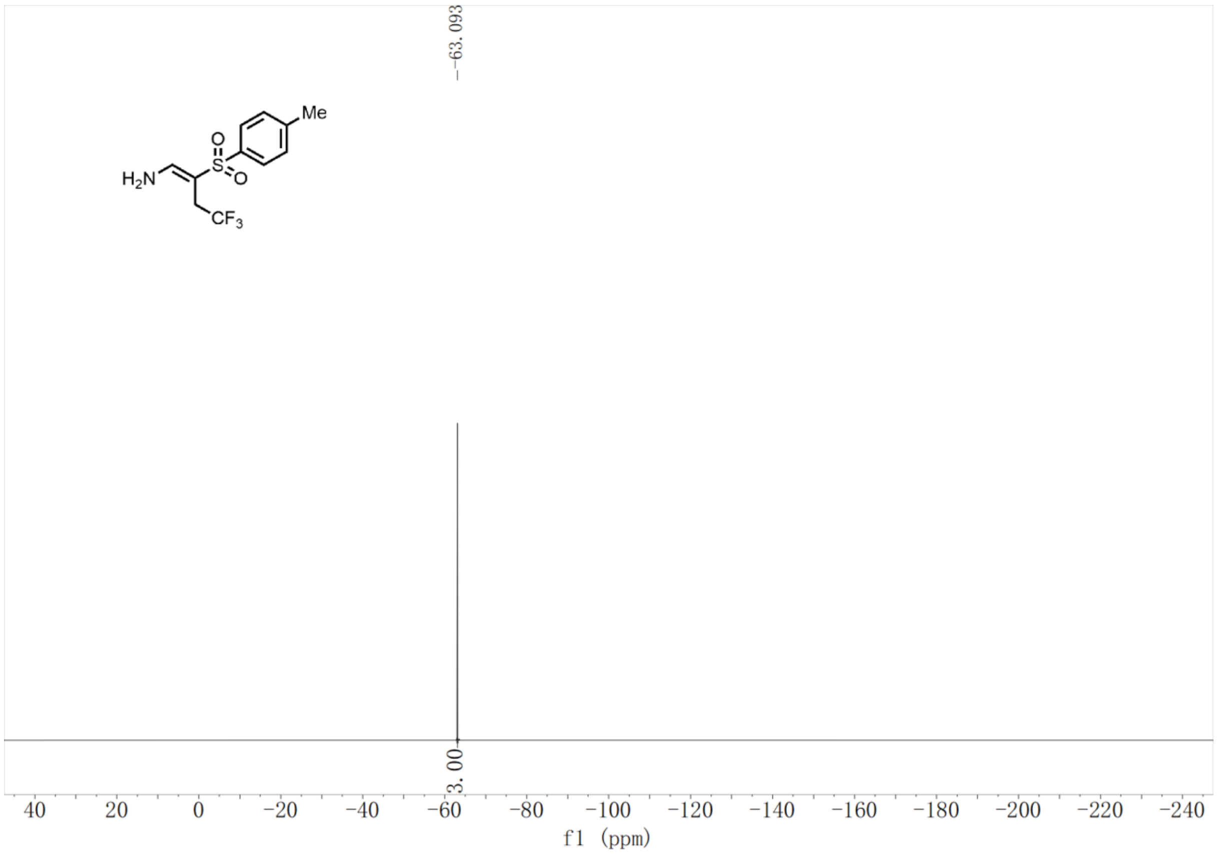

7f – ^{1}H NMR (500 MHz, $CDCl_3$)

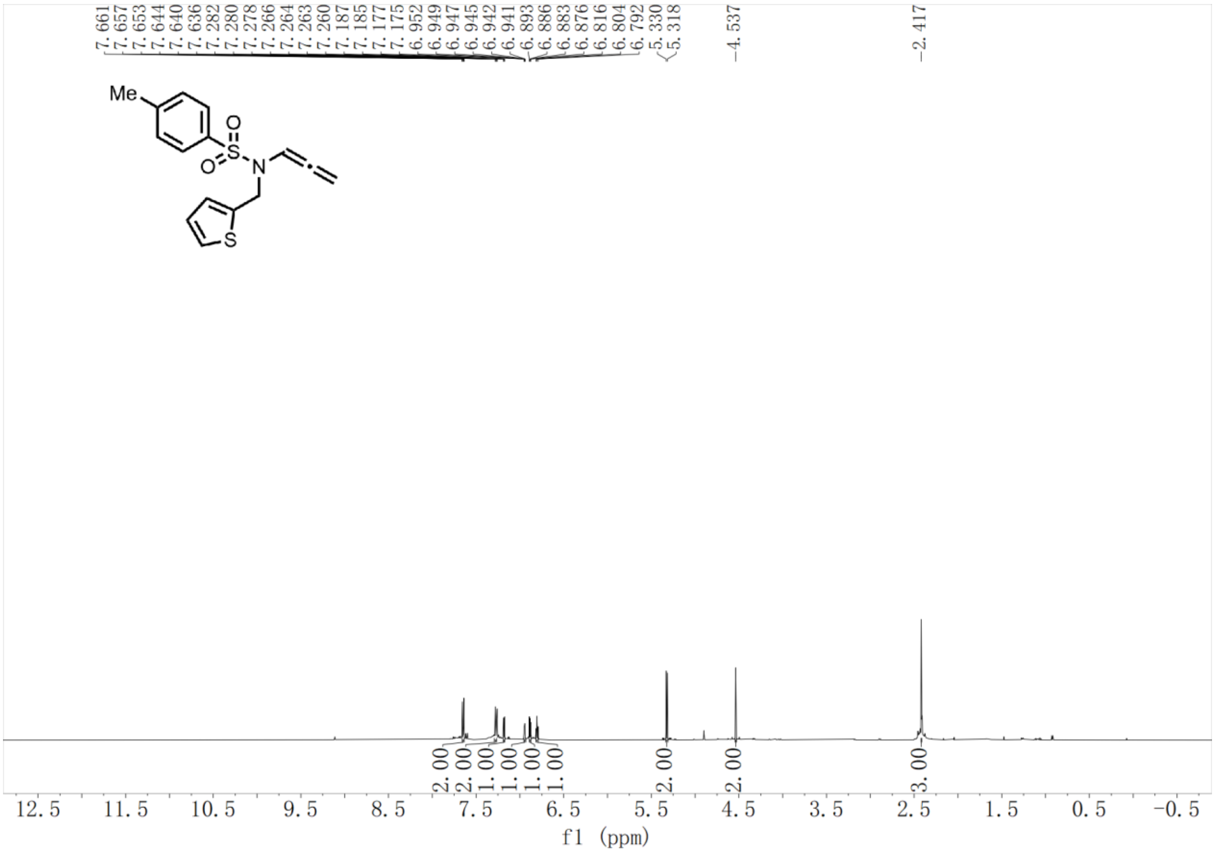

7f – ^{13}C NMR (126 MHz, $CDCl_3$)

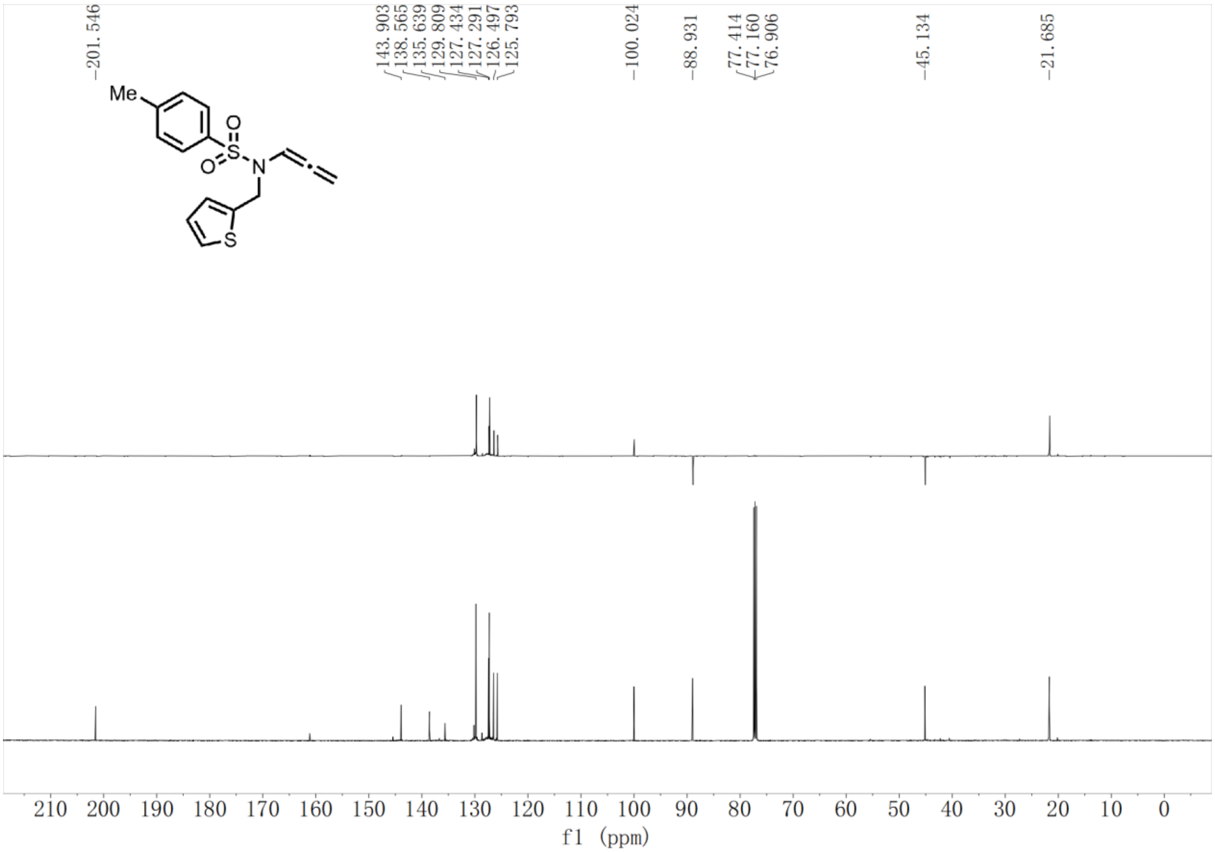

7i – ^{1}H NMR (500 MHz, CDCl$_3$)

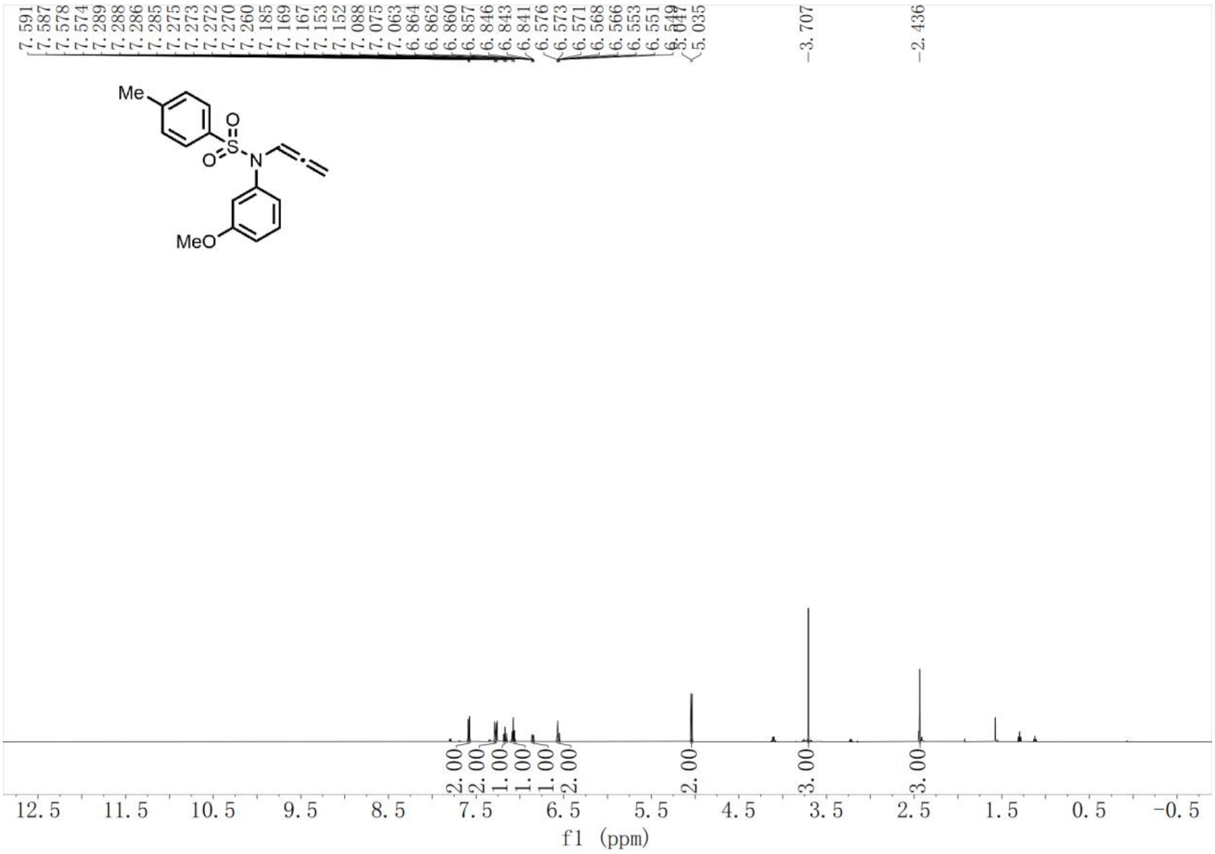

7i – ^{13}C NMR (126 MHz, CDCl$_3$)

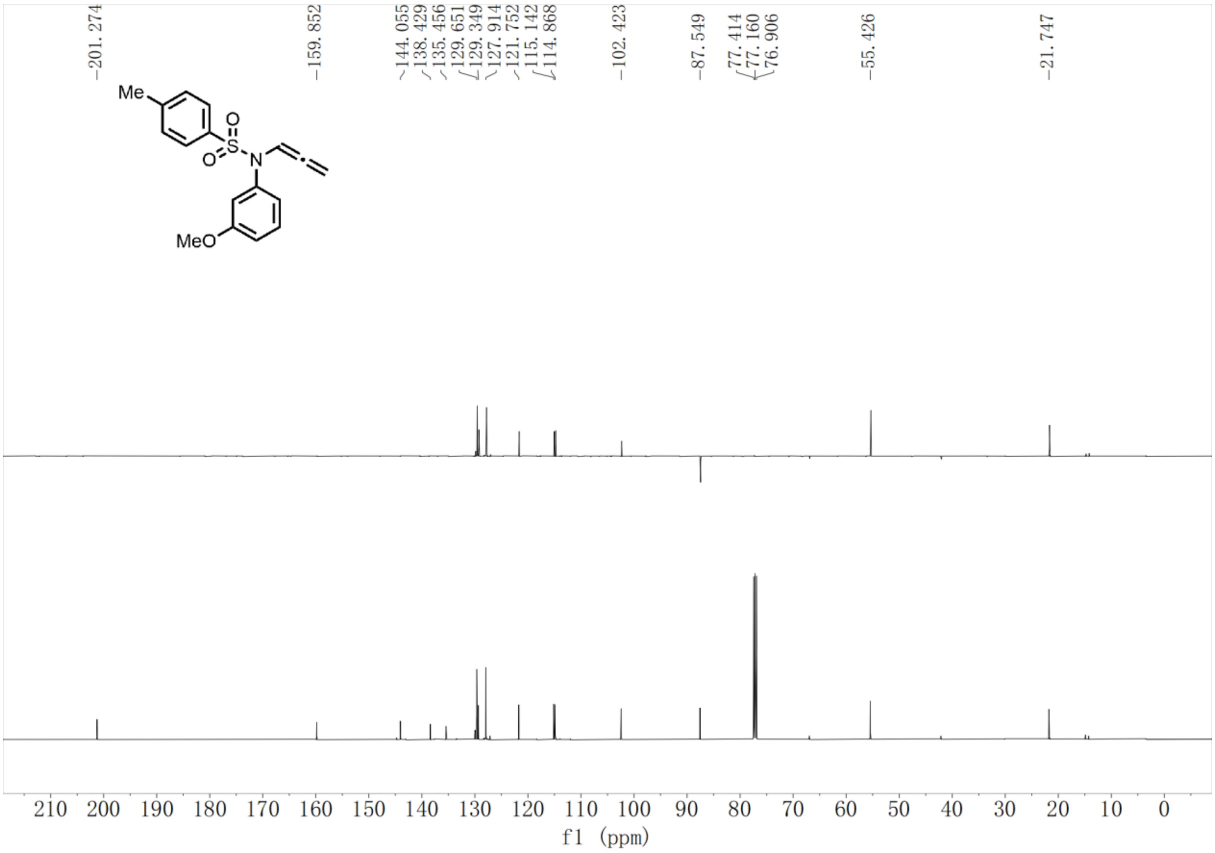

7j – ^{1}H NMR (500 MHz, $CDCl_3$)

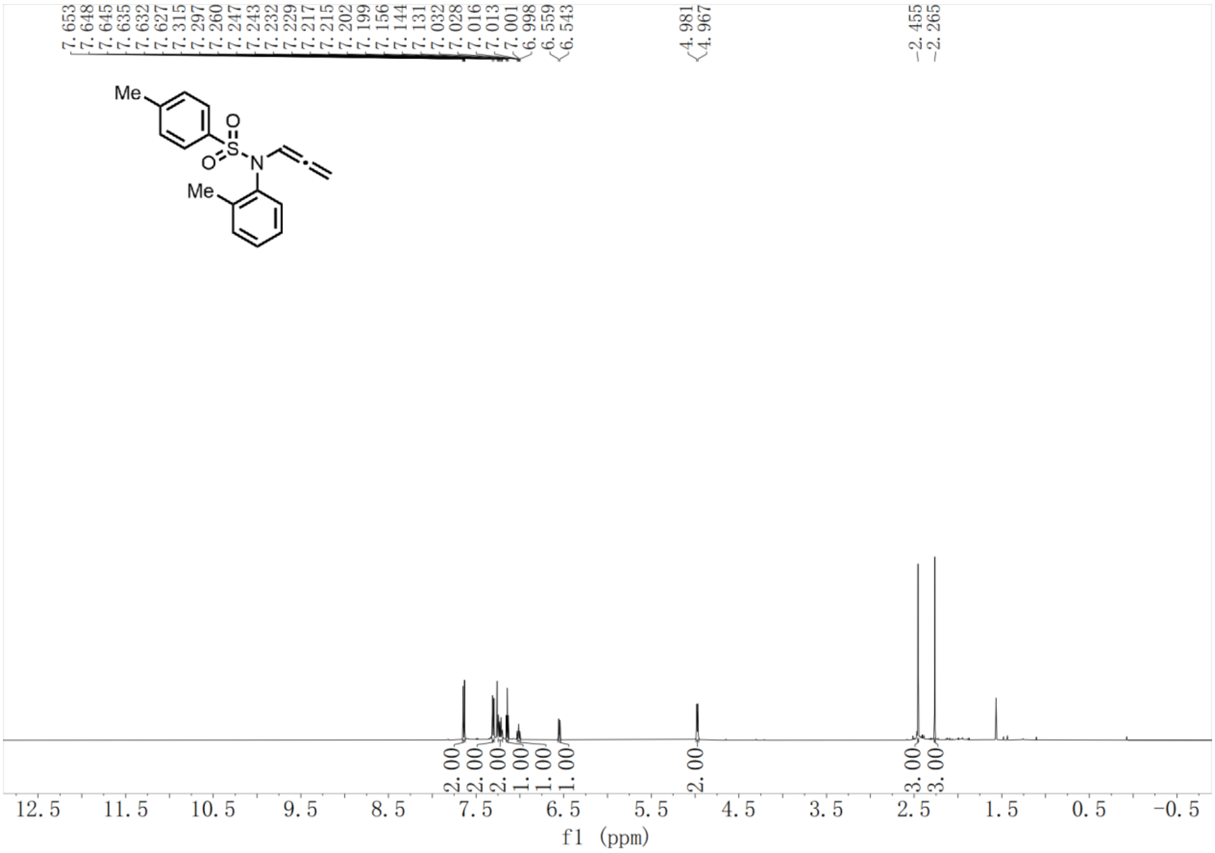

7j – ^{13}C NMR (126 MHz, $CDCl_3$)

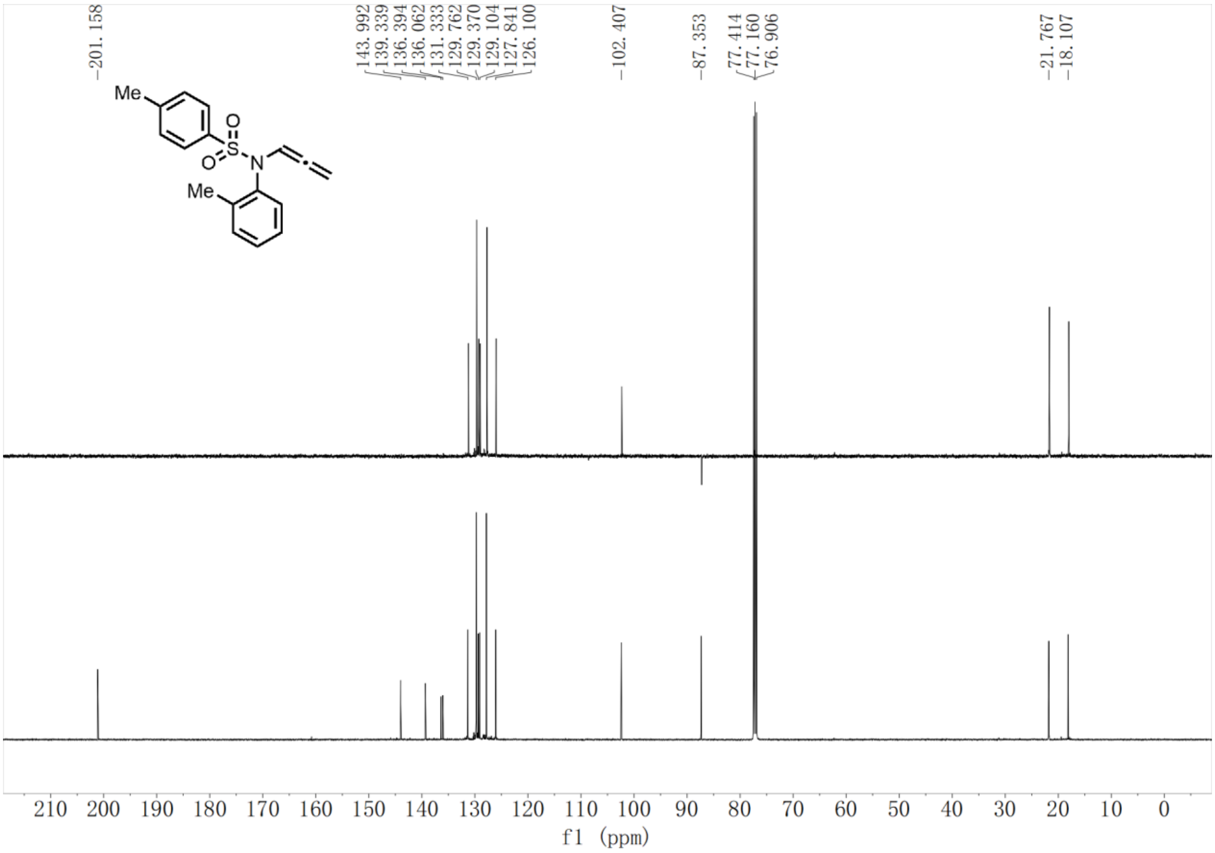

7k – ^{1}H NMR (500 MHz, $CDCl_3$)

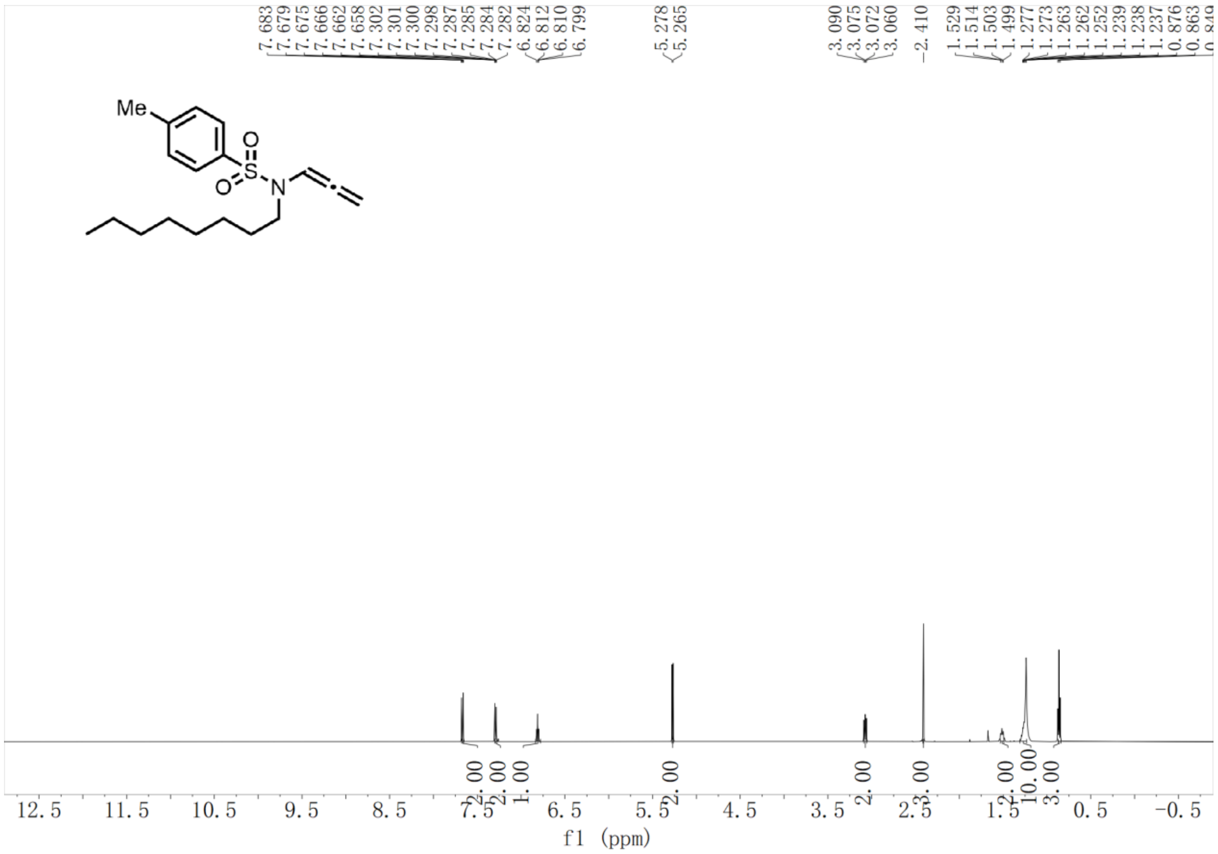

7k – ^{13}C NMR (126 MHz, $CDCl_3$)

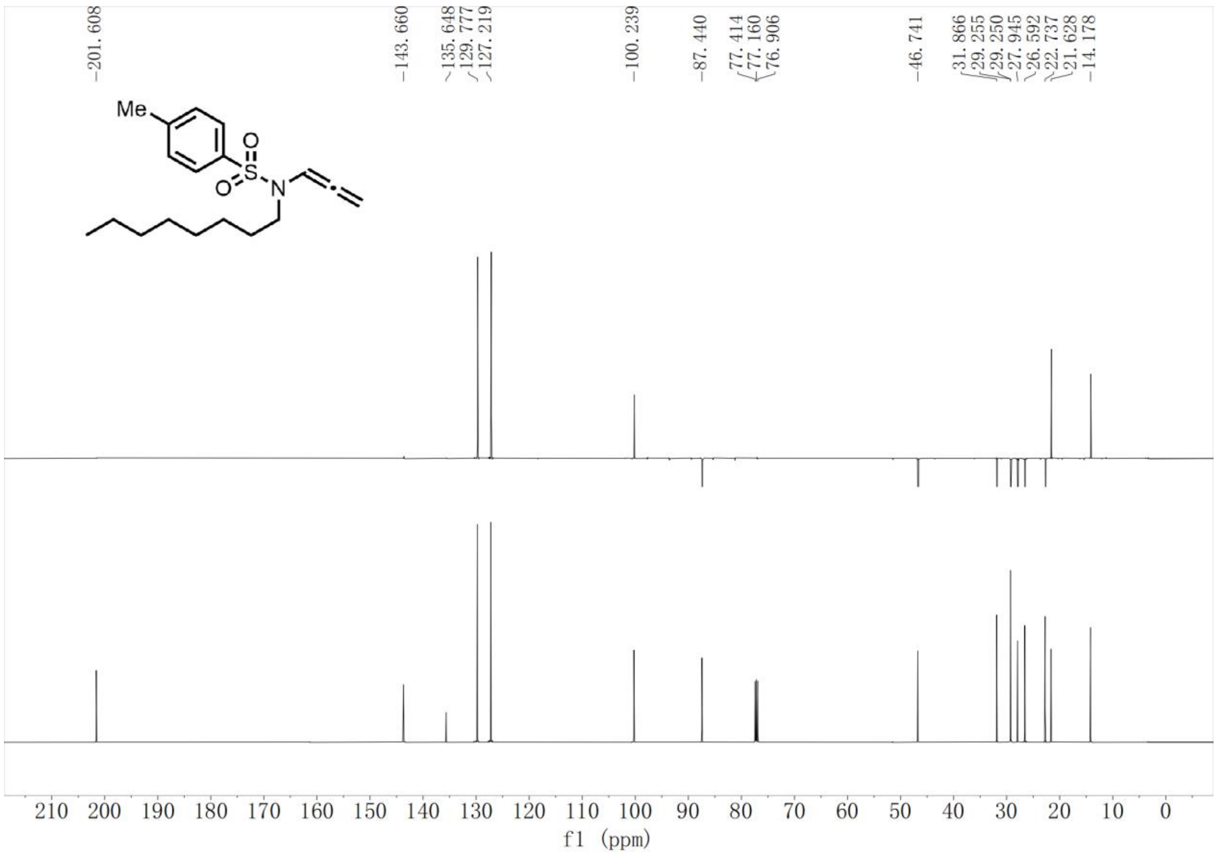

7m – ^{1}H NMR (500 MHz, $CDCl_3$)

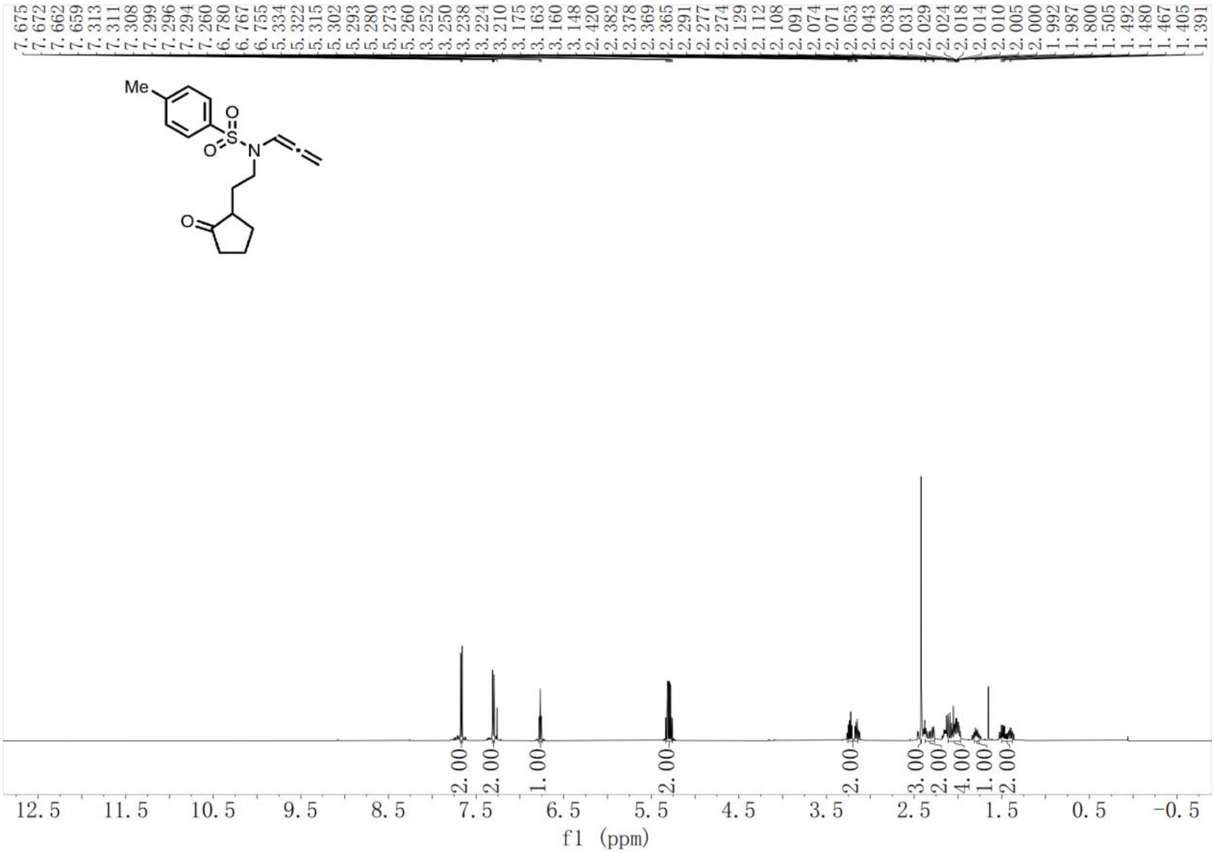

7m – ^{13}C NMR (126 MHz, $CDCl_3$)

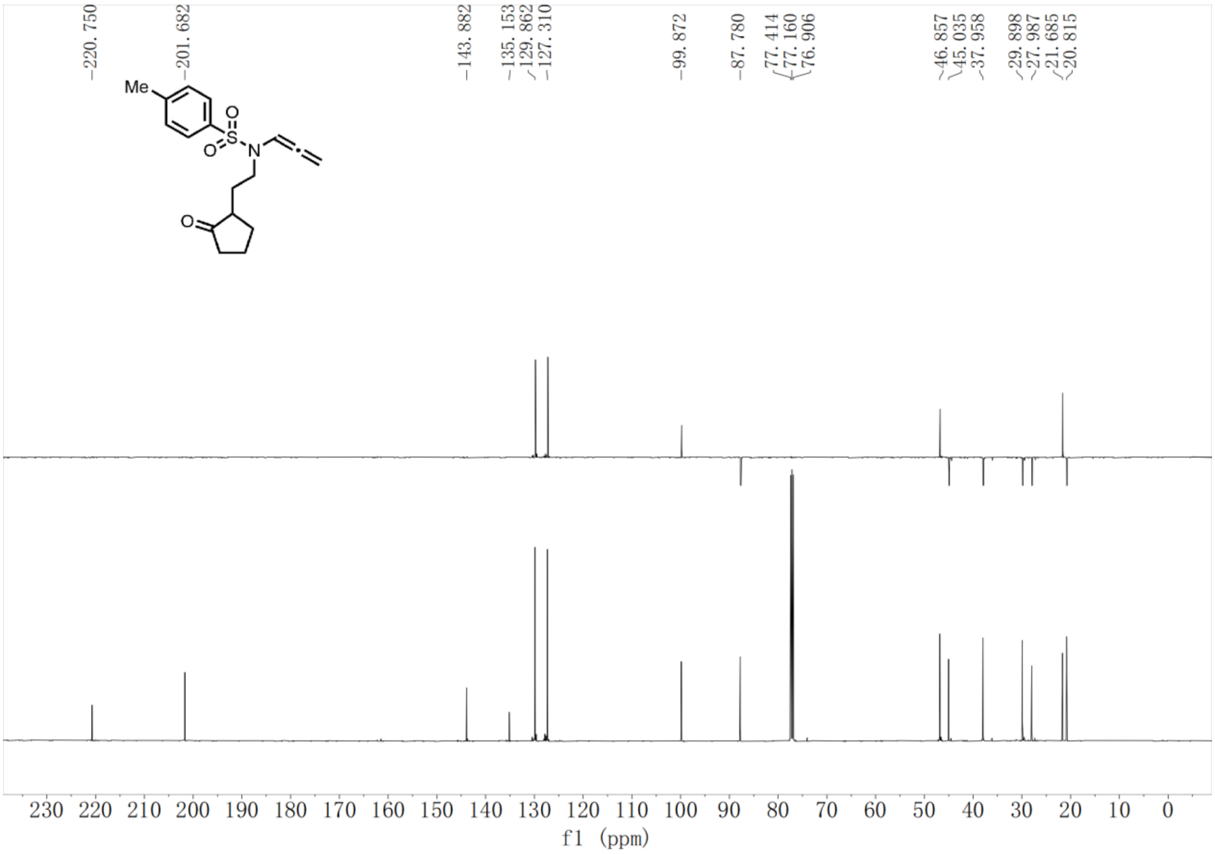

7q – ^{1}H NMR (500 MHz, $CDCl_3$)

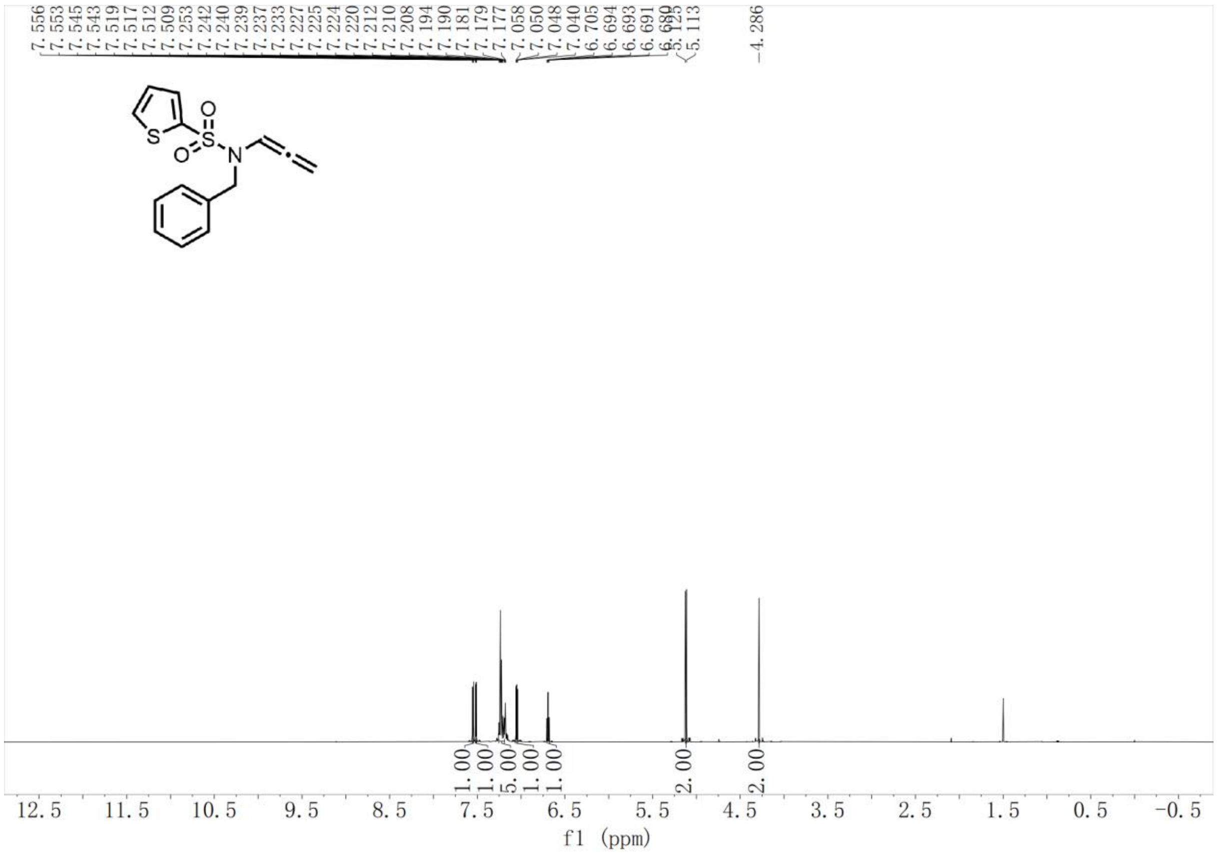

7q – ^{13}C NMR (126 MHz, $CDCl_3$)

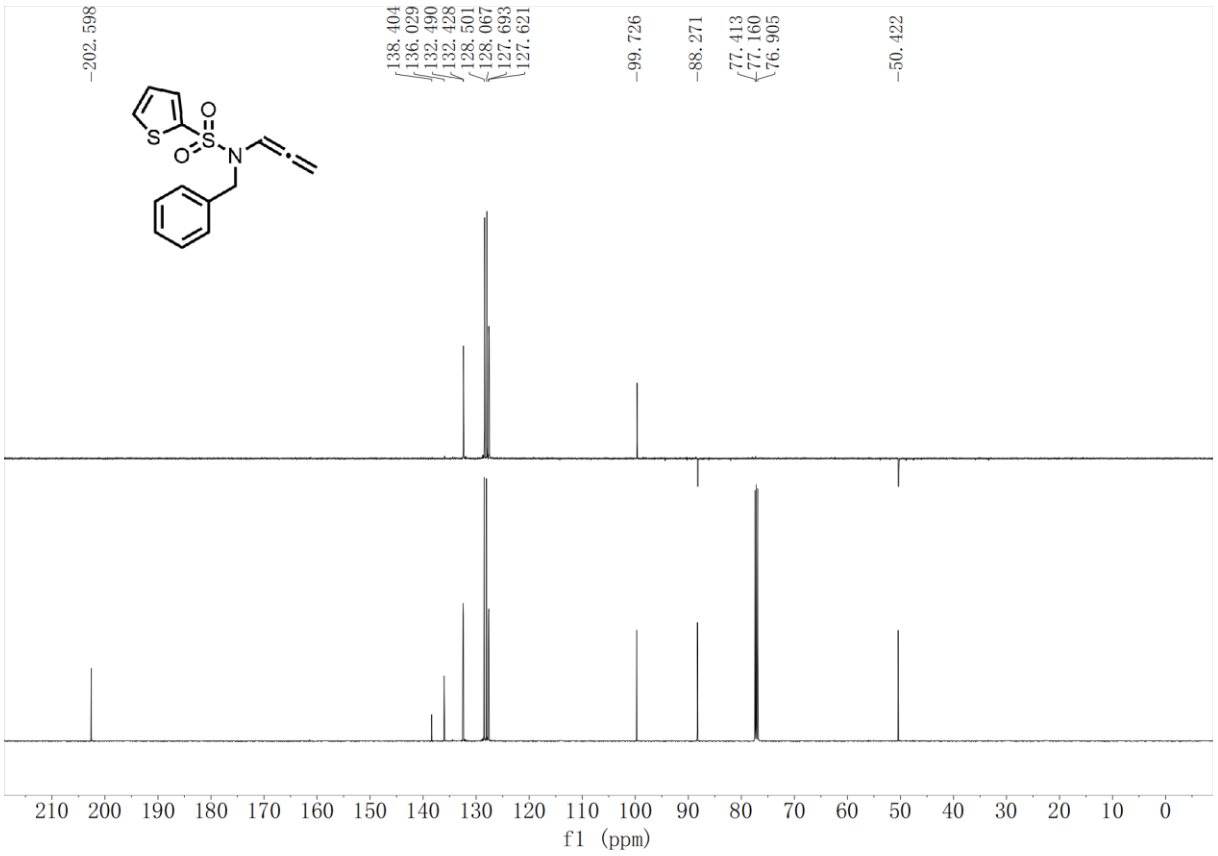

7t – ^{1}H NMR (500 MHz, $CDCl_3$)

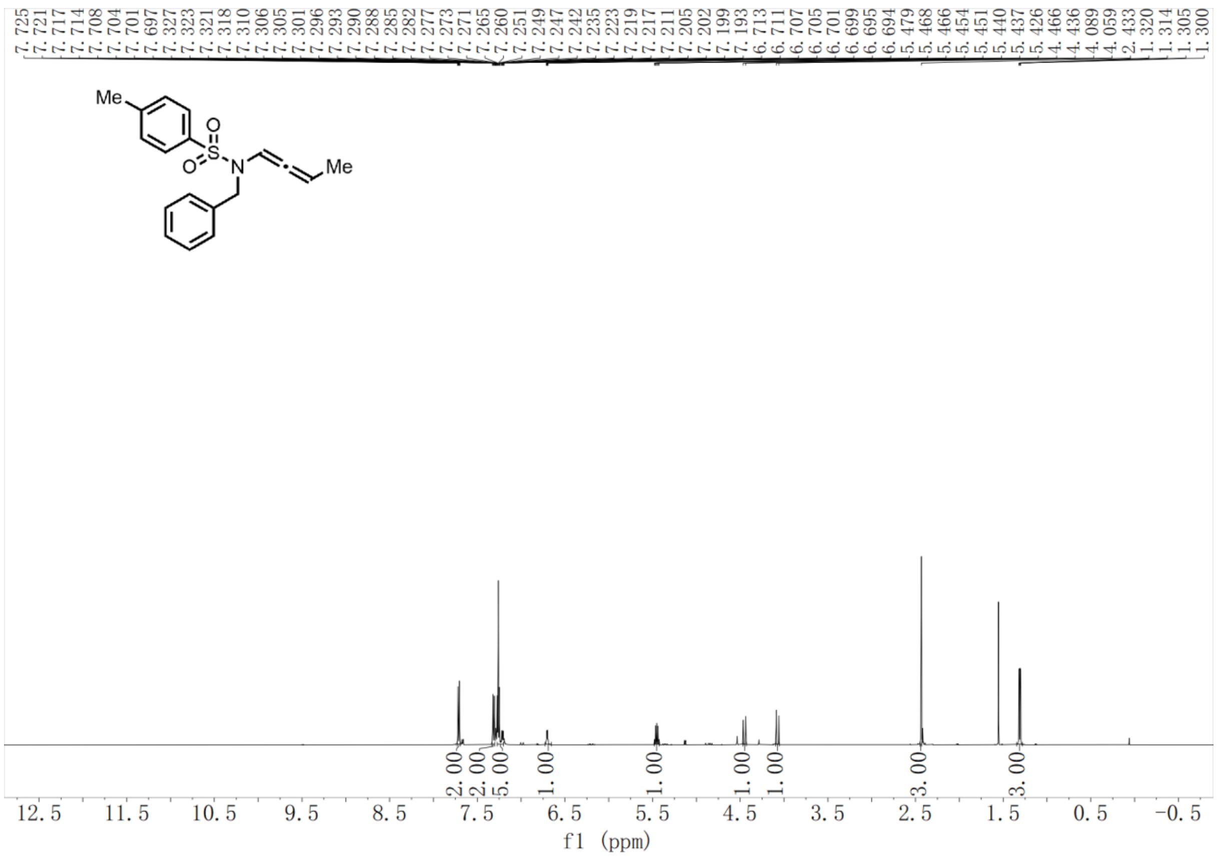

7t – ^{13}C NMR (126 MHz, $CDCl_3$)

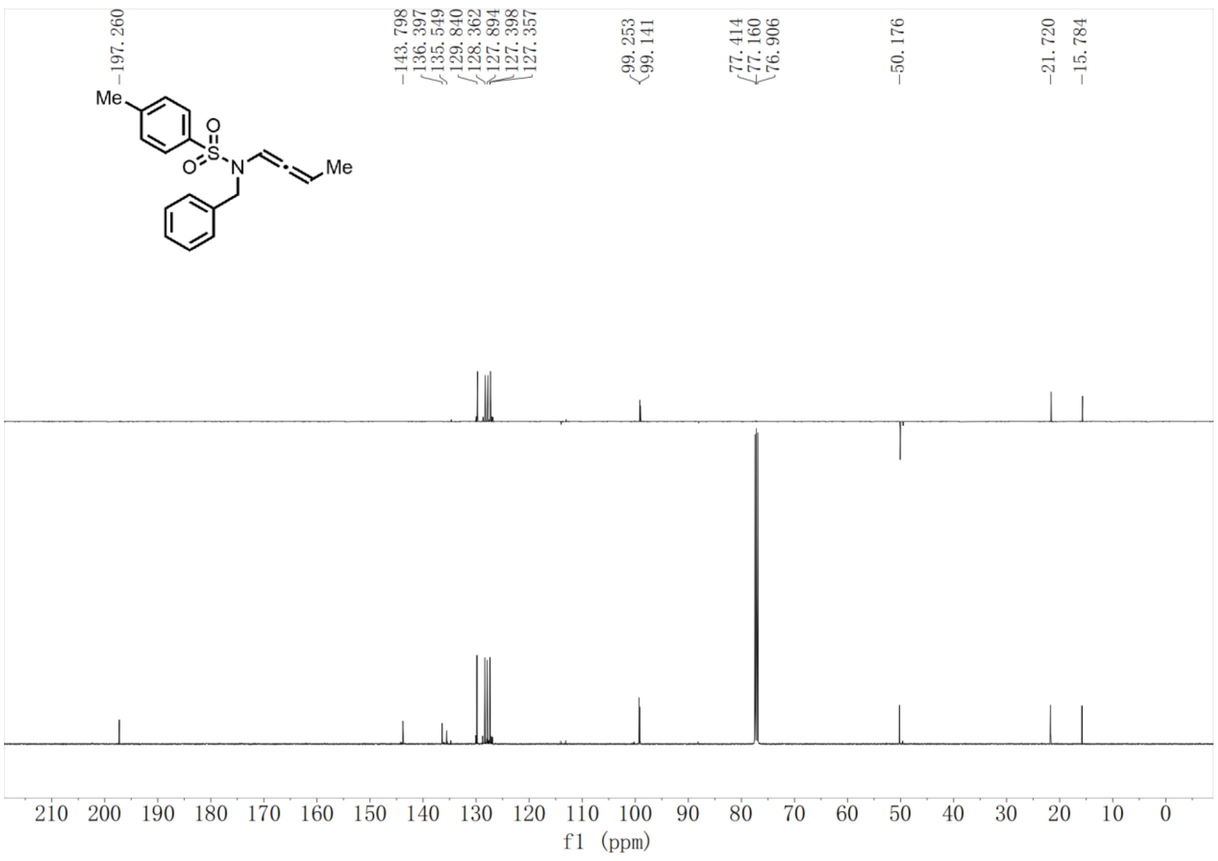

8a – ^{1}H NMR (500 MHz, $CDCl_3$)

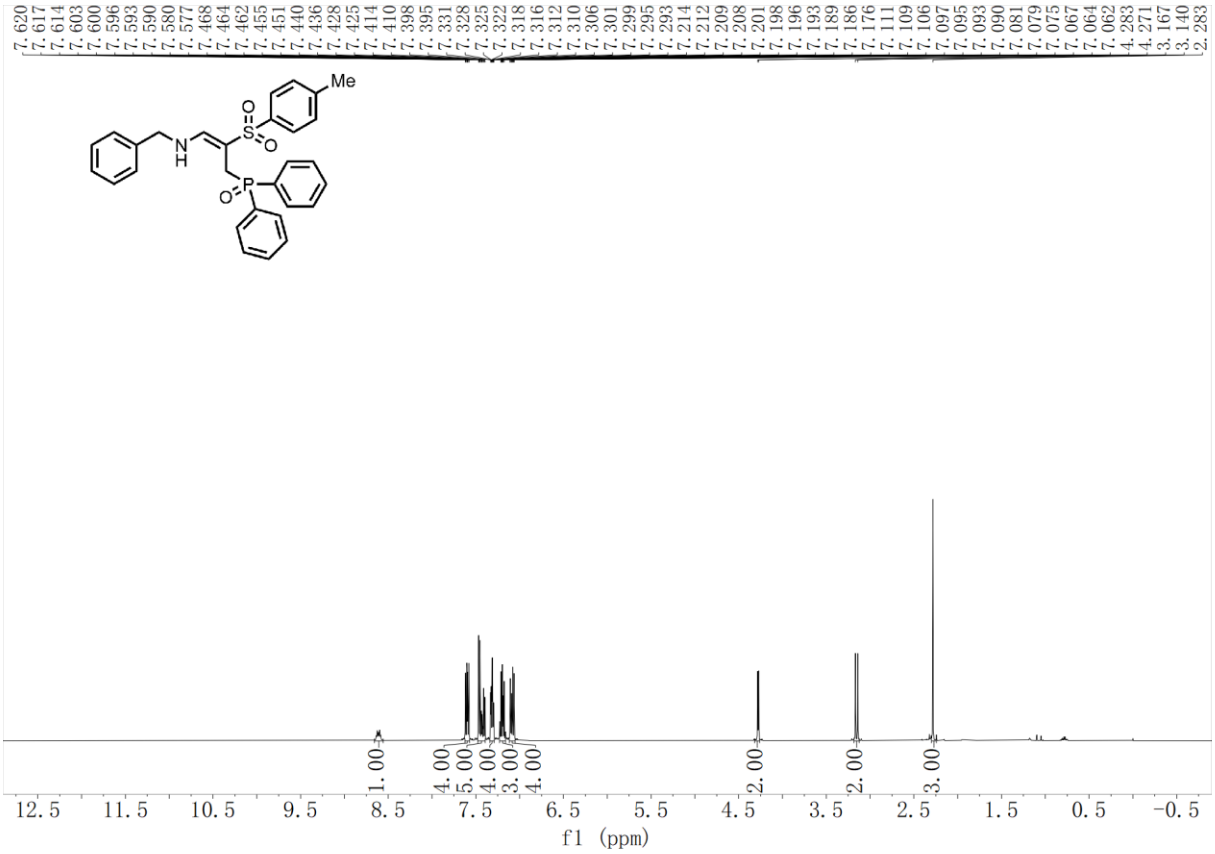

8a – ^{13}C NMR (126 MHz, $CDCl_3$)

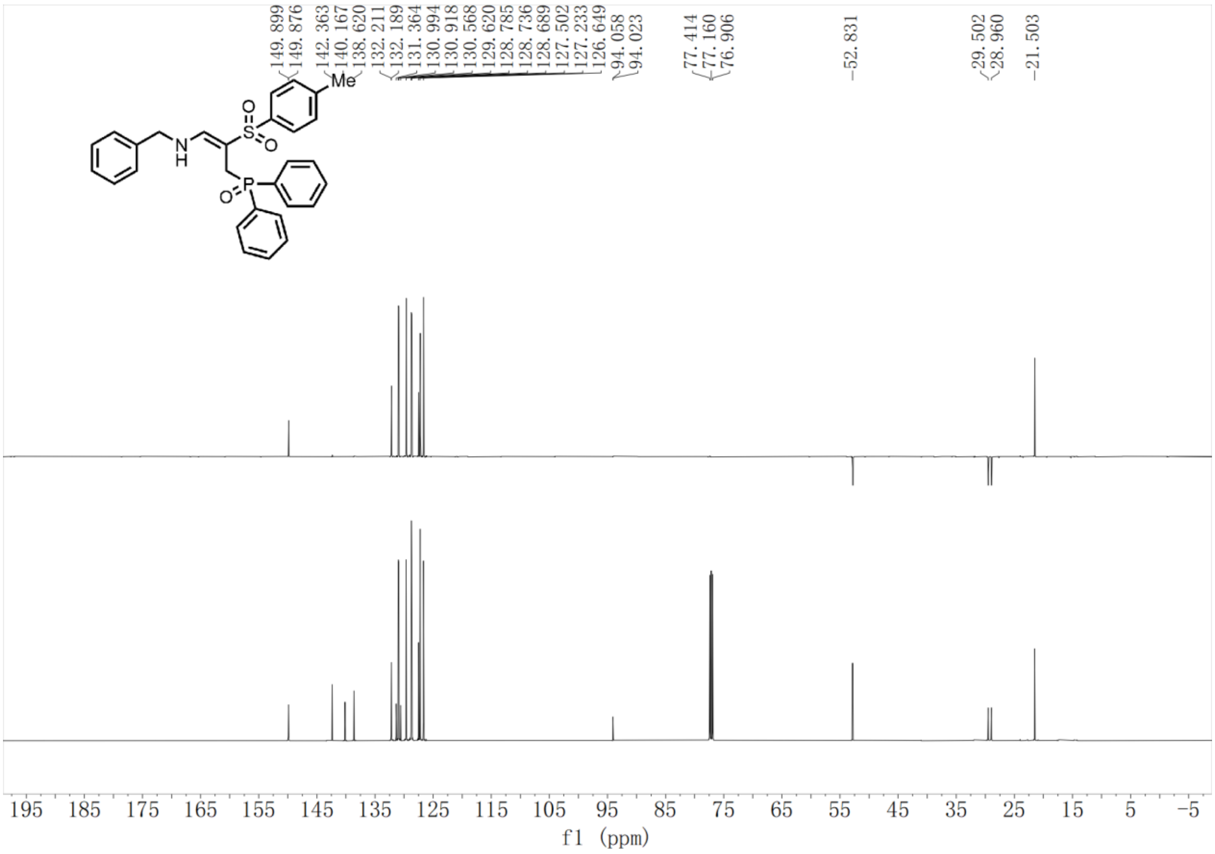

8a – ^{31}P NMR (121 MHz, $CDCl_3$)

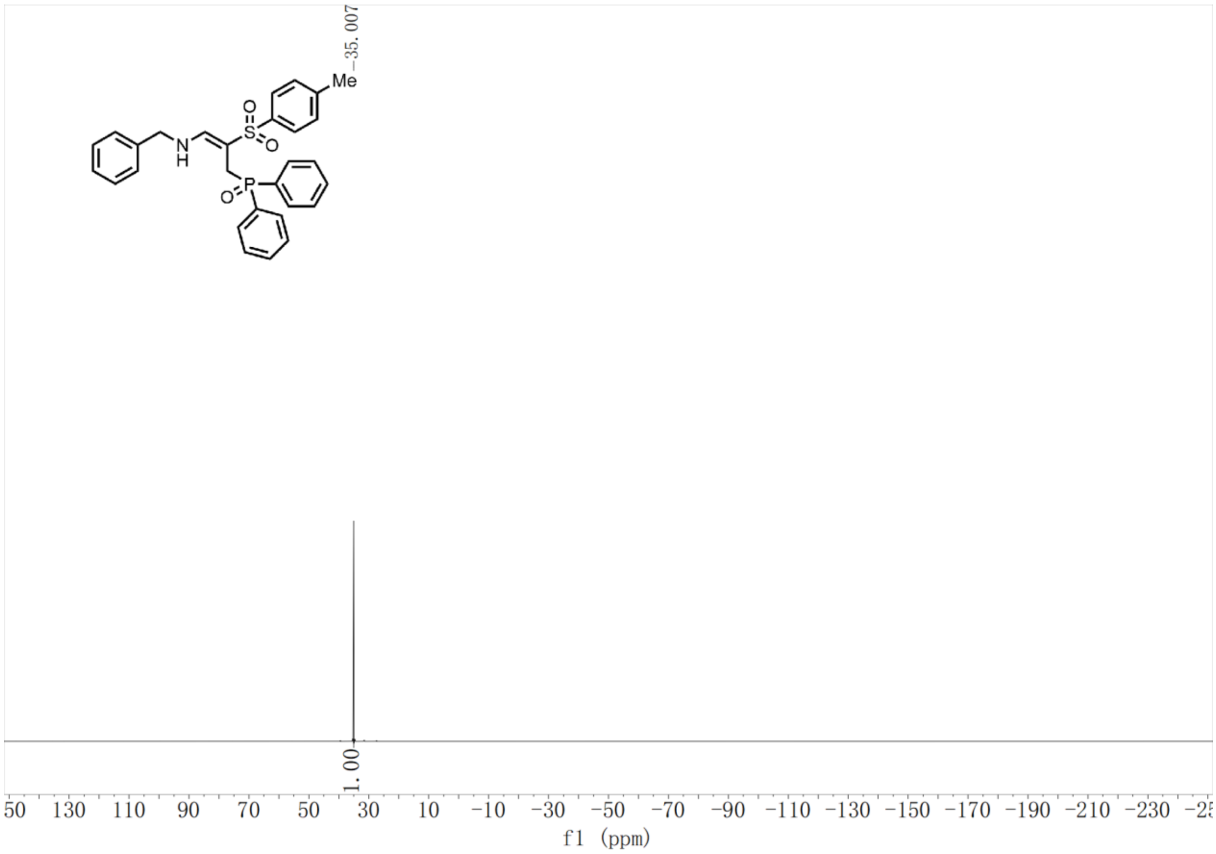

8b – ^{1}H NMR (500 MHz, $CDCl_3$)

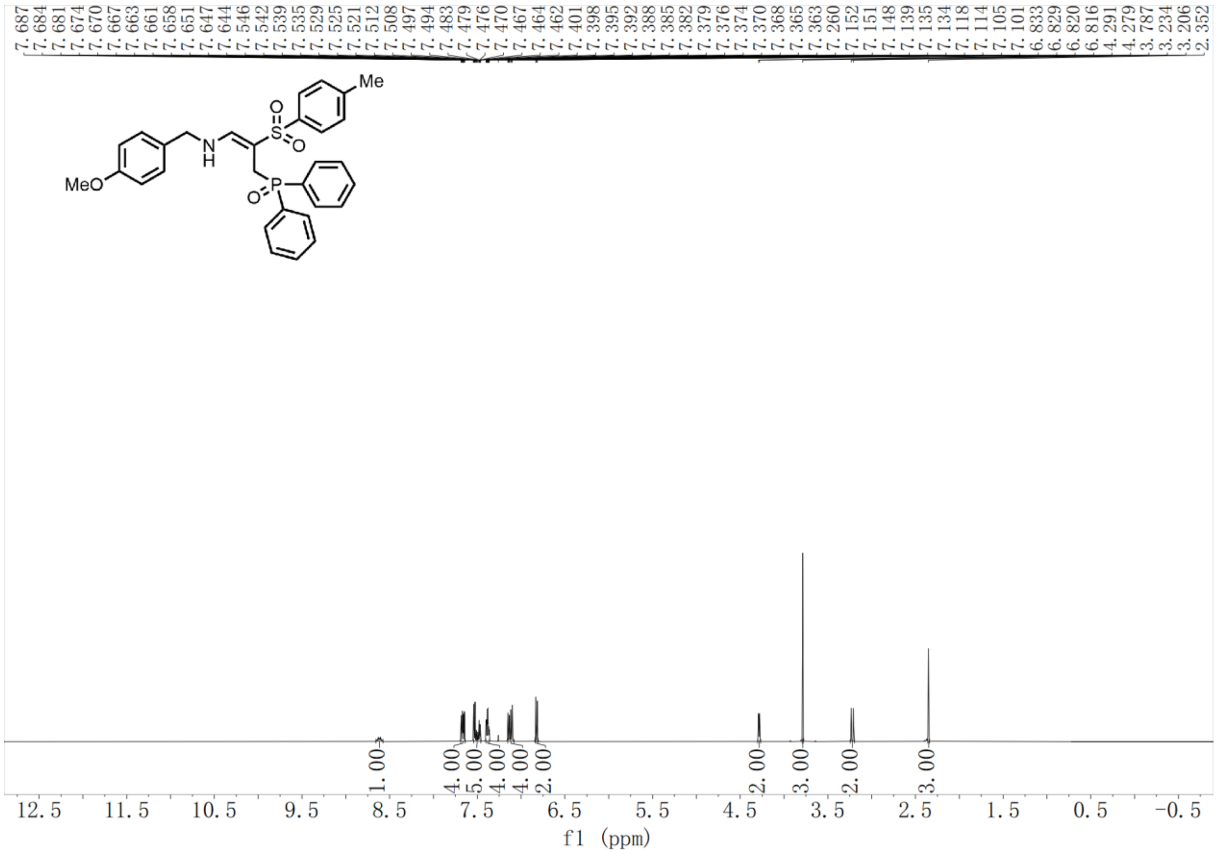

8b – ^{13}C NMR (126 MHz, $CDCl_3$)

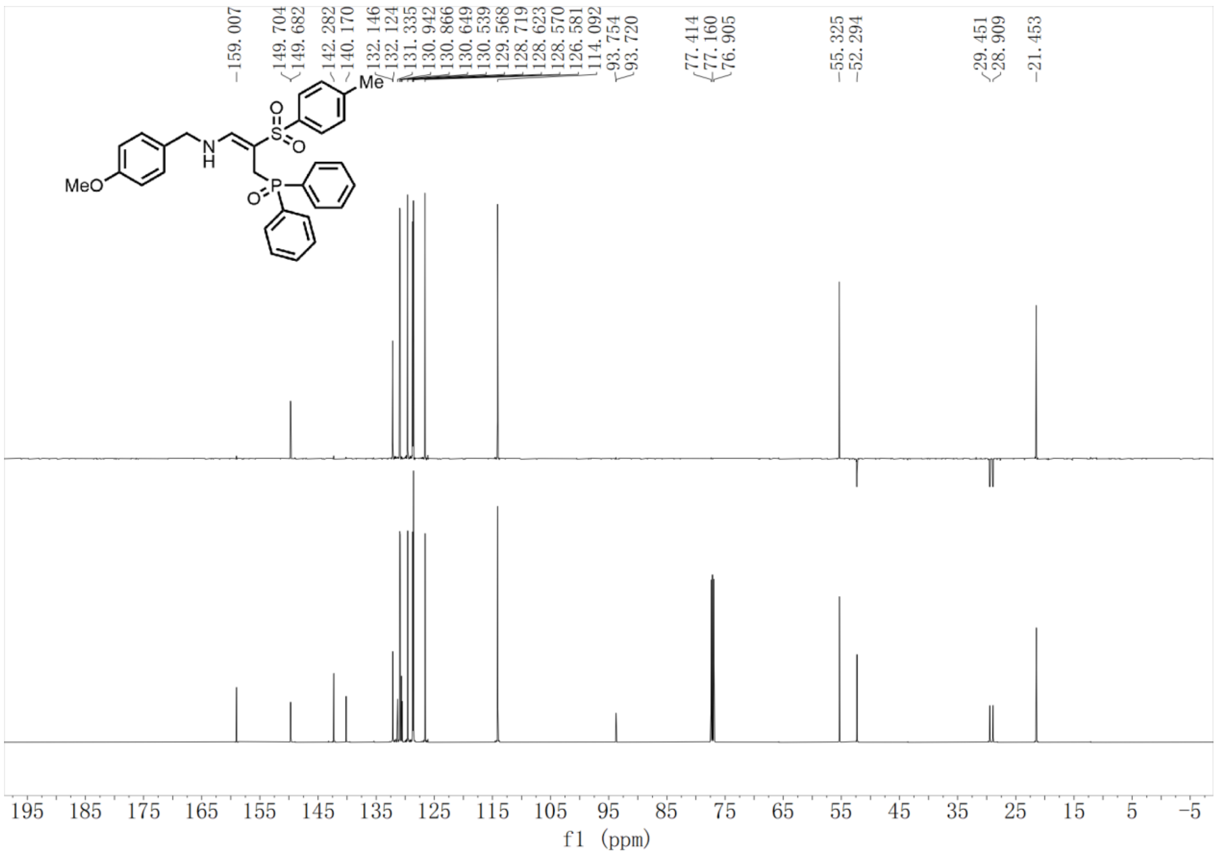

8b – ^{31}P NMR (121 MHz, CDCl$_3$)

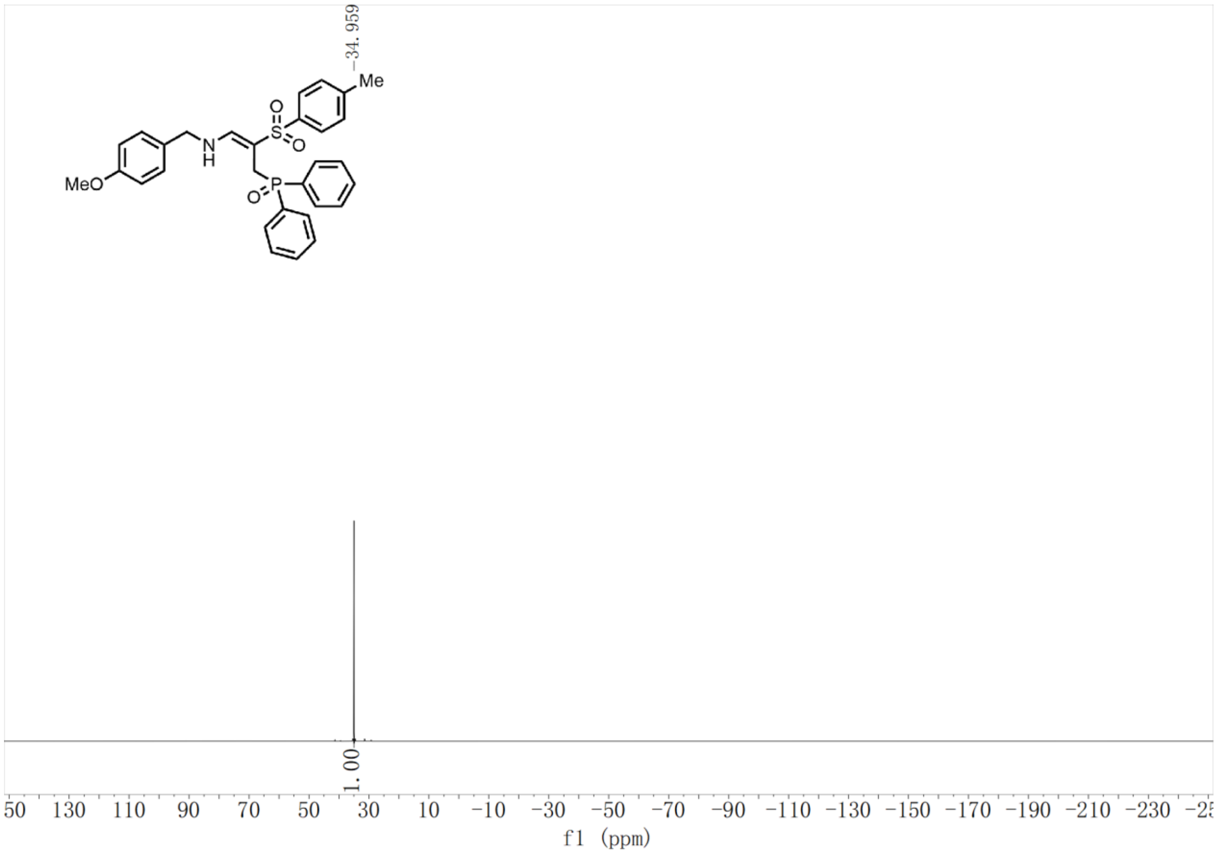

8c – ^{1}H NMR (500 MHz, $CDCl_3$)

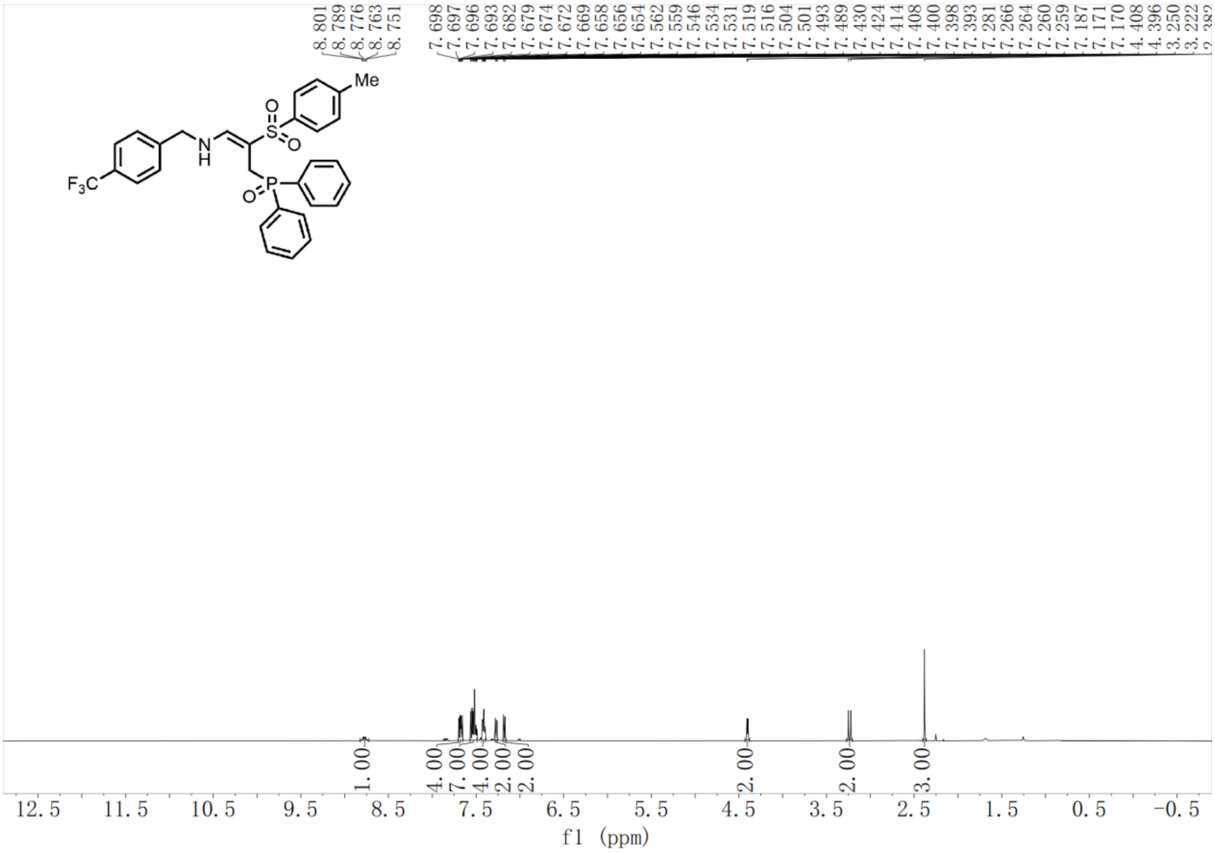

8c – ^{13}C NMR (126 MHz, $CDCl_3$)

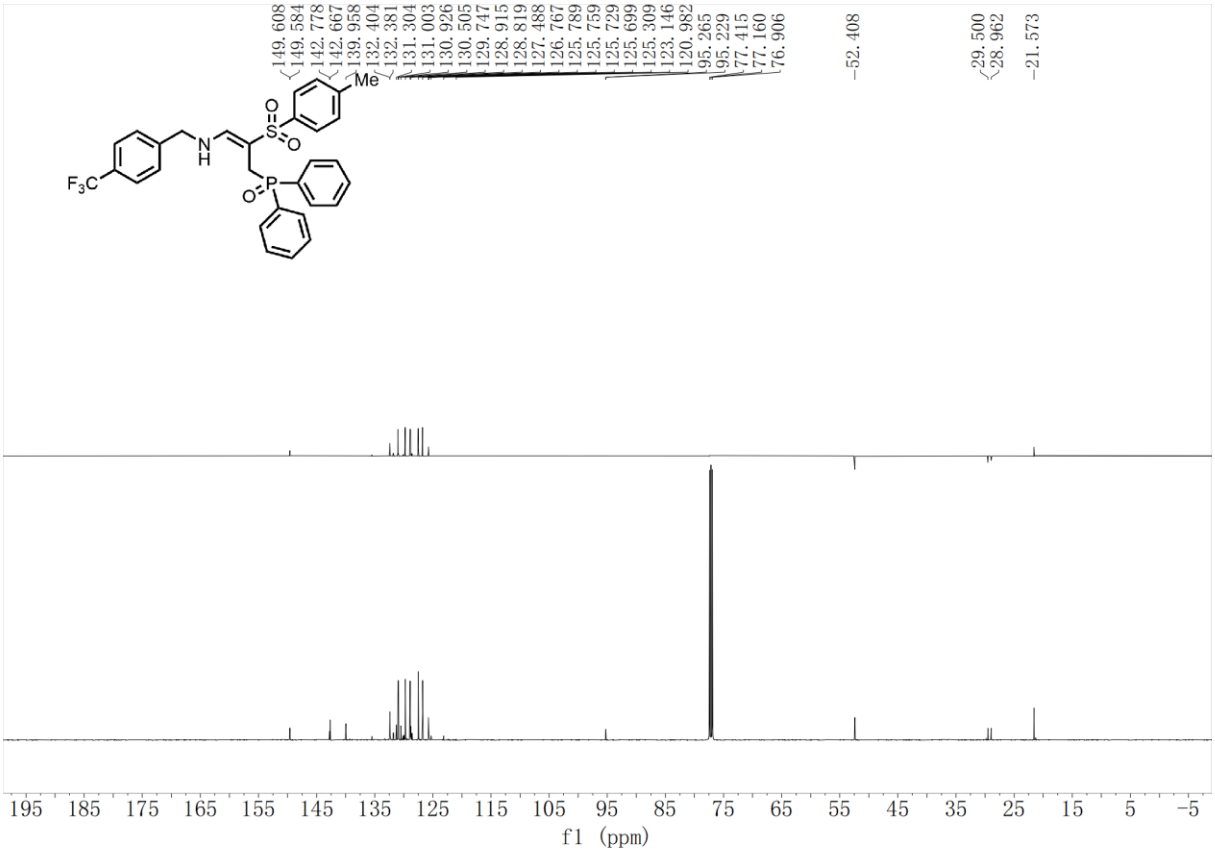

8c – ^{19}F NMR (471 MHz, $CDCl_3$)

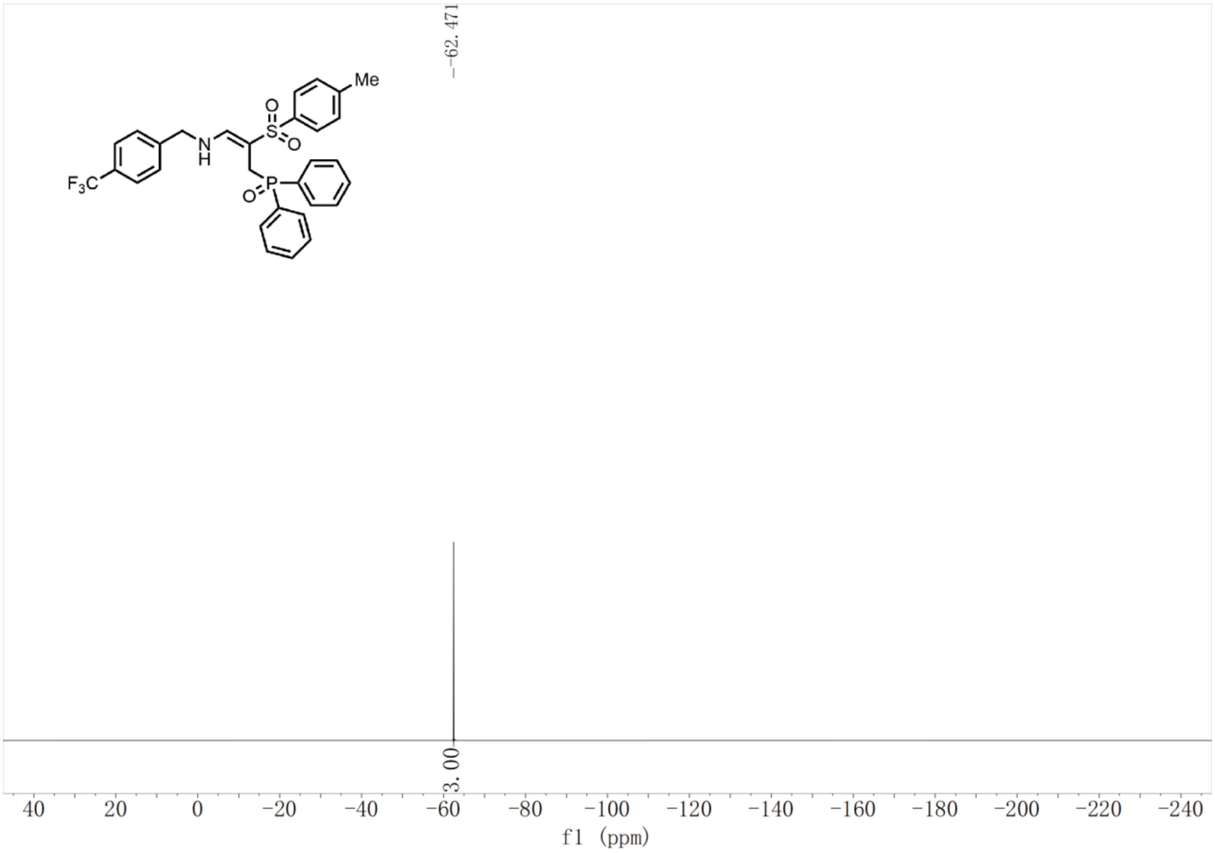

8c – ^{31}P NMR (202 MHz, $CDCl_3$)

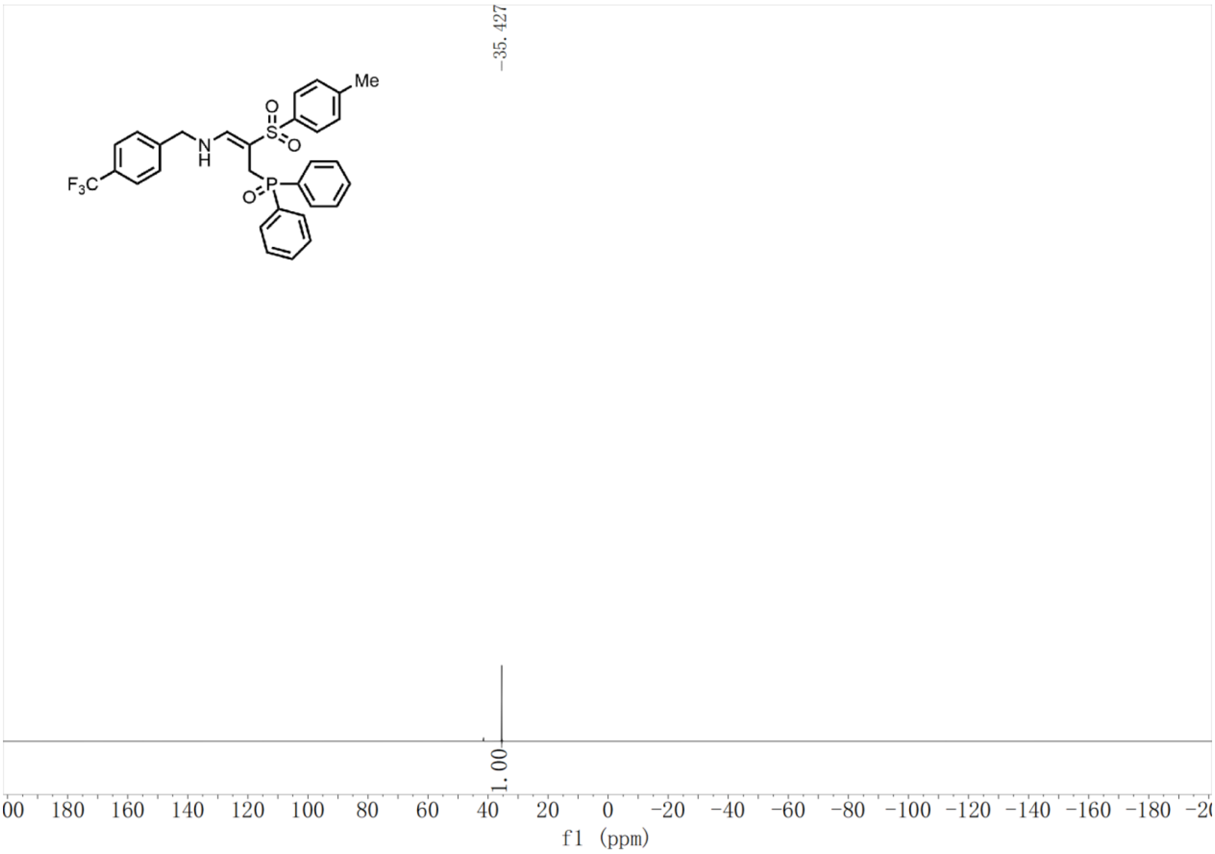

8d – ^{1}H NMR (500 MHz, $CDCl_3$)

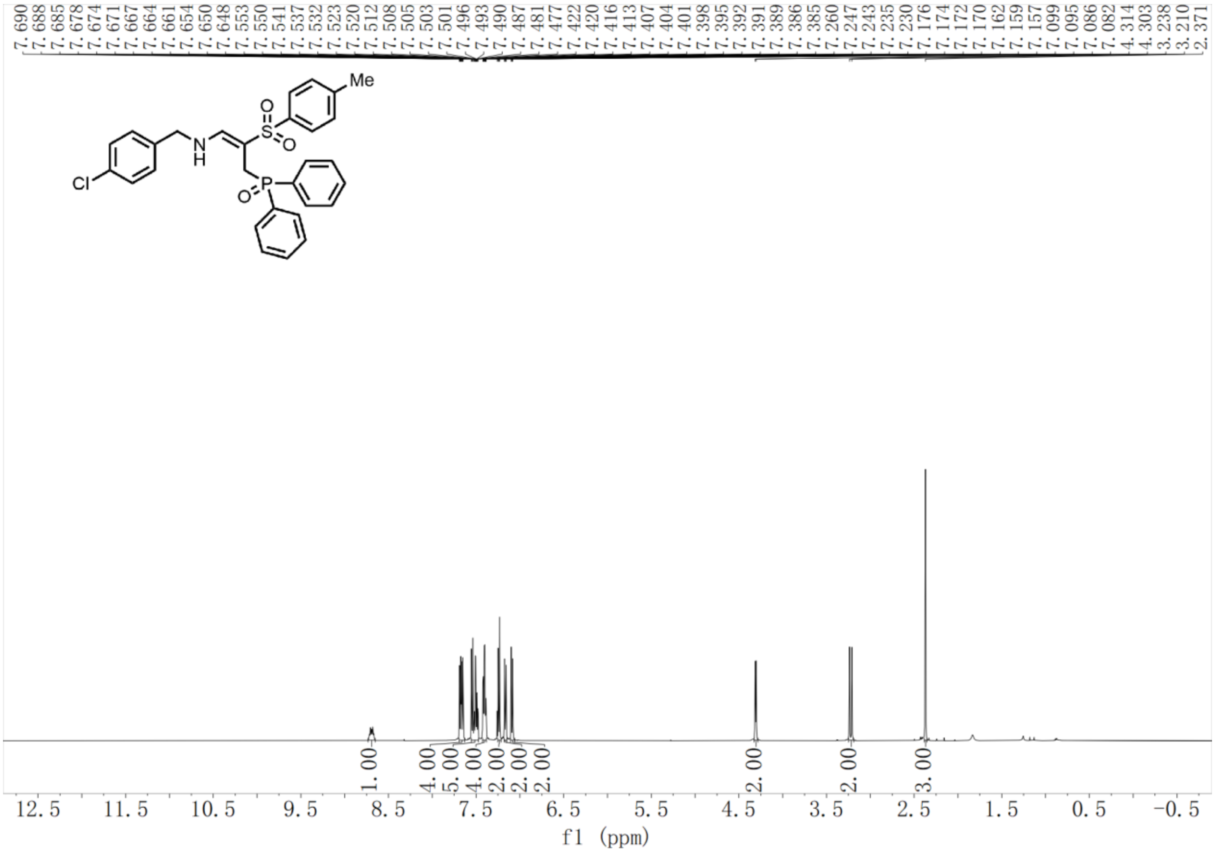

8d – ^{13}C NMR (126 MHz, $CDCl_3$)

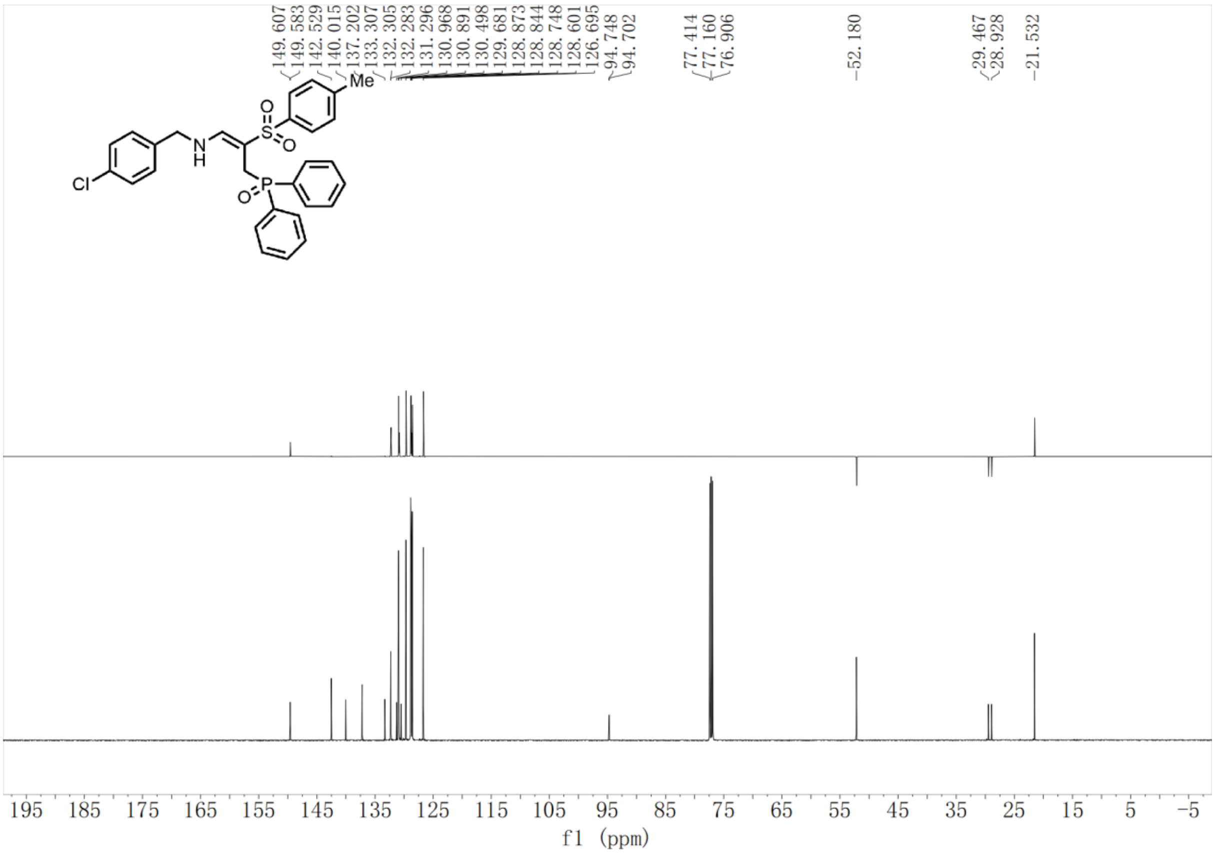

8d – ^{31}P NMR (121 MHz, $CDCl_3$)

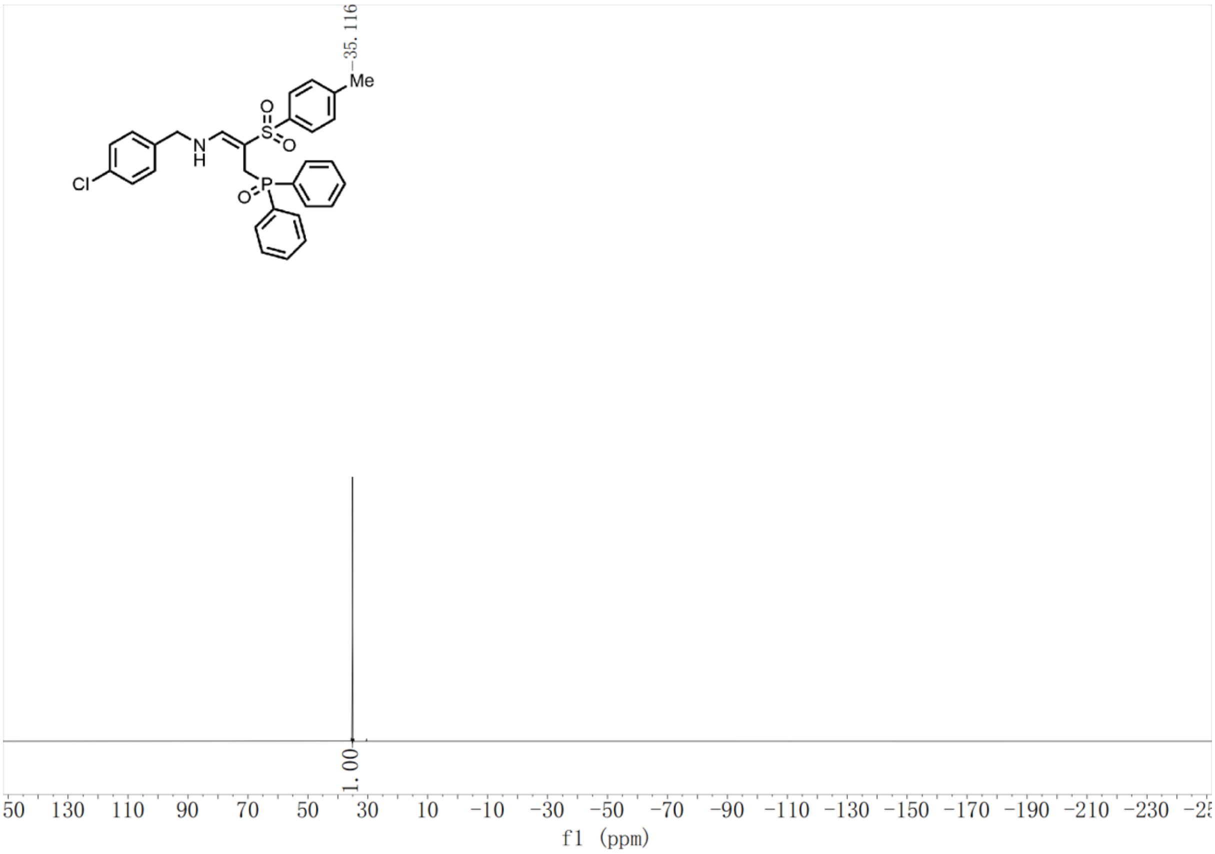

8e – ¹H NMR (300 MHz, $CDCl_3$)

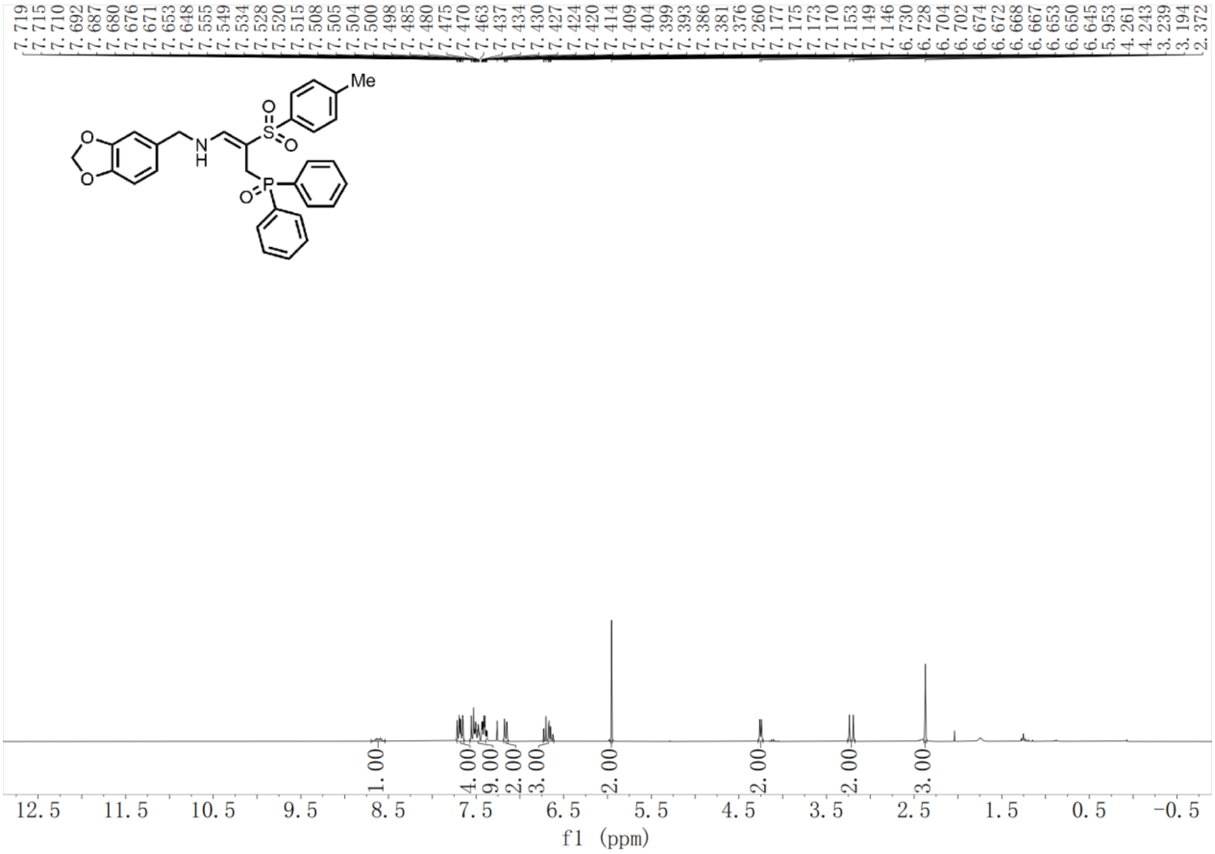

8e – ¹³C NMR (126 MHz, $CDCl_3$)

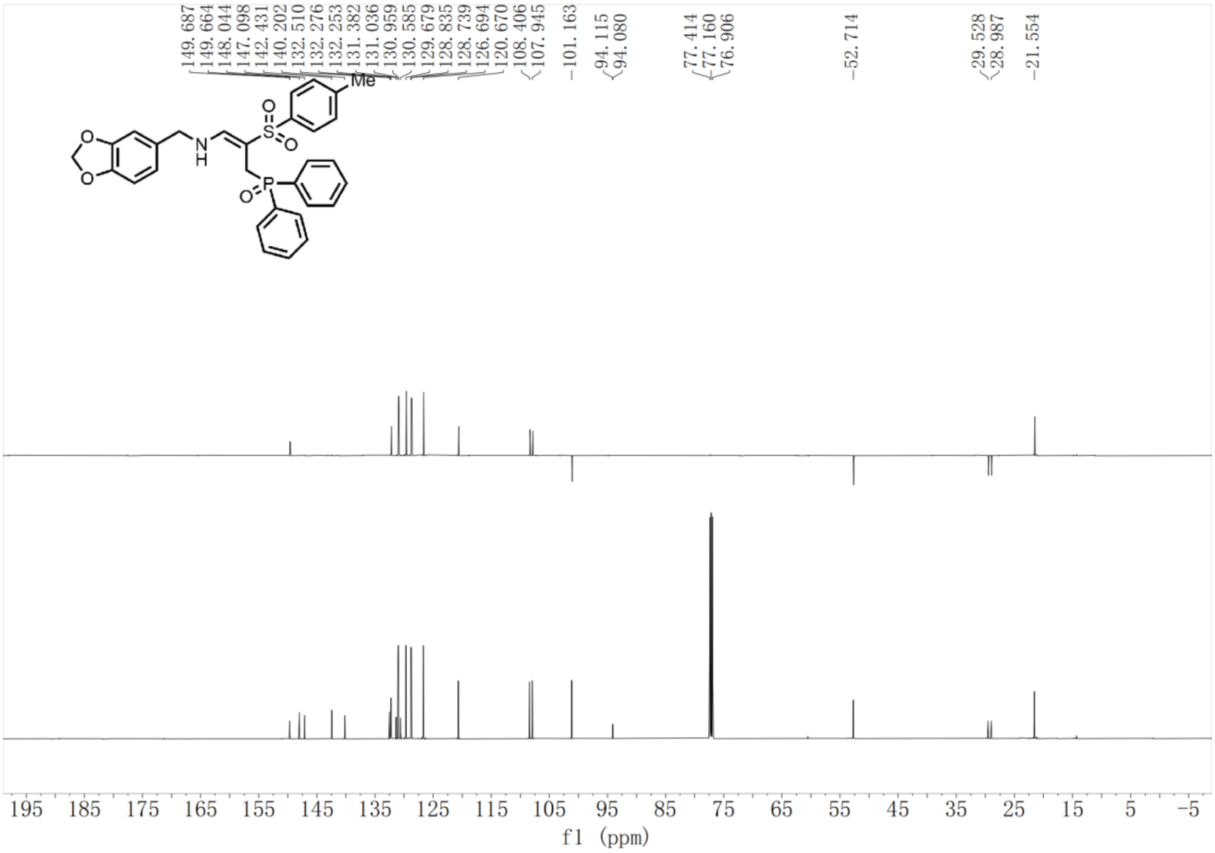

8e – ^{31}P NMR (121 MHz, $CDCl_3$)

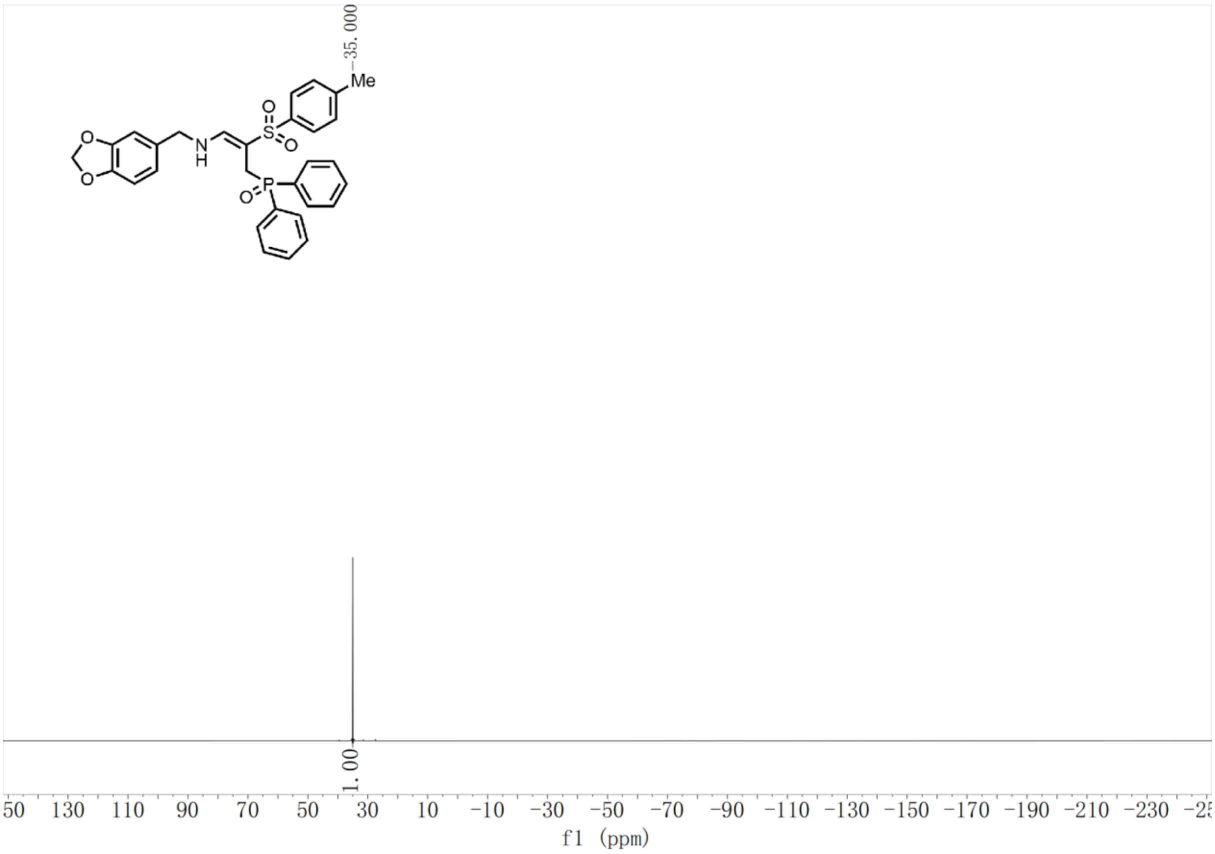

8f – ^{1}H NMR (500 MHz, $CDCl_3$)

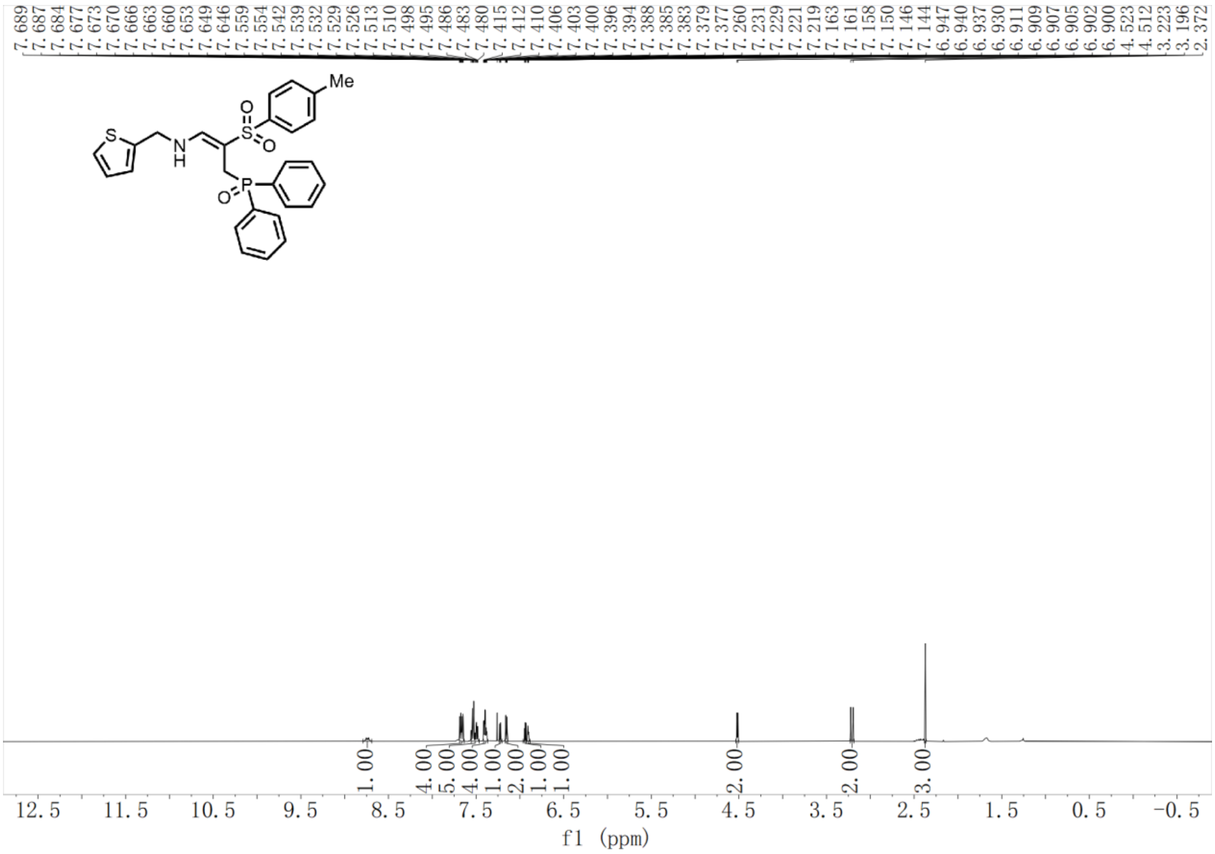

8f – ^{13}C NMR (126 MHz, $CDCl_3$)

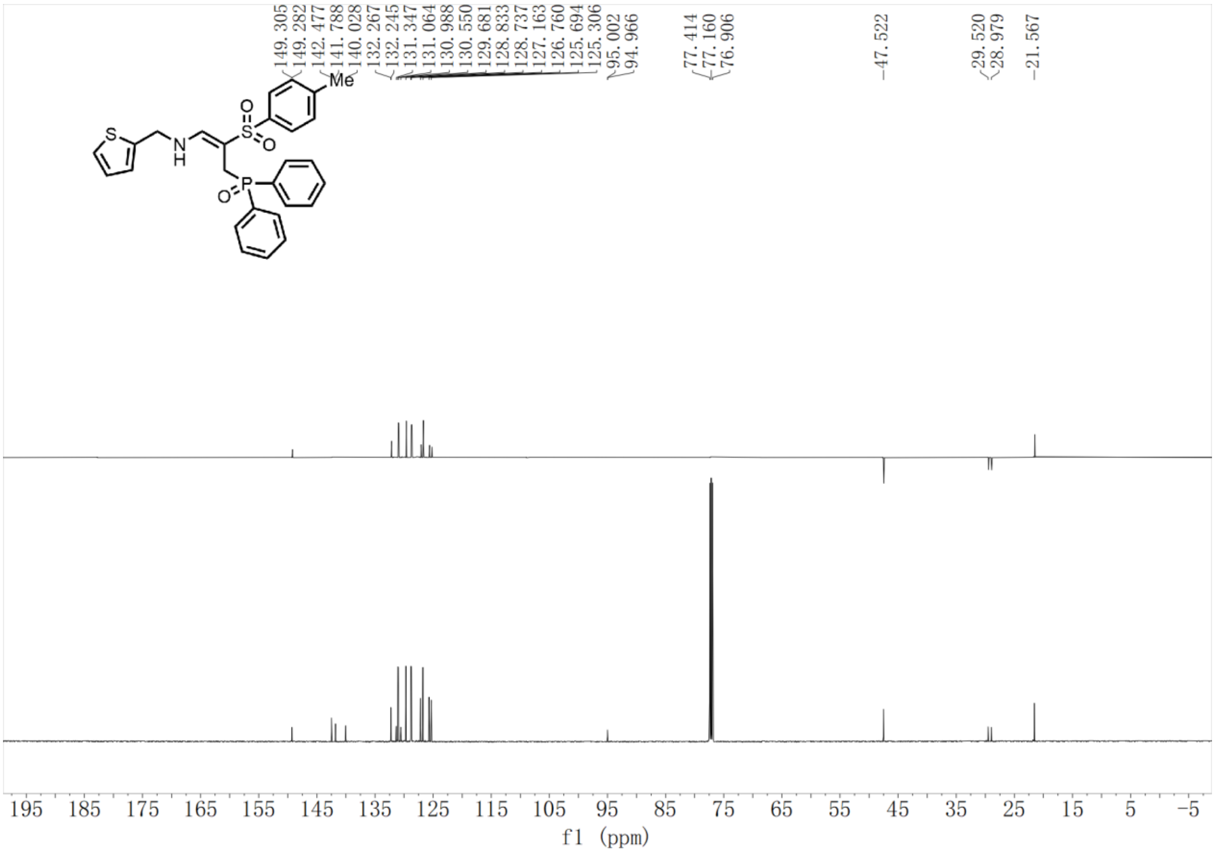

8f – ^{31}P NMR (121 MHz, $CDCl_3$)

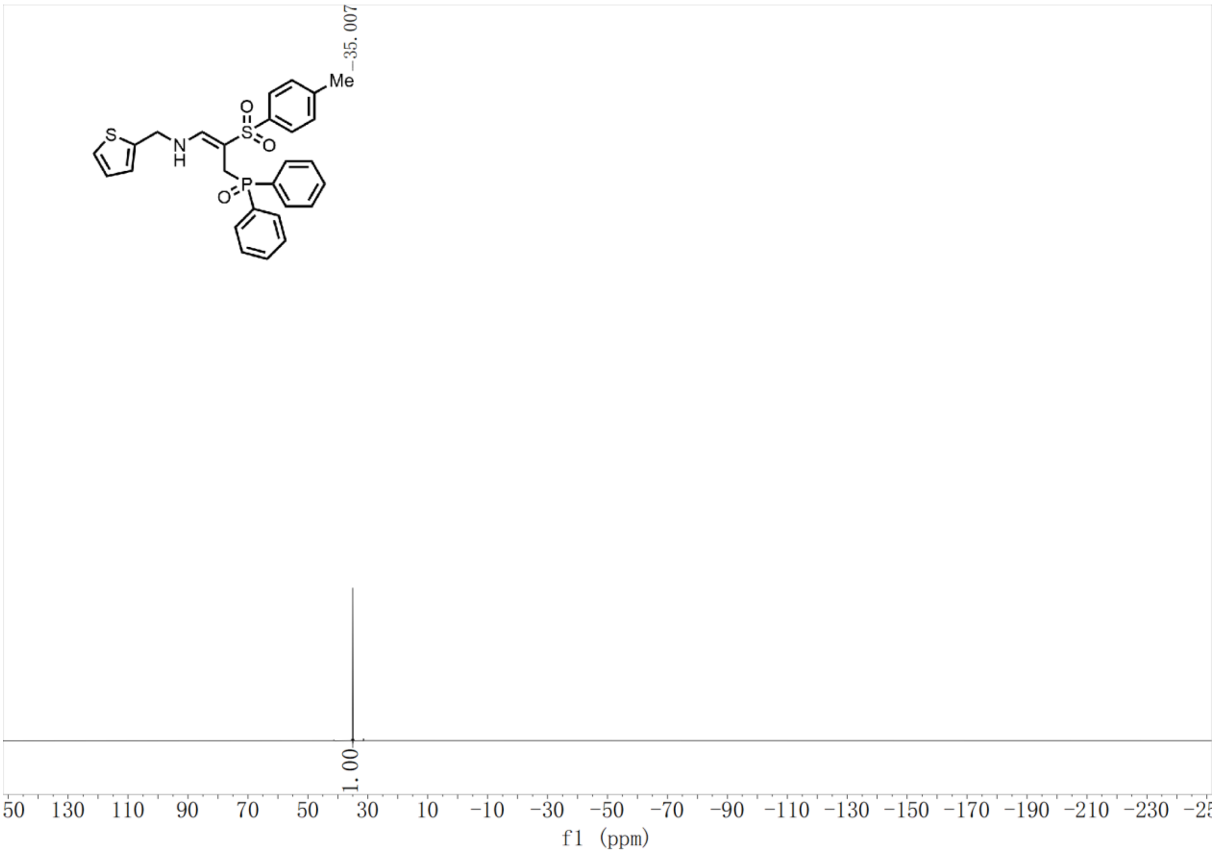

8g – ^{1}H NMR (500 MHz, $CDCl_3$)

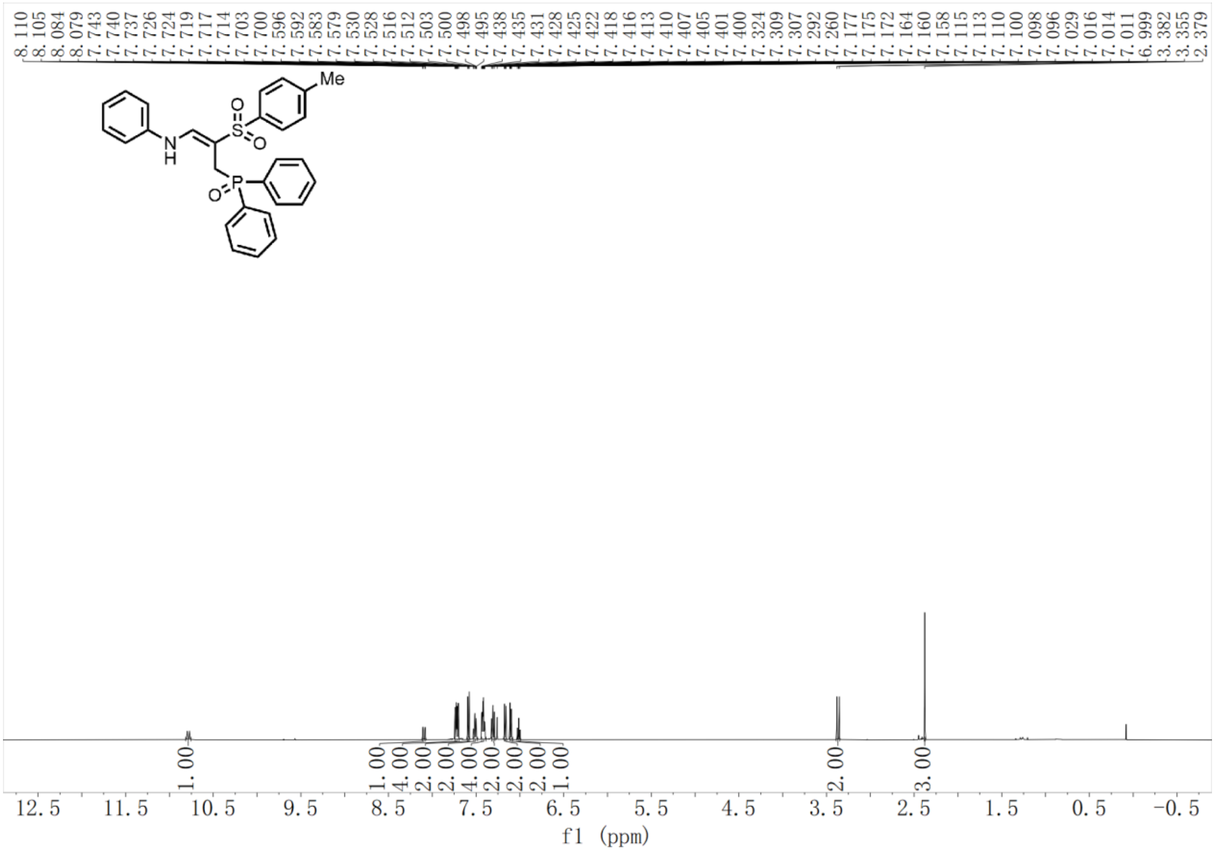

8g – ^{13}C NMR (126 MHz, $CDCl_3$)

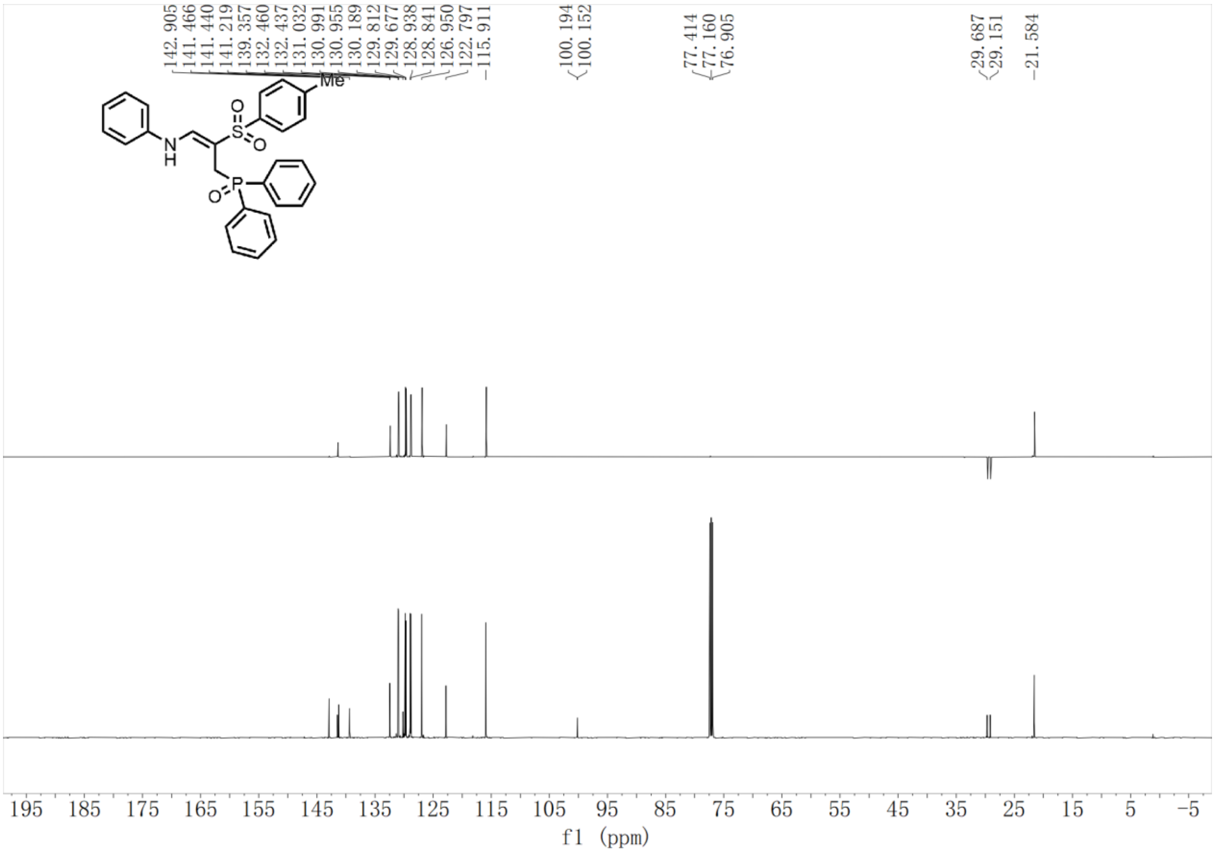

8g – ^{31}P NMR (202 MHz, $CDCl_3$)

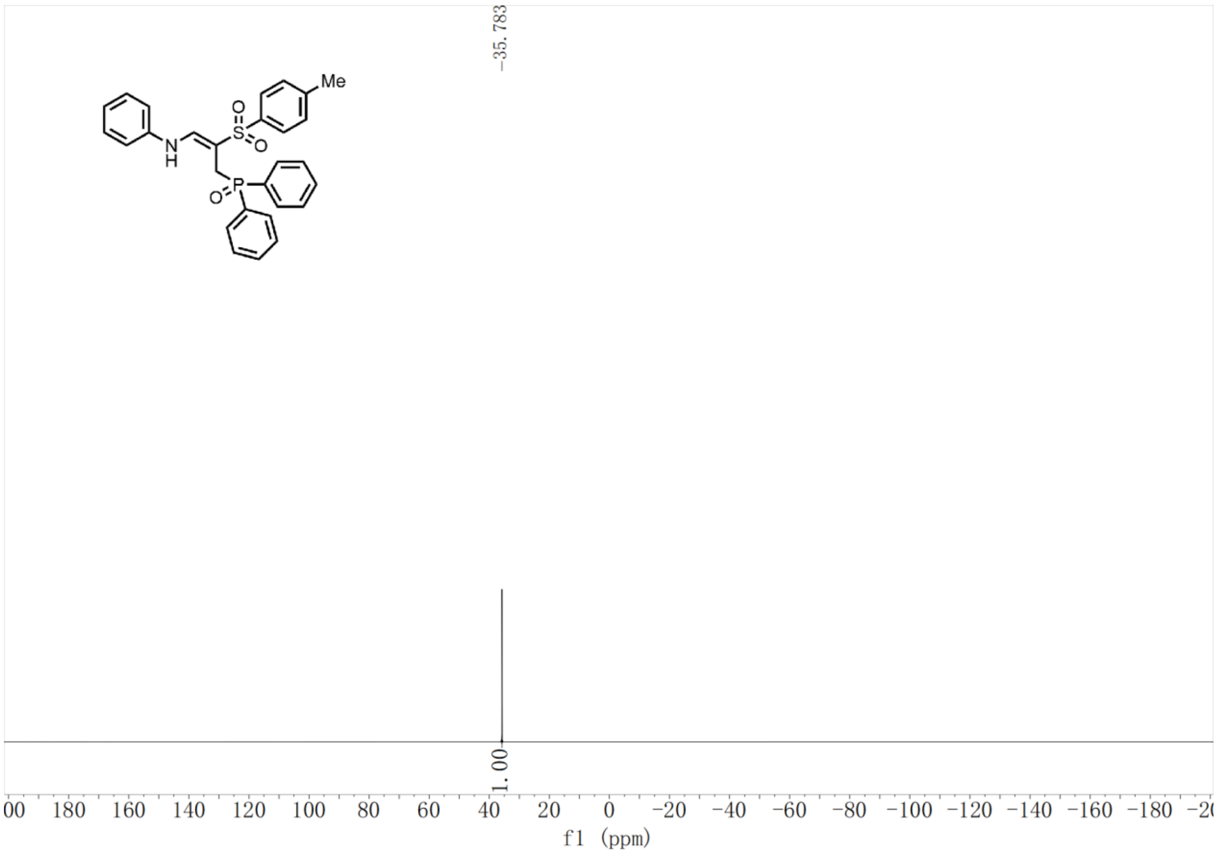

8h – ^{1}H NMR (500 MHz, $CDCl_3$)

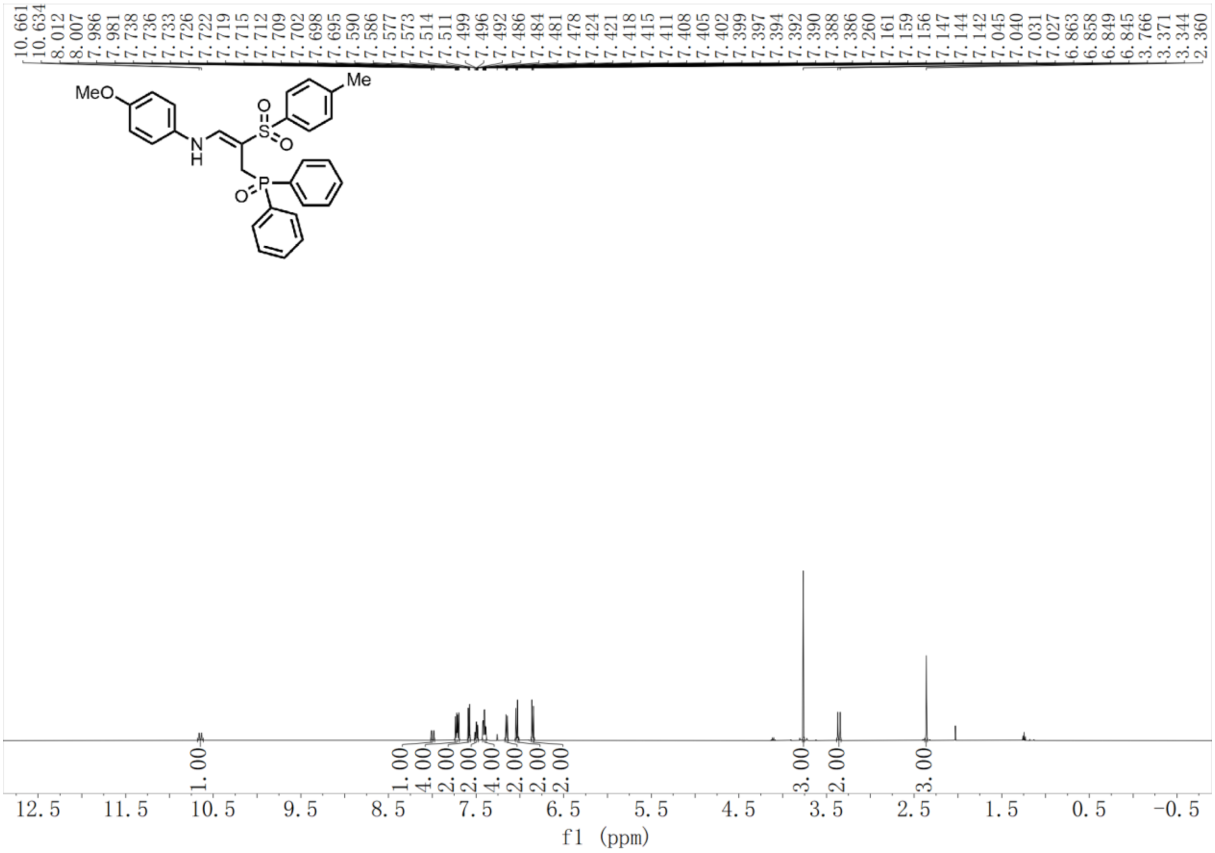

8h – ^{13}C NMR (126 MHz, $CDCl_3$)

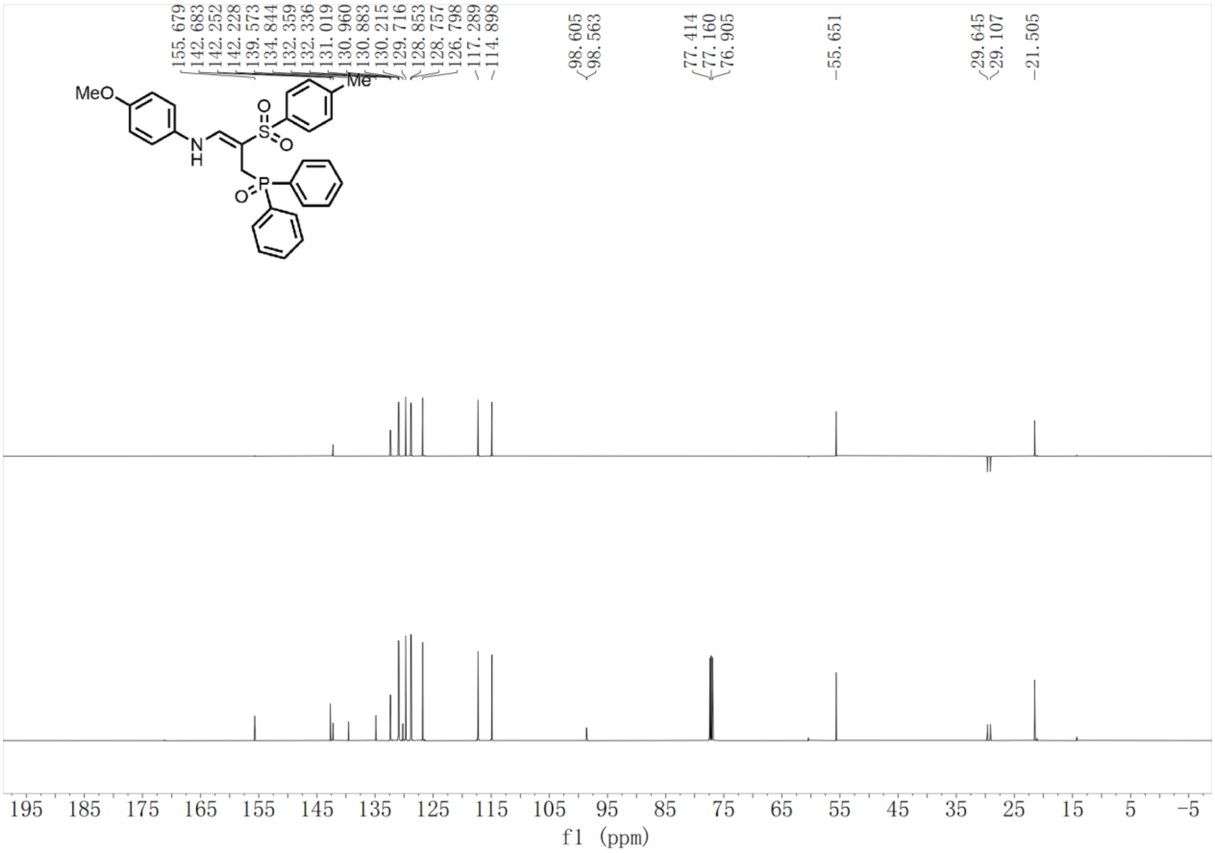

8h – ^{31}P NMR (121 MHz, $CDCl_3$)

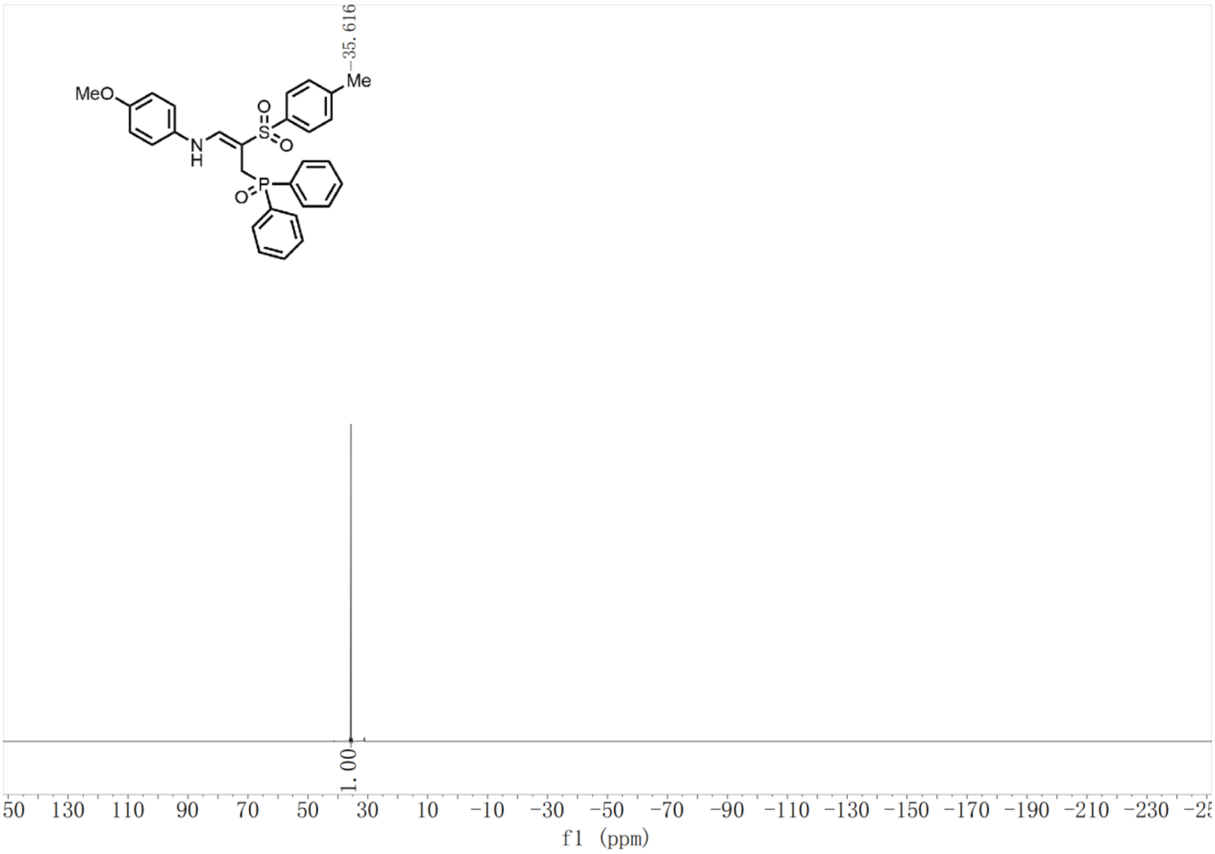

8i – ^{1}H NMR (500 MHz, $CDCl_3$)

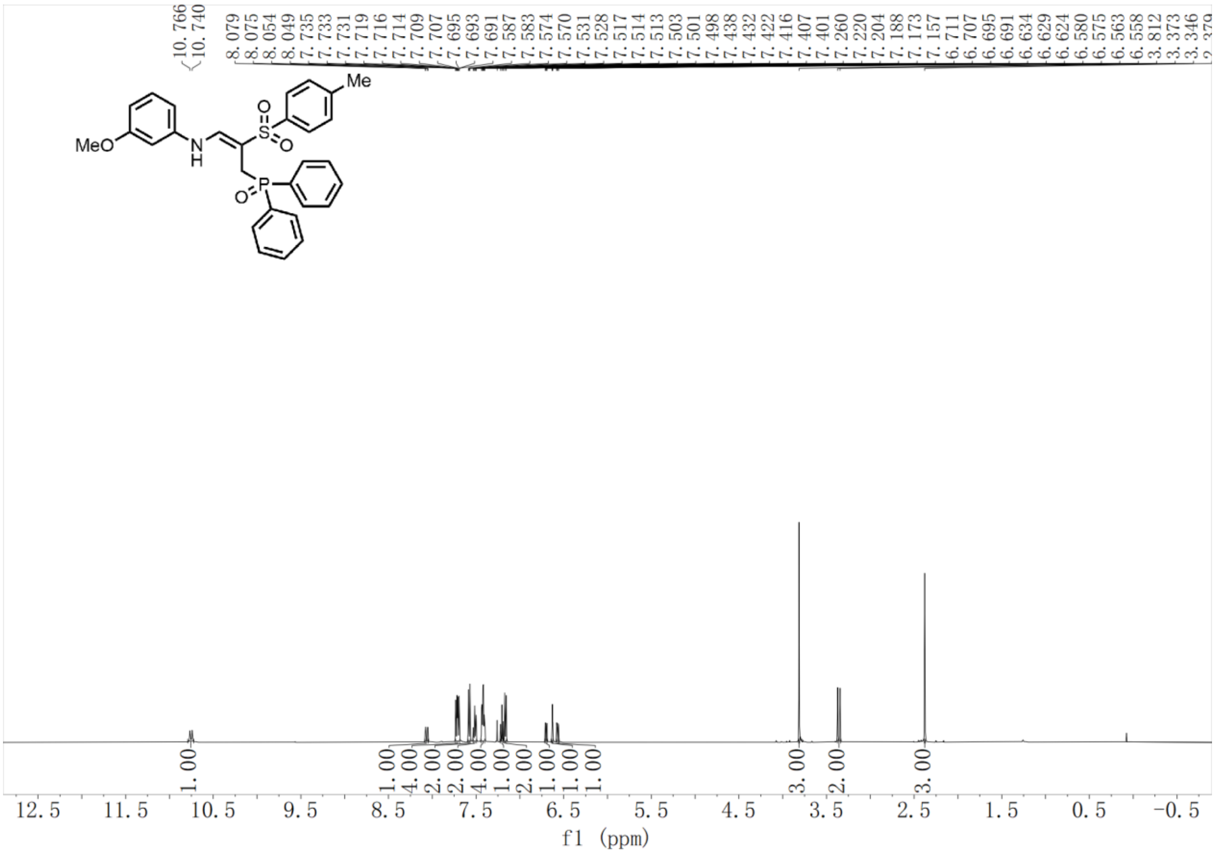

8i – ^{13}C NMR (126 MHz, $CDCl_3$)

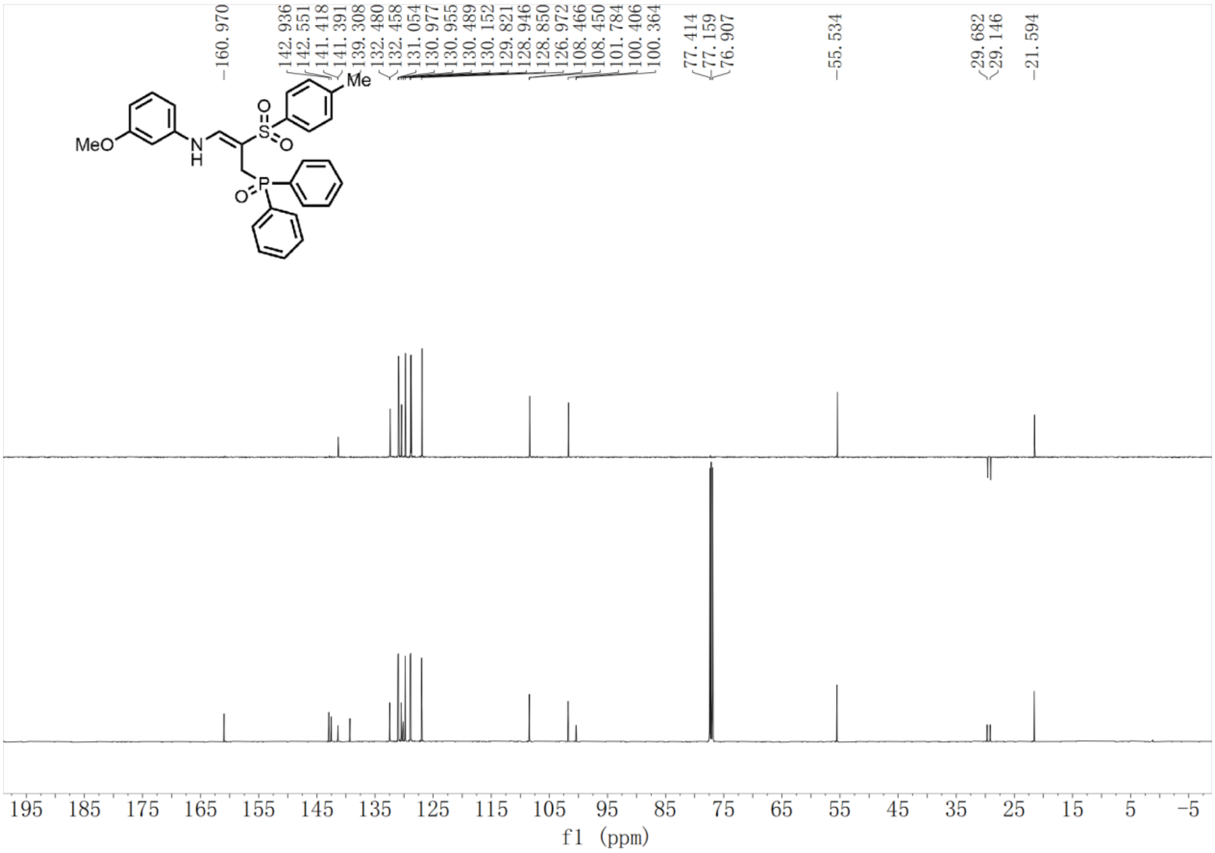

8i – ^{31}P NMR (202 MHz, $CDCl_3$)

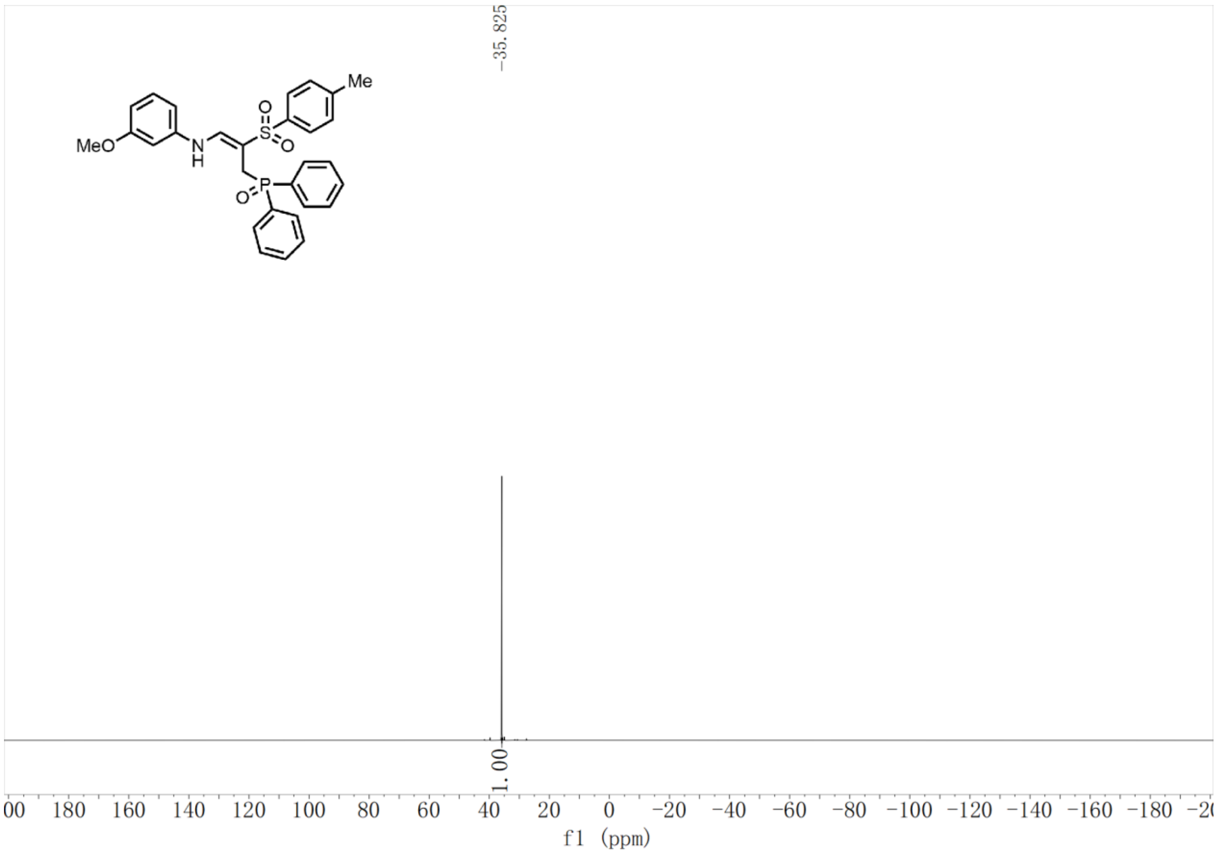

8j – ^{1}H NMR (500 MHz, $CDCl_3$)

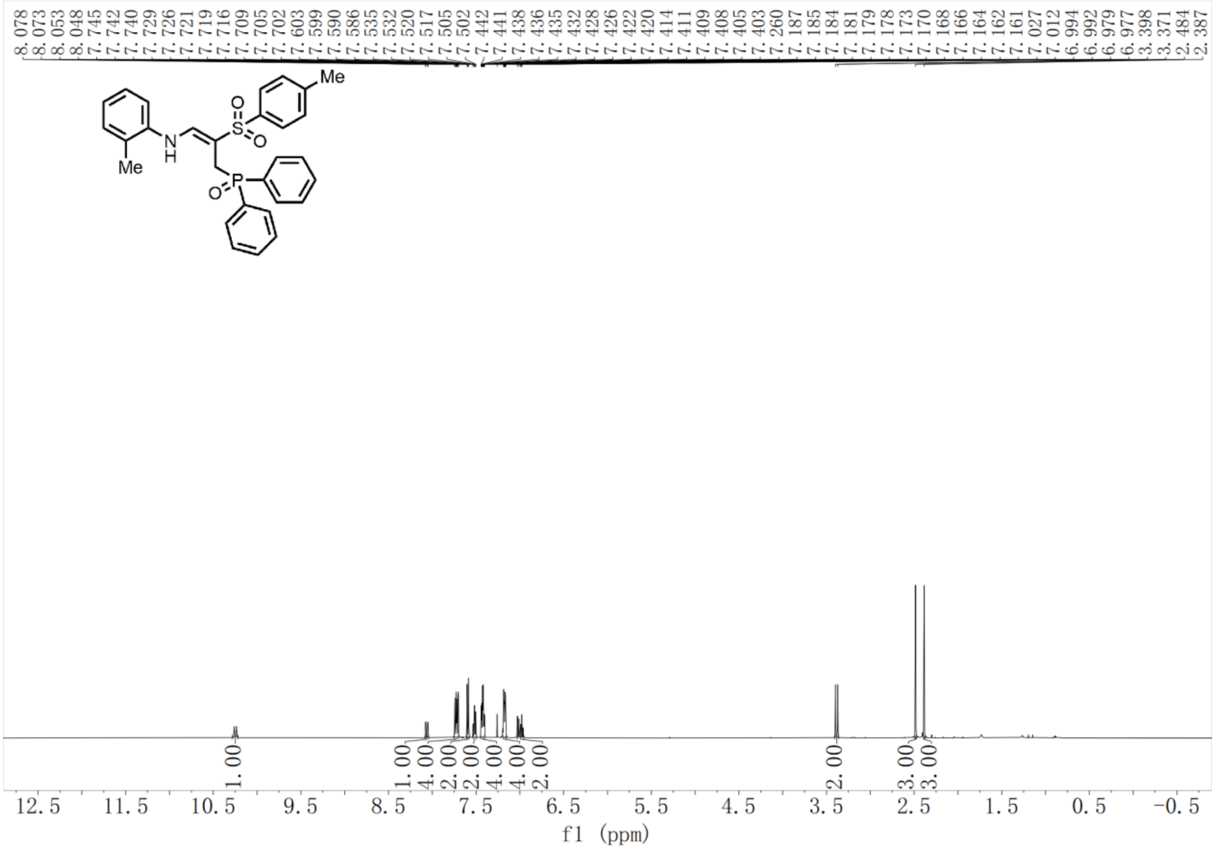

8j – ^{13}C NMR (126 MHz, $CDCl_3$)

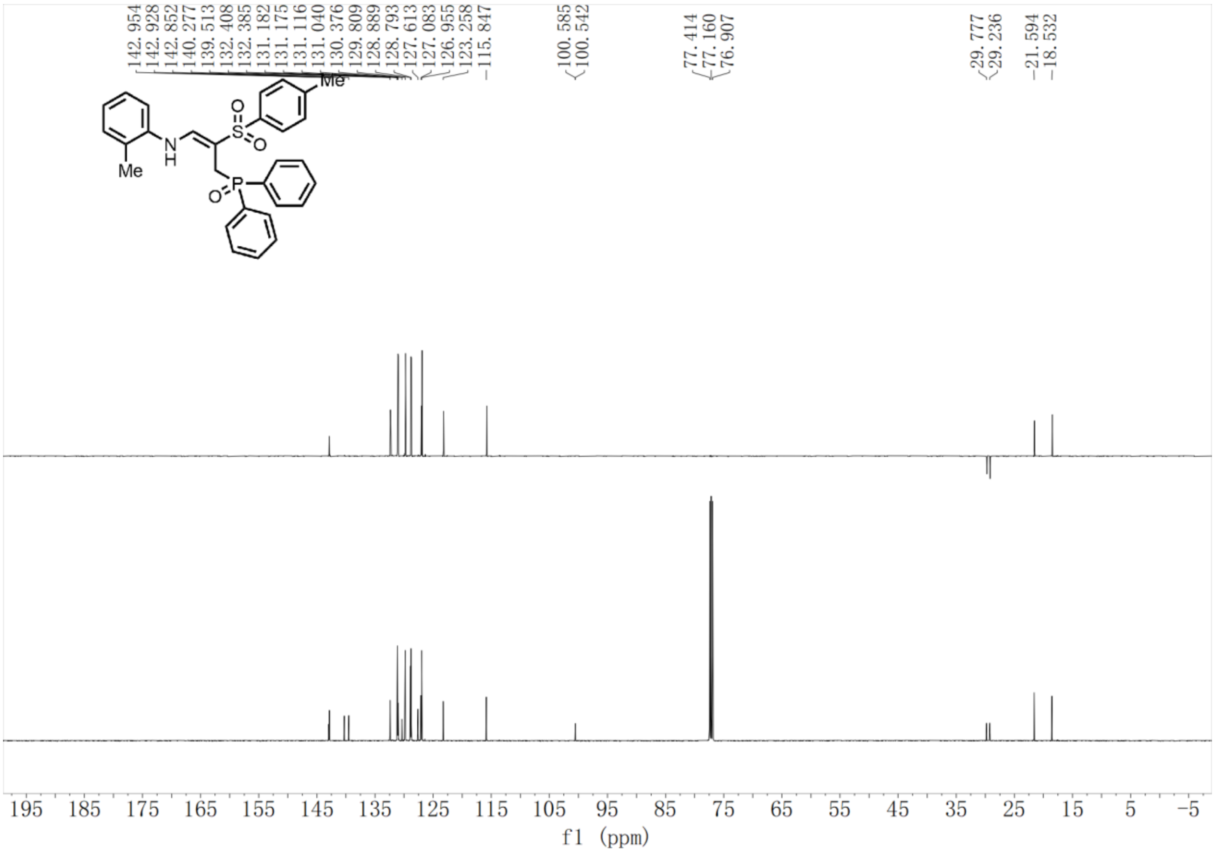

8j – ^{31}P NMR (202 MHz, $CDCl_3$)

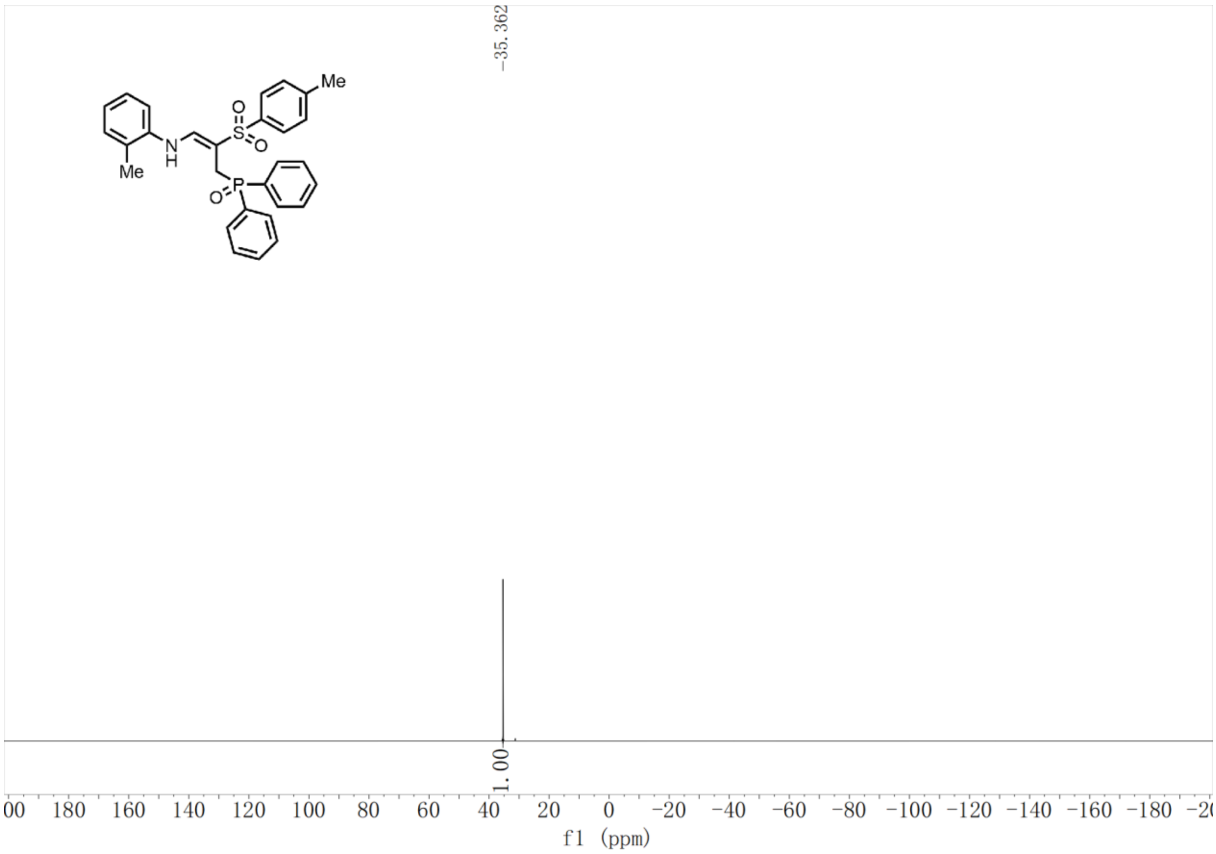

8k – ^{1}H NMR (500 MHz, CDCl$_3$)

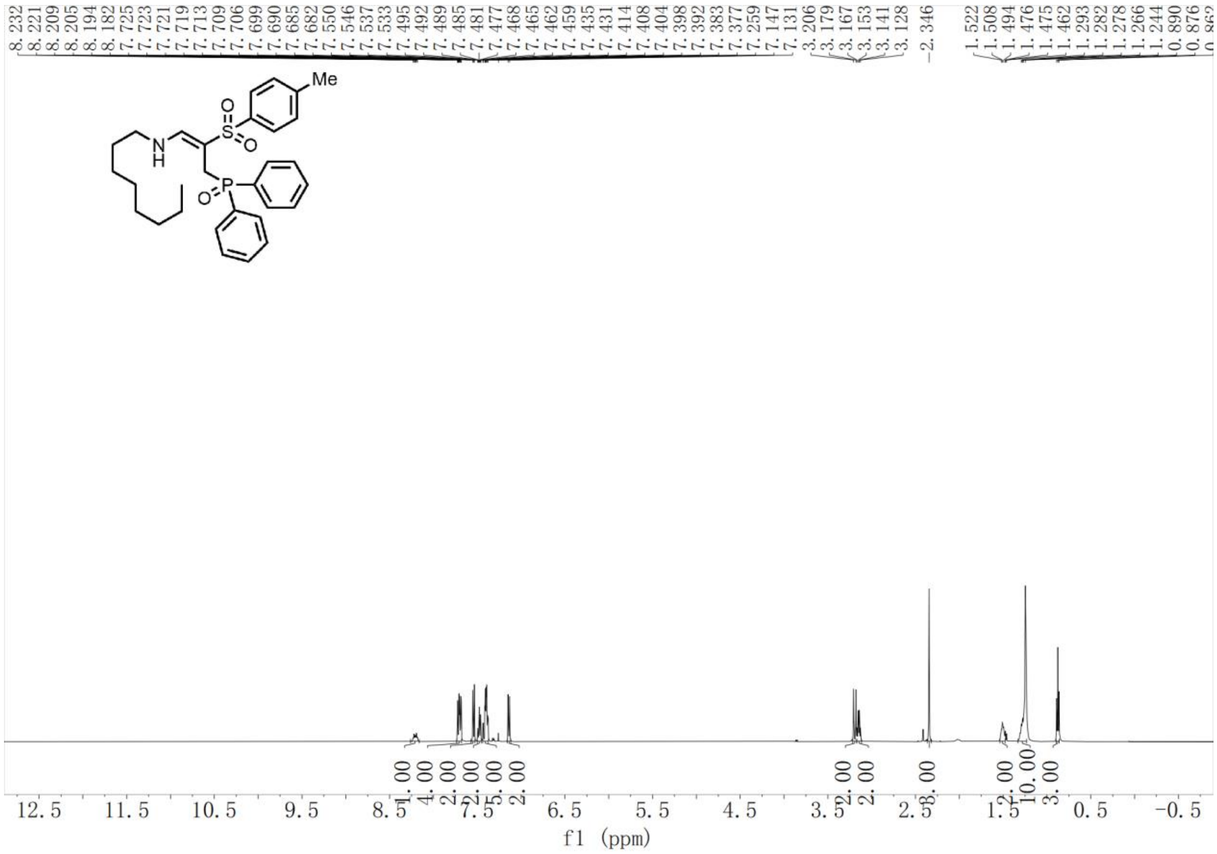

8k – ^{13}C NMR (126 MHz, CDCl$_3$)

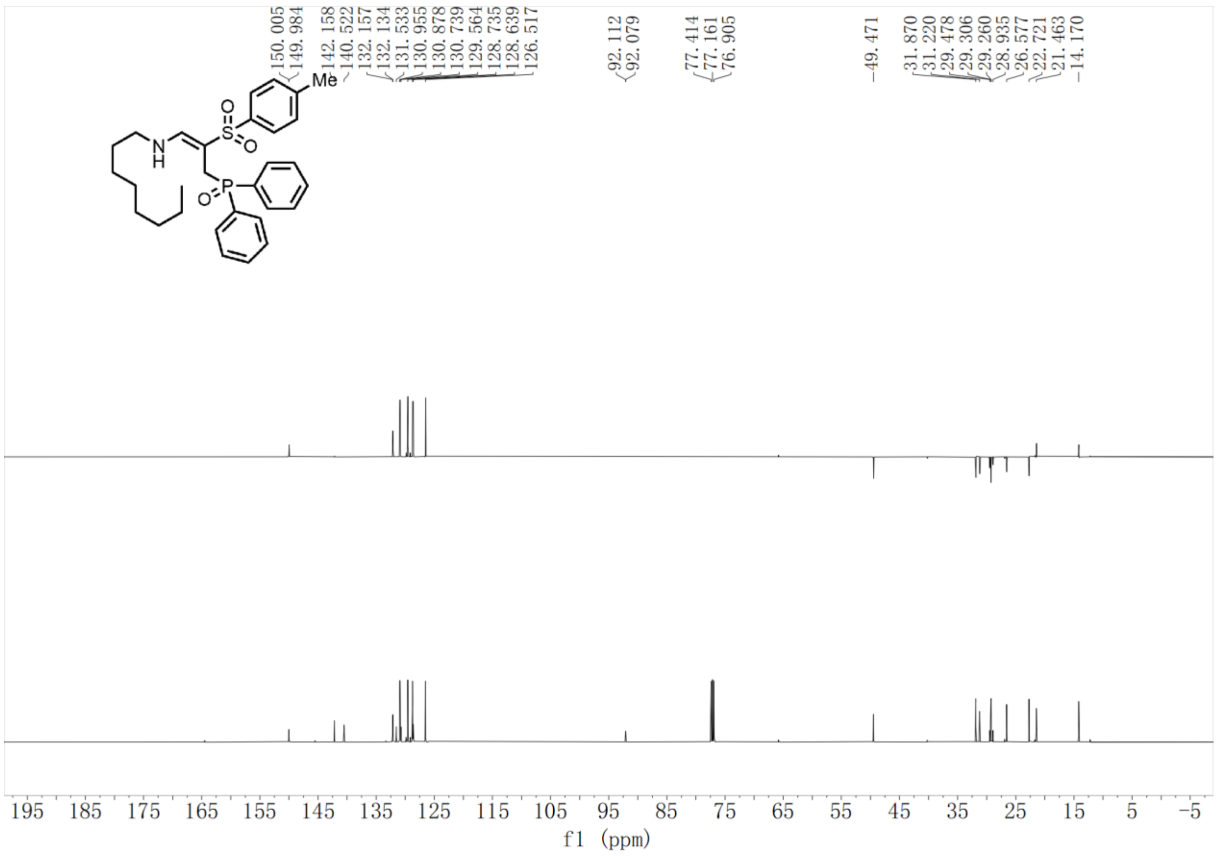

8k – ^{31}P NMR (202 MHz, $CDCl_3$)

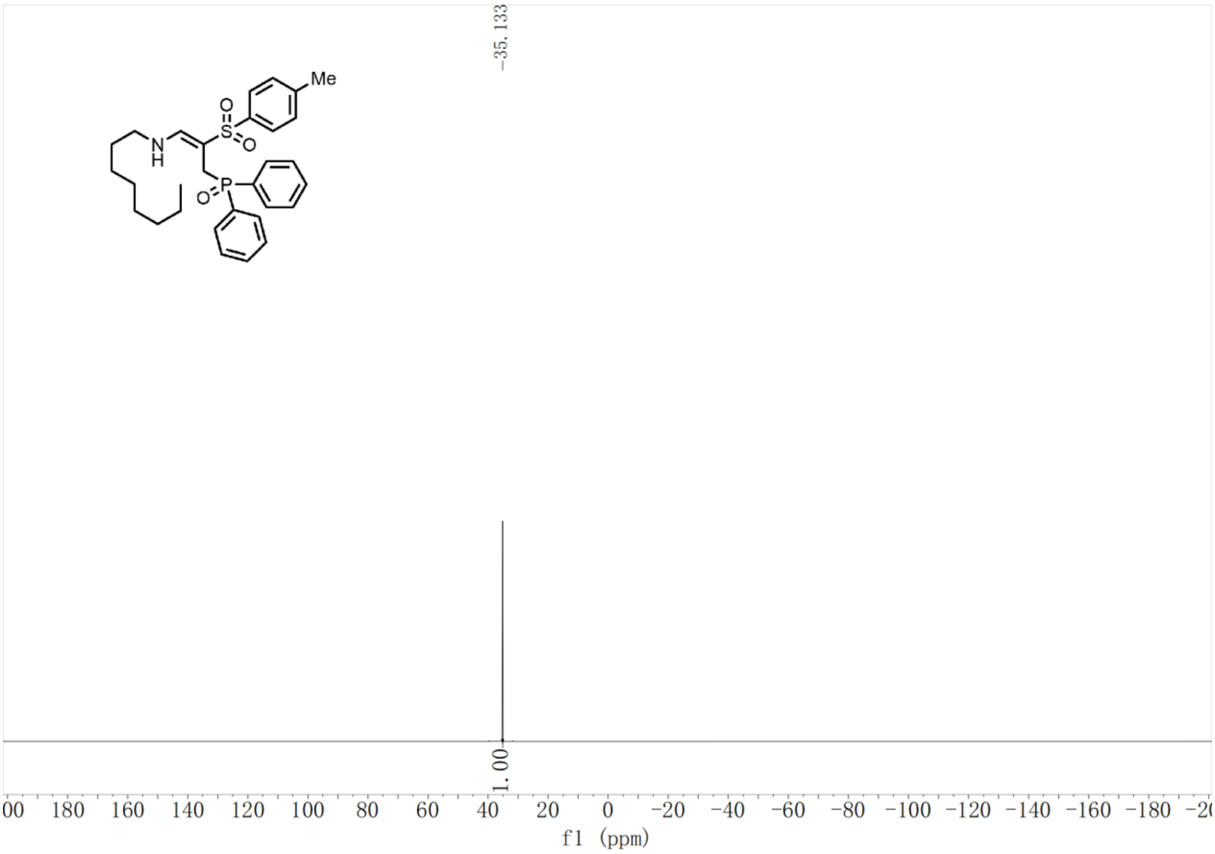

8l – ^{1}H NMR (500 MHz, $CDCl_3$)

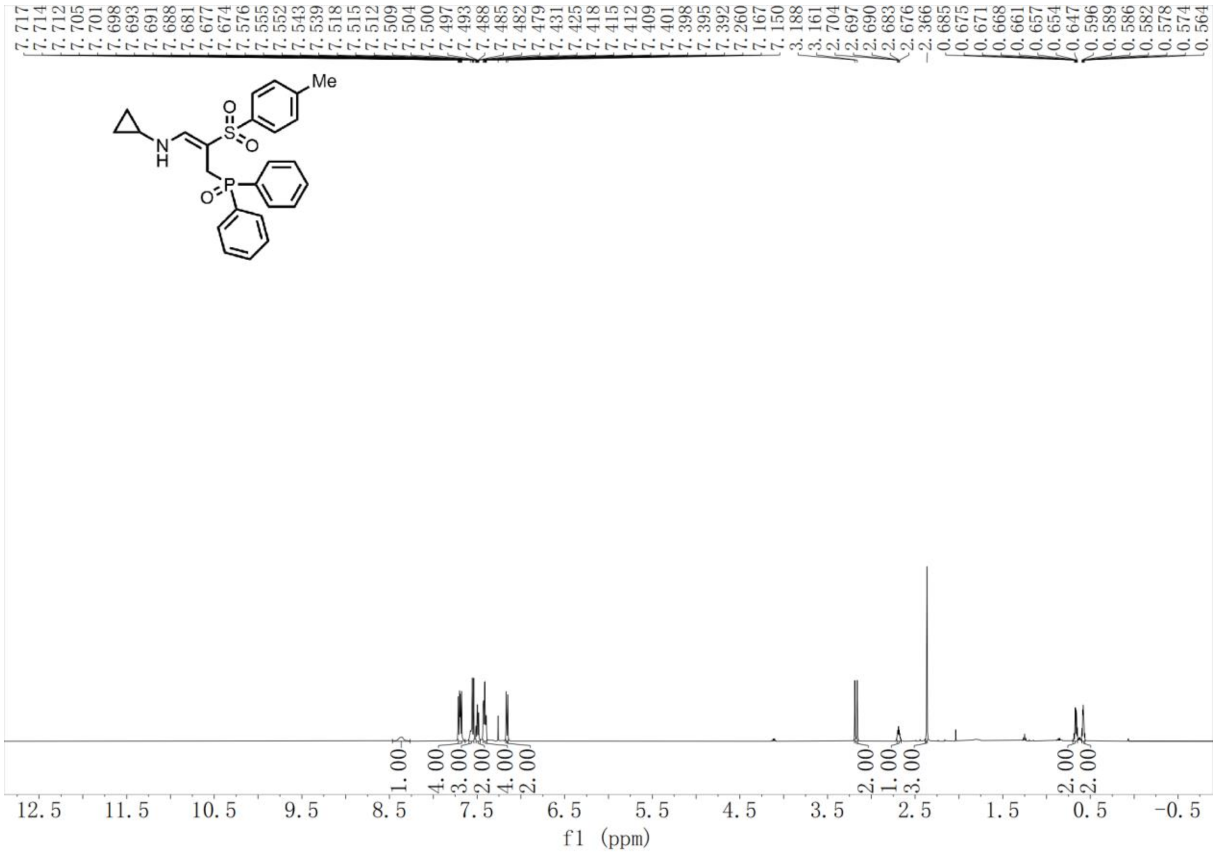

8l – ^{13}C NMR (126 MHz, $CDCl_3$)

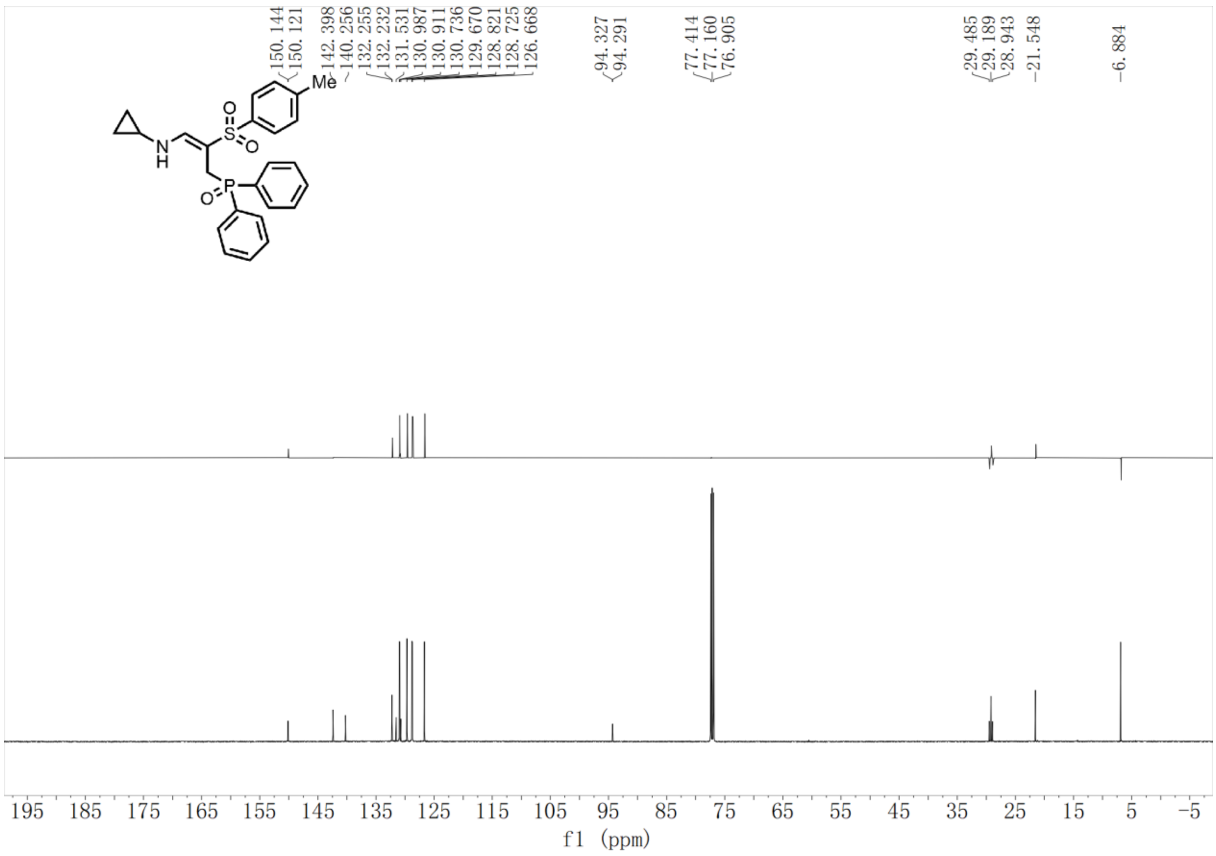

8l – ^{31}P NMR (202 MHz, $CDCl_3$)

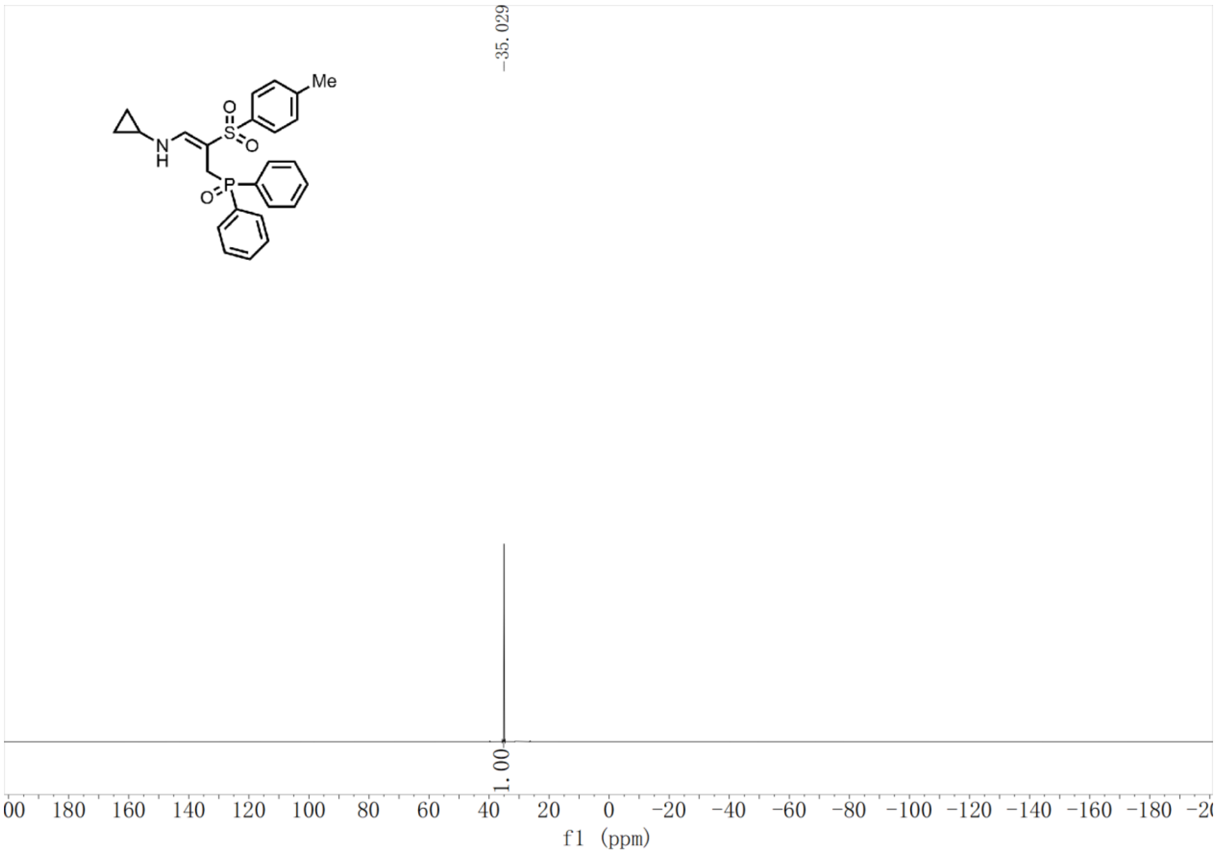

8m – ^{1}H NMR (500 MHz, $CDCl_3$)

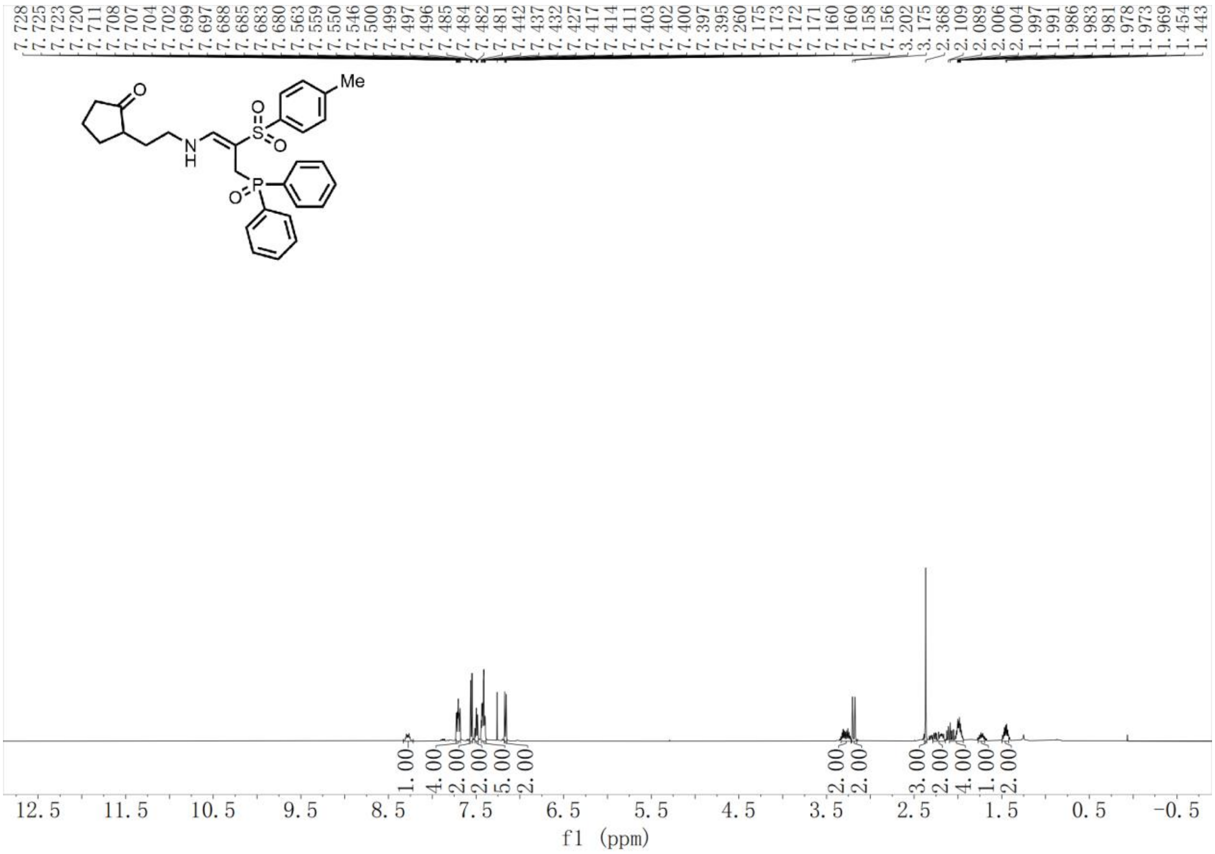

8m – ^{13}C NMR (126 MHz, $CDCl_3$)

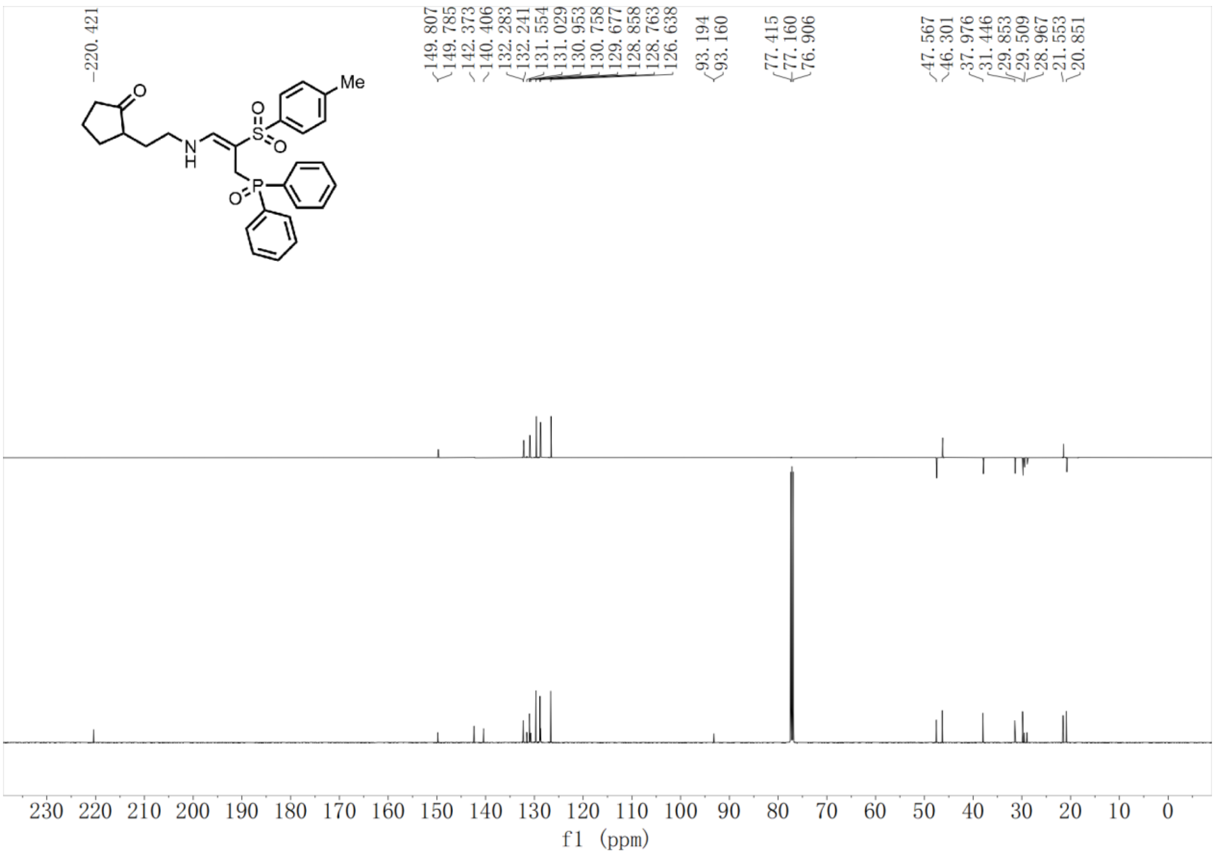

8m – ^{31}P NMR (202 MHz, $CDCl_3$)

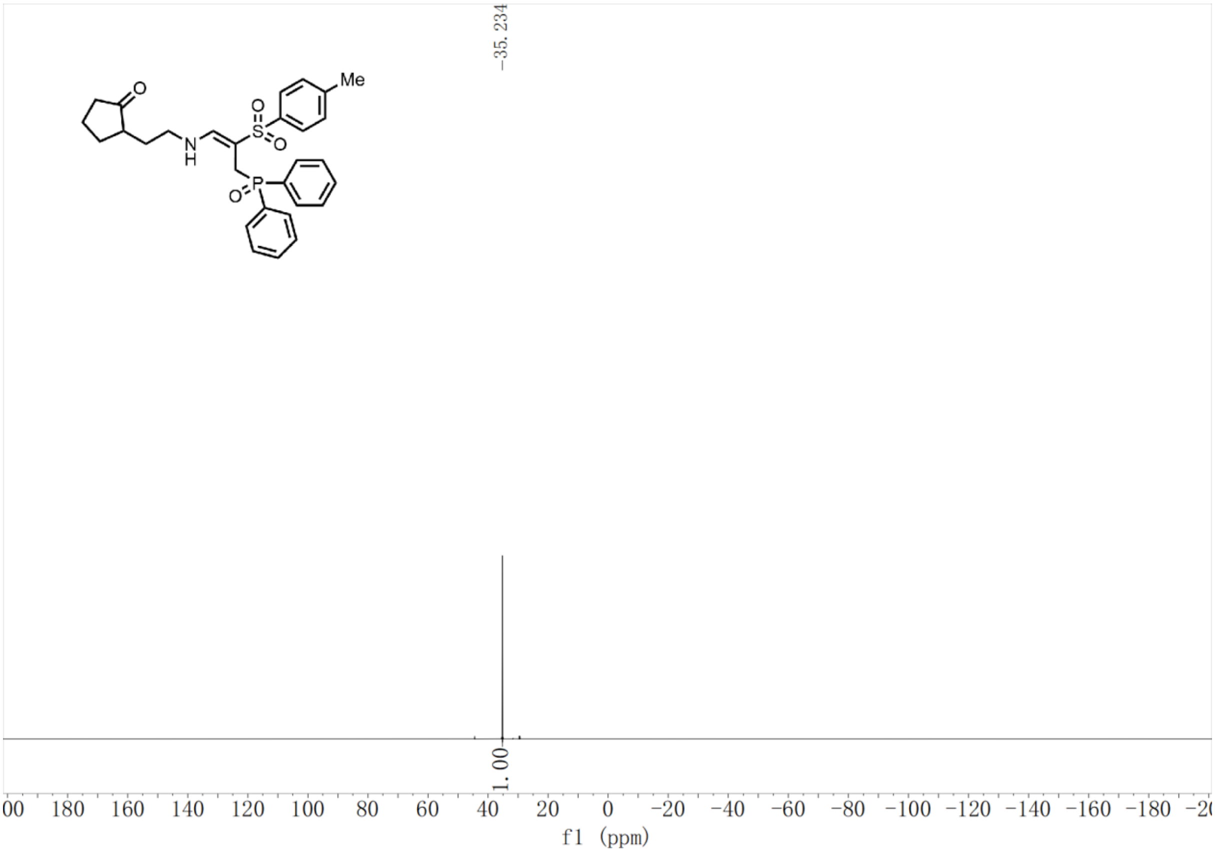

8n – ^{1}H NMR (500 MHz, $CDCl_3$)

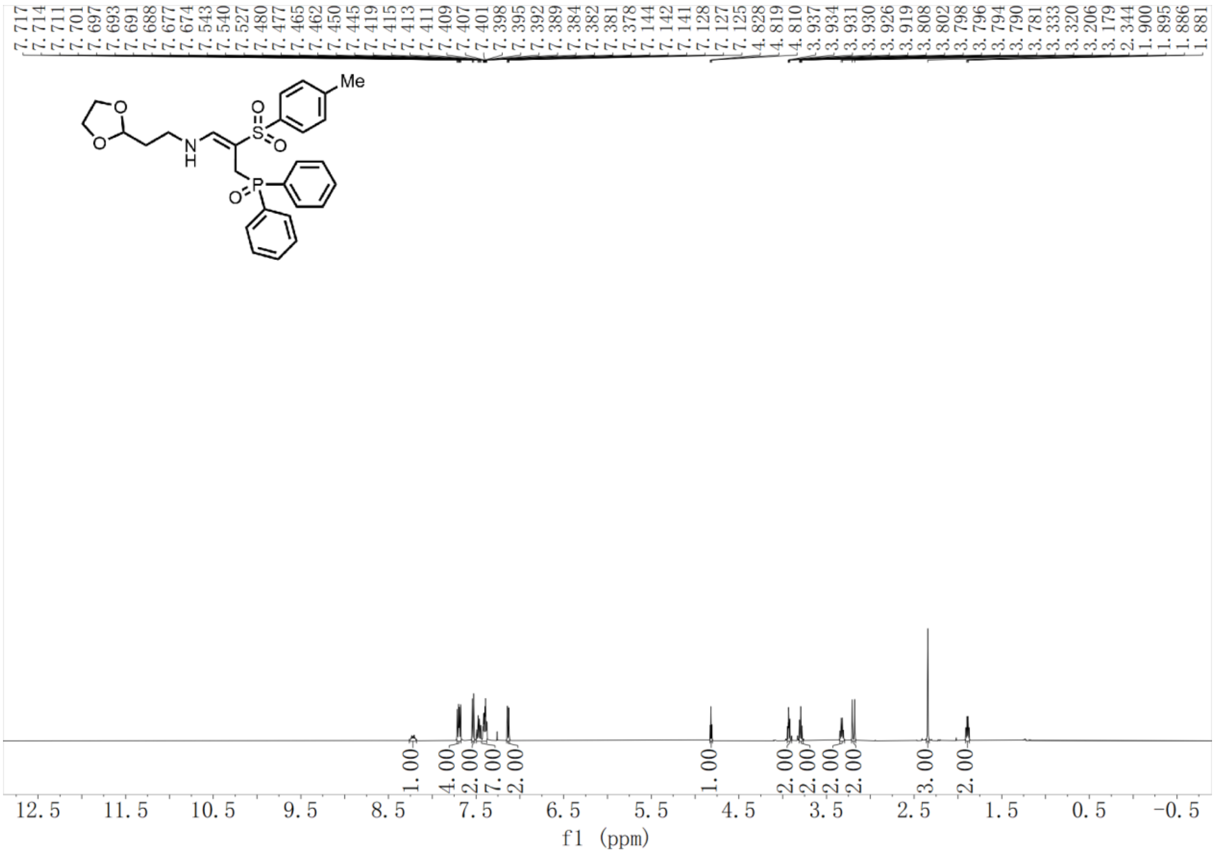

8n – ^{13}C NMR (126 MHz, $CDCl_3$)

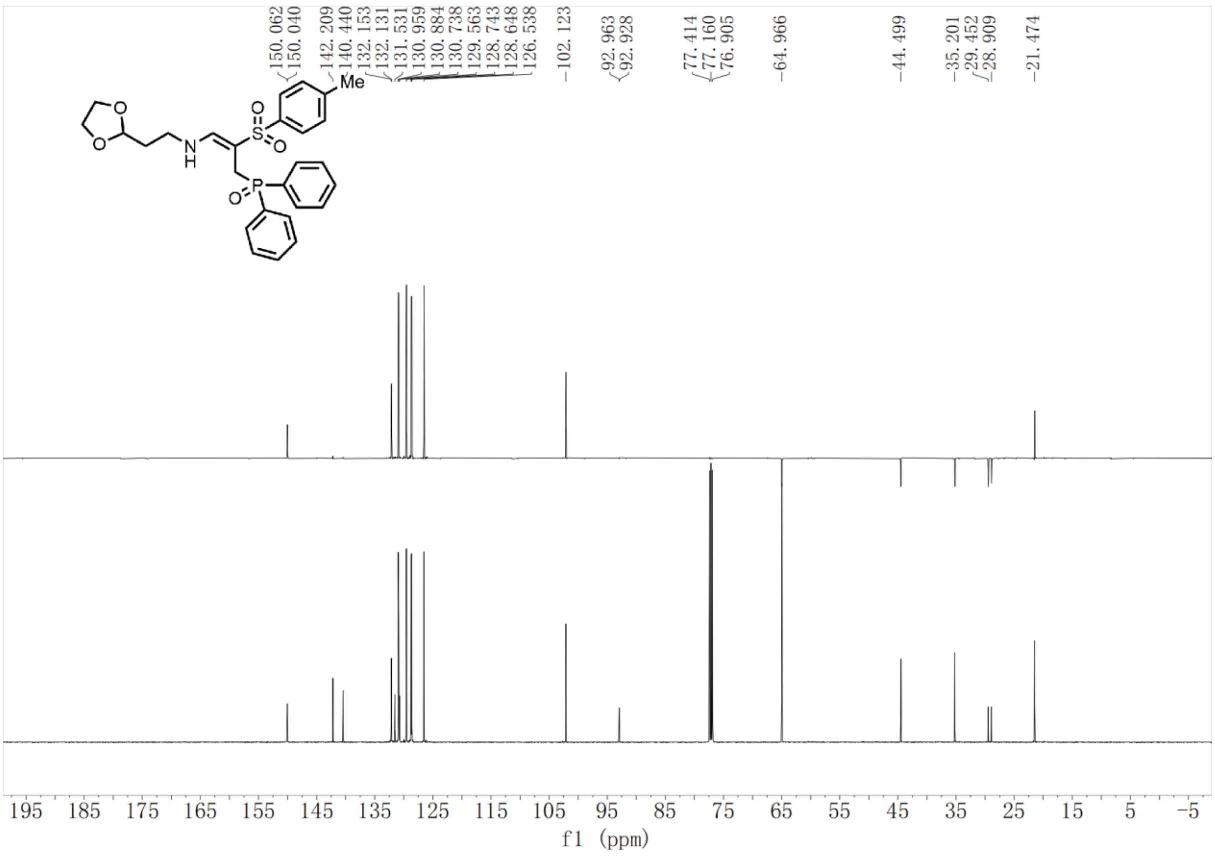

8n – ^{31}P NMR (202 MHz, $CDCl_3$)

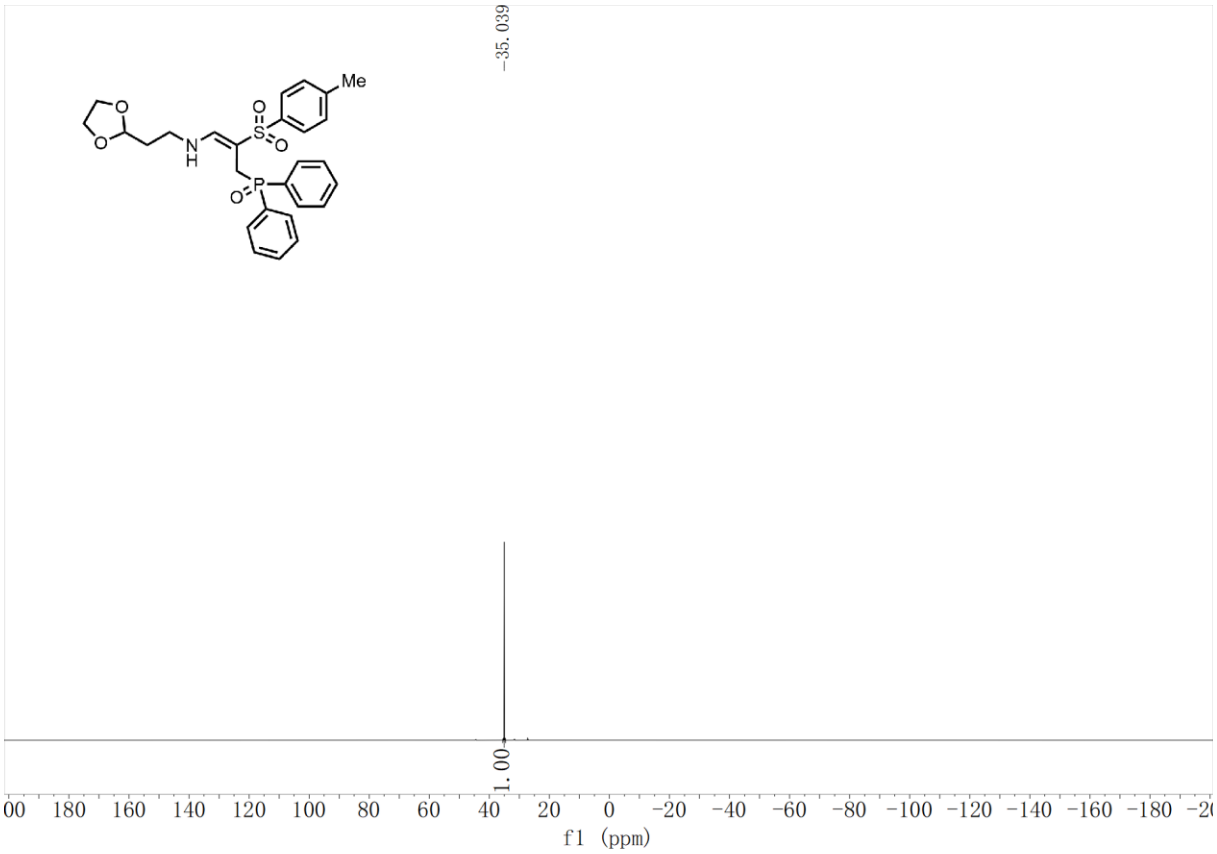

8o – ^{1}H NMR (500 MHz, $CDCl_3$)

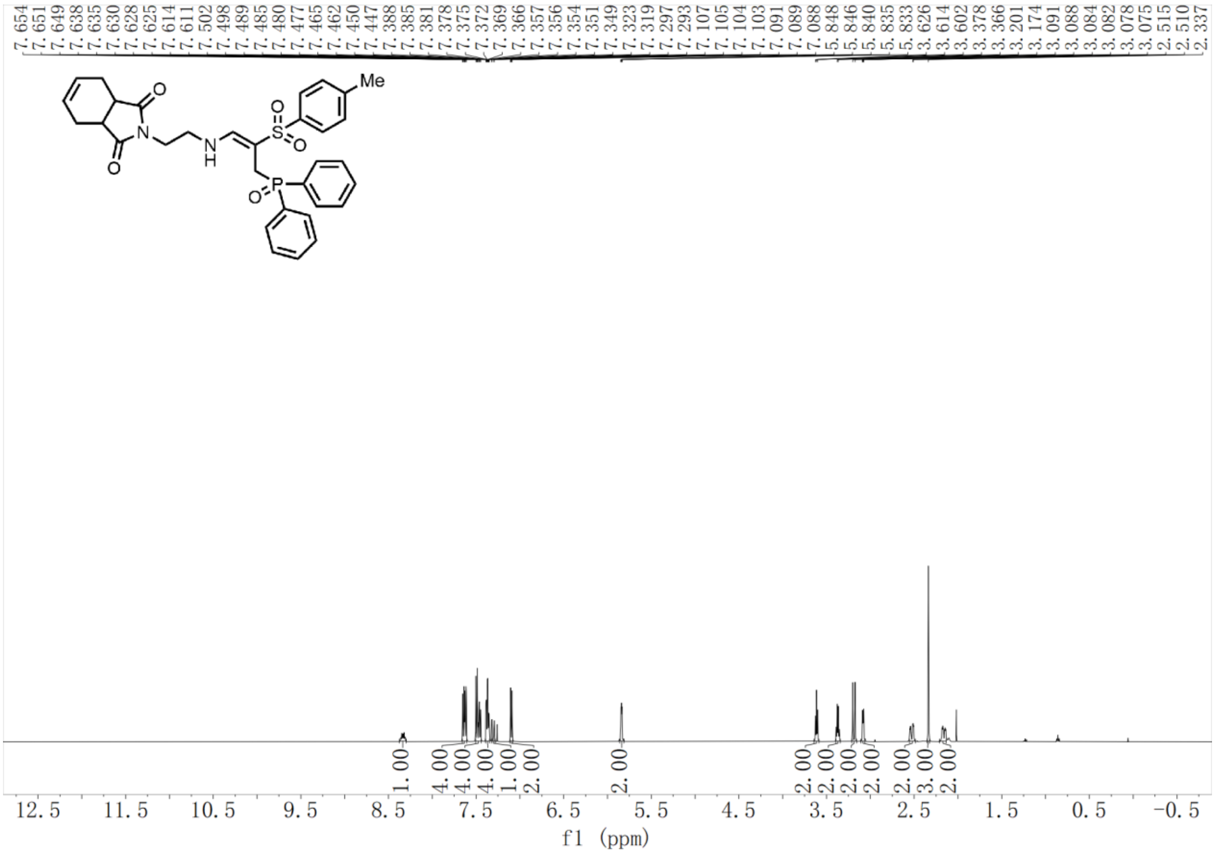

8o – ^{13}C NMR (126 MHz, $CDCl_3$)

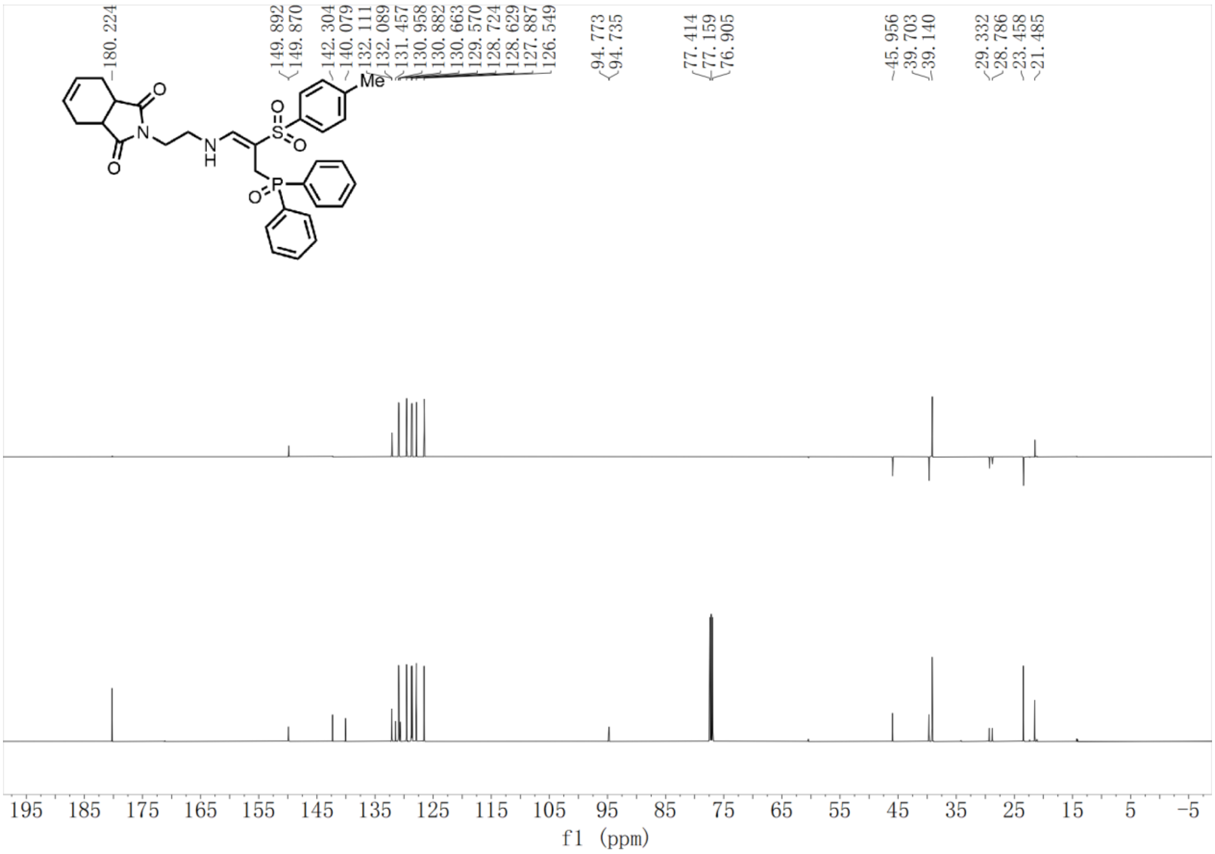

8o – ^{31}P NMR (202 MHz, $CDCl_3$)

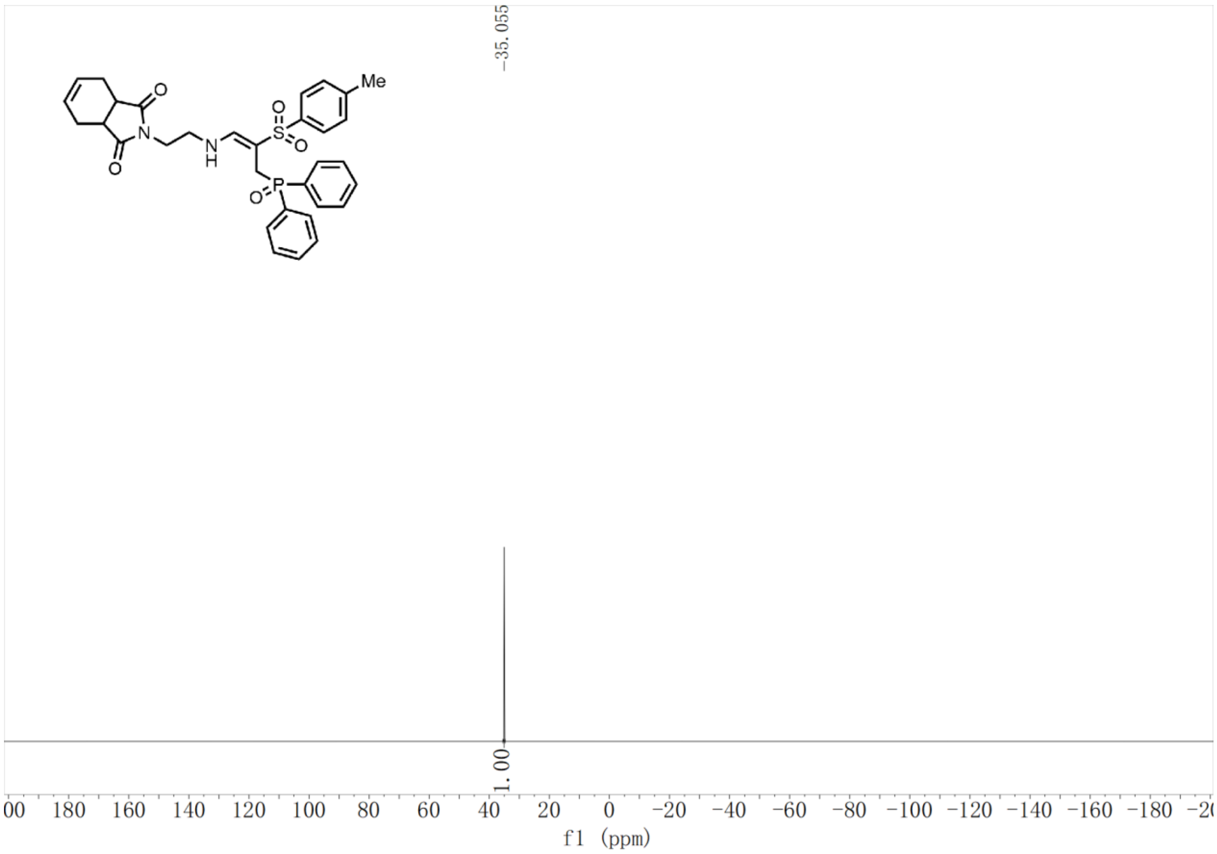

8p – ^{1}H NMR (500 MHz, $CDCl_3$)

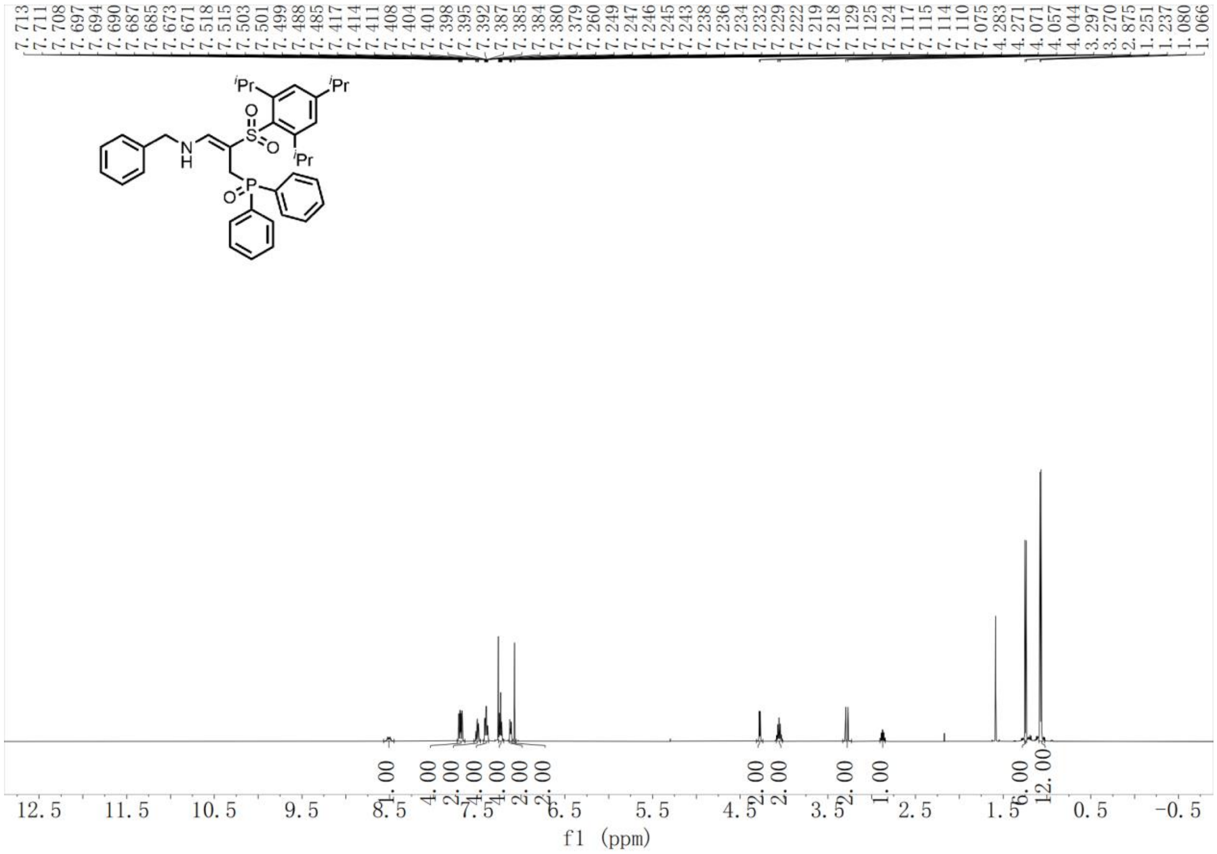

8p – ^{13}C NMR (126 MHz, $CDCl_3$)

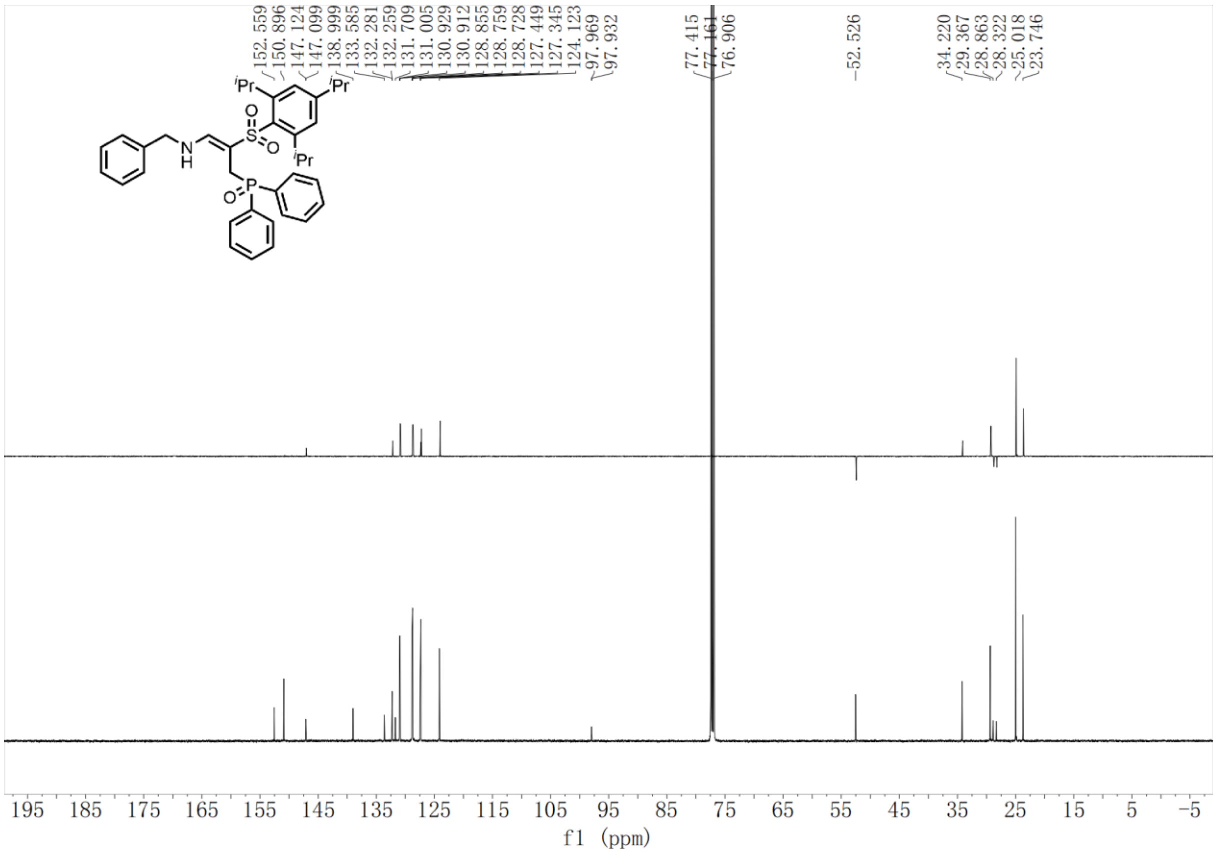

8p – ^{31}P NMR (121 MHz, $CDCl_3$)

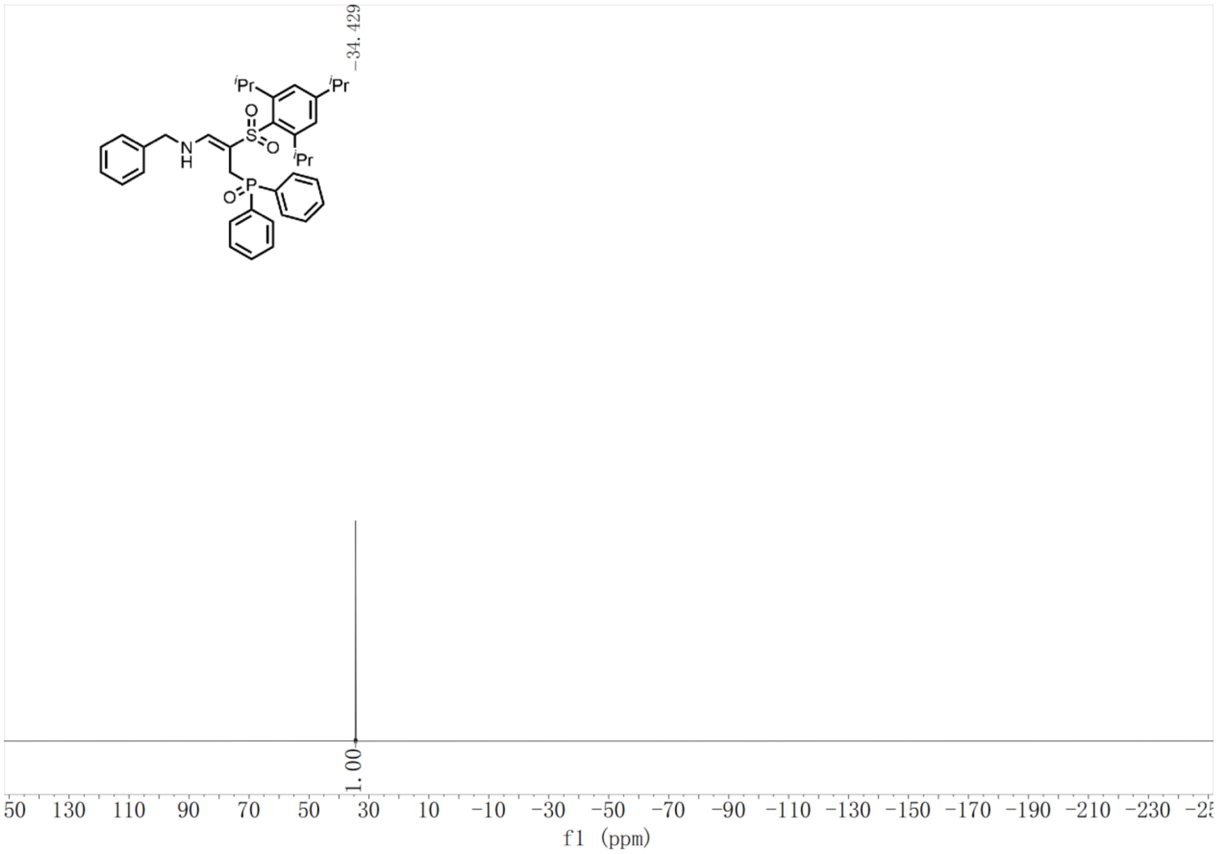

8q – ^{1}H NMR (500 MHz, $CDCl_3$)

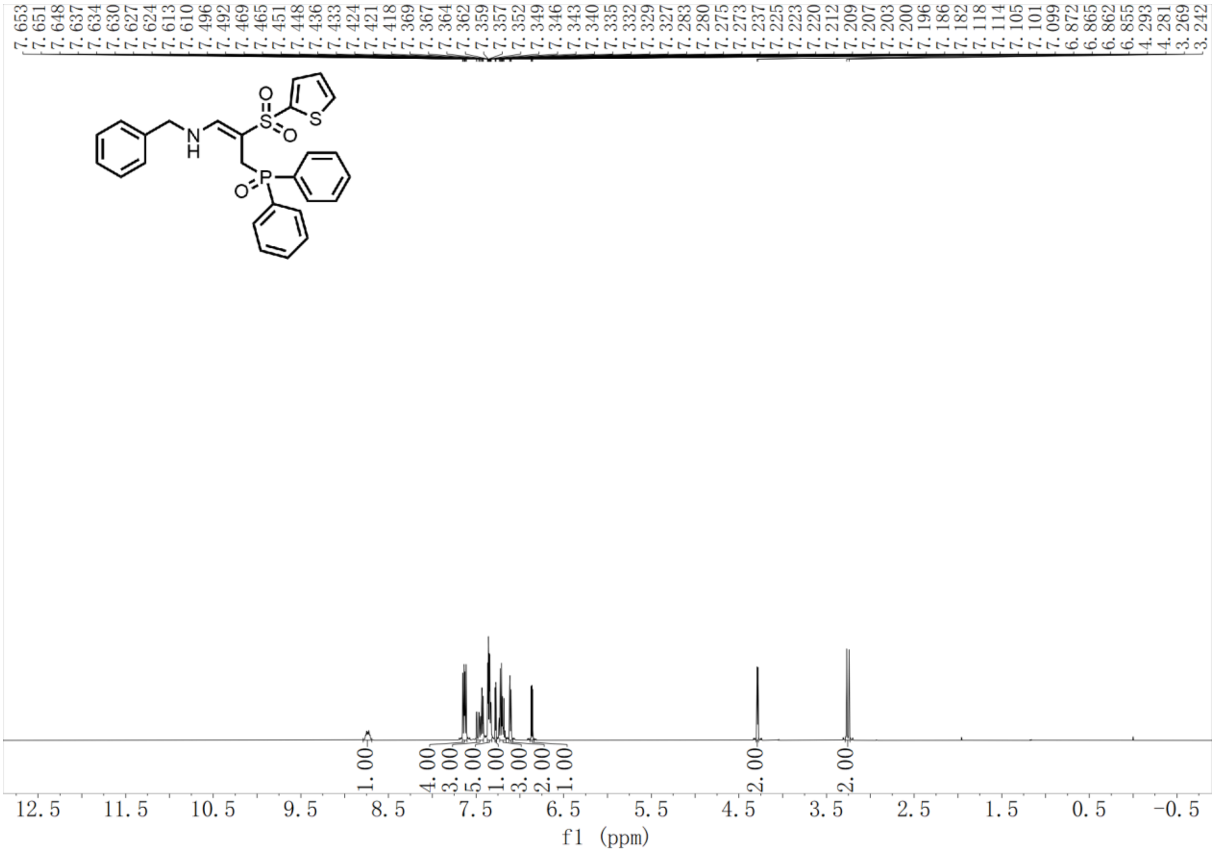

8q – ^{13}C NMR (126 MHz, $CDCl_3$)

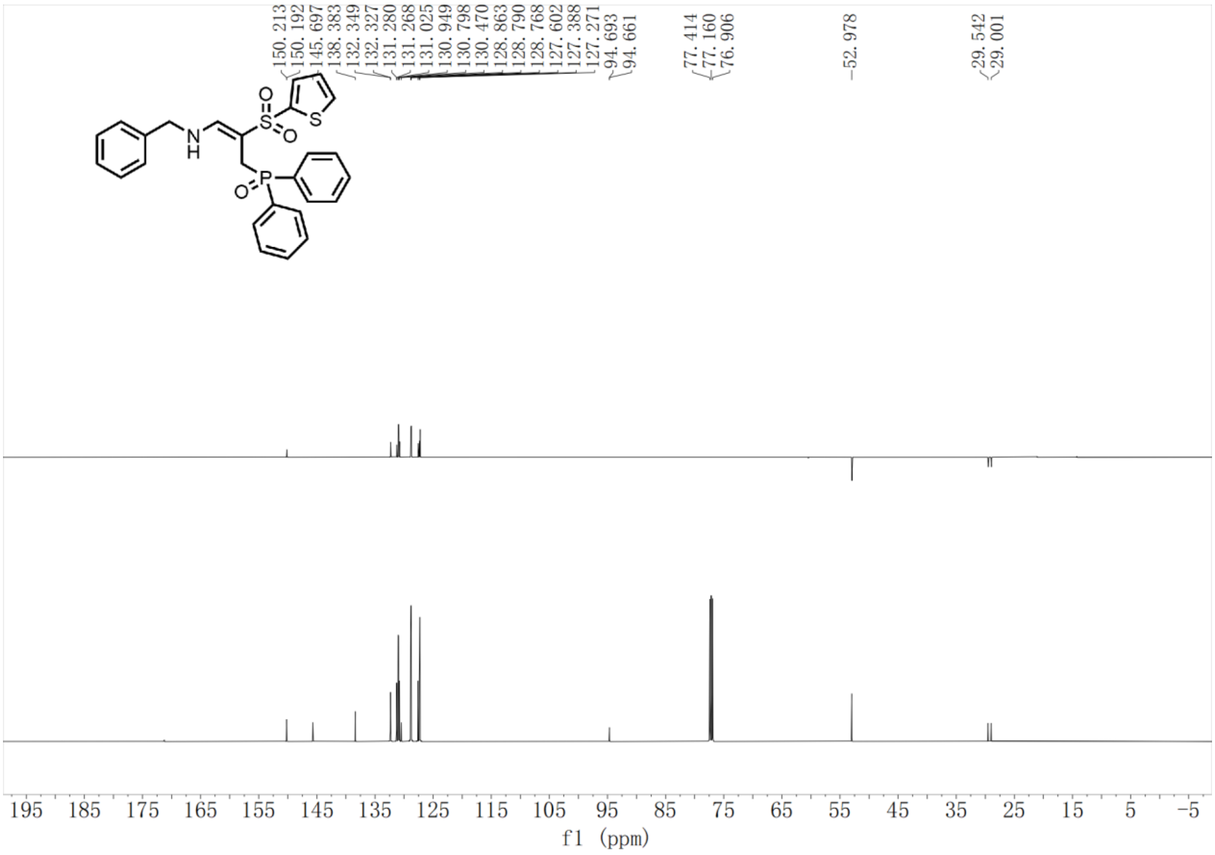

8q – ^{31}P NMR (121 MHz, $CDCl_3$)

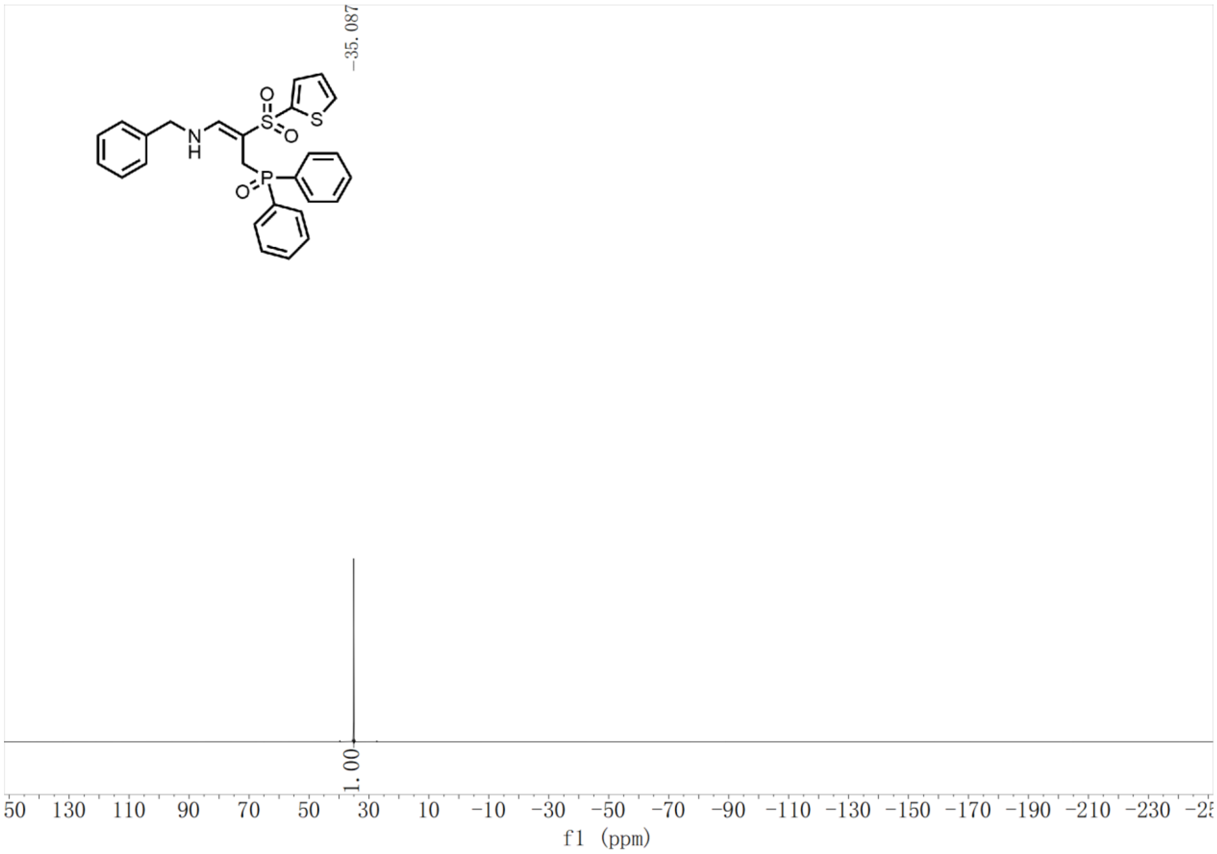

8r – ^{1}H NMR (500 MHz, $CDCl_3$)

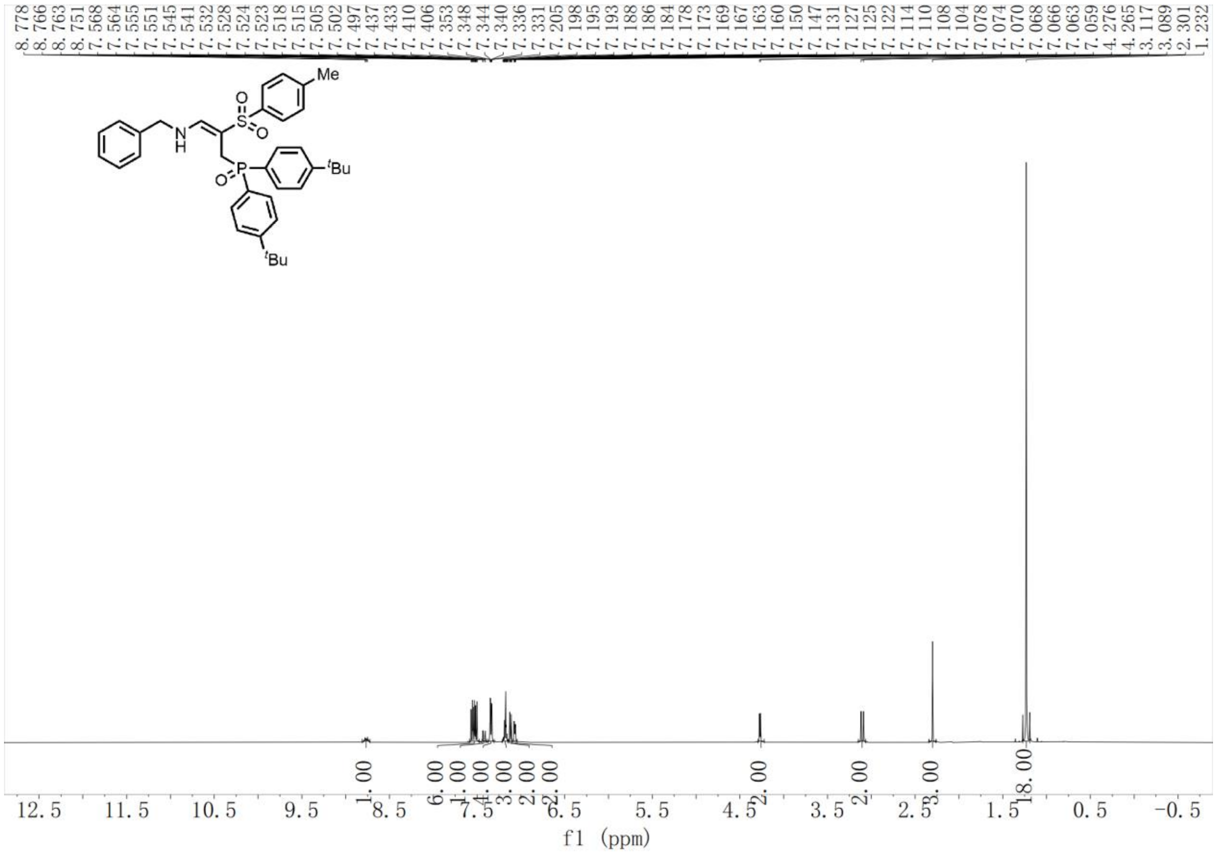

8r – ^{13}C NMR (126 MHz, $CDCl_3$)

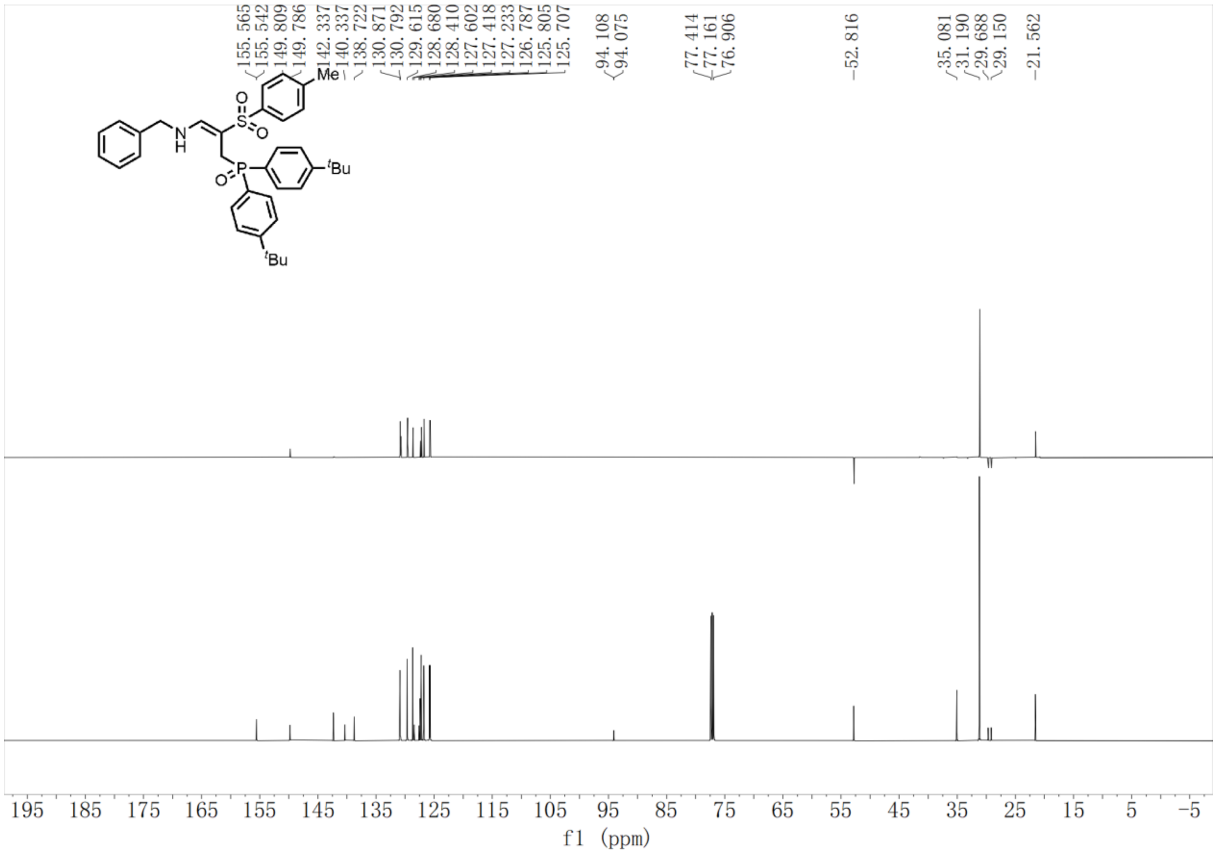

8r – ^{31}P NMR (121 MHz, $CDCl_3$)

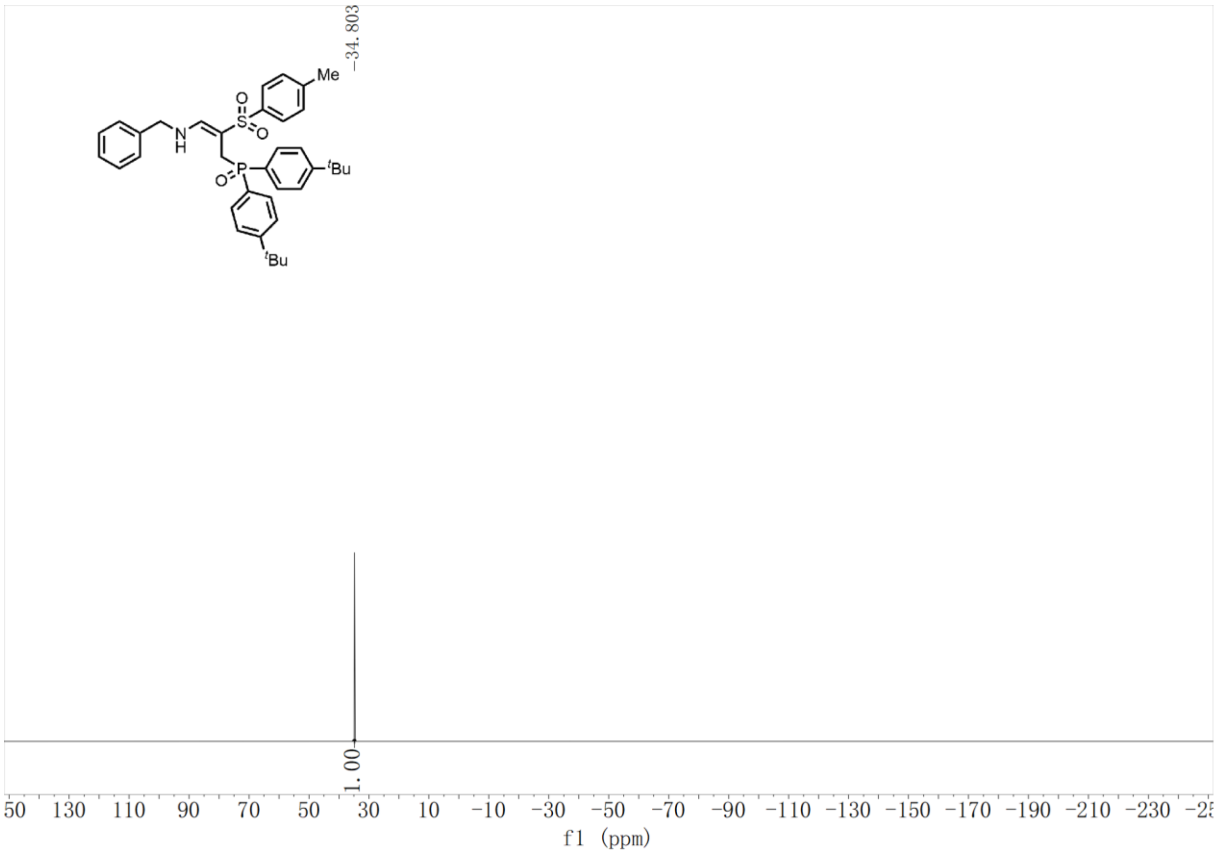

8s – ^{1}H NMR (500 MHz, $CDCl_3$)

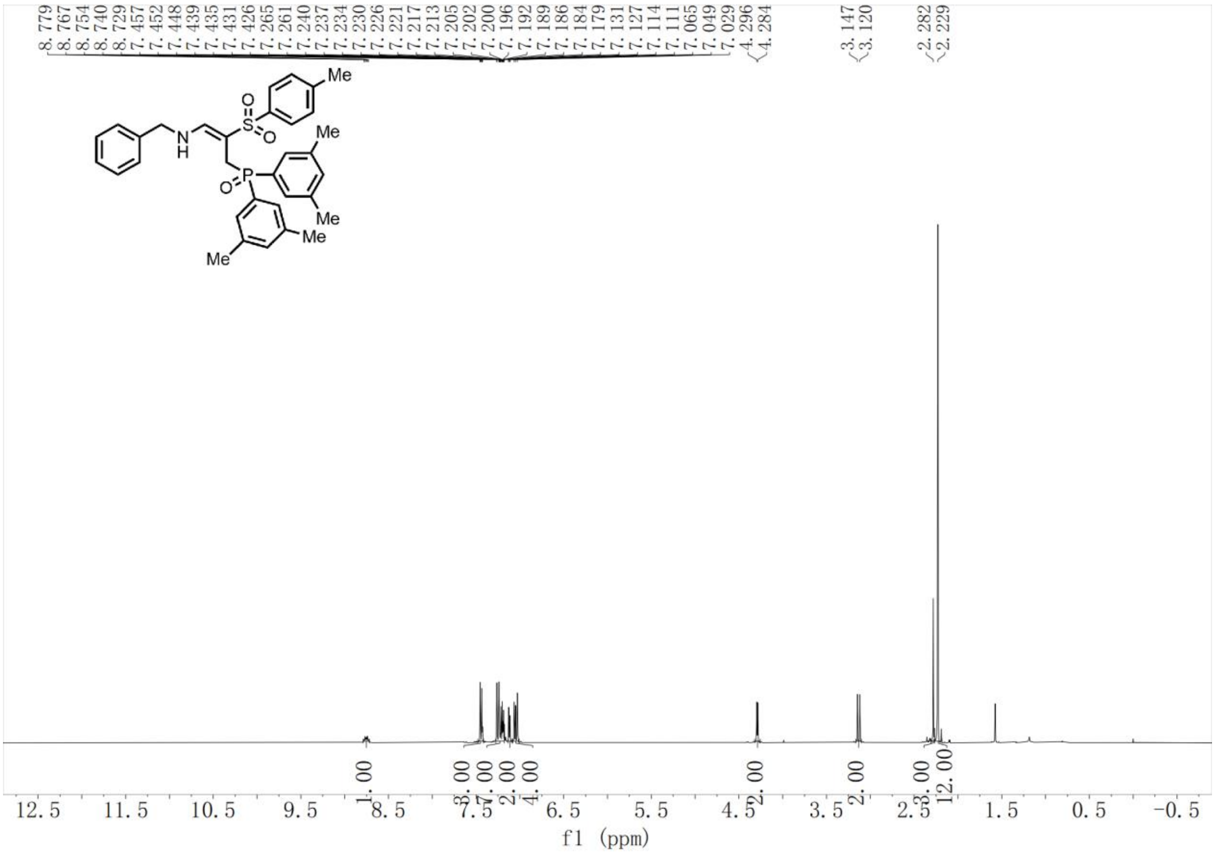

8s – ^{13}C NMR (126 MHz, $CDCl_3$)

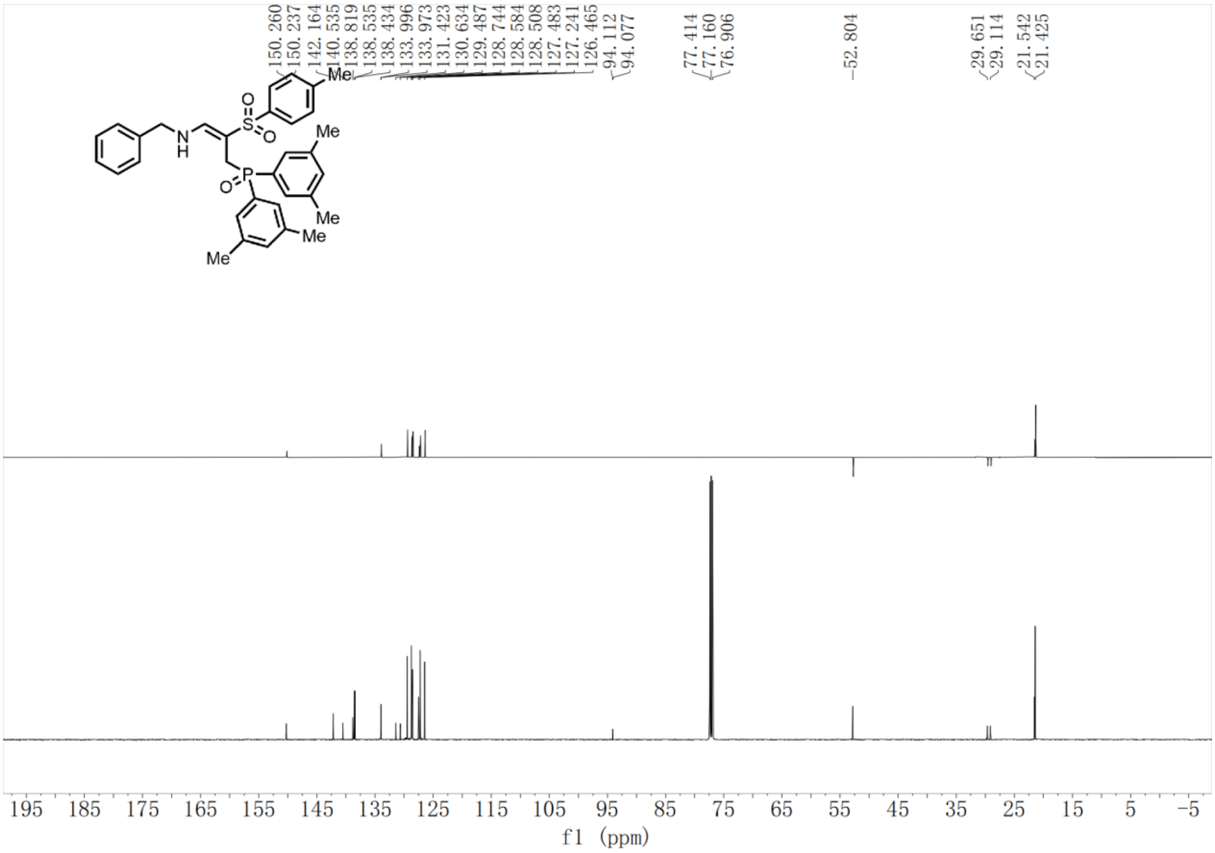

8s – ^{31}P NMR (121 MHz, $CDCl_3$)

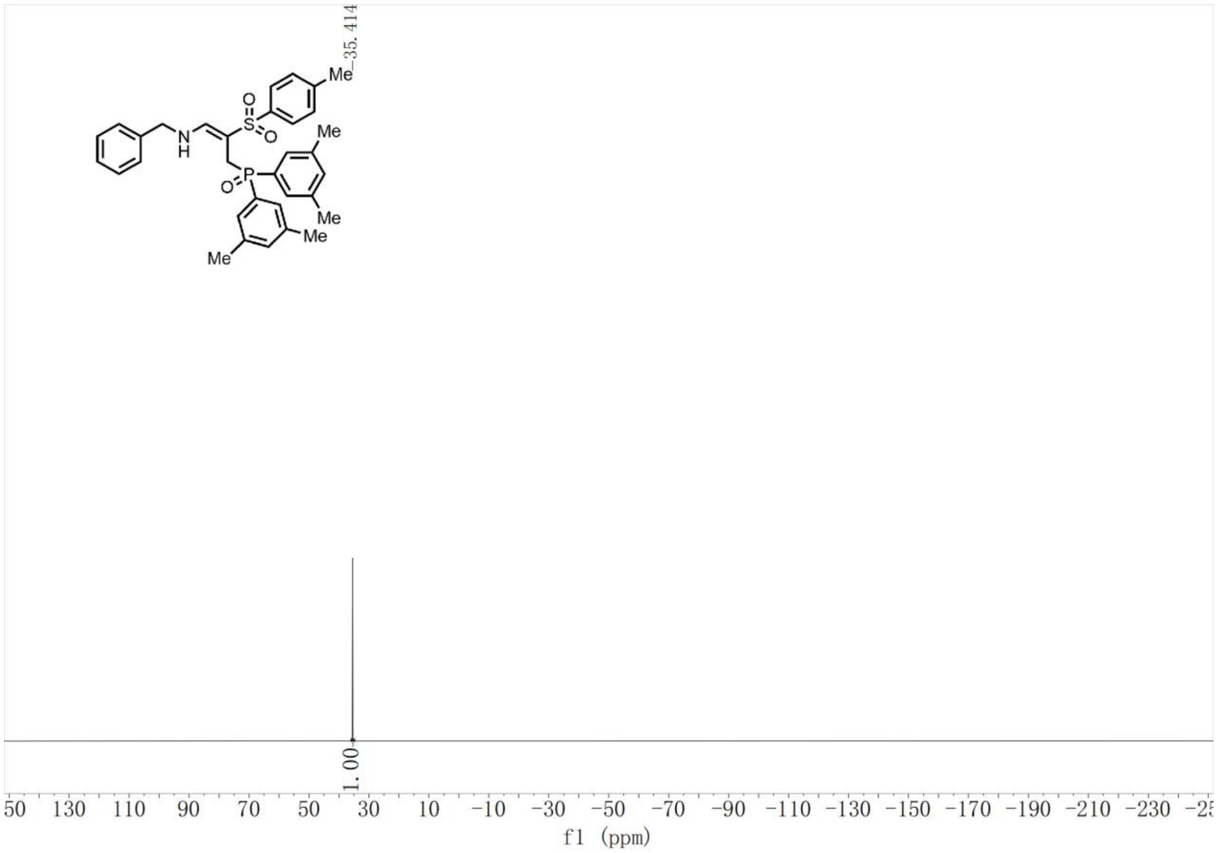

8t – ^{1}H NMR (500 MHz, $CDCl_3$)

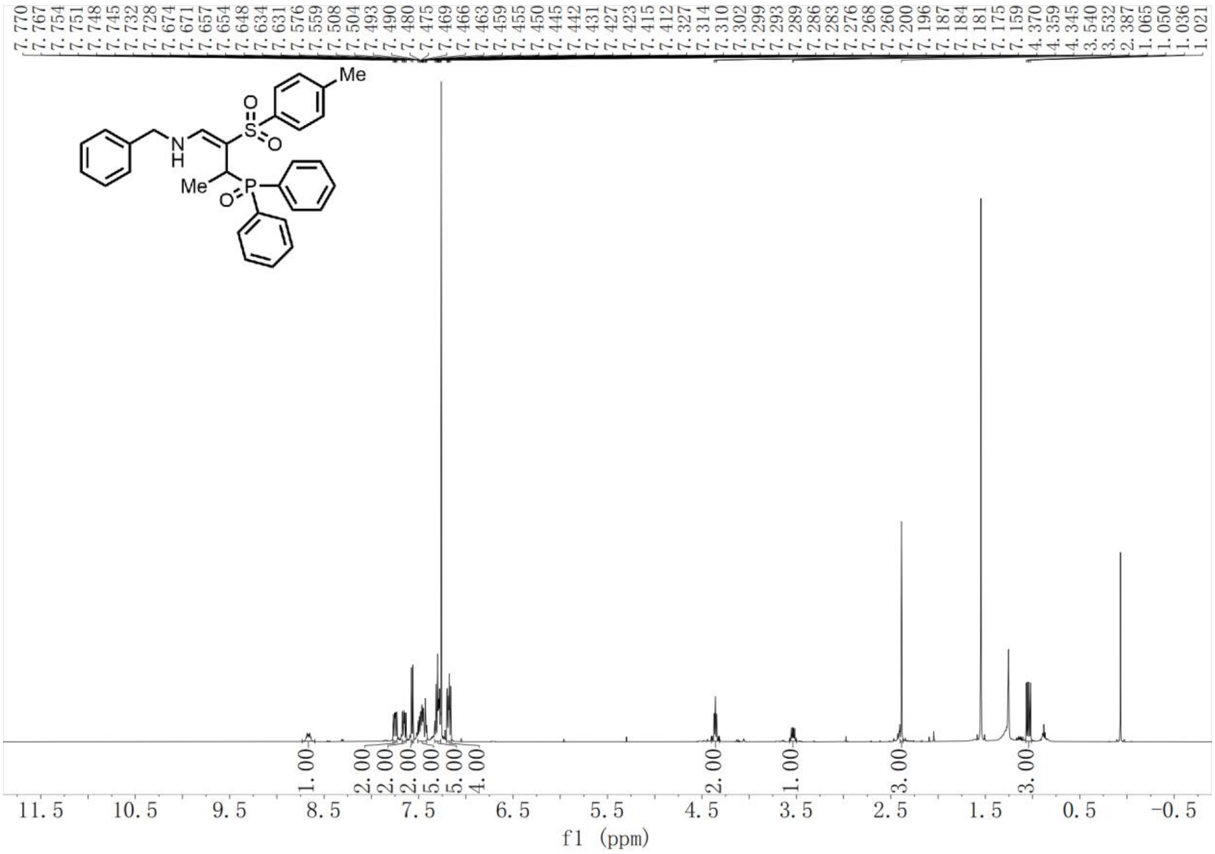

8t – ^{13}C NMR (126 MHz, $CDCl_3$)

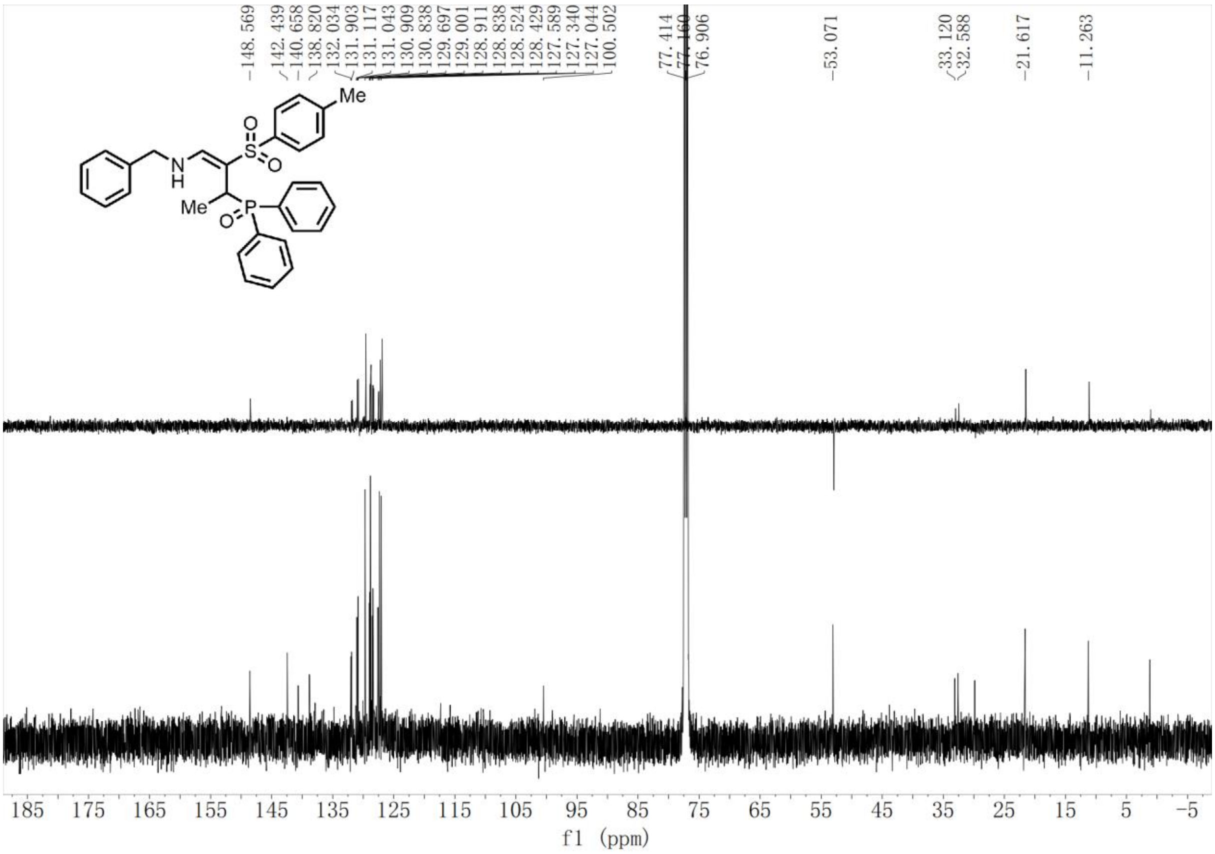

8t – ^{31}P NMR (162 MHz, $CDCl_3$)

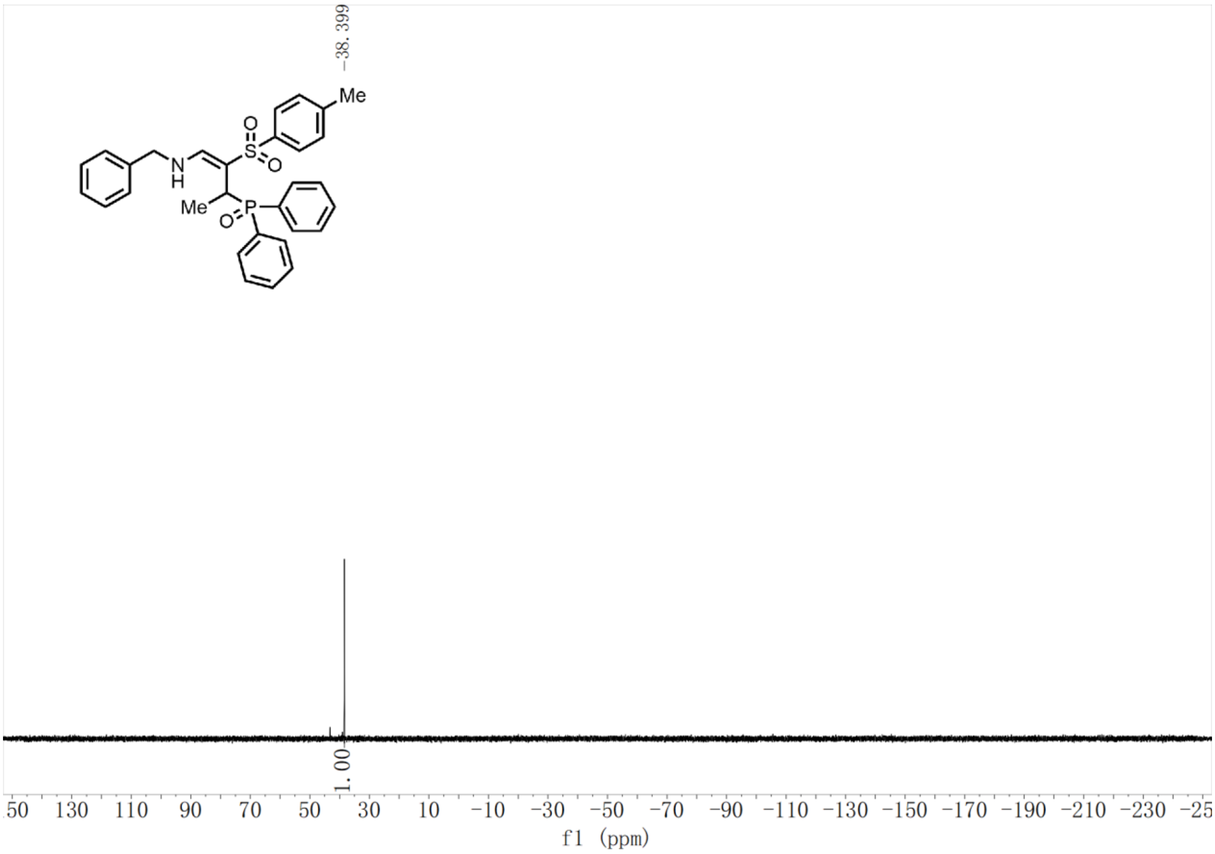

8u – ^{1}H NMR (500 MHz, $CDCl_3$)

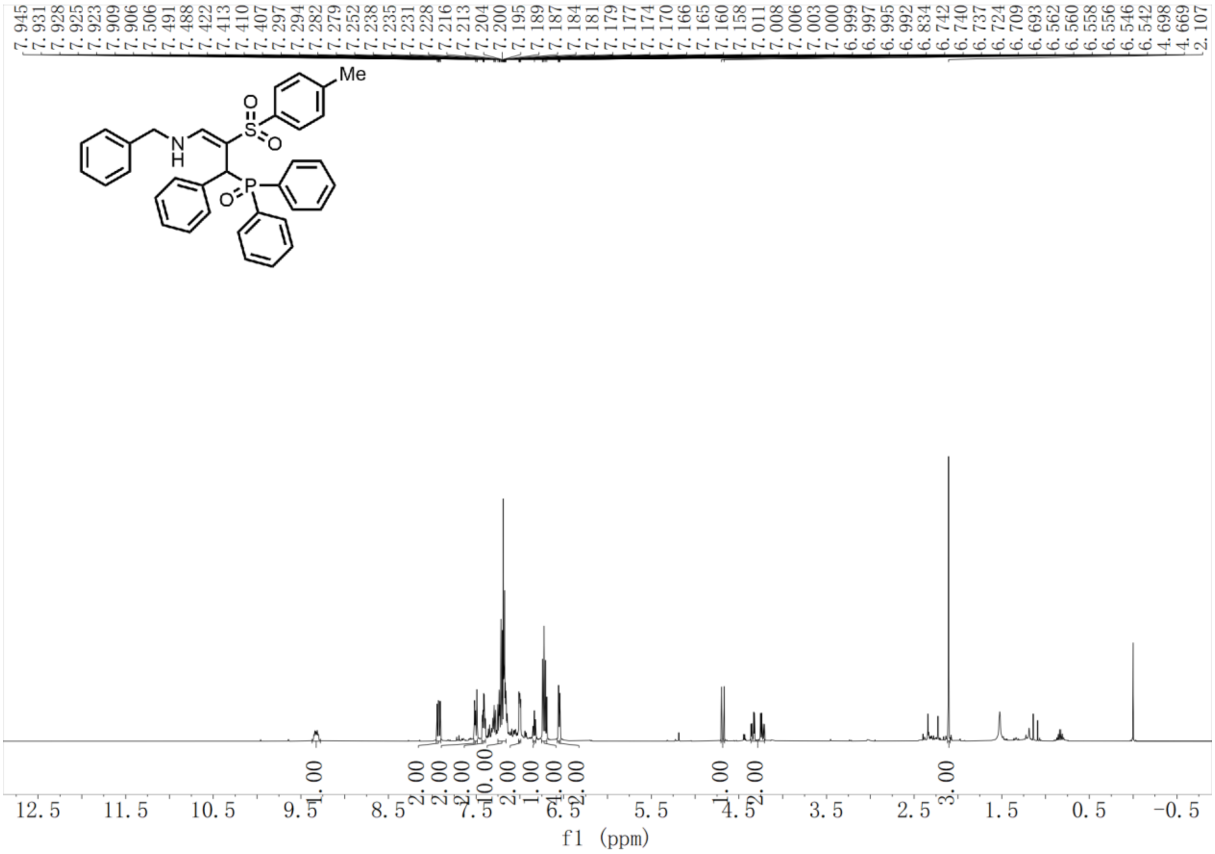

8u – ^{13}C NMR (126 MHz, $CDCl_3$)

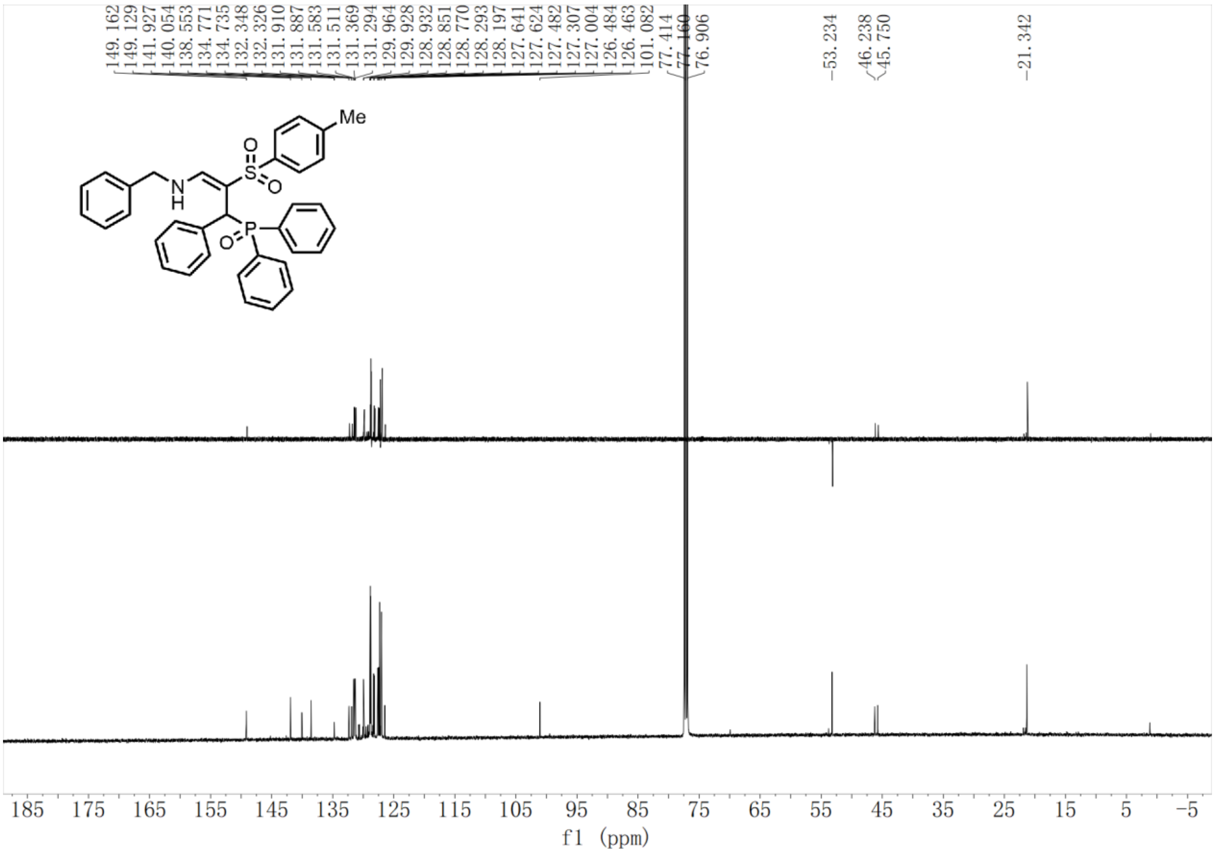

8u – ^{31}P NMR (121 MHz, $CDCl_3$)

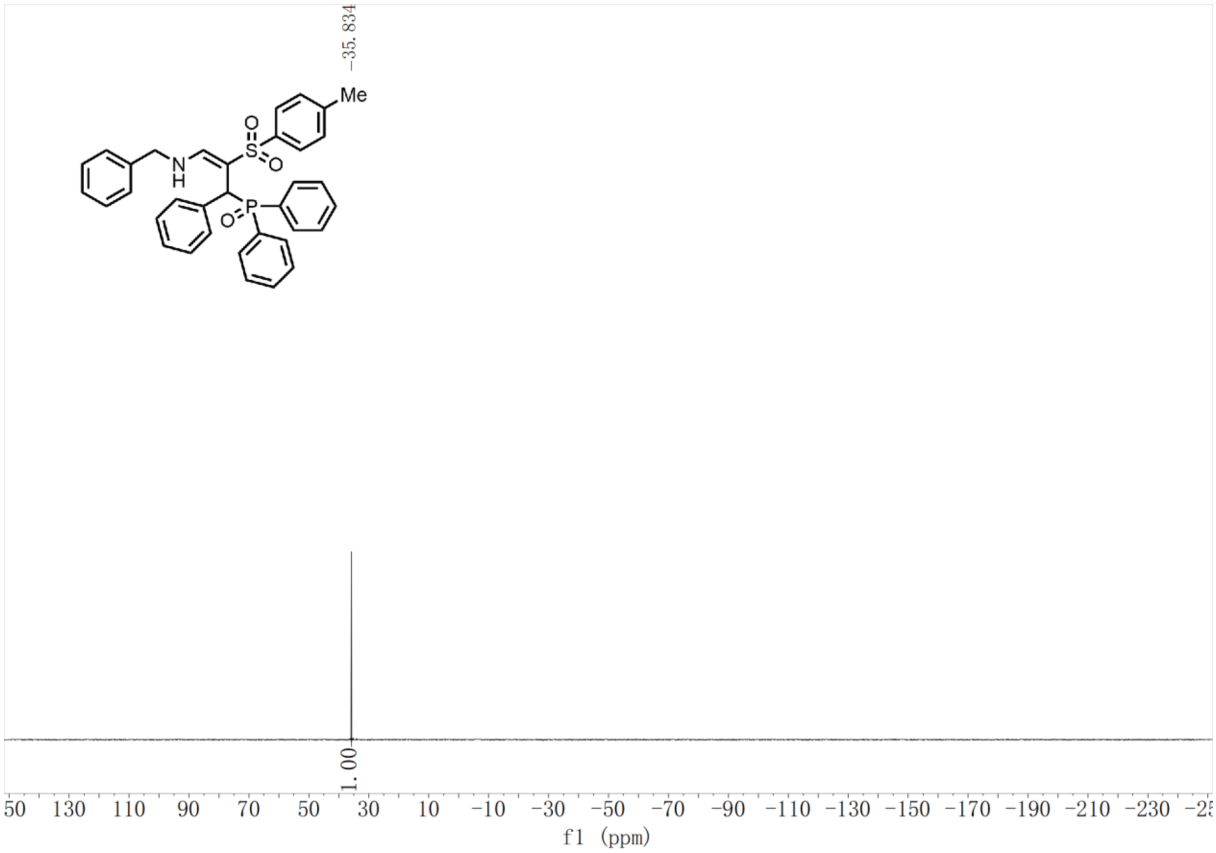

9 – ^{1}H NMR (500 MHz, $CDCl_3$)

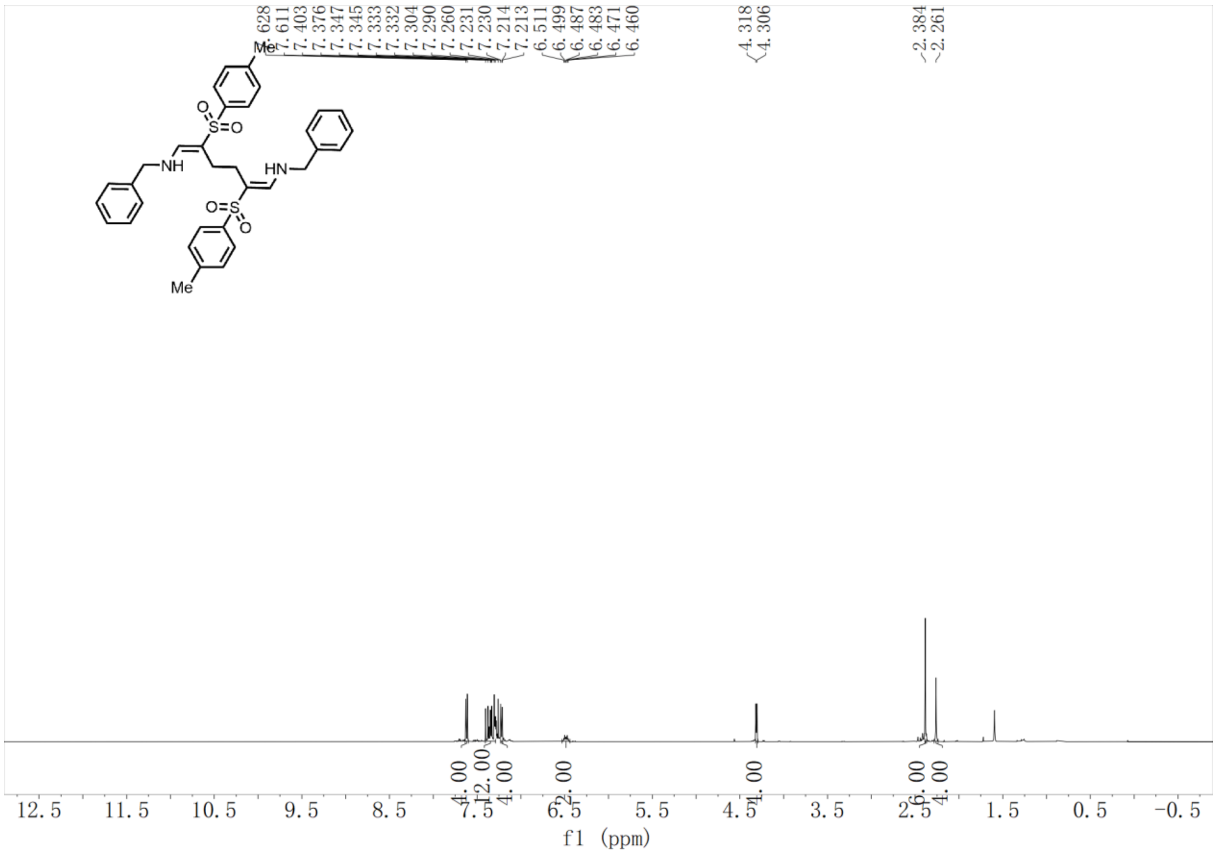

9 – ^{13}C NMR (126 MHz, $CDCl_3$)

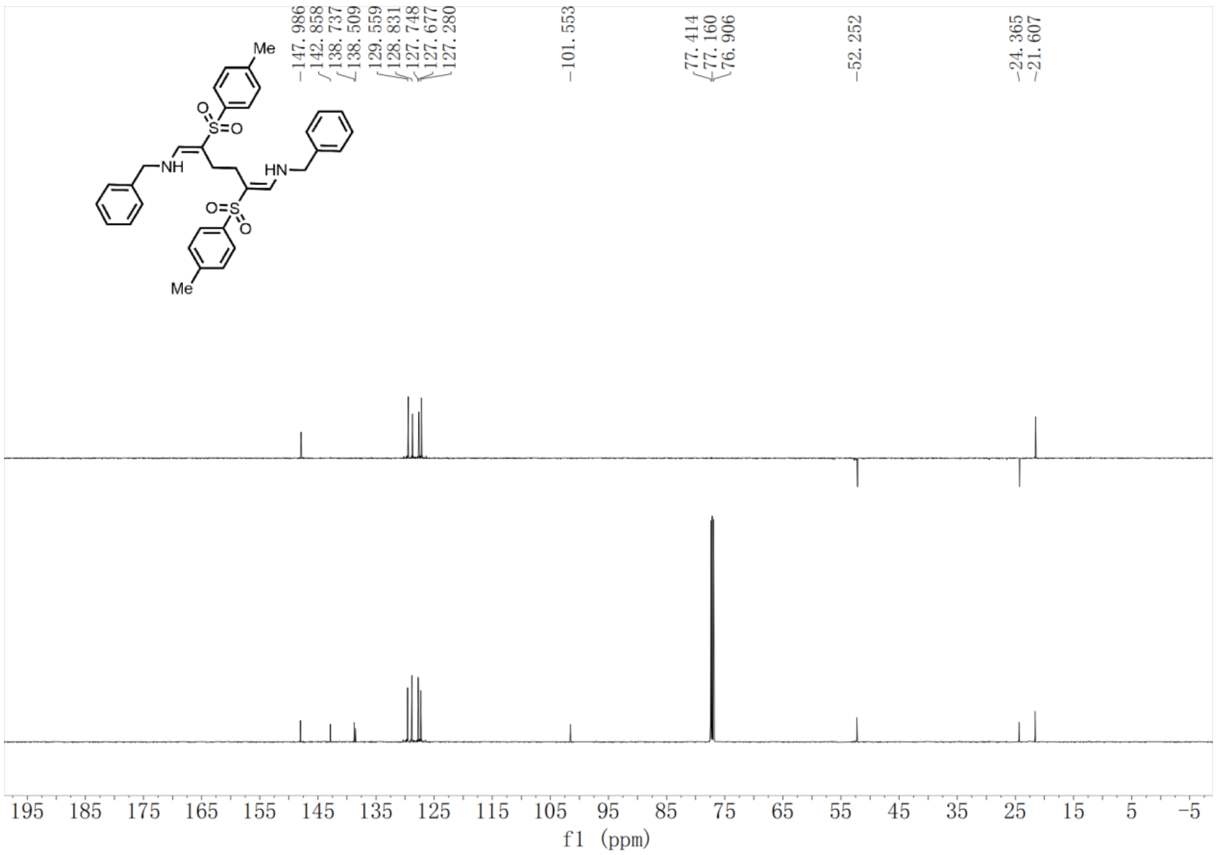

TEMPO-**3a** – ^{1}H NMR (500 MHz, $CDCl_3$)

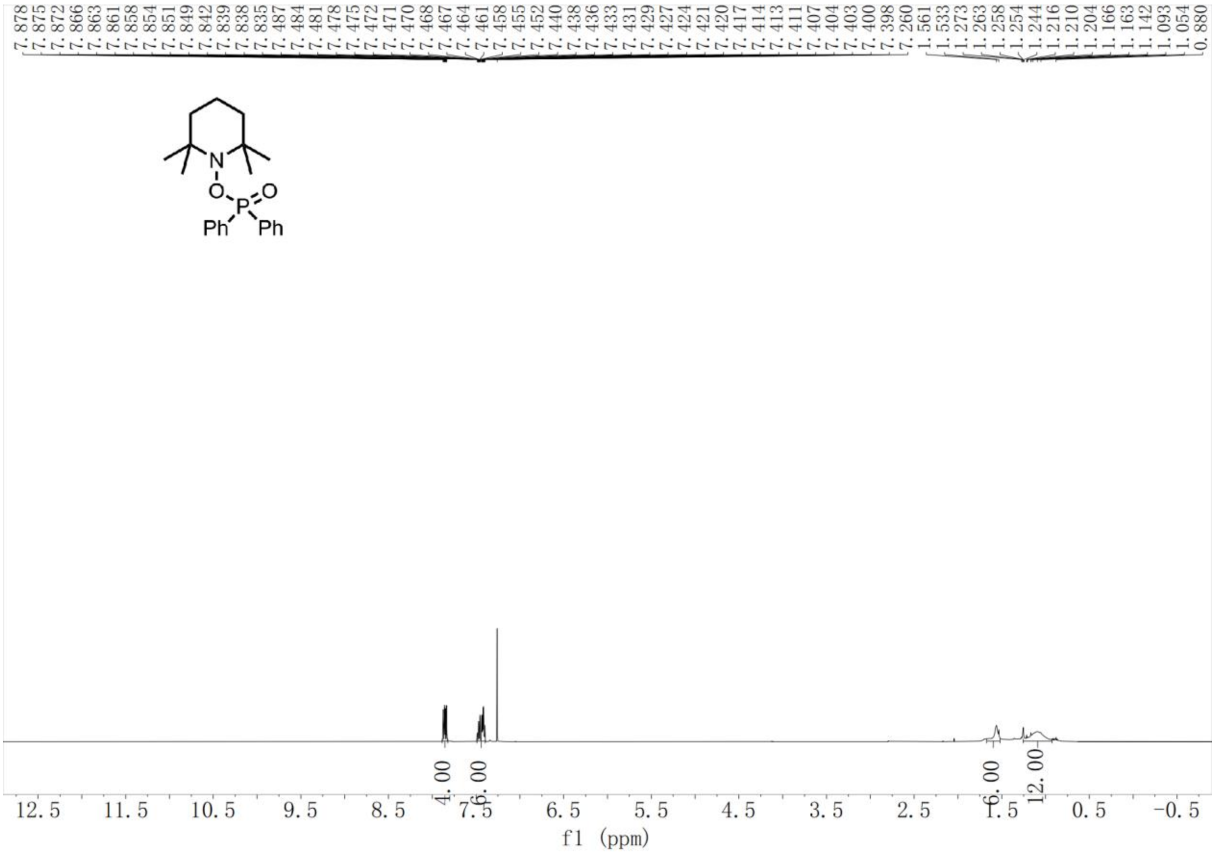

TEMPO-**3a** – ^{13}C NMR (126 MHz, $CDCl_3$)

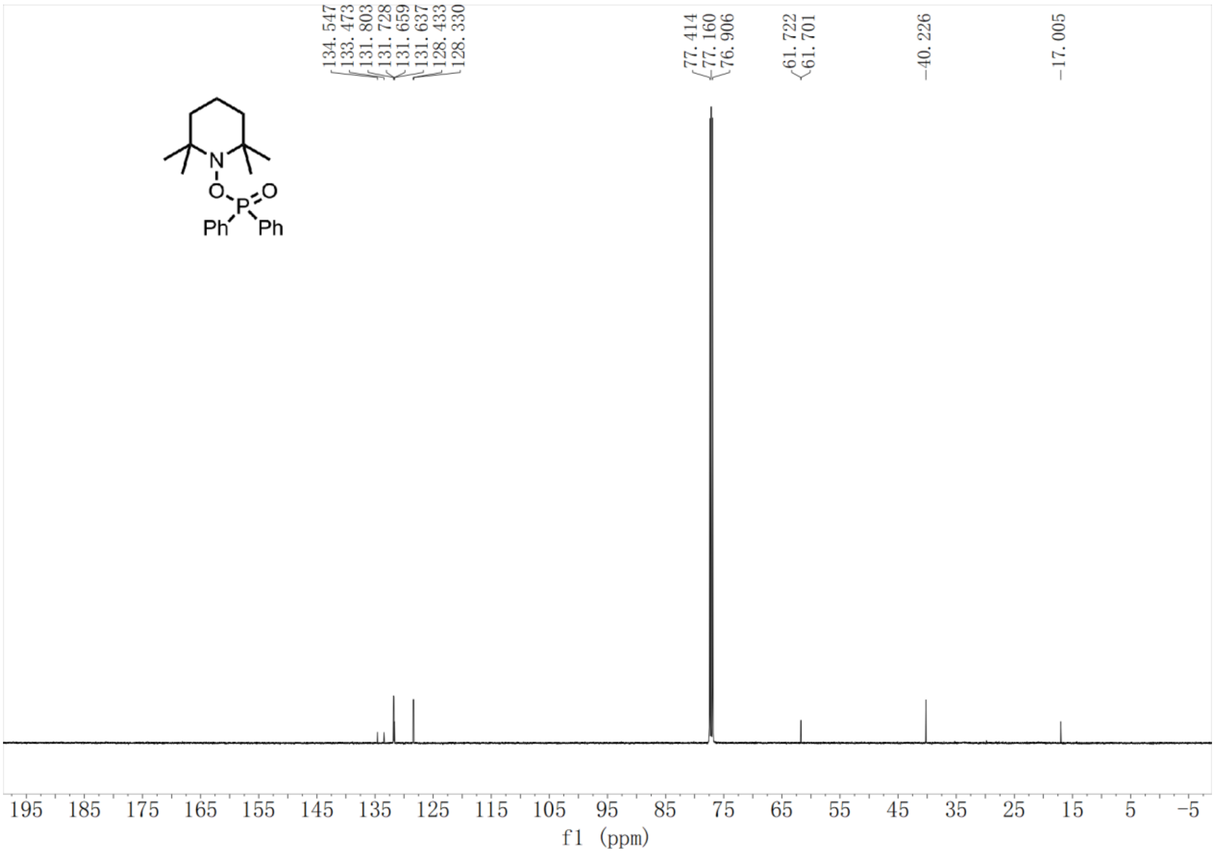

TEMPO-**3a** – ^{31}P NMR (202 MHz, $CDCl_3$)

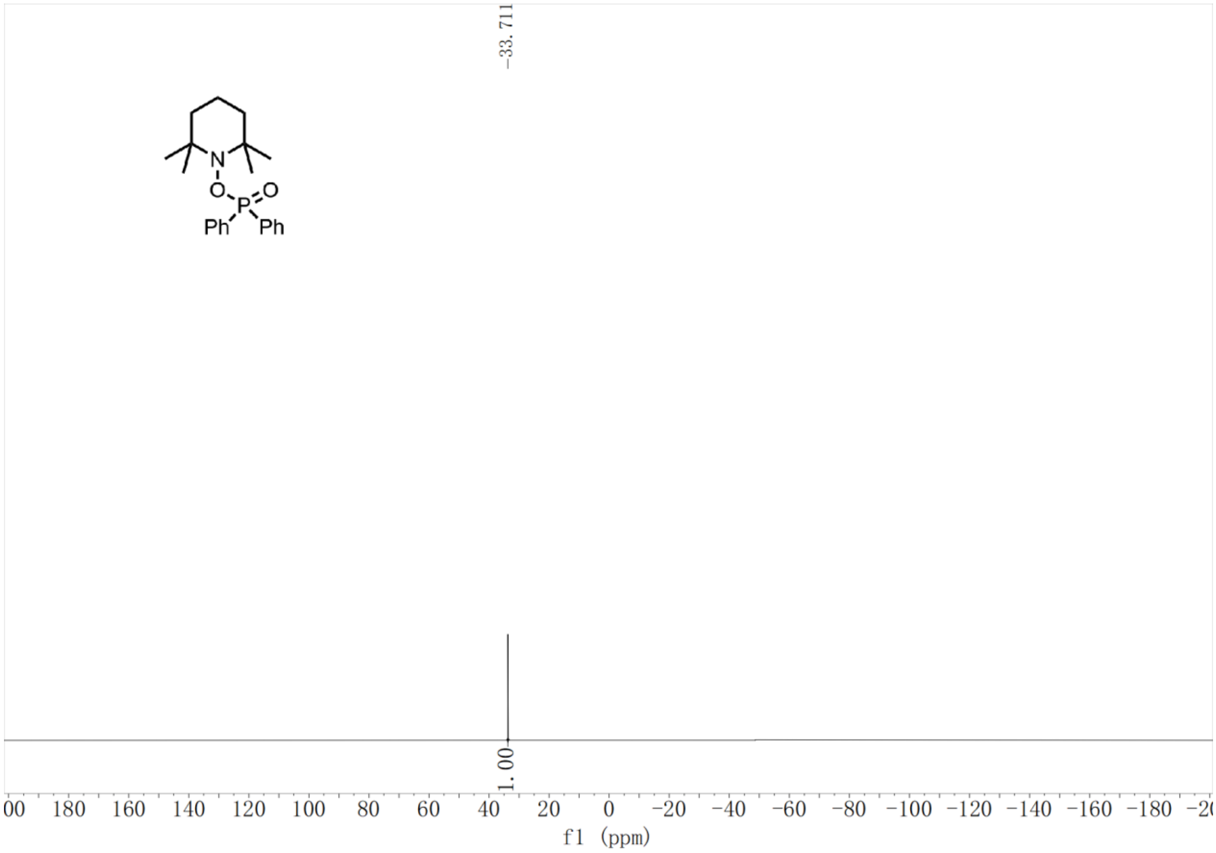

16. X-ray crystallography data

4l (CCDC 2281377) contains the supplementary crystallographic data for this paper. These data can be obtained free of charge from the Cambridge Crystallographic Data Centre.

Compound **4l** was dissolved in a dichloromethane (0.5 mL), and hexane (3.0 mL) were added. The sample was maintained at 4 °C for several days. Crystals were obtained through diffusion.

5 (CCDC 2281378) contains the supplementary crystallographic data for this paper. These data can be obtained free of charge from the Cambridge Crystallographic Data Centre.

Compound **5** was dissolved in benzene-d_6 (3.0 mL). The sample was maintained at 4 °C for several days. Crystals were obtained through diffusion.

8a (CCDC 2310786) contains the supplementary crystallographic data for this paper. These data can be obtained free of charge from the Cambridge Crystallographic Data Centre.

Compound **8a** was dissolved in a dichloromethane (0.5 mL), and hexane (3.0 mL) were added. The sample was maintained at 4 °C for several days. Crystals were obtained through diffusion.

The X-ray diffraction data were collected at and 173 K on a Bruker SMART CCD diffractometer with MoKα radiation (λ = 0.71073 Å). The diffraction data were corrected for absorption using the SADABS program. The structures were solved using SHELXS977 and refined by full matrix least-squares on F2 using SHELXL-2014 in the anisotropic approximation for all non-hydrogen atoms. The hydrogen atoms were introduced at calculated positions and not refined (riding model).

16.1 Crystallography data of 4l (CCDC 2281377)

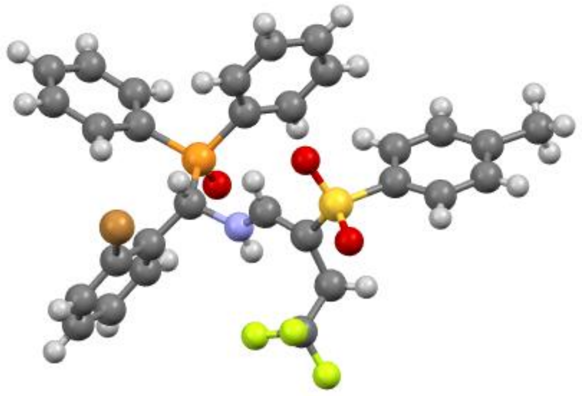

Figure S9. Structure of **4l** (CCDC 2281377): ellipsoid contour probability: 50%.

Table S2. Crystal data and structure refinement for **4l** (CCDC 2281377).

Identification code	**4l** (CCDC 2281377)
Empirical formula	C30 H26 Br F3 N O3 P S
Formula weight	648.46
Temperature	120(2) K
Wavelength	0.71073 A
Crystal system, space group	Triclinic, P -1
Unit cell dimensions	a = 10.2345(4) A alpha = 112.2560(10) deg.<br>b = 12.0047(4) A beta = 111.385(2) deg.<br>c = 13.9944(6) A gamma = 90.4840(10) deg.
Volume	1460.15(10) A^3
Z, Calculated density	2, 1.475 Mg/m^3
Absorption coefficient	1.585 mm^-1
F(000)	660
Crystal size	0.200 x 0.120 x 0.120 mm
Theta range for data collection	1.945 to 30.122 deg.
Limiting indices	-14<=h<=14, -14<=k<=16, -19<=l<=19
Reflections collected / unique	79769 / 8606 [R(int) = 0.0478]
Completeness to theta = 25.242	100.0 %
Absorption correction	Semi-empirical from equivalents
Max. and min. transmission	0.7460 and 0.6389
Refinement method	Full-matrix least-squares on F^2
Data / restraints / parameters	8606 / 0 / 362
Goodness-of-fit on F^2	1.012
Final R indices [I>2sigma(I)]	R1 = 0.0379, wR2 = 0.0870
R indices (all data)	R1 = 0.0513, wR2 = 0.0961
Extinction coefficient	n/a
Largest diff. peak and hole	1.611 and -1.708 e.A^-3

Table S3. Atomic coordinates (x 10^4) and equivalent isotropic displacement parameters (A^2 x 10^3) for **4l** (CCDC 2281377). U(eq) is defined as one third of the trace of the orthogonalized Uij tensor.

	x	y	z	U(eq)
S(1)	6361(1)	1642(1)	7239(1)	18(1)
O(1)	5486(1)	522(1)	6327(1)	23(1)
O(2)	7721(1)	1568(1)	8007(1)	25(1)
C(1)	5390(2)	2320(2)	8069(1)	20(1)
C(2)	4078(2)	2611(2)	7578(2)	28(1)
C(3)	3317(2)	3126(2)	8230(2)	33(1)
C(4)	3856(2)	3360(2)	9369(2)	34(1)
C(5)	5146(3)	3025(3)	9825(2)	45(1)
C(6)	5928(2)	2515(2)	9188(2)	36(1)
C(7)	3066(3)	3980(3)	10093(2)	51(1)
C(8)	6599(2)	2673(2)	6703(1)	17(1)
C(9)	5833(2)	2346(2)	5585(1)	18(1)
N(1)	5766(2)	3040(1)	5013(1)	19(1)
C(10)	5101(2)	2518(2)	3786(1)	18(1)
P(1)	3338(1)	2974(1)	3311(1)	19(1)
O(3)	3382(2)	4318(1)	3660(1)	27(1)
C(11)	2520(2)	2101(2)	1809(2)	24(1)
C(12)	1230(2)	2380(2)	1239(2)	35(1)
C(13)	524(3)	1750(3)	84(2)	47(1)
C(14)	1098(3)	850(3)	-509(2)	51(1)
C(15)	2367(3)	557(3)	45(2)	56(1)
C(16)	3082(3)	1173(2)	1210(2)	42(1)
C(17)	2321(2)	2392(2)	3885(2)	23(1)
C(18)	2033(2)	1146(2)	3603(2)	32(1)
C(19)	1273(3)	724(2)	4075(2)	43(1)
C(20)	762(3)	1544(3)	4803(2)	46(1)
C(21)	1005(3)	2769(3)	5056(2)	45(1)
C(22)	1793(2)	3207(2)	4604(2)	33(1)
C(23)	6109(2)	2834(2)	3315(1)	21(1)
C(24)	7028(2)	2055(2)	2998(2)	26(1)
C(25)	8007(2)	2380(2)	2635(2)	34(1)
C(26)	8078(3)	3500(2)	2574(2)	36(1)
C(27)	7178(3)	4284(2)	2870(2)	35(1)
C(28)	6210(2)	3958(2)	3243(2)	28(1)
Br(1)	6957(1)	494(1)	3026(1)	42(1)
C(29)	7603(2)	3851(2)	7531(1)	19(1)
C(30)	9034(2)	3876(2)	7459(2)	30(1)
F(1)	8961(2)	3902(2)	6493(1)	48(1)
F(2)	9954(1)	4884(1)	8286(1)	44(1)
F(3)	9654(1)	2924(1)	7544(2)	49(1)

Table S4. Selected bond lengths [A] and angles [deg] for **4l** (CCDC 2281377).

Symmetry transformations used to generate equivalent atoms:

Table S5. Bond lengths [A] and angles [deg] for **4l** (CCDC 2281377).

S(1)-O(1)	1.4437(13)
S(1)-O(2)	1.4441(13)
S(1)-C(8)	1.7273(17)
S(1)-C(1)	1.7708(18)
C(1)-C(6)	1.379(3)
C(1)-C(2)	1.384(3)
C(2)-C(3)	1.386(3)
C(2)-H(2)	0.9500
C(3)-C(4)	1.392(3)
C(3)-H(3)	0.9500
C(4)-C(5)	1.379(3)
C(4)-C(7)	1.508(3)
C(5)-C(6)	1.388(3)
C(5)-H(5)	0.9500
C(6)-H(6)	0.9500
C(7)-H(7A)	0.9800
C(7)-H(7B)	0.9800
C(7)-H(7C)	0.9800
C(8)-C(9)	1.361(2)
C(8)-C(29)	1.505(2)
C(9)-N(1)	1.344(2)
C(9)-H(9)	0.9500
N(1)-C(10)	1.460(2)
N(1)-H(1)	0.8800
C(10)-C(23)	1.523(2)
C(10)-P(1)	1.8480(18)
C(10)-H(10)	1.0000
P(1)-O(3)	1.4928(14)
P(1)-C(17)	1.7999(19)
P(1)-C(11)	1.8025(19)
C(11)-C(16)	1.389(3)
C(11)-C(12)	1.390(3)
C(12)-C(13)	1.385(3)
C(12)-H(12)	0.9500
C(13)-C(14)	1.374(4)
C(13)-H(13)	0.9500
C(14)-C(15)	1.375(4)
C(14)-H(14)	0.9500
C(15)-C(16)	1.395(3)
C(15)-H(15)	0.9500
C(16)-H(16)	0.9500
C(17)-C(22)	1.387(3)
C(17)-C(18)	1.394(3)
C(18)-C(19)	1.385(3)
C(18)-H(18)	0.9500
C(19)-C(20)	1.385(4)
C(19)-H(19)	0.9500
C(20)-C(21)	1.373(4)
C(20)-H(20)	0.9500
C(21)-C(22)	1.395(3)
C(21)-H(21)	0.9500
C(22)-H(22)	0.9500
C(23)-C(28)	1.396(3)
C(23)-C(24)	1.401(3)
C(24)-C(25)	1.389(3)
C(24)-Br(1)	1.891(2)
C(25)-C(26)	1.383(3)
C(25)-H(25)	0.9500
C(26)-C(27)	1.379(3)
C(26)-H(26)	0.9500
C(27)-C(28)	1.390(3)
C(27)-H(27)	0.9500
C(28)-H(28)	0.9500
C(29)-C(30)	1.505(3)
C(29)-H(29A)	0.9900
C(29)-H(29B)	0.9900
C(30)-F(3)	1.335(2)
C(30)-F(1)	1.339(3)
C(30)-F(2)	1.348(2)

O(1)-S(1)-O(2)	118.08(8)
O(1)-S(1)-C(8)	109.41(8)
O(2)-S(1)-C(8)	109.58(8)
O(1)-S(1)-C(1)	107.99(8)
O(2)-S(1)-C(1)	106.07(8)
C(8)-S(1)-C(1)	104.86(8)
C(6)-C(1)-C(2)	120.65(18)
C(6)-C(1)-S(1)	119.70(15)
C(2)-C(1)-S(1)	119.62(14)
C(1)-C(2)-C(3)	119.24(18)
C(1)-C(2)-H(2)	120.4
C(3)-C(2)-H(2)	120.4
C(2)-C(3)-C(4)	121.1(2)
C(2)-C(3)-H(3)	119.5
C(4)-C(3)-H(3)	119.5
C(5)-C(4)-C(3)	118.35(19)
C(5)-C(4)-C(7)	120.5(2)
C(3)-C(4)-C(7)	121.2(2)
C(4)-C(5)-C(6)	121.4(2)
C(4)-C(5)-H(5)	119.3
C(6)-C(5)-H(5)	119.3
C(1)-C(6)-C(5)	119.2(2)
C(1)-C(6)-H(6)	120.4
C(5)-C(6)-H(6)	120.4
C(4)-C(7)-H(7A)	109.5
C(4)-C(7)-H(7B)	109.5
H(7A)-C(7)-H(7B)	109.5
C(4)-C(7)-H(7C)	109.5
H(7A)-C(7)-H(7C)	109.5
H(7B)-C(7)-H(7C)	109.5
C(9)-C(8)-C(29)	127.01(15)
C(9)-C(8)-S(1)	116.56(13)
C(29)-C(8)-S(1)	116.41(12)
N(1)-C(9)-C(8)	126.63(16)
N(1)-C(9)-H(9)	116.7
C(8)-C(9)-H(9)	116.7
C(9)-N(1)-C(10)	121.16(14)
C(9)-N(1)-H(1)	119.4
C(10)-N(1)-H(1)	119.4
N(1)-C(10)-C(23)	110.36(14)
N(1)-C(10)-P(1)	110.01(12)
C(23)-C(10)-P(1)	114.48(12)
N(1)-C(10)-H(10)	107.2
C(23)-C(10)-H(10)	107.2
P(1)-C(10)-H(10)	107.2
O(3)-P(1)-C(17)	110.86(9)
O(3)-P(1)-C(11)	113.69(8)
C(17)-P(1)-C(11)	105.89(9)
O(3)-P(1)-C(10)	114.36(8)
C(17)-P(1)-C(10)	105.51(8)
C(11)-P(1)-C(10)	105.84(8)
C(16)-C(11)-C(12)	119.25(19)
C(16)-C(11)-P(1)	124.26(15)
C(12)-C(11)-P(1)	116.48(16)
C(13)-C(12)-C(11)	120.3(2)
C(13)-C(12)-H(12)	119.8
C(11)-C(12)-H(12)	119.8
C(14)-C(13)-C(12)	120.2(2)
C(14)-C(13)-H(13)	119.9
C(12)-C(13)-H(13)	119.9
C(13)-C(14)-C(15)	120.1(2)
C(13)-C(14)-H(14)	119.9
C(15)-C(14)-H(14)	119.9
C(14)-C(15)-C(16)	120.3(3)
C(14)-C(15)-H(15)	119.8
C(16)-C(15)-H(15)	119.8
C(11)-C(16)-C(15)	119.8(2)
C(11)-C(16)-H(16)	120.1
C(15)-C(16)-H(16)	120.1
C(22)-C(17)-C(18)	119.73(19)
C(22)-C(17)-P(1)	118.79(16)
C(18)-C(17)-P(1)	121.47(15)
C(19)-C(18)-C(17)	120.3(2)
C(19)-C(18)-H(18)	119.9

C(17)-C(18)-H(18)	119.9
C(18)-C(19)-C(20)	119.7(2)
C(18)-C(19)-H(19)	120.2
C(20)-C(19)-H(19)	120.2
C(21)-C(20)-C(19)	120.3(2)
C(21)-C(20)-H(20)	119.9
C(19)-C(20)-H(20)	119.9
C(20)-C(21)-C(22)	120.6(2)
C(20)-C(21)-H(21)	119.7
C(22)-C(21)-H(21)	119.7
C(17)-C(22)-C(21)	119.4(2)
C(17)-C(22)-H(22)	120.3
C(21)-C(22)-H(22)	120.3
C(28)-C(23)-C(24)	116.99(17)
C(28)-C(23)-C(10)	120.71(17)
C(24)-C(23)-C(10)	122.20(16)
C(25)-C(24)-C(23)	121.99(19)
C(25)-C(24)-Br(1)	117.25(16)
C(23)-C(24)-Br(1)	120.76(14)
C(26)-C(25)-C(24)	119.5(2)
C(26)-C(25)-H(25)	120.3
C(24)-C(25)-H(25)	120.3
C(25)-C(26)-C(27)	119.97(19)
C(25)-C(26)-H(26)	120.0
C(27)-C(26)-H(26)	120.0
C(26)-C(27)-C(28)	120.3(2)
C(26)-C(27)-H(27)	119.9
C(28)-C(27)-H(27)	119.9
C(27)-C(28)-C(23)	121.3(2)
C(27)-C(28)-H(28)	119.3
C(23)-C(28)-H(28)	119.3
C(8)-C(29)-C(30)	112.70(15)
C(8)-C(29)-H(29A)	109.1
C(30)-C(29)-H(29A)	109.1
C(8)-C(29)-H(29B)	109.1
C(30)-C(29)-H(29B)	109.1
H(29A)-C(29)-H(29B)	107.8
F(3)-C(30)-F(1)	106.80(19)
F(3)-C(30)-F(2)	106.34(17)
F(1)-C(30)-F(2)	105.96(17)
F(3)-C(30)-C(29)	113.79(17)
F(1)-C(30)-C(29)	112.25(16)
F(2)-C(30)-C(29)	111.20(17)

Symmetry transformations used to generate equivalent atoms:

Table S6. Anisotropic displacement parameters (A^2 x 10^3) for **4l** (CCDC 2281377). The anisotropic displacement factor exponent takes the form:
-2 pi^2 [h^2 a*^2 U11 + ... + 2 h k a* b* U12]

	U11	U22	U33	U23	U13	U12
S(1)	20(1)	18(1)	16(1)	8(1)	6(1)	4(1)
O(1)	29(1)	19(1)	20(1)	7(1)	8(1)	0(1)
O(2)	24(1)	28(1)	22(1)	13(1)	5(1)	9(1)
C(1)	22(1)	20(1)	19(1)	8(1)	9(1)	2(1)
C(2)	28(1)	37(1)	26(1)	18(1)	13(1)	12(1)
C(3)	33(1)	40(1)	40(1)	24(1)	23(1)	15(1)
C(4)	40(1)	35(1)	37(1)	14(1)	26(1)	7(1)
C(5)	42(1)	74(2)	21(1)	17(1)	16(1)	13(1)
C(6)	29(1)	60(2)	22(1)	19(1)	10(1)	12(1)
C(7)	62(2)	55(2)	55(2)	22(1)	45(2)	18(1)
C(8)	17(1)	18(1)	17(1)	8(1)	7(1)	3(1)
C(9)	18(1)	18(1)	17(1)	7(1)	8(1)	4(1)
N(1)	23(1)	17(1)	14(1)	5(1)	5(1)	2(1)
C(10)	19(1)	17(1)	15(1)	6(1)	6(1)	1(1)
P(1)	21(1)	17(1)	17(1)	7(1)	6(1)	4(1)
O(3)	33(1)	18(1)	26(1)	9(1)	8(1)	7(1)
C(11)	23(1)	26(1)	18(1)	9(1)	5(1)	1(1)
C(12)	27(1)	43(1)	28(1)	16(1)	3(1)	7(1)
C(13)	34(1)	62(2)	30(1)	21(1)	-5(1)	1(1)
C(14)	48(2)	59(2)	20(1)	7(1)	-3(1)	-9(1)
C(15)	54(2)	60(2)	23(1)	-5(1)	6(1)	7(1)
C(16)	36(1)	45(1)	23(1)	1(1)	2(1)	11(1)
C(17)	19(1)	26(1)	23(1)	11(1)	8(1)	6(1)
C(18)	32(1)	29(1)	46(1)	19(1)	22(1)	9(1)
C(19)	39(1)	45(1)	60(2)	31(1)	25(1)	5(1)
C(20)	31(1)	72(2)	42(1)	27(1)	18(1)	-3(1)
C(21)	37(1)	60(2)	36(1)	7(1)	24(1)	4(1)
C(22)	30(1)	34(1)	29(1)	5(1)	15(1)	6(1)
C(23)	22(1)	21(1)	15(1)	5(1)	6(1)	-2(1)
C(24)	25(1)	23(1)	28(1)	7(1)	12(1)	-2(1)
C(25)	30(1)	33(1)	36(1)	6(1)	19(1)	-3(1)
C(26)	39(1)	40(1)	32(1)	12(1)	19(1)	-8(1)
C(27)	48(1)	31(1)	33(1)	15(1)	19(1)	-3(1)
C(28)	36(1)	25(1)	26(1)	13(1)	14(1)	2(1)
Br(1)	33(1)	27(1)	79(1)	24(1)	35(1)	11(1)
C(29)	18(1)	19(1)	19(1)	7(1)	7(1)	2(1)
C(30)	19(1)	31(1)	39(1)	14(1)	10(1)	2(1)
F(1)	35(1)	73(1)	50(1)	27(1)	30(1)	8(1)
F(2)	24(1)	38(1)	55(1)	18(1)	3(1)	-9(1)
F(3)	22(1)	41(1)	89(1)	31(1)	23(1)	13(1)

Table S7. Hydrogen coordinates (x 10^4) and isotropic displacement parameters (A^2 x 10^3) for **4l** (CCDC 2281377).

	x	y	z	U(eq)
H(2)	3702	2459	6803	34
H(3)	2413	3324	7895	39
H(5)	5507	3145	10593	54
H(6)	6823	2303	9519	43
H(7A)	3286	3708	10704	76
H(7B)	2039	3767	9635	76
H(7C)	3359	4867	10411	76
H(9)	5291	1545	5166	21
H(1)	6128	3823	5385	23
H(10)	4938	1610	3521	21
H(12)	831	3006	1643	41
H(13)	-361	1941	-300	56
H(14)	619	430	-1302	61
H(15)	2758	-69	-367	67
H(16)	3950	959	1592	50
H(18)	2358	583	3085	39
H(19)	1104	-123	3901	52
H(20)	242	1258	5129	55
H(21)	632	3323	5544	54
H(22)	1967	4057	4788	39
H(25)	8623	1836	2430	41
H(26)	8746	3731	2328	44
H(27)	7220	5049	2820	43
H(28)	5605	4510	3453	33
H(29A)	7735	3971	8302	23
H(29B)	7172	4538	7388	23

Table S8. Selected torsion angles [deg] for **4l** (CCDC 2281377).

Symmetry transformations used to generate equivalent atoms:

Table S9. Torsion angles [deg] for **4l** (CCDC 2281377).

O(1)-S(1)-C(1)-C(6)	119.72(18)
O(2)-S(1)-C(1)-C(6)	-7.77(19)
C(8)-S(1)-C(1)-C(6)	-123.70(18)
O(1)-S(1)-C(1)-C(2)	-58.15(17)
O(2)-S(1)-C(1)-C(2)	174.37(15)
C(8)-S(1)-C(1)-C(2)	58.44(17)
C(6)-C(1)-C(2)-C(3)	1.3(3)
S(1)-C(1)-C(2)-C(3)	179.16(16)
C(1)-C(2)-C(3)-C(4)	0.3(3)
C(2)-C(3)-C(4)-C(5)	-2.3(4)
C(2)-C(3)-C(4)-C(7)	176.8(2)
C(3)-C(4)-C(5)-C(6)	2.8(4)
C(7)-C(4)-C(5)-C(6)	-176.4(3)
C(2)-C(1)-C(6)-C(5)	-0.9(4)
S(1)-C(1)-C(6)-C(5)	-178.7(2)
C(4)-C(5)-C(6)-C(1)	-1.2(4)
O(1)-S(1)-C(8)-C(9)	7.13(16)
O(2)-S(1)-C(8)-C(9)	138.06(13)
C(1)-S(1)-C(8)-C(9)	-108.47(14)
O(1)-S(1)-C(8)-C(29)	-174.38(12)
O(2)-S(1)-C(8)-C(29)	-43.45(15)
C(1)-S(1)-C(8)-C(29)	70.01(14)
C(29)-C(8)-C(9)-N(1)	-3.9(3)
S(1)-C(8)-C(9)-N(1)	174.40(14)
C(8)-C(9)-N(1)-C(10)	169.50(16)
C(9)-N(1)-C(10)-C(23)	-127.70(17)
C(9)-N(1)-C(10)-P(1)	105.04(16)
N(1)-C(10)-P(1)-O(3)	62.42(14)
C(23)-C(10)-P(1)-O(3)	-62.51(14)
N(1)-C(10)-P(1)-C(17)	-59.67(13)
C(23)-C(10)-P(1)-C(17)	175.40(12)
N(1)-C(10)-P(1)-C(11)	-171.64(11)
C(23)-C(10)-P(1)-C(11)	63.43(14)
O(3)-P(1)-C(11)-C(16)	134.0(2)
C(17)-P(1)-C(11)-C(16)	-104.1(2)
C(10)-P(1)-C(11)-C(16)	7.6(2)
O(3)-P(1)-C(11)-C(12)	-47.28(19)
C(17)-P(1)-C(11)-C(12)	74.67(18)
C(10)-P(1)-C(11)-C(12)	-173.63(16)
C(16)-C(11)-C(12)-C(13)	-0.9(3)
P(1)-C(11)-C(12)-C(13)	-179.68(19)
C(11)-C(12)-C(13)-C(14)	-0.5(4)
C(12)-C(13)-C(14)-C(15)	1.1(5)
C(13)-C(14)-C(15)-C(16)	-0.3(5)
C(12)-C(11)-C(16)-C(15)	1.6(4)
P(1)-C(11)-C(16)-C(15)	-179.7(2)
C(14)-C(15)-C(16)-C(11)	-1.0(5)
O(3)-P(1)-C(17)-C(22)	-3.82(18)
C(11)-P(1)-C(17)-C(22)	-127.57(16)
C(10)-P(1)-C(17)-C(22)	120.50(16)
O(3)-P(1)-C(17)-C(18)	174.81(16)
C(11)-P(1)-C(17)-C(18)	51.05(18)
C(10)-P(1)-C(17)-C(18)	-60.88(18)
C(22)-C(17)-C(18)-C(19)	-2.8(3)
P(1)-C(17)-C(18)-C(19)	178.63(18)
C(17)-C(18)-C(19)-C(20)	2.0(4)
C(18)-C(19)-C(20)-C(21)	0.0(4)
C(19)-C(20)-C(21)-C(22)	-1.4(4)
C(18)-C(17)-C(22)-C(21)	1.4(3)
P(1)-C(17)-C(22)-C(21)	-179.93(18)
C(20)-C(21)-C(22)-C(17)	0.6(4)
N(1)-C(10)-C(23)-C(28)	-82.9(2)
P(1)-C(10)-C(23)-C(28)	41.8(2)
N(1)-C(10)-C(23)-C(24)	93.3(2)
P(1)-C(10)-C(23)-C(24)	-141.92(15)
C(28)-C(23)-C(24)-C(25)	0.5(3)
C(10)-C(23)-C(24)-C(25)	-175.93(18)
C(28)-C(23)-C(24)-Br(1)	-178.47(14)
C(10)-C(23)-C(24)-Br(1)	5.1(2)
C(23)-C(24)-C(25)-C(26)	-0.5(3)

Br(1)-C(24)-C(25)-C(26)	178.50(17)
C(24)-C(25)-C(26)-C(27)	-0.1(3)
C(25)-C(26)-C(27)-C(28)	0.7(3)
C(26)-C(27)-C(28)-C(23)	-0.8(3)
C(24)-C(23)-C(28)-C(27)	0.2(3)
C(10)-C(23)-C(28)-C(27)	176.60(18)
C(9)-C(8)-C(29)-C(30)	-79.0(2)
S(1)-C(8)-C(29)-C(30)	102.71(16)
C(8)-C(29)-C(30)-F(3)	-53.4(2)
C(8)-C(29)-C(30)-F(1)	68.0(2)
C(8)-C(29)-C(30)-F(2)	-173.45(16)

Symmetry transformations used to generate equivalent atoms:

Table S10. Hydrogen bonds for **4l** (CCDC 2281377) [A and deg.].

D-H...A	d(D-H)	d(H...A)	d(D...A)	<(DHA)

16.2 Crystallography data of 5 (CCDC 2281378)

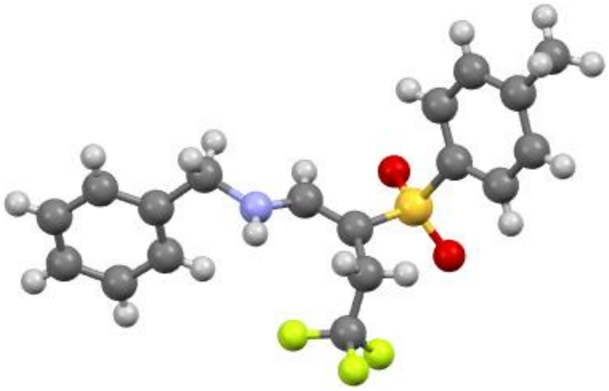

Figure S10. Structure of **5** (CCDC 2281378): ellipsoid contour probability: 50%.

Table S11. Crystal data and structure refinement for **5** (CCDC 2281378).

Identification code	**5** (CCDC 2281378)
Empirical formula	C18 H18 F3 N O2 S
Formula weight	369.39
Temperature	173(2) K
Wavelength	0.71073 A
Crystal system, space group	Monoclinic, P 21/c
Unit cell dimensions	a = 17.628(2) A alpha = 90 deg.<br>b = 7.0992(10) A beta = 92.821(7) deg.<br>c = 13.6449(16) A gamma = 90 deg.
Volume	1705.5(4) A^3
Z, Calculated density	4, 1.439 Mg/m^3
Absorption coefficient	0.232 mm^-1
F(000)	768
Crystal size	0.200 x 0.150 x 0.100 mm
Theta range for data collection	1.157 to 27.962 deg.
Limiting indices	-22<=h<=23, -9<=k<=9, -17<=l<=15
Reflections collected / unique	19385 / 4081 [R(int) = 0.0662]
Completeness to theta = 25.242	100.0 %
Absorption correction	Semi-empirical from equivalents
Max. and min. transmission	0.7456 and 0.6067
Refinement method	Full-matrix least-squares on F^2
Data / restraints / parameters	4081 / 0 / 227
Goodness-of-fit on F^2	1.009
Final R indices [I>2sigma(I)]	R1 = 0.0440, wR2 = 0.0957
R indices (all data)	R1 = 0.0796, wR2 = 0.1115
Extinction coefficient	n/a
Largest diff. peak and hole	0.368 and -0.314 e.A^-3

Table S12. Atomic coordinates (x 10^4) and equivalent isotropic displacement parameters (A^2 x 10^3) for **5** (CCDC 2281378). U(eq) is defined as one third of the trace of the orthogonalized Uij tensor.

	x	y	z	U(eq)
S(1)	2166(1)	6380(1)	6358(1)	25(1)
O(1)	1944(1)	4467(2)	6537(1)	35(1)
O(2)	2711(1)	7277(2)	7034(1)	33(1)
C(1)	1325(1)	7740(3)	6358(1)	24(1)
C(2)	623(1)	6852(3)	6218(2)	32(1)
C(3)	-35(1)	7919(4)	6200(2)	37(1)
C(4)	-11(1)	9866(4)	6323(2)	34(1)
C(5)	698(1)	10712(3)	6465(2)	36(1)
C(6)	1359(1)	9670(3)	6474(2)	32(1)
C(7)	-731(1)	11010(4)	6279(2)	50(1)
C(8)	2485(1)	6557(3)	5183(1)	24(1)
C(9)	2063(1)	5437(3)	4393(2)	27(1)
C(10)	2448(1)	3637(3)	4127(2)	32(1)
F(1)	3163(1)	3931(2)	3865(1)	54(1)
F(2)	2495(1)	2377(2)	4849(1)	48(1)
F(3)	2084(1)	2790(2)	3370(1)	59(1)
N(1)	3328(1)	8262(3)	4157(1)	29(1)
C(11)	3040(1)	7825(3)	5018(1)	25(1)
C(12)	3919(1)	9664(3)	4045(2)	30(1)
C(13)	4687(1)	8860(3)	3813(1)	26(1)
C(14)	4915(1)	7056(3)	4074(2)	34(1)
C(15)	5640(1)	6415(4)	3870(2)	41(1)
C(16)	6135(1)	7574(4)	3402(2)	41(1)
C(17)	5912(1)	9358(4)	3132(2)	38(1)
C(18)	5192(1)	10011(3)	3339(2)	32(1)

Table S13. Selected bond lengths [A] and angles [deg] for **5** (CCDC 2281378).

Symmetry transformations used to generate equivalent atoms:

Table S14. Bond lengths [A] and angles [deg] for **5** (CCDC 2281378).

S(1)-O(1)	1.4381(16)
S(1)-O(2)	1.4476(15)
S(1)-C(8)	1.7282(19)
S(1)-C(1)	1.769(2)
C(1)-C(6)	1.380(3)
C(1)-C(2)	1.393(3)
C(2)-C(3)	1.385(3)
C(2)-H(2)	0.9500
C(3)-C(4)	1.392(3)
C(3)-H(3)	0.9500
C(4)-C(5)	1.392(3)
C(4)-C(7)	1.505(3)
C(5)-C(6)	1.379(3)
C(5)-H(5)	0.9500
C(6)-H(6)	0.9500
C(7)-H(7A)	0.9800
C(7)-H(7B)	0.9800
C(7)-H(7C)	0.9800
C(8)-C(11)	1.357(3)
C(8)-C(9)	1.506(3)
C(9)-C(10)	1.500(3)
C(9)-H(9A)	0.9900
C(9)-H(9B)	0.9900
C(10)-F(2)	1.330(3)
C(10)-F(3)	1.332(2)
C(10)-F(1)	1.343(3)
N(1)-C(11)	1.340(2)
N(1)-C(12)	1.454(3)
N(1)-H(1)	0.8800
C(11)-H(11)	0.9500
C(12)-C(13)	1.517(3)
C(12)-H(12A)	0.9900
C(12)-H(12B)	0.9900
C(13)-C(14)	1.384(3)
C(13)-C(18)	1.391(3)
C(14)-C(15)	1.398(3)
C(14)-H(14)	0.9500
C(15)-C(16)	1.379(3)
C(15)-H(15)	0.9500
C(16)-C(17)	1.371(4)
C(16)-H(16)	0.9500
C(17)-C(18)	1.394(3)
C(17)-H(17)	0.9500
C(18)-H(18)	0.9500
O(1)-S(1)-O(2)	119.01(9)
O(1)-S(1)-C(8)	109.30(10)
O(2)-S(1)-C(8)	108.86(9)
O(1)-S(1)-C(1)	106.26(9)
O(2)-S(1)-C(1)	106.86(9)
C(8)-S(1)-C(1)	105.72(9)
C(6)-C(1)-C(2)	119.90(19)
C(6)-C(1)-S(1)	120.65(16)
C(2)-C(1)-S(1)	119.44(16)
C(3)-C(2)-C(1)	119.5(2)
C(3)-C(2)-H(2)	120.2
C(1)-C(2)-H(2)	120.2
C(2)-C(3)-C(4)	121.3(2)
C(2)-C(3)-H(3)	119.3
C(4)-C(3)-H(3)	119.3
C(5)-C(4)-C(3)	117.8(2)
C(5)-C(4)-C(7)	121.5(2)
C(3)-C(4)-C(7)	120.7(2)
C(6)-C(5)-C(4)	121.5(2)
C(6)-C(5)-H(5)	119.3
C(4)-C(5)-H(5)	119.3
C(5)-C(6)-C(1)	119.9(2)
C(5)-C(6)-H(6)	120.0
C(1)-C(6)-H(6)	120.0
C(4)-C(7)-H(7A)	109.5

C(4)-C(7)-H(7B)	109.5
H(7A)-C(7)-H(7B)	109.5
C(4)-C(7)-H(7C)	109.5
H(7A)-C(7)-H(7C)	109.5
H(7B)-C(7)-H(7C)	109.5
C(11)-C(8)-C(9)	124.47(17)
C(11)-C(8)-S(1)	118.19(15)
C(9)-C(8)-S(1)	116.98(14)
C(10)-C(9)-C(8)	114.21(17)
C(10)-C(9)-H(9A)	108.7
C(8)-C(9)-H(9A)	108.7
C(10)-C(9)-H(9B)	108.7
C(8)-C(9)-H(9B)	108.7
H(9A)-C(9)-H(9B)	107.6
F(2)-C(10)-F(3)	106.32(19)
F(2)-C(10)-F(1)	106.01(18)
F(3)-C(10)-F(1)	106.62(17)
F(2)-C(10)-C(9)	114.01(17)
F(3)-C(10)-C(9)	111.46(18)
F(1)-C(10)-C(9)	111.93(18)
C(11)-N(1)-C(12)	123.66(17)
C(11)-N(1)-H(1)	118.2
C(12)-N(1)-H(1)	118.2
N(1)-C(11)-C(8)	127.35(19)
N(1)-C(11)-H(11)	116.3
C(8)-C(11)-H(11)	116.3
N(1)-C(12)-C(13)	114.59(18)
N(1)-C(12)-H(12A)	108.6
C(13)-C(12)-H(12A)	108.6
N(1)-C(12)-H(12B)	108.6
C(13)-C(12)-H(12B)	108.6
H(12A)-C(12)-H(12B)	107.6
C(14)-C(13)-C(18)	118.63(19)
C(14)-C(13)-C(12)	123.00(19)
C(18)-C(13)-C(12)	118.34(19)
C(13)-C(14)-C(15)	120.5(2)
C(13)-C(14)-H(14)	119.8
C(15)-C(14)-H(14)	119.8
C(16)-C(15)-C(14)	120.2(2)
C(16)-C(15)-H(15)	119.9
C(14)-C(15)-H(15)	119.9
C(17)-C(16)-C(15)	119.8(2)
C(17)-C(16)-H(16)	120.1
C(15)-C(16)-H(16)	120.1
C(16)-C(17)-C(18)	120.3(2)
C(16)-C(17)-H(17)	119.8
C(18)-C(17)-H(17)	119.8
C(13)-C(18)-C(17)	120.6(2)
C(13)-C(18)-H(18)	119.7
C(17)-C(18)-H(18)	119.7

Symmetry transformations used to generate equivalent atoms:

Table S15. Anisotropic displacement parameters (A^2 x 10^3) for **5** (CCDC 2281378). The anisotropic displacement factor exponent takes the form: -2 pi^2 [h^2 a*^2 U11 + ... + 2 h k a* b* U12]

	U11	U22	U33	U23	U13	U12
S(1)	26(1)	31(1)	20(1)	3(1)	2(1)	2(1)
O(1)	39(1)	32(1)	34(1)	9(1)	8(1)	5(1)
O(2)	31(1)	49(1)	19(1)	2(1)	-2(1)	-1(1)
C(1)	26(1)	29(1)	18(1)	1(1)	6(1)	0(1)
C(2)	32(1)	32(1)	33(1)	2(1)	4(1)	0(1)
C(3)	25(1)	49(2)	38(1)	2(1)	4(1)	-1(1)
C(4)	38(1)	46(2)	19(1)	3(1)	6(1)	12(1)
C(5)	47(1)	30(1)	33(1)	0(1)	8(1)	8(1)
C(6)	35(1)	34(1)	27(1)	-1(1)	3(1)	-2(1)
C(7)	48(1)	67(2)	34(1)	5(1)	5(1)	26(1)
C(8)	23(1)	30(1)	18(1)	-1(1)	2(1)	4(1)
C(9)	26(1)	32(1)	23(1)	0(1)	0(1)	2(1)
C(10)	39(1)	31(1)	27(1)	-2(1)	3(1)	0(1)
F(1)	49(1)	45(1)	70(1)	-7(1)	27(1)	9(1)
F(2)	71(1)	33(1)	41(1)	4(1)	9(1)	10(1)
F(3)	83(1)	51(1)	43(1)	-24(1)	-13(1)	3(1)
N(1)	28(1)	39(1)	19(1)	-3(1)	4(1)	-4(1)
C(11)	24(1)	30(1)	20(1)	-1(1)	0(1)	6(1)
C(12)	33(1)	31(1)	26(1)	2(1)	6(1)	0(1)
C(13)	26(1)	34(1)	18(1)	0(1)	1(1)	-1(1)
C(14)	37(1)	39(1)	27(1)	7(1)	5(1)	2(1)
C(15)	42(1)	45(2)	36(1)	2(1)	0(1)	13(1)
C(16)	30(1)	64(2)	29(1)	-9(1)	1(1)	5(1)
C(17)	29(1)	58(2)	26(1)	-7(1)	7(1)	-12(1)
C(18)	35(1)	37(1)	24(1)	-1(1)	1(1)	-4(1)

Table S16. Hydrogen coordinates (x 10^4) and isotropic displacement parameters (A^2 x 10^3) for **5** (CCDC 2281378).

	x	y	z	U(eq)
H(2)	596	5524	6135	39
H(3)	-513	7310	6102	45
H(5)	728	12036	6558	44
H(6)	1837	10280	6559	38
H(7A)	-918	11138	5594	75
H(7B)	-627	12261	6557	75
H(7C)	-1115	10375	6655	75
H(9A)	1550	5137	4615	33
H(9B)	1998	6229	3798	33
H(1)	3149	7667	3628	34
H(11)	3248	8477	5577	30
H(12A)	3978	10402	4660	36
H(12B)	3752	10542	3514	36
H(14)	4577	6248	4395	41
H(15)	5793	5176	4054	49
H(16)	6628	7138	3266	49
H(17)	6251	10152	2802	45
H(18)	5045	11254	3155	38

Table S17. Selected torsion angles [deg] for **5** (CCDC 2281378).

Symmetry transformations used to generate equivalent atoms:

Table S18. Torsion angles [deg] for **5** (CCDC 2281378).

O(1)-S(1)-C(1)-C(6)	162.16(16)
O(2)-S(1)-C(1)-C(6)	34.12(19)
C(8)-S(1)-C(1)-C(6)	-81.75(18)
O(1)-S(1)-C(1)-C(2)	-19.26(19)
O(2)-S(1)-C(1)-C(2)	-147.30(16)
C(8)-S(1)-C(1)-C(2)	96.83(17)
C(6)-C(1)-C(2)-C(3)	-0.3(3)
S(1)-C(1)-C(2)-C(3)	-178.85(16)
C(1)-C(2)-C(3)-C(4)	-0.2(3)
C(2)-C(3)-C(4)-C(5)	0.0(3)
C(2)-C(3)-C(4)-C(7)	178.6(2)
C(3)-C(4)-C(5)-C(6)	0.8(3)
C(7)-C(4)-C(5)-C(6)	-177.8(2)
C(4)-C(5)-C(6)-C(1)	-1.2(3)
C(2)-C(1)-C(6)-C(5)	1.0(3)
S(1)-C(1)-C(6)-C(5)	179.55(16)
O(1)-S(1)-C(8)-C(11)	-147.76(16)
O(2)-S(1)-C(8)-C(11)	-16.26(19)
C(1)-S(1)-C(8)-C(11)	98.23(17)
O(1)-S(1)-C(8)-C(9)	38.78(17)
O(2)-S(1)-C(8)-C(9)	170.27(15)
C(1)-S(1)-C(8)-C(9)	-75.23(17)
C(11)-C(8)-C(9)-C(10)	86.5(2)
S(1)-C(8)-C(9)-C(10)	-100.47(19)
C(8)-C(9)-C(10)-F(2)	65.0(2)
C(8)-C(9)-C(10)-F(3)	-174.67(18)
C(8)-C(9)-C(10)-F(1)	-55.4(2)
C(12)-N(1)-C(11)-C(8)	179.17(19)
C(9)-C(8)-C(11)-N(1)	-2.4(3)
S(1)-C(8)-C(11)-N(1)	-175.34(16)
C(11)-N(1)-C(12)-C(13)	109.0(2)
N(1)-C(12)-C(13)-C(14)	-25.8(3)
N(1)-C(12)-C(13)-C(18)	156.02(18)
C(18)-C(13)-C(14)-C(15)	0.4(3)
C(12)-C(13)-C(14)-C(15)	-177.8(2)
C(13)-C(14)-C(15)-C(16)	-0.2(3)
C(14)-C(15)-C(16)-C(17)	-0.3(3)
C(15)-C(16)-C(17)-C(18)	0.8(3)
C(14)-C(13)-C(18)-C(17)	0.1(3)
C(12)-C(13)-C(18)-C(17)	178.31(19)
C(16)-C(17)-C(18)-C(13)	-0.6(3)

Symmetry transformations used to generate equivalent atoms:

Table S19. Hydrogen bonds for **5** (CCDC 2281378) [A and deg.].

D-H...A	d(D-H)	d(H...A)	d(D...A)	<(DHA)

16.3 Crystallography data of 8a (CCDC 2310786)

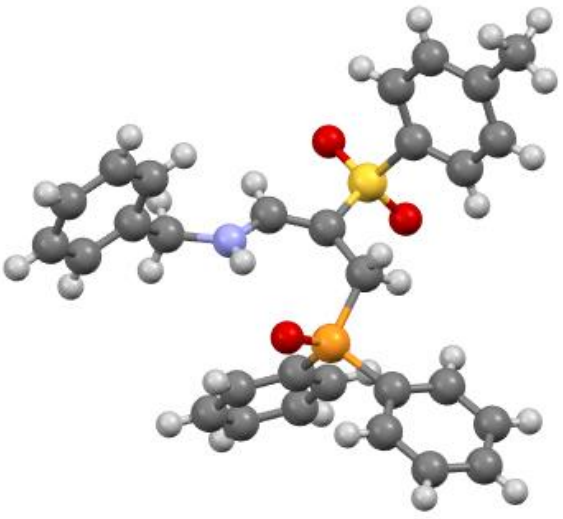

Figure S11. Structure of **8a** (CCDC 2310786): ellipsoid contour probability: 50%.

Table S20. Crystal data and structure refinement for **8a** (CCDC 2310786).

Identification code	**8a** (CCDC 2310786)
Empirical formula	C29 H28 N O3 P S
Formula weight	501.55
Temperature	120(2) K
Wavelength	1.54178 A
Crystal system, space group	Triclinic, P -1
Unit cell dimensions	a = 11.2161(17) A alpha = 89.379(5) deg.<br>b = 11.2502(18) A beta = 62.804(4) deg.<br>c = 11.6904(18) A gamma = 74.413(5) deg.
Volume	1253.0(3) A^3
Z, Calculated density	2, 1.329 Mg/m^3
Absorption coefficient	2.005 mm^-1
F(000)	528
Crystal size	0.200 x 0.180 x 0.150 mm
Theta range for data collection	4.641 to 66.842 deg.
Limiting indices	-13<=h<=13, -13<=k<=13, -13<=l<=13
Reflections collected / unique	20327 / 4345 [R(int) = 0.0525]
Completeness to theta = 66.843	97.6 %
Absorption correction	Semi-empirical from equivalents
Max. and min. transmission	0.7528 and 0.6157
Refinement method	Full-matrix least-squares on F^2
Data / restraints / parameters	4345 / 0 / 317
Goodness-of-fit on F^2	1.021
Final R indices [I>2sigma(I)]	R1 = 0.0416, wR2 = 0.1345
R indices (all data)	R1 = 0.0423, wR2 = 0.1355
Extinction coefficient	n/a
Largest diff. peak and hole	0.404 and -0.511 e.A^-3

Table S21. Atomic coordinates (x 10^4) and equivalent isotropic displacement parameters (A^2 x 10^3) for **8a** (CCDC 2310786). U(eq) is defined as one third of the trace of the orthogonalized Uij tensor.

	x	y	z	U(eq)
C(1)	4849(2)	3743(2)	7387(2)	15(1)
S(1)	3485(1)	4741(1)	8747(1)	14(1)
O(1)	4070(1)	5447(1)	9269(1)	19(1)
O(2)	2382(1)	5423(1)	8456(1)	20(1)
C(2)	2823(2)	3729(2)	9886(2)	16(1)
C(3)	1690(2)	3360(2)	9999(2)	20(1)
C(4)	1266(2)	2486(2)	10815(2)	22(1)
C(5)	1971(2)	1953(2)	11501(2)	21(1)
C(6)	3100(2)	2342(2)	11376(2)	20(1)
C(7)	3522(2)	3238(2)	10590(2)	18(1)
C(8)	1527(2)	977(2)	12358(2)	29(1)
C(9)	6065(2)	3040(2)	7604(2)	16(1)
P(1)	7813(1)	2587(1)	6233(1)	13(1)
O(3)	8024(1)	1580(1)	5277(1)	18(1)
C(10)	8963(2)	2122(2)	6954(2)	15(1)
C(11)	8657(2)	2699(2)	8150(2)	19(1)
C(12)	9588(2)	2314(2)	8649(2)	24(1)
C(13)	10827(2)	1361(2)	7965(2)	24(1)
C(14)	11134(2)	782(2)	6779(2)	21(1)
C(15)	10209(2)	1159(2)	6270(2)	17(1)
C(16)	8112(2)	3966(2)	5482(2)	15(1)
C(17)	7980(2)	5040(2)	6172(2)	19(1)
C(18)	8260(2)	6055(2)	5534(2)	21(1)
C(19)	8673(2)	6010(2)	4215(2)	21(1)
C(20)	8816(2)	4942(2)	3526(2)	21(1)
C(21)	8533(2)	3919(2)	4154(2)	18(1)
C(22)	4636(2)	3648(2)	6338(2)	16(1)
N(1)	5484(2)	2887(1)	5217(1)	17(1)
C(23)	5327(2)	3114(2)	4047(2)	19(1)
C(24)	5452(2)	1944(2)	3323(2)	17(1)
C(25)	4667(2)	1146(2)	3952(2)	23(1)
C(26)	4761(2)	97(2)	3267(2)	27(1)
C(27)	5649(2)	-174(2)	1938(2)	27(1)
C(28)	6425(2)	618(2)	1304(2)	26(1)
C(29)	6331(2)	1672(2)	1992(2)	22(1)

Table S22. Selected bond lengths [A] and angles [deg] for **8a** (CCDC 2310786).

Symmetry transformations used to generate equivalent atoms:

Table S23. Bond lengths [A] and angles [deg] for **8a** (CCDC 2310786).

C(1)-C(22)	1.363(2)
C(1)-C(9)	1.511(2)
C(1)-S(1)	1.7381(18)
S(1)-O(1)	1.4462(13)
S(1)-O(2)	1.4493(13)
S(1)-C(2)	1.7721(17)
C(2)-C(3)	1.390(3)
C(2)-C(7)	1.394(3)
C(3)-C(4)	1.386(3)
C(3)-H(3)	0.9500
C(4)-C(5)	1.393(3)
C(4)-H(4)	0.9500
C(5)-C(6)	1.394(3)
C(5)-C(8)	1.507(3)
C(6)-C(7)	1.386(3)
C(6)-H(6)	0.9500
C(7)-H(7)	0.9500
C(8)-H(8A)	0.9800
C(8)-H(8B)	0.9800
C(8)-H(8C)	0.9800
C(9)-P(1)	1.8118(18)
C(9)-H(9A)	0.9900
C(9)-H(9B)	0.9900
P(1)-O(3)	1.4933(13)
P(1)-C(16)	1.8077(17)
P(1)-C(10)	1.8081(18)
C(10)-C(15)	1.396(3)
C(10)-C(11)	1.399(2)
C(11)-C(12)	1.388(3)
C(11)-H(11)	0.9500
C(12)-C(13)	1.386(3)
C(12)-H(12)	0.9500
C(13)-C(14)	1.389(3)
C(13)-H(13)	0.9500
C(14)-C(15)	1.390(3)
C(14)-H(14)	0.9500
C(15)-H(15)	0.9500
C(16)-C(17)	1.397(2)
C(16)-C(21)	1.398(3)
C(17)-C(18)	1.385(3)
C(17)-H(17)	0.9500
C(18)-C(19)	1.389(3)
C(18)-H(18)	0.9500
C(19)-C(20)	1.385(3)
C(19)-H(19)	0.9500
C(20)-C(21)	1.389(3)
C(20)-H(20)	0.9500
C(21)-H(21)	0.9500
C(22)-N(1)	1.343(2)
C(22)-H(22)	0.9500
N(1)-C(23)	1.467(2)
N(1)-H(1)	0.8800
C(23)-C(24)	1.512(2)
C(23)-H(23A)	0.9900
C(23)-H(23B)	0.9900
C(24)-C(29)	1.390(3)
C(24)-C(25)	1.393(3)
C(25)-C(26)	1.386(3)
C(25)-H(25)	0.9500
C(26)-C(27)	1.390(3)
C(26)-H(26)	0.9500
C(27)-C(28)	1.384(3)
C(27)-H(27)	0.9500
C(28)-C(29)	1.393(3)
C(28)-H(28)	0.9500
C(29)-H(29)	0.9500
C(22)-C(1)-C(9)	130.00(16)
C(22)-C(1)-S(1)	116.68(13)
C(9)-C(1)-S(1)	113.16(12)

O(1)-S(1)-O(2)	118.02(8)
O(1)-S(1)-C(1)	108.54(8)
O(2)-S(1)-C(1)	110.29(8)
O(1)-S(1)-C(2)	106.78(8)
O(2)-S(1)-C(2)	108.26(8)
C(1)-S(1)-C(2)	103.98(8)
C(3)-C(2)-C(7)	120.81(16)
C(3)-C(2)-S(1)	120.11(14)
C(7)-C(2)-S(1)	118.90(13)
C(4)-C(3)-C(2)	119.11(17)
C(4)-C(3)-H(3)	120.4
C(2)-C(3)-H(3)	120.4
C(3)-C(4)-C(5)	121.29(17)
C(3)-C(4)-H(4)	119.4
C(5)-C(4)-H(4)	119.4
C(4)-C(5)-C(6)	118.48(17)
C(4)-C(5)-C(8)	120.87(17)
C(6)-C(5)-C(8)	120.65(17)
C(7)-C(6)-C(5)	121.29(17)
C(7)-C(6)-H(6)	119.4
C(5)-C(6)-H(6)	119.4
C(6)-C(7)-C(2)	118.97(16)
C(6)-C(7)-H(7)	120.5
C(2)-C(7)-H(7)	120.5
C(5)-C(8)-H(8A)	109.5
C(5)-C(8)-H(8B)	109.5
H(8A)-C(8)-H(8B)	109.5
C(5)-C(8)-H(8C)	109.5
H(8A)-C(8)-H(8C)	109.5
H(8B)-C(8)-H(8C)	109.5
C(1)-C(9)-P(1)	118.51(12)
C(1)-C(9)-H(9A)	107.7
P(1)-C(9)-H(9A)	107.7
C(1)-C(9)-H(9B)	107.7
P(1)-C(9)-H(9B)	107.7
H(9A)-C(9)-H(9B)	107.1
O(3)-P(1)-C(16)	111.19(8)
O(3)-P(1)-C(10)	112.65(8)
C(16)-P(1)-C(10)	107.80(8)
O(3)-P(1)-C(9)	112.95(8)
C(16)-P(1)-C(9)	107.61(8)
C(10)-P(1)-C(9)	104.24(8)
C(15)-C(10)-C(11)	119.49(16)
C(15)-C(10)-P(1)	117.99(13)
C(11)-C(10)-P(1)	122.51(13)
C(12)-C(11)-C(10)	120.03(17)
C(12)-C(11)-H(11)	120.0
C(10)-C(11)-H(11)	120.0
C(13)-C(12)-C(11)	120.33(17)
C(13)-C(12)-H(12)	119.8
C(11)-C(12)-H(12)	119.8
C(12)-C(13)-C(14)	119.87(17)
C(12)-C(13)-H(13)	120.1
C(14)-C(13)-H(13)	120.1
C(13)-C(14)-C(15)	120.32(17)
C(13)-C(14)-H(14)	119.8
C(15)-C(14)-H(14)	119.8
C(14)-C(15)-C(10)	119.96(17)
C(14)-C(15)-H(15)	120.0
C(10)-C(15)-H(15)	120.0
C(17)-C(16)-C(21)	119.85(16)
C(17)-C(16)-P(1)	122.30(13)
C(21)-C(16)-P(1)	117.82(13)
C(18)-C(17)-C(16)	119.56(17)
C(18)-C(17)-H(17)	120.2
C(16)-C(17)-H(17)	120.2
C(17)-C(18)-C(19)	120.54(17)
C(17)-C(18)-H(18)	119.7
C(19)-C(18)-H(18)	119.7
C(20)-C(19)-C(18)	120.05(17)
C(20)-C(19)-H(19)	120.0
C(18)-C(19)-H(19)	120.0
C(19)-C(20)-C(21)	120.04(17)
C(19)-C(20)-H(20)	120.0

C(21)-C(20)-H(20)	120.0
C(20)-C(21)-C(16)	119.95(17)
C(20)-C(21)-H(21)	120.0
C(16)-C(21)-H(21)	120.0
N(1)-C(22)-C(1)	127.83(17)
N(1)-C(22)-H(22)	116.1
C(1)-C(22)-H(22)	116.1
C(22)-N(1)-C(23)	121.56(15)
C(22)-N(1)-H(1)	119.2
C(23)-N(1)-H(1)	119.2
N(1)-C(23)-C(24)	112.99(14)
N(1)-C(23)-H(23A)	109.0
C(24)-C(23)-H(23A)	109.0
N(1)-C(23)-H(23B)	109.0
C(24)-C(23)-H(23B)	109.0
H(23A)-C(23)-H(23B)	107.8
C(29)-C(24)-C(25)	118.85(17)
C(29)-C(24)-C(23)	119.71(16)
C(25)-C(24)-C(23)	121.40(17)
C(26)-C(25)-C(24)	120.73(18)
C(26)-C(25)-H(25)	119.6
C(24)-C(25)-H(25)	119.6
C(25)-C(26)-C(27)	120.17(18)
C(25)-C(26)-H(26)	119.9
C(27)-C(26)-H(26)	119.9
C(28)-C(27)-C(26)	119.45(18)
C(28)-C(27)-H(27)	120.3
C(26)-C(27)-H(27)	120.3
C(27)-C(28)-C(29)	120.42(19)
C(27)-C(28)-H(28)	119.8
C(29)-C(28)-H(28)	119.8
C(24)-C(29)-C(28)	120.38(18)
C(24)-C(29)-H(29)	119.8
C(28)-C(29)-H(29)	119.8

Symmetry transformations used to generate equivalent atoms:

Table S24. Anisotropic displacement parameters (A^2 x 10^3) for **8a** (CCDC 2310786). The anisotropic displacement factor exponent takes the form: -2 pi^2 [h^2 a*^2 U11 + ... + 2 h k a* b* U12]

	U11	U22	U33	U23	U13	U12
C(1)	14(1)	17(1)	14(1)	2(1)	-6(1)	-6(1)
S(1)	14(1)	14(1)	14(1)	1(1)	-7(1)	-4(1)
O(1)	22(1)	18(1)	21(1)	0(1)	-11(1)	-7(1)
O(2)	17(1)	21(1)	20(1)	3(1)	-10(1)	-2(1)
C(2)	15(1)	17(1)	13(1)	0(1)	-5(1)	-5(1)
C(3)	19(1)	25(1)	18(1)	2(1)	-10(1)	-7(1)
C(4)	20(1)	27(1)	20(1)	1(1)	-7(1)	-12(1)
C(5)	23(1)	20(1)	13(1)	0(1)	-3(1)	-6(1)
C(6)	19(1)	22(1)	15(1)	1(1)	-7(1)	-2(1)
C(7)	16(1)	21(1)	15(1)	0(1)	-7(1)	-5(1)
C(8)	33(1)	26(1)	24(1)	8(1)	-8(1)	-11(1)
C(9)	15(1)	18(1)	17(1)	4(1)	-8(1)	-6(1)
P(1)	13(1)	14(1)	13(1)	0(1)	-6(1)	-4(1)
O(3)	19(1)	17(1)	19(1)	-2(1)	-10(1)	-4(1)
C(10)	16(1)	14(1)	16(1)	3(1)	-7(1)	-6(1)
C(11)	16(1)	21(1)	16(1)	-2(1)	-7(1)	-3(1)
C(12)	23(1)	31(1)	18(1)	0(1)	-11(1)	-6(1)
C(13)	22(1)	26(1)	27(1)	6(1)	-16(1)	-6(1)
C(14)	17(1)	17(1)	28(1)	2(1)	-11(1)	-3(1)
C(15)	18(1)	15(1)	17(1)	1(1)	-7(1)	-6(1)
C(16)	12(1)	16(1)	17(1)	1(1)	-7(1)	-4(1)
C(17)	18(1)	21(1)	16(1)	1(1)	-8(1)	-6(1)
C(18)	21(1)	17(1)	25(1)	0(1)	-11(1)	-6(1)
C(19)	17(1)	21(1)	28(1)	8(1)	-11(1)	-8(1)
C(20)	18(1)	28(1)	17(1)	5(1)	-9(1)	-7(1)
C(21)	17(1)	20(1)	17(1)	0(1)	-8(1)	-5(1)
C(22)	17(1)	16(1)	18(1)	3(1)	-8(1)	-8(1)
N(1)	18(1)	19(1)	16(1)	1(1)	-10(1)	-4(1)
C(23)	24(1)	20(1)	16(1)	4(1)	-11(1)	-8(1)
C(24)	20(1)	17(1)	18(1)	3(1)	-13(1)	-5(1)
C(25)	28(1)	25(1)	21(1)	7(1)	-14(1)	-11(1)
C(26)	36(1)	20(1)	33(1)	8(1)	-21(1)	-13(1)
C(27)	31(1)	20(1)	34(1)	-5(1)	-21(1)	-2(1)
C(28)	25(1)	27(1)	23(1)	-4(1)	-12(1)	-2(1)
C(29)	22(1)	22(1)	22(1)	3(1)	-12(1)	-6(1)

Table S25. Hydrogen coordinates (x 10^4) and isotropic displacement parameters (A^2 x 10^3) for **8a** (CCDC 2310786).

	x	y	z	U(eq)
H(3)	1213	3703	9522	24
H(4)	479	2246	10909	27
H(6)	3590	1987	11838	24
H(7)	4276	3512	10531	21
H(8A)	654	1372	13151	43
H(8B)	2263	564	12585	43
H(8C)	1373	361	11895	43
H(9A)	5868	2275	7969	20
H(9B)	6078	3558	8276	20
H(11)	7811	3355	8622	22
H(12)	9376	2706	9463	29
H(13)	11464	1104	8307	28
H(14)	11980	125	6313	25
H(15)	10424	761	5457	21
H(17)	7700	5075	7074	22
H(18)	8170	6788	6001	25
H(19)	8858	6712	3787	25
H(20)	9109	4909	2622	25
H(21)	8626	3189	3682	22
H(22)	3785	4185	6409	19
H(1)	6149	2236	5182	21
H(23A)	6055	3488	3462	22
H(23B)	4398	3721	4297	22
H(25)	4061	1322	4861	28
H(26)	4217	-438	3708	32
H(27)	5722	-898	1469	32
H(28)	7026	442	394	31
H(29)	6872	2209	1548	26

Table S26. Selected torsion angles [deg] for **8a** (CCDC 2310786).

Symmetry transformations used to generate equivalent atoms:

Table S27. Torsion angles [deg] for **8a** (CCDC 2310786).

C(22)-C(1)-S(1)-O(1)	-137.24(14)
C(9)-C(1)-S(1)-O(1)	46.89(14)
C(22)-C(1)-S(1)-O(2)	-6.52(16)
C(9)-C(1)-S(1)-O(2)	177.61(11)
C(22)-C(1)-S(1)-C(2)	109.35(14)
C(9)-C(1)-S(1)-C(2)	-66.52(13)
O(1)-S(1)-C(2)-C(3)	150.74(15)
O(2)-S(1)-C(2)-C(3)	22.70(17)
C(1)-S(1)-C(2)-C(3)	-94.59(16)
O(1)-S(1)-C(2)-C(7)	-34.11(16)
O(2)-S(1)-C(2)-C(7)	-162.16(14)
C(1)-S(1)-C(2)-C(7)	80.55(15)
C(7)-C(2)-C(3)-C(4)	-0.5(3)
S(1)-C(2)-C(3)-C(4)	174.54(14)
C(2)-C(3)-C(4)-C(5)	-1.4(3)
C(3)-C(4)-C(5)-C(6)	1.6(3)
C(3)-C(4)-C(5)-C(8)	-178.27(17)
C(4)-C(5)-C(6)-C(7)	0.0(3)
C(8)-C(5)-C(6)-C(7)	179.88(17)
C(5)-C(6)-C(7)-C(2)	-1.8(3)
C(3)-C(2)-C(7)-C(6)	2.1(3)
S(1)-C(2)-C(7)-C(6)	-173.05(13)
C(22)-C(1)-C(9)-P(1)	34.4(2)
S(1)-C(1)-C(9)-P(1)	-150.40(10)
C(1)-C(9)-P(1)-O(3)	-70.41(14)
C(1)-C(9)-P(1)-C(16)	52.69(15)
C(1)-C(9)-P(1)-C(10)	166.98(13)
O(3)-P(1)-C(10)-C(15)	23.55(16)
C(16)-P(1)-C(10)-C(15)	-99.49(14)
C(9)-P(1)-C(10)-C(15)	146.36(14)
O(3)-P(1)-C(10)-C(11)	-157.30(14)
C(16)-P(1)-C(10)-C(11)	79.66(16)
C(9)-P(1)-C(10)-C(11)	-34.50(17)
C(15)-C(10)-C(11)-C(12)	0.0(3)
P(1)-C(10)-C(11)-C(12)	-179.09(14)
C(10)-C(11)-C(12)-C(13)	0.2(3)
C(11)-C(12)-C(13)-C(14)	-0.4(3)
C(12)-C(13)-C(14)-C(15)	0.4(3)
C(13)-C(14)-C(15)-C(10)	-0.1(3)
C(11)-C(10)-C(15)-C(14)	-0.1(3)
P(1)-C(10)-C(15)-C(14)	179.10(13)
O(3)-P(1)-C(16)-C(17)	179.98(13)
C(10)-P(1)-C(16)-C(17)	-56.09(16)
C(9)-P(1)-C(16)-C(17)	55.81(16)
O(3)-P(1)-C(16)-C(21)	-2.18(16)
C(10)-P(1)-C(16)-C(21)	121.75(14)
C(9)-P(1)-C(16)-C(21)	-126.35(14)
C(21)-C(16)-C(17)-C(18)	0.4(3)
P(1)-C(16)-C(17)-C(18)	178.23(13)
C(16)-C(17)-C(18)-C(19)	-0.1(3)
C(17)-C(18)-C(19)-C(20)	-0.4(3)
C(18)-C(19)-C(20)-C(21)	0.7(3)
C(19)-C(20)-C(21)-C(16)	-0.4(3)
C(17)-C(16)-C(21)-C(20)	-0.2(3)
P(1)-C(16)-C(21)-C(20)	-178.06(13)
C(9)-C(1)-C(22)-N(1)	-0.2(3)
S(1)-C(1)-C(22)-N(1)	-175.24(14)
C(1)-C(22)-N(1)-C(23)	-162.57(17)
C(22)-N(1)-C(23)-C(24)	-136.85(17)
N(1)-C(23)-C(24)-C(29)	-131.05(17)
N(1)-C(23)-C(24)-C(25)	51.1(2)
C(29)-C(24)-C(25)-C(26)	0.1(3)
C(23)-C(24)-C(25)-C(26)	178.00(17)
C(24)-C(25)-C(26)-C(27)	0.3(3)
C(25)-C(26)-C(27)-C(28)	-0.7(3)
C(26)-C(27)-C(28)-C(29)	0.7(3)
C(25)-C(24)-C(29)-C(28)	-0.1(3)
C(23)-C(24)-C(29)-C(28)	-178.04(17)
C(27)-C(28)-C(29)-C(24)	-0.3(3)

Symmetry transformations used to generate equivalent atoms:

Table S28. Hydrogen bonds for **8a** (CCDC 2310786) [A and deg.].

D-H...A	d(D-H)	d(H...A)	d(D...A)	<(DHA)

17. Computational details

DFT and time-dependent DFT (TDDFT) calculations have been performed to describe ground and excited states, respectively. All the calculations were carried out using Gaussian16 program suite and the global hybrid functional PBE0[12] in conjunction with the 6-311++G(d,p) basis set[13]. The effect of the solvent (here DMSO) has been modeled by the means of the Polarisable Continuum Model (PCM)[14] using the integral equation formalism variant (IEFPCM)[15]. Only the absorption spectrum of compound **1a** and the corresponding fluorescence from ^{1}**A*** state have been modeled considering acetonitrile (MeCN) as solvent to enable direct comparison with the experimental data. The computed spectra are reported in **Figure S12**. For the same reason, the phosphorescence of the triplet ^{3}**A***$_{\textit{s-trans-E}}$ state has been computed using tetrahydrofuran (THF) as solvent. In the case of open shell diradicals, the Broken Symmetry approach has been applied.

All the calculations were performed using an ultrafine integral grid, and geometry optimizations were performed using tight convergence criteria with no symmetry constrains. After optimization, frequency calculations were performed to ensure the presence of positive eigenvalues of the Hessian matrix in the case of minima, while all transition states were characterized as first order transition states (by the presence of a single imaginary frequency). It should be noted that since the geometry difference between the diradical triplet intermediate ^{3}**D*** and the corresponding diradical singlet is expected to be small, the energies of the open-shell singlet intermediate ^{1}**D*** and the transition state **TS IV** are obtained from single point calculations making use of the Broken Symmetry formalism at the corresponding triplet state geometries. This was done because the open-shell singlet intermediate ^{1}**D*** optimization directly leads to the minimum of the potential energy curve of the corresponding closed-shell ^{1}**D*** state. We therefore expect the actual minimum of ^{1}**D*** and **TS IV** to be slightly lower in energy than those reported in **Scheme 3B.**

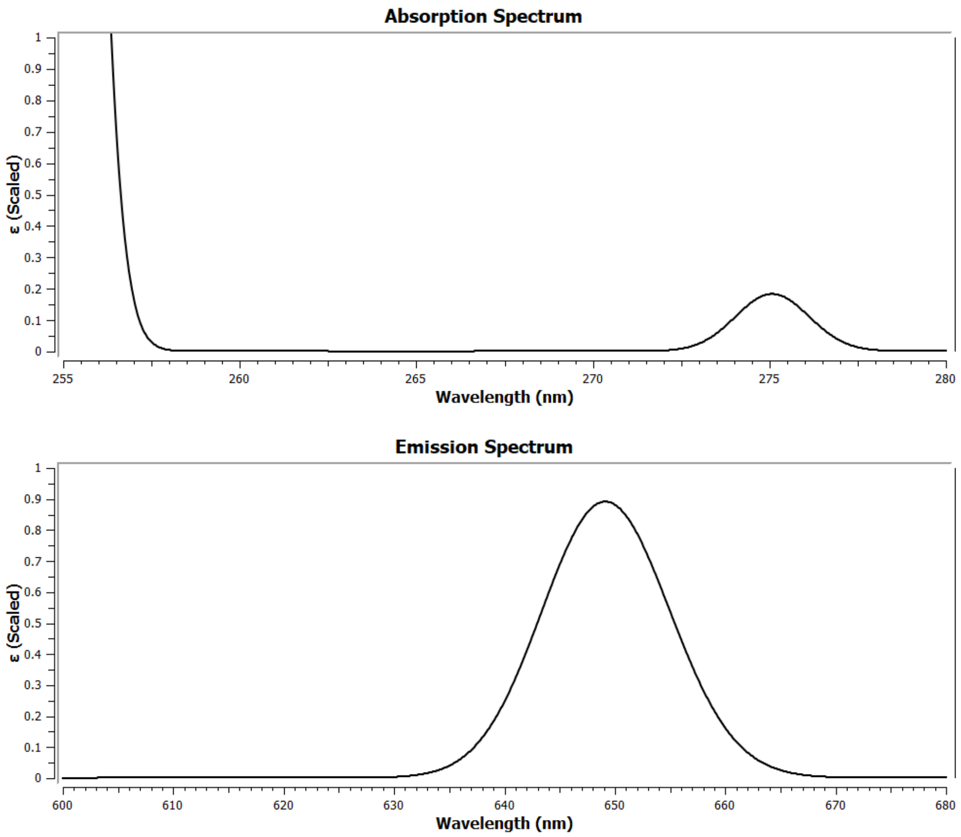

Figure S12. Computed absorption and fluorescence spectra in MeCN (spectrum broadening 0.02 eV).

Diradical Triplet ^{3}A*

In order to evaluate the most plausible geometry of intermediate ^{3}**A***, four different possible conformations have been considered. These geometries, together with their spin densities and Gibbs free energy difference with respect to the most stable structure (^{3}**A***$_{s\text{-}trans\text{-}E}$ set as zero) are reported in **Scheme S10**. The intermediate ^{3}**A***$_{s\text{-}trans\text{-}E}$ is found to be the most energetically stable and is therefore the one considered in the following study of the reaction mechanism. The computed phosphorescence from the ^{3}**A***$_{s\text{-}trans\text{-}E}$ in THF (ε=7.43) is computed at 2.30 eV, which is in good agreement with the experimental value of 2.51 eV recorded in 2-Methyl-THF (ε = 6.97).

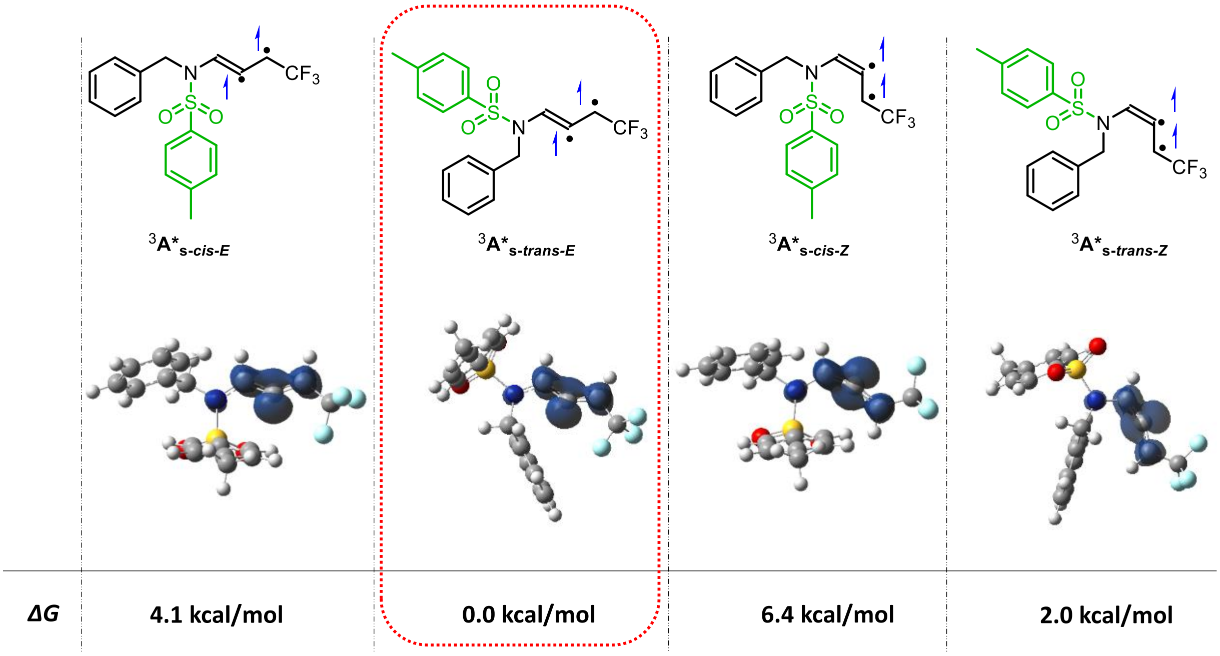

Scheme S10. Four proposed geometries of the diradical triplet intermediate ^{3}**A***. For each, the corresponding spin density (isocontour value 0.01 au) and the ΔG with respect to the most stable intermediate ^{3}**A***$_{s\text{-}trans\text{-}E}$ are reported.

As discussed in the main text, DFT calculations reported in **Scheme 3** show that route II is more favorable compared to route I. The pathway II involves a first shift of the Ts group from the nitrogen to the *β*-carbon to the CF_3- moiety. This transfer would occur as a frontside or a backside shift to afford the ***E*** or ***Z*** isomer intermediate **^{3}D*** through a four-membered transition state **TS III** in **Scheme S11A**. The reaction mechanism for both isomers has been thus computed and is reported in **Scheme S11B**. These results show that the ***E*** isomer of all the intermediates and transition states considered is more stable than the corresponding ***Z*** isomer.

A. Possible shift directions of Ts group: CF_3-substituted *N*-sulfonyl allenamide

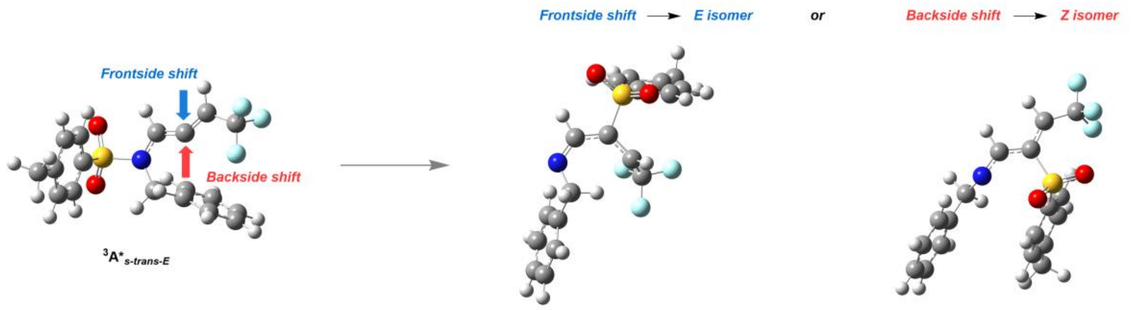

B. Reaction mechanism with frontside (E isomer) and backside (Z isomer) shift of Ts group: CF_3-substituted *N*-sulfonyl allenamide

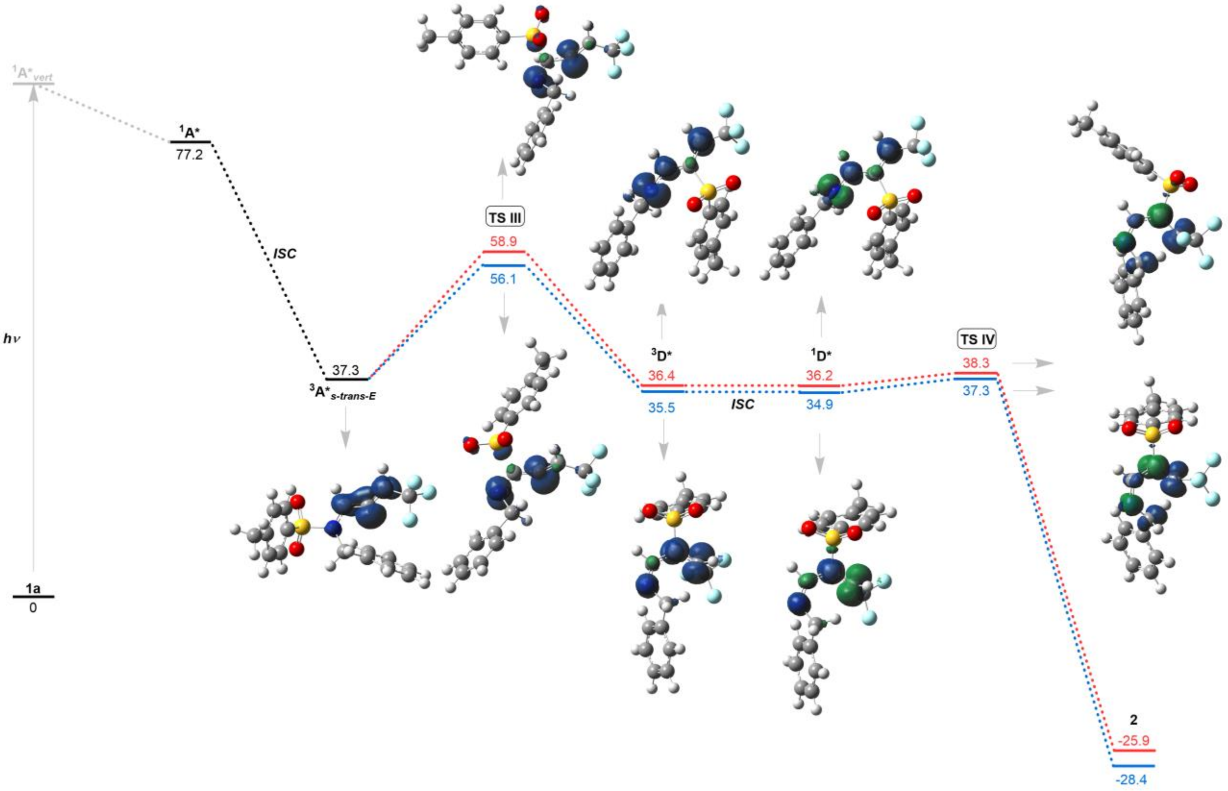

Scheme S11. Possible shift directions of the Ts group and reaction mechanism of route II for frontside (***E*** isomer) and backside shift (***Z*** isomer).

TableS29. SCF energies (Ha), spin expectation value (<S2>) and optimized geometries in cartesian coordinates (Å) of all intermediate presented in Scheme 3B.**

1a

Energy: -1597.24112952

<S**2>=0

Geometry:

C	4.41488300	-0.84788700	1.80845800
C	3.05102200	-0.59590800	1.70191100
C	2.28770200	-1.23454800	0.72618500
C	2.90893200	-2.12345800	-0.14931200
C	4.27384500	-2.37010300	-0.04898900
C	5.03064800	-1.73496900	0.93129400
H	4.99784700	-0.34355200	2.57242500
H	2.57669000	0.10624700	2.38196900
H	2.32115800	-2.61769300	-0.91648900
H	4.74745700	-3.06149900	-0.73865500
H	6.09567100	-1.92803700	1.00882400
C	0.79659200	-0.99737300	0.67742400
H	0.28988800	-1.78846400	1.23526700
H	0.54730800	-0.04177200	1.15414700
N	0.27459900	-1.01906500	-0.68788600
C	0.51472000	0.08938000	-1.50723700
H	0.11141700	0.01740600	-2.51296600
C	1.20708900	1.14259200	-1.15566800
C	1.95120700	2.17559300	-0.88445600
H	3.02655800	2.18961600	-1.05133900
C	1.39619900	3.43442800	-0.30247600
F	0.07285600	3.39564800	-0.11560300
F	1.95820000	3.70948200	0.89209800
F	1.65570400	4.49477600	-1.09343700
S	-1.13553000	-1.89743400	-0.98782400
O	-1.31137600	-1.86029800	-2.43092100
O	-0.96417700	-3.16066400	-0.29058500
C	-2.46698700	-1.01569400	-0.22719000
C	-2.86211400	-1.35592400	1.06317400
C	-3.06735000	0.03543900	-0.91484300
C	-3.87574200	-0.62646800	1.66717200
H	-2.39317800	-2.18613300	1.57870200
C	-4.07794000	0.75187500	-0.29128200
H	-2.76005100	0.28055300	-1.92521800
C	-4.49759400	0.43523700	1.00415500

H	-4.19100700	-0.88957600	2.67203300
H	-4.55242700	1.57080900	-0.82267100
C	-5.61208100	1.19655600	1.65544200
H	-6.57137800	0.70284500	1.46493500
H	-5.47977100	1.24668700	2.73835600
H	-5.68206700	2.21305600	1.26308100

[1]A*

Energy: -1597.18506647

<S**2>=0

Geometry:

C	3.92708900	-2.49682600	1.57454800
C	2.77086900	-1.72277700	1.58102000
C	1.72091400	-2.01658200	0.71325200
C	1.83929100	-3.09411800	-0.16453500
C	2.99938400	-3.86073200	-0.17822500
C	4.04503900	-3.56490300	0.69156700
H	4.73703900	-2.26103400	2.25715000
H	2.68454900	-0.87998800	2.25947200
H	1.02226100	-3.33241700	-0.83785600
H	3.08427800	-4.69496600	-0.86710300
H	4.94830800	-4.16623900	0.68154900
C	0.45945500	-1.18821300	0.76931200
H	-0.40303500	-1.81844700	0.99456700
H	0.54296400	-0.42590100	1.54544100
N	0.17099900	-0.48731900	-0.49207400
C	0.86871100	0.59094800	-0.89721100
H	0.57685600	0.97879200	-1.87340300
C	1.85972200	1.14654600	-0.10810800
C	2.68644700	2.22479600	-0.44377400
H	3.75147900	2.03940500	-0.57809300
C	2.37763100	3.57224900	0.02242500
F	1.07239600	3.90228100	-0.13407600
F	2.62579500	3.80816200	1.35831400
F	3.10553100	4.51141500	-0.62379300
S	-1.20940500	-1.01389300	-1.42836400
O	-1.12239500	-0.28053700	-2.67336300
O	-1.16042500	-2.46079900	-1.39677400
C	-2.57085300	-0.44870400	-0.47258700
C	-3.22362600	-1.34345100	0.37040200
C	-2.96250500	0.88541800	-0.57364000
C	-4.29119700	-0.88326600	1.12630500
H	-2.91096300	-2.37994300	0.42129600
C	-4.02951900	1.32029600	0.19281800
H	-2.44993200	1.56627700	-1.24384300
C	-4.70899800	0.44803600	1.05209000
H	-4.80976500	-1.57287000	1.78421800

H	-4.34501400	2.35615800	0.12229600
C	-5.87948200	0.92981100	1.85145600
H	-6.78688500	0.91268400	1.23773600
H	-6.05641000	0.29600300	2.72215700
H	-5.73340400	1.95880000	2.18716000

^{3}A*$_{s\text{-}trans\text{-}E}$

Energy: -1597.17679407

<S**2>=2.01

Geometry:

C	4.34331300	-0.60768300	1.73929000
C	2.97097400	-0.38969300	1.66840900
C	2.19111100	-1.08928800	0.74964200
C	2.80273200	-2.00689400	-0.10437700
C	4.17449400	-2.21988400	-0.03925300
C	4.94869800	-1.52207800	0.88413800
H	4.94002300	-0.05548700	2.45811500
H	2.50330800	0.33344700	2.33025200
H	2.20248300	-2.55075300	-0.82738800
H	4.64082500	-2.93357900	-0.71082900
H	6.01977500	-1.68900200	0.93386700
C	0.69523200	-0.88744100	0.73477100
H	0.21027300	-1.71425800	1.25854500
H	0.43003600	0.04072600	1.25159800
N	0.15525100	-0.85849200	-0.62633800
C	0.43302100	0.21191400	-1.45002600
H	0.00445100	0.15939200	-2.44886900
C	1.23598200	1.25077500	-1.07853700
C	1.75951500	2.38998300	-1.57582700
H	1.55399600	2.71802500	-2.59473100
C	2.67367400	3.25955000	-0.80547600
F	2.71930000	2.95922600	0.50058400
F	3.95193100	3.19971600	-1.25785900
F	2.32275300	4.56227100	-0.90026100
S	-1.19719600	-1.82246000	-0.98423100
O	-1.35471700	-1.73974400	-2.42619800
O	-0.95243800	-3.09547800	-0.33016700
C	-2.57670700	-1.04264300	-0.20487700
C	-2.97401700	-1.46506600	1.05932000
C	-3.21621500	0.01186500	-0.85315300
C	-4.03275100	-0.81535200	1.67881600
H	-2.47270000	-2.29539900	1.54311700
C	-4.26964200	0.64630700	-0.21507600
H	-2.90407000	0.32113500	-1.84426100
C	-4.69436400	0.24572700	1.05675300
H	-4.35115600	-1.14228100	2.66353200

H	-4.77553200	1.46620300	-0.71526000
C	-5.85283800	0.92610100	1.72026300
H	-6.79758600	0.57143000	1.29427100
H	-5.87406300	0.72327300	2.79245300
H	-5.81537300	2.00765500	1.56904800

TS I

Energy: -1597.12121939

<S**2>=2.01

Geometry:

C	-0.38051900	-1.82590900	-4.66221900
C	-0.81474000	-0.77375300	-3.86753200
C	-0.78101600	-0.87427400	-2.47044500
C	-0.28673700	-2.04772400	-1.89024100
C	0.14417800	-3.09922000	-2.68873300
C	0.10066500	-2.99401100	-4.07652400
H	-0.42201000	-1.73562400	-5.74284700
H	-1.19574600	0.13250500	-4.32957200
H	-0.24846700	-2.14567700	-0.80999600
H	0.51589100	-4.00683300	-2.22418900
H	0.43886400	-3.81711700	-4.69714200
C	-1.21207400	0.29311200	-1.67297700
H	-1.96015200	0.91548800	-2.16125700
N	-1.50646500	0.10728200	-0.28621100
C	-0.50777700	0.21504800	0.66280500
H	-0.82027400	0.07456600	1.69173500
C	0.74450500	0.54151100	0.31640900
C	2.04148100	2.27078500	-0.94758500
F	1.44205800	3.28627700	-0.29763400
F	3.24779700	2.10581200	-0.34771900
F	2.30148500	2.69765400	-2.19689300
S	-3.14238200	-0.10060200	0.21041800
O	-3.13899300	0.07908700	1.65167200
O	-3.91861200	0.77018600	-0.65388300
C	-3.52490900	-1.77703800	-0.17496500
C	-4.06925500	-2.08120000	-1.41825800
C	-3.24345700	-2.76878700	0.76138900
C	-4.33381900	-3.40883600	-1.72220400
H	-4.28926100	-1.29374000	-2.12962800
C	-3.51557300	-4.08733700	0.43537800
H	-2.82719100	-2.51045600	1.72846000
C	-4.06169800	-4.42880300	-0.80701900
H	-4.76048700	-3.65599200	-2.68902500
H	-3.30259600	-4.86736900	1.15956700
C	-4.37447800	-5.85720200	-1.13311200
H	-5.33559100	-6.14512500	-0.69318000

H	-4.44300300	-6.01462300	-2.21104500
H	-3.61668600	-6.53116700	-0.72699600
C	1.21347100	1.03690600	-0.96111300
H	1.63118300	0.29206200	-1.64425600
H	-0.07169500	1.00838500	-1.55065900

^{3}B*

Energy: -1597.17142494

<S**2>=2.05

Geometry:

C	3.99611400	-1.47879100	2.33718000
C	2.64731100	-1.38373600	2.05088000
C	2.18525800	-1.39869200	0.71130300
C	3.14565800	-1.52550100	-0.32185600
C	4.49241400	-1.62383500	-0.01997600
C	4.93068400	-1.59545500	1.30541100
H	4.32800900	-1.46408500	3.37034000
H	1.92494200	-1.29454500	2.85682100
H	2.81899500	-1.56348400	-1.35459100
H	5.21347800	-1.72801000	-0.82455500
H	5.98881600	-1.66954800	1.53230400
C	0.79399300	-1.31118500	0.47609900
H	0.10024100	-1.36927500	1.30874900
N	0.24196900	-1.17760900	-0.80476600
C	0.54707200	-0.07992800	-1.63748100
H	0.14702600	-0.16955400	-2.64766200
C	1.28321200	0.94993100	-1.27838300
C	1.42793800	3.42589800	-1.32230300
F	1.72732500	3.51675300	-0.01573700
F	2.01786500	4.46718100	-1.93379200
F	0.10162800	3.60570200	-1.43266600
S	-1.24766200	-1.95984900	-1.07340300
O	-1.49231600	-1.84811700	-2.50214300
O	-1.12769300	-3.25689900	-0.43245300
C	-2.45768500	-1.01129600	-0.20285900
C	-2.86341700	-1.42039300	1.06282800
C	-2.95354600	0.15486500	-0.78280600
C	-3.78320200	-0.64307100	1.75424200
H	-2.47656000	-2.33763300	1.49160800
C	-3.86865900	0.91564300	-0.07376500
H	-2.63888900	0.45471300	-1.77601700
C	-4.29755800	0.53155400	1.20200400
H	-4.10767100	-0.95853700	2.74074100
H	-4.26153400	1.82392700	-0.51989400
C	-5.30757400	1.35382300	1.94320900
H	-6.31073100	1.18106700	1.53884000

H	-5.32741300	1.09976300	3.00445100
H	-5.09778900	2.42159400	1.84283500
C	1.88351500	2.11713000	-1.92463300
H	1.62840300	2.14433200	-2.99329300
H	2.97670700	2.09657000	-1.85181500

TS II

Energy: -1597.16699801

<S**2>=2.00

Geometry:

C	0.32921000	-5.01501800	2.57601700
C	-0.00790100	-3.69258500	2.31281200
C	0.61821300	-2.99763000	1.27448000
C	1.58896700	-3.64296900	0.49911700
C	1.92318600	-4.96127600	0.76344700
C	1.29430300	-5.64958300	1.80177500
H	-0.16056100	-5.54895800	3.38326700
H	-0.76141500	-3.19231900	2.91435100
H	2.07117800	-3.09739900	-0.30464300
H	2.67581100	-5.45959900	0.16145300
H	1.55956200	-6.68200000	2.00497200
C	0.23409300	-1.60698500	1.03042300
H	-0.53189000	-1.20242800	1.70478600
N	0.75532400	-0.90390200	0.10585800
C	0.32647400	0.46560100	-0.03939600
H	0.91622900	1.12576800	0.61060600
C	-1.09161900	0.75200700	0.06746900
C	-3.56838600	0.41881000	0.29250300
F	-3.36101300	1.18247800	1.37523800
F	-4.48185600	1.05542400	-0.46033700
F	-4.14904100	-0.71438200	0.72461400
S	0.94362800	0.96073500	-1.75244600
O	2.39903900	0.79615000	-1.78059300
O	0.12242900	0.25309100	-2.74119700
C	0.56555200	2.69179700	-1.84278000
C	-0.59530200	3.10523800	-2.48586600
C	1.42802200	3.60784200	-1.24629800
C	-0.89276400	4.46065300	-2.52837500
H	-1.24599800	2.37799900	-2.95816400
C	1.11060300	4.95641800	-1.29539500
H	2.33804800	3.27243300	-0.76081900
C	-0.05017200	5.40386200	-1.93561900
H	-1.79577600	4.79060900	-3.03226300
H	1.77817600	5.67654200	-0.83247700
C	-0.36394000	6.86768900	-2.00572500
H	0.21978300	7.34450100	-2.80062100

H	-1.42015100	7.03949200	-2.22102500
H	-0.11053100	7.37197700	-1.07020100
C	-2.29334500	0.12917500	-0.49754100
H	-2.17598100	-0.96144100	-0.54502500
H	-2.46446600	0.46369200	-1.52901000

^{3}C*

Energy: -1597.21246382

<S**2>=2.04

Geometry:

N	1.48014600	-0.21750800	0.24605700
C	2.30413200	-1.15115900	0.83537100
H	1.94575900	-1.71176200	1.70062600
C	3.60151000	-1.38709900	0.34574600
C	4.43164200	-2.34862200	0.98372700
C	4.12722500	-0.69082600	-0.77690700
C	5.70760900	-2.59726100	0.52197400
H	4.04623000	-2.88870400	1.84338400
C	5.40648800	-0.95129700	-1.22659000
H	3.50390700	0.04437800	-1.27229300
C	6.20755500	-1.90218300	-0.58568600
H	6.32691700	-3.33576300	1.02065200
H	5.79372300	-0.41289600	-2.08579900
H	7.21143800	-2.10077100	-0.94533000
C	0.37775900	0.09040300	0.83325000
H	0.07568900	-0.35141100	1.78821600
C	-0.52329000	1.05960300	0.22857300
S	-2.19506300	1.08202000	0.80394400
O	-2.87553200	2.18975800	0.13521800
O	-2.14303100	1.02672200	2.26672400
C	-2.89213100	-0.43829200	0.22457700
C	-3.47201200	-0.48472100	-1.04213200
C	-2.82278800	-1.57279500	1.03013500
C	-3.98833900	-1.68681900	-1.49803800
H	-3.52767600	0.40968700	-1.65234500
C	-3.34470600	-2.76586700	0.55362600
H	-2.38370500	-1.51570400	2.01971800
C	-3.93300800	-2.84351000	-0.71229000
H	-4.44546100	-1.72957300	-2.48165500
H	-3.29830400	-3.65272600	1.17768200
C	-4.52004600	-4.12986300	-1.20645100
H	-5.58669500	-4.17827200	-0.96047700
H	-4.43130200	-4.21639400	-2.29148100
H	-4.03643000	-4.99240300	-0.74377500
C	-0.12531400	1.90935400	-0.92002700
H	-0.99017900	2.19968700	-1.51966700

H	0.58566100	1.35411200	-1.54042300
C	0.56744200	3.19415200	-0.50769400
F	-0.22287800	3.98523700	0.23410500
F	1.67995300	2.97456800	0.20843700
F	0.92743100	3.89994000	-1.59199500

2

Energy: -1597.28827305

<S**2>=0

Geometry:

N	1.84718600	-0.19671100	0.27617400
C	2.81503200	-0.79665700	0.87459100
H	2.69879500	-1.17486700	1.89903400
C	4.11258000	-1.01227500	0.25869600
C	5.10303600	-1.67831500	0.99068900
C	4.39399300	-0.57232800	-1.04282700
C	6.35448000	-1.90357000	0.43311400
H	4.88575500	-2.01794400	1.99908700
C	5.64299500	-0.79912900	-1.59531800
H	3.62249700	-0.05589900	-1.60293300
C	6.62459200	-1.46462600	-0.85919200
H	7.11821400	-2.42000900	1.00433400
H	5.85973500	-0.45861100	-2.60225500
H	7.60209000	-1.63929700	-1.29673700
C	0.66573000	-0.09289800	0.97325000
H	0.55971400	-0.60385200	1.93257500
C	-0.37656000	0.61062700	0.49130000
S	-1.88273400	0.55923500	1.43115600
O	-2.48108200	1.89492400	1.44607000
O	-1.59861200	-0.10773400	2.70440400
C	-2.94944900	-0.49037900	0.47095800
C	-3.94663100	0.08058400	-0.30892800
C	-2.76167100	-1.87079200	0.50733600
C	-4.77117300	-0.74940200	-1.06024100
H	-4.08351200	1.15608200	-0.31447000
C	-3.59238400	-2.68041300	-0.24837100
H	-1.98211200	-2.30388700	1.12491600
C	-4.60823200	-2.13480900	-1.04390500
H	-5.55545000	-0.30897100	-1.66779300
H	-3.45458500	-3.75701100	-0.22074500
C	-5.50190600	-3.02983200	-1.84814200
H	-6.10185300	-3.66761000	-1.19125900
H	-6.18296800	-2.45379000	-2.47669100
H	-4.91517000	-3.69221700	-2.49108900
C	-0.38602600	1.32226800	-0.82541000
H	-1.39098100	1.37609100	-1.25177100

H	0.24731400	0.77165900	-1.52529000
C	0.15415000	2.73531100	-0.79128800
F	-0.59000900	3.57406600	-0.05765100
F	1.40285900	2.79993800	-0.30398800
F	0.19472500	3.24173500	-2.03972600

TS III

Energy: -1597.14490149

<S**2>=2.00

Geometry:

C	0.44965400	-5.57886700	1.21045800
C	-0.14229000	-4.33859300	0.98870200
C	0.56221400	-3.16219900	1.23748400
C	1.87237100	-3.24438600	1.71081900
C	2.46687700	-4.48157200	1.92920100
C	1.75626700	-5.65316000	1.68035100
H	-0.10990600	-6.48681100	1.00957600
H	-1.16129600	-4.28633500	0.61539200
H	2.42936900	-2.33252600	1.90368500
H	3.48721600	-4.53226300	2.29579000
H	2.22052600	-6.61903800	1.85122100
C	-0.09570700	-1.82488400	1.03232400
H	-1.04062600	-1.94392800	0.47849400
H	-0.39423000	-1.39958900	2.00627700
N	0.75715400	-0.89849600	0.34549800
C	0.22870800	0.39447700	0.11524900
H	0.91807500	1.14849900	0.52368400
C	-1.14083200	0.67392600	0.48502600
C	-2.37490400	0.79883100	0.07921700
H	-2.66197100	0.70439200	-0.96998200
C	-3.50507800	1.07452400	1.01862700
F	-3.11857700	1.16546000	2.29499300
F	-4.12576700	2.22667600	0.70492500
F	-4.43744600	0.10655800	0.94991100
S	0.51099400	0.72579000	-1.78454100
O	1.90257200	0.38408200	-2.07159800
O	-0.57132200	0.05150900	-2.50527300
C	0.30878300	2.47836400	-1.94994900
C	-0.88674200	2.98607400	-2.44519500
C	1.35202900	3.31715700	-1.56573000
C	-1.03293400	4.36132900	-2.55791800
H	-1.68078000	2.31513600	-2.75166800
C	1.18160700	4.68794900	-1.67892700
H	2.28590500	2.90671000	-1.19682800
C	-0.00808800	5.23084500	-2.17629000
H	-1.96096700	4.76584500	-2.94927000

H	1.98903700	5.34947700	-1.38139300
C	-0.16561300	6.71344900	-2.32411900
H	0.25999300	7.04635100	-3.27716000
H	-1.21741900	7.00553600	-2.31266000
H	0.35702500	7.24957600	-1.52893200

^{3}D*

Energy: -1597.18062726

<S**2>=2.04

Geometry:

C	3.31663400	-4.75142200	1.25296200
C	2.88072600	-3.61634200	0.57724400
C	1.86519000	-2.82285900	1.11073800
C	1.29340400	-3.18419100	2.33163500
C	1.73272800	-4.31467900	3.01069700
C	2.74600200	-5.10275200	2.47223500
H	4.10544600	-5.36231000	0.82566300
H	3.32812000	-3.34776200	-0.37460600
H	0.49710100	-2.57680200	2.75290300
H	1.27826100	-4.58449600	3.95857000
H	3.08708000	-5.98775400	2.99944700
C	1.41040800	-1.56723000	0.41494900
H	0.35031200	-1.39404000	0.64878300
H	1.94542300	-0.69182300	0.82962100
N	1.50337700	-1.65137800	-1.01134600
C	1.98251600	-0.66953600	-1.72710200
H	1.86242300	-0.76948900	-2.80546000
C	3.10864300	0.89185000	-0.00279100
H	2.59916900	1.68131900	0.54142200
C	4.39412800	0.42898800	0.58869700
F	4.99804900	-0.50198100	-0.15975800
F	5.26729400	1.44348100	0.74882000
F	4.21118400	-0.09825300	1.81620800
C	2.66008900	0.49790600	-1.31848000
S	3.00840400	1.68593700	-2.64170700
O	2.35163400	1.20407300	-3.85615600
O	2.65790600	2.99936700	-2.10172100
C	4.75954800	1.62610200	-2.88075500
C	5.56406200	2.57480300	-2.25939700
C	5.30273300	0.62045700	-3.67862500
C	6.93772800	2.51182000	-2.44708800
H	5.11918200	3.35336200	-1.65076000
C	6.67609400	0.57396300	-3.84868900
H	4.66022800	-0.10605700	-4.16354500
C	7.51381500	1.51520600	-3.23825000
H	7.57297100	3.25082500	-1.96930400

H	7.10864700	-0.20491700	-4.46874100
C	8.99527800	1.46689100	-3.45514600
H	9.25392500	1.93562900	-4.41097100
H	9.53143700	2.00056400	-2.66829600
H	9.35699300	0.43667900	-3.49057700

^{1}D*

Energy: -1597.18232705

<S**2>=1.02

Geometry:

C	3.31663400	-4.75142200	1.25296200
C	2.88072600	-3.61634200	0.57724400
C	1.86519000	-2.82285900	1.11073800
C	1.29340400	-3.18419100	2.33163500
C	1.73272800	-4.31467900	3.01069700
C	2.74600200	-5.10275200	2.47223500
H	4.10544600	-5.36231000	0.82566300
H	3.32812000	-3.34776200	-0.37460600
H	0.49710100	-2.57680200	2.75290300
H	1.27826100	-4.58449600	3.95857000
H	3.08708000	-5.98775400	2.99944700
C	1.41040800	-1.56723000	0.41494900
H	0.35031200	-1.39404000	0.64878300
H	1.94542300	-0.69182300	0.82962100
N	1.50337700	-1.65137800	-1.01134600
C	1.98251600	-0.66953600	-1.72710200
H	1.86242300	-0.76948900	-2.80546000
C	3.10864300	0.89185000	-0.00279100
H	2.59916900	1.68131900	0.54142200
C	4.39412800	0.42898800	0.58869700
F	4.99804900	-0.50198100	-0.15975800
F	5.26729400	1.44348100	0.74882000
F	4.21118400	-0.09825300	1.81620800
C	2.66008900	0.49790600	-1.31848000
S	3.00840400	1.68593700	-2.64170700
O	2.35163400	1.20407300	-3.85615600
O	2.65790600	2.99936700	-2.10172100
C	4.75954800	1.62610200	-2.88075500
C	5.56406200	2.57480300	-2.25939700
C	5.30273300	0.62045700	-3.67862500
C	6.93772800	2.51182000	-2.44708800
H	5.11918200	3.35336200	-1.65076000
C	6.67609400	0.57396300	-3.84868900
H	4.66022800	-0.10605700	-4.16354500
C	7.51381500	1.51520600	-3.23825000
H	7.57297100	3.25082500	-1.96930400

H	7.10864700	-0.20491700	-4.46874100
C	8.99527800	1.46689100	-3.45514600
H	9.25392500	1.93562900	-4.41097100
H	9.53143700	2.00056400	-2.66829600
H	9.35699300	0.43667900	-3.49057700

TS IV

Energy: -1597.17623658

<S**2>=0.96

Geometry:

N	1.63585400	-0.02572900	0.04354300
C	1.44542500	-1.09661500	-0.86298200
H	1.93677700	-0.87658000	-1.81769900
C	1.77753100	-2.44947800	-0.35660400
C	2.17726300	-3.44345300	-1.25690800
C	1.64717900	-2.77220200	0.99829000
C	2.44120100	-4.73172900	-0.81169500
H	2.28358200	-3.20108600	-2.31029100
C	1.91733400	-4.06079000	1.44153200
H	1.34258600	-2.00856900	1.70689500
C	2.31233500	-5.04490700	0.53929100
H	2.75340000	-5.49267500	-1.51949700
H	1.81928100	-4.29830100	2.49587900
H	2.52209900	-6.05083200	0.88767100
C	0.64327600	0.75826200	0.35739900
H	0.85211800	1.57994300	1.03870100
C	-0.68423300	0.63248100	-0.11515600
S	-1.85811500	1.85436900	0.49662400
O	-2.56962300	2.39539700	-0.65978100
O	-1.13092000	2.76191400	1.38420500
C	-2.98789700	0.89296800	1.45998200
C	-4.12682900	0.37221900	0.85418000
C	-2.69929800	0.64778600	2.80073900
C	-4.99286900	-0.40066200	1.61465600
H	-4.33398900	0.57922500	-0.18954100
C	-3.57764500	-0.12672400	3.54108900
H	-1.80718600	1.06487000	3.25448400
C	-4.73484200	-0.66120600	2.96324100
H	-5.88642300	-0.80775600	1.15221800
H	-3.36363900	-0.31918300	4.58774500
C	-5.68891800	-1.47396500	3.78395700
H	-6.32619900	-2.09737400	3.15417800
H	-5.15710200	-2.11574500	4.49018000
H	-6.34082200	-0.81635500	4.36939500
C	-1.12576600	-0.45777300	-0.97469400
H	-1.65122800	-1.28522200	-0.49698400

H	0.23889500	-1.06917100	-1.13462700
C	-1.58701600	-0.21372300	-2.36991100
F	-2.90593700	0.05865000	-2.47293900
F	-0.93139100	0.79865600	-2.95718500
F	-1.38516400	-1.31262000	-3.12801300

Table S30.SCF energies (Ha), spin expectations (<S2>) and optimized geometries in cartesian coordinates (Å) of the two proposed mechanisms in Scheme 4C.**

7a

Energy: -1260.40583997

<S**2>=0

Geometry:

C	4.434189	-0.873442	1.793774
C	3.065891	-0.643166	1.694159
C	2.311460	-1.272440	0.705305
C	2.947729	-2.130333	-0.190150
C	4.316884	-2.356102	-0.096489
C	5.064371	-1.730196	0.896798
H	5.009356	-0.375785	2.568053
H	2.580622	0.035747	2.389976
H	2.366528	-2.616044	-0.967615
H	4.801349	-3.023718	-0.801993
H	6.132689	-1.906573	0.969069
C	0.816310	-1.061070	0.663450
H	0.328606	-1.866747	1.217472
H	0.554542	-0.114203	1.150784
N	0.292231	-1.084021	-0.700338
C	0.492979	0.063921	-1.501846
H	0.151669	-0.036071	-2.526952
C	1.088097	1.157568	-1.096002
S	-1.130721	-1.937900	-0.971859
O	-1.321631	-1.928571	-2.414753
O	-0.984488	-3.196399	-0.256540
C	-2.450685	-1.024883	-0.223819
C	-2.842571	-1.330519	1.076380
C	-3.041801	0.019773	-0.928905
C	-3.843457	-0.575814	1.670583
H	-2.380813	-2.155148	1.607209
C	-4.040057	0.762579	-0.315240
H	-2.736566	0.238865	-1.945770
C	-4.456639	0.478968	0.988443
H	-4.156139	-0.813315	2.682692
H	-4.507000	1.576278	-0.861312
C	-5.558579	1.266889	1.629946
H	-6.522620	0.770891	1.472165
H	-5.409220	1.353969	2.708409

H	-5.631118	2.269656	1.204136
C	1.733907	2.238024	-0.762344
H	1.217257	3.105396	-0.357845
H	2.814155	2.310203	-0.870872

[1]E*

Energy: -1260.29503269

<S**2>=0

Geometry:

C	4.314506	-0.455764	1.619919
C	2.929637	-0.332169	1.552302
C	2.186186	-1.161601	0.715518
C	2.847024	-2.111400	-0.063406
C	4.229667	-2.232132	-0.001634
C	4.967442	-1.405573	0.842930
H	4.882686	0.196988	2.274805
H	2.423358	0.419512	2.151327
H	2.273548	-2.753718	-0.725119
H	4.733930	-2.973783	-0.612831
H	6.047369	-1.500728	0.890994
C	0.681673	-1.053069	0.688898
H	0.244536	-1.928300	1.173601
H	0.357484	-0.160435	1.235167
N	0.165276	-1.008929	-0.682824
C	0.386155	0.139735	-1.434123
H	-0.016092	0.118572	-2.444476
C	1.127761	1.187809	-0.965550
C	1.613145	2.384616	-1.317286
H	1.409786	2.820901	-2.298768
S	-1.229194	-1.903057	-1.024742
O	-1.398051	-1.817965	-2.466602
O	-1.039979	-3.190358	-0.376579
C	-2.577004	-1.070193	-0.241224
C	-2.988505	-1.476428	1.024629
C	-3.167940	0.017051	-0.880322
C	-4.012444	-0.780674	1.651173
H	-2.523551	-2.330541	1.503427
C	-4.187158	0.699978	-0.233872
H	-2.846229	0.314711	-1.871814
C	-4.626038	0.314111	1.036415
H	-4.341963	-1.096170	2.636138
H	-4.654018	1.546640	-0.727395
C	-5.751473	1.039659	1.709496
H	-6.713255	0.603865	1.417266
H	-5.676452	0.970105	2.796606

H	-5.770805	2.094083	1.425784
H	2.224311	2.975051	-0.639250

^{3}E*$_{s\text{-}trans\text{-}E}$

Energy: -1260.33880326

<S**2>=2.01

Geometry:

C	4.325328	-0.567309	1.722810
C	2.945498	-0.404802	1.644793
C	2.197317	-1.139277	0.726759
C	2.849390	-2.035567	-0.119755
C	4.228435	-2.193584	-0.047972
C	4.970633	-1.461042	0.874880
H	4.896442	0.011957	2.441221
H	2.446092	0.302244	2.301234
H	2.273039	-2.604493	-0.842948
H	4.725758	-2.891540	-0.713947
H	6.047292	-1.584997	0.929959
C	0.694966	-0.998040	0.701980
H	0.243122	-1.854387	1.207677
H	0.391818	-0.089863	1.234573
N	0.167921	-0.970666	-0.664064
C	0.396006	0.158275	-1.446350
H	-0.005091	0.109723	-2.456657
C	1.127268	1.228303	-1.015921
C	1.614778	2.411445	-1.438829
H	1.403674	2.780234	-2.444534
S	-1.213373	-1.888593	-0.996104
O	-1.383909	-1.822459	-2.438957
O	-1.010379	-3.166141	-0.332458
C	-2.573479	-1.065047	-0.222474
C	-2.976385	-1.458931	1.050054
C	-3.184530	0.001214	-0.877435
C	-4.010810	-0.771127	1.667973
H	-2.496603	-2.297861	1.540913
C	-4.214560	0.676193	-0.239842
H	-2.869212	0.289375	-1.873860
C	-4.644102	0.303392	1.037507
H	-4.332860	-1.077102	2.658442
H	-4.697025	1.506599	-0.745767
C	-5.779834	1.020715	1.702090
H	-6.733378	0.550685	1.437153
H	-5.689344	0.987902	2.789825

H	-5.830438	2.064651	1.385570
H	2.225865	3.040320	-0.798963

TS V

Energy: -1260.31171794

<S**2>=2.02

Geometry:

C	0.328555	-5.578453	1.345629
C	-0.227567	-4.318468	1.146077
C	0.560434	-3.171669	1.236030
C	1.918212	-3.306033	1.526348
C	2.476866	-4.564206	1.722529
C	1.683460	-5.704752	1.633758
H	-0.297070	-6.462008	1.268967
H	-1.285274	-4.227096	0.914474
H	2.539192	-2.418310	1.592131
H	3.535254	-4.654620	1.945834
H	2.119900	-6.686533	1.786438
C	-0.066430	-1.813627	1.064722
H	-1.025775	-1.897294	0.529685
H	-0.340426	-1.402508	2.052607
N	0.791132	-0.888274	0.388720
C	0.261144	0.405450	0.162792
H	0.938792	1.152750	0.603847
C	-1.122239	0.679773	0.479622
C	-2.339938	0.839109	0.040589
H	-2.576097	0.799038	-1.025722
S	0.648064	0.753003	-1.718189
O	2.073869	0.488666	-1.922443
O	-0.343464	0.026328	-2.517084
C	0.371565	2.495523	-1.911487
C	-0.803925	2.942638	-2.502420
C	1.339587	3.386968	-1.456460
C	-1.006113	4.308625	-2.641669
H	-1.539767	2.231127	-2.858805
C	1.113970	4.747621	-1.596198
H	2.260365	3.024509	-1.012331
C	-0.056773	5.229377	-2.191360
H	-1.919075	4.665390	-3.108020
H	1.863266	5.449201	-1.243125
C	-0.271950	6.701955	-2.366848
H	0.226704	7.052692	-3.277094
H	-1.333045	6.941473	-2.458552

H	0.144463	7.266441	-1.529555
H	-3.172768	1.014967	0.719033

^{3}F*

Energy: -1260.34886265

<S**2>=2.04

Geometry:

C	3.332359	-4.760206	1.145376
C	2.910669	-3.588124	0.526344
C	1.958016	-2.768727	1.133099
C	1.435834	-3.144581	2.371373
C	1.859976	-4.313480	2.994315
C	2.809951	-5.125807	2.382353
H	4.071916	-5.389409	0.660184
H	3.319591	-3.308866	-0.439845
H	0.688463	-2.518544	2.851142
H	1.442607	-4.593423	3.956335
H	3.138922	-6.040204	2.865340
C	1.530543	-1.473876	0.492637
H	0.527909	-1.210324	0.856943
H	2.196668	-0.657095	0.825598
N	1.443237	-1.571613	-0.938261
C	1.875088	-0.614289	-1.716157
H	1.685057	-0.753229	-2.779925
C	3.067324	1.039005	-0.117026
H	2.549498	1.827934	0.418588
C	2.568945	0.568838	-1.385701
S	2.933390	1.656777	-2.785304
O	2.337170	1.089239	-3.996852
O	2.557290	3.010848	-2.372094
C	4.696568	1.589838	-2.939314
C	5.478505	2.491740	-2.225112
C	5.271311	0.609463	-3.743961
C	6.860020	2.407437	-2.327941
H	5.011441	3.253259	-1.610963
C	6.652880	0.543433	-3.833564
H	4.644559	-0.082055	-4.295911
C	7.467292	1.437394	-3.129948
H	7.477449	3.109659	-1.776670
H	7.108895	-0.215158	-4.462062
C	8.958850	1.371582	-3.257966
H	9.287588	1.914626	-4.150898
H	9.453416	1.823570	-2.396066

H	9.303500	0.340021	-3.359490
H	4.043455	0.724457	0.241067

TS VI

Energy: -2140.22740063

<S**2>=2.04

Geometry:

C	5.317692	-3.460103	-1.611143
C	4.018405	-3.005466	-1.818118
C	3.271701	-2.488913	-0.760193
C	3.852536	-2.434313	0.508243
C	5.147308	-2.894034	0.718524
C	5.885556	-3.408303	-0.343167
H	5.886837	-3.853109	-2.447418
H	3.600198	-3.047377	-2.818520
H	3.285136	-2.025885	1.340153
H	5.582418	-2.842534	1.711422
H	6.898600	-3.762924	-0.183490
C	1.838677	-2.035148	-0.914154
H	1.154372	-2.864091	-0.691091
H	1.609776	-1.290019	-0.136765
C	2.221500	-0.773150	-3.021021
H	2.241232	-1.107028	-4.056629
C	2.907555	1.098905	-1.496867
H	3.470443	2.018184	-1.405523
C	2.870478	0.417456	-2.692668
S	3.821634	1.117080	-4.048363
O	3.112560	0.825769	-5.298906
O	4.128255	2.508418	-3.706289
C	5.352986	0.212913	-4.081190
C	6.391384	0.596963	-3.239353
C	5.483187	-0.872830	-4.939575
C	7.576337	-0.124214	-3.263976
H	6.276834	1.450984	-2.581051
C	6.678618	-1.578226	-4.953307
H	4.665595	-1.152282	-5.594916
C	7.740119	-1.218421	-4.118931
H	8.390537	0.169667	-2.608641
H	6.787883	-2.424456	-5.624787
C	9.036548	-1.970235	-4.158883
H	9.727723	-1.501158	-4.867851
H	9.524317	-1.973985	-3.181587
H	8.888139	-3.003377	-4.480031

H	2.348265	0.749537	-0.638381
N	1.414989	-1.469637	-2.172258
H	0.710205	-2.549293	-3.012254
O	-1.340240	-2.887880	-4.397530
P	0.011306	-3.440930	-4.028012
C	-0.045377	-5.081728	-3.266969
C	0.965997	-5.546943	-2.422794
C	-1.155792	-5.885604	-3.535913
C	0.874077	-6.818350	-1.868552
H	1.825296	-4.926322	-2.186519
C	-1.240957	-7.155640	-2.978970
H	-1.948277	-5.512429	-4.176376
C	-0.226957	-7.622158	-2.147691
H	1.659857	-7.177668	-1.212742
H	-2.102719	-7.779802	-3.190488
H	-0.297530	-8.612986	-1.711191
C	1.166397	-3.476661	-5.426769
C	0.975883	-2.531917	-6.439782
C	2.240637	-4.367503	-5.497232
C	1.856496	-2.479645	-7.513501
H	0.134870	-1.848059	-6.386766
C	3.117352	-4.309162	-6.573851
H	2.389773	-5.114997	-4.725167
C	2.927857	-3.365290	-7.579337
H	1.704370	-1.747938	-8.299840
H	3.946246	-5.006799	-6.630728
H	3.614397	-3.323528	-8.418575

G + [P(O)·]

Energy: -2140.28125868

<S**2>=2.03

Geometry:

C	4.174924	-3.527894	-0.018256
C	3.176002	-2.779427	-0.635584
C	2.402573	-1.887890	0.103278
C	2.644353	-1.757695	1.472302
C	3.637669	-2.507313	2.091159
C	4.408856	-3.395597	1.345896
H	4.772456	-4.216011	-0.608071
H	3.002004	-2.885834	-1.701583
H	2.050765	-1.061092	2.059006
H	3.813946	-2.393934	3.156170
H	5.188373	-3.978075	1.826196
C	1.284453	-1.079336	-0.525787
H	0.325751	-1.521687	-0.238037
H	1.282534	-0.066165	-0.111880
C	2.115353	-0.309952	-2.756294
H	1.928639	-0.461721	-3.813810
C	3.637665	1.019711	-1.197733
H	4.406866	1.780035	-1.173990
C	3.119870	0.591965	-2.418278
S	3.960543	1.246042	-3.854913
O	3.077381	1.135060	-5.024007
O	4.506967	2.564328	-3.511374
C	5.344890	0.158889	-4.136316
C	6.573385	0.449768	-3.555726
C	5.168060	-0.983255	-4.910650
C	7.637822	-0.419352	-3.759125
H	6.695686	1.348613	-2.961974
C	6.243171	-1.838246	-5.104647
H	4.205884	-1.195093	-5.364502
C	7.492167	-1.571953	-4.534574
H	8.600089	-0.195132	-3.308856
H	6.110237	-2.728691	-5.711928
C	8.654011	-2.488704	-4.775928
H	9.166155	-2.220255	-5.706544
H	9.385996	-2.425395	-3.968066
H	8.327865	-3.526886	-4.871449

H	3.303582	0.605223	-0.257648
N	1.294795	-1.023502	-1.966897
H	0.677118	-1.670624	-2.458633
O	-0.248550	-2.912405	-3.471325
P	0.382099	-4.072088	-4.221381
C	-0.808342	-5.344975	-4.684357
C	-0.615672	-6.181345	-5.789053
C	-1.922899	-5.524373	-3.856646
C	-1.542154	-7.177628	-6.068354
H	0.241963	-6.047564	-6.439391
C	-2.844930	-6.520604	-4.146798
H	-2.068780	-4.875661	-2.999297
C	-2.654938	-7.349070	-5.249808
H	-1.397156	-7.818061	-6.931877
H	-3.714000	-6.650792	-3.510653
H	-3.376043	-8.128469	-5.472523
C	1.359856	-3.568263	-5.653831
C	1.111347	-2.324649	-6.243825
C	2.392371	-4.380003	-6.134067
C	1.876450	-1.912951	-7.327664
H	0.319492	-1.690471	-5.859023
C	3.150534	-3.960520	-7.220295
H	2.613502	-5.329472	-5.655992
C	2.892395	-2.729742	-7.816954
H	1.679599	-0.951807	-7.790721
H	3.949701	-4.591277	-7.594684
H	3.489634	-2.402486	-8.661576

^{3}8a*

Energy: -2140.28502982

<S**2>=2.01

Geometry:

C	3.546836	4.391931	-5.096751
C	2.349781	4.722118	-5.726398
C	1.124752	4.413703	-5.134769
C	1.120583	3.759009	-3.901320
C	2.313539	3.427677	-3.268912
C	3.531263	3.745596	-3.865263
H	4.492080	4.636844	-5.570948
H	2.368604	5.219543	-6.692303
H	0.173552	3.503997	-3.432868
H	2.293953	2.915195	-2.312209
H	4.463302	3.484920	-3.374115
C	-0.177710	4.812077	-5.792940
H	-0.019840	4.964293	-6.865197
H	-0.910518	4.008099	-5.682953
C	-0.192643	7.240682	-5.504972
H	0.448060	7.318443	-6.377702
C	-0.794054	8.444117	-4.957543
S	-1.884780	9.390923	-6.018391
O	-2.313114	10.597177	-5.295437
O	-1.180924	9.558755	-7.298298
C	-3.313646	8.388360	-6.334856
C	-3.299534	7.499741	-7.405894
C	-4.419860	8.489976	-5.496384
C	-4.414022	6.706134	-7.635200
H	-2.431469	7.441246	-8.052290
C	-5.526152	7.691109	-5.745350
H	-4.415063	9.190321	-4.668423
C	-5.542138	6.789080	-6.813652
H	-4.409713	6.011724	-8.469813
H	-6.394033	7.769177	-5.097518
C	-6.754515	5.951646	-7.089578
H	-7.454608	6.495073	-7.733861
H	-6.489937	5.024156	-7.601723
H	-7.284886	5.703645	-6.167528
O	-1.414779	6.585406	-2.500866
P	-1.363737	8.072803	-2.239731

C	-3.020673	8.776009	-2.040630
C	-3.270795	10.150922	-2.034892
C	-4.076406	7.876360	-1.881568
C	-4.569068	10.617165	-1.870778
H	-2.462723	10.864111	-2.160177
C	-5.373498	8.348121	-1.714521
H	-3.873113	6.810811	-1.898064
C	-5.619606	9.716944	-1.710685
H	-4.761531	11.684702	-1.870251
H	-6.191586	7.646125	-1.592351
H	-6.632345	10.085671	-1.584360
C	-0.403251	8.494071	-0.758128
C	-0.944375	9.202918	0.315369
C	0.917229	8.035407	-0.688412
C	-0.169495	9.458245	1.442408
H	-1.969934	9.553668	0.278797
C	1.686287	8.292559	0.438301
H	1.345502	7.466679	-1.508853
C	1.143667	9.006593	1.503760
H	-0.596048	10.009274	2.273887
H	2.709093	7.934175	0.486165
H	1.745902	9.207456	2.383725
N	-0.770379	6.020294	-5.246236
H	-1.154976	5.944286	-4.302846
C	-0.514977	8.996419	-3.609613
H	0.556278	8.896505	-3.401424
H	-0.793808	10.050756	-3.528161

8a

Energy: -2140.38153446

<S**2>=0

Geometry:

C	3.037092	4.873700	-2.476701
C	1.850325	5.015727	-3.189764
C	1.151872	3.893360	-3.632195
C	1.658840	2.626108	-3.341309
C	2.846804	2.481928	-2.633348
C	3.540815	3.607331	-2.198370
H	3.567768	5.757292	-2.136080
H	1.462595	6.008791	-3.394127
H	1.116822	1.743398	-3.670192
H	3.226731	1.489114	-2.414077
H	4.465220	3.497289	-1.640700
C	-0.115914	4.014192	-4.450388
H	0.132412	4.000542	-5.517096
H	-0.744706	3.136723	-4.269245
C	-0.933385	6.237319	-5.037904
H	-0.385493	6.098934	-5.966828
C	-1.579648	7.429678	-4.867961
S	-1.493041	8.532337	-6.218193
O	-1.433409	9.907141	-5.695256
O	-0.438018	8.083532	-7.137639
C	-3.045001	8.403127	-7.090880
C	-3.243749	7.344226	-7.974276
C	-4.048986	9.332403	-6.854288
C	-4.460598	7.227522	-8.625678
H	-2.450467	6.627365	-8.157337
C	-5.266031	9.197929	-7.514913
H	-3.874560	10.157864	-6.173489
C	-5.492183	8.148937	-8.405701
H	-4.615200	6.407443	-9.320618
H	-6.050983	9.925950	-7.334352
C	-6.800733	8.008754	-9.124659
H	-6.666333	8.136600	-10.203500
H	-7.228568	7.013987	-8.969596
H	-7.524707	8.750773	-8.783393
O	-1.810640	6.132560	-1.683513
P	-1.938245	7.598316	-2.045084

C	-3.195797	8.417816	-1.025391
C	-3.324759	9.808216	-0.981915
C	-4.060611	7.613903	-0.282003
C	-4.319960	10.386918	-0.204437
H	-2.645924	10.444627	-1.541948
C	-5.054059	8.198512	0.496555
H	-3.944653	6.535505	-0.313610
C	-5.184716	9.582603	0.533659
H	-4.417588	11.466838	-0.169702
H	-5.724497	7.571990	1.075519
H	-5.959298	10.037877	1.142151
C	-0.386200	8.504863	-1.831052
C	0.460988	8.088426	-0.800187
C	-0.027236	9.589913	-2.633832
C	1.653588	8.760967	-0.566574
H	0.187527	7.233512	-0.189933
C	1.169511	10.258012	-2.394348
H	-0.656126	9.905401	-3.460555
C	2.006582	9.847671	-1.362058
H	2.309182	8.435197	0.234204
H	1.450364	11.097408	-3.021899
H	2.939263	10.371808	-1.180172
N	-0.886127	5.209211	-4.188349
H	-1.276160	5.328069	-3.246806
C	-2.518124	7.829175	-3.767813
H	-2.796055	8.882370	-3.872815
H	-3.455812	7.255943	-3.799533

18. References

1. H. Hofmeister, K. Annen, H. Laurent and R. Wiechert, *Angew. Chem. Int. Ed. Engl.*, 1984, **23**, 727.
2. C. Gommenginger, Y. Zheng, D. Maccarone, I. Ciofini and L. Miesch, *Org. Chem. Front.*, 2023, **10**, 4055.
3. J. Li, C. Ma, D. Xing and W. Hu, *Org. Lett.*, 2019, **21**, 2101.
4. Y. Zheng, B. Moegle, S. Ghosh, A. Perfetto, D. Luise, I. Ciofini and L. Miesch, *Chem. Eur. J.*, 2022, **28**, e202103598.
5. P.-W. Zhu, H.-M. Ma, Y. Li, L.-Z. Miao and J. Zhu, *J. Org. Chem.*, 2023, **88**, 2069.
6. M. Hourtoule and L. Miesch, *Org. Lett.*, 2022, **24**, 3896.
7. C. Gommenginger, M. Hourtoule, M. Menghini and L. Miesch, *Org. Biomol. Chem.*, 2024, **22**, 940.
8. W.-F. Zheng, G.-J. Sun, L. Chen and Q. Kang, *Adv. Synth. Catal.* , 2018, **360**, 1790.
9. G. Luo, Y. Liu, N. Ding, X. Li and Z. Zhao, *ACS Omega*, 2019, **4**, 15312.
10. L. García, J. Sendra, N. Miralles, E. Reyes, J. J. Carbó, J. L. Vicario and E. Fernández, *Chem. Eur. J.*, 2018, **24**, 14059.
11. H.-H. Li, X.-X. Li, Z.-G. Zhao, X. Yuan and C.-Y. Sun, *Org. Biomol. Chem.*, 2017, **15**, 4005.
12. C. Adamo and V. Barone, *J. Chem. Phys.*, 1999, **110**, 6158.
13. K. B. Wiberg, *J. Comput. Chem.*, 1986, **7**, 379.
14. S. Miertuš, E. Scrocco and J. Tomasi, *Chem. Phys.*, 1981, **55**, 117.
15. E. Cancès, B. Mennucci and J. Tomasi, *J. Chem. Phys.*, 1997, **107**, 3032.
